# Effects of monochromatic lights on the growth performance, carcass characteristics, eyeball development, oxidation resistance, and cecal bacteria of Pekin ducks

**DOI:** 10.5713/ajas.20.0215

**Published:** 2020-06-24

**Authors:** Dengke Hua, Fuguang Xue, Hairui Xin, Yiguang Zhao, Yue Wang, Benhai Xiong

**Affiliations:** 1 State Key Laboratory of Animal Nutrition, Institute of Animal Sciences, Chinese Academy of Agricultural Sciences, Beijing 100193, China; 2 Animal Nutrition Group, Department of Animal Sciences, Wageningen University & Research, Wageningen 6708WD, the Netherlands; 3 Jiangxi Province Key Laboratory of Animal Nutrition/Engineering Research Center of Feed Development, Jiangxi Agricultural University, Nanchang 330045, Jiangxi, China

**Keywords:** Duck, Light, Growth Performance, Oxidation Resistance, Eyeball, Cecal Bacteria

## Abstract

**Objective:**

Light is a significant component of housing environment in commercial poultry industry. This study was conducted to investigate whether Pekin ducks perform better under monochromatic lights than under white light with respect to their growth performance, carcass quality, eyeball development, oxidation resistance, and cecal bacterial communities.

**Methods:**

A total of 320 one-day-old male Pekin ducklings were randomly distributed into five rooms with different light treatments, white, red, yellow, green, and blue light. Each room consisted of 4 replicated pens with 16 ducklings per pen.

**Results:**

Blue light significantly decreased fat deposition by decreasing abdominal fat. Long wavelength light, such as red, green, and yellow light, considerably increased the back-to-front eyeball diameter and the red light potentially enlarged the side-to-side eyeball diameter. Besides, the blue light had adverse effects on the oxidation resistance status in terms of increasing the product malonaldehyde of lipid oxidation and decreasing the plasma concentration of total superoxide dismutase. The phyla of *Firmicutes* had the greatest abundance in the green and blue treatments, while *Bacteroidetes* in blue treatment was the least. The genus of *Faecalibacterium* was significantly lower under the red light.

**Conclusion:**

The high risk of cecal health status and decreased anti-oxidation activity were observed under blue light. Red, yellow, and green light might increase the risk of oversized eyeball and cecal illness. Therefore, monochromatic lights compared to white light did not show advantages on the performance of housing ducks, it turns out that the white light is the best light condition for grow-out ducks.

## INTRODUCTION

Light has become a significant microclimate factor in the commercial poultry industry as a critical component of the housing environment. It influenced poultry production by affecting growth development, carcass characteristics, physiological functioning, welfare, behavior, and other parameters [1]. At present, artificial lighting is extensively used to modulate avian growth rate and reproduction. Such lighting is often provided by light-emitting diodes (LEDs), which are well known for their high energy efficiency and availability in differing peak wavelengths [2]. Poultry is sensitive to light. Information with regards to environmental light is received by the poultry brain through photoreception via pathways from the pineal gland and retina [3]. Previous studies have indicated that light colors influenced performance in poultry. Blue, green, or yellow lights were reported to have a positive effect on the productive performance of broilers by increasing growth and improving feed conversion rate, whereas red light had adverse effects [4,5]. Other reports concluded that green light stimulates growth at early ages in broilers, while blue light stimulates the growth of older broilers [6]. Green-blue light has been found to improve the structure of muscles in growing broiler chickens [7]. Previous studies of the effects of light color have mainly focused on chickens, while reports on how colored lighting affects ducks was limited. Compared with chickens, ducks have a preponderance of short-wavelength sensing cones and relatively fewer long-wavelength ones [8], which may cause different reactions of colored light.

The gastrointestinal microbiota has been well studied in recent years and proved to play a significant role in metabolism, nutrition digestion, detoxification of certain compounds, and protection against pathogenic bacteria in avian species [9,10]. The microbial community of the gastrointestinal tract may further interact with intestinal epithelium and ultimately reflect the coevolution of microorganisms with their animal host and the diet adopted by the host [11]. Many factors have been reported to affect the composition of gut microbiota, such as diet, age, and light [12]. Given these factors affecting gut microbiota, it is of scientific interest to study the effect of light color on the gut microbiota of ducks. The cecum is generally accepted as the predominant site for colonization of bacteria in poultry [13]. The effects of light color on the cecal bacteria of ducks have yet to be evaluated. Thus, in the present study, a hypothesis was made that the light colors might influence the composition and diversity of cecal bacteria and subsequently regulated the absorption of feed nutrients, finally affected the production performances of ducks. Based on previous studies on broiler chickens, the present study was designed to compare the effects of white light (composed of multiple monochromatic lights) with those of four types of monochromatic light on the growth, carcass characteristics, eyeball development, oxidation resistance, and cecal bacteria of Pekin ducks to determine the most suitable light color for commercial grow-out ducks.

## MATERIALS AND METHODS

All experimental procedures performed in this study were approved by the Animal Ethics Committee of the Chinese Academy of Agricultural Sciences (Beijing, China). The experimental procedures used in this study were in accordance with the recommendations of the academy’s guidelines for animal research.

### Animals and experimental design

One-day-old Pekin ducklings were obtained from a commercial hatchery (Qianjin Farms, Beijing, China), and reared in an environmentally controlled aviary at the farm of Chinese Academy of Agricultural Sciences. Upon arrival, all ducklings were sexed; 320 male ducklings were selected, group-weighed and randomly distributed into five different treatments. Treatments were arranged in five isolated rooms which were light-proofed to prevent light contamination among treatments. Each treatment had 64 ducklings kept in four replicated pens with 16 ducklings per pen. The pen was made of plastic net with a floor area of 3 m^2^ (duck density, ~0.19 m^2^/duck). Each treatment was provided with one of the following color lights throughout the study: white (390 to 760 nm), red (630 to 780 nm), yellow (570 to 600 nm), green (500 to 570 nm), or blue light (420 to 470 nm). All lights were provided by LEDs. Continuous lighting (24 L:0 D for the first week, 23 L:1 D afterwards) and low light intensity (30 lx at the level of ducks’ head for the first week, 5 lx for the rest weeks) were used.

Living condition adhered to commercial standard, ventilation, temperature and humidity were controlled automatically. The temperature was progressively reduced from 32°C to 21°C. All ducklings had *ad libitum* access to diet and water. The nutrient composition of the experimental diet is shown in Table 1.

### Growth performance, carcass traits, eyeball development, and oxidation resistance

Ducks and feed were weighed on a pen basis at the ages of 1 d, 14 d, 35 d, and 42 d using a scale with an accuracy of 0.01 g and range of 3 kg (LEQI, Shanghai, China). The feed intake (FI), body weight gain (BWG), and FI/BWG (FCR) per duck for the age periods 1 to 14 d, 15 to 35 d, 36 to 42 d, and 1 to 42 d were calculated. All traits were adjusted for mortality.

At 42 d of age, four males from each pen were randomly selected (80 ducks in total), subjected to a 12-hour overnight feed-withdrawal period, and then weighed individually. Thereafter, the electrical water bath stunning process was used for slaughtering, including hanging, water bath stunning (voltage, 2.5 to 10.5 v; current, 0.2 to 0.6 A; time, 3–5 s), slaughter (incised in the neck, <1 cm), bleeding (5 to 6 min), scalding, depilation, viscera and cooling. Carcasses were analyzed by measuring the following parameters: dressed weight, carcass weight, breast meat weight (superficial pectoral plus profound pectoral muscle), leg meat weight (thigh plus drumstick muscle), and abdominal fat weight. The relative percentage of each weight to live weight was then calculated.

The left eyeball was excised, trimmed of extraneous tissue, and weighed. This weight was multiplied by two to obtain an estimate of the total eye weight. The eyeball weight was then divided by BW to get a relative percentage of eyeball weight. The front-to-back and side-to-side diameters of the left eyeball were then measured separately with a precise vernier (Tajima, Japan; range 100 mm; accuracy 0.01 mm).

### Oxidation resistance

At the age of 42 d, four males were randomly selected from each pen and bled via a wing vein to collect 2 mL blood samples. All blood samples were immediately centrifuged at 3,000×g for 20 min at 4°C to 8°C to collect serum. The serum samples were stored at −20°C until analysis for serology parameters: melatonin (Mel), malonaldehyde (MDA), total superoxide dismutase (T-SOD), and glutathione peroxidase (GSH-Px). All parameters were tested via specific enzyme-linked immunosorbent assays (ELISA, Nanjing Jian Cheng Bioengineering Institute, Nanjing, China) using an auto-analyzer (Rainbow GF-D200 automatic auto-analyzer, Gaomi, Shandong, China).

### Cecum sampling and bacteria analysis

On day 42, fresh cecum samples were collected from one bird per replication and stored at −80°C for further analysis on the effect of diets on microbiota profile. Total genome DNA from samples was extracted using the cetyltrimethyl ammonium bromide/sodium dodecyl sulfate method. DNA concentration and purity were monitored on 1% agarose gels. 16S rRNA genes of distinct regions (16SV4) were amplified using specific primer pairs (F: GTGCCAGCMGCC GCGGTAA and R: GGACTACHVGGGTWTCTAAT) with the barcode (GeneAmp 9700, ABI, Foster City, CA, USA). Samples with a bright main strip between 400 to 450 bp were chosen for further experiments. The mixture of polymerized chain reaction (PCR) products was purified with the Qiagen Gel Extraction Kit (Qiagen, Hilden, Germany). Sequencing libraries were generated using TruSeq DNA PCR-Free Sample Preparation Kit (Illumina, San Diego, CA, USA) following the manufacturer’s recommendations and index codes were added. The library quality was assessed on the Qubit 2.0 Fluorometer (Thermo Scientific, Waltham, MA, USA). At last, the library was sequenced on an Illumina HiSeq 4000 platform (Illumina Inc., USA) and 250 bp paired-end reads were generated. Quality filtering on the raw tags was performed under specific filtering conditions to obtain the high-quality clean tags according to the Quantitative Insights Into Microbial Ecology (QIIME, version 1.7.0) quality controlling process. Sequences analysis were subsequently performed by Uparse software (Uparse version 7.0.1001). Sequences with >97% similarity were assigned to the same operational taxonomic unit (OTU). For each representative sequence, the GreenGene Database was used based on the ribosomal database project classifier algorithm to annotate taxonomic information.

### Statistical analysis

All data were tested for normal distribution and homogeneity using the Shapiro-Wilk’s and Levene’s test in SAS 9.2 (SAS Institute Inc., Cary, NC, USA) before further analysis. For the differential analysis of growth performances, carcass performances, one-way analysis of variance (ANOVA) Student-Newman-Keuls test was applied to investigate the differences among treatments. A probability level of p<0.05 was considered statistically significant. The OTU abundances of cecal bacteria were firstly conducted with a transformation of normal distribution using log2, and then one-way ANOVA Student-Newman-Keuls test of SAS 9.2 was applied to analyze the differences of bacteria. Alpha diversity and Beta diversity in our samples were calculated with the Quantitative Insights Into Microbial Ecology (QIIME, version 1.7.0) and displayed with R software (version 3.15.3). The principal coordinates analysis (PCoA) analysis was displayed by the unweighted UniFrac method using R software (version 3.15.3). Spearman correlation coefficients between bacteria communities and performance parameters were assessed using SAS 9.2, and then correlation matrix was created and visualized in a heatmap format using R software (version3.15.3). A significant correlation was considered at p<0.05.

## RESULTS

### Growth performance, carcass traits, eyeball development, and oxidation resistance

The production performance data for the different periods are shown in Table 2. There were no significant differences among different light treatments for the overall stage (1 to 42 d) (p>0.05). However, the FI of the red group showed a decreased trend compared with that of the other groups in the last week (p<0.10). Light color performed no significant effect on BWG in the first two weeks, the middle two weeks or the whole stage (p>0.05), whereas BWG under red light was lower than that under the other colors during the last week (p<0.05).

The influences of monochromatic lights on carcass traits in 42-day-old Pekin ducks are presented in Table 3. No significant difference among treatments was observed for dressed weight percentage, carcass weight percentage, breast meat percentage or leg meat percentage. However, blue light significantly decreased abdominal fat percentage compared with the percentages under the other lights (p<0.05). In comparison with ducks raised under the white, red, yellow, and green light, ducks reared under blue light showed decreases in the abdominal fat percentage of 31%, 35%, 33%, and 29%, respectively.

Effects of monochromatic lights on eyeball development are also presented in Table 3. The long wavelength light, including red, yellow, and green light, considerably increased the back-to-front eyeball diameter of Pekin ducks (p<0.05) in comparison with the diameters obtained under blue light and white light. What is more, the side-to-side diameter was potentially larger under long wavelength light as well (p< 0.10). Interestingly, eyeball weight and its percentage of live weight were not affected by light color.

The effects of light color on the anti-oxidation capacities of Pekin ducks are presented in Table 4. No differences were observed in GSH-Px and Mel concentrations in serum among light groups (p>0.05). However, the MDA content of ducks reared under red- and white- light were lower than those of ducks reared under the other light colors (p<0.05). The ducks reared under blue light had the highest MDA concentration (p<0.05). In addition, blue light significantly decreased the T-SOD content (p<0.05).

### Diversity, richness and composition of bacterial communities in the cecum

The 16S rRNA gene amplicon sequences from cecal contents of 20 samples were conducted to investigate the effects of light color on cecal microbes of Pekin ducks. Taxonomy results of all bacteria are shown in Supplementary Table S1. The average total number of raw tags, after quality filtering, the valid tag number was from 60,000 to 68,000. The average length of a sequence read was about 410 nt. After taxonomy analysis, six phyla, and more than 200 genera were identified in the present study, and all the results were shown in Supplementary Table S1.

Alpha diversity was applied in analyzing the complexity of microbiota diversity. Indexes of Sobs, Chao1, Shannon, Simpson, and abundance-based coverage estimator (ACE) were applied, and results were displayed in Table 5. Based on the table, the Shannon’s index was significantly decreased while Simpson’s index was significantly increased by the green light compared with the other treatments. However, no significant differences were found in the Sobs, ACE, and Chao1 indexes among all treatments.

**Figure 1:**
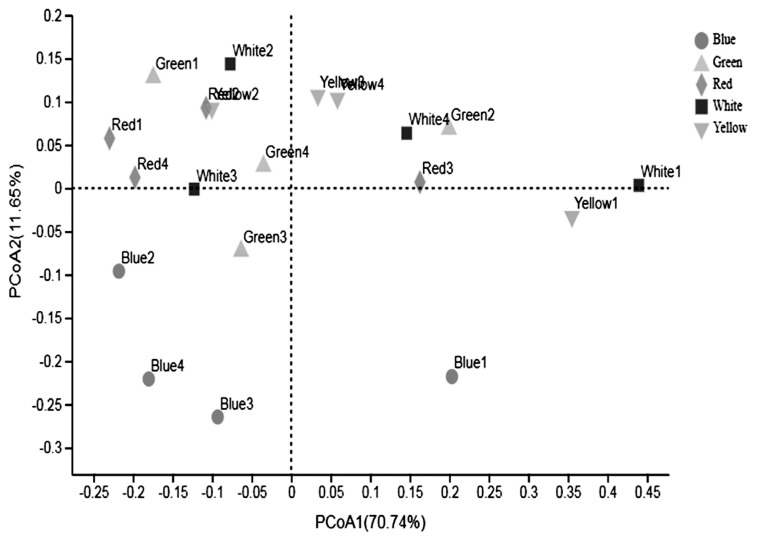
Principal coordinate analysis (PCoA) of bacteria community of Pekin ducks under different light color. PCoa plots were constructed using the unweighted UniFrac method. Blue light, the dark-grey circle; Green light, the light-grey triangle; Red light, the dark-grey diamond; White light, the dark square; Yellow light, the light-grey nabla.

The PCoA was conducted to compare the bacterial profile among the five treatments. As shown in Figure 1, PCoA axes 1 and 2 accounted for 70.74% and 11.65% of the total variation, respectively. Based on the result, bacteria in white light could separate clearly from the other treatments by PCo1. A clear separation was also seen between the green light and the white, yellow light along PCo1. Bacteria in blue treatment could be clearly separated from that in red, white, and yellow light based on PCo1 and PCo2. No significant difference was detected of bacteria in red and yellow light treatment.

Differential analysis of cecal bacteria in phyla and genus level of different light colors was then conducted. Results are shown in Table 6, 7, respectively. Based on the results in Table 6, *Firmicutes* was significantly increased while *Bacteroidetes* was significantly decreased by the monochromatic light treatments compared with white light treatment. *Firmicutes* performed the most abundance in the green- and blue- treatments, while *Bacteroidetes* performed the least in Blue treatment among the five treatments. No significant differences were detected of *Bacteroidetes* in the red, yellow, and green treatments. Besides, *Tenericutes* was found significant suppressed in red treatment while performed the most abundances in green- and white- treatment. There was no difference in other phyla among different light treatments. As for the top 20 genera (Table 7), *Faecalibacterium* performed the most abundant among all genera and significantly decreased in red treatment. No significant differences were detected on the other phyla among the five light treatments.

### Correlation between cecal bacteria and other parameters

At last, the most abundant genera were selected for the correlation analysis with the production performance, carcass, eyeball growth, and oxidation resistance parameters. Results are shown in Supplementary Figure S1. As shown in Supplementary Figure S1, the most abundant genus *Faecalibacterium* was positively correlated with BWG and eviscerated weight, but negatively correlated with eyeball weight and FCR. The relative abundance of genus *Lactobacillus* was positively correlated with Mel and GSH-Px, while negatively correlated with T-SOD. The FI was positively correlated with the genus *Clostridiales*. Meanwhile, *Ruminiclostridium* was negatively correlated with BWG.

## DISCUSSION

### Effects of light color on growth performance and carcass traits

Previous studies about the effects of light color in poultry production have mainly focused on galliform birds, such as broilers or turkeys, with few studies worked on ducks. In the present study, the results indicated that light color had little effect on production performance in Pekin ducks in the overall stage. These findings are in agreement with some previous studies on broilers [6,14], which indicated that light color treatments might not perform significant work in the feeding period of about 42 d. Interestingly, Campbell et al [15] found decreased BW in ducks under blue light compared with that under white and red lights. Whereas, they provided a high light intensity (25 lx) in their study, which might stimulate duck activity and consequently led to differences in production performance. In the present study, low light intensity of 5 lx was applied based on the previous studies [16], and this might be the reason why no effect of light color on overall production performance was found, while more studies are still needed to prove the interactive effect of light color and intensity on ducks.

The light color treatments did not differentially affect most carcass traits, except that blue light decreased the percentage of abdominal fat. In the previous study, ducks raised under blue light were observed to do more activities, including preening and foraging, compared to ducks raised under red light [15]. Another study found that ducks preferred blue-colored enrichment devices than red- and white- ones, and showed more feather picking [17]. Thus, the decreased fat deposition of ducks under blue light in the present study might be due to their increased activity.

Besides, according to the correlation data (Supplementary Figure S1), The genus of *Faecalibacterium* was positively correlated with BWG and eviscerated weight, but negatively correlated with FCR. Although the BWG of the whole period did not differ among treatments, the BWG in the fattening period was in the same tendency with the genus *Faecalibacterium*. However, further work is still needed to confirm these correlations and determine their causality, as recommended by other researchers [18].

### Effects of light color on eyeball growth

The present study indicated that long-wavelength light, such as red, green, and yellow light, considerably increased the back-to-front eyeball diameter and the red light potentially increased the side-to-side eyeball diameter of Pekin ducks, which mean long-wavelength light would increase the risk to enlarge eyeball size. Compared with the human eyes, the bird’s eye possesses more photosensitive pigments associated with cone cells which are responsible for color vision and are more sensitive to broader spectrums [19]. Basically, the retina contains four to five kinds of cones for visualizing color, long-wavelength sensing, medium-wavelength sensing, and two short-wavelength sensing. Previous studies demonstrated that shorebirds, like ducks, contained more cones for short-wavelength sensing (blue-sensitive) than that for long-wavelength sensing (red-sensitive) [20]. The evolutionary selection for color-sensitive vision probably resulted in the difference in eyeball growth of ducks [21]. A larger eye might cause pressure on the optic nerve which was located at the caudal aspect of the eyeball and then induced nerve injury, and the injured nerve would increase the release of inflammatory mediators which could lead to hyperalgesia, a painful condition [22]. In summary, according to the present results, long-wavelength monochromatic light including red, green and yellow light, especially red light, had the potential to cause enlarged eyeballs of the ducks thereby might affect their welfare.

### Effects of light color on oxidative stress resistance

Oxidative stress is related to the pathogenesis of some chronic diseases and plays a paramount role in the ageing process. Of the many biological targets of oxidative stress, lipids are the most involved class of biomolecules. Lipid oxidation leads to the production of many secondary products. Among these products, MDA is the principal and most studied product of polyunsaturated fatty acid peroxidation [23]. T-SOD is another indicator of oxidative status. Superoxide is degraded into hydrogen peroxide by SOD and subsequently catalyzed to water by a series of enzymes [24]. In this study, the increased plasma MDA and the decreased T-SOD in blue-light treatment suggested that blue light might promoted lipid peroxidation in ducks and had adverse effects on the antioxidant capacity. In addition, the ratio of *Bacteroidetes* to *Firmicutes* considerably decreased under the blue light. Previous studies had proved that the *Bacteroidetes* to *Firmicutes* ratio affected the ability to absorb nutrient and were strongly correlated with lipid metabolism [25]. The mRNA levels of lipogenic enzymes were increased while the ratio of *Bacteroidetes* to *Firmicutes* decreased [11]. Furthermore, lipid accumulation leads to the production of many secondary products including MDA [23]. The changes of the cecal bacterial communities might contribute to the difference in the MDA.

### Effects of light color on cecal bacterial communities

In the present study, the cecal bacterial communities consisted of three dominant bacterial phyla: *Firmicutes*, *Bacteroidetes*, and *Proteobacteria*, which agreed with previous research [26]. The microbiota composition is highly related to gut health and thereby related to the host health status. The present study demonstrated that the blue- and green- light increased the amount of *Firmicutes*, but decreased the amount of *Bacteroidetes*, compared to white light; consequently, the *Firmicutes* to *Bacteroidetes* ratio was increased by the blue- and green- light. Higher *Firmicutes*/*Bacteroidetes* ratio has been linked with chicken obesity [27]. Moreover, previous studies reported that the *Firmicutes* to *Bacteroidetes* ratio was positively correlated with the farm *Campylobacter* counts [27], which could contaminate chicken meat and cause diarrheal illness among human. Besides, both *Firmicutes* and *Bacteriodetes* have been reported to be associated with short-chain fatty acid metabolism. To be more specifically, *Firmicutes* contributed to butyrate and propionate synthesis, whereas *Bacteroidetes* primarily synthesized propionate [28]. Butyrate was one of the significant short-chain fatty acids, which provided ~70% energy for normal colonic epithelial cells, meanwhile had the capacity to increase the thickness of the mucus layer in order to prevent the invasion of pathogenic bacteria and maintain gut health [29]. The genus of *Faecalibacterium* was one of the main butyrate-producing genera [30]. The present study illustrated that the red light decreased the abundance of *Faecalibacterum*, which might improve the potential to restrain the butyrate production and thereby might influence cecal health status. As for the phyla *Tenericutes*, very few studies about its function in poultry cecum were found, further work would be required. In summary, the blue- and green- light increased the *Firmicutes*/*Bacteroidetes* ratio, which might improve the risk of *Campylobacter* infection; while the red light might damage the cecal health status by improving the amount of butyrate-producing genus of *Faecalibacterium*.

## CONCLUSION

Different from chickens, housing ducks exposed to monochromatic lights did not perform better in most performance tested in the present study, compared to ducks under white light. Although ducks reared under blue light had lower fat deposition, the high risk of cecal health status and decreased anti-oxidation activity were also observed. Red light may increase the risk of the oversized eyeball and cecal illness. Therefore, the present study suggests that the white light should be the best choice for grow-out ducks in a commercial setting. In order to elucidate more comprehensive knowledge about the influence of monochromatic light, it would be valuable to investigate their interactions with light intensity and photoperiod.

## Figures and Tables

**Table 1:** Composition and nutrient levels of basal diet (as-fed basis)

Items	1 to 14 d of age	15 to 42 d of age
Ingredients (g/kg)		
Corn	628.0	716.0
Soybean meal	334.0	244.0
Dicalcium Phosphate	15.0	15.0
Limestone	10.0	12.0
Sodium chloride	3.0	3.0
Premix^1)^	10.0	10.0
Total	1,000	1,000
Nutrient composition (g/kg)		
ME (MJ/kg)	12.03	12.29
CP	213.6	174.9
Calcium	8.3	8.7
Available phosphorus	6.1	6.0
Lysine	11.4	9.0
Methionine	4.2	3.9
Methionine+cysteine	7.7	6.9

ME, metabolizable energy; CP, crude protein.

1) Provided the following quantities per kg of premix: Iron (from FeSO_4_·7H_2_O), 90 mg; Copper (from CuSO_4_·5H_2_O), 8.12 mg; Zinc (from ZnSO_4_·7H_2_O), 89.7 mg; Manganese (from MnSO_4_·H_2_O), 101.76 mg; Selenium (from Na_2_SeO_3_), 0.3 mg; Iodine, 0.8 mg; Vitamin A, 10,350 IU; Vitamin D_3_, 2,760 IU; Vitamin E, 27.6 IU; Vitamin K_3_, 3.45 mg; Vitamin B_12_, 0.03 mg; Riboflavin, 4.6 mg; Thiamine, 2.76 mg; Calcium pantothenate, 12.65 mg; Nicotinamide 29.9 mg; Pyridoxine, 4.5 mg; Biotin, 0.18 mg; Folic acid, 1.61 mg; Choline chloride, 1,000 mg.

**Table 2:** Effects of light color on the production performance of Pekin ducks

Items	Stage d	Treatment					SEM	p-value^1)^

		White	Red	Yellow	Green	Blue		
BW (g)	1	54.8	55.9	54.6	54.8	55.3	0.255	0.834
	14	635.2	626.3	660.7	661.5	633.3	8.251	0.498
	35	2,178.9	2,116.7	2,212.9	2,118.6	2,168.4	20.641	0.579
	42	2,570.5	2,450.5	2,601.8	2,509.6	2,567.4	30.038	0.901
FI (g)	1–14	887.1	852.2	912.6	898.2	857.1	14.930	0.690
	15–35	4,098.8	4,060.6	4,238.6	4,005.9	4,094.7	51.421	0.779
	36–42	1,258.9	1,122.9	1,286.3	1,266.3	1,268.8	21.038	0.082
	1–42	6,244.7	6,035.7	6,437.6	6,170.3	6,220.6	73.031	0.602
BWG (g)	1–14	580.4	570.4	606.1	606.7	577.9	9.669	0.735
	15–35	1,543.7	1,490.4	1,552.2	1,457.1	1,535.2	25.767	0.832
	36–42	391.7^a^	333.9^b^	388.9^a^	391.0^a^	399.0^a^	7.207	0.01
	1–42	2,515.7	2,394.6	2,547.2	2,454.8	2,512.1	30.494	0.598
FCR	1–14	1.53	1.49	1.5	1.48	1.48	0.01	0.611
	15–35	2.66	2.73	2.74	2.77	2.67	0.025	0.635
	36–42	3.22	3.37	3.31	3.25	3.18	0.037	0.484
	1–42	2.48	2.52	2.53	2.52	2.48	0.013	0.599

SEM, standard error of the mean; BW, body weight; FI, feed intake; BWG, body weight gain; FCR, feed intake/body weight gain.

1) Comparisons among groups based on a multivariate analysis of variance.

a,b Different letters differ significantly within a row (p<0.05).

**Table 3:** Effects of light color on the carcass performance and eyeball of Pekin ducks

Items	Treatment					SEM	p-value^1)^

	White	Red	Yellow	Green	Blue		
Dressed weight (%)	85	85.49	84.49	85.75	85.92	0.327	0.644
Carcass weight (%)	72.99	71.82	71.9	72.79	72.96	0.294	0.684
Breast meat (%)	14.56	14.51	14.84	14.52	14.97	0.242	0.872
Leg meat (%)	12.03	11.96	11.75	11.64	12.26	0.178	0.841
Abdominal fat (%)	1.79^a^	1.83^a^	1.81^a^	1.77^a^	1.48^b^	0.038	0.037
Eyeball weight (g)	1.65	1.76	1.72	1.71	1.72	0.018	0.471
Side-to-side diameter (mm)	15.57	16.27	16.03	16.06	15.91	0.08	0.088
Back-to-front diameter (mm)	11.41^b^	12.01^a^	12.01^a^	12.17^a^	11.54^b^	0.067	0.000
Eyeball-to-BW ratio (%)	0.14	0.15	0.14	0.14	0.14	0.003	0.357

SEM, standard error of the mean; BW, body weight.

1) Comparisons among groups based on a multivariate analysis of variance.

a,b Different letters differ significantly within a row (p<0.05).

**Table 4:** Effects of light color on the antioxidant capacity of Pekin ducks

Items	Treatment					SEM	p-value^1)^

	White	Red	Yellow	Green	Blue		
MDA (mmol/mL)	2.74^c^	2.81^c^	3.61^b^	3.94^b^	4.53^a^	0.118	0.000
T-SOD (U/mL)	58.22^a^	51.76^a^	52.15^a^	60.02^a^	40.38^b^	1.037	0.000
GSH-Px (U/L)	52.73	54	52.85	57.81	52.55	0.846	0.245
Mel (pg/mL)	168.36	164.73	163.81	176.82	168.41	3.540	0.803

SEM, standard error of the mean; MDA, malonaldehyde; T-SOD, total superoxide dismutase; GSH-Px, glutathione peroxidase; Mel, melatonin.

1) Comparisons among groups based on a multivariate analysis of variance.

a–c Different letters differ significantly within a row (p<0.05).

**Table 5:** Effects of light color on the α-diversity of cecal bacteria of Pekin ducks

Items	Treatment					SEM	p-value^1)^

	White	Red	Yellow	Green	Blue		
Sobs	489.0	410.0	444.3	446.3	456.3	9.005	0.0730
Shannon	4.48^a^	3.95^c^	4.13^b^	3.66^c^	4.01^b^	0.069	<0.001
Simpson	0.03^c^	0.06^b^	0.05^b^	0.10^a^	0.05^b^	0.006	<0.001
ACE	532.9	456.2	490.7	492.8	510.8	10.149	0.177
Chao	539.9	474.4	496.9	498.4	523.7	10.573	0.345

SEM, standard error of the mean; ACE, abundance-based coverage estimator.

1) Comparisons among groups based on a multivariate analysis of variance.

a–c Different letters differed significantly within a row (p<0.05).

**Table 6:** Effects of light color on the cecal bacteria of Pekin ducks at the level of phyla

Items	Treatment					SEM	p-value^1)^

	White	Red	Yellow	Green	Blue		
*Firmicutes*	15.31^c^	15.48^b^	15.48^b^	15.63^a^	15.73^a^	0.036	<0.001
*Bacteroidetes*	13.63^a^	12.66^b^	12.50^b^	12.85^b^	11.71^c^	0.179	0.004
*Proteobacteria*	11.40	10.50	10.74	11.17	11.21	0.207	0.676
*Tenericutes*	10.28^a^	8.019^c^	9.43^b^	10.57^a^	9.18^b^	0.28	0.015
*Cyanobacteria*	4.45	7.49	6.73	7.09	6.75	0.64	0.621
*Actinobacteria*	6.73	7.33	5.02	6.29	5.85	0.289	0.100

All values were firstly transformed using log2.

SEM, standard error of the mean.

1) Comparisons among groups based on a multivariate analysis of variance.

a–c Different letters differed significantly within a row (p<0.05).

**Table 7:** Effects of light color on the cecal bacteria of Pekin ducks at the genus level

Items	Treatment					SEM	p-value^1)^

	White	Red	Yellow	Blue	Green		
*g__Faecalibacterium*	14.28^a^	12.87^c^	13.33^b^	13.58^b^	13.32^b^	0.139	0.008
*g__Alistipes*	12.54	13.22	11.7	12.77	11.95	0.249	0.321
*g__Lachnospiraceae*	11.42	11.96	11.64	11.78	12.03	0.136	0.668
*g__Lactobacillus*	10.98	10.03	10.10	10.39	12.06	0.244	0.207
*g__Clostridiales*	9.95	8.75	11.15	10.50	9.46	0.360	0.267
*g__Escherichia-Shigella*	10.51	9.52	7.73	8.06	11.56	0.618	0.248
*g__Ruminococcaceae*	10.61	10.40	9.98	10.50	10.83	0.141	0.443
*g__Butyricicoccus*	9.30	9.07	10.45	10.44	9.81	0.303	0.513
*g__Subdoligranulum*	9.85	9.41	8.81	8.95	9.40	0.381	0.936
*g__Anaerotruncus*	10.23	10.13	10.09	10.34	10.44	0.125	0.925
*g__Blautia*	9.13	9.52	9.34	10.35	10.27	0.260	0.494
*g__Lachnoclostridium*	9.62	10.34	10.12	9.79	9.96	0.120	0.382
*g__Ruminiclostridium*	8.82	10.55	9.60	9.68	9.66	0.199	0.088
*g__Eisenbergiella*	8.72	9.64	10.51	9.75	9.72	0.217	0.128
*g__Shuttleworthia*	9.06	8.84	9.06	9.48	10.17	0.181	0.132
*g__Erysipelatoclostridium*	7.98	9.03	8.74	10.02	8.45	0.298	0.269
*g__Salmonella*	8.53	8.77	8.93	9.75	9.39	0.173	0.160
*g__Mollicutes_RF9*	8.20	10.12	7.71	8.78	8.01	0.335	0.156
*g__Parasutterella*	9.34	7.64	9.00	9.15	8.59	0.283	0.352
*g__coprostanoligenes*	8.21	9.27	9.17	8.99	8.35	0.279	0.702
Others	11.11	12.34	11.93	12.07	11.93	0.155	0.115

All values were firstly transformed using log2.

SEM, standard error of the mean.

1) Comparisons among groups based on a multivariate analysis of variance.

a–c Different letters differed significantly within a row (p<0.05).
