# Supplementary material for: Effects of monochromatic lights on the growth performance, carcass characteristics, eyeball development, oxidation resistance, and cecal bacteria of Pekin ducks
Source: Anim Biosci. 2020 Jun 24;34(5):931–40. doi: 10.5713/ajas.20.0215 (PMC8100473; doi:10.5713/ajas.20.0215)
Supplement: Supplementary file 1 [file ajas-20-0215-suppl.pdf]

Additional file 1. All cecal bacterial communities of Pekin ducks under different light color

Phylum	Class	Order	Family	Genus	Species	OTU	White1	White2	White3	White4
Firmicutes	Clostridia	Clostridiales	Ruminococcaceae	Faecalibacterium	uncultured_bacterium_g__Faecalibacterium	OTU705	17744	7428	4943	14936
Bacteroidetes	Bacteroidia	Bacteroidales	Rikenellaceae	Alistipes	gut_metagenome_g__Alistipes	OTU287	399	14671	5164	3738
Firmicutes	Clostridia	Clostridiales	Lachnospiraceae	[Ruminococcus]_torques_group	uncultured_Clostridiales_bacterium_g__[Ruminococcus]_torques_group	OTU361	2120	3885	4676	1798
Proteobacteria	Gammaproteobacteria	Enterobacteriales	Enterobacteriaceae	Escherichia-Shigella	Escherichia_coli_g__Escherichia-Shigella	OTU589	4625	1733	2269	2648
Firmicutes	Clostridia	Clostridiales	Ruminococcaceae	Faecalibacterium	bacterium_ic1379	OTU50	9	2219	3057	1822
Bacteroidetes	Bacteroidia	Bacteroidales	Rikenellaceae	Alistipes	unclassified_g__Alistipes	OTU501	283	1678	2119	1365
Firmicutes	Bacilli	Lactobacillales	Lactobacillaceae	Lactobacillus	unclassified_g__Lactobacillus	OTU445	781	691	672	605
Firmicutes	Clostridia	Clostridiales	Ruminococcaceae	Subdoligranulum	uncultured_bacterium_g__Subdoligranulum	OTU816	1264	18	984	365
Firmicutes	Clostridia	Clostridiales	Lachnospiraceae	unclassified_f__Lachnospiraceae	unclassified_f__Lachnospiraceae	OTU581	994	833	298	1208
Firmicutes	Clostridia	Clostridiales	Lachnospiraceae	Blautia	unclassified_g__Blautia	OTU78	15	473	392	1071
Firmicutes	Clostridia	Clostridiales	Lachnospiraceae	Eisenbergiella	uncultured_bacterium_g__Eisenbergiella	OTU48	73	2420	1211	1232
Firmicutes	Clostridia	Clostridiales	Ruminococcaceae	Anaerotruncus	unclassified_g__Anaerotruncus	OTU213	351	564	428	1029
Firmicutes	Clostridia	Clostridiales	Lachnospiraceae	unclassified_f__Lachnospiraceae	unclassified_f__Lachnospiraceae	OTU710	627	264	197	750
Firmicutes	Bacilli	Lactobacillales	Lactobacillaceae	Lactobacillus	Lactobacillus_salivarius	OTU775	1552	133	114	253
Firmicutes	Clostridia	Clostridiales	Lachnospiraceae	Shuttleworthia	uncultured_bacterium_g__Shuttleworthia	OTU683	453	727	119	1107
Firmicutes	Clostridia	Clostridiales	Ruminococcaceae	Butyricicoccus	uncultured_bacterium_g__Butyricicoccus	OTU616	46	618	1192	245
Firmicutes	Clostridia	Clostridiales	Lachnospiraceae	[Ruminococcus]_torques_group	unclassified_g__[Ruminococcus]_torques_group	OTU196	1785	33	18	9
Firmicutes	Clostridia	Clostridiales	Lachnospiraceae	Lachnoclostridium	unclassified_g__Lachnoclostridium	OTU527	455	713	617	582
Firmicutes	Clostridia	Clostridiales	Ruminococcaceae	Ruminiclostridium_9	unclassified_g__Ruminiclostridium_9	OTU174	85	258	1099	560
Firmicutes	Clostridia	Clostridiales	Ruminococcaceae	Subdoligranulum	unclassified_g__Subdoligranulum	OTU88	822	257	332	647
Firmicutes	Clostridia	Clostridiales	Ruminococcaceae	Ruminococcaceae_UCG-014	unclassified_g__Ruminococcaceae_UCG-014	OTU712	108	196	119	383
Proteobacteria	Betaproteobacteria	Burkholderiales	Alcaligenaceae	Parasutterella	uncultured_organism_g__Parasutterella	OTU692	2403	64	385	195
Firmicutes	Clostridia	Clostridiales	Ruminococcaceae	Faecalibacterium	uncultured_bacterium_g__Faecalibacterium	OTU122	23	37	51	6
Firmicutes	Clostridia	Clostridiales	Clostridiales_vadinBB6	norank_f__Clostridiales_vadinBB60_	uncultured_bacterium_g__norank_f__Clostridiales_vadinBB60_group	OTU151	2	2249	260	37
Firmicutes	Clostridia	Clostridiales	Ruminococcaceae	Faecalibacterium	uncultured_bacterium_g__Faecalibacterium	OTU70	3	114	189	469
Firmicutes	Clostridia	Clostridiales	Lachnospiraceae	unclassified_f__Lachnospiraceae	unclassified_f__Lachnospiraceae	OTU711	92	422	435	440
Firmicutes	Bacilli	Lactobacillales	Lactobacillaceae	Lactobacillus	Lactobacillus_vaginalis	OTU489	4	4	208	176
Firmicutes	Clostridia	Clostridiales	Lachnospiraceae	Sellimonas	unclassified_g__Sellimonas	OTU476	389	145	296	522
Firmicutes	Clostridia	Clostridiales	Lachnospiraceae	unclassified_f__Lachnospiraceae	unclassified_f__Lachnospiraceae	OTU179	211	776	554	505
Firmicutes	Clostridia	Clostridiales	Ruminococcaceae	norank_f__Ruminococcaceae	unclassified_g__norank_f__Ruminococcaceae	OTU191	1709	127	24	120
Firmicutes	Erysipelotrichia	Erysipelotrichales	Erysipelotrichaceae	Erysipelatoclostridium	bacterium_ic1391	OTU561	268	264	232	336
Firmicutes	Clostridia	Clostridiales	Ruminococcaceae	[Eubacterium]_coprostanoligenes_g	unclassified_g__[Eubacterium]_coprostanoligenes_group	OTU13	43	952	612	1007
Firmicutes	Bacilli	Lactobacillales	Enterococcaceae	Enterococcus	Enterococcus_cecorum	OTU562	33	0	15	123
Firmicutes	Clostridia	Clostridiales	Lachnospiraceae	unclassified_f__Lachnospiraceae	unclassified_f__Lachnospiraceae	OTU484	521	177	139	123
Firmicutes	Clostridia	Clostridiales	Ruminococcaceae	unclassified_f__Ruminococcaceae	unclassified_f__Ruminococcaceae	OTU478	19	325	949	491
Firmicutes	Clostridia	Clostridiales	Lachnospiraceae	Lachnoclostridium	Clostridium_sp._BR31	OTU836	268	376	310	249
Firmicutes	Clostridia	Clostridiales	Ruminococcaceae	norank_f__Ruminococcaceae	Clostridiales_bacterium_24-4c	OTU743	5	4	155	124
Firmicutes	Bacilli	Bacillales	Bacillaceae	unclassified_f__Bacillaceae	unclassified_f__Bacillaceae	OTU851	0	84	58	11
Firmicutes	Clostridia	Clostridiales	Lachnospiraceae	[Ruminococcus]_torques_group	uncultured_Clostridiales_bacterium_g__[Ruminococcus]_torques_group	OTU774	35	22	5	53
Firmicutes	Clostridia	Clostridiales	Lachnospiraceae	[Eubacterium]_hallii_group	uncultured_bacterium_g__[Eubacterium]_hallii_group	OTU202	51	425	44	181
Firmicutes	Clostridia	Clostridiales	Lachnospiraceae	Anaerostipes	Anaerostipes_butyraticus	OTU189	599	37	603	14
Firmicutes	Clostridia	Clostridiales	Ruminococcaceae	Ruminococcaceae_UCG-014	uncultured_bacterium_g__Ruminococcaceae_UCG-014	OTU578	4	140	111	555
Firmicutes	Clostridia	Clostridiales	Lachnospiraceae	Sellimonas	bacterium_P1C11	OTU218	140	111	206	220
Firmicutes	Clostridia	Clostridiales	Christensenellaceae	Christensenellaceae_R-7_group	unclassified_g__Christensenellaceae_R-7_group	OTU709	0	8	264	426
Firmicutes	Clostridia	Clostridiales	Ruminococcaceae	Butyricicoccus	unclassified_g__Butyricicoccus	OTU33	5	5	1554	37
Bacteroidetes	Bacteroidia	Bacteroidales	Bacteroidaceae	Bacteroides	Bacteroides_acidifaciens	OTU226	0	0	0	0
Firmicutes	Clostridia	Clostridiales	Ruminococcaceae	Ruminococcaceae_UCG-014	unclassified_g__Ruminococcaceae_UCG-014	OTU65	41	139	111	255
Firmicutes	Clostridia	Clostridiales	Lachnospiraceae	Lachnoclostridium	unclassified_g__Lachnoclostridium	OTU469	128	215	245	180
Firmicutes	Clostridia	Clostridiales	Ruminococcaceae	Ruminococcaceae_UCG-005	uncultured_organism_g__Ruminococcaceae_UCG-005	OTU690	1	65	198	81
Firmicutes	Erysipelotrichia	Erysipelotrichales	Erysipelotrichaceae	Erysipelatoclostridium	[Clostridium]_spiroforme	OTU625	12	170	151	157
Tenericutes	Mollicutes	Mollicutes_RF9	norank_o__Mollicutes	norank_o__Mollicutes_RF9	uncultured_bacterium_g__norank_o__Mollicutes_RF9	OTU588	28	214	1245	354
Firmicutes	Clostridia	Clostridiales	Lachnospiraceae	Tyzzerella	uncultured_organism_g__Tyzzerella	OTU795	79	128	322	81
Firmicutes	Clostridia	Clostridiales	Ruminococcaceae	Ruminiclostridium	uncultured_bacterium_g__Ruminiclostridium	OTU368	0	759	712	198
Firmicutes	Clostridia	Clostridiales	Ruminococcaceae	Butyricicoccus	Butyricicoccus_pullicaecorum_1.2	OTU769	0	1	48	92
Firmicutes	Clostridia	Clostridiales	Ruminococcaceae	Ruminococcaceae_UCG-014	uncultured_bacterium_g__Ruminococcaceae_UCG-014	OTU346	18	381	422	167
Firmicutes	Clostridia	Clostridiales	Lachnospiraceae	norank_f__Lachnospiraceae	Clostridiales_bacterium_CHKCI001	OTU738	6	513	343	112
Proteobacteria	Gammaproteobacteria	Pseudomonadales	Pseudomonadaceae	Pseudomonas	Pseudomonas_brenneri	OTU580	18	418	656	55
Firmicutes	Clostridia	Clostridiales	Ruminococcaceae	norank_f__Ruminococcaceae	unclassified_g__norank_f__Ruminococcaceae	OTU460	11	209	263	29
Firmicutes	Clostridia	Clostridiales	Ruminococcaceae	Ruminococcaceae_UCG-014	unidentified_rumen_bacterium_JW32	OTU565	1	231	482	49
Firmicutes	Clostridia	Clostridiales	Ruminococcaceae	Ruminococcaceae_UCG-014	uncultured_rumen_bacterium_g__Ruminococcaceae_UCG-014	OTU813	3	5	0	2
Firmicutes	Clostridia	Clostridiales	Lachnospiraceae	Lachnospiraceae_NK4A136_group	unclassified_g__Lachnospiraceae_NK4A136_group	OTU193	0	178	446	465
Firmicutes	Clostridia	Clostridiales	Clostridiales_vadinBB6	norank_f__Clostridiales_vadinBB60_	uncultured_bacterium_g__norank_f__Clostridiales_vadinBB60_group	OTU573	111	814	118	324
Firmicutes	Clostridia	Clostridiales	Lachnospiraceae	Coprococcus_1	uncultured_organism_g__Coprococcus_1	OTU591	103	134	263	54
Firmicutes	Clostridia	Clostridiales	Lachnospiraceae	unclassified_f__Lachnospiraceae	unclassified_f__Lachnospiraceae	OTU515	104	1013	93	220
Firmicutes	Clostridia	Clostridiales	Ruminococcaceae	Ruminococcaceae_UCG-014	uncultured_organism_g__Ruminococcaceae_UCG-014	OTU457	197	439	17	172
Firmicutes	Clostridia	Clostridiales	Lachnospiraceae	Blautia	Blautia_hydrogenotrophica	OTU718	250	389	131	118
Firmicutes	Clostridia	Clostridiales	Ruminococcaceae	norank_f__Ruminococcaceae	unclassified_g__norank_f__Ruminococcaceae	OTU735	9	119	343	149
Firmicutes	Clostridia	Clostridiales	Peptostreptococcacea	Romboutsia	uncultured_bacterium_g__Romboutsia	OTU79	1	102	4	1
Firmicutes	Clostridia	Clostridiales	Ruminococcaceae	Anaerotruncus	uncultured_bacterium_g__Anaerotruncus	OTU499	180	71	391	714
Firmicutes	Clostridia	Clostridiales	Ruminococcaceae	Ruminococcaceae_UCG-014	uncultured_bacterium_g__Ruminococcaceae_UCG-014	OTU7	6	374	279	262
Firmicutes	Clostridia	Clostridiales	Ruminococcaceae	Ruminococcaceae_UCG-014	unclassified_g__Ruminococcaceae_UCG-014	OTU25	4	290	165	79
Firmicutes	Clostridia	Clostridiales	Ruminococcaceae	[Eubacterium]_coprostanoligenes_g	gut_metagenome_g__[Eubacterium]_coprostanoligenes_group	OTU214	241	4	73	187
Firmicutes	Clostridia	Clostridiales	Ruminococcaceae	Ruminococcaceae_UCG-005	unclassified_g__Ruminococcaceae_UCG-005	OTU160	1	404	248	55
Firmicutes	Clostridia	Clostridiales	Ruminococcaceae	Flavonifractor	uncultured_bacterium_g__Flavonifractor	OTU716	29	202	65	53
Firmicutes	Clostridia	Clostridiales	Ruminococcaceae	Ruminococcaceae_UCG-014	uncultured_bacterium_g__Ruminococcaceae_UCG-014	OTU691	248	0	26	89
Firmicutes	Clostridia	Clostridiales	Ruminococcaceae	Ruminococcaceae_UCG-005	uncultured_prokaryote_g__Ruminococcaceae_UCG-005	OTU504	0	0	45	5
Firmicutes	Clostridia	Clostridiales	Ruminococcaceae	unclassified_f__Ruminococcaceae	unclassified_f__Ruminococcaceae	OTU496	5	38	89	26
Firmicutes	Clostridia	Clostridiales	Ruminococcaceae	Butyricicoccus	unclassified_g__Butyricicoccus	OTU480	6	5	44	4
Firmicutes	Clostridia	Clostridiales	Lachnospiraceae	unclassified_f__Lachnospiraceae	unclassified_f__Lachnospiraceae	OTU533	8	293	316	149
Firmicutes	Bacilli	Lactobacillales	Streptococcaceae	Streptococcus	Streptococcus_gallolyticus_subsp._macedonicus	OTU474	0	5	3	0
Firmicutes	Clostridia	Clostridiales	Ruminococcaceae	norank_f__Ruminococcaceae	Ruminococcaceae_bacterium_AM2	OTU242	5	31	122	141
Firmicutes	Clostridia	Clostridiales	Clostridiales_vadinBB6	norank_f__Clostridiales_vadinBB60_	uncultured_organism_g__norank_f__Clostridiales_vadinBB60_group	OTU150	0	0	8	0
Firmicutes	Clostridia	Clostridiales	Lachnospiraceae	Shuttleworthia	uncultured_bacterium_g__Shuttleworthia	OTU507	1	72	195	63
Firmicutes	Clostridia	Clostridiales	Lachnospiraceae	Blautia	uncultured_bacterium_g__Blautia	OTU450	1	14	78	108
Firmicutes	Clostridia	Clostridiales	Ruminococcaceae	Ruminococcaceae_UCG-014	uncultured_rumen_bacterium_g__Ruminococcaceae_UCG-014	OTU230	1	0	240	21
Firmicutes	Clostridia	Clostridiales	Ruminococcaceae	Ruminococcaceae_UCG-004	unclassified_g__Ruminococcaceae_UCG-004	OTU355	96	142	168	75
Firmicutes	Clostridia	Clostridiales	Ruminococcaceae	norank_f__Ruminococcaceae	human_gut_metagenome_g__norank_f__Ruminococcaceae	OTU623	68	136	132	157
Firmicutes	Clostridia	Clostridiales	Lachnospiraceae	[Ruminococcus]_gauvreauii_group	bacterium_ic1296	OTU564	91	54	88	91
Firmicutes	Clostridia	Clostridiales	Ruminococcaceae	Anaerofilum	uncultured_bacterium_g__Anaerofilum	OTU123	0	116	178	41
Firmicutes	Clostridia	Clostridiales	Lachnospiraceae	unclassified_f__Lachnospiraceae	unclassified_f__Lachnospiraceae	OTU834	12	176	191	195
Firmicutes	Clostridia	Clostridiales	Clostridiales_vadinBB6	norank_f__Clostridiales_vadinBB60_	uncultured_bacterium_g__norank_f__Clostridiales_vadinBB60_group	OTU512	8	232	200	45
Firmicutes	Erysipelotrichia	Erysipelotrichales	Erysipelotrichaceae	Erysipelatoclostridium	Candidatus_Stoquefichus_sp._SN16	OTU707	0	86	4	2
Firmicutes	Clostridia	Clostridiales	Lachnospiraceae	[Eubacterium]_hallii_group	uncultured_bacterium_g__[Eubacterium]_hallii_group	OTU848	10	81	5	21
Firmicutes	Clostridia	Clostridiales	Ruminococcaceae	Ruminiclostridium_5	uncultured_bacterium_g__Ruminiclostridium_5	OTU856	34	90	60	65
Firmicutes	Clostridia	Clostridiales	Christensenellaceae	Christensenellaceae_R-7_group	uncultured_bacterium_g__Christensenellaceae_R-7_group	OTU171	3	34	61	29
Firmicutes	Clostridia	Clostridiales	Lachnospiraceae	Blautia	unclassified_g__Blautia	OTU767	0	13	20	26
Firmicutes	Clostridia	Clostridiales	Lachnospiraceae	unclassified_f__Lachnospiraceae	unclassified_f__Lachnospiraceae	OTU828	1	4	15	53
Firmicutes	Clostridia	Clostridiales	Ruminococcaceae	Ruminococcaceae_UCG-005	uncultured_Clostridiales_bacterium_g__Ruminococcaceae_UCG-005	OTU815	1	65	36	27
Firmicutes	Clostridia	Clostridiales	Ruminococcaceae	unclassified_f__Ruminococcaceae	unclassified_f__Ruminococcaceae	OTU845	144	19	25	59
Firmicutes	Clostridia	Clostridiales	Lachnospiraceae	unclassified_f__Lachnospiraceae	unclassified_f__Lachnospiraceae	OTU770	62	34	114	127
Firmicutes	Clostridia	Clostridiales	Ruminococcaceae	Anaerotruncus	uncultured_bacterium_g__Anaerotruncus	OTU838	0	21	10	69
Firmicutes	Clostridia	Clostridiales	Lachnospiraceae	unclassified_f__Lachnospiraceae	unclassified_f__Lachnospiraceae	OTU82	5	98	46	232
Firmicutes	Clostridia	Clostridiales	Ruminococcaceae	Anaerotruncus	Anaerotruncus_colihominis_DSM_17241	OTU125	0	69	5	22
Firmicutes	Clostridia	Clostridiales	Clostridiales_vadinBB6	norank_f__Clostridiales_vadinBB60_	uncultured_Clostridia_bacterium_g__norank_f__Clostridiales_vadinBB60_group	OTU697	18	2	538	220
Proteobacteria	Gammaproteobacteria	Pseudomonadales	Pseudomonadaceae	Pseudomonas	unclassified_g__Pseudomonas	OTU58	13	189	295	20
Firmicutes	Clostridia	Clostridiales	Christensenellaceae	Christensenellaceae_R-7_group	unclassified_g__Christensenellaceae_R-7_group	OTU830	1	0	44	65
Firmicutes	Clostridia	Clostridiales	Ruminococcaceae	Subdoligranulum	uncultured_Subdoligranulum_sp.	OTU519	37	1	107	17
Firmicutes	Clostridia	Clostridiales	Lachnospiraceae	Marvinbryantia	unclassified_g__Marvinbryantia	OTU177	2	32	8	10

Firmicutes	Clostridia	Clostridiales	Clostridiales_vadinBB6	norank_f__Clostridiales_vadinBB60_	unclassified_g__norank_f__Clostridiales_vadinBB60_group	OTU107	20	11	67	130
Firmicutes	Clostridia	Clostridiales	Lachnospiraceae	unclassified_f__Lachnospiraceae	unclassified_f__Lachnospiraceae	OTU216	72	9	76	33
Firmicutes	Clostridia	Clostridiales	Ruminococcaceae	Ruminococcaceae_NK4A214_group	uncultured_organism_g__Ruminococcaceae_NK4A214_group	OTU703	6	45	144	95
Firmicutes	Clostridia	Clostridiales	Lachnospiraceae	unclassified_f__Lachnospiraceae	unclassified_f__Lachnospiraceae	OTU540	24	50	9	197
Firmicutes	Clostridia	Clostridiales	Defluviitaleaceae	Defluviitaleaceae_UCG-011	uncultured_bacterium_g__Defluviitaleaceae_UCG-011	OTU725	19	119	20	247
Firmicutes	Clostridia	Clostridiales	Ruminococcaceae	Ruminococcaceae_UCG-010	uncultured_bacterium_g__Ruminococcaceae_UCG-010	OTU799	0	2	62	55
Firmicutes	Clostridia	Clostridiales	Ruminococcaceae	Ruminococcaceae_UCG-014	unclassified_g__Ruminococcaceae_UCG-014	OTU163	1	0	502	12
Firmicutes	Clostridia	Clostridiales	Clostridiales_vadinBB6	norank_f__Clostridiales_vadinBB60_	unclassified_g__norank_f__Clostridiales_vadinBB60_group	OTU57	58	99	463	80
Tenericutes	Mollicutes	Mollicutes_RF9	norank_o__Mollicutes	norank_o__Mollicutes_RF9	unclassified_g__norank_o__Mollicutes_RF9	OTU294	69	79	55	40
Firmicutes	Clostridia	Clostridiales	Ruminococcaceae	Hydrogenoanaerobacterium	uncultured_bacterium_g__Hydrogenoanaerobacterium	OTU613	5	75	52	64
Firmicutes	Clostridia	Clostridiales	Ruminococcaceae	Ruminococcaceae_UCG-014	unclassified_g__Ruminococcaceae_UCG-014	OTU405	8	504	17	7
Firmicutes	Clostridia	Clostridiales	Ruminococcaceae	Ruminococcaceae_UCG-014	uncultured_bacterium_g__Ruminococcaceae_UCG-014	OTU570	1	57	175	31
Firmicutes	Clostridia	Clostridiales	Ruminococcaceae	norank_f__Ruminococcaceae	unclassified_g__norank_f__Ruminococcaceae	OTU313	9	37	13	80
Firmicutes	Bacilli	Lactobacillales	Lactobacillaceae	Lactobacillus	Lactobacillus_aviarius	OTU599	0	0	0	0
Firmicutes	Clostridia	Clostridiales	Lachnospiraceae	unclassified_f__Lachnospiraceae	unclassified_f__Lachnospiraceae	OTU717	31	27	35	20
Firmicutes	Clostridia	Clostridiales	Lachnospiraceae	Eisenbergiella	unclassified_g__Eisenbergiella	OTU443	27	66	64	100
Firmicutes	Clostridia	Clostridiales	Ruminococcaceae	norank_f__Ruminococcaceae	Ruminococcus_sp._16442	OTU113	26	208	61	18
Firmicutes	Clostridia	Clostridiales	Ruminococcaceae	Ruminococcus_1	unclassified_g__Ruminococcus_1	OTU140	8	29	23	45
Firmicutes	Clostridia	Clostridiales	Lachnospiraceae	Lachnospira	unclassified_g__Lachnospira	OTU170	21	91	113	405
Tenericutes	Mollicutes	Mollicutes_RF9	norank_o__Mollicutes	norank_o__Mollicutes_RF9	uncultured_bacterium_g__norank_o__Mollicutes_RF9	OTU823	54	103	174	16
Firmicutes	Clostridia	Clostridiales	Ruminococcaceae	unclassified_f__Ruminococcaceae	unclassified_f__Ruminococcaceae	OTU835	32	251	16	44
Firmicutes	Erysipelotrichia	Erysipelotrichales	Erysipelotrichaceae	norank_f__Erysipelotrichaceae	Clostridiales_bacterium_10-3b	OTU821	58	20	120	23
Firmicutes	Clostridia	Clostridiales	Peptococcaceae	norank_f__Peptococcaceae	uncultured_bacterium_g__norank_f__Peptococcaceae	OTU451	3	153	60	38
Firmicutes	Clostridia	Clostridiales	Lachnospiraceae	unclassified_f__Lachnospiraceae	unclassified_f__Lachnospiraceae	OTU135	0	0	2	0
Firmicutes	Clostridia	Clostridiales	Lachnospiraceae	unclassified_f__Lachnospiraceae	unclassified_f__Lachnospiraceae	OTU550	0	0	89	34
Firmicutes	Clostridia	Clostridiales	Ruminococcaceae	Anaerotruncus	uncultured_bacterium_g__Anaerotruncus	OTU556	5	15	27	15
Firmicutes	Erysipelotrichia	Erysipelotrichales	Erysipelotrichaceae	norank_f__Erysipelotrichaceae	Clostridiales_bacterium_60-7e	OTU583	244	47	17	16
Firmicutes	Clostridia	Clostridiales	Ruminococcaceae	Ruminococcaceae_UCG-014	unclassified_g__Ruminococcaceae_UCG-014	OTU200	1	66	94	17
Bacteroidetes	Bacteroidia	Bacteroidales	Rikenellaceae	Alistipes	gut_metagenome_g__Alistipes	OTU197	10	46	20	127
Tenericutes	Mollicutes	Mollicutes_RF9	norank_o__Mollicutes	norank_o__Mollicutes_RF9	gut_metagenome_g__norank_o__Mollicutes_RF9	OTU156	0	0	163	0
Firmicutes	Clostridia	Clostridiales	Ruminococcaceae	unclassified_f__Ruminococcaceae	unclassified_f__Ruminococcaceae	OTU66	249	1	11	66
Firmicutes	Erysipelotrichia	Erysipelotrichales	Erysipelotrichaceae	Faecalitalea	unclassified_g__Faecalitalea	OTU784	1	9	166	1
Firmicutes	Bacilli	Lactobacillales	Lactobacillaceae	Lactobacillus	Lactobacillus_johnsonii	OTU601	2	108	404	285
Bacteroidetes	Bacteroidia	Bacteroidales	Rikenellaceae	Alistipes	unclassified_g__Alistipes	OTU577	10	45	16	141
Firmicutes	Clostridia	Clostridiales	Ruminococcaceae	Subdoligranulum	unclassified_g__Subdoligranulum	OTU83	235	2	6	71
Proteobacteria	Deltaproteobacteria	Desulfovibrionales	Desulfovibrionaceae	Desulfovibrio	unclassified_g__Desulfovibrio	OTU636	1	79	95	18
Firmicutes	Clostridia	Clostridiales	Clostridiales_vadinBB6	norank_f__Clostridiales_vadinBB60_	unclassified_g__norank_f__Clostridiales_vadinBB60_group	OTU102	1	239	181	35
Firmicutes	Erysipelotrichia	Erysipelotrichales	Erysipelotrichaceae	Erysipelatoclostridium	unclassified_g__Erysipelatoclostridium	OTU8	73	159	49	24
Firmicutes	Clostridia	Clostridiales	Lachnospiraceae	[Ruminococcus]_torques_group	unclassified_g__[Ruminococcus]_torques_group	OTU531	177	2	2	18
Firmicutes	Clostridia	Clostridiales	Ruminococcaceae	Ruminococcaceae_UCG-014	unclassified_g__Ruminococcaceae_UCG-014	OTU614	0	0	10	0
Actinobacteria	Actinobacteria	Coriobacteriales	Coriobacteriaceae	Senegalimassilia	Coriobacteriaceae_bacterium_CHKCI002	OTU206	42	267	29	28
Firmicutes	Clostridia	Clostridiales	Ruminococcaceae	Ruminococcaceae_UCG-014	uncultured_bacterium_g__Ruminococcaceae_UCG-014	OTU842	0	62	168	4
Firmicutes	Clostridia	Clostridiales	Ruminococcaceae	Anaerotruncus	uncultured_organism_g__Anaerotruncus	OTU568	0	9	192	65
Firmicutes	Clostridia	Clostridiales	Ruminococcaceae	norank_f__Ruminococcaceae	unclassified_g__norank_f__Ruminococcaceae	OTU141	0	317	7	43
Firmicutes	Clostridia	Clostridiales	Clostridiales_vadinBB6	norank_f__Clostridiales_vadinBB60_	uncultured_bacterium_g__norank_f__Clostridiales_vadinBB60_group	OTU109	0	0	114	0
Cyanobacteria	Cyanobacteria	Gastranaerophilales	norank_o__Gastranae	norank_o__Gastranaerophilales	uncultured_rumen_bacterium_g__norank_o__Gastranaerophilales	OTU142	0	0	0	16
Firmicutes	Clostridia	Clostridiales	Lachnospiraceae	unclassified_f__Lachnospiraceae	unclassified_f__Lachnospiraceae	OTU699	1	87	101	55
Firmicutes	Clostridia	Clostridiales	Ruminococcaceae	Ruminococcaceae_UCG-010	gut_metagenome_g__Ruminococcaceae_UCG-010	OTU827	14	49	31	52
Firmicutes	Clostridia	Clostridiales	Lachnospiraceae	unclassified_f__Lachnospiraceae	unclassified_f__Lachnospiraceae	OTU612	20	19	52	154
Firmicutes	Clostridia	Clostridiales	Defluviitaleaceae	Defluviitaleaceae_UCG-011	uncultured_bacterium_g__Defluviitaleaceae_UCG-011	OTU567	2	39	48	43
Firmicutes	Clostridia	Clostridiales	Lachnospiraceae	Blautia	uncultured_bacterium_g__Blautia	OTU766	10	6	1	0
Firmicutes	Clostridia	Clostridiales	Christensenellaceae	Christensenellaceae_R-7_group	unclassified_g__Christensenellaceae_R-7_group	OTU148	2	88	151	13
Tenericutes	Mollicutes	Mollicutes_RF9	norank_o__Mollicutes	norank_o__Mollicutes_RF9	uncultured_bacterium_g__norank_o__Mollicutes_RF9	OTU559	16	46	159	48
Firmicutes	Clostridia	Clostridiales	Ruminococcaceae	Anaerotruncus	unclassified_g__Anaerotruncus	OTU657	51	104	2	19
Firmicutes	Clostridia	Clostridiales	Clostridiales_vadinBB6	norank_f__Clostridiales_vadinBB60_	uncultured_bacterium_g__norank_f__Clostridiales_vadinBB60_group	OTU22	0	65	73	0
Firmicutes	Clostridia	Clostridiales	Ruminococcaceae	Anaerotruncus	uncultured_Clostridiaceae_bacterium_g__Anaerotruncus	OTU618	29	48	18	614
Firmicutes	Clostridia	Clostridiales	Ruminococcaceae	Flavonifractor	uncultured_bacterium_g__Flavonifractor	OTU511	4	7	101	49
Firmicutes	Clostridia	Clostridiales	Clostridiales_vadinBB6	norank_f__Clostridiales_vadinBB60_	uncultured_bacterium_g__norank_f__Clostridiales_vadinBB60_group	OTU761	0	54	0	0
Firmicutes	Clostridia	Clostridiales	Clostridiales_vadinBB6	norank_f__Clostridiales_vadinBB60_	unclassified_g__norank_f__Clostridiales_vadinBB60_group	OTU231	13	103	65	0
Firmicutes	Clostridia	Clostridiales	Lachnospiraceae	unclassified_f__Lachnospiraceae	unclassified_f__Lachnospiraceae	OTU73	5	42	51	22
Firmicutes	Erysipelotrichia	Erysipelotrichales	Erysipelotrichaceae	Erysipelatoclostridium	Clostridiales_bacterium_CHKCI006	OTU700	52	19	32	30
Firmicutes	Clostridia	Clostridiales	Lachnospiraceae	unclassified_f__Lachnospiraceae	unclassified_f__Lachnospiraceae	OTU742	0	0	6	29
Firmicutes	Clostridia	Clostridiales	Ruminococcaceae	Anaerotruncus	uncultured_bacterium_g__Anaerotruncus	OTU146	0	38	37	62
Firmicutes	Clostridia	Clostridiales	Ruminococcaceae	Ruminococcaceae_UCG-010	uncultured_bacterium_g__Ruminococcaceae_UCG-010	OTU741	0	61	28	41
Firmicutes	Clostridia	Clostridiales	Ruminococcaceae	Subdoligranulum	unclassified_g__Subdoligranulum	OTU62	0	0	9	14
Firmicutes	Clostridia	Clostridiales	Lachnospiraceae	Blautia	uncultured_bacterium_g__Blautia	OTU466	10	61	28	75
Firmicutes	Clostridia	Clostridiales	Christensenellaceae	Christensenellaceae_R-7_group	uncultured_bacterium_g__Christensenellaceae_R-7_group	OTU746	7	52	102	34
Tenericutes	Mollicutes	Mollicutes_RF9	norank_o__Mollicutes	norank_o__Mollicutes_RF9	uncultured_bacterium_g__norank_o__Mollicutes_RF9	OTU192	2	1	68	35
Firmicutes	Clostridia	Clostridiales	Clostridiales_vadinBB6	norank_f__Clostridiales_vadinBB60_	uncultured_organism_g__norank_f__Clostridiales_vadinBB60_group	OTU103	0	0	11	12
Firmicutes	Clostridia	Clostridiales	Lachnospiraceae	unclassified_f__Lachnospiraceae	unclassified_f__Lachnospiraceae	OTU473	11	17	0	15
Firmicutes	Clostridia	Clostridiales	Ruminococcaceae	Ruminiclostridium_9	uncultured_bacterium_g__Ruminiclostridium_9	OTU110	2	0	203	12
Firmicutes	Bacilli	Lactobacillales	Lactobacillaceae	Lactobacillus	Lactobacillus_agilis	OTU607	1	0	0	0
Firmicutes	Clostridia	Clostridiales	Lachnospiraceae	Marvinbryantia	uncultured_organism_g__Marvinbryantia	OTU437	0	101	24	71
Firmicutes	Clostridia	Clostridiales	Ruminococcaceae	Ruminococcaceae_UCG-014	unclassified_g__Ruminococcaceae_UCG-014	OTU338	0	1	10	37
Firmicutes	Clostridia	Clostridiales	Clostridiales_vadinBB6	norank_f__Clostridiales_vadinBB60_	uncultured_rumen_bacterium_g__norank_f__Clostridiales_vadinBB60_group	OTU330	0	0	350	0
Tenericutes	Mollicutes	Mollicutes_RF9	norank_o__Mollicutes	norank_o__Mollicutes_RF9	uncultured_bacterium_g__norank_o__Mollicutes_RF9	OTU28	0	372	60	2
Firmicutes	Clostridia	Clostridiales	Lachnospiraceae	Coprococcus_1	unclassified_g__Coprococcus_1	OTU539	0	0	269	4
Firmicutes	Clostridia	Clostridiales	Lachnospiraceae	unclassified_f__Lachnospiraceae	unclassified_f__Lachnospiraceae	OTU642	4	23	39	13
Firmicutes	Clostridia	Clostridiales	Ruminococcaceae	Ruminococcaceae_UCG-014	unclassified_g__Ruminococcaceae_UCG-014	OTU590	0	5	4	26
Firmicutes	Clostridia	Clostridiales	Ruminococcaceae	norank_f__Ruminococcaceae	unclassified_g__norank_f__Ruminococcaceae	OTU701	3	2	11	3
Firmicutes	Clostridia	Clostridiales	Ruminococcaceae	Anaerotruncus	uncultured_bacterium_g__Anaerotruncus	OTU249	0	22	1	17
Firmicutes	Clostridia	Clostridiales	Ruminococcaceae	Anaerotruncus	uncultured_bacterium_g__Anaerotruncus	OTU248	0	35	14	33
Firmicutes	Clostridia	Clostridiales	Ruminococcaceae	Ruminiclostridium_5	unclassified_g__Ruminiclostridium_5	OTU826	40	6	2	0
Firmicutes	Clostridia	Clostridiales	Family_XIII	[Eubacterium]_nodatum_group	Eubacteriaceae_bacterium_Marseille-P2843	OTU585	23	11	9	35
Firmicutes	Clostridia	Clostridiales	Christensenellaceae	Christensenellaceae_R-7_group	uncultured_bacterium_g__Christensenellaceae_R-7_group	OTU167	0	48	91	23
Firmicutes	Clostridia	Clostridiales	Lachnospiraceae	norank_f__Lachnospiraceae	Clostridiales_bacterium_CHKCI001	OTU520	2	50	21	23
Firmicutes	Clostridia	Clostridiales	Ruminococcaceae	unclassified_f__Ruminococcaceae	unclassified_f__Ruminococcaceae	OTU238	5	37	84	26
Firmicutes	Clostridia	Clostridiales	Ruminococcaceae	unclassified_f__Ruminococcaceae	unclassified_f__Ruminococcaceae	OTU133	40	25	13	88
Tenericutes	Mollicutes	Mollicutes_RF9	norank_o__Mollicutes	norank_o__Mollicutes_RF9	uncultured_bacterium_g__norank_o__Mollicutes_RF9	OTU606	11	9	90	13
Firmicutes	Clostridia	Clostridiales	Ruminococcaceae	Ruminococcaceae_UCG-009	uncultured_bacterium_g__Ruminococcaceae_UCG-009	OTU779	0	52	41	29
Firmicutes	Clostridia	Clostridiales	Ruminococcaceae	Ruminococcaceae_NK4A214_group	uncultured_organism_g__Ruminococcaceae_NK4A214_group	OTU410	0	51	39	14
Verrucomicrobia	Verrucomicrobiae	Verrucomicrobiales	Verrucomicrobiaceae	Akkermansia	unclassified_g__Akkermansia	OTU344	0	0	0	0
Firmicutes	Clostridia	Clostridiales	Clostridiales_vadinBB6	norank_f__Clostridiales_vadinBB60_	uncultured_bacterium_g__norank_f__Clostridiales_vadinBB60_group	OTU1	2	0	25	47
Firmicutes	Clostridia	Clostridiales	Ruminococcaceae	Oscillospira	uncultured_bacterium_g__Oscillospira	OTU158	0	1	110	22
Firmicutes	Clostridia	Clostridiales	Lachnospiraceae	Lachnospiraceae_NK4A136_group	unclassified_g__Lachnospiraceae_NK4A136_group	OTU187	0	0	103	11
Firmicutes	Clostridia	Clostridiales	Lachnospiraceae	unclassified_f__Lachnospiraceae	unclassified_f__Lachnospiraceae	OTU463	0	62	156	13
Firmicutes	Clostridia	Clostridiales	Peptostreptococcacea	unclassified_f__Peptostreptococca	unclassified_f__Peptostreptococcaceae	OTU526	8	47	3	6
Firmicutes	Bacilli	Lactobacillales	Lactobacillaceae	Lactobacillus	unclassified_g__Lactobacillus	OTU448	0	0	1	10
Firmicutes	Clostridia	Clostridiales	Ruminococcaceae	Ruminococcaceae_NK4A214_group	uncultured_rumen_bacterium_g__Ruminococcaceae_NK4A214_group	OTU181	0	0	101	36
Firmicutes	Clostridia	Clostridiales	Ruminococcaceae	Ruminococcaceae_UCG-014	unclassified_g__Ruminococcaceae_UCG-014	OTU822	7	6	7	56
Firmicutes	Clostridia	Clostridiales	Ruminococcaceae	Ruminococcaceae_UCG-013	unclassified_g__Ruminococcaceae_UCG-013	OTU458	0	0	0	1
Firmicutes	Clostridia	Clostridiales	Clostridiales_vadinBB6	norank_f__Clostridiales_vadinBB60_	unclassified_g__norank_f__Clostridiales_vadinBB60_group	OTU198	4	0	5	27
Firmicutes	Clostridia	Clostridiales	Clostridiales_vadinBB6	norank_f__Clostridiales_vadinBB60_	unclassified_g__norank_f__Clostridiales_vadinBB60_group	OTU241	0	0	0	0
Tenericutes	Mollicutes	Mollicutes_RF9	norank_o__Mollicutes	norank_o__Mollicutes_RF9	unclassified_g__norank_o__Mollicutes_RF9	OTU803	0	0	78	1
Firmicutes	Clostridia	Clostridiales	Lachnospiraceae	unclassified_f__Lachnospiraceae	unclassified_f__Lachnospiraceae	OTU81	3	3	26	0
Firmicutes	Clostridia	Clostridiales	Ruminococcaceae	Ruminococcaceae_UCG-014	unclassified_g__Ruminococcaceae_UCG-014	OTU12	0	0	84	0
Firmicutes	Clostridia	Clostridiales	Christensenellaceae	Christensenellaceae_R-7_group	unclassified_g__Christensenellaceae_R-7_group	OTU165	0	0	0	3
Firmicutes	Clostridia	Clostridiales	unclassified_o__Clostr	unclassified_o__Clostridiales	unclassified_o__Clostridiales	OTU682	0	379	1	1
Firmicutes	Clostridia	Clostridiales	Ruminococcaceae	unclassified_f__Ruminococcaceae	unclassified_f__Ruminococcaceae	OTU679	0	0	0	19
Firmicutes	Clostridia	Clostridiales	Ruminococcaceae	unclassified_f__Ruminococcaceae	unclassified_f__Ruminococcaceae	OTU455	105	4	2	43
Firmicutes	Clostridia	Clostridiales	Family_XIII	unclassified_f__Family_XIII	unclassified_f__Family_XIII	OTU488	10	25	11	28

Firmicutes	Clostridia	Clostridiales	Ruminococcaceae	Ruminococcaceae_UCG-013	uncultured_bacterium_g__Ruminococcaceae_UCG-013	OTU498	5	58	48	40
Firmicutes	Clostridia	Clostridiales	Ruminococcaceae	Anaerotruncus	unclassified_g__Anaerotruncus	OTU566	0	0	84	16
Firmicutes	Clostridia	Clostridiales	Clostridiales_vadinBB6	norank_f__Clostridiales_vadinBB60_	uncultured_Firmicutes_bacterium_g__norank_f__Clostridiales_vadinBB60_group	OTU638	0	6	29	34
Firmicutes	Clostridia	Clostridiales	Clostridiales_vadinBB6	norank_f__Clostridiales_vadinBB60_	uncultured_bacterium_g__norank_f__Clostridiales_vadinBB60_group	OTU3	0	3	715	8
Firmicutes	Clostridia	Clostridiales	Clostridiales_vadinBB6	norank_f__Clostridiales_vadinBB60_	uncultured_bacterium_g__norank_f__Clostridiales_vadinBB60_group	OTU494	5	38	21	12
Firmicutes	Clostridia	Clostridiales	Ruminococcaceae	Ruminiclostridium_5	uncultured_bacterium_g__Ruminiclostridium_5	OTU849	13	21	12	27
Firmicutes	Clostridia	Clostridiales	Ruminococcaceae	Ruminococcaceae_UCG-014	uncultured_bacterium_g__Ruminococcaceae_UCG-014	OTU114	0	0	0	5
Firmicutes	Clostridia	Clostridiales	Ruminococcaceae	Ruminococcaceae_UCG-010	uncultured_bacterium_g__Ruminococcaceae_UCG-010	OTU326	0	51	7	7
Firmicutes	Clostridia	Clostridiales	Ruminococcaceae	Ruminococcaceae_UCG-014	uncultured_bacterium_g__Ruminococcaceae_UCG-014	OTU749	2	60	22	40
Firmicutes	Clostridia	Clostridiales	Ruminococcaceae	[Eubacterium]_coprostanoligenes_g	unclassified_g__[Eubacterium]_coprostanoligenes_group	OTU233	0	116	231	0
Firmicutes	Clostridia	Clostridiales	Ruminococcaceae	Ruminococcaceae_UCG-014	unidentified_rumen_bacterium_JW32	OTU36	1	78	169	42
Firmicutes	Clostridia	Clostridiales	Ruminococcaceae	Ruminococcaceae_UCG-008	uncultured_bacterium_g__Ruminococcaceae_UCG-008	OTU39	0	0	0	0
Firmicutes	Clostridia	Clostridiales	Family_XIII	Family_XIII_UCG-001	uncultured_bacterium_g__Family_XIII_UCG-001	OTU329	4	5	13	25
Firmicutes	Clostridia	Clostridiales	Ruminococcaceae	Ruminiclostridium_5	uncultured_bacterium_g__Ruminiclostridium_5	OTU75	11	32	20	29
Firmicutes	Clostridia	Clostridiales	Peptococcaceae	norank_f__Peptococcaceae	uncultured_organism_g__norank_f__Peptococcaceae	OTU343	0	0	0	0
Firmicutes	Clostridia	Clostridiales	Ruminococcaceae	Anaerofilum	uncultured_bacterium_g__Anaerofilum	OTU660	0	5	45	14
Firmicutes	Clostridia	Clostridiales	Lachnospiraceae	Marvinbryantia	uncultured_bacterium_g__Marvinbryantia	OTU536	15	7	7	19
Firmicutes	Clostridia	Clostridiales	Ruminococcaceae	Anaerotruncus	unclassified_g__Anaerotruncus	OTU10	0	9	34	34
Firmicutes	Clostridia	Clostridiales	Clostridiales_vadinBB6	norank_f__Clostridiales_vadinBB60_	uncultured_bacterium_g__norank_f__Clostridiales_vadinBB60_group	OTU678	0	0	103	0
Firmicutes	Clostridia	Clostridiales	Ruminococcaceae	Ruminiclostridium_5	unclassified_g__Ruminiclostridium_5	OTU84	5	28	17	16
Firmicutes	Clostridia	Clostridiales	unclassified_o__Clostr	unclassified_o__Clostridiales	unclassified_o__Clostridiales	OTU137	80	3	7	27
Tenericutes	Mollicutes	Mollicutes_RF9	norank_o__Mollicutes	norank_o__Mollicutes_RF9	uncultured_bacterium_g__norank_o__Mollicutes_RF9	OTU604	0	1	44	17
Firmicutes	Erysipelotrichia	Erysipelotrichales	Erysipelotrichaceae	norank_f__Erysipelotrichaceae	uncultured_Erysipelotrichaceae_bacterium_g__norank	OTU772	0	0	0	24
Tenericutes	Mollicutes	Anaeroplasmatales	Anaeroplasmataceae	Anaeroplasma	uncultured_bacterium_g__Anaeroplasma	OTU801	0	71	17	3
Firmicutes	Clostridia	Clostridiales	Ruminococcaceae	Ruminococcaceae_UCG-014	unclassified_g__Ruminococcaceae_UCG-014	OTU220	0	0	0	0
Firmicutes	Clostridia	Clostridiales	Ruminococcaceae	Ruminiclostridium_9	unclassified_g__Ruminiclostridium_9	OTU587	3	13	17	15
Firmicutes	Clostridia	Clostridiales	Ruminococcaceae	norank_f__Ruminococcaceae	human_gut_metagenome_g__norank_f__Ruminococcaceae	OTU85	0	16	32	9
Cyanobacteria	Cyanobacteria	Gastranaerophilales	norank_o__Gastranae	norank_o__Gastranaerophilales	uncultured_bacterium_g__norank_o__Gastranaerophilales	OTU502	2	0	10	0
Firmicutes	Clostridia	Clostridiales	Clostridiales_vadinBB6	norank_f__Clostridiales_vadinBB60_	uncultured_bacterium_g__norank_f__Clostridiales_vadinBB60_group	OTU17	0	56	37	11
Firmicutes	Clostridia	Clostridiales	Lachnospiraceae	unclassified_f__Lachnospiraceae	unclassified_f__Lachnospiraceae	OTU513	7	15	27	13
Firmicutes	Clostridia	Clostridiales	Ruminococcaceae	unclassified_f__Ruminococcaceae	unclassified_f__Ruminococcaceae	OTU166	2	12	7	10
Tenericutes	Mollicutes	NB1-n	norank_o__NB1-n	norank_o__NB1-n	uncultured_organism_g__norank_o__NB1-n	OTU603	0	0	34	0
Firmicutes	Clostridia	Clostridiales	Ruminococcaceae	Ruminococcaceae_UCG-013	uncultured_bacterium_g__Ruminococcaceae_UCG-013	OTU720	0	15	11	31
Firmicutes	Clostridia	Clostridiales	Ruminococcaceae	Ruminococcaceae_UCG-014	unclassified_g__Ruminococcaceae_UCG-014	OTU347	0	6	13	10
Firmicutes	Clostridia	Clostridiales	Ruminococcaceae	Ruminiclostridium	uncultured_bacterium_g__Ruminiclostridium	OTU576	0	0	1	0
Cyanobacteria	Cyanobacteria	Gastranaerophilales	norank_o__Gastranae	norank_o__Gastranaerophilales	unclassified_g__norank_o__Gastranaerophilales	OTU563	0	0	0	1
Firmicutes	Clostridia	Clostridiales	Lachnospiraceae	unclassified_f__Lachnospiraceae	unclassified_f__Lachnospiraceae	OTU731	1	59	14	51
Firmicutes	Clostridia	Clostridiales	Lachnospiraceae	Roseburia	unclassified_g__Roseburia	OTU449	1	2	41	6
Firmicutes	Clostridia	Clostridiales	Clostridiales_vadinBB6	norank_f__Clostridiales_vadinBB60_	uncultured_bacterium_g__norank_f__Clostridiales_vadinBB60_group	OTU669	0	0	2	4
Tenericutes	Mollicutes	Mollicutes_RF9	norank_o__Mollicutes	norank_o__Mollicutes_RF9	unclassified_g__norank_o__Mollicutes_RF9	OTU104	0	0	8	23
Firmicutes	Clostridia	Clostridiales	Lachnospiraceae	Lachnospiraceae_UCG-010	uncultured_bacterium_g__Lachnospiraceae_UCG-010	OTU663	7	23	2	5
Firmicutes	Clostridia	Clostridiales	Ruminococcaceae	unclassified_f__Ruminococcaceae	unclassified_f__Ruminococcaceae	OTU847	0	1	63	43
Firmicutes	Clostridia	Clostridiales	Ruminococcaceae	Ruminococcaceae_UCG-013	uncultured_bacterium_g__Ruminococcaceae_UCG-013	OTU721	6	21	42	41
Tenericutes	Mollicutes	Mollicutes_RF9	norank_o__Mollicutes	norank_o__Mollicutes_RF9	uncultured_bacterium_g__norank_o__Mollicutes_RF9	OTU627	0	0	25	0
Firmicutes	Clostridia	Clostridiales	Ruminococcaceae	[Eubacterium]_coprostanoligenes_g	unclassified_g__[Eubacterium]_coprostanoligenes_group	OTU121	0	2	1	2
Firmicutes	Clostridia	Clostridiales	Clostridiales_vadinBB6	norank_f__Clostridiales_vadinBB60_	unclassified_g__norank_f__Clostridiales_vadinBB60_group	OTU180	22	67	4	12
Firmicutes	Clostridia	Clostridiales	Christensenellaceae	Christensenellaceae_R-7_group	unclassified_g__Christensenellaceae_R-7_group	OTU54	0	0	8	0
Firmicutes	Clostridia	Clostridiales	Ruminococcaceae	norank_f__Ruminococcaceae	unclassified_g__norank_f__Ruminococcaceae	OTU534	10	0	17	2
Firmicutes	Clostridia	Clostridiales	Ruminococcaceae	unclassified_f__Ruminococcaceae	unclassified_f__Ruminococcaceae	OTU493	11	10	20	17
Firmicutes	Clostridia	Clostridiales	Lachnospiraceae	norank_f__Lachnospiraceae	unclassified_g__norank_f__Lachnospiraceae	OTU560	70	1	13	7
Firmicutes	Clostridia	Clostridiales	Lachnospiraceae	[Eubacterium]_ventriosum_group	uncultured_organism_g__[Eubacterium]_ventriosum_group	OTU464	1	9	2	32
Tenericutes	Mollicutes	Mollicutes_RF9	norank_o__Mollicutes	norank_o__Mollicutes_RF9	unclassified_g__norank_o__Mollicutes_RF9	OTU234	0	0	255	11
Firmicutes	Clostridia	Clostridiales	Lachnospiraceae	unclassified_f__Lachnospiraceae	unclassified_f__Lachnospiraceae	OTU64	17	98	14	6
Tenericutes	Mollicutes	Mollicutes_RF9	norank_o__Mollicutes	norank_o__Mollicutes_RF9	uncultured_bacterium_g__norank_o__Mollicutes_RF9	OTU602	0	0	55	0
Firmicutes	Clostridia	Clostridiales	Ruminococcaceae	Anaerotruncus	uncultured_bacterium_g__Anaerotruncus	OTU6	26	0	10	1
Firmicutes	Bacilli	Lactobacillales	Lactobacillaceae	Lactobacillus	Lactobacillus_murinus_g__Lactobacillus	OTU843	0	0	0	0
Firmicutes	Clostridia	Clostridiales	Christensenellaceae	Christensenellaceae_R-7_group	uncultured_prokaryote_g__Christensenellaceae_R-7_group	OTU67	0	0	11	49
Firmicutes	Clostridia	Clostridiales	Clostridiales_vadinBB6	norank_f__Clostridiales_vadinBB60_	uncultured_bacterium_g__norank_f__Clostridiales_vadinBB60_group	OTU748	0	0	2	0
Firmicutes	Clostridia	Clostridiales	Lachnospiraceae	Coprococcus_1	unclassified_g__Coprococcus_1	OTU739	4	18	9	5
Tenericutes	Mollicutes	Mollicutes_RF9	norank_o__Mollicutes	norank_o__Mollicutes_RF9	unclassified_g__norank_o__Mollicutes_RF9	OTU229	0	19	39	9
Firmicutes	Clostridia	Clostridiales	Clostridiales_vadinBB6	norank_f__Clostridiales_vadinBB60_	uncultured_bacterium_g__norank_f__Clostridiales_vadinBB60_group	OTU23	0	7	14	0
Firmicutes	Clostridia	Clostridiales	Clostridiales_vadinBB6	norank_f__Clostridiales_vadinBB60_	uncultured_bacterium_g__norank_f__Clostridiales_vadinBB60_group	OTU131	0	0	0	0
Firmicutes	Clostridia	Clostridiales	Ruminococcaceae	Ruminococcaceae_UCG-014	unclassified_g__Ruminococcaceae_UCG-014	OTU837	1	0	138	8
Firmicutes	Clostridia	Clostridiales	unclassified_o__Clostr	unclassified_o__Clostridiales	unclassified_o__Clostridiales	OTU91	0	44	16	19
Firmicutes	Clostridia	Clostridiales	Lachnospiraceae	Roseburia	unclassified_g__Roseburia	OTU43	51	2	10	1
Firmicutes	Clostridia	Clostridiales	Lachnospiraceae	unclassified_f__Lachnospiraceae	unclassified_f__Lachnospiraceae	OTU11	62	1	5	10
Proteobacteria	Betaproteobacteria	Burkholderiales	Oxalobacteraceae	Oxalobacter	unclassified_g__Oxalobacter	OTU557	0	0	0	0
Firmicutes	Clostridia	Clostridiales	Ruminococcaceae	Faecalibacterium	unclassified_g__Faecalibacterium	OTU219	154	0	0	7
Firmicutes	Clostridia	Clostridiales	Ruminococcaceae	Ruminiclostridium_5	uncultured_bacterium_g__Ruminiclostridium_5	OTU59	1	4	24	3
Firmicutes	Clostridia	Clostridiales	Lachnospiraceae	Lachnospiraceae_UCG-010	uncultured_bacterium_g__Lachnospiraceae_UCG-010	OTU755	0	6	6	22
Bacteroidetes	Bacteroidia	Bacteroidales	Rikenellaceae	Alistipes	uncultured_bacterium_g__Alistipes	OTU765	0	0	0	0
Firmicutes	Erysipelotrichia	Erysipelotrichales	Erysipelotrichaceae	[Clostridium]_innocuum_group	uncultured_bacterium_g__[Clostridium]_innocuum_group	OTU794	5	11	0	0
Firmicutes	Clostridia	Clostridiales	unclassified_o__Clostr	unclassified_o__Clostridiales	unclassified_o__Clostridiales	OTU673	0	0	99	0
Firmicutes	Clostridia	Clostridiales	Clostridiales_vadinBB6	norank_f__Clostridiales_vadinBB60_	gut_metagenome_g__norank_f__Clostridiales_vadinBB60_group	OTU548	35	68	33	23
Tenericutes	Mollicutes	Mollicutes_RF9	norank_o__Mollicutes	norank_o__Mollicutes_RF9	uncultured_bacterium_g__norank_o__Mollicutes_RF9	OTU544	0	0	57	3
Cyanobacteria	Cyanobacteria	Gastranaerophilales	norank_o__Gastranae	norank_o__Gastranaerophilales	unclassified_g__norank_o__Gastranaerophilales	OTU155	0	0	44	1
Firmicutes	Clostridia	Clostridiales	Clostridiales_vadinBB6	norank_f__Clostridiales_vadinBB60_	uncultured_bacterium_g__norank_f__Clostridiales_vadinBB60_group	OTU481	0	0	7	0
Firmicutes	Clostridia	Clostridiales	Lachnospiraceae	unclassified_f__Lachnospiraceae	unclassified_f__Lachnospiraceae	OTU467	0	10	3	2
Firmicutes	Clostridia	Clostridiales	Ruminococcaceae	norank_f__Ruminococcaceae	unclassified_g__norank_f__Ruminococcaceae	OTU622	25	0	0	2
Firmicutes	Clostridia	Clostridiales	Lachnospiraceae	unclassified_f__Lachnospiraceae	unclassified_f__Lachnospiraceae	OTU201	32	3	2	35
Firmicutes	Clostridia	Clostridiales	Lachnospiraceae	norank_f__Lachnospiraceae	uncultured_organism_g__norank_f__Lachnospiraceae	OTU654	0	2	1	3
Firmicutes	Clostridia	Clostridiales	Ruminococcaceae	Ruminococcaceae_UCG-014	unclassified_g__Ruminococcaceae_UCG-014	OTU575	0	0	2	0
Tenericutes	Mollicutes	Mollicutes_RF9	norank_o__Mollicutes	norank_o__Mollicutes_RF9	unclassified_g__norank_o__Mollicutes_RF9	OTU600	1	0	38	8
Firmicutes	Clostridia	Clostridiales	Ruminococcaceae	unclassified_f__Ruminococcaceae	unclassified_f__Ruminococcaceae	OTU728	18	3	1	40
Firmicutes	Clostridia	Clostridiales	Ruminococcaceae	Anaerotruncus	unclassified_g__Anaerotruncus	OTU159	1	0	23	7
Tenericutes	Mollicutes	Mollicutes_RF9	norank_o__Mollicutes	norank_o__Mollicutes_RF9	uncultured_bacterium_g__norank_o__Mollicutes_RF9	OTU608	1	0	0	0
Firmicutes	Clostridia	Clostridiales	Ruminococcaceae	Ruminococcaceae_UCG-010	uncultured_bacterium_g__Ruminococcaceae_UCG-010	OTU674	0	2	8	12
Firmicutes	Clostridia	Clostridiales	Lachnospiraceae	Lachnospiraceae_FCS020_group	uncultured_bacterium_g__Lachnospiraceae_FCS020_group	OTU802	2	4	26	18
Firmicutes	Clostridia	Clostridiales	Ruminococcaceae	Caproiciproducens	uncultured_bacterium_g__Caproiciproducens	OTU68	0	19	13	12
Firmicutes	Clostridia	Clostridiales	Peptococcaceae	norank_f__Peptococcaceae	unclassified_g__norank_f__Peptococcaceae	OTU586	0	5	8	5
Firmicutes	Clostridia	Clostridiales	Ruminococcaceae	Anaerotruncus	uncultured_bacterium_g__Anaerotruncus	OTU175	0	10	9	15
Firmicutes	Clostridia	Clostridiales	Lachnospiraceae	unclassified_f__Lachnospiraceae	unclassified_f__Lachnospiraceae	OTU854	0	0	0	16
Firmicutes	Clostridia	Clostridiales	Christensenellaceae	Christensenellaceae_R-7_group	uncultured_bacterium_g__Christensenellaceae_R-7_group	OTU164	0	5	11	4
Firmicutes	Clostridia	Clostridiales	Ruminococcaceae	Ruminococcus_2	uncultured_bacterium_g__Ruminococcus_2	OTU447	0	0	51	0
Actinobacteria	Actinobacteria	Coriobacteriales	Coriobacteriaceae	unclassified_f__Coriobacteriaceae	unclassified_f__Coriobacteriaceae	OTU490	17	55	1	2
Proteobacteria	Alphaproteobacteria	Rhizobiales	Brucellaceae	Brucella	Brucella_melitensis_g__Brucella	OTU812	0	0	0	0
Firmicutes	Clostridia	Clostridiales	Ruminococcaceae	Ruminiclostridium_1	uncultured_bacterium_g__Ruminiclostridium_1	OTU485	0	2	10	1
Tenericutes	Mollicutes	Mollicutes_RF9	norank_o__Mollicutes	norank_o__Mollicutes_RF9	unclassified_g__norank_o__Mollicutes_RF9	OTU611	0	0	66	1
Firmicutes	Clostridia	Clostridiales	Ruminococcaceae	Ruminococcaceae_UCG-014	unclassified_g__Ruminococcaceae_UCG-014	OTU100	0	0	1	0
Firmicutes	Clostridia	Clostridiales	Ruminococcaceae	norank_f__Ruminococcaceae	unclassified_g__norank_f__Ruminococcaceae	OTU98	0	0	0	0
Firmicutes	Bacilli	Lactobacillales	Enterococcaceae	Enterococcus	Enterococcus_faecalis_g__Enterococcus	OTU763	1	0	2	0
Firmicutes	Bacilli	Lactobacillales	Lactobacillaceae	Lactobacillus	Lactobacillus_coleohominis	OTU628	0	1	5	1
Firmicutes	Erysipelotrichia	Erysipelotrichales	Erysipelotrichaceae	unclassified_f__Erysipelotrichaceae	unclassified_f__Erysipelotrichaceae	OTU839	0	46	5	1
Firmicutes	Clostridia	Clostridiales	Christensenellaceae	Christensenellaceae_R-7_group	bacterium_YE57	OTU754	1	1	4	12
Firmicutes	Clostridia	Clostridiales	Ruminococcaceae	Ruminiclostridium_9	unclassified_g__Ruminiclostridium_9	OTU500	0	0	6	0
Firmicutes	Clostridia	Clostridiales	Ruminococcaceae	Faecalibacterium	unclassified_g__Faecalibacterium	OTU846	21	0	3	22
Tenericutes	Mollicutes	Mollicutes_RF9	norank_o__Mollicutes	norank_o__Mollicutes_RF9	unclassified_g__norank_o__Mollicutes_RF9	OTU610	0	0	0	0
Firmicutes	Clostridia	Clostridiales	Ruminococcaceae	norank_f__Ruminococcaceae	unclassified_g__norank_f__Ruminococcaceae	OTU132	60	3	0	4
Firmicutes	Clostridia	Clostridiales	Ruminococcaceae	Ruminococcus_1	uncultured_bacterium_g__Ruminococcus_1	OTU639	0	0	0	0
Firmicutes	Clostridia	Clostridiales	Peptococcaceae	unclassified_f__Peptococcaceae	unclassified_f__Peptococcaceae	OTU483	0	34	12	12
Tenericutes	Mollicutes	Mollicutes_RF9	norank_o__Mollicutes	norank_o__Mollicutes_RF9	uncultured_bacterium_g__norank_o__Mollicutes_RF9	OTU631	0	0	80	13

Firmicutes	Clostridia	Clostridiales	Ruminococcaceae	Caproiciproducens	unclassified_g__Caproiciproducens	OTU824	0	1	0	0
Firmicutes	Clostridia	Clostridiales	Clostridiales_vadinBB6	norank_f__Clostridiales_vadinBB60_	uncultured_bacterium_g__norank_f__Clostridiales_vadinBB60_group	OTU757	0	0	0	0
Tenericutes	Mollicutes	Mollicutes_RF9	norank_o__Mollicutes	norank_o__Mollicutes_RF9	uncultured_bacterium_g__norank_o__Mollicutes_RF9	OTU792	1	8	15	1
Firmicutes	Clostridia	Clostridiales	Lachnospiraceae	Coprococcus_1	uncultured_bacterium_g__Coprococcus_1	OTU629	0	0	63	3
Firmicutes	Clostridia	Clostridiales	Peptococcaceae	Peptococcus	uncultured_organism_g__Peptococcus	OTU665	0	11	14	19
Firmicutes	Clostridia	Clostridiales	Ruminococcaceae	Ruminococcaceae_UCG-014	unclassified_g__Ruminococcaceae_UCG-014	OTU532	0	0	0	0
Firmicutes	Clostridia	Clostridiales	Ruminococcaceae	norank_f__Ruminococcaceae	unclassified_g__norank_f__Ruminococcaceae	OTU671	1	15	157	6
Firmicutes	Clostridia	Clostridiales	Ruminococcaceae	Ruminococcus_1	uncultured_organism_g__Ruminococcus_1	OTU56	1	4	43	8
Firmicutes	Clostridia	Clostridiales	Clostridiales_vadinBB6	norank_f__Clostridiales_vadinBB60_	uncultured_bacterium_g__norank_f__Clostridiales_vadinBB60_group	OTU714	5	27	18	2
Firmicutes	Clostridia	Clostridiales	Lachnospiraceae	unclassified_f__Lachnospiraceae	unclassified_f__Lachnospiraceae	OTU726	2	2	13	10
Firmicutes	Clostridia	Clostridiales	Ruminococcaceae	norank_f__Ruminococcaceae	Ruminococcaceae_bacterium_GD7	OTU72	0	0	0	0
Firmicutes	Erysipelotrichia	Erysipelotrichales	Erysipelotrichaceae	Holdemania	unclassified_g__Holdemania	OTU702	3	15	1	7
Firmicutes	Clostridia	Clostridiales	Ruminococcaceae	[Eubacterium]_coprostanoligenes_g	unclassified_g__[Eubacterium]_coprostanoligenes_group	OTU747	0	0	158	3
Firmicutes	Clostridia	Clostridiales	Family_XIII	Family_XIII_AD3011_group	uncultured_bacterium_g__Family_XIII_AD3011_group	OTU579	4	27	5	5
Tenericutes	Mollicutes	Mollicutes_RF9	norank_o__Mollicutes	norank_o__Mollicutes_RF9	uncultured_bacterium_g__norank_o__Mollicutes_RF9	OTU756	14	1	0	18
Actinobacteria	Actinobacteria	Coriobacteriales	Coriobacteriaceae	unclassified_f__Coriobacteriaceae	unclassified_f__Coriobacteriaceae	OTU4	0	39	22	1
Firmicutes	Clostridia	Clostridiales	Ruminococcaceae	Candidatus_Soleaferrea	uncultured_organism_g__Candidatus_Soleaferrea	OTU492	1	8	4	3
Firmicutes	Clostridia	Clostridiales	Ruminococcaceae	[Eubacterium]_coprostanoligenes_g	unclassified_g__[Eubacterium]_coprostanoligenes_group	OTU656	0	15	8	9
Firmicutes	Erysipelotrichia	Erysipelotrichales	Erysipelotrichaceae	Erysipelatoclostridium	unclassified_g__Erysipelatoclostridium	OTU506	0	1	2	2
Firmicutes	Clostridia	Clostridiales	Ruminococcaceae	Anaerotruncus	uncultured_bacterium_g__Anaerotruncus	OTU42	2	11	7	10
Firmicutes	Clostridia	Clostridiales	Lachnospiraceae	unclassified_f__Lachnospiraceae	unclassified_f__Lachnospiraceae	OTU509	1	1	1	1
Firmicutes	Clostridia	Clostridiales	Lachnospiraceae	Tyzzerella	uncultured_bacterium_g__Tyzzerella	OTU777	0	0	11	7
Firmicutes	Clostridia	Clostridiales	Clostridiales_vadinBB6	norank_f__Clostridiales_vadinBB60_	gut_metagenome_g__norank_f__Clostridiales_vadinBB60_group	OTU19	2	13	0	0
Firmicutes	Clostridia	Clostridiales	Ruminococcaceae	Ruminococcus_2	unclassified_g__Ruminococcus_2	OTU549	0	0	1	0
Firmicutes	Clostridia	Clostridiales	Ruminococcaceae	Anaerofilum	uncultured_bacterium_g__Anaerofilum	OTU677	0	2	10	6
Firmicutes	Clostridia	Clostridiales	Ruminococcaceae	Ruminococcaceae_UCG-014	unclassified_g__Ruminococcaceae_UCG-014	OTU487	0	3	5	5
Firmicutes	Clostridia	Clostridiales	Ruminococcaceae	Ruminococcaceae_UCG-013	unclassified_g__Ruminococcaceae_UCG-013	OTU145	0	6	3	1
Bacteroidetes	Bacteroidia	Bacteroidales	Rikenellaceae	Alistipes	unclassified_g__Alistipes	OTU667	3	9	4	19
Firmicutes	Clostridia	Clostridiales	Lachnospiraceae	unclassified_f__Lachnospiraceae	unclassified_f__Lachnospiraceae	OTU771	0	1	1	2
Firmicutes	Clostridia	Clostridiales	Ruminococcaceae	Ruminococcaceae_UCG-009	uncultured_bacterium_g__Ruminococcaceae_UCG-009	OTU750	0	2	28	2
Tenericutes	Mollicutes	Mollicutes_RF9	norank_o__Mollicutes	norank_o__Mollicutes_RF9	uncultured_bacterium_g__norank_o__Mollicutes_RF9	OTU543	0	0	11	6
Firmicutes	Clostridia	Clostridiales	unclassified_o__Clostr	unclassified_o__Clostridiales	unclassified_o__Clostridiales	OTU535	5	0	2	4
Tenericutes	Mollicutes	Mollicutes_RF9	norank_o__Mollicutes	norank_o__Mollicutes_RF9	uncultured_bacterium_g__norank_o__Mollicutes_RF9	OTU153	0	0	61	0
Firmicutes	Clostridia	Clostridiales	Clostridiales_vadinBB6	norank_f__Clostridiales_vadinBB60_	uncultured_bacterium_g__norank_f__Clostridiales_vadinBB60_group	OTU409	1	26	14	1
Firmicutes	Clostridia	Clostridiales	Ruminococcaceae	Oscillibacter	unclassified_g__Oscillibacter	OTU609	0	0	5	4
Firmicutes	Clostridia	Clostridiales	Ruminococcaceae	Butyricicoccus	unclassified_g__Butyricicoccus	OTU411	1	2	0	4
Firmicutes	Clostridia	Clostridiales	unclassified_o__Clostr	unclassified_o__Clostridiales	unclassified_o__Clostridiales	OTU34	0	30	24	2
Firmicutes	Clostridia	Clostridiales	Clostridiales_vadinBB6	norank_f__Clostridiales_vadinBB60_	uncultured_rumen_bacterium_g__norank_f__Clostridiales_vadinBB60_group	OTU517	0	0	41	0
Firmicutes	Clostridia	Clostridiales	Ruminococcaceae	Ruminococcaceae_UCG-014	uncultured_bacterium_g__Ruminococcaceae_UCG-014	OTU850	1	56	37	0
Firmicutes	Clostridia	Clostridiales	Ruminococcaceae	Ruminiclostridium_5	uncultured_organism_g__Ruminiclostridium_5	OTU541	6	6	7	5
Firmicutes	Clostridia	Clostridiales	Lachnospiraceae	Tyzzerella_3	uncultured_bacterium_g__Tyzzerella_3	OTU635	3	14	11	16
Firmicutes	Clostridia	Clostridiales	Eubacteriaceae	Anaerofustis	Anaerofustis_sp._Marseille-P2441	OTU136	6	11	8	3
Firmicutes	Clostridia	Clostridiales	Ruminococcaceae	Ruminococcaceae_UCG-013	unclassified_g__Ruminococcaceae_UCG-013	OTU76	0	25	16	25
Firmicutes	Clostridia	Clostridiales	Ruminococcaceae	Anaerotruncus	unclassified_g__Anaerotruncus	OTU542	0	28	6	9
Firmicutes	Clostridia	Clostridiales	Ruminococcaceae	unclassified_f__Ruminococcaceae	unclassified_f__Ruminococcaceae	OTU831	8	7	3	5
Firmicutes	Clostridia	Clostridiales	Ruminococcaceae	Ruminococcaceae_NK4A214_group	unclassified_g__Ruminococcaceae_NK4A214_group	OTU168	0	3	29	1
Firmicutes	Clostridia	Clostridiales	Ruminococcaceae	Ruminococcaceae_UCG-009	unclassified_g__Ruminococcaceae_UCG-009	OTU71	0	0	11	10
Firmicutes	Clostridia	Clostridiales	Ruminococcaceae	Ruminococcaceae_UCG-013	uncultured_organism_g__Ruminococcaceae_UCG-013	OTU204	0	4	4	12
Actinobacteria	Actinobacteria	Coriobacteriales	Coriobacteriaceae	Enterorhabdus	uncultured_bacterium_g__Enterorhabdus	OTU221	2	22	1	3
Tenericutes	Mollicutes	Mollicutes_RF9	norank_o__Mollicutes	norank_o__Mollicutes_RF9	unclassified_g__norank_o__Mollicutes_RF9	OTU788	0	0	25	0
Firmicutes	Clostridia	Clostridiales	Ruminococcaceae	Ruminococcaceae_UCG-010	uncultured_organism_g__Ruminococcaceae_UCG-010	OTU20	0	23	20	10
Firmicutes	Clostridia	Clostridiales	Ruminococcaceae	Anaerotruncus	uncultured_bacterium_g__Anaerotruncus	OTU582	39	17	0	0
Firmicutes	Clostridia	Clostridiales	Family_XIII	Family_XIII_AD3011_group	uncultured_bacterium_g__Family_XIII_AD3011_group	OTU634	5	20	4	5
Firmicutes	Clostridia	Clostridiales	Ruminococcaceae	Anaerofilum	uncultured_bacterium_g__Anaerofilum	OTU832	39	0	0	0
Firmicutes	Clostridia	Clostridiales	Clostridiales_vadinBB6	norank_f__Clostridiales_vadinBB60_	uncultured_bacterium_g__norank_f__Clostridiales_vadinBB60_group	OTU38	0	250	0	0
Firmicutes	Clostridia	Clostridiales	unclassified_o__Clostr	unclassified_o__Clostridiales	unclassified_o__Clostridiales	OTU672	9	4	3	5
Firmicutes	Clostridia	Clostridiales	Ruminococcaceae	unclassified_f__Ruminococcaceae	unclassified_f__Ruminococcaceae	OTU661	0	15	10	7
Firmicutes	Clostridia	Clostridiales	Ruminococcaceae	unclassified_f__Ruminococcaceae	unclassified_f__Ruminococcaceae	OTU169	0	1	12	3
Tenericutes	Mollicutes	Mollicutes_RF9	norank_o__Mollicutes	norank_o__Mollicutes_RF9	uncultured_bacterium_g__norank_o__Mollicutes_RF9	OTU820	0	0	0	0
Firmicutes	Clostridia	Clostridiales	Eubacteriaceae	Eubacterium	Eubacterium_limosum	OTU337	0	5	89	0
Tenericutes	Mollicutes	Mollicutes_RF9	norank_o__Mollicutes	norank_o__Mollicutes_RF9	uncultured_bacterium_g__norank_o__Mollicutes_RF9	OTU584	1	0	3	4
Firmicutes	Clostridia	Clostridiales	Ruminococcaceae	Anaerofilum	uncultured_bacterium_g__Anaerofilum	OTU643	1	27	8	1
Firmicutes	Clostridia	Clostridiales	Clostridiales_vadinBB6	norank_f__Clostridiales_vadinBB60_	uncultured_bacterium_g__norank_f__Clostridiales_vadinBB60_group	OTU35	0	53	1	10
Firmicutes	Clostridia	Clostridiales	Lachnospiraceae	Coprococcus_1	uncultured_bacterium_g__Coprococcus_1	OTU811	0	2	2	10
Firmicutes	Clostridia	Clostridiales	Ruminococcaceae	Ruminococcaceae_UCG-014	unclassified_g__Ruminococcaceae_UCG-014	OTU87	0	0	21	13
Firmicutes	Clostridia	Clostridiales	Ruminococcaceae	[Eubacterium]_coprostanoligenes_g	unclassified_g__[Eubacterium]_coprostanoligenes_group	OTU751	0	0	0	9
Firmicutes	Clostridia	Clostridiales	Clostridiales_vadinBB6	norank_f__Clostridiales_vadinBB60_	uncultured_bacterium_g__norank_f__Clostridiales_vadinBB60_group	OTU694	0	0	0	0
Firmicutes	Clostridia	Clostridiales	Clostridiales_vadinBB6	norank_f__Clostridiales_vadinBB60_	uncultured_Thermoanaerobacterales_bacterium_g__norank_f__Clostridiales_vadinBB60_gro	OTU224	1	17	2	4
Firmicutes	Clostridia	Clostridiales	Lachnospiraceae	unclassified_f__Lachnospiraceae	unclassified_f__Lachnospiraceae	OTU655	0	2	10	3
Firmicutes	Clostridia	Clostridiales	Ruminococcaceae	Oscillospira	uncultured_bacterium_g__Oscillospira	OTU574	0	0	38	12
Firmicutes	Clostridia	Clostridiales	Clostridiales_vadinBB6	norank_f__Clostridiales_vadinBB60_	unclassified_g__norank_f__Clostridiales_vadinBB60_group	OTU2	0	0	1	0
Firmicutes	Clostridia	Clostridiales	Clostridiales_vadinBB6	norank_f__Clostridiales_vadinBB60_	unclassified_g__norank_f__Clostridiales_vadinBB60_group	OTU778	0	0	0	0
Firmicutes	Clostridia	Clostridiales	Ruminococcaceae	Ruminococcaceae_UCG-013	unclassified_g__Ruminococcaceae_UCG-013	OTU676	0	0	0	4
Tenericutes	Mollicutes	Mollicutes_RF9	norank_o__Mollicutes	norank_o__Mollicutes_RF9	unclassified_g__norank_o__Mollicutes_RF9	OTU626	0	5	20	4
Firmicutes	Clostridia	Clostridiales	Ruminococcaceae	Ruminococcaceae_UCG-010	unclassified_g__Ruminococcaceae_UCG-010	OTU61	0	14	3	5
Firmicutes	Clostridia	Clostridiales	Ruminococcaceae	norank_f__Ruminococcaceae	unclassified_g__norank_f__Ruminococcaceae	OTU516	1	6	3	3
Firmicutes	Clostridia	Clostridiales	Ruminococcaceae	unclassified_f__Ruminococcaceae	unclassified_f__Ruminococcaceae	OTU228	0	10	9	6
Firmicutes	Clostridia	Clostridiales	Ruminococcaceae	Ruminococcaceae_UCG-010	uncultured_bacterium_g__Ruminococcaceae_UCG-010	OTU203	0	0	171	0
Firmicutes	Clostridia	Clostridiales	Ruminococcaceae	Anaerotruncus	uncultured_bacterium_g__Anaerotruncus	OTU853	1	0	0	5
Firmicutes	Clostridia	Thermoanaerobact	Thermoanaerobactera	Gelria	uncultured_bacterium_g__Gelria	OTU16	0	35	26	1
Firmicutes	Clostridia	Clostridiales	Clostridiales_vadinBB6	norank_f__Clostridiales_vadinBB60_	uncultured_bacterium_g__norank_f__Clostridiales_vadinBB60_group	OTU143	0	0	0	2
Firmicutes	Clostridia	Clostridiales	Family_XIII	unclassified_f__Family_XIII	unclassified_f__Family_XIII	OTU537	1	8	4	2
Firmicutes	Clostridia	Clostridiales	Family_XIII	[Eubacterium]_brachy_group	uncultured_bacterium_g__[Eubacterium]_brachy_group	OTU69	5	18	6	3
Firmicutes	Clostridia	Clostridiales	Ruminococcaceae	unclassified_f__Ruminococcaceae	unclassified_f__Ruminococcaceae	OTU14	1	6	2	33
Firmicutes	Clostridia	Clostridiales	Ruminococcaceae	Intestinimonas	uncultured_bacterium_g__Intestinimonas	OTU615	0	4	11	3
Firmicutes	Clostridia	Clostridiales	Lachnospiraceae	unclassified_f__Lachnospiraceae	unclassified_f__Lachnospiraceae	OTU118	1	8	4	5
Firmicutes	Clostridia	Clostridiales	Ruminococcaceae	Anaerotruncus	unclassified_g__Anaerotruncus	OTU505	0	4	0	0
Firmicutes	Bacilli	Lactobacillales	Lactobacillaceae	Lactobacillus	uncultured_bacterium_g__Lactobacillus	OTU637	0	0	0	0
Firmicutes	Clostridia	Clostridiales	Ruminococcaceae	Ruminococcaceae_V9D2013_group	uncultured_bacterium_g__Ruminococcaceae_V9D2013_group	OTU652	0	12	3	5
Firmicutes	Erysipelotrichia	Erysipelotrichales	Erysipelotrichaceae	norank_f__Erysipelotrichaceae	uncultured_Erysipelotrichales_bacterium_g__norank	OTU323	0	0	0	0
Firmicutes	Clostridia	Clostridiales	Ruminococcaceae	Papillibacter	uncultured_bacterium_g__Papillibacter	OTU715	3	6	6	5
Tenericutes	Mollicutes	Mollicutes_RF9	norank_o__Mollicutes	norank_o__Mollicutes_RF9	unclassified_g__norank_o__Mollicutes_RF9	OTU729	0	0	0	0
Cyanobacteria	Cyanobacteria	Gastranaerophilales	norank_o__Gastranae	norank_o__Gastranaerophilales	uncultured_bacterium_g__norank_o__Gastranaerophilales	OTU94	0	0	0	0
Firmicutes	Clostridia	Clostridiales	Ruminococcaceae	Ruminococcaceae_UCG-013	uncultured_bacterium_g__Ruminococcaceae_UCG-013	OTU780	0	0	10	2
Firmicutes	Clostridia	Clostridiales	Ruminococcaceae	Anaerotruncus	unclassified_g__Anaerotruncus	OTU723	0	10	5	3
Firmicutes	Clostridia	Clostridiales	Ruminococcaceae	Anaerotruncus	unclassified_g__Anaerotruncus	OTU101	0	1	1	5
Tenericutes	Mollicutes	Mollicutes_RF9	norank_o__Mollicutes	norank_o__Mollicutes_RF9	unclassified_g__norank_o__Mollicutes_RF9	OTU117	0	1	11	1
Firmicutes	Clostridia	Clostridiales	Ruminococcaceae	unclassified_f__Ruminococcaceae	unclassified_f__Ruminococcaceae	OTU93	0	0	0	5
Firmicutes	Clostridia	Clostridiales	Lachnospiraceae	unclassified_f__Lachnospiraceae	unclassified_f__Lachnospiraceae	OTU759	9	0	0	3
Firmicutes	Clostridia	Clostridiales	Ruminococcaceae	Ruminococcaceae_UCG-014	unclassified_g__Ruminococcaceae_UCG-014	OTU95	0	0	0	0
Firmicutes	Erysipelotrichia	Erysipelotrichales	Erysipelotrichaceae	Dielma	unclassified_g__Dielma	OTU173	0	0	0	0
Firmicutes	Clostridia	Clostridiales	Ruminococcaceae	Anaerotruncus	unclassified_g__Anaerotruncus	OTU124	0	2	6	4
Firmicutes	Clostridia	Clostridiales	Ruminococcaceae	norank_f__Ruminococcaceae	unclassified_g__norank_f__Ruminococcaceae	OTU134	10	0	0	2
Firmicutes	Clostridia	Clostridiales	Lachnospiraceae	unclassified_f__Lachnospiraceae	unclassified_f__Lachnospiraceae	OTU510	2	0	2	1
Actinobacteria	Actinobacteria	Coriobacteriales	Coriobacteriaceae	Gordonibacter	unclassified_g__Gordonibacter	OTU461	6	30	3	3
Proteobacteria	Betaproteobacteria	Burkholderiales	Burkholderiaceae	Burkholderia-Paraburkholderia	unclassified_g__Burkholderia-Paraburkholderia	OTU435	0	0	0	0
Firmicutes	Clostridia	Clostridiales	Family_XIII	Family_XIII_AD3011_group	uncultured_bacterium_g__Family_XIII_AD3011_group	OTU648	0	0	0	2
Firmicutes	Clostridia	Clostridiales	Ruminococcaceae	unclassified_f__Ruminococcaceae	unclassified_f__Ruminococcaceae	OTU722	0	2	0	0
Firmicutes	Clostridia	Clostridiales	Clostridiales_vadinBB6	norank_f__Clostridiales_vadinBB60_	uncultured_bacterium_g__norank_f__Clostridiales_vadinBB60_group	OTU740	0	0	0	0
Bacteroidetes	Bacteroidia	Bacteroidales	Bacteroidales_S24-7_g	norank_f__Bacteroidales_S24-7_gro	uncultured_bacterium_g__norank_f__Bacteroidales_S24-7_group	OTU322	0	0	0	0
Firmicutes	Clostridia	Clostridiales	Ruminococcaceae	unclassified_f__Ruminococcaceae	unclassified_f__Ruminococcaceae	OTU538	0	0	2	2

Firmicutes	Clostridia	Clostridiales	Lachnospiraceae	unclassified_f__Lachnospiraceae	unclassified_f__Lachnospiraceae	OTU255	0	0	3	4
Firmicutes	Clostridia	Clostridiales	Christensenellaceae	Christensenellaceae_R-7_group	unclassified_g__Christensenellaceae_R-7_group	OTU106	0	0	13	0
Firmicutes	Clostridia	Clostridiales	Ruminococcaceae	[Eubacterium]_coprostanoligenes_gr	uncultured_bacterium_g__[Eubacterium]_coprostanoligenes_group	OTU129	0	0	4	0
Firmicutes	Erysipelotrichia	Erysipelotrichales	Erysipelotrichaceae	Erysipelatoclostridium	Clostridiales_bacterium_CHKCI006	OTU232	0	0	1	9
Firmicutes	Clostridia	Clostridiales	Ruminococcaceae	unclassified_f__Ruminococcaceae	unclassified_f__Ruminococcaceae	OTU518	4	0	1	4
Firmicutes	Clostridia	Clostridiales	Ruminococcaceae	Ruminococcaceae_UCG-013	uncultured_Clostridiaceae_bacterium_g__Ruminococcaceae_UCG-013	OTU675	0	0	3	27
Firmicutes	Clostridia	Clostridiales	Christensenellaceae	Christensenellaceae_R-7_group	human_gut_metagenome_g__Christensenellaceae_R-7_group	OTU223	0	0	0	0
Firmicutes	Clostridia	Clostridiales	Ruminococcaceae	Faecalibacterium	uncultured_bacterium_g__Faecalibacterium	OTU41	1	1	5	2
Firmicutes	Clostridia	Clostridiales	Ruminococcaceae	Anaerotruncus	unclassified_g__Anaerotruncus	OTU647	0	0	0	0
Firmicutes	Erysipelotrichia	Erysipelotrichales	Erysipelotrichaceae	Turicibacter	uncultured_bacterium_g__Turicibacter	OTU598	0	0	0	0
Firmicutes	Clostridia	Clostridiales	Ruminococcaceae	Subdoligranulum	unclassified_g__Subdoligranulum	OTU188	3	3	0	6
Firmicutes	Clostridia	Clostridiales	Ruminococcaceae	Anaerotruncus	uncultured_Clostridiaceae_bacterium_g__Anaerotruncus	OTU235	0	3	3	0
Firmicutes	Clostridia	Clostridiales	Family_XIII	unclassified_f__Family_XIII	unclassified_f__Family_XIII	OTU236	0	0	7	1
Firmicutes	Clostridia	Clostridiales	Ruminococcaceae	Ruminococcaceae_UCG-014	unidentified_rumen_bacterium_JW32	OTU21	0	19	11	0
Firmicutes	Clostridia	Clostridiales	Ruminococcaceae	Papillibacter	unclassified_g__Papillibacter	OTU521	0	0	3	2
Firmicutes	Clostridia	Clostridiales	Ruminococcaceae	unclassified_f__Ruminococcaceae	unclassified_f__Ruminococcaceae	OTU653	0	9	6	3
Firmicutes	Clostridia	Clostridiales	Peptococcaceae	norank_f__Peptococcaceae	uncultured_bacterium_g__norank_f__Peptococcaceae	OTU605	0	0	6	0
Firmicutes	Bacilli	Lactobacillales	Lactobacillaceae	Lactobacillus	Lactobacillus_ingluviei	OTU182	0	0	0	0
Firmicutes	Clostridia	Clostridiales	Ruminococcaceae	[Eubacterium]_coprostanoligenes_gr	unclassified_g__[Eubacterium]_coprostanoligenes_group	OTU333	0	0	0	0
Firmicutes	Clostridia	Clostridiales	unclassified_o__Clostr	unclassified_o__Clostridiales	unclassified_o__Clostridiales	OTU472	3	1	0	3
Firmicutes	Clostridia	Clostridiales	Ruminococcaceae	[Eubacterium]_coprostanoligenes_gr	unclassified_g__[Eubacterium]_coprostanoligenes_group	OTU730	2	14	1	2
Firmicutes	Clostridia	Clostridiales	Peptococcaceae	norank_f__Peptococcaceae	unclassified_g__norank_f__Peptococcaceae	OTU172	0	0	6	5
Firmicutes	Clostridia	Clostridiales	Ruminococcaceae	Ruminococcaceae_UCG-013	uncultured_bacterium_g__Ruminococcaceae_UCG-013	OTU658	0	0	0	0
Proteobacteria	Gammaproteobacteria	Enterobacteriales	Enterobacteriaceae	Proteus	Proteus_mirabilis	OTU785	0	0	0	0
Firmicutes	Clostridia	Clostridiales	Ruminococcaceae	norank_f__Ruminococcaceae	unclassified_g__norank_f__Ruminococcaceae	OTU744	0	5	1	0
Firmicutes	Clostridia	Clostridiales	Lachnospiraceae	Tyzzerella_3	unclassified_g__Tyzzerella_3	OTU841	0	0	0	4
Bacteroidetes	Bacteroidia	Bacteroidales	Bacteroidales_S24-7_g	norank_f__Bacteroidales_S24-7_gro	uncultured_bacterium_g__norank_f__Bacteroidales_S24-7_group	OTU360	0	0	0	0
Firmicutes	Clostridia	Clostridiales	Ruminococcaceae	unclassified_f__Ruminococcaceae	unclassified_f__Ruminococcaceae	OTU737	0	0	6	3
Firmicutes	Clostridia	Clostridiales	Ruminococcaceae	Anaerotruncus	unclassified_g__Anaerotruncus	OTU752	0	1	4	2
Firmicutes	Clostridia	Clostridiales	Lachnospiraceae	unclassified_f__Lachnospiraceae	unclassified_f__Lachnospiraceae	OTU63	0	1	1	0
Firmicutes	Clostridia	Clostridiales	Ruminococcaceae	Caproiciproducens	unclassified_g__Caproiciproducens	OTU452	0	30	0	2
Firmicutes	Clostridia	Clostridiales	Ruminococcaceae	Butyricicoccus	unclassified_g__Butyricicoccus	OTU475	0	0	2	2
Proteobacteria	Betaproteobacteria	Burkholderiales	Oxalobacteraceae	Herminiimonas	uncultured_bacterium_g__Herminiimonas	OTU804	0	4	6	1
Tenericutes	Mollicutes	Mollicutes_RF9	norank_o__Mollicutes	norank_o__Mollicutes_RF9	unclassified_g__norank_o__Mollicutes_RF9	OTU630	0	0	10	0
Firmicutes	Clostridia	Clostridiales	Ruminococcaceae	Ruminococcaceae_UCG-013	unclassified_g__Ruminococcaceae_UCG-013	OTU680	0	0	5	1
Tenericutes	Mollicutes	Mollicutes_RF9	norank_o__Mollicutes	norank_o__Mollicutes_RF9	gut_metagenome_g__norank_o__Mollicutes_RF9	OTU773	0	0	0	0
Firmicutes	Clostridia	Clostridiales	Peptococcaceae	norank_f__Peptococcaceae	uncultured_bacterium_g__norank_f__Peptococcaceae	OTU624	0	21	0	1
Firmicutes	Clostridia	Clostridiales	Ruminococcaceae	Ruminiclostridium	unclassified_g__Ruminiclostridium	OTU154	0	4	6	0
Bacteroidetes	Bacteroidia	Bacteroidales	Bacteroidales_S24-7_g	norank_f__Bacteroidales_S24-7_gro	unclassified_g__norank_f__Bacteroidales_S24-7_group	OTU184	0	0	0	0
Firmicutes	Clostridia	Clostridiales	Ruminococcaceae	Ruminococcaceae_UCG-014	unclassified_g__Ruminococcaceae_UCG-014	OTU524	4	0	0	4
Firmicutes	Clostridia	Clostridiales	Peptococcaceae	norank_f__Peptococcaceae	uncultured_bacterium_g__norank_f__Peptococcaceae	OTU115	0	12	3	2
Firmicutes	Clostridia	Clostridiales	Lachnospiraceae	unclassified_f__Lachnospiraceae	unclassified_f__Lachnospiraceae	OTU127	0	0	0	1
Firmicutes	Clostridia	Clostridiales	Lachnospiraceae	unclassified_f__Lachnospiraceae	unclassified_f__Lachnospiraceae	OTU664	0	1	5	1
Firmicutes	Clostridia	Clostridiales	Lachnospiraceae	unclassified_f__Lachnospiraceae	unclassified_f__Lachnospiraceae	OTU465	2	0	2	2
Firmicutes	Clostridia	Clostridiales	Ruminococcaceae	Ruminococcaceae_UCG-005	uncultured_Clostridiales_bacterium_g__Ruminococcaceae_UCG-005	OTU689	0	8	9	0
Tenericutes	Mollicutes	Mollicutes_RF9	norank_o__Mollicutes	norank_o__Mollicutes_RF9	uncultured_bacterium_g__norank_o__Mollicutes_RF9	OTU52	0	0	3	0
Firmicutes	Clostridia	Clostridiales	Ruminococcaceae	[Eubacterium]_coprostanoligenes_gr	unclassified_g__[Eubacterium]_coprostanoligenes_group	OTU74	0	2	0	0
Firmicutes	Clostridia	Clostridiales	Lachnospiraceae	Coprococcus_1	unclassified_g__Coprococcus_1	OTU659	0	0	0	4
Firmicutes	Clostridia	Clostridiales	Lachnospiraceae	unclassified_f__Lachnospiraceae	unclassified_f__Lachnospiraceae	OTU688	0	0	3	0
Firmicutes	Clostridia	Clostridiales	Ruminococcaceae	unclassified_f__Ruminococcaceae	unclassified_f__Ruminococcaceae	OTU732	0	0	0	0
Firmicutes	Clostridia	Clostridiales	Christensenellaceae	Christensenellaceae_R-7_group	uncultured_organism_g__Christensenellaceae_R-7_group	OTU684	2	1	2	8
Bacteroidetes	Bacteroidia	Bacteroidales	Porphyromonadaceae	Odoribacter	uncultured_bacterium_g__Odoribacter	OTU327	0	0	0	0
Firmicutes	Clostridia	Clostridiales	Clostridiales_vadinBB6	norank_f__Clostridiales_vadinBB60_	uncultured_bacterium_g__norank_f__Clostridiales_vadinBB60_group	OTU325	0	9	1	0
Firmicutes	Clostridia	Clostridiales	Lachnospiraceae	unclassified_f__Lachnospiraceae	unclassified_f__Lachnospiraceae	OTU551	0	0	14	0
Firmicutes	Bacilli	Lactobacillales	Enterococcaceae	Enterococcus	unclassified_g__Enterococcus	OTU237	2	0	0	2
Bacteroidetes	Bacteroidia	Bacteroidales	Prevotellaceae	Alloprevotella	gut_metagenome_g__Alloprevotella	OTU312	0	0	0	0
Firmicutes	Clostridia	Clostridiales	Ruminococcaceae	unclassified_f__Ruminococcaceae	unclassified_f__Ruminococcaceae	OTU855	8	2	1	4
Actinobacteria	Actinobacteria	Bifidobacteriales	Bifidobacteriaceae	Bifidobacterium	Bifidobacterium_pseudolongum_PV8-2	OTU468	0	0	0	0
Firmicutes	Clostridia	Clostridiales	Christensenellaceae	norank_f__Christensenellaceae	unclassified_g__norank_f__Christensenellaceae	OTU529	0	4	0	1
Tenericutes	Mollicutes	Mollicutes_RF9	norank_o__Mollicutes	norank_o__Mollicutes_RF9	unclassified_g__norank_o__Mollicutes_RF9	OTU764	0	0	3	4
Firmicutes	Clostridia	Clostridiales	Ruminococcaceae	norank_f__Ruminococcaceae	unclassified_g__norank_f__Ruminococcaceae	OTU805	0	9	13	0
Firmicutes	Clostridia	Clostridiales	Ruminococcaceae	Faecalibacterium	uncultured_bacterium_g__Faecalibacterium	OTU60	0	8	1	0
Firmicutes	Clostridia	Clostridiales	Ruminococcaceae	unclassified_f__Ruminococcaceae	unclassified_f__Ruminococcaceae	OTU530	0	15	0	1
Firmicutes	Clostridia	Clostridiales	Peptococcaceae	norank_f__Peptococcaceae	uncultured_bacterium_g__norank_f__Peptococcaceae	OTU619	0	1	0	0
Firmicutes	Clostridia	Clostridiales	Ruminococcaceae	unclassified_f__Ruminococcaceae	unclassified_f__Ruminococcaceae	OTU471	0	0	0	0
Firmicutes	Clostridia	Clostridiales	Ruminococcaceae	Candidatus_Soleaferrea	uncultured_bacterium_g__Candidatus_Soleaferrea	OTU284	0	0	2	1
Firmicutes	Clostridia	Clostridiales	Ruminococcaceae	Anaerotruncus	unclassified_g__Anaerotruncus	OTU508	0	0	0	0
Firmicutes	Clostridia	Clostridiales	Ruminococcaceae	Anaerotruncus	uncultured_bacterium_g__Anaerotruncus	OTU704	0	2	2	2
Tenericutes	Mollicutes	Mollicutes_RF9	norank_o__Mollicutes	norank_o__Mollicutes_RF9	uncultured_bacterium_g__norank_o__Mollicutes_RF9	OTU783	0	0	1	1
Firmicutes	Clostridia	Clostridiales	Eubacteriaceae	Anaerofustis	uncultured_bacterium_g__Anaerofustis	OTU111	0	0	0	2
Firmicutes	Clostridia	Clostridiales	Clostridiales_vadinBB6	norank_f__Clostridiales_vadinBB60_	unclassified_g__norank_f__Clostridiales_vadinBB60_group	OTU645	0	0	11	1
Firmicutes	Clostridia	Clostridiales	Peptococcaceae	norank_f__Peptococcaceae	uncultured_bacterium_g__norank_f__Peptococcaceae	OTU27	0	3	5	1
Firmicutes	Clostridia	Clostridiales	Ruminococcaceae	Anaerotruncus	unclassified_g__Anaerotruncus	OTU809	0	0	1	0
Bacteroidetes	Bacteroidia	Bacteroidales	Rikenellaceae	Rikenellaceae_RC9_gut_group	unclassified_g__Rikenellaceae_RC9_gut_group	OTU321	0	0	0	0
Firmicutes	Clostridia	Clostridiales	Ruminococcaceae	Ruminococcaceae_UCG-005	unclassified_g__Ruminococcaceae_UCG-005	OTU482	0	2	3	3
Tenericutes	Mollicutes	Mollicutes_RF9	norank_o__Mollicutes	norank_o__Mollicutes_RF9	uncultured_Erysipelotrichaceae_bacterium_g__norank_o__Mollicutes_RF9	OTU558	0	0	7	0
Firmicutes	Clostridia	Clostridiales	Clostridiaceae_1	Candidatus_Arthromitus	Candidatus_Arthromitus_sp._SFB-mouse-Japan	OTU162	3	1	2	4
Firmicutes	Clostridia	Clostridiales	Ruminococcaceae	Subdoligranulum	unclassified_g__Subdoligranulum	OTU810	0	2	14	0
Firmicutes	Clostridia	Clostridiales	Ruminococcaceae	norank_f__Ruminococcaceae	unclassified_g__norank_f__Ruminococcaceae	OTU666	0	0	2	1
Firmicutes	Clostridia	Clostridiales	Ruminococcaceae	unclassified_f__Ruminococcaceae	unclassified_f__Ruminococcaceae	OTU693	0	1	0	3
Firmicutes	Clostridia	Clostridiales	Ruminococcaceae	unclassified_f__Ruminococcaceae	unclassified_f__Ruminococcaceae	OTU858	0	0	2	3
Firmicutes	Clostridia	Clostridiales	Ruminococcaceae	Ruminiclostridium_5	unclassified_g__Ruminiclostridium_5	OTU470	2	2	0	1
Firmicutes	Negativicutes	Selenomonadales	Acidaminococcaceae	Phascolarctobacterium	uncultured_organism_g__Phascolarctobacterium	OTU787	2	4	26	1
Firmicutes	Clostridia	Clostridiales	Christensenellaceae	Christensenellaceae_R-7_group	unclassified_g__Christensenellaceae_R-7_group	OTU745	0	0	0	6
Firmicutes	Clostridia	Clostridiales	Peptococcaceae	norank_f__Peptococcaceae	unclassified_g__norank_f__Peptococcaceae	OTU495	0	1	4	4
Firmicutes	Clostridia	Clostridiales	Ruminococcaceae	Ruminococcaceae_UCG-010	uncultured_bacterium_g__Ruminococcaceae_UCG-010	OTU18	0	7	4	0
Firmicutes	Clostridia	Clostridiales	Ruminococcaceae	Ruminococcaceae_UCG-013	unclassified_g__Ruminococcaceae_UCG-013	OTU89	0	0	1	52
Firmicutes	Clostridia	Clostridiales	Ruminococcaceae	Ruminiclostridium_5	unclassified_g__Ruminiclostridium_5	OTU649	1	2	3	1
Firmicutes	Clostridia	Clostridiales	Lachnospiraceae	unclassified_f__Lachnospiraceae	unclassified_f__Lachnospiraceae	OTU324	3	0	0	3
Firmicutes	Clostridia	Clostridiales	Ruminococcaceae	Ruminococcaceae_UCG-013	unclassified_g__Ruminococcaceae_UCG-013	OTU31	0	2	0	0
Firmicutes	Clostridia	Clostridiales	Ruminococcaceae	Ruminococcus_1	uncultured_organism_g__Ruminococcus_1	OTU651	0	0	0	0
Firmicutes	Clostridia	Clostridiales	Peptococcaceae	unclassified_f__Peptococcaceae	unclassified_f__Peptococcaceae	OTU768	0	0	0	3
Firmicutes	Clostridia	Clostridiales	Christensenellaceae	unclassified_f__Christensenellaceae	unclassified_f__Christensenellaceae	OTU857	0	0	2	0
Tenericutes	Mollicutes	Mollicutes_RF9	norank_o__Mollicutes	norank_o__Mollicutes_RF9	unclassified_g__norank_o__Mollicutes_RF9	OTU758	0	0	6	0
Firmicutes	Clostridia	Clostridiales	Ruminococcaceae	Candidatus_Soleaferrea	uncultured_bacterium_g__Candidatus_Soleaferrea	OTU791	1	4	3	2
unclassified_k__r	unclassified_k__norank	unclassified_k__nor	unclassified_k__noran	unclassified_k__norank	unclassified_k__norank	OTU9	5	0	0	3
Firmicutes	Clostridia	Clostridiales	Ruminococcaceae	unclassified_f__Ruminococcaceae	unclassified_f__Ruminococcaceae	OTU733	0	0	1	0
Firmicutes	Clostridia	Clostridiales	Peptococcaceae	norank_f__Peptococcaceae	unclassified_g__norank_f__Peptococcaceae	OTU90	0	0	0	0
Firmicutes	Clostridia	Clostridiales	Lachnospiraceae	unclassified_f__Lachnospiraceae	unclassified_f__Lachnospiraceae	OTU644	0	0	0	0
Firmicutes	Clostridia	Clostridiales	Ruminococcaceae	[Eubacterium]_coprostanoligenes_gr	unclassified_g__[Eubacterium]_coprostanoligenes_group	OTU29	0	1	1	1
Firmicutes	Clostridia	Clostridiales	Ruminococcaceae	Ruminococcaceae_UCG-013	uncultured_Clostridiaceae_bacterium_g__Ruminococcaceae_UCG-013	OTU152	0	0	0	1
Firmicutes	Clostridia	Clostridiales	Ruminococcaceae	Faecalibacterium	unclassified_g__Faecalibacterium	OTU243	0	0	0	0
Bacteroidetes	Bacteroidia	Bacteroidales	Bacteroidales_S24-7_g	norank_f__Bacteroidales_S24-7_gro	uncultured_Bacteroidales_bacterium_g__norank_f__Bacteroidales_S24-7_group	OTU339	0	0	0	0
Tenericutes	Mollicutes	Mollicutes_RF9	norank_o__Mollicutes	norank_o__Mollicutes_RF9	unclassified_g__norank_o__Mollicutes_RF9	OTU51	0	0	9	0
Firmicutes	Clostridia	Clostridiales	Christensenellaceae	Christensenellaceae_R-7_group	uncultured_organism_g__Christensenellaceae_R-7_group	OTU332	0	0	0	0
Firmicutes	Clostridia	Clostridiales	Ruminococcaceae	Anaerotruncus	unclassified_g__Anaerotruncus	OTU194	0	4	0	3
Firmicutes	Clostridia	Clostridiales	Ruminococcaceae	Ruminococcaceae_UCG-013	uncultured_bacterium_g__Ruminococcaceae_UCG-013	OTU334	0	0	0	0
Firmicutes	Clostridia	Clostridiales	Ruminococcaceae	Ruminococcaceae_NK4A214_group	unclassified_g__Ruminococcaceae_NK4A214_group	OTU32	0	2	2	2
Firmicutes	Clostridia	Clostridiales	Christensenellaceae	Christensenellaceae_R-7_group	unclassified_g__Christensenellaceae_R-7_group	OTU105	0	2	0	0
Firmicutes	Bacilli	Bacillales	Staphylococcaceae	Staphylococcus	Staphylococcus_warneri	OTU597	0	1	1	0
Firmicutes	Bacilli	Bacillales	Staphylococcaceae	Staphylococcus	Staphylococcus_lentus_g__Staphylococcus	OTU432	0	0	0	0

Firmicutes	Clostridia	Clostridiales	Ruminococcaceae	unclassified_f__Ruminococcaceae	unclassified_f__Ruminococcaceae	OTU698	0	0	0	0
Tenericutes	Mollicutes	Mollicutes_RF9	norank_o__Mollicutes	norank_o__Mollicutes_RF9	unclassified_g__norank_o__Mollicutes_RF9	OTU781	0	0	26	0
Firmicutes	Clostridia	Clostridiales	Ruminococcaceae	Anaerotruncus	uncultured_bacterium_g__Anaerotruncus	OTU53	0	0	7	0
Actinobacteria	Actinobacteria	Micrococcales	Intrasporangiaceae	Terrabacter	uncultured_bacterium_g__Terrabacter	OTU270	0	0	0	0
Firmicutes	Clostridia	Clostridiales	Ruminococcaceae	unclassified_f__Ruminococcaceae	unclassified_f__Ruminococcaceae	OTU681	0	4	1	1
Bacteroidetes	Bacteroidia	Bacteroidales	Bacteroidales_S24-7_g	norank_f__Bacteroidales_S24-7_gro	uncultured_bacterium_g__norank_f__Bacteroidales_S24-7_group	OTU310	0	0	0	0
Firmicutes	Clostridia	Clostridiales	Clostridiales_vadinBB6	norank_f__Clostridiales_vadinBB60_	unclassified_g__norank_f__Clostridiales_vadinBB60_group	OTU528	0	0	0	0
Firmicutes	Clostridia	Clostridiales	Ruminococcaceae	Ruminiclostridium	uncultured_bacterium_g__Ruminiclostridium	OTU633	0	0	3	2
Firmicutes	Clostridia	Clostridiales	Christensenellaceae	Christensenellaceae_R-7_group	unclassified_g__Christensenellaceae_R-7_group	OTU695	1	0	2	0
Cyanobacteria	Cyanobacteria	norank_c__Cyanoba	norank_c__Cyanobact	norank_c__Cyanobacteria	unclassified_g__norank_c__Cyanobacteria	OTU258	0	0	0	0
Bacteroidetes	Bacteroidia	Bacteroidales	Prevotellaceae	Prevotellaceae_UCG-001	uncultured_Bacteroidales_bacterium_g__Prevotellaceae_UCG-001	OTU276	0	0	0	0
Firmicutes	Bacilli	Lactobacillales	Lactobacillaceae	Lactobacillus	unclassified_g__Lactobacillus	OTU632	1	0	0	3
Firmicutes	Clostridia	Clostridiales	Ruminococcaceae	Ruminiclostridium_9	Clostridiales_bacterium_NK3B98_g__Ruminiclostridium_9	OTU130	0	0	0	0
Firmicutes	Clostridia	Clostridiales	Ruminococcaceae	Ruminococcaceae_UCG-013	uncultured_bacterium_g__Ruminococcaceae_UCG-013	OTU807	0	0	0	0
Bacteroidetes	Bacteroidia	Bacteroidales	Rikenellaceae	Alistipes	gut_metagenome_g__Alistipes	OTU808	1	2	0	0
Firmicutes	Clostridia	unclassified_c__Clo	unclassified_c__Clostr	unclassified_c__Clostridia	unclassified_c__Clostridia	OTU99	0	2	0	0
Firmicutes	Clostridia	Clostridiales	Christensenellaceae	norank_f__Christensenellaceae	unclassified_g__norank_f__Christensenellaceae	OTU553	1	0	0	0
Firmicutes	Clostridia	Clostridiales	Lachnospiraceae	unclassified_f__Lachnospiraceae	unclassified_f__Lachnospiraceae	OTU80	5	0	0	3
Bacteroidetes	Bacteroidia	Bacteroidales	Prevotellaceae	Paraprevotella	unclassified_g__Paraprevotella	OTU308	0	0	0	0
Firmicutes	Clostridia	Clostridiales	Peptococcaceae	norank_f__Peptococcaceae	unclassified_g__norank_f__Peptococcaceae	OTU596	0	0	0	0
Firmicutes	Clostridia	Clostridiales	Lachnospiraceae	Lachnospiraceae_NK4A136_group	unclassified_g__Lachnospiraceae_NK4A136_group	OTU251	0	0	0	0
Firmicutes	Clostridia	Clostridiales	Family_XIII	[Eubacterium]_brachy_group	uncultured_bacterium_g__[Eubacterium]_brachy_group	OTU335	0	0	0	0
Firmicutes	Clostridia	Clostridiales	Lachnospiraceae	unclassified_f__Lachnospiraceae	unclassified_f__Lachnospiraceae	OTU86	0	2	0	8
Firmicutes	Clostridia	Clostridiales	Ruminococcaceae	Ruminococcaceae_UCG-005	uncultured_bacterium_adhufec311	OTU215	0	0	0	1
Firmicutes	Clostridia	Clostridiales	Ruminococcaceae	Ruminococcaceae_UCG-009	uncultured_bacterium_g__Ruminococcaceae_UCG-009	OTU641	0	0	2	0
Firmicutes	Clostridia	Clostridiales	Ruminococcaceae	Ruminiclostridium_1	unclassified_g__Ruminiclostridium_1	OTU178	0	0	5	0
Actinobacteria	Actinobacteria	Micrococcales	Micrococcaceae	Pseudarthrobacter	unclassified_g__Pseudarthrobacter	OTU423	0	0	0	0
Firmicutes	Clostridia	Clostridiales	Ruminococcaceae	Ruminococcaceae_UCG-010	unclassified_g__Ruminococcaceae_UCG-010	OTU26	0	10	2	0
Firmicutes	Clostridia	Clostridiales	Clostridiaceae_1	Clostridium_sensu_stricto_1	uncultured_bacterium_g__Clostridium_sensu_stricto_1	OTU128	0	0	0	0
Firmicutes	Clostridia	Clostridiales	Ruminococcaceae	Anaerotruncus	uncultured_bacterium_g__Anaerotruncus	OTU245	1	0	0	4
Firmicutes	Clostridia	Clostridiales	Ruminococcaceae	Ruminococcaceae_UCG-014	unclassified_g__Ruminococcaceae_UCG-014	OTU429	0	0	0	0
Firmicutes	Erysipelotrichia	Erysipelotrichales	Erysipelotrichaceae	Faecalibaculum	uncultured_bacterium_g__Faecalibaculum	OTU497	0	0	0	0
Firmicutes	Clostridia	Clostridiales	Ruminococcaceae	unclassified_f__Ruminococcaceae	unclassified_f__Ruminococcaceae	OTU525	0	0	0	0
Firmicutes	Clostridia	Clostridiales	Ruminococcaceae	Anaerotruncus	unclassified_g__Anaerotruncus	OTU640	0	0	0	0
Actinobacteria	Actinobacteria	Coriobacteriales	Coriobacteriaceae	Coriobacteriaceae_UCG-002	uncultured_bacterium_g__Coriobacteriaceae_UCG-002	OTU365	0	0	0	0
Firmicutes	Clostridia	Clostridiales	Christensenellaceae	Christensenellaceae_R-7_group	unclassified_g__Christensenellaceae_R-7_group	OTU670	0	0	1	0
Firmicutes	Clostridia	Clostridiales	Ruminococcaceae	Ruminococcaceae_UCG-010	uncultured_bacterium_g__Ruminococcaceae_UCG-010	OTU776	0	0	0	0
Firmicutes	Clostridia	Clostridiales	Christensenellaceae	norank_f__Christensenellaceae	uncultured_bacterium_g__norank_f__Christensenellaceae	OTU139	0	0	0	0
Firmicutes	Clostridia	Clostridiales	Christensenellaceae	Christensenellaceae_R-7_group	uncultured_bacterium_g__Christensenellaceae_R-7_group	OTU207	0	0	0	0
Actinobacteria	Actinobacteria	Coriobacteriales	Coriobacteriaceae	unclassified_f__Coriobacteriaceae	unclassified_f__Coriobacteriaceae	OTU477	0	1	0	0
Firmicutes	Clostridia	Clostridiales	Ruminococcaceae	unclassified_f__Ruminococcaceae	unclassified_f__Ruminococcaceae	OTU176	0	0	0	1
Bacteroidetes	Bacteroidia	Bacteroidales	Bacteroidales_S24-7_g	norank_f__Bacteroidales_S24-7_gro	uncultured_bacterium_g__norank_f__Bacteroidales_S24-7_group	OTU262	0	0	0	0
Firmicutes	Clostridia	Clostridiales	Ruminococcaceae	Anaerotruncus	unclassified_g__Anaerotruncus	OTU444	0	0	0	0
Firmicutes	Clostridia	Clostridiales	Ruminococcaceae	Ruminococcaceae_UCG-013	uncultured_Clostridiales_bacterium_g__Ruminococcaceae_UCG-013	OTU522	0	0	0	0
Firmicutes	Clostridia	Clostridiales	Ruminococcaceae	Ruminococcaceae_UCG-010	uncultured_organism_g__Ruminococcaceae_UCG-010	OTU782	0	0	1	1
Saccharibacteria	Unknown_Class_p__Sacc	Unknown_Order_c_	Unknown_Family_o__	Candidatus_Saccharimonas	uncultured_bacterium_g__Candidatus_Saccharimonas	OTU261	0	0	0	0
Firmicutes	Clostridia	Clostridiales	Lachnospiraceae	unclassified_f__Lachnospiraceae	unclassified_f__Lachnospiraceae	OTU120	0	0	0	0
Chloroflexi	KD4-96	norank_c__KD4-96	norank_c__KD4-96	norank_c__KD4-96	unclassified_g__norank_c__KD4-96	OTU254	0	0	0	0
Firmicutes	Clostridia	Clostridiales	Clostridiales_vadinBB6	norank_f__Clostridiales_vadinBB60_	unclassified_g__norank_f__Clostridiales_vadinBB60_group	OTU209	0	2	0	0
Tenericutes	Mollicutes	Mollicutes_RF9	norank_o__Mollicutes	norank_o__Mollicutes_RF9	unclassified_g__norank_o__Mollicutes_RF9	OTU572	0	0	1	0
Firmicutes	Clostridia	Clostridiales	Ruminococcaceae	unclassified_f__Ruminococcaceae	unclassified_f__Ruminococcaceae	OTU195	0	0	0	0
Proteobacteria	Alphaproteobacteria	Sphingomonadales	Sphingomonadaceae	Sphingomonas	uncultured_Sphingomonadaceae_bacterium_g__Sphingomonas	OTU375	0	0	0	0
Tenericutes	Mollicutes	Mollicutes_RF9	norank_o__Mollicutes	norank_o__Mollicutes_RF9	unclassified_g__norank_o__Mollicutes_RF9	OTU727	1	0	2	0
Firmicutes	Erysipelotrichia	Erysipelotrichales	Erysipelotrichaceae	Turicibacter	uncultured_bacterium_g__Turicibacter	OTU40	0	0	0	0
Firmicutes	Clostridia	Clostridiales	Lachnospiraceae	Coprococcus_1	intestinal_bacterium_CG19-1	OTU45	0	1	1	0
Firmicutes	Clostridia	Clostridiales	Peptococcaceae	norank_f__Peptococcaceae	unclassified_g__norank_f__Peptococcaceae	OTU116	0	0	0	0
Firmicutes	Clostridia	Clostridiales	Peptococcaceae	norank_f__Peptococcaceae	unclassified_g__norank_f__Peptococcaceae	OTU199	0	0	2	0
Bacteroidetes	Bacteroidia	Bacteroidales	Bacteroidaceae	Bacteroides	Bacteroides_acidifaciens	OTU320	0	0	0	0
Firmicutes	Clostridia	Clostridiales	Ruminococcaceae	unclassified_f__Ruminococcaceae	unclassified_f__Ruminococcaceae	OTU552	0	10	1	0
Firmicutes	Clostridia	Clostridiales	Lachnospiraceae	Butyrivibrio	uncultured_organism_g__Butyrivibrio	OTU762	0	0	0	0
Tenericutes	Mollicutes	Mollicutes_RF9	norank_o__Mollicutes	norank_o__Mollicutes_RF9	uncultured_bacterium_g__norank_o__Mollicutes_RF9	OTU797	0	0	0	0
Firmicutes	Clostridia	Clostridiales	Lachnospiraceae	unclassified_f__Lachnospiraceae	unclassified_f__Lachnospiraceae	OTU852	0	2	0	0
Proteobacteria	Alphaproteobacteria	Rhizobiales	Bradyrhizobiaceae	Bradyrhizobium	Bradyrhizobium_elkanii	OTU185	0	0	1	0
Chloroflexi	Chloroflexia	Chloroflexales	Roseiflexaceae	Roseiflexus	uncultured_bacterium_g__Roseiflexus	OTU415	0	0	0	0
Proteobacteria	Alphaproteobacteria	Rhizobiales	Xanthobacteraceae	norank_f__Xanthobacteraceae	uncultured_bacterium_g__norank_f__Xanthobacteraceae	OTU427	0	0	0	0
Firmicutes	unclassified_p__Firmicut	unclassified_p__Fir	unclassified_p__Firmi	unclassified_p__Firmicutes	unclassified_p__Firmicutes	OTU454	0	0	0	0
Firmicutes	Clostridia	Clostridiales	Ruminococcaceae	Anaerotruncus	unclassified_g__Anaerotruncus	OTU621	0	0	2	0
Firmicutes	Clostridia	Clostridiales	Ruminococcaceae	Anaerotruncus	uncultured_Clostridiales_bacterium_g__Anaerotruncus	OTU736	0	0	0	3
Firmicutes	Clostridia	Clostridiales	Ruminococcaceae	Hydrogenoanaerobacterium	Selenomonadales_bacterium_Marseille-P2399	OTU5	0	0	0	2
Bacteroidetes	Bacteroidia	Bacteroidales	Bacteroidales_S24-7_g	norank_f__Bacteroidales_S24-7_gro	uncultured_bacterium_g__norank_f__Bacteroidales_S24-7_group	OTU300	0	0	0	0
Bacteroidetes	Bacteroidia	Bacteroidales	Prevotellaceae	Prevotellaceae_NK3B31_group	uncultured_bacterium_g__Prevotellaceae_NK3B31_group	OTU331	0	0	0	0
Proteobacteria	Betaproteobacteria	Burkholderiales	Burkholderiaceae	Ralstonia	unidentified_marine_bacterioplankton_g__Ralstonia	OTU593	0	2	0	0
Tenericutes	Mollicutes	Mollicutes_RF9	norank_o__Mollicutes	norank_o__Mollicutes_RF9	uncultured_Erysipelotrichia_bacterium_g__norank	OTU594	0	0	6	0
Firmicutes	Clostridia	Clostridiales	Ruminococcaceae	Anaerotruncus	unclassified_g__Anaerotruncus	OTU713	0	0	2	0
Firmicutes	Clostridia	Clostridiales	Clostridiales_vadinBB6	norank_f__Clostridiales_vadinBB60_	uncultured_bacterium_g__norank_f__Clostridiales_vadinBB60_group	OTU724	0	0	0	0
Firmicutes	Clostridia	Clostridiales	Christensenellaceae	unclassified_f__Christensenellaceae	unclassified_f__Christensenellaceae	OTU793	0	0	2	0
Firmicutes	Clostridia	Clostridiales	Lachnospiraceae	Tyzzerella	unclassified_g__Tyzzerella	OTU796	0	0	0	0
Actinobacteria	Actinobacteria	Bifidobacteriales	Bifidobacteriaceae	Bifidobacterium	Bifidobacterium_pseudocatenulatum_DSM_20438__JCM_1200__LMG_10505	OTU55	1	0	4	0
Firmicutes	Clostridia	Clostridiales	Clostridiales_vadinBB6	norank_f__Clostridiales_vadinBB60_	uncultured_bacterium_g__norank_f__Clostridiales_vadinBB60_group	OTU336	0	0	0	0
Bacteroidetes	Bacteroidia	Bacteroidales	Bacteroidaceae	Bacteroides	Bacteroides_sartorii	OTU345	0	0	0	0
Firmicutes	Clostridia	Clostridiales	Ruminococcaceae	Ruminiclostridium_5	unclassified_g__Ruminiclostridium_5	OTU818	0	0	1	0
Firmicutes	Clostridia	Clostridiales	Christensenellaceae	norank_f__Christensenellaceae	uncultured_bacterium_g__norank_f__Christensenellaceae	OTU144	0	0	2	0
Firmicutes	Clostridia	Clostridiales	Ruminococcaceae	[Eubacterium]_coprostanoligenes_g	unclassified_g__[Eubacterium]_coprostanoligenes_group	OTU212	0	1	0	0
Firmicutes	Clostridia	Clostridiales	Ruminococcaceae	Ruminococcaceae_UCG-013	unclassified_g__Ruminococcaceae_UCG-013	OTU342	0	0	0	0
Chloroflexi	Chloroflexia	Chloroflexales	Roseiflexaceae	Roseiflexus	uncultured_bacterium_g__Roseiflexus	OTU433	0	0	0	0
Firmicutes	Clostridia	Clostridiales	Ruminococcaceae	unclassified_f__Ruminococcaceae	unclassified_f__Ruminococcaceae	OTU646	0	0	0	0
Firmicutes	Bacilli	Lactobacillales	Lactobacillaceae	Lactobacillus	Lactobacillus_saerimneri	OTU719	0	0	0	0
Firmicutes	Clostridia	Clostridiales	Ruminococcaceae	Ruminococcus_1	uncultured_organism_g__Ruminococcus_1	OTU227	0	0	0	0
Deferribacteres	Deferribacteres	Deferribacterales	Deferribacteraceae	Mucispirillum	uncultured_bacterium_g__Mucispirillum	OTU272	0	0	0	0
Chloroflexi	Chloroflexia	Chloroflexales	Roseiflexaceae	Roseiflexus	uncultured_bacterium_g__Roseiflexus	OTU277	0	0	0	0
Actinobacteria	Actinobacteria	Solirubrobacterales	Solirubrobacteraceae	Solirubrobacter	uncultured_bacterium_g__Solirubrobacter	OTU414	0	0	0	0
Firmicutes	Clostridia	Clostridiales	Ruminococcaceae	Anaerotruncus	unclassified_g__Anaerotruncus	OTU546	0	0	1	1
Tenericutes	Mollicutes	Mollicutes_RF9	norank_o__Mollicutes	norank_o__Mollicutes_RF9	uncultured_bacterium_g__norank_o__Mollicutes_RF9	OTU571	0	0	0	0
Firmicutes	Clostridia	Clostridiales	Ruminococcaceae	Ruminiclostridium_5	uncultured_bacterium_g__Ruminiclostridium_5	OTU806	0	1	0	0
Firmicutes	Clostridia	Clostridiales	Ruminococcaceae	Ruminococcaceae_UCG-013	unclassified_g__Ruminococcaceae_UCG-013	OTU108	0	0	2	0
Firmicutes	Clostridia	Clostridiales	Peptococcaceae	norank_f__Peptococcaceae	uncultured_bacterium_g__norank_f__Peptococcaceae	OTU112	0	0	0	0
Acidobacteria	Acidobacteria	Blastocatellales	Blastocatellaceae__Su	RB41	unclassified_g__RB41	OTU374	0	0	0	0
Actinobacteria	Actinobacteria	Propionibacteriales	Nocardioidaceae	Marmoricola	uncultured_bacterium_g__Marmoricola	OTU395	0	0	0	0
Firmicutes	Clostridia	Clostridiales	Ruminococcaceae	unclassified_f__Ruminococcaceae	unclassified_f__Ruminococcaceae	OTU650	0	0	0	0
Firmicutes	Clostridia	Clostridiales	Ruminococcaceae	Ruminiclostridium_9	unclassified_g__Ruminiclostridium_9	OTU92	0	0	0	0
Actinobacteria	Actinobacteria	Streptomycetales	Streptomycetaceae	Streptomyces	unclassified_g__Streptomyces	OTU358	0	0	0	0
Proteobacteria	Deltaproteobacteria	Desulfovibrionales	Desulfovibrionaceae	Desulfovibrio	unclassified_g__Desulfovibrio	OTU431	0	0	0	0
Firmicutes	Clostridia	Clostridiales	Lachnospiraceae	unclassified_f__Lachnospiraceae	unclassified_f__Lachnospiraceae	OTU668	0	0	0	1
Firmicutes	Clostridia	Clostridiales	Syntrophomonadacea	Syntrophomonas	unclassified_g__Syntrophomonas	OTU790	0	0	0	0
Bacteroidetes	Bacteroidia	Bacteroidales	Bacteroidales_S24-7_g	norank_f__Bacteroidales_S24-7_gro	uncultured_bacterium_g__norank_f__Bacteroidales_S24-7_group	OTU369	0	0	0	0
Firmicutes	Clostridia	Clostridiales	Peptostreptococcacea	Peptoclostridium	Peptoclostridium_difficile_g__Peptoclostridium	OTU459	0	0	0	0
Firmicutes	Erysipelotrichia	Erysipelotrichales	Erysipelotrichaceae	[Clostridium]_innocuum_group	uncultured_bacterium_g__[Clostridium]_innocuum_group	OTU503	3	0	1	0
Firmicutes	Clostridia	Clostridiales	Ruminococcaceae	Ruminococcaceae_UCG-009	uncultured_bacterium_g__Ruminococcaceae_UCG-009	OTU686	0	0	0	0
Firmicutes	Clostridia	Clostridiales	Ruminococcaceae	Anaerotruncus	unclassified_g__Anaerotruncus	OTU753	0	0	0	1
Firmicutes	Clostridia	Clostridiales	Ruminococcaceae	Ruminiclostridium	uncultured_organism_g__Ruminiclostridium	OTU829	0	0	0	0
Bacteroidetes	Bacteroidia	Bacteroidales	Bacteroidales_S24-7_g	norank_f__Bacteroidales_S24-7_gro	uncultured_bacterium_g__norank_f__Bacteroidales_S24-7_group	OTU304	0	0	0	0

Bacteroidetes	Bacteroidia	Bacteroidales	Bacteroidales_S24-7_g	norank_f__Bacteroidales_S24-7_gro	uncultured_bacterium_g__norank_f__Bacteroidales_S24-7_group	OTU350	0	0	0	0
Bacteroidetes	Bacteroidia	Bacteroidales	Bacteroidales_S24-7_g	norank_f__Bacteroidales_S24-7_gro	uncultured_bacterium_g__norank_f__Bacteroidales_S24-7_group	OTU380	0	0	0	0
Firmicutes	Clostridia	Clostridiales	Ruminococcaceae	Candidatus_Soleaferrea	Ruminococcaceae_bacterium_GD6	OTU456	0	0	0	0
Firmicutes	Clostridia	Clostridiales	Ruminococcaceae	unclassified_f__Ruminococcaceae	unclassified_f__Ruminococcaceae	OTU545	0	0	0	0
Firmicutes	Clostridia	Clostridiales	Ruminococcaceae	Ruminococcaceae_UCG-009	uncultured_bacterium_g__Ruminococcaceae_UCG-009	OTU595	0	0	0	0
Firmicutes	Clostridia	Clostridiales	Ruminococcaceae	unclassified_f__Ruminococcaceae	unclassified_f__Ruminococcaceae	OTU844	0	0	0	0
Firmicutes	Clostridia	Clostridiales	Christensenellaceae	norank_f__Christensenellaceae	uncultured_bacterium_g__norank_f__Christensenellaceae	OTU126	0	1	0	0
Firmicutes	Clostridia	Clostridiales	Clostridiales_vadinBB6	norank_f__Clostridiales_vadinBB60_	uncultured_bacterium_g__norank_f__Clostridiales_vadinBB60_group	OTU161	0	0	0	0
Bacteroidetes	Bacteroidia	Bacteroidales	Bacteroidales_S24-7_g	norank_f__Bacteroidales_S24-7_gro	unclassified_g__norank_f__Bacteroidales_S24-7_group	OTU267	0	0	0	0
Actinobacteria	Actinobacteria	Streptosporangiales	Thermomonosporacea	Actinomadura	Actinomadura_hallensis	OTU291	0	0	0	0
Firmicutes	Clostridia	Clostridiales	Ruminococcaceae	Ruminiclostridium_5	unclassified_g__Ruminiclostridium_5	OTU341	0	0	0	0
Actinobacteria	Actinobacteria	Corynebacteriales	Mycobacteriaceae	Mycobacterium	unclassified_g__Mycobacterium	OTU381	0	0	0	0
Firmicutes	Clostridia	Clostridiales	Ruminococcaceae	Ruminococcus_1	unclassified_g__Ruminococcus_1	OTU523	0	0	0	0
Bacteroidetes	Bacteroidia	Bacteroidales	Rikenellaceae	Alistipes	Alistipes_sp._CHKCI003	OTU15	0	2	0	0
Firmicutes	Clostridia	Clostridiales	Ruminococcaceae	Ruminococcaceae_UCG-010	unclassified_g__Ruminococcaceae_UCG-010	OTU222	0	0	0	0
Firmicutes	Clostridia	Clostridiales	Clostridiales_vadinBB6	norank_f__Clostridiales_vadinBB60_	uncultured_prokaryote_g__norank_f__Clostridiales_vadinBB60_group	OTU247	0	0	0	0
Bacteroidetes	Bacteroidia	Bacteroidales	Bacteroidales_S24-7_g	norank_f__Bacteroidales_S24-7_gro	uncultured_bacterium_g__norank_f__Bacteroidales_S24-7_group	OTU253	0	0	0	0
Verrucomicrobia	Spartobacteria	Chthoniobacterales	DA101_soil_group	norank_f__DA101_soil_group	uncultured_Prosthecobacter_sp._g__norank_f__DA101_soil_group	OTU279	0	0	0	0
Cyanobacteria	Cyanobacteria	Gastranaerophiales	norank_o__Gastranae	norank_o__Gastranaerophilales	unclassified_g__norank_o__Gastranaerophilales	OTU328	0	0	0	0
Proteobacteria	Epsilonproteobacteria	Campylobacterales	Helicobacteraceae	Helicobacter	Helicobacter_ganmani	OTU446	0	0	0	0
Firmicutes	Clostridia	Clostridiales	Ruminococcaceae	Ruminococcaceae_UCG-007	unclassified_g__Ruminococcaceae_UCG-007	OTU789	0	0	1	0
Proteobacteria	Alphaproteobacteria	Rhizobiales	Xanthobacteraceae	Variibacter	uncultured_forest_soil_bacterium_g__Variibacter	OTU840	0	0	0	0
Firmicutes	Clostridia	Clostridiales	Ruminococcaceae	Ruminiclostridium_5	uncultured_organism_g__Ruminiclostridium_5	OTU24	0	6	2	0
Gemmatimonade	Gemmatimonadetes	Gemmatimonadales	Gemmatimonadaceae	Gemmatimonas	uncultured_soil_bacterium_g__Gemmatimonas	OTU354	0	0	0	0
Actinobacteria	Actinobacteria	Propionibacteriales	Nocardioidaceae	Kribbella	uncultured_bacterium_g__Kribbella	OTU391	0	0	0	0
Actinobacteria	Actinobacteria	Pseudonocardiales	Pseudonocardiaceae	Pseudonocardia	uncultured_bacterium_g__Pseudonocardia	OTU392	0	0	0	0
Firmicutes	Clostridia	Clostridiales	Lachnospiraceae	unclassified_f__Lachnospiraceae	unclassified_f__Lachnospiraceae	OTU462	0	0	0	0
Firmicutes	Clostridia	Clostridiales	Ruminococcaceae	Anaerotruncus	unclassified_g__Anaerotruncus	OTU486	0	0	0	0
Actinobacteria	Actinobacteria	Coriobacteriales	Coriobacteriaceae	Gordonibacter	unclassified_g__Gordonibacter	OTU555	1	0	0	0
Firmicutes	Clostridia	Clostridiales	Ruminococcaceae	Anaerotruncus	unclassified_g__Anaerotruncus	OTU687	0	0	0	0
unclassified_k__r	unclassified_k__norank	unclassified_k__no	unclassified_k__noran	unclassified_k__norank	unclassified_k__norank	OTU706	1	0	0	0
Proteobacteria	Alphaproteobacteria	Rhizobiales	Xanthobacteraceae	Variibacter	uncultured_forest_soil_bacterium_g__Variibacter	OTU252	0	0	0	0
Actinobacteria	Actinobacteria	Micromonosporales	Micromonosporaceae	Longispora	uncultured_bacterium_g__Longispora	OTU357	0	0	0	0
Actinobacteria	Actinobacteria	Frankiales	Acidothermaceae	Acidothermus	Thermomonosporaceae_bacterium_YE4-D4-16-CH2	OTU403	0	0	0	0
Tenericutes	Mollicutes	Mollicutes_RF9	norank_o__Mollicutes	norank_o__Mollicutes_RF9	unclassified_g__norank_o__Mollicutes_RF9	OTU547	0	0	0	0
Bacteroidetes	Bacteroidia	Bacteroidales	Bacteroidaceae	Bacteroides	unclassified_g__Bacteroides	OTU554	0	0	1	0
Firmicutes	Clostridia	Clostridiales	Ruminococcaceae	unclassified_f__Ruminococcaceae	unclassified_f__Ruminococcaceae	OTU708	0	0	0	0
Firmicutes	Clostridia	Clostridiales	Christensenellaceae	unclassified_f__Christensenellaceae	unclassified_f__Christensenellaceae	OTU205	0	0	0	0
Firmicutes	Clostridia	Clostridiales	Ruminococcaceae	Ruminococcaceae_UCG-010	uncultured_bacterium_g__Ruminococcaceae_UCG-010	OTU240	0	0	0	0
Bacteroidetes	Bacteroidia	Bacteroidales	Rikenellaceae	Rikenella	uncultured_bacterium_g__Rikenella	OTU244	0	0	0	0
Bacteroidetes	Bacteroidia	Bacteroidales	Bacteroidales_S24-7_g	norank_f__Bacteroidales_S24-7_gro	uncultured_bacterium_g__norank_f__Bacteroidales_S24-7_group	OTU259	0	0	0	0
Bacteroidetes	Bacteroidia	Bacteroidales	Bacteroidales_S24-7_g	norank_f__Bacteroidales_S24-7_gro	unclassified_g__norank_f__Bacteroidales_S24-7_group	OTU280	0	0	0	0
Actinobacteria	Actinobacteria	Micromonosporales	Micromonosporaceae	unclassified_f__Micromonosporacea	unclassified_f__Micromonosporaceae	OTU286	0	0	0	0
Actinobacteria	Actinobacteria	Gaiellales	Gaiellaceae	Gaiella	uncultured_bacterium_g__Gaiella	OTU309	0	0	0	0
Bacteroidetes	Bacteroidia	Bacteroidales	Bacteroidales_S24-7_g	norank_f__Bacteroidales_S24-7_gro	uncultured_Bacteroidales_bacterium_g__norank_f__Bacteroidales_S24-7_group	OTU348	0	0	0	0
Actinobacteria	Actinobacteria	Propionibacteriales	Nocardioidaceae	Nocardioides	uncultured_bacterium_g__Nocardioides	OTU367	0	0	0	0
Actinobacteria	Actinobacteria	norank_c__Actinob	norank_c__Actinobact	norank_c__Actinobacteria	uncultured_bacterium_g__norank_c__Actinobacteria	OTU396	0	0	0	0
Firmicutes	Clostridia	Clostridiales	Ruminococcaceae	unclassified_f__Ruminococcaceae	unclassified_f__Ruminococcaceae	OTU617	0	0	0	0
Proteobacteria	Alphaproteobacteria	Rhizobiales	Methylobacteriaceae	Meganema	Meganema_perideroedes	OTU30	0	3	2	0
Firmicutes	Clostridia	Clostridiales	Ruminococcaceae	unclassified_f__Ruminococcaceae	unclassified_f__Ruminococcaceae	OTU239	0	0	2	1
Proteobacteria	Betaproteobacteria	Burkholderiales	Alcaligenaceae	Parasutterella	Sutterellaceae_bacterium_YL45	OTU281	0	0	0	0
Bacteroidetes	Bacteroidia	Bacteroidales	Bacteroidales_S24-7_g	norank_f__Bacteroidales_S24-7_gro	unclassified_g__norank_f__Bacteroidales_S24-7_group	OTU303	0	0	0	0
Bacteroidetes	Bacteroidia	Bacteroidales	Bacteroidales_S24-7_g	norank_f__Bacteroidales_S24-7_gro	uncultured_Bacteroidales_bacterium_g__norank_f__Bacteroidales_S24-7_group	OTU307	0	0	0	0
Chloroflexi	Anaerolineae	Anaerolineales	Anaerolineaceae	norank_f__Anaerolineaceae	wastewater_metagenome_g__norank_f__Anaerolineaceae	OTU352	0	0	0	0
Bacteroidetes	Bacteroidia	Bacteroidales	Bacteroidales_S24-7_g	norank_f__Bacteroidales_S24-7_gro	uncultured_bacterium_g__norank_f__Bacteroidales_S24-7_group	OTU389	0	0	0	0
Chloroflexi	Chloroflexia	Chloroflexales	Roseiflexaceae	Roseiflexus	uncultured_bacterium_g__Roseiflexus	OTU404	0	0	0	0
Firmicutes	Clostridia	Clostridiales	Lachnospiraceae	Lachnoclostridium	unclassified_g__Lachnoclostridium	OTU416	0	0	0	0
Chloroflexi	Chloroflexia	Chloroflexales	Roseiflexaceae	Roseiflexus	uncultured_bacterium_g__Roseiflexus	OTU425	0	0	0	0
Actinobacteria	Actinobacteria	Corynebacteriales	Nocardiaceae	Rhodococcus	Rhodococcus_erythropolis	OTU662	0	0	0	0
Proteobacteria	Gammaproteobacteria	Pseudomonadales	Moraxellaceae	Acinetobacter	unclassified_g__Acinetobacter	OTU685	0	1	0	0
Firmicutes	Clostridia	Clostridiales	Clostridiaceae_1	Clostridium_sensu_stricto_1	unclassified_g__Clostridium_sensu_stricto_1	OTU817	0	0	0	0
Firmicutes	Clostridia	Clostridiales	Christensenellaceae	Christensenellaceae_R-7_group	uncultured_Clostridiaceae_bacterium_g__Christensenellaceae_R-7_group	OTU47	0	0	0	0
Firmicutes	Clostridia	Clostridiales	Ruminococcaceae	Anaerotruncus	unclassified_g__Anaerotruncus	OTU96	0	0	0	0
Firmicutes	Clostridia	Clostridiales	Clostridiales_vadinBB6	norank_f__Clostridiales_vadinBB60_	uncultured_prokaryote_g__norank_f__Clostridiales_vadinBB60_group	OTU147	0	0	0	0
Firmicutes	Clostridia	Clostridiales	Ruminococcaceae	Candidatus_Soleaferrea	Ruminococcaceae_bacterium_Marseille-P2935	OTU183	0	0	0	0
Tenericutes	Mollicutes	Mollicutes_RF9	norank_o__Mollicutes	norank_o__Mollicutes_RF9	uncultured_bacterium_g__norank_o__Mollicutes_RF9	OTU208	0	0	0	0
Firmicutes	unclassified_p__Firmicut	unclassified_p__Fir	unclassified_p__Firmi	unclassified_p__Firmicutes	unclassified_p__Firmicutes	OTU246	0	0	0	0
Bacteroidetes	Bacteroidia	Bacteroidales	Bacteroidales_S24-7_g	norank_f__Bacteroidales_S24-7_gro	uncultured_bacterium_g__norank_f__Bacteroidales_S24-7_group	OTU266	0	0	0	0
Bacteroidetes	Bacteroidia	Bacteroidales	Rikenellaceae	Alistipes	uncultured_bacterium_g__Alistipes	OTU282	0	0	0	0
Actinobacteria	Actinobacteria	Solirubrobacterales	Solirubrobacteraceae	Solirubrobacter	uncultured_bacterium_g__Solirubrobacter	OTU288	0	0	0	0
Bacteroidetes	Bacteroidia	Bacteroidales	Bacteroidales_S24-7_g	norank_f__Bacteroidales_S24-7_gro	uncultured_bacterium_g__norank_f__Bacteroidales_S24-7_group	OTU289	0	0	0	0
Bacteroidetes	Bacteroidia	Bacteroidales	Bacteroidaceae	Bacteroides	unclassified_g__Bacteroides	OTU290	0	0	0	0
Chloroflexi	Chloroflexia	Chloroflexales	Roseiflexaceae	Roseiflexus	uncultured_bacterium_g__Roseiflexus	OTU318	0	0	0	0
Acidobacteria	Acidobacteria	norank_c__Acidoba	norank_c__Acidobacte	norank_c__Acidobacteria	uncultured_Acidobacteriales_bacterium_g__norank_c__Acidobacteria	OTU366	0	0	0	0
Proteobacteria	Epsilonproteobacteria	Campylobacterales	Campylobacteraceae	Arcobacter	unclassified_g__Arcobacter	OTU376	0	0	0	0
Gemmatimonade	Gemmatimonadetes	Gemmatimonadales	Gemmatimonadaceae	Gemmatimonas	unclassified_g__Gemmatimonas	OTU377	0	0	0	0
Proteobacteria	Alphaproteobacteria	Rhizobiales	Methylocystaceae	unclassified_f__Methylocystaceae	unclassified_f__Methylocystaceae	OTU379	0	0	0	0
Proteobacteria	Alphaproteobacteria	Rhodobacterales	Rhodobacteraceae	Pannonibacter	unclassified_g__Pannonibacter	OTU421	0	0	0	0
Proteobacteria	Epsilonproteobacteria	Campylobacterales	Helicobacteraceae	Helicobacter	Helicobacter_hepaticus	OTU422	0	0	0	0
Tenericutes	Mollicutes	Mollicutes_RF9	norank_o__Mollicutes	norank_o__Mollicutes_RF9	unclassified_g__norank_o__Mollicutes_RF9	OTU491	0	0	0	0
Firmicutes	Clostridia	Clostridiales	Ruminococcaceae	unclassified_f__Ruminococcaceae	unclassified_f__Ruminococcaceae	OTU696	0	0	0	0
Firmicutes	Clostridia	Clostridiales	Christensenellaceae	norank_f__Christensenellaceae	uncultured_bacterium_g__norank_f__Christensenellaceae	OTU819	0	0	0	0
Firmicutes	Clostridia	Clostridiales	Ruminococcaceae	unclassified_f__Ruminococcaceae	unclassified_f__Ruminococcaceae	OTU186	0	0	0	0
Firmicutes	Clostridia	Clostridiales	Ruminococcaceae	Ruminiclostridium	uncultured_bacterium_g__Ruminiclostridium	OTU269	0	0	0	0
Bacteroidetes	Bacteroidia	Bacteroidales	Porphyromonadaceae	Parabacteroides	uncultured_organism_g__Parabacteroides	OTU285	0	0	0	0
Bacteroidetes	Bacteroidia	Bacteroidales	Rikenellaceae	Alistipes	uncultured_bacterium_g__Alistipes	OTU299	0	0	0	0
Actinobacteria	Actinobacteria	Acidimicrobiales	norank_o__Acidimicro	norank_o__Acidimicrobiales	uncultured_Acidimicrobiales_bacterium_g__norank	OTU305	0	0	0	0
Chloroflexi	S085	norank_c__S085	norank_c__S085	norank_c__S085	uncultured_Chloroflexi_bacterium_g__norank_c__S085	OTU362	0	0	0	0
Proteobacteria	Alphaproteobacteria	Rhodospirillales	Acetobacteraceae	norank_f__Acetobacteraceae	uncultured_bacterium_g__norank_f__Acetobacteraceae	OTU371	0	0	0	0
Chloroflexi	TK10	norank_c__TK10	norank_c__TK10	norank_c__TK10	unclassified_g__norank_c__TK10	OTU382	0	0	0	0
Actinobacteria	Actinobacteria	Micrococcales	Brevibacteriaceae	Brevibacterium	unclassified_g__Brevibacterium	OTU399	0	0	0	0
Bacteroidetes	Bacteroidia	Bacteroidales	Bacteroidales_S24-7_g	norank_f__Bacteroidales_S24-7_gro	uncultured_organism_g__norank_f__Bacteroidales_S24-7_group	OTU401	0	0	0	0
Firmicutes	Erysipelotrichia	Erysipelotrichales	Erysipelotrichaceae	Erysipelatoclostridium	uncultured_bacterium_g__Erysipelatoclostridium	OTU407	0	0	0	0
Proteobacteria	Betaproteobacteria	Nitrosomonadales	Nitrosomonadaceae	norank_f__Nitrosomonadaceae	unclassified_g__norank_f__Nitrosomonadaceae	OTU420	0	0	0	0
Firmicutes	Clostridia	Clostridiales	Ruminococcaceae	Ruminococcaceae_UCG-014	uncultured_Firmicutes_bacterium_g__Ruminococcaceae_UCG-014	OTU798	0	0	0	0
Firmicutes	Clostridia	Clostridiales	Ruminococcaceae	Ruminococcaceae_UCG-010	unclassified_g__Ruminococcaceae_UCG-010	OTU800	0	0	0	0
Tenericutes	Mollicutes	Mycoplasmatales	Mycoplasmataceae	norank_f__Mycoplasmataceae	unclassified_g__norank_f__Mycoplasmataceae	OTU825	0	0	0	0
Firmicutes	Clostridia	Clostridiales	Christensenellaceae	Christensenellaceae_R-7_group	unclassified_g__Christensenellaceae_R-7_group	OTU833	0	0	0	0
Bacteroidetes	Bacteroidia	Bacteroidales	Bacteroidaceae	Bacteroides	Bacteroides_caccae	OTU46	0	1	0	0
Bacteroidetes	Flavobacteriia	Flavobacteriales	Flavobacteriaceae	Cloacibacterium	uncultured_bacterium_g__Cloacibacterium	OTU49	0	0	4	0
Firmicutes	Clostridia	Clostridiales	Ruminococcaceae	Ruminococcaceae_UCG-010	uncultured_bacterium_g__Ruminococcaceae_UCG-010	OTU97	0	0	0	0
Proteobacteria	Gammaproteobacteria	Pseudomonadales	Moraxellaceae	Acinetobacter	Acinetobacter_guillouiae	OTU138	0	0	0	0
Bacteroidetes	Bacteroidia	Bacteroidales	Bacteroidales_S24-7_g	norank_f__Bacteroidales_S24-7_gro	uncultured_bacterium_g__norank_f__Bacteroidales_S24-7_group	OTU211	0	0	0	0
Actinobacteria	Actinobacteria	Acidimicrobiales	Acidimicrobiaceae	CL500-29_marine_group	uncultured_bacterium_g__CL500-29_marine_group	OTU260	0	0	0	0
Acidobacteria	Acidobacteria	Acidobacteriales	Acidobacteriaceae__S	norank_f__Acidobacteriaceae__Subg	uncultured_bacterium_g__norank_f__Acidobacteriaceae__Subgroup_1_	OTU263	0	0	0	0
Proteobacteria	Alphaproteobacteria	Rhodospirillales	Rhodospirillaceae	norank_f__Rhodospirillaceae	unclassified_g__norank_f__Rhodospirillaceae	OTU273	0	0	0	0
Actinobacteria	Actinobacteria	Propionibacteriales	Nocardioidaceae	unclassified_f__Nocardioidaceae	unclassified_f__Nocardioidaceae	OTU298	0	0	0	0
Bacteroidetes	Bacteroidia	Bacteroidales	Bacteroidaceae	Bacteroides	Bacteroides_stercoris_ATCC_43183	OTU317	0	0	0	0
Chloroflexi	Chloroflexia	Chloroflexales	Roseiflexaceae	Roseiflexus	uncultured_bacterium_g__Roseiflexus	OTU372	0	0	0	0
Chloroflexi	Anaerolineae	Anaerolineales	Anaerolineaceae	norank_f__Anaerolineaceae	Chloroflexi_bacterium_OLB13	OTU383	0	0	0	0
Actinobacteria	Actinobacteria	Propionibacteriales	Nocardioidaceae	Nocardioides	unclassified_g__Nocardioides	OTU386	0	0	0	0

Chloroflexi	Thermomicrobia	JG30-KF-CM45	norank_o__JG30-KF-C	norank_o__JG30-KF-CM45	unclassified_g__norank_o__JG30-KF-CM45	OTU408	0	0	0	0
Chloroflexi	Gitt-GS-136	norank_c__Gitt-GS-	norank_c__Gitt-GS-13	norank_c__Gitt-GS-136	uncultured_bacterium_g__norank_c__Gitt-GS-136	OTU413	0	0	0	0
Actinobacteria	Actinobacteria	Acidimicrobiales	Iamiaceae	Iamia	uncultured_bacterium_g__Iamia	OTU417	0	0	0	0
Actinobacteria	Actinobacteria	Frankiales	Geodermatophilaceae	Blastococcus	uncultured_Blastococcus_sp._g__Blastococcus	OTU419	0	0	0	0
Actinobacteria	Actinobacteria	Propionibacteriales	Nocardioidaceae	Nocardioides	uncultured_organism_g__Nocardioides	OTU430	0	0	0	0
Gemmatimonade	Gemmatimonadetes	Gemmatimonadales	Gemmatimonadaceae	norank_f__Gemmatimonadaceae	uncultured_Gemmatimonadetes_bacterium_g__norank_f__Gemmatimonadaceae	OTU442	0	0	0	0
Firmicutes	Clostridia	Clostridiales	Ruminococcaceae	Anaerotruncus	uncultured_bacterium_g__Anaerotruncus	OTU592	0	0	0	0
Firmicutes	Clostridia	Clostridiales	Lachnospiraceae	Coprococcus_1	unclassified_g__Coprococcus_1	OTU734	0	0	0	0
Firmicutes	Clostridia	Clostridiales	Ruminococcaceae	Candidatus_Soleaferrea	Candidatus_Soleaferrea_massiliensis	OTU37	0	3	0	0
Cyanobacteria	Cyanobacteria	norank_c__Cyanoba	norank_c__Cyanobact	norank_c__Cyanobacteria	unclassified_g__norank_c__Cyanobacteria	OTU149	0	1	0	0
Firmicutes	Clostridia	Clostridiales	Ruminococcaceae	Ruminococcaceae_UCG-010	unclassified_g__Ruminococcaceae_UCG-010	OTU157	0	0	0	0
Bacteroidetes	Bacteroidia	Bacteroidales	Bacteroidales_S24-7_g	norank_f__Bacteroidales_S24-7_gro	unclassified_g__norank_f__Bacteroidales_S24-7_group	OTU190	0	0	0	0
Firmicutes	Clostridia	Clostridiales	Ruminococcaceae	Ruminococcaceae_UCG-010	uncultured_bacterium_g__Ruminococcaceae_UCG-010	OTU225	0	0	0	0
Proteobacteria	Deltaproteobacteria	Desulfovibrionales	Desulfovibrionaceae	Desulfovibrio	uncultured_bacterium_g__Desulfovibrio	OTU250	0	0	1	0
Bacteroidetes	Bacteroidia	Bacteroidales	Bacteroidales_S24-7_g	norank_f__Bacteroidales_S24-7_gro	uncultured_bacterium_g__norank_f__Bacteroidales_S24-7_group	OTU265	0	0	0	0
Proteobacteria	Betaproteobacteria	Burkholderiales	Comamonadaceae	unclassified_f__Comamonadaceae	unclassified_f__Comamonadaceae	OTU268	0	0	0	0
Firmicutes	Bacilli	Bacillales	Bacillaceae	unclassified_f__Bacillaceae	unclassified_f__Bacillaceae	OTU274	0	0	0	0
Firmicutes	Bacilli	Bacillales	Bacillaceae	Bacillus	Bacillus_anthracis_g__Bacillus	OTU275	0	0	0	0
Proteobacteria	Alphaproteobacteria	Rickettsiales	Mitochondria	norank_f__Mitochondria	Brassica_nigra__black_mustard_	OTU283	0	0	0	0
Firmicutes	Clostridia	Clostridiales	Ruminococcaceae	Anaerotruncus	Anaerotruncus_sp._G3_2012_	OTU296	0	0	0	0
Verrucomicrobia	Spartobacteria	Chthoniobacterales	DA101_soil_group	norank_f__DA101_soil_group	uncultured_Spartobacteria_bacterium_g__norank	OTU302	0	0	0	0
Actinobacteria	Actinobacteria	Acidimicrobiales	norank_o__Acidimicro	norank_o__Acidimicrobiales	uncultured_bacterium_g__norank_o__Acidimicrobiales	OTU314	0	0	0	0
Chloroflexi	Anaerolineae	Anaerolineales	Anaerolineaceae	norank_f__Anaerolineaceae	unclassified_g__norank_f__Anaerolineaceae	OTU316	0	0	0	0
Actinobacteria	Actinobacteria	Pseudonocardiales	Pseudonocardiaceae	Pseudonocardia	unclassified_g__Pseudonocardia	OTU351	0	0	0	0
Acidobacteria	Acidobacteria	Blastocatellales	Blastocatellaceae__Su	RB41	uncultured_Acidobacteria_bacterium_g__RB41	OTU359	0	0	0	0
Actinobacteria	Actinobacteria	Gaiellales	norank_o__Gaiellales	norank_o__Gaiellales	uncultured_bacterium_g__norank_o__Gaiellales	OTU370	0	0	0	0
Chloroflexi	Chloroflexia	Chloroflexales	Roseiflexaceae	Roseiflexus	uncultured_bacterium_g__Roseiflexus	OTU378	0	0	0	0
Chloroflexi	Chloroflexia	Chloroflexales	Roseiflexaceae	Roseiflexus	uncultured_bacterium_g__Roseiflexus	OTU384	0	0	0	0
Acidobacteria	Acidobacteria	Blastocatellales	Blastocatellaceae__Su	RB41	unclassified_g__RB41	OTU387	0	0	0	0
Acidobacteria	Acidobacteria	Solibacterales	Solibacteraceae__Sub	Bryobacter	uncultured_Acidobacterium_sp._g__Bryobacter	OTU388	0	0	0	0
Nitrospirae	Nitrospira	norank_c__Nitrospi	norank_c__Nitrospira	Nitrospira	unclassified_g__Nitrospira	OTU390	0	0	0	0
Actinobacteria	Actinobacteria	Acidimicrobiales	Acidimicrobiales_Ince	Candidatus_Microthrix	uncultured_bacterium_g__Candidatus_Microthrix	OTU393	0	0	0	0
Chloroflexi	Anaerolineae	Anaerolineales	Anaerolineaceae	norank_f__Anaerolineaceae	unclassified_g__norank_f__Anaerolineaceae	OTU397	0	0	0	0
Firmicutes	Clostridia	Clostridiales	Lachnospiraceae	norank_f__Lachnospiraceae	Lachnospiraceae_bacterium_A2	OTU398	0	0	0	0
Actinobacteria	Actinobacteria	Solirubrobacterales	Gsoil-1167	norank_f__Gsoil-1167	uncultured_bacterium_g__norank_f__Gsoil-1167	OTU406	0	0	0	0
Proteobacteria	Alphaproteobacteria	Rhizobiales	Bradyrhizobiaceae	Bosea	unclassified_g__Bosea	OTU426	0	0	0	0
Chloroflexi	Ktedonobacteria	JG30-KF-AS9	norank_o__JG30-KF-A	norank_o__JG30-KF-AS9	uncultured_bacterium_g__norank_o__JG30-KF-AS9	OTU440	0	0	0	0
Gemmatimonade	Gemmatimonadetes	Gemmatimonadales	Gemmatimonadaceae	Gemmatimonas	unclassified_g__Gemmatimonas	OTU441	0	0	0	0
Firmicutes	Clostridia	Clostridiales	Clostridiales_vadinBB6	norank_f__Clostridiales_vadinBB60_	unclassified_g__norank_f__Clostridiales_vadinBB60_group	OTU479	0	0	0	0
Firmicutes	Clostridia	Clostridiales	Lachnospiraceae	unclassified_f__Lachnospiraceae	unclassified_f__Lachnospiraceae	OTU514	0	0	0	0
Firmicutes	Clostridia	Clostridiales	Ruminococcaceae	unclassified_f__Ruminococcaceae	unclassified_f__Ruminococcaceae	OTU569	0	0	0	0
Firmicutes	Clostridia	Clostridiales	Ruminococcaceae	unclassified_f__Ruminococcaceae	unclassified_f__Ruminococcaceae	OTU760	0	0	0	0
Firmicutes	Clostridia	Clostridiales	Ruminococcaceae	Ruminococcaceae_UCG-005	unclassified_g__Ruminococcaceae_UCG-005	OTU814	0	0	0	0
Firmicutes	Clostridia	Clostridiales	Ruminococcaceae	Anaerotruncus	unclassified_g__Anaerotruncus	OTU44	0	0	0	0
Firmicutes	Clostridia	Clostridiales	Clostridiaceae_1	Clostridium_sensu_stricto_1	human_gut_metagenome_g__Clostridium_sensu_stricto_1	OTU77	0	0	0	0
Firmicutes	Clostridia	Clostridiales	Christensenellaceae	Christensenellaceae_R-7_group	unclassified_g__Christensenellaceae_R-7_group	OTU119	0	0	0	0
Bacteroidetes	Bacteroidia	Bacteroidales	Porphyromonadaceae	Odoribacter	uncultured_bacterium_g__Odoribacter	OTU210	0	0	0	0
Bacteroidetes	Bacteroidia	Bacteroidales	Bacteroidales_S24-7_g	norank_f__Bacteroidales_S24-7_gro	uncultured_bacterium_g__norank_f__Bacteroidales_S24-7_group	OTU217	0	0	0	0
Acidobacteria	Acidobacteria	norank_c__Acidoba	norank_c__Acidobacte	norank_c__Acidobacteria	uncultured_Acidobacteriales_bacterium_g__norank_c__Acidobacteria	OTU256	0	0	0	0
Gemmatimonade	Gemmatimonadetes	Gemmatimonadales	Gemmatimonadaceae	unclassified_f__Gemmatimonadacea	unclassified_f__Gemmatimonadaceae	OTU257	0	0	0	0
Acidobacteria	Acidobacteria	norank_c__Acidoba	norank_c__Acidobacte	norank_c__Acidobacteria	unclassified_g__norank_c__Acidobacteria	OTU264	0	0	0	0
Proteobacteria	Betaproteobacteria	Neisseriales	Neisseriaceae	Neisseria	unclassified_g__Neisseria	OTU271	0	0	0	0
Planctomycetes	OM190	norank_c__OM190	norank_c__OM190	norank_c__OM190	uncultured_bacterium_g__norank_c__OM190	OTU278	0	0	0	0
Actinobacteria	Actinobacteria	Coriobacteriales	Coriobacteriaceae	Parvibacter	uncultured_bacterium_g__Parvibacter	OTU292	0	0	0	0
Acidobacteria	Acidobacteria	norank_c__Acidoba	norank_c__Acidobacte	norank_c__Acidobacteria	uncultured_prokaryote_g__norank_c__Acidobacteria	OTU293	0	0	0	0
Actinobacteria	Actinobacteria	Gaiellales	norank_o__Gaiellales	norank_o__Gaiellales	uncultured_Rubrobacteria_bacterium_g__norank	OTU295	0	0	0	0
Proteobacteria	Gammaproteobacteria	BD7-8_marine_grou	norank_o__BD7-8_ma	norank_o__BD7-8_marine_group	unclassified_g__norank_o__BD7-8_marine_group	OTU297	0	0	0	0
Chloroflexi	Chloroflexia	Chloroflexales	Roseiflexaceae	Roseiflexus	uncultured_bacterium_g__Roseiflexus	OTU301	0	0	0	0
Actinobacteria	Actinobacteria	Propionibacteriales	Nocardioidaceae	unclassified_f__Nocardioidaceae	unclassified_f__Nocardioidaceae	OTU306	0	0	0	0
Saccharibacteria	norank_p__Saccharibact	norank_p__Sacchar	norank_p__Saccharib	norank_p__Saccharibacteria	uncultured_bacterium_g__norank_p__Saccharibacteria	OTU311	0	0	0	0
Acidobacteria	Acidobacteria	norank_c__Acidoba	norank_c__Acidobacte	norank_c__Acidobacteria	uncultured_bacterium_66	OTU315	0	0	0	0
Firmicutes	Bacilli	Bacillales	Bacillaceae	unclassified_f__Bacillaceae	unclassified_f__Bacillaceae	OTU319	0	0	0	0
Firmicutes	Clostridia	Clostridiales	Clostridiales_vadinBB6	norank_f__Clostridiales_vadinBB60_	uncultured_bacterium_g__norank_f__Clostridiales_vadinBB60_group	OTU340	0	0	0	0
Acidobacteria	Acidobacteria	norank_c__Acidoba	norank_c__Acidobacte	norank_c__Acidobacteria	unclassified_g__norank_c__Acidobacteria	OTU349	0	0	0	0
Saccharibacteria	norank_p__Saccharibact	norank_p__Sacchar	norank_p__Saccharib	norank_p__Saccharibacteria	uncultured_bacterium_g__norank_p__Saccharibacteria	OTU353	0	0	0	0
Chloroflexi	Anaerolineae	Anaerolineales	Anaerolineaceae	norank_f__Anaerolineaceae	unclassified_g__norank_f__Anaerolineaceae	OTU356	0	0	0	0
Bacteroidetes	Bacteroidia	Bacteroidales	Porphyromonadaceae	Odoribacter	uncultured_bacterium_g__Odoribacter	OTU363	0	0	0	0
Bacteroidetes	Bacteroidia	Bacteroidales	Bacteroidales_S24-7_g	norank_f__Bacteroidales_S24-7_gro	uncultured_bacterium_g__norank_f__Bacteroidales_S24-7_group	OTU364	0	0	0	0
Bacteroidetes	Cytophagia	Cytophagales	Cytophagaceae	Persicitalea	uncultured_bacterium_g__Persicitalea	OTU373	0	0	0	0
Proteobacteria	Deltaproteobacteria	Myxococcales	BIrii41	norank_f__BIrii41	unclassified_g__norank_f__BIrii41	OTU385	0	0	0	0
Chloroflexi	unclassified_p__Chlorofl	unclassified_p__Ch	unclassified_p__Chlor	unclassified_p__Chloroflexi	unclassified_p__Chloroflexi	OTU394	0	0	0	0
Planctomycetes	Planctomycetacia	Planctomycetales	Planctomycetaceae	norank_f__Planctomycetaceae	unclassified_g__norank_f__Planctomycetaceae	OTU400	0	0	0	0
Nitrospirae	Nitrospira	norank_c__Nitrospi	norank_c__Nitrospira	Nitrospira	unclassified_g__Nitrospira	OTU402	0	0	0	0
Actinobacteria	Actinobacteria	Acidimicrobiales	OM1_clade	norank_f__OM1_clade	unclassified_g__norank_f__OM1_clade	OTU412	0	0	0	0
Firmicutes	Clostridia	Clostridiales	Lachnospiraceae	norank_f__Lachnospiraceae	uncultured_bacterium_g__norank_f__Lachnospiraceae	OTU418	0	0	0	0
Gemmatimonade	Gemmatimonadetes	Gemmatimonadales	Gemmatimonadaceae	Gemmatimonas	uncultured_bacterium_g__Gemmatimonas	OTU424	0	0	0	0
Chloroflexi	Thermomicrobia	JG30-KF-CM45	norank_o__JG30-KF-C	norank_o__JG30-KF-CM45	uncultured_Sphaerobacter_sp._g__norank	OTU428	0	0	0	0
Proteobacteria	Alphaproteobacteria	Rhodospirillales	Acetobacteraceae	norank_f__Acetobacteraceae	uncultured_bacterium_g__norank_f__Acetobacteraceae	OTU434	0	0	0	0
Actinobacteria	Actinobacteria	Coriobacteriales	Coriobacteriaceae	Coriobacteriaceae_UCG-002	uncultured_bacterium_g__Coriobacteriaceae_UCG-002	OTU436	0	0	0	0
Acidobacteria	Acidobacteria	unclassified_c__Aci	unclassified_c__Acido	unclassified_c__Acidobacteria	unclassified_c__Acidobacteria	OTU438	0	0	0	0
Chloroflexi	Ktedonobacteria	C0119	norank_o__C0119	norank_o__C0119	unclassified_g__norank_o__C0119	OTU439	0	0	0	0
unclassified_k__r	unclassified_k__norank	unclassified_k__no	unclassified_k__noran	unclassified_k__norank	unclassified_k__norank	OTU453	0	0	0	0
unclassified_k__r	unclassified_k__norank	unclassified_k__no	unclassified_k__noran	unclassified_k__norank	unclassified_k__norank	OTU620	0	0	0	0
unclassified_k__r	unclassified_k__norank	unclassified_k__no	unclassified_k__noran	unclassified_k__norank	unclassified_k__norank	OTU786	0	0	0	0

Phylum	Class	Order	Family	Genus	Species	OTU	Red1	Red2	Red3	Red4
Firmicutes	Clostridia	Clostridiales	Ruminococcaceae	Faecalibacterium	uncultured_bacterium_g__Faecalibacterium	OTU705	2856	3820	15533	1290
Bacteroidetes	Bacteroidia	Bacteroidales	Rikenellaceae	Alistipes	gut_metagenome_g__Alistipes	OTU287	7740	7418	3745	8250
Firmicutes	Clostridia	Clostridiales	Lachnospiraceae	[Ruminococcus]_torques_group	uncultured_Clostridiales_bacterium_g__[Ruminococcus]_torques_group	OTU361	2199	4570	2552	1939
Proteobacteria	Gammaproteobacteria	Enterobacteriales	Enterobacteriaceae	Escherichia-Shigella	Escherichia_coli_g__Escherichia-Shigella	OTU589	47	1559	1205	245
Firmicutes	Clostridia	Clostridiales	Ruminococcaceae	Faecalibacterium	bacterium_ic1379	OTU50	3414	1930	120	3882
Bacteroidetes	Bacteroidia	Bacteroidales	Rikenellaceae	Alistipes	unclassified_g__Alistipes	OTU501	654	641	333	1815
Firmicutes	Bacilli	Lactobacillales	Lactobacillaceae	Lactobacillus	unclassified_g__Lactobacillus	OTU445	305	26	2024	2473
Firmicutes	Clostridia	Clostridiales	Ruminococcaceae	Subdoligranulum	uncultured_bacterium_g__Subdoligranulum	OTU816	243	209	692	1540
Firmicutes	Clostridia	Clostridiales	Lachnospiraceae	unclassified_f__Lachnospiraceae	unclassified_f__Lachnospiraceae	OTU581	1189	1386	2750	616
Firmicutes	Clostridia	Clostridiales	Lachnospiraceae	Blautia	unclassified_g__Blautia	OTU78	637	135	1591	321
Firmicutes	Clostridia	Clostridiales	Lachnospiraceae	Eisenbergiella	uncultured_bacterium_g__Eisenbergiella	OTU48	1305	1307	735	389
Firmicutes	Clostridia	Clostridiales	Ruminococcaceae	Anaerotruncus	unclassified_g__Anaerotruncus	OTU213	845	870	473	415
Firmicutes	Clostridia	Clostridiales	Lachnospiraceae	unclassified_f__Lachnospiraceae	unclassified_f__Lachnospiraceae	OTU710	123	1365	620	426
Firmicutes	Bacilli	Lactobacillales	Lactobacillaceae	Lactobacillus	Lactobacillus_salivarius	OTU775	372	922	169	208
Firmicutes	Clostridia	Clostridiales	Lachnospiraceae	Shuttleworthia	uncultured_bacterium_g__Shuttleworthia	OTU683	481	175	1047	260
Firmicutes	Clostridia	Clostridiales	Ruminococcaceae	Butyricicoccus	uncultured_bacterium_g__Butyricicoccus	OTU616	202	526	549	969
Firmicutes	Clostridia	Clostridiales	Lachnospiraceae	[Ruminococcus]_torques_group	unclassified_g__[Ruminococcus]_torques_group	OTU196	29	85	38	495
Firmicutes	Clostridia	Clostridiales	Lachnospiraceae	Lachnoclostridium	unclassified_g__Lachnoclostridium	OTU527	482	772	453	470
Firmicutes	Clostridia	Clostridiales	Ruminococcaceae	Ruminiclostridium_9	unclassified_g__Ruminiclostridium_9	OTU174	959	590	926	407
Firmicutes	Clostridia	Clostridiales	Ruminococcaceae	Subdoligranulum	unclassified_g__Subdoligranulum	OTU88	40	392	38	34
Firmicutes	Clostridia	Clostridiales	Ruminococcaceae	Ruminococcaceae_UCG-014	unclassified_g__Ruminococcaceae_UCG-014	OTU712	564	194	730	412
Proteobacteria	Betaproteobacteria	Burkholderiales	Alcaligenaceae	Parasutterella	uncultured_organism_g__Parasutterella	OTU692	247	261	171	445
Firmicutes	Clostridia	Clostridiales	Ruminococcaceae	Faecalibacterium	uncultured_bacterium_g__Faecalibacterium	OTU122	117	3	493	4
Firmicutes	Clostridia	Clostridiales	Clostridiales_vadinBB6	norank_f__Clostridiales_vadinBB60_	uncultured_bacterium_g__norank_f__Clostridiales_vadinBB60_group	OTU151	4271	1226	41	1241
Firmicutes	Clostridia	Clostridiales	Ruminococcaceae	Faecalibacterium	uncultured_bacterium_g__Faecalibacterium	OTU70	252	33	521	496
Firmicutes	Clostridia	Clostridiales	Lachnospiraceae	unclassified_f__Lachnospiraceae	unclassified_f__Lachnospiraceae	OTU711	331	587	1006	299
Firmicutes	Bacilli	Lactobacillales	Lactobacillaceae	Lactobacillus	Lactobacillus_vaginalis	OTU489	244	0	57	166
Firmicutes	Clostridia	Clostridiales	Lachnospiraceae	Sellimonas	unclassified_g__Sellimonas	OTU476	178	179	319	576
Firmicutes	Clostridia	Clostridiales	Lachnospiraceae	unclassified_f__Lachnospiraceae	unclassified_f__Lachnospiraceae	OTU179	704	517	343	455
Firmicutes	Clostridia	Clostridiales	Ruminococcaceae	norank_f__Ruminococcaceae	unclassified_g__norank_f__Ruminococcaceae	OTU191	1	455	177	138
Firmicutes	Erysipelotrichia	Erysipelotrichales	Erysipelotrichaceae	Erysipelatoclostridium	bacterium_ic1391	OTU561	307	322	143	294
Firmicutes	Clostridia	Clostridiales	Ruminococcaceae	[Eubacterium]_coprostanoligenes_g	unclassified_g__[Eubacterium]_coprostanoligenes_group	OTU13	465	356	561	566
Firmicutes	Bacilli	Lactobacillales	Enterococcaceae	Enterococcus	Enterococcus_cecorum	OTU562	12	4280	21	6
Firmicutes	Clostridia	Clostridiales	Lachnospiraceae	unclassified_f__Lachnospiraceae	unclassified_f__Lachnospiraceae	OTU484	237	312	308	221
Firmicutes	Clostridia	Clostridiales	Ruminococcaceae	unclassified_f__Ruminococcaceae	unclassified_f__Ruminococcaceae	OTU478	309	1003	554	296
Firmicutes	Clostridia	Clostridiales	Lachnospiraceae	Lachnoclostridium	Clostridium_sp._BR31	OTU836	374	550	192	319
Firmicutes	Clostridia	Clostridiales	Ruminococcaceae	norank_f__Ruminococcaceae	Clostridiales_bacterium_24-4c	OTU743	515	110	47	0
Firmicutes	Bacilli	Bacillales	Bacillaceae	unclassified_f__Bacillaceae	unclassified_f__Bacillaceae	OTU851	1235	602	29	178
Firmicutes	Clostridia	Clostridiales	Lachnospiraceae	[Ruminococcus]_torques_group	uncultured_Clostridiales_bacterium_g__[Ruminococcus]_torques_group	OTU774	2	482	555	9
Firmicutes	Clostridia	Clostridiales	Lachnospiraceae	[Eubacterium]_hallii_group	uncultured_bacterium_g__[Eubacterium]_hallii_group	OTU202	111	187	131	356
Firmicutes	Clostridia	Clostridiales	Lachnospiraceae	Anaerostipes	Anaerostipes_butyraticus	OTU189	110	116	146	31
Firmicutes	Clostridia	Clostridiales	Ruminococcaceae	Ruminococcaceae_UCG-014	uncultured_bacterium_g__Ruminococcaceae_UCG-014	OTU578	2874	177	198	399
Firmicutes	Clostridia	Clostridiales	Lachnospiraceae	Sellimonas	bacterium_P1C11	OTU218	289	426	249	94
Firmicutes	Clostridia	Clostridiales	Christensenellaceae	Christensenellaceae_R-7_group	unclassified_g__Christensenellaceae_R-7_group	OTU709	0	42	61	1056
Firmicutes	Clostridia	Clostridiales	Ruminococcaceae	Butyricicoccus	unclassified_g__Butyricicoccus	OTU33	11	91	105	474
Bacteroidetes	Bacteroidia	Bacteroidales	Bacteroidaceae	Bacteroides	Bacteroides_acidifaciens	OTU226	0	0	0	0
Firmicutes	Clostridia	Clostridiales	Ruminococcaceae	Ruminococcaceae_UCG-014	unclassified_g__Ruminococcaceae_UCG-014	OTU65	163	194	130	1016
Firmicutes	Clostridia	Clostridiales	Lachnospiraceae	Lachnoclostridium	unclassified_g__Lachnoclostridium	OTU469	197	453	217	205
Firmicutes	Clostridia	Clostridiales	Ruminococcaceae	Ruminococcaceae_UCG-005	uncultured_organism_g__Ruminococcaceae_UCG-005	OTU690	555	86	251	111
Firmicutes	Erysipelotrichia	Erysipelotrichales	Erysipelotrichaceae	Erysipelatoclostridium	[Clostridium]_spiroforme	OTU625	283	90	73	226
Tenericutes	Mollicutes	Mollicutes_RF9	norank_o__Mollicutes	norank_o__Mollicutes_RF9	uncultured_bacterium_g__norank_o__Mollicutes_RF9	OTU588	356	31	10	1804
Firmicutes	Clostridia	Clostridiales	Lachnospiraceae	Tyzzerella	uncultured_organism_g__Tyzzerella	OTU795	662	156	76	157
Firmicutes	Clostridia	Clostridiales	Ruminococcaceae	Ruminiclostridium	uncultured_bacterium_g__Ruminiclostridium	OTU368	166	571	1052	123
Firmicutes	Clostridia	Clostridiales	Ruminococcaceae	Butyricicoccus	Butyricicoccus_pullicaecorum_1.2	OTU769	18	111	73	18
Firmicutes	Clostridia	Clostridiales	Ruminococcaceae	Ruminococcaceae_UCG-014	uncultured_bacterium_g__Ruminococcaceae_UCG-014	OTU346	356	218	235	364
Firmicutes	Clostridia	Clostridiales	Lachnospiraceae	norank_f__Lachnospiraceae	Clostridiales_bacterium_CHKCI001	OTU738	251	310	256	283
Proteobacteria	Gammaproteobacteria	Pseudomonadales	Pseudomonadaceae	Pseudomonas	Pseudomonas_brenneri	OTU580	333	273	270	63
Firmicutes	Clostridia	Clostridiales	Ruminococcaceae	norank_f__Ruminococcaceae	unclassified_g__norank_f__Ruminococcaceae	OTU460	242	421	51	114
Firmicutes	Clostridia	Clostridiales	Ruminococcaceae	Ruminococcaceae_UCG-014	unidentified_rumen_bacterium_JW32	OTU565	581	1348	813	303
Firmicutes	Clostridia	Clostridiales	Ruminococcaceae	Ruminococcaceae_UCG-014	uncultured_rumen_bacterium_g__Ruminococcaceae_UCG-014	OTU813	4	0	7	1
Firmicutes	Clostridia	Clostridiales	Lachnospiraceae	Lachnospiraceae_NK4A136_group	unclassified_g__Lachnospiraceae_NK4A136_group	OTU193	138	8	94	34
Firmicutes	Clostridia	Clostridiales	Clostridiales_vadinBB6	norank_f__Clostridiales_vadinBB60_	uncultured_bacterium_g__norank_f__Clostridiales_vadinBB60_group	OTU573	257	395	163	90
Firmicutes	Clostridia	Clostridiales	Lachnospiraceae	Coprococcus_1	uncultured_organism_g__Coprococcus_1	OTU591	315	343	164	88
Firmicutes	Clostridia	Clostridiales	Lachnospiraceae	unclassified_f__Lachnospiraceae	unclassified_f__Lachnospiraceae	OTU515	35	43	420	108
Firmicutes	Clostridia	Clostridiales	Ruminococcaceae	Ruminococcaceae_UCG-014	uncultured_organism_g__Ruminococcaceae_UCG-014	OTU457	51	0	79	418
Firmicutes	Clostridia	Clostridiales	Lachnospiraceae	Blautia	Blautia_hydrogenotrophica	OTU718	109	73	77	121
Firmicutes	Clostridia	Clostridiales	Ruminococcaceae	norank_f__Ruminococcaceae	unclassified_g__norank_f__Ruminococcaceae	OTU735	84	153	18	56
Firmicutes	Clostridia	Clostridiales	Peptostreptococcacea	Romboutsia	uncultured_bacterium_g__Romboutsia	OTU79	68	60	34	3
Firmicutes	Clostridia	Clostridiales	Ruminococcaceae	Anaerotruncus	uncultured_bacterium_g__Anaerotruncus	OTU499	217	167	215	121
Firmicutes	Clostridia	Clostridiales	Ruminococcaceae	Ruminococcaceae_UCG-014	uncultured_bacterium_g__Ruminococcaceae_UCG-014	OTU7	502	89	122	60
Firmicutes	Clostridia	Clostridiales	Ruminococcaceae	Ruminococcaceae_UCG-014	unclassified_g__Ruminococcaceae_UCG-014	OTU25	714	85	98	240
Firmicutes	Clostridia	Clostridiales	Ruminococcaceae	[Eubacterium]_coprostanoligenes_g	gut_metagenome_g__[Eubacterium]_coprostanoligenes_group	OTU214	155	0	7	1
Firmicutes	Clostridia	Clostridiales	Ruminococcaceae	Ruminococcaceae_UCG-005	unclassified_g__Ruminococcaceae_UCG-005	OTU160	220	234	61	24
Firmicutes	Clostridia	Clostridiales	Ruminococcaceae	Flavonifractor	uncultured_bacterium_g__Flavonifractor	OTU716	382	93	106	99
Firmicutes	Clostridia	Clostridiales	Ruminococcaceae	Ruminococcaceae_UCG-014	uncultured_bacterium_g__Ruminococcaceae_UCG-014	OTU691	47	26	250	75
Firmicutes	Clostridia	Clostridiales	Ruminococcaceae	Ruminococcaceae_UCG-005	uncultured_prokaryote_g__Ruminococcaceae_UCG-005	OTU504	815	265	255	1
Firmicutes	Clostridia	Clostridiales	Ruminococcaceae	unclassified_f__Ruminococcaceae	unclassified_f__Ruminococcaceae	OTU496	366	83	141	252
Firmicutes	Clostridia	Clostridiales	Ruminococcaceae	Butyricicoccus	unclassified_g__Butyricicoccus	OTU480	14	18	4	131
Firmicutes	Clostridia	Clostridiales	Lachnospiraceae	unclassified_f__Lachnospiraceae	unclassified_f__Lachnospiraceae	OTU533	79	106	234	242
Firmicutes	Bacilli	Lactobacillales	Streptococcaceae	Streptococcus	Streptococcus_gallolyticus_subsp._macedonicus	OTU474	30	23	0	0
Firmicutes	Clostridia	Clostridiales	Ruminococcaceae	norank_f__Ruminococcaceae	Ruminococcaceae_bacterium_AM2	OTU242	93	51	195	1
Firmicutes	Clostridia	Clostridiales	Clostridiales_vadinBB6	norank_f__Clostridiales_vadinBB60_	uncultured_organism_g__norank_f__Clostridiales_vadinBB60_group	OTU150	504	3032	0	0
Firmicutes	Clostridia	Clostridiales	Lachnospiraceae	Shuttleworthia	uncultured_bacterium_g__Shuttleworthia	OTU507	133	111	179	136
Firmicutes	Clostridia	Clostridiales	Lachnospiraceae	Blautia	uncultured_bacterium_g__Blautia	OTU450	58	10	68	14
Firmicutes	Clostridia	Clostridiales	Ruminococcaceae	Ruminococcaceae_UCG-014	uncultured_rumen_bacterium_g__Ruminococcaceae_UCG-014	OTU230	34	0	18	0
Firmicutes	Clostridia	Clostridiales	Ruminococcaceae	Ruminococcaceae_UCG-004	unclassified_g__Ruminococcaceae_UCG-004	OTU355	167	336	68	101
Firmicutes	Clostridia	Clostridiales	Ruminococcaceae	norank_f__Ruminococcaceae	human_gut_metagenome_g__norank_f__Ruminococcaceae	OTU623	328	229	79	103
Firmicutes	Clostridia	Clostridiales	Lachnospiraceae	[Ruminococcus]_gauvreauii_group	bacterium_ic1296	OTU564	50	80	78	76
Firmicutes	Clostridia	Clostridiales	Ruminococcaceae	Anaerofilum	uncultured_bacterium_g__Anaerofilum	OTU123	21	441	155	7
Firmicutes	Clostridia	Clostridiales	Lachnospiraceae	unclassified_f__Lachnospiraceae	unclassified_f__Lachnospiraceae	OTU834	58	130	143	139
Firmicutes	Clostridia	Clostridiales	Clostridiales_vadinBB6	norank_f__Clostridiales_vadinBB60_	uncultured_bacterium_g__norank_f__Clostridiales_vadinBB60_group	OTU512	929	7	13	71
Firmicutes	Erysipelotrichia	Erysipelotrichales	Erysipelotrichaceae	Erysipelatoclostridium	Candidatus_Stoquefichus_sp._SN16	OTU707	0	121	1	0
Firmicutes	Clostridia	Clostridiales	Lachnospiraceae	[Eubacterium]_hallii_group	uncultured_bacterium_g__[Eubacterium]_hallii_group	OTU848	25	21	317	11
Firmicutes	Clostridia	Clostridiales	Ruminococcaceae	Ruminiclostridium_5	uncultured_bacterium_g__Ruminiclostridium_5	OTU856	354	76	97	39
Firmicutes	Clostridia	Clostridiales	Christensenellaceae	Christensenellaceae_R-7_group	uncultured_bacterium_g__Christensenellaceae_R-7_group	OTU171	13	236	55	121
Firmicutes	Clostridia	Clostridiales	Lachnospiraceae	Blautia	unclassified_g__Blautia	OTU767	24	389	0	346
Firmicutes	Clostridia	Clostridiales	Lachnospiraceae	unclassified_f__Lachnospiraceae	unclassified_f__Lachnospiraceae	OTU828	171	16	43	58
Firmicutes	Clostridia	Clostridiales	Ruminococcaceae	Ruminococcaceae_UCG-005	uncultured_Clostridiales_bacterium_g__Ruminococcaceae_UCG-005	OTU815	21	40	46	96
Firmicutes	Clostridia	Clostridiales	Ruminococcaceae	unclassified_f__Ruminococcaceae	unclassified_f__Ruminococcaceae	OTU845	9	18	48	71
Firmicutes	Clostridia	Clostridiales	Lachnospiraceae	unclassified_f__Lachnospiraceae	unclassified_f__Lachnospiraceae	OTU770	109	233	23	47
Firmicutes	Clostridia	Clostridiales	Ruminococcaceae	Anaerotruncus	uncultured_bacterium_g__Anaerotruncus	OTU838	22	100	172	26
Firmicutes	Clostridia	Clostridiales	Lachnospiraceae	unclassified_f__Lachnospiraceae	unclassified_f__Lachnospiraceae	OTU82	79	334	38	94
Firmicutes	Clostridia	Clostridiales	Ruminococcaceae	Anaerotruncus	Anaerotruncus_colihominis_DSM_17241	OTU125	80	8	10	107
Firmicutes	Clostridia	Clostridiales	Clostridiales_vadinBB6	norank_f__Clostridiales_vadinBB60_	uncultured_Clostridia_bacterium_g__norank_f__Clostridiales_vadinBB60_group	OTU697	2	13	64	104
Proteobacteria	Gammaproteobacteria	Pseudomonadales	Pseudomonadaceae	Pseudomonas	unclassified_g__Pseudomonas	OTU58	132	128	131	28
Firmicutes	Clostridia	Clostridiales	Christensenellaceae	Christensenellaceae_R-7_group	unclassified_g__Christensenellaceae_R-7_group	OTU830	104	194	39	244
Firmicutes	Clostridia	Clostridiales	Ruminococcaceae	Subdoligranulum	uncultured_Subdoligranulum_sp.	OTU519	123	15	38	2
Firmicutes	Clostridia	Clostridiales	Lachnospiraceae	Marvinbryantia	unclassified_g__Marvinbryantia	OTU177	378	57	65	18
Firmicutes	Clostridia	Clostridiales	Clostridiales_vadinBB6	norank_f__Clostridiales_vadinBB60_	unclassified_g__norank_f__Clostridiales_vadinBB60_group	OTU107	166	56	21	31
Firmicutes	Clostridia	Clostridiales	Lachnospiraceae	unclassified_f__Lachnospiraceae	unclassified_f__Lachnospiraceae	OTU216	37	44	81	108

Firmicutes	Clostridia	Clostridiales	Ruminococcaceae	Ruminococcaceae_NK4A214_group	uncultured_organism_g__Ruminococcaceae_NK4A214_group	OTU703	38	127	37	144
Firmicutes	Clostridia	Clostridiales	Lachnospiraceae	unclassified_f__Lachnospiraceae	unclassified_f__Lachnospiraceae	OTU540	16	20	37	41
Firmicutes	Clostridia	Clostridiales	Defluviitaleaceae	Defluviitaleaceae_UCG-011	uncultured_bacterium_g__Defluviitaleaceae_UCG-011	OTU725	34	69	146	197
Firmicutes	Clostridia	Clostridiales	Ruminococcaceae	Ruminococcaceae_UCG-010	uncultured_bacterium_g__Ruminococcaceae_UCG-010	OTU799	221	95	95	161
Firmicutes	Clostridia	Clostridiales	Ruminococcaceae	Ruminococcaceae_UCG-014	unclassified_g__Ruminococcaceae_UCG-014	OTU163	194	9	17	125
Firmicutes	Clostridia	Clostridiales	Clostridiales_vadinBB6	norank_f__Clostridiales_vadinBB60_	unclassified_g__norank_f__Clostridiales_vadinBB60_group	OTU57	141	287	82	40
Tenericutes	Mollicutes	Mollicutes_RF9	norank_o__Mollicutes	norank_o__Mollicutes_RF9	unclassified_g__norank_o__Mollicutes_RF9	OTU294	3	46	40	208
Firmicutes	Clostridia	Clostridiales	Ruminococcaceae	Hydrogenoanaerobacterium	uncultured_bacterium_g__Hydrogenoanaerobacterium	OTU613	170	125	136	107
Firmicutes	Clostridia	Clostridiales	Ruminococcaceae	Ruminococcaceae_UCG-014	unclassified_g__Ruminococcaceae_UCG-014	OTU405	15	73	69	192
Firmicutes	Clostridia	Clostridiales	Ruminococcaceae	Ruminococcaceae_UCG-014	uncultured_bacterium_g__Ruminococcaceae_UCG-014	OTU570	107	70	91	202
Firmicutes	Clostridia	Clostridiales	Ruminococcaceae	norank_f__Ruminococcaceae	unclassified_g__norank_f__Ruminococcaceae	OTU313	8	32	51	260
Firmicutes	Bacilli	Lactobacillales	Lactobacillaceae	Lactobacillus	Lactobacillus_aviarius	OTU599	0	0	0	0
Firmicutes	Clostridia	Clostridiales	Lachnospiraceae	unclassified_f__Lachnospiraceae	unclassified_f__Lachnospiraceae	OTU717	16	105	86	58
Firmicutes	Clostridia	Clostridiales	Lachnospiraceae	Eisenbergiella	unclassified_g__Eisenbergiella	OTU443	7	81	20	44
Firmicutes	Clostridia	Clostridiales	Ruminococcaceae	norank_f__Ruminococcaceae	Ruminococcus_sp._16442	OTU113	42	56	26	33
Firmicutes	Clostridia	Clostridiales	Ruminococcaceae	Ruminococcus_1	unclassified_g__Ruminococcus_1	OTU140	31	544	57	13
Firmicutes	Clostridia	Clostridiales	Lachnospiraceae	Lachnospira	unclassified_g__Lachnospira	OTU170	27	30	123	65
Tenericutes	Mollicutes	Mollicutes_RF9	norank_o__Mollicutes	norank_o__Mollicutes_RF9	uncultured_bacterium_g__norank_o__Mollicutes_RF9	OTU823	348	94	41	142
Firmicutes	Clostridia	Clostridiales	Ruminococcaceae	unclassified_f__Ruminococcaceae	unclassified_f__Ruminococcaceae	OTU835	3	18	47	7
Firmicutes	Erysipelotrichia	Erysipelotrichales	Erysipelotrichaceae	norank_f__Erysipelotrichaceae	Clostridiales_bacterium_10-3b	OTU821	75	36	8	48
Firmicutes	Clostridia	Clostridiales	Peptococcaceae	norank_f__Peptococcaceae	uncultured_bacterium_g__norank_f__Peptococcaceae	OTU451	77	40	132	20
Firmicutes	Clostridia	Clostridiales	Lachnospiraceae	unclassified_f__Lachnospiraceae	unclassified_f__Lachnospiraceae	OTU135	0	0	4	0
Firmicutes	Clostridia	Clostridiales	Lachnospiraceae	unclassified_f__Lachnospiraceae	unclassified_f__Lachnospiraceae	OTU550	41	51	99	183
Firmicutes	Clostridia	Clostridiales	Ruminococcaceae	Anaerotruncus	uncultured_bacterium_g__Anaerotruncus	OTU556	19	4	106	2
Firmicutes	Erysipelotrichia	Erysipelotrichales	Erysipelotrichaceae	norank_f__Erysipelotrichaceae	Clostridiales_bacterium_60-7e	OTU583	123	2	59	22
Firmicutes	Clostridia	Clostridiales	Ruminococcaceae	Ruminococcaceae_UCG-014	unclassified_g__Ruminococcaceae_UCG-014	OTU200	72	6	28	98
Bacteroidetes	Bacteroidia	Bacteroidales	Rikenellaceae	Alistipes	gut_metagenome_g__Alistipes	OTU197	11	17	17	154
Tenericutes	Mollicutes	Mollicutes_RF9	norank_o__Mollicutes	norank_o__Mollicutes_RF9	gut_metagenome_g__norank_o__Mollicutes_RF9	OTU156	156	613	28	82
Firmicutes	Clostridia	Clostridiales	Ruminococcaceae	unclassified_f__Ruminococcaceae	unclassified_f__Ruminococcaceae	OTU66	0	4	46	27
Firmicutes	Erysipelotrichia	Erysipelotrichales	Erysipelotrichaceae	Faecalitalea	unclassified_g__Faecalitalea	OTU784	17	50	17	5
Firmicutes	Bacilli	Lactobacillales	Lactobacillaceae	Lactobacillus	Lactobacillus_johnsonii	OTU601	21	14	69	77
Bacteroidetes	Bacteroidia	Bacteroidales	Rikenellaceae	Alistipes	unclassified_g__Alistipes	OTU577	8	18	14	156
Firmicutes	Clostridia	Clostridiales	Ruminococcaceae	Subdoligranulum	unclassified_g__Subdoligranulum	OTU83	3	4	29	45
Proteobacteria	Deltaproteobacteria	Desulfovibrionales	Desulfovibrionaceae	Desulfovibrio	unclassified_g__Desulfovibrio	OTU636	156	187	65	252
Firmicutes	Clostridia	Clostridiales	Clostridiales_vadinBB6	norank_f__Clostridiales_vadinBB60_	unclassified_g__norank_f__Clostridiales_vadinBB60_group	OTU102	523	145	236	107
Firmicutes	Erysipelotrichia	Erysipelotrichales	Erysipelotrichaceae	Erysipelatoclostridium	unclassified_g__Erysipelatoclostridium	OTU8	33	58	41	45
Firmicutes	Clostridia	Clostridiales	Lachnospiraceae	[Ruminococcus]_torques_group	unclassified_g__[Ruminococcus]_torques_group	OTU531	8	8	11	57
Firmicutes	Clostridia	Clostridiales	Ruminococcaceae	Ruminococcaceae_UCG-014	unclassified_g__Ruminococcaceae_UCG-014	OTU614	0	0	0	0
Actinobacteria	Actinobacteria	Coriobacteriales	Coriobacteriaceae	Senegalimassilia	Coriobacteriaceae_bacterium_CHKCI002	OTU206	46	43	13	36
Firmicutes	Clostridia	Clostridiales	Ruminococcaceae	Ruminococcaceae_UCG-014	uncultured_bacterium_g__Ruminococcaceae_UCG-014	OTU842	98	0	42	132
Firmicutes	Clostridia	Clostridiales	Ruminococcaceae	Anaerotruncus	uncultured_organism_g__Anaerotruncus	OTU568	15	78	72	0
Firmicutes	Clostridia	Clostridiales	Ruminococcaceae	norank_f__Ruminococcaceae	unclassified_g__norank_f__Ruminococcaceae	OTU141	16	376	155	126
Firmicutes	Clostridia	Clostridiales	Clostridiales_vadinBB6	norank_f__Clostridiales_vadinBB60_	uncultured_bacterium_g__norank_f__Clostridiales_vadinBB60_group	OTU109	103	98	3	0
Cyanobacteria	Cyanobacteria	Gastranaerophilales	norank_o__Gastranae	norank_o__Gastranaerophilales	uncultured_rumen_bacterium_g__norank_o__Gastranaerophilales	OTU142	1	641	263	0
Firmicutes	Clostridia	Clostridiales	Lachnospiraceae	unclassified_f__Lachnospiraceae	unclassified_f__Lachnospiraceae	OTU699	276	119	86	39
Firmicutes	Clostridia	Clostridiales	Ruminococcaceae	Ruminococcaceae_UCG-010	gut_metagenome_g__Ruminococcaceae_UCG-010	OTU827	45	75	6	77
Firmicutes	Clostridia	Clostridiales	Lachnospiraceae	unclassified_f__Lachnospiraceae	unclassified_f__Lachnospiraceae	OTU612	29	18	43	90
Firmicutes	Clostridia	Clostridiales	Defluviitaleaceae	Defluviitaleaceae_UCG-011	uncultured_bacterium_g__Defluviitaleaceae_UCG-011	OTU567	36	68	55	57
Firmicutes	Clostridia	Clostridiales	Lachnospiraceae	Blautia	uncultured_bacterium_g__Blautia	OTU766	12	23	21	11
Firmicutes	Clostridia	Clostridiales	Christensenellaceae	Christensenellaceae_R-7_group	unclassified_g__Christensenellaceae_R-7_group	OTU148	117	182	41	112
Tenericutes	Mollicutes	Mollicutes_RF9	norank_o__Mollicutes	norank_o__Mollicutes_RF9	uncultured_bacterium_g__norank_o__Mollicutes_RF9	OTU559	267	30	29	157
Firmicutes	Clostridia	Clostridiales	Ruminococcaceae	Anaerotruncus	unclassified_g__Anaerotruncus	OTU657	22	88	37	41
Firmicutes	Clostridia	Clostridiales	Clostridiales_vadinBB6	norank_f__Clostridiales_vadinBB60_	uncultured_bacterium_g__norank_f__Clostridiales_vadinBB60_group	OTU22	23	8	41	16
Firmicutes	Clostridia	Clostridiales	Ruminococcaceae	Anaerotruncus	uncultured_Clostridiaceae_bacterium_g__Anaerotruncus	OTU618	11	0	55	144
Firmicutes	Clostridia	Clostridiales	Ruminococcaceae	Flavonifractor	uncultured_bacterium_g__Flavonifractor	OTU511	11	2	134	45
Firmicutes	Clostridia	Clostridiales	Clostridiales_vadinBB6	norank_f__Clostridiales_vadinBB60_	uncultured_bacterium_g__norank_f__Clostridiales_vadinBB60_group	OTU761	4	38	17	9
Firmicutes	Clostridia	Clostridiales	Clostridiales_vadinBB6	norank_f__Clostridiales_vadinBB60_	unclassified_g__norank_f__Clostridiales_vadinBB60_group	OTU231	99	84	51	112
Firmicutes	Clostridia	Clostridiales	Lachnospiraceae	unclassified_f__Lachnospiraceae	unclassified_f__Lachnospiraceae	OTU73	44	26	18	16
Firmicutes	Erysipelotrichia	Erysipelotrichales	Erysipelotrichaceae	Erysipelatoclostridium	Clostridiales_bacterium_CHKCI006	OTU700	122	46	16	18
Firmicutes	Clostridia	Clostridiales	Lachnospiraceae	unclassified_f__Lachnospiraceae	unclassified_f__Lachnospiraceae	OTU742	236	49	126	31
Firmicutes	Clostridia	Clostridiales	Ruminococcaceae	Anaerotruncus	uncultured_bacterium_g__Anaerotruncus	OTU146	2	78	16	4
Firmicutes	Clostridia	Clostridiales	Ruminococcaceae	Ruminococcaceae_UCG-010	uncultured_bacterium_g__Ruminococcaceae_UCG-010	OTU741	19	95	76	47
Firmicutes	Clostridia	Clostridiales	Ruminococcaceae	Subdoligranulum	unclassified_g__Subdoligranulum	OTU62	459	0	75	0
Firmicutes	Clostridia	Clostridiales	Lachnospiraceae	Blautia	uncultured_bacterium_g__Blautia	OTU466	10	81	52	32
Firmicutes	Clostridia	Clostridiales	Christensenellaceae	Christensenellaceae_R-7_group	uncultured_bacterium_g__Christensenellaceae_R-7_group	OTU746	40	65	11	105
Tenericutes	Mollicutes	Mollicutes_RF9	norank_o__Mollicutes	norank_o__Mollicutes_RF9	uncultured_bacterium_g__norank_o__Mollicutes_RF9	OTU192	2	51	1	67
Firmicutes	Clostridia	Clostridiales	Clostridiales_vadinBB6	norank_f__Clostridiales_vadinBB60_	uncultured_organism_g__norank_f__Clostridiales_vadinBB60_group	OTU103	326	174	0	181
Firmicutes	Clostridia	Clostridiales	Lachnospiraceae	unclassified_f__Lachnospiraceae	unclassified_f__Lachnospiraceae	OTU473	0	8	25	6
Firmicutes	Clostridia	Clostridiales	Ruminococcaceae	Ruminiclostridium_9	uncultured_bacterium_g__Ruminiclostridium_9	OTU110	275	8	4	6
Firmicutes	Bacilli	Lactobacillales	Lactobacillaceae	Lactobacillus	Lactobacillus_agilis	OTU607	0	1	1	3
Firmicutes	Clostridia	Clostridiales	Lachnospiraceae	Marvinbryantia	uncultured_organism_g__Marvinbryantia	OTU437	4	3	9	27
Firmicutes	Clostridia	Clostridiales	Ruminococcaceae	Ruminococcaceae_UCG-014	unclassified_g__Ruminococcaceae_UCG-014	OTU338	9	8	35	4
Firmicutes	Clostridia	Clostridiales	Clostridiales_vadinBB6	norank_f__Clostridiales_vadinBB60_	uncultured_rumen_bacterium_g__norank_f__Clostridiales_vadinBB60_group	OTU330	236	14	31	54
Tenericutes	Mollicutes	Mollicutes_RF9	norank_o__Mollicutes	norank_o__Mollicutes_RF9	uncultured_bacterium_g__norank_o__Mollicutes_RF9	OTU28	22	62	0	150
Firmicutes	Clostridia	Clostridiales	Lachnospiraceae	Coprococcus_1	unclassified_g__Coprococcus_1	OTU539	352	2	3	2
Firmicutes	Clostridia	Clostridiales	Lachnospiraceae	unclassified_f__Lachnospiraceae	unclassified_f__Lachnospiraceae	OTU642	103	79	14	9
Firmicutes	Clostridia	Clostridiales	Ruminococcaceae	Ruminococcaceae_UCG-014	unclassified_g__Ruminococcaceae_UCG-014	OTU590	114	7	54	63
Firmicutes	Clostridia	Clostridiales	Ruminococcaceae	norank_f__Ruminococcaceae	unclassified_g__norank_f__Ruminococcaceae	OTU701	0	26	28	3
Firmicutes	Clostridia	Clostridiales	Ruminococcaceae	Anaerotruncus	uncultured_bacterium_g__Anaerotruncus	OTU249	66	2	86	11
Firmicutes	Clostridia	Clostridiales	Ruminococcaceae	Anaerotruncus	uncultured_bacterium_g__Anaerotruncus	OTU248	33	11	54	5
Firmicutes	Clostridia	Clostridiales	Ruminococcaceae	Ruminiclostridium_5	unclassified_g__Ruminiclostridium_5	OTU826	2	14	48	30
Firmicutes	Clostridia	Clostridiales	Family_XIII	[Eubacterium]_nodatum_group	Eubacteriaceae_bacterium_Marseille-P2843	OTU585	25	33	46	25
Firmicutes	Clostridia	Clostridiales	Christensenellaceae	Christensenellaceae_R-7_group	uncultured_bacterium_g__Christensenellaceae_R-7_group	OTU167	103	45	30	10
Firmicutes	Clostridia	Clostridiales	Lachnospiraceae	norank_f__Lachnospiraceae	Clostridiales_bacterium_CHKCI001	OTU520	25	32	41	63
Firmicutes	Clostridia	Clostridiales	Ruminococcaceae	unclassified_f__Ruminococcaceae	unclassified_f__Ruminococcaceae	OTU238	71	100	49	28
Firmicutes	Clostridia	Clostridiales	Ruminococcaceae	unclassified_f__Ruminococcaceae	unclassified_f__Ruminococcaceae	OTU133	15	17	24	13
Tenericutes	Mollicutes	Mollicutes_RF9	norank_o__Mollicutes	norank_o__Mollicutes_RF9	uncultured_bacterium_g__norank_o__Mollicutes_RF9	OTU606	33	18	31	49
Firmicutes	Clostridia	Clostridiales	Ruminococcaceae	Ruminococcaceae_UCG-009	uncultured_bacterium_g__Ruminococcaceae_UCG-009	OTU779	9	13	54	7
Firmicutes	Clostridia	Clostridiales	Ruminococcaceae	Ruminococcaceae_NK4A214_group	uncultured_organism_g__Ruminococcaceae_NK4A214_group	OTU410	374	10	42	34
Verrucomicrobia	Verrucomicrobiae	Verrucomicrobiales	Verrucomicrobiaceae	Akkermansia	unclassified_g__Akkermansia	OTU344	406	0	0	0
Firmicutes	Clostridia	Clostridiales	Clostridiales_vadinBB6	norank_f__Clostridiales_vadinBB60_	uncultured_bacterium_g__norank_f__Clostridiales_vadinBB60_group	OTU1	253	29	118	20
Firmicutes	Clostridia	Clostridiales	Ruminococcaceae	Oscillospira	uncultured_bacterium_g__Oscillospira	OTU158	15	7	63	5
Firmicutes	Clostridia	Clostridiales	Lachnospiraceae	Lachnospiraceae_NK4A136_group	unclassified_g__Lachnospiraceae_NK4A136_group	OTU187	276	2	4	5
Firmicutes	Clostridia	Clostridiales	Lachnospiraceae	unclassified_f__Lachnospiraceae	unclassified_f__Lachnospiraceae	OTU463	103	3	37	55
Firmicutes	Clostridia	Clostridiales	Peptostreptococcacea	unclassified_f__Peptostreptococca	unclassified_f__Peptostreptococcaceae	OTU526	1	23	1	2
Firmicutes	Bacilli	Lactobacillales	Lactobacillaceae	Lactobacillus	unclassified_g__Lactobacillus	OTU448	1	0	1	28
Firmicutes	Clostridia	Clostridiales	Ruminococcaceae	Ruminococcaceae_NK4A214_group	uncultured_rumen_bacterium_g__Ruminococcaceae_NK4A214_group	OTU181	3	0	36	3
Firmicutes	Clostridia	Clostridiales	Ruminococcaceae	Ruminococcaceae_UCG-014	unclassified_g__Ruminococcaceae_UCG-014	OTU822	28	0	25	44
Firmicutes	Clostridia	Clostridiales	Ruminococcaceae	Ruminococcaceae_UCG-013	unclassified_g__Ruminococcaceae_UCG-013	OTU458	0	0	0	5
Firmicutes	Clostridia	Clostridiales	Clostridiales_vadinBB6	norank_f__Clostridiales_vadinBB60_	unclassified_g__norank_f__Clostridiales_vadinBB60_group	OTU198	26	0	46	187
Firmicutes	Clostridia	Clostridiales	Clostridiales_vadinBB6	norank_f__Clostridiales_vadinBB60_	unclassified_g__norank_f__Clostridiales_vadinBB60_group	OTU241	0	0	0	1
Tenericutes	Mollicutes	Mollicutes_RF9	norank_o__Mollicutes	norank_o__Mollicutes_RF9	unclassified_g__norank_o__Mollicutes_RF9	OTU803	257	3	0	0
Firmicutes	Clostridia	Clostridiales	Lachnospiraceae	unclassified_f__Lachnospiraceae	unclassified_f__Lachnospiraceae	OTU81	16	3	6	112
Firmicutes	Clostridia	Clostridiales	Ruminococcaceae	Ruminococcaceae_UCG-014	unclassified_g__Ruminococcaceae_UCG-014	OTU12	88	67	39	37
Firmicutes	Clostridia	Clostridiales	Christensenellaceae	Christensenellaceae_R-7_group	unclassified_g__Christensenellaceae_R-7_group	OTU165	0	0	1	0
Firmicutes	Clostridia	Clostridiales	unclassified_o__Clostr	unclassified_o__Clostridiales	unclassified_o__Clostridiales	OTU682	0	1	3	7
Firmicutes	Clostridia	Clostridiales	Ruminococcaceae	unclassified_f__Ruminococcaceae	unclassified_f__Ruminococcaceae	OTU679	178	215	22	16
Firmicutes	Clostridia	Clostridiales	Ruminococcaceae	unclassified_f__Ruminococcaceae	unclassified_f__Ruminococcaceae	OTU455	2	6	14	10
Firmicutes	Clostridia	Clostridiales	Family_XIII	unclassified_f__Family_XIII	unclassified_f__Family_XIII	OTU488	22	43	19	49
Firmicutes	Clostridia	Clostridiales	Ruminococcaceae	Ruminococcaceae_UCG-013	uncultured_bacterium_g__Ruminococcaceae_UCG-013	OTU498	1	1	4	26
Firmicutes	Clostridia	Clostridiales	Ruminococcaceae	Anaerotruncus	unclassified_g__Anaerotruncus	OTU566	5	5	77	0

Firmicutes	Clostridia	Clostridiales	Clostridiales_vadinBB6(	norank_f__Clostridiales_vadinBB60_	uncultured_Firmicutes_bacterium_g__norank_f__Clostridiales_vadinBB60_group	OTU638	0	3	11	15
Firmicutes	Clostridia	Clostridiales	Clostridiales_vadinBB6(	norank_f__Clostridiales_vadinBB60_	uncultured_bacterium_g__norank_f__Clostridiales_vadinBB60_group	OTU3	0	80	0	0
Firmicutes	Clostridia	Clostridiales	Clostridiales_vadinBB6(	norank_f__Clostridiales_vadinBB60_	uncultured_bacterium_g__norank_f__Clostridiales_vadinBB60_group	OTU494	21	34	21	48
Firmicutes	Clostridia	Clostridiales	Ruminococcaceae	Ruminiclostridium_5	uncultured_bacterium_g__Ruminiclostridium_5	OTU849	16	30	12	6
Firmicutes	Clostridia	Clostridiales	Ruminococcaceae	Ruminococcaceae_UCG-014	uncultured_bacterium_g__Ruminococcaceae_UCG-014	OTU114	156	0	89	39
Firmicutes	Clostridia	Clostridiales	Ruminococcaceae	Ruminococcaceae_UCG-010	uncultured_bacterium_g__Ruminococcaceae_UCG-010	OTU326	5	1	6	171
Firmicutes	Clostridia	Clostridiales	Ruminococcaceae	Ruminococcaceae_UCG-014	uncultured_bacterium_g__Ruminococcaceae_UCG-014	OTU749	80	27	54	24
Firmicutes	Clostridia	Clostridiales	Ruminococcaceae	[Eubacterium]_coprostanoligenes_g	unclassified_g__[Eubacterium]_coprostanoligenes_group	OTU233	60	42	64	68
Firmicutes	Clostridia	Clostridiales	Ruminococcaceae	Ruminococcaceae_UCG-014	unidentified_rumen_bacterium_JW32	OTU36	6	28	7	164
Firmicutes	Clostridia	Clostridiales	Ruminococcaceae	Ruminococcaceae_UCG-008	uncultured_bacterium_g__Ruminococcaceae_UCG-008	OTU39	0	0	0	0
Firmicutes	Clostridia	Clostridiales	Family_XIII	Family_XIII_UCG-001	uncultured_bacterium_g__Family_XIII_UCG-001	OTU329	16	45	21	7
Firmicutes	Clostridia	Clostridiales	Ruminococcaceae	Ruminiclostridium_5	uncultured_bacterium_g__Ruminiclostridium_5	OTU75	31	36	12	8
Firmicutes	Clostridia	Clostridiales	Peptococcaceae	norank_f__Peptococcaceae	uncultured_organism_g__norank_f__Peptococcaceae	OTU343	240	0	0	0
Firmicutes	Clostridia	Clostridiales	Ruminococcaceae	Anaerofilum	uncultured_bacterium_g__Anaerofilum	OTU660	45	4	66	5
Firmicutes	Clostridia	Clostridiales	Lachnospiraceae	Marvinbryantia	uncultured_bacterium_g__Marvinbryantia	OTU536	22	11	30	14
Firmicutes	Clostridia	Clostridiales	Ruminococcaceae	Anaerotruncus	unclassified_g__Anaerotruncus	OTU10	20	18	6	14
Firmicutes	Clostridia	Clostridiales	Clostridiales_vadinBB6(	norank_f__Clostridiales_vadinBB60_	uncultured_bacterium_g__norank_f__Clostridiales_vadinBB60_group	OTU678	51	19	0	0
Firmicutes	Clostridia	Clostridiales	Ruminococcaceae	Ruminiclostridium_5	unclassified_g__Ruminiclostridium_5	OTU84	9	40	18	10
Firmicutes	Clostridia	Clostridiales	unclassified_o__Clostr	unclassified_o__Clostridiales	unclassified_o__Clostridiales	OTU137	1	2	18	7
Tenericutes	Mollicutes	Mollicutes_RF9	norank_o__Mollicutes	norank_o__Mollicutes_RF9	uncultured_bacterium_g__norank_o__Mollicutes_RF9	OTU604	153	69	15	25
Firmicutes	Erysipelotrichia	Erysipelotrichales	Erysipelotrichaceae	norank_f__Erysipelotrichaceae	uncultured_Erysipelotrichaceae_bacterium_g__norank	OTU772	0	1	0	0
Tenericutes	Mollicutes	Anaeroplasmatales	Anaeroplasmataceae	Anaeroplasma	uncultured_bacterium_g__Anaeroplasma	OTU801	4	86	27	13
Firmicutes	Clostridia	Clostridiales	Ruminococcaceae	Ruminococcaceae_UCG-014	unclassified_g__Ruminococcaceae_UCG-014	OTU220	0	0	0	1
Firmicutes	Clostridia	Clostridiales	Ruminococcaceae	Ruminiclostridium_9	unclassified_g__Ruminiclostridium_9	OTU587	16	54	13	3
Firmicutes	Clostridia	Clostridiales	Ruminococcaceae	norank_f__Ruminococcaceae	human_gut_metagenome_g__norank_f__Ruminococcaceae	OTU85	22	30	33	22
Cyanobacteria	Cyanobacteria	Gastranaerophilales	norank_o__Gastranae	norank_o__Gastranaerophilales	uncultured_bacterium_g__norank_o__Gastranaerophilales	OTU502	127	17	87	13
Firmicutes	Clostridia	Clostridiales	Clostridiales_vadinBB6(	norank_f__Clostridiales_vadinBB60_	uncultured_bacterium_g__norank_f__Clostridiales_vadinBB60_group	OTU17	33	9	0	30
Firmicutes	Clostridia	Clostridiales	Lachnospiraceae	unclassified_f__Lachnospiraceae	unclassified_f__Lachnospiraceae	OTU513	22	16	5	11
Firmicutes	Clostridia	Clostridiales	Ruminococcaceae	unclassified_f__Ruminococcaceae	unclassified_f__Ruminococcaceae	OTU166	12	41	19	8
Tenericutes	Mollicutes	NB1-n	norank_o__NB1-n	norank_o__NB1-n	uncultured_organism_g__norank_o__NB1-n	OTU603	66	24	12	7
Firmicutes	Clostridia	Clostridiales	Ruminococcaceae	Ruminococcaceae_UCG-013	uncultured_bacterium_g__Ruminococcaceae_UCG-013	OTU720	61	8	23	54
Firmicutes	Clostridia	Clostridiales	Ruminococcaceae	Ruminococcaceae_UCG-014	unclassified_g__Ruminococcaceae_UCG-014	OTU347	61	3	26	21
Firmicutes	Clostridia	Clostridiales	Ruminococcaceae	Ruminiclostridium	uncultured_bacterium_g__Ruminiclostridium	OTU576	15	0	14	0
Cyanobacteria	Cyanobacteria	Gastranaerophilales	norank_o__Gastranae	norank_o__Gastranaerophilales	unclassified_g__norank_o__Gastranaerophilales	OTU563	38	2	23	0
Firmicutes	Clostridia	Clostridiales	Lachnospiraceae	unclassified_f__Lachnospiraceae	unclassified_f__Lachnospiraceae	OTU731	21	9	39	9
Firmicutes	Clostridia	Clostridiales	Lachnospiraceae	Roseburia	unclassified_g__Roseburia	OTU449	5	0	46	23
Firmicutes	Clostridia	Clostridiales	Clostridiales_vadinBB6(	norank_f__Clostridiales_vadinBB60_	unclassified_g__norank_f__Clostridiales_vadinBB60_group	OTU669	0	2	0	0
Tenericutes	Mollicutes	Mollicutes_RF9	norank_o__Mollicutes	norank_o__Mollicutes_RF9	unclassified_g__norank_o__Mollicutes_RF9	OTU104	600	0	0	0
Firmicutes	Clostridia	Clostridiales	Lachnospiraceae	Lachnospiraceae_UCG-010	uncultured_bacterium_g__Lachnospiraceae_UCG-010	OTU663	83	16	20	5
Firmicutes	Clostridia	Clostridiales	Ruminococcaceae	unclassified_f__Ruminococcaceae	unclassified_f__Ruminococcaceae	OTU847	15	2	1	14
Firmicutes	Clostridia	Clostridiales	Ruminococcaceae	Ruminococcaceae_UCG-013	uncultured_bacterium_g__Ruminococcaceae_UCG-013	OTU721	1	26	14	8
Tenericutes	Mollicutes	Mollicutes_RF9	norank_o__Mollicutes	norank_o__Mollicutes_RF9	uncultured_bacterium_g__norank_o__Mollicutes_RF9	OTU627	15	4	34	41
Firmicutes	Clostridia	Clostridiales	Ruminococcaceae	[Eubacterium]_coprostanoligenes_g	unclassified_g__[Eubacterium]_coprostanoligenes_group	OTU121	0	0	2	52
Firmicutes	Clostridia	Clostridiales	Clostridiales_vadinBB6(	norank_f__Clostridiales_vadinBB60_	unclassified_g__norank_f__Clostridiales_vadinBB60_group	OTU180	23	53	20	10
Firmicutes	Clostridia	Clostridiales	Christensenellaceae	Christensenellaceae_R-7_group	unclassified_g__Christensenellaceae_R-7_group	OTU54	41	0	0	0
Firmicutes	Clostridia	Clostridiales	Ruminococcaceae	norank_f__Ruminococcaceae	unclassified_g__norank_f__Ruminococcaceae	OTU534	1	22	0	9
Firmicutes	Clostridia	Clostridiales	Ruminococcaceae	unclassified_f__Ruminococcaceae	unclassified_f__Ruminococcaceae	OTU493	37	29	9	13
Firmicutes	Clostridia	Clostridiales	Lachnospiraceae	norank_f__Lachnospiraceae	unclassified_g__norank_f__Lachnospiraceae	OTU560	8	0	60	8
Firmicutes	Clostridia	Clostridiales	Lachnospiraceae	[Eubacterium]_ventriosum_group	uncultured_organism_g__[Eubacterium]_ventriosum_group	OTU464	22	7	17	55
Tenericutes	Mollicutes	Mollicutes_RF9	norank_o__Mollicutes	norank_o__Mollicutes_RF9	unclassified_g__norank_o__Mollicutes_RF9	OTU234	166	2	38	8
Firmicutes	Clostridia	Clostridiales	Lachnospiraceae	unclassified_f__Lachnospiraceae	unclassified_f__Lachnospiraceae	OTU64	4	3	30	13
Tenericutes	Mollicutes	Mollicutes_RF9	norank_o__Mollicutes	norank_o__Mollicutes_RF9	uncultured_bacterium_g__norank_o__Mollicutes_RF9	OTU602	8	0	0	3
Firmicutes	Clostridia	Clostridiales	Ruminococcaceae	Anaerotruncus	uncultured_bacterium_g__Anaerotruncus	OTU6	0	5	3	12
Firmicutes	Bacilli	Lactobacillales	Lactobacillaceae	Lactobacillus	Lactobacillus_murinus_g__Lactobacillus	OTU843	0	1	0	0
Firmicutes	Clostridia	Clostridiales	Christensenellaceae	Christensenellaceae_R-7_group	uncultured_prokaryote_g__Christensenellaceae_R-7_group	OTU67	2	0	6	64
Firmicutes	Clostridia	Clostridiales	Clostridiales_vadinBB6(	norank_f__Clostridiales_vadinBB60_	uncultured_bacterium_g__norank_f__Clostridiales_vadinBB60_group	OTU748	1	9	16	48
Firmicutes	Clostridia	Clostridiales	Lachnospiraceae	Coprococcus_1	unclassified_g__Coprococcus_1	OTU739	3	13	20	16
Tenericutes	Mollicutes	Mollicutes_RF9	norank_o__Mollicutes	norank_o__Mollicutes_RF9	unclassified_g__norank_o__Mollicutes_RF9	OTU229	106	12	0	33
Firmicutes	Clostridia	Clostridiales	Clostridiales_vadinBB6(	norank_f__Clostridiales_vadinBB60_	uncultured_bacterium_g__norank_f__Clostridiales_vadinBB60_group	OTU23	0	0	25	0
Firmicutes	Clostridia	Clostridiales	Clostridiales_vadinBB6(	norank_f__Clostridiales_vadinBB60_	uncultured_bacterium_g__norank_f__Clostridiales_vadinBB60_group	OTU131	0	0	0	0
Firmicutes	Clostridia	Clostridiales	Ruminococcaceae	Ruminococcaceae_UCG-014	unclassified_g__Ruminococcaceae_UCG-014	OTU837	0	0	66	0
Firmicutes	Clostridia	Clostridiales	unclassified_o__Clostr	unclassified_o__Clostridiales	unclassified_o__Clostridiales	OTU91	45	37	10	22
Firmicutes	Clostridia	Clostridiales	Lachnospiraceae	Roseburia	unclassified_g__Roseburia	OTU43	8	11	5	5
Firmicutes	Clostridia	Clostridiales	Lachnospiraceae	unclassified_f__Lachnospiraceae	unclassified_f__Lachnospiraceae	OTU11	2	2	18	6
Proteobacteria	Betaproteobacteria	Burkholderiales	Oxalobacteraceae	Oxalobacter	unclassified_g__Oxalobacter	OTU557	0	0	0	0
Firmicutes	Clostridia	Clostridiales	Ruminococcaceae	Faecalibacterium	unclassified_g__Faecalibacterium	OTU219	0	1	7	2
Firmicutes	Clostridia	Clostridiales	Ruminococcaceae	Ruminiclostridium_5	uncultured_bacterium_g__Ruminiclostridium_5	OTU59	21	4	27	1
Firmicutes	Clostridia	Clostridiales	Lachnospiraceae	Lachnospiraceae_UCG-010	uncultured_bacterium_g__Lachnospiraceae_UCG-010	OTU755	4	17	18	28
Bacteroidetes	Bacteroidia	Bacteroidales	Rikenellaceae	Alistipes	uncultured_bacterium_g__Alistipes	OTU765	0	0	0	0
Firmicutes	Erysipelotrichia	Erysipelotrichales	Erysipelotrichaceae	[Clostridium]_innocuum_group	uncultured_bacterium_g__[Clostridium]_innocuum_group	OTU794	0	0	0	0
Firmicutes	Clostridia	Clostridiales	unclassified_o__Clostr	unclassified_o__Clostridiales	unclassified_o__Clostridiales	OTU673	18	7	8	12
Firmicutes	Clostridia	Clostridiales	Clostridiales_vadinBB6(	norank_f__Clostridiales_vadinBB60_	gut_metagenome_g__norank_f__Clostridiales_vadinBB60_group	OTU548	8	33	16	2
Tenericutes	Mollicutes	Mollicutes_RF9	norank_o__Mollicutes	norank_o__Mollicutes_RF9	uncultured_bacterium_g__norank_o__Mollicutes_RF9	OTU544	11	10	1	0
Cyanobacteria	Cyanobacteria	Gastranaerophilales	norank_o__Gastranae	norank_o__Gastranaerophilales	unclassified_g__norank_o__Gastranaerophilales	OTU155	40	50	45	29
Firmicutes	Clostridia	Clostridiales	Clostridiales_vadinBB6(	norank_f__Clostridiales_vadinBB60_	uncultured_bacterium_g__norank_f__Clostridiales_vadinBB60_group	OTU481	38	33	0	39
Firmicutes	Clostridia	Clostridiales	Lachnospiraceae	unclassified_f__Lachnospiraceae	unclassified_f__Lachnospiraceae	OTU467	10	6	19	6
Firmicutes	Clostridia	Clostridiales	Ruminococcaceae	norank_f__Ruminococcaceae	unclassified_g__norank_f__Ruminococcaceae	OTU622	0	1	2	0
Firmicutes	Clostridia	Clostridiales	Lachnospiraceae	unclassified_f__Lachnospiraceae	unclassified_f__Lachnospiraceae	OTU201	0	5	11	4
Firmicutes	Clostridia	Clostridiales	Lachnospiraceae	norank_f__Lachnospiraceae	uncultured_organism_g__norank_f__Lachnospiraceae	OTU654	32	0	0	0
Firmicutes	Clostridia	Clostridiales	Ruminococcaceae	Ruminococcaceae_UCG-014	unclassified_g__Ruminococcaceae_UCG-014	OTU575	8	0	0	52
Tenericutes	Mollicutes	Mollicutes_RF9	norank_o__Mollicutes	norank_o__Mollicutes_RF9	unclassified_g__norank_o__Mollicutes_RF9	OTU600	83	0	2	29
Firmicutes	Clostridia	Clostridiales	Ruminococcaceae	unclassified_f__Ruminococcaceae	unclassified_f__Ruminococcaceae	OTU728	2	4	13	3
Firmicutes	Clostridia	Clostridiales	Ruminococcaceae	Anaerotruncus	unclassified_g__Anaerotruncus	OTU159	0	1	0	0
Tenericutes	Mollicutes	Mollicutes_RF9	norank_o__Mollicutes	norank_o__Mollicutes_RF9	uncultured_bacterium_g__norank_o__Mollicutes_RF9	OTU608	49	5	14	52
Firmicutes	Clostridia	Clostridiales	Ruminococcaceae	Ruminococcaceae_UCG-010	uncultured_bacterium_g__Ruminococcaceae_UCG-010	OTU674	23	21	0	51
Firmicutes	Clostridia	Clostridiales	Lachnospiraceae	Lachnospiraceae_FCS020_group	uncultured_bacterium_g__Lachnospiraceae_FCS020_group	OTU802	19	5	24	6
Firmicutes	Clostridia	Clostridiales	Ruminococcaceae	Caproiciproducens	uncultured_bacterium_g__Caproiciproducens	OTU68	5	12	12	2
Firmicutes	Clostridia	Clostridiales	Peptococcaceae	norank_f__Peptococcaceae	unclassified_g__norank_f__Peptococcaceae	OTU586	107	13	10	5
Firmicutes	Clostridia	Clostridiales	Ruminococcaceae	Anaerotruncus	uncultured_bacterium_g__Anaerotruncus	OTU175	16	5	17	17
Firmicutes	Clostridia	Clostridiales	Lachnospiraceae	unclassified_f__Lachnospiraceae	unclassified_f__Lachnospiraceae	OTU854	8	1	0	0
Firmicutes	Clostridia	Clostridiales	Christensenellaceae	Christensenellaceae_R-7_group	uncultured_bacterium_g__Christensenellaceae_R-7_group	OTU164	46	12	7	2
Firmicutes	Clostridia	Clostridiales	Ruminococcaceae	Ruminococcus_2	uncultured_bacterium_g__Ruminococcus_2	OTU447	0	0	0	0
Actinobacteria	Actinobacteria	Coriobacteriales	Coriobacteriaceae	unclassified_f__Coriobacteriaceae	unclassified_f__Coriobacteriaceae	OTU490	14	18	6	48
Proteobacteria	Alphaproteobacteria	Rhizobiales	Brucellaceae	Brucella	Brucella_melitensis_g__Brucella	OTU812	2	0	0	0
Firmicutes	Clostridia	Clostridiales	Ruminococcaceae	Ruminiclostridium_1	uncultured_bacterium_g__Ruminiclostridium_1	OTU485	14	37	4	3
Tenericutes	Mollicutes	Mollicutes_RF9	norank_o__Mollicutes	norank_o__Mollicutes_RF9	unclassified_g__norank_o__Mollicutes_RF9	OTU611	33	18	10	61
Firmicutes	Clostridia	Clostridiales	Ruminococcaceae	Ruminococcaceae_UCG-014	unclassified_g__Ruminococcaceae_UCG-014	OTU100	42	0	6	0
Firmicutes	Clostridia	Clostridiales	Ruminococcaceae	norank_f__Ruminococcaceae	unclassified_g__norank_f__Ruminococcaceae	OTU98	117	1	0	0
Firmicutes	Bacilli	Lactobacillales	Enterococcaceae	Enterococcus	Enterococcus_faecalis_g__Enterococcus	OTU763	0	2	0	0
Firmicutes	Bacilli	Lactobacillales	Lactobacillaceae	Lactobacillus	Lactobacillus_coleohominis	OTU628	12	0	0	10
Firmicutes	Erysipelotrichia	Erysipelotrichales	Erysipelotrichaceae	unclassified_f__Erysipelotrichaceae	unclassified_f__Erysipelotrichaceae	OTU839	82	33	11	13
Firmicutes	Clostridia	Clostridiales	Christensenellaceae	Christensenellaceae_R-7_group	bacterium_YE57	OTU754	7	11	15	3
Firmicutes	Clostridia	Clostridiales	Ruminococcaceae	Ruminiclostridium_9	unclassified_g__Ruminiclostridium_9	OTU500	3	1	0	0
Firmicutes	Clostridia	Clostridiales	Ruminococcaceae	Faecalibacterium	unclassified_g__Faecalibacterium	OTU846	3	11	10	4
Tenericutes	Mollicutes	Mollicutes_RF9	norank_o__Mollicutes	norank_o__Mollicutes_RF9	unclassified_g__norank_o__Mollicutes_RF9	OTU610	1	0	0	0
Firmicutes	Clostridia	Clostridiales	Ruminococcaceae	norank_f__Ruminococcaceae	unclassified_g__norank_f__Ruminococcaceae	OTU132	0	2	1	4
Firmicutes	Clostridia	Clostridiales	Ruminococcaceae	Ruminococcus_1	uncultured_bacterium_g__Ruminococcus_1	OTU639	0	0	65	0
Firmicutes	Clostridia	Clostridiales	Peptococcaceae	unclassified_f__Peptococcaceae	unclassified_f__Peptococcaceae	OTU483	23	3	22	13
Tenericutes	Mollicutes	Mollicutes_RF9	norank_o__Mollicutes	norank_o__Mollicutes_RF9	uncultured_bacterium_g__norank_o__Mollicutes_RF9	OTU631	15	16	15	0
Firmicutes	Clostridia	Clostridiales	Ruminococcaceae	Caproiciproducens	unclassified_g__Caproiciproducens	OTU824	81	27	0	4
Firmicutes	Clostridia	Clostridiales	Clostridiales_vadinBB6(	norank_f__Clostridiales_vadinBB60_	uncultured_bacterium_g__norank_f__Clostridiales_vadinBB60_group	OTU757	0	0	0	0

Tenericutes	Mollicutes	Mollicutes_RF9	norank_o__Mollicutes	norank_o__Mollicutes_RF9	uncultured_bacterium_g__norank_o__Mollicutes_RF9	OTU792	11	8	8	7
Firmicutes	Clostridia	Clostridiales	Lachnospiraceae	Coprococcus_1	uncultured_bacterium_g__Coprococcus_1	OTU629	12	0	0	0
Firmicutes	Clostridia	Clostridiales	Peptococcaceae	Peptococcus	uncultured_organism_g__Peptococcus	OTU665	20	4	28	15
Firmicutes	Clostridia	Clostridiales	Ruminococcaceae	Ruminococcaceae_UCG-014	unclassified_g__Ruminococcaceae_UCG-014	OTU532	0	0	0	0
Firmicutes	Clostridia	Clostridiales	Ruminococcaceae	norank_f__Ruminococcaceae	unclassified_g__norank_f__Ruminococcaceae	OTU671	9	10	11	7
Firmicutes	Clostridia	Clostridiales	Ruminococcaceae	Ruminococcus_1	uncultured_organism_g__Ruminococcus_1	OTU56	8	4	13	11
Firmicutes	Clostridia	Clostridiales	Clostridiales_vadinBB6	norank_f__Clostridiales_vadinBB60_	uncultured_bacterium_g__norank_f__Clostridiales_vadinBB60_group	OTU714	25	42	11	4
Firmicutes	Clostridia	Clostridiales	Lachnospiraceae	unclassified_f__Lachnospiraceae	unclassified_f__Lachnospiraceae	OTU726	7	9	15	10
Firmicutes	Clostridia	Clostridiales	Ruminococcaceae	norank_f__Ruminococcaceae	Ruminococcaceae_bacterium_GD7	OTU72	42	0	8	3
Firmicutes	Erysipelotrichia	Erysipelotrichales	Erysipelotrichaceae	Holdemania	unclassified_g__Holdemania	OTU702	76	16	4	2
Firmicutes	Clostridia	Clostridiales	Ruminococcaceae	[Eubacterium]_coprostanoligenes_g	unclassified_g__[Eubacterium]_coprostanoligenes_group	OTU747	4	11	1	1
Firmicutes	Clostridia	Clostridiales	Family_XIII	Family_XIII_AD3011_group	uncultured_bacterium_g__Family_XIII_AD3011_group	OTU579	3	6	3	18
Tenericutes	Mollicutes	Mollicutes_RF9	norank_o__Mollicutes	norank_o__Mollicutes_RF9	uncultured_bacterium_g__norank_o__Mollicutes_RF9	OTU756	226	0	2	2
Actinobacteria	Actinobacteria	Coriobacteriales	Coriobacteriaceae	unclassified_f__Coriobacteriaceae	unclassified_f__Coriobacteriaceae	OTU4	27	9	2	9
Firmicutes	Clostridia	Clostridiales	Ruminococcaceae	Candidatus_Soleaferrea	uncultured_organism_g__Candidatus_Soleaferrea	OTU492	8	10	4	2
Firmicutes	Clostridia	Clostridiales	Ruminococcaceae	[Eubacterium]_coprostanoligenes_g	unclassified_g__[Eubacterium]_coprostanoligenes_group	OTU656	18	2	16	8
Firmicutes	Erysipelotrichia	Erysipelotrichales	Erysipelotrichaceae	Erysipelatoclostridium	unclassified_g__Erysipelatoclostridium	OTU506	130	0	6	0
Firmicutes	Clostridia	Clostridiales	Ruminococcaceae	Anaerotruncus	uncultured_bacterium_g__Anaerotruncus	OTU42	8	9	2	5
Firmicutes	Clostridia	Clostridiales	Lachnospiraceae	unclassified_f__Lachnospiraceae	unclassified_f__Lachnospiraceae	OTU509	1	0	3	2
Firmicutes	Clostridia	Clostridiales	Lachnospiraceae	Tyzzerella	uncultured_bacterium_g__Tyzzerella	OTU777	9	16	8	4
Firmicutes	Clostridia	Clostridiales	Clostridiales_vadinBB6	norank_f__Clostridiales_vadinBB60_	gut_metagenome_g__norank_f__Clostridiales_vadinBB60_group	OTU19	21	19	9	3
Firmicutes	Clostridia	Clostridiales	Ruminococcaceae	Ruminococcus_2	unclassified_g__Ruminococcus_2	OTU549	1	0	3	0
Firmicutes	Clostridia	Clostridiales	Ruminococcaceae	Anaerofilum	uncultured_bacterium_g__Anaerofilum	OTU677	1	3	18	1
Firmicutes	Clostridia	Clostridiales	Ruminococcaceae	Ruminococcaceae_UCG-014	unclassified_g__Ruminococcaceae_UCG-014	OTU487	2	7	11	11
Firmicutes	Clostridia	Clostridiales	Ruminococcaceae	Ruminococcaceae_UCG-013	unclassified_g__Ruminococcaceae_UCG-013	OTU145	49	46	4	2
Bacteroidetes	Bacteroidia	Bacteroidales	Rikenellaceae	Alistipes	unclassified_g__Alistipes	OTU667	0	2	2	12
Firmicutes	Clostridia	Clostridiales	Lachnospiraceae	unclassified_f__Lachnospiraceae	unclassified_f__Lachnospiraceae	OTU771	3	1	1	4
Firmicutes	Clostridia	Clostridiales	Ruminococcaceae	Ruminococcaceae_UCG-009	uncultured_bacterium_g__Ruminococcaceae_UCG-009	OTU750	8	21	23	26
Tenericutes	Mollicutes	Mollicutes_RF9	norank_o__Mollicutes	norank_o__Mollicutes_RF9	uncultured_bacterium_g__norank_o__Mollicutes_RF9	OTU543	100	17	6	0
Firmicutes	Clostridia	Clostridiales	unclassified_o__Clostr	unclassified_o__Clostridiales	unclassified_o__Clostridiales	OTU535	2	0	2	8
Tenericutes	Mollicutes	Mollicutes_RF9	norank_o__Mollicutes	norank_o__Mollicutes_RF9	uncultured_bacterium_g__norank_o__Mollicutes_RF9	OTU153	14	10	18	6
Firmicutes	Clostridia	Clostridiales	Clostridiales_vadinBB6	norank_f__Clostridiales_vadinBB60_	uncultured_bacterium_g__norank_f__Clostridiales_vadinBB60_group	OTU409	4	6	4	3
Firmicutes	Clostridia	Clostridiales	Ruminococcaceae	Oscillibacter	unclassified_g__Oscillibacter	OTU609	3	1	2	0
Firmicutes	Clostridia	Clostridiales	Ruminococcaceae	Butyricicoccus	unclassified_g__Butyricicoccus	OTU411	2	2	0	4
Firmicutes	Clostridia	Clostridiales	unclassified_o__Clostridiales	unclassified_o__Clostridiales	unclassified_o__Clostridiales	OTU34	26	0	2	2
Firmicutes	Clostridia	Clostridiales	Clostridiales_vadinBB6	norank_f__Clostridiales_vadinBB60_	uncultured_rumen_bacterium_g__norank_f__Clostridiales_vadinBB60_group	OTU517	129	27	0	4
Firmicutes	Clostridia	Clostridiales	Ruminococcaceae	Ruminococcaceae_UCG-014	uncultured_bacterium_g__Ruminococcaceae_UCG-014	OTU850	3	0	0	0
Firmicutes	Clostridia	Clostridiales	Ruminococcaceae	Ruminiclostridium_5	uncultured_organism_g__Ruminiclostridium_5	OTU541	3	3	1	4
Firmicutes	Clostridia	Clostridiales	Lachnospiraceae	Tyzzerella_3	uncultured_bacterium_g__Tyzzerella_3	OTU635	4	0	20	2
Firmicutes	Clostridia	Clostridiales	Eubacteriaceae	Anaerofustis	Anaerofustis_sp._Marseille-P2441	OTU136	6	5	11	18
Firmicutes	Clostridia	Clostridiales	Ruminococcaceae	Ruminococcaceae_UCG-013	unclassified_g__Ruminococcaceae_UCG-013	OTU76	3	4	6	5
Firmicutes	Clostridia	Clostridiales	Ruminococcaceae	Anaerotruncus	unclassified_g__Anaerotruncus	OTU542	38	2	6	7
Firmicutes	Clostridia	Clostridiales	Ruminococcaceae	unclassified_f__Ruminococcaceae	unclassified_f__Ruminococcaceae	OTU831	0	4	10	1
Firmicutes	Clostridia	Clostridiales	Ruminococcaceae	Ruminococcaceae_NK4A214_group	unclassified_g__Ruminococcaceae_NK4A214_group	OTU168	8	3	35	5
Firmicutes	Clostridia	Clostridiales	Ruminococcaceae	Ruminococcaceae_UCG-009	unclassified_g__Ruminococcaceae_UCG-009	OTU71	17	14	11	12
Firmicutes	Clostridia	Clostridiales	Ruminococcaceae	Ruminococcaceae_UCG-013	uncultured_organism_g__Ruminococcaceae_UCG-013	OTU204	0	0	2	0
Actinobacteria	Actinobacteria	Coriobacteriales	Coriobacteriaceae	Enterorhabdus	uncultured_bacterium_g__Enterorhabdus	OTU221	11	7	4	17
Tenericutes	Mollicutes	Mollicutes_RF9	norank_o__Mollicutes	norank_o__Mollicutes_RF9	unclassified_g__norank_o__Mollicutes_RF9	OTU788	32	4	2	23
Firmicutes	Clostridia	Clostridiales	Ruminococcaceae	Ruminococcaceae_UCG-010	uncultured_organism_g__Ruminococcaceae_UCG-010	OTU20	16	20	3	7
Firmicutes	Clostridia	Clostridiales	Ruminococcaceae	Anaerotruncus	uncultured_bacterium_g__Anaerotruncus	OTU582	0	1	1	0
Firmicutes	Clostridia	Clostridiales	Family_XIII	Family_XIII_AD3011_group	uncultured_bacterium_g__Family_XIII_AD3011_group	OTU634	3	5	0	5
Firmicutes	Clostridia	Clostridiales	Ruminococcaceae	Anaerofilum	uncultured_bacterium_g__Anaerofilum	OTU832	0	0	0	0
Firmicutes	Clostridia	Clostridiales	Clostridiales_vadinBB6	norank_f__Clostridiales_vadinBB60_	uncultured_bacterium_g__norank_f__Clostridiales_vadinBB60_group	OTU38	0	0	0	0
Firmicutes	Clostridia	Clostridiales	unclassified_o__Clostr	unclassified_o__Clostridiales	unclassified_o__Clostridiales	OTU672	0	2	18	14
Firmicutes	Clostridia	Clostridiales	Ruminococcaceae	unclassified_f__Ruminococcaceae	unclassified_f__Ruminococcaceae	OTU661	16	7	8	18
Firmicutes	Clostridia	Clostridiales	Ruminococcaceae	unclassified_f__Ruminococcaceae	unclassified_f__Ruminococcaceae	OTU169	11	7	20	5
Tenericutes	Mollicutes	Mollicutes_RF9	norank_o__Mollicutes	norank_o__Mollicutes_RF9	uncultured_bacterium_g__norank_o__Mollicutes_RF9	OTU820	0	0	1	1
Firmicutes	Clostridia	Clostridiales	Eubacteriaceae	Eubacterium	Eubacterium_limosum	OTU337	9	10	0	10
Tenericutes	Mollicutes	Mollicutes_RF9	norank_o__Mollicutes	norank_o__Mollicutes_RF9	uncultured_bacterium_g__norank_o__Mollicutes_RF9	OTU584	29	0	28	2
Firmicutes	Clostridia	Clostridiales	Ruminococcaceae	Anaerofilum	uncultured_bacterium_g__Anaerofilum	OTU643	9	2	2	8
Firmicutes	Clostridia	Clostridiales	Clostridiales_vadinBB6	norank_f__Clostridiales_vadinBB60_	uncultured_bacterium_g__norank_f__Clostridiales_vadinBB60_group	OTU35	3	53	3	23
Firmicutes	Clostridia	Clostridiales	Lachnospiraceae	Coprococcus_1	uncultured_bacterium_g__Coprococcus_1	OTU811	7	5	6	0
Firmicutes	Clostridia	Clostridiales	Ruminococcaceae	Ruminococcaceae_UCG-014	unclassified_g__Ruminococcaceae_UCG-014	OTU87	3	0	6	28
Firmicutes	Clostridia	Clostridiales	Ruminococcaceae	[Eubacterium]_coprostanoligenes_g	unclassified_g__[Eubacterium]_coprostanoligenes_group	OTU751	0	19	2	17
Firmicutes	Clostridia	Clostridiales	Clostridiales_vadinBB6	norank_f__Clostridiales_vadinBB60_	uncultured_bacterium_g__norank_f__Clostridiales_vadinBB60_group	OTU694	0	0	0	0
Firmicutes	Clostridia	Clostridiales	Clostridiales_vadinBB6	norank_f__Clostridiales_vadinBB60_	uncultured_Thermoanaerobacterales_bacterium_g__norank_f__Clostridiales_vadinBB60_gr	OTU224	27	5	5	7
Firmicutes	Clostridia	Clostridiales	Lachnospiraceae	unclassified_f__Lachnospiraceae	unclassified_f__Lachnospiraceae	OTU655	18	5	20	1
Firmicutes	Clostridia	Clostridiales	Ruminococcaceae	Oscillospira	uncultured_bacterium_g__Oscillospira	OTU574	7	3	6	0
Firmicutes	Clostridia	Clostridiales	Clostridiales_vadinBB6	norank_f__Clostridiales_vadinBB60_	unclassified_g__norank_f__Clostridiales_vadinBB60_group	OTU2	0	0	0	0
Firmicutes	Clostridia	Clostridiales	Clostridiales_vadinBB6	norank_f__Clostridiales_vadinBB60_	unclassified_g__norank_f__Clostridiales_vadinBB60_group	OTU778	0	1	0	0
Firmicutes	Clostridia	Clostridiales	Ruminococcaceae	Ruminococcaceae_UCG-013	unclassified_g__Ruminococcaceae_UCG-013	OTU676	6	0	0	0
Tenericutes	Mollicutes	Mollicutes_RF9	norank_o__Mollicutes	norank_o__Mollicutes_RF9	unclassified_g__norank_o__Mollicutes_RF9	OTU626	4	0	6	7
Firmicutes	Clostridia	Clostridiales	Ruminococcaceae	Ruminococcaceae_UCG-010	unclassified_g__Ruminococcaceae_UCG-010	OTU61	15	0	0	13
Firmicutes	Clostridia	Clostridiales	Ruminococcaceae	norank_f__Ruminococcaceae	unclassified_g__norank_f__Ruminococcaceae	OTU516	5	8	6	3
Firmicutes	Clostridia	Clostridiales	Ruminococcaceae	unclassified_f__Ruminococcaceae	unclassified_f__Ruminococcaceae	OTU228	7	5	4	0
Firmicutes	Clostridia	Clostridiales	Ruminococcaceae	Ruminococcaceae_UCG-010	uncultured_bacterium_g__Ruminococcaceae_UCG-010	OTU203	0	0	0	0
Firmicutes	Clostridia	Clostridiales	Ruminococcaceae	Anaerotruncus	uncultured_bacterium_g__Anaerotruncus	OTU853	3	23	0	0
Firmicutes	Clostridia	Thermoanaerobacte	Thermoanaerobactera	Gelria	uncultured_bacterium_g__Gelria	OTU16	20	13	5	0
Firmicutes	Clostridia	Clostridiales	Clostridiales_vadinBB6	norank_f__Clostridiales_vadinBB60_	uncultured_bacterium_g__norank_f__Clostridiales_vadinBB60_group	OTU143	32	47	0	1
Firmicutes	Clostridia	Clostridiales	Family_XIII	unclassified_f__Family_XIII	unclassified_f__Family_XIII	OTU537	2	0	7	17
Firmicutes	Clostridia	Clostridiales	Family_XIII	[Eubacterium]_brachy_group	uncultured_bacterium_g__[Eubacterium]_brachy_group	OTU69	5	5	4	4
Firmicutes	Clostridia	Clostridiales	Ruminococcaceae	unclassified_f__Ruminococcaceae	unclassified_f__Ruminococcaceae	OTU14	2	1	2	2
Firmicutes	Clostridia	Clostridiales	Ruminococcaceae	Intestinimonas	uncultured_bacterium_g__Intestinimonas	OTU615	4	6	7	1
Firmicutes	Clostridia	Clostridiales	Lachnospiraceae	unclassified_f__Lachnospiraceae	unclassified_f__Lachnospiraceae	OTU118	5	0	4	4
Firmicutes	Clostridia	Clostridiales	Ruminococcaceae	Anaerotruncus	unclassified_g__Anaerotruncus	OTU505	16	3	2	1
Firmicutes	Bacilli	Lactobacillales	Lactobacillaceae	Lactobacillus	uncultured_bacterium_g__Lactobacillus	OTU637	0	0	0	4
Firmicutes	Clostridia	Clostridiales	Ruminococcaceae	Ruminococcaceae_V9D2013_group	uncultured_bacterium_g__Ruminococcaceae_V9D2013_group	OTU652	14	5	16	1
Firmicutes	Erysipelotrichia	Erysipelotrichales	Erysipelotrichaceae	norank_f__Erysipelotrichaceae	uncultured_Erysipelotrichales_bacterium_g__norank	OTU323	0	0	0	0
Firmicutes	Clostridia	Clostridiales	Ruminococcaceae	Papillibacter	uncultured_bacterium_g__Papillibacter	OTU715	4	9	2	2
Tenericutes	Mollicutes	Mollicutes_RF9	norank_o__Mollicutes	norank_o__Mollicutes_RF9	unclassified_g__norank_o__Mollicutes_RF9	OTU729	83	0	0	0
Cyanobacteria	Cyanobacteria	Gastranaerophilales	norank_o__Gastranae	norank_o__Gastranaerophilales	uncultured_bacterium_g__norank_o__Gastranaerophilales	OTU94	72	0	0	0
Firmicutes	Clostridia	Clostridiales	Ruminococcaceae	Ruminococcaceae_UCG-013	uncultured_bacterium_g__Ruminococcaceae_UCG-013	OTU780	3	0	2	3
Firmicutes	Clostridia	Clostridiales	Ruminococcaceae	Anaerotruncus	unclassified_g__Anaerotruncus	OTU723	7	2	10	3
Firmicutes	Clostridia	Clostridiales	Ruminococcaceae	Anaerotruncus	unclassified_g__Anaerotruncus	OTU101	5	6	3	9
Tenericutes	Mollicutes	Mollicutes_RF9	norank_o__Mollicutes	norank_o__Mollicutes_RF9	unclassified_g__norank_o__Mollicutes_RF9	OTU117	63	0	3	5
Firmicutes	Clostridia	Clostridiales	Ruminococcaceae	unclassified_f__Ruminococcaceae	unclassified_f__Ruminococcaceae	OTU93	6	2	0	3
Firmicutes	Clostridia	Clostridiales	Lachnospiraceae	unclassified_f__Lachnospiraceae	unclassified_f__Lachnospiraceae	OTU759	3	4	0	3
Firmicutes	Clostridia	Clostridiales	Ruminococcaceae	Ruminococcaceae_UCG-014	unclassified_g__Ruminococcaceae_UCG-014	OTU95	32	0	0	0
Firmicutes	Erysipelotrichia	Erysipelotrichales	Erysipelotrichaceae	Dielma	unclassified_g__Dielma	OTU173	0	0	187	0
Firmicutes	Clostridia	Clostridiales	Ruminococcaceae	Anaerotruncus	unclassified_g__Anaerotruncus	OTU124	4	4	14	0
Firmicutes	Clostridia	Clostridiales	Ruminococcaceae	norank_f__Ruminococcaceae	unclassified_g__norank_f__Ruminococcaceae	OTU134	0	3	1	0
Firmicutes	Clostridia	Clostridiales	Lachnospiraceae	unclassified_f__Lachnospiraceae	unclassified_f__Lachnospiraceae	OTU510	4	4	2	5
Actinobacteria	Actinobacteria	Coriobacteriales	Coriobacteriaceae	Gordonibacter	unclassified_g__Gordonibacter	OTU461	2	8	1	3
Proteobacteria	Betaproteobacteria	Burkholderiales	Burkholderiaceae	Burkholderia-Paraburkholderia	unclassified_g__Burkholderia-Paraburkholderia	OTU435	0	0	0	0
Firmicutes	Clostridia	Clostridiales	Family_XIII	Family_XIII_AD3011_group	uncultured_bacterium_g__Family_XIII_AD3011_group	OTU648	9	0	1	2
Firmicutes	Clostridia	Clostridiales	Ruminococcaceae	unclassified_f__Ruminococcaceae	unclassified_f__Ruminococcaceae	OTU722	7	0	0	1
Firmicutes	Clostridia	Clostridiales	Clostridiales_vadinBB6	norank_f__Clostridiales_vadinBB60_	uncultured_bacterium_g__norank_f__Clostridiales_vadinBB60_group	OTU740	54	21	1	9
Bacteroidetes	Bacteroidia	Bacteroidales	Bacteroidales_S24-7_g	norank_f__Bacteroidales_S24-7_gro	uncultured_bacterium_g__norank_f__Bacteroidales_S24-7_group	OTU322	0	0	0	0
Firmicutes	Clostridia	Clostridiales	Ruminococcaceae	unclassified_f__Ruminococcaceae	unclassified_f__Ruminococcaceae	OTU538	15	0	10	2
Firmicutes	Clostridia	Clostridiales	Lachnospiraceae	unclassified_f__Lachnospiraceae	unclassified_f__Lachnospiraceae	OTU255	40	0	4	1
Firmicutes	Clostridia	Clostridiales	Christensenellaceae	Christensenellaceae_R-7_group	unclassified_g__Christensenellaceae_R-7_group	OTU106	15	0	0	0

Firmicutes	Clostridia	Clostridiales	Ruminococcaceae	[Eubacterium]_coprostanoligenes_g	uncultured_bacterium_g__[Eubacterium]_coprostanoligenes_group	OTU129	5	3	3	4
Firmicutes	Erysipelotrichia	Erysipelotrichales	Erysipelotrichaceae	Erysipelatoclostridium	Clostridiales_bacterium_CHKCI006	OTU232	0	0	0	0
Firmicutes	Clostridia	Clostridiales	Ruminococcaceae	unclassified_f__Ruminococcaceae	unclassified_f__Ruminococcaceae	OTU518	7	0	5	27
Firmicutes	Clostridia	Clostridiales	Ruminococcaceae	Ruminococcaceae_UCG-013	uncultured_Clostridiaceae_bacterium_g__Ruminococcaceae_UCG-013	OTU675	4	1	0	4
Firmicutes	Clostridia	Clostridiales	Christensenellaceae	Christensenellaceae_R-7_group	human_gut_metagenome_g__Christensenellaceae_R-7_group	OTU223	0	0	0	0
Firmicutes	Clostridia	Clostridiales	Ruminococcaceae	Faecalibacterium	uncultured_bacterium_g__Faecalibacterium	OTU41	6	2	1	2
Firmicutes	Clostridia	Clostridiales	Ruminococcaceae	Anaerotruncus	unclassified_g__Anaerotruncus	OTU647	0	0	18	0
Firmicutes	Erysipelotrichia	Erysipelotrichales	Erysipelotrichaceae	Turicibacter	uncultured_bacterium_g__Turicibacter	OTU598	0	0	0	0
Firmicutes	Clostridia	Clostridiales	Ruminococcaceae	Subdoligranulum	unclassified_g__Subdoligranulum	OTU188	1	7	3	2
Firmicutes	Clostridia	Clostridiales	Ruminococcaceae	Anaerotruncus	uncultured_Clostridiaceae_bacterium_g__Anaerotruncus	OTU235	14	4	1	1
Firmicutes	Clostridia	Clostridiales	Family_XIII	unclassified_f__Family_XIII	unclassified_f__Family_XIII	OTU236	5	6	3	5
Firmicutes	Clostridia	Clostridiales	Ruminococcaceae	Ruminococcaceae_UCG-014	unidentified_rumen_bacterium_JW32	OTU21	0	26	0	8
Firmicutes	Clostridia	Clostridiales	Ruminococcaceae	Papillibacter	unclassified_g__Papillibacter	OTU521	9	7	0	0
Firmicutes	Clostridia	Clostridiales	Ruminococcaceae	unclassified_f__Ruminococcaceae	unclassified_f__Ruminococcaceae	OTU653	1	9	6	1
Firmicutes	Clostridia	Clostridiales	Peptococcaceae	norank_f__Peptococcaceae	uncultured_bacterium_g__norank_f__Peptococcaceae	OTU605	15	11	0	7
Firmicutes	Bacilli	Lactobacillales	Lactobacillaceae	Lactobacillus	Lactobacillus_ingluviei	OTU182	0	0	1	0
Firmicutes	Clostridia	Clostridiales	Ruminococcaceae	[Eubacterium]_coprostanoligenes_g	unclassified_g__[Eubacterium]_coprostanoligenes_group	OTU333	0	0	0	1
Firmicutes	Clostridia	Clostridiales	unclassified_o__Clostr	unclassified_o__Clostridiales	unclassified_o__Clostridiales	OTU472	0	2	1	2
Firmicutes	Clostridia	Clostridiales	Ruminococcaceae	[Eubacterium]_coprostanoligenes_g	unclassified_g__[Eubacterium]_coprostanoligenes_group	OTU730	0	11	0	0
Firmicutes	Clostridia	Clostridiales	Peptococcaceae	norank_f__Peptococcaceae	unclassified_g__norank_f__Peptococcaceae	OTU172	6	0	11	2
Firmicutes	Clostridia	Clostridiales	Ruminococcaceae	Ruminococcaceae_UCG-013	uncultured_bacterium_g__Ruminococcaceae_UCG-013	OTU658	7	0	0	0
Proteobacteria	Gammaproteobacteria	Enterobacteriales	Enterobacteriaceae	Proteus	Proteus_mirabilis	OTU785	5	18	0	0
Firmicutes	Clostridia	Clostridiales	Ruminococcaceae	norank_f__Ruminococcaceae	unclassified_g__norank_f__Ruminococcaceae	OTU744	3	12	10	0
Firmicutes	Clostridia	Clostridiales	Lachnospiraceae	Tyzzerella_3	unclassified_g__Tyzzerella_3	OTU841	0	1	0	0
Bacteroidetes	Bacteroidia	Bacteroidales	Bacteroidales_S24-7_g	norank_f__Bacteroidales_S24-7_grou	uncultured_bacterium_g__norank_f__Bacteroidales_S24-7_group	OTU360	0	0	0	0
Firmicutes	Clostridia	Clostridiales	Ruminococcaceae	unclassified_f__Ruminococcaceae	unclassified_f__Ruminococcaceae	OTU737	1	11	8	1
Firmicutes	Clostridia	Clostridiales	Ruminococcaceae	Anaerotruncus	unclassified_g__Anaerotruncus	OTU752	4	3	5	0
Firmicutes	Clostridia	Clostridiales	Lachnospiraceae	unclassified_f__Lachnospiraceae	unclassified_f__Lachnospiraceae	OTU63	5	4	4	1
Firmicutes	Clostridia	Clostridiales	Ruminococcaceae	Caproiciproducens	unclassified_g__Caproiciproducens	OTU452	3	2	0	0
Firmicutes	Clostridia	Clostridiales	Ruminococcaceae	Butyricicoccus	unclassified_g__Butyricicoccus	OTU475	1	0	1	6
Proteobacteria	Betaproteobacteria	Burkholderiales	Oxalobacteraceae	Herminiimonas	uncultured_bacterium_g__Herminiimonas	OTU804	5	4	4	0
Tenericutes	Mollicutes	Mollicutes_RF9	norank_o__Mollicutes	norank_o__Mollicutes_RF9	unclassified_g__norank_o__Mollicutes_RF9	OTU630	0	0	3	8
Firmicutes	Clostridia	Clostridiales	Ruminococcaceae	Ruminococcaceae_UCG-013	unclassified_g__Ruminococcaceae_UCG-013	OTU680	0	4	2	3
Tenericutes	Mollicutes	Mollicutes_RF9	norank_o__Mollicutes	norank_o__Mollicutes_RF9	gut_metagenome_g__norank_o__Mollicutes_RF9	OTU773	18	8	1	2
Firmicutes	Clostridia	Clostridiales	Peptococcaceae	norank_f__Peptococcaceae	uncultured_bacterium_g__norank_f__Peptococcaceae	OTU624	3	4	3	3
Firmicutes	Clostridia	Clostridiales	Ruminococcaceae	Ruminiclostridium	unclassified_g__Ruminiclostridium	OTU154	11	7	7	0
Bacteroidetes	Bacteroidia	Bacteroidales	Bacteroidales_S24-7_g	norank_f__Bacteroidales_S24-7_grou	unclassified_g__norank_f__Bacteroidales_S24-7_group	OTU184	0	0	0	0
Firmicutes	Clostridia	Clostridiales	Ruminococcaceae	Ruminococcaceae_UCG-014	unclassified_g__Ruminococcaceae_UCG-014	OTU524	1	0	6	1
Firmicutes	Clostridia	Clostridiales	Peptococcaceae	norank_f__Peptococcaceae	uncultured_bacterium_g__norank_f__Peptococcaceae	OTU115	16	3	5	0
Firmicutes	Clostridia	Clostridiales	Lachnospiraceae	unclassified_f__Lachnospiraceae	unclassified_f__Lachnospiraceae	OTU127	6	0	0	2
Firmicutes	Clostridia	Clostridiales	Lachnospiraceae	unclassified_f__Lachnospiraceae	unclassified_f__Lachnospiraceae	OTU664	2	1	5	3
Firmicutes	Clostridia	Clostridiales	Lachnospiraceae	unclassified_f__Lachnospiraceae	unclassified_f__Lachnospiraceae	OTU465	1	0	2	2
Firmicutes	Clostridia	Clostridiales	Ruminococcaceae	Ruminococcaceae_UCG-005	uncultured_Clostridiales_bacterium_g__Ruminococcaceae_UCG-005	OTU689	2	7	8	1
Tenericutes	Mollicutes	Mollicutes_RF9	norank_o__Mollicutes	norank_o__Mollicutes_RF9	uncultured_bacterium_g__norank_o__Mollicutes_RF9	OTU52	3	8	0	0
Firmicutes	Clostridia	Clostridiales	Ruminococcaceae	[Eubacterium]_coprostanoligenes_g	unclassified_g__[Eubacterium]_coprostanoligenes_group	OTU74	3	3	1	0
Firmicutes	Clostridia	Clostridiales	Lachnospiraceae	Coprococcus_1	unclassified_g__Coprococcus_1	OTU659	0	0	3	4
Firmicutes	Clostridia	Clostridiales	Lachnospiraceae	unclassified_f__Lachnospiraceae	unclassified_f__Lachnospiraceae	OTU688	3	2	3	2
Firmicutes	Clostridia	Clostridiales	Ruminococcaceae	unclassified_f__Ruminococcaceae	unclassified_f__Ruminococcaceae	OTU732	2	0	1	2
Firmicutes	Clostridia	Clostridiales	Christensenellaceae	Christensenellaceae_R-7_group	uncultured_organism_g__Christensenellaceae_R-7_group	OTU684	2	2	4	3
Bacteroidetes	Bacteroidia	Bacteroidales	Porphyromonadaceae	Odoribacter	uncultured_bacterium_g__Odoribacter	OTU327	0	0	0	0
Firmicutes	Clostridia	Clostridiales	Clostridiales_vadinBB6	norank_f__Clostridiales_vadinBB60_	uncultured_bacterium_g__norank_f__Clostridiales_vadinBB60_group	OTU325	4	0	0	0
Firmicutes	Clostridia	Clostridiales	Lachnospiraceae	unclassified_f__Lachnospiraceae	unclassified_f__Lachnospiraceae	OTU551	2	2	0	0
Firmicutes	Bacilli	Lactobacillales	Enterococcaceae	Enterococcus	unclassified_g__Enterococcus	OTU237	0	12	0	0
Bacteroidetes	Bacteroidia	Bacteroidales	Prevotellaceae	Alloprevotella	gut_metagenome_g__Alloprevotella	OTU312	0	0	0	0
Firmicutes	Clostridia	Clostridiales	Ruminococcaceae	unclassified_f__Ruminococcaceae	unclassified_f__Ruminococcaceae	OTU855	0	0	2	1
Actinobacteria	Actinobacteria	Bifidobacteriales	Bifidobacteriaceae	Bifidobacterium	Bifidobacterium_pseudolongum_PV8-2	OTU468	0	0	0	0
Firmicutes	Clostridia	Clostridiales	Christensenellaceae	norank_f__Christensenellaceae	unclassified_g__norank_f__Christensenellaceae	OTU529	2	2	0	3
Tenericutes	Mollicutes	Mollicutes_RF9	norank_o__Mollicutes	norank_o__Mollicutes_RF9	unclassified_g__norank_o__Mollicutes_RF9	OTU764	0	0	0	7
Firmicutes	Clostridia	Clostridiales	Ruminococcaceae	norank_f__Ruminococcaceae	unclassified_g__norank_f__Ruminococcaceae	OTU805	2	0	6	0
Firmicutes	Clostridia	Clostridiales	Ruminococcaceae	Faecalibacterium	uncultured_bacterium_g__Faecalibacterium	OTU60	0	1	5	0
Firmicutes	Clostridia	Clostridiales	Ruminococcaceae	unclassified_f__Ruminococcaceae	unclassified_f__Ruminococcaceae	OTU530	2	5	0	1
Firmicutes	Clostridia	Clostridiales	Peptococcaceae	norank_f__Peptococcaceae	uncultured_bacterium_g__norank_f__Peptococcaceae	OTU619	11	2	2	0
Firmicutes	Clostridia	Clostridiales	Ruminococcaceae	unclassified_f__Ruminococcaceae	unclassified_f__Ruminococcaceae	OTU471	0	8	0	1
Firmicutes	Clostridia	Clostridiales	Ruminococcaceae	Candidatus_Soleaferrea	uncultured_bacterium_g__Candidatus_Soleaferrea	OTU284	5	1	0	2
Firmicutes	Clostridia	Clostridiales	Ruminococcaceae	Anaerotruncus	unclassified_g__Anaerotruncus	OTU508	0	0	0	1
Firmicutes	Clostridia	Clostridiales	Ruminococcaceae	Anaerotruncus	uncultured_bacterium_g__Anaerotruncus	OTU704	3	0	2	1
Tenericutes	Mollicutes	Mollicutes_RF9	norank_o__Mollicutes	norank_o__Mollicutes_RF9	uncultured_bacterium_g__norank_o__Mollicutes_RF9	OTU783	51	0	0	0
Firmicutes	Clostridia	Clostridiales	Eubacteriaceae	Anaerofustis	uncultured_bacterium_g__Anaerofustis	OTU111	2	0	0	0
Firmicutes	Clostridia	Clostridiales	Clostridiales_vadinBB6	norank_f__Clostridiales_vadinBB60_	unclassified_g__norank_f__Clostridiales_vadinBB60_group	OTU645	2	0	11	0
Firmicutes	Clostridia	Clostridiales	Peptococcaceae	norank_f__Peptococcaceae	uncultured_bacterium_g__norank_f__Peptococcaceae	OTU27	4	0	3	0
Firmicutes	Clostridia	Clostridiales	Ruminococcaceae	Anaerotruncus	unclassified_g__Anaerotruncus	OTU809	4	2	1	0
Bacteroidetes	Bacteroidia	Bacteroidales	Rikenellaceae	Rikenellaceae_RC9_gut_group	unclassified_g__Rikenellaceae_RC9_gut_group	OTU321	0	0	0	0
Firmicutes	Clostridia	Clostridiales	Ruminococcaceae	Ruminococcaceae_UCG-005	unclassified_g__Ruminococcaceae_UCG-005	OTU482	0	3	2	3
Tenericutes	Mollicutes	Mollicutes_RF9	norank_o__Mollicutes	norank_o__Mollicutes_RF9	uncultured_Erysipelotrichaceae_bacterium_g__norank_o__Mollicutes_RF9	OTU558	1	0	4	5
Firmicutes	Clostridia	Clostridiales	Clostridiaceae_1	Candidatus_Arthromitus	Candidatus_Arthromitus_sp._SFB-mouse-Japan	OTU162	1	3	2	0
Firmicutes	Clostridia	Clostridiales	Ruminococcaceae	Subdoligranulum	unclassified_g__Subdoligranulum	OTU810	0	0	0	0
Firmicutes	Clostridia	Clostridiales	Ruminococcaceae	norank_f__Ruminococcaceae	unclassified_g__norank_f__Ruminococcaceae	OTU666	0	1	0	0
Firmicutes	Clostridia	Clostridiales	Ruminococcaceae	unclassified_f__Ruminococcaceae	unclassified_f__Ruminococcaceae	OTU693	2	0	1	0
Firmicutes	Clostridia	Clostridiales	Ruminococcaceae	unclassified_f__Ruminococcaceae	unclassified_f__Ruminococcaceae	OTU858	3	0	3	2
Firmicutes	Clostridia	Clostridiales	Ruminococcaceae	Ruminiclostridium_5	unclassified_g__Ruminiclostridium_5	OTU470	0	2	0	1
Firmicutes	Negativicutes	Selenomonadales	Acidaminococcaceae	Phascolarctobacterium	uncultured_organism_g__Phascolarctobacterium	OTU787	2	0	0	0
Firmicutes	Clostridia	Clostridiales	Christensenellaceae	Christensenellaceae_R-7_group	unclassified_g__Christensenellaceae_R-7_group	OTU745	0	0	0	9
Firmicutes	Clostridia	Clostridiales	Peptococcaceae	norank_f__Peptococcaceae	unclassified_g__norank_f__Peptococcaceae	OTU495	0	0	0	1
Firmicutes	Clostridia	Clostridiales	Ruminococcaceae	Ruminococcaceae_UCG-010	uncultured_bacterium_g__Ruminococcaceae_UCG-010	OTU18	0	4	2	0
Firmicutes	Clostridia	Clostridiales	Ruminococcaceae	Ruminococcaceae_UCG-013	unclassified_g__Ruminococcaceae_UCG-013	OTU89	6	0	0	0
Firmicutes	Clostridia	Clostridiales	Ruminococcaceae	Ruminiclostridium_5	unclassified_g__Ruminiclostridium_5	OTU649	2	0	0	1
Firmicutes	Clostridia	Clostridiales	Lachnospiraceae	unclassified_f__Lachnospiraceae	unclassified_f__Lachnospiraceae	OTU324	1	0	0	2
Firmicutes	Clostridia	Clostridiales	Ruminococcaceae	Ruminococcaceae_UCG-013	unclassified_g__Ruminococcaceae_UCG-013	OTU31	3	6	2	0
Firmicutes	Clostridia	Clostridiales	Ruminococcaceae	Ruminococcus_1	uncultured_organism_g__Ruminococcus_1	OTU651	0	0	0	0
Firmicutes	Clostridia	Clostridiales	Peptococcaceae	unclassified_f__Peptococcaceae	unclassified_f__Peptococcaceae	OTU768	1	2	0	1
Firmicutes	Clostridia	Clostridiales	Christensenellaceae	unclassified_f__Christensenellaceae	unclassified_f__Christensenellaceae	OTU857	0	0	7	0
Tenericutes	Mollicutes	Mollicutes_RF9	norank_o__Mollicutes	norank_o__Mollicutes_RF9	unclassified_g__norank_o__Mollicutes_RF9	OTU758	5	0	0	3
Firmicutes	Clostridia	Clostridiales	Ruminococcaceae	Candidatus_Soleaferrea	uncultured_bacterium_g__Candidatus_Soleaferrea	OTU791	3	1	1	5
unclassified_k__r	unclassified_k__norank	unclassified_k__nor	unclassified_k__noran	unclassified_k__norank	unclassified_k__norank	OTU9	0	2	5	1
Firmicutes	Clostridia	Clostridiales	Ruminococcaceae	unclassified_f__Ruminococcaceae	unclassified_f__Ruminococcaceae	OTU733	1	1	0	0
Firmicutes	Clostridia	Clostridiales	Peptococcaceae	norank_f__Peptococcaceae	unclassified_g__norank_f__Peptococcaceae	OTU90	6	1	3	2
Firmicutes	Clostridia	Clostridiales	Lachnospiraceae	unclassified_f__Lachnospiraceae	unclassified_f__Lachnospiraceae	OTU644	2	1	0	2
Firmicutes	Clostridia	Clostridiales	Ruminococcaceae	[Eubacterium]_coprostanoligenes_g	unclassified_g__[Eubacterium]_coprostanoligenes_group	OTU29	1	0	3	0
Firmicutes	Clostridia	Clostridiales	Ruminococcaceae	Ruminococcaceae_UCG-013	uncultured_Clostridiaceae_bacterium_g__Ruminococcaceae_UCG-013	OTU152	1	17	8	0
Firmicutes	Clostridia	Clostridiales	Ruminococcaceae	Faecalibacterium	unclassified_g__Faecalibacterium	OTU243	0	0	1	0
Bacteroidetes	Bacteroidia	Bacteroidales	Bacteroidales_S24-7_g	norank_f__Bacteroidales_S24-7_grou	uncultured_Bacteroidales_bacterium_g__norank_f__Bacteroidales_S24-7_group	OTU339	0	0	0	0
Tenericutes	Mollicutes	Mollicutes_RF9	norank_o__Mollicutes	norank_o__Mollicutes_RF9	unclassified_g__norank_o__Mollicutes_RF9	OTU51	11	5	1	3
Firmicutes	Clostridia	Clostridiales	Christensenellaceae	Christensenellaceae_R-7_group	uncultured_organism_g__Christensenellaceae_R-7_group	OTU332	4	0	0	0
Firmicutes	Clostridia	Clostridiales	Ruminococcaceae	Anaerotruncus	unclassified_g__Anaerotruncus	OTU194	0	0	0	0
Firmicutes	Clostridia	Clostridiales	Ruminococcaceae	Ruminococcaceae_UCG-013	uncultured_bacterium_g__Ruminococcaceae_UCG-013	OTU334	0	0	1	0
Firmicutes	Clostridia	Clostridiales	Ruminococcaceae	Ruminococcaceae_NK4A214_group	unclassified_g__Ruminococcaceae_NK4A214_group	OTU32	3	1	2	2
Firmicutes	Clostridia	Clostridiales	Christensenellaceae	Christensenellaceae_R-7_group	unclassified_g__Christensenellaceae_R-7_group	OTU105	4	0	0	1
Firmicutes	Bacilli	Bacillales	Staphylococcaceae	Staphylococcus	Staphylococcus_warneri	OTU597	0	0	0	0
Firmicutes	Bacilli	Bacillales	Staphylococcaceae	Staphylococcus	Staphylococcus_lentus_g__Staphylococcus	OTU432	0	0	0	0
Firmicutes	Clostridia	Clostridiales	Ruminococcaceae	unclassified_f__Ruminococcaceae	unclassified_f__Ruminococcaceae	OTU698	9	0	0	0
Tenericutes	Mollicutes	Mollicutes_RF9	norank_o__Mollicutes	norank_o__Mollicutes_RF9	unclassified_g__norank_o__Mollicutes_RF9	OTU781	0	0	0	0

Firmicutes	Clostridia	Clostridiales	Ruminococcaceae	Anaerotruncus	uncultured_bacterium_g__Anaerotruncus	OTU53	2	4	0	0
Actinobacteria	Actinobacteria	Micrococcales	Intrasporangiaceae	Terrabacter	uncultured_bacterium_g__Terrabacter	OTU270	0	0	0	0
Firmicutes	Clostridia	Clostridiales	Ruminococcaceae	unclassified_f__Ruminococcaceae	unclassified_f__Ruminococcaceae	OTU681	6	0	1	0
Bacteroidetes	Bacteroidia	Bacteroidales	Bacteroidales_S24-7_g	norank_f__Bacteroidales_S24-7_gro	uncultured_bacterium_g__norank_f__Bacteroidales_S24-7_group	OTU310	0	0	0	0
Firmicutes	Clostridia	Clostridiales	Clostridiales_vadinBB6	norank_f__Clostridiales_vadinBB60_	unclassified_g__norank_f__Clostridiales_vadinBB60_group	OTU528	0	0	0	0
Firmicutes	Clostridia	Clostridiales	Ruminococcaceae	Ruminiclostridium	uncultured_bacterium_g__Ruminiclostridium	OTU633	0	0	0	0
Firmicutes	Clostridia	Clostridiales	Christensenellaceae	Christensenellaceae_R-7_group	unclassified_g__Christensenellaceae_R-7_group	OTU695	1	2	1	0
Cyanobacteria	Cyanobacteria	norank_c__Cyanoba	norank_c__Cyanobact	norank_c__Cyanobacteria	unclassified_g__norank_c__Cyanobacteria	OTU258	0	0	0	0
Bacteroidetes	Bacteroidia	Bacteroidales	Prevotellaceae	Prevotellaceae_UCG-001	uncultured_Bacteroidales_bacterium_g__Prevotellaceae_UCG-001	OTU276	0	0	0	0
Firmicutes	Bacilli	Lactobacillales	Lactobacillaceae	Lactobacillus	unclassified_g__Lactobacillus	OTU632	0	0	2	0
Firmicutes	Clostridia	Clostridiales	Ruminococcaceae	Ruminiclostridium_9	Clostridiales_bacterium_NK3B98_g__Ruminiclostridium_9	OTU130	2	0	0	0
Firmicutes	Clostridia	Clostridiales	Ruminococcaceae	Ruminococcaceae_UCG-013	uncultured_bacterium_g__Ruminococcaceae_UCG-013	OTU807	1	0	0	0
Bacteroidetes	Bacteroidia	Bacteroidales	Rikenellaceae	Alistipes	gut_metagenome_g__Alistipes	OTU808	0	2	1	0
Firmicutes	Clostridia	unclassified_c__Clo	unclassified_c__Clostr	unclassified_c__Clostridia	unclassified_c__Clostridia	OTU99	5	1	1	0
Firmicutes	Clostridia	Clostridiales	Christensenellaceae	norank_f__Christensenellaceae	unclassified_g__norank_f__Christensenellaceae	OTU553	0	1	0	0
Firmicutes	Clostridia	Clostridiales	Lachnospiraceae	unclassified_f__Lachnospiraceae	unclassified_f__Lachnospiraceae	OTU80	0	0	2	0
Bacteroidetes	Bacteroidia	Bacteroidales	Prevotellaceae	Paraprevotella	unclassified_g__Paraprevotella	OTU308	0	0	0	0
Firmicutes	Clostridia	Clostridiales	Peptococcaceae	norank_f__Peptococcaceae	unclassified_g__norank_f__Peptococcaceae	OTU596	2	0	0	1
Firmicutes	Clostridia	Clostridiales	Lachnospiraceae	Lachnospiraceae_NK4A136_group	unclassified_g__Lachnospiraceae_NK4A136_group	OTU251	0	0	0	0
Firmicutes	Clostridia	Clostridiales	Family_XIII	[Eubacterium]_brachy_group	uncultured_bacterium_g__[Eubacterium]_brachy_group	OTU335	0	0	0	0
Firmicutes	Clostridia	Clostridiales	Lachnospiraceae	unclassified_f__Lachnospiraceae	unclassified_f__Lachnospiraceae	OTU86	0	0	0	0
Firmicutes	Clostridia	Clostridiales	Ruminococcaceae	Ruminococcaceae_UCG-005	uncultured_bacterium_adhufec311	OTU215	0	0	1	0
Firmicutes	Clostridia	Clostridiales	Ruminococcaceae	Ruminococcaceae_UCG-009	uncultured_bacterium_g__Ruminococcaceae_UCG-009	OTU641	8	0	1	1
Firmicutes	Clostridia	Clostridiales	Ruminococcaceae	Ruminiclostridium_1	unclassified_g__Ruminiclostridium_1	OTU178	0	0	5	0
Actinobacteria	Actinobacteria	Micrococcales	Micrococcaceae	Pseudarthrobacter	unclassified_g__Pseudarthrobacter	OTU423	0	1	0	0
Firmicutes	Clostridia	Clostridiales	Ruminococcaceae	Ruminococcaceae_UCG-010	unclassified_g__Ruminococcaceae_UCG-010	OTU26	1	1	0	0
Firmicutes	Clostridia	Clostridiales	Clostridiaceae_1	Clostridium_sensu_stricto_1	uncultured_bacterium_g__Clostridium_sensu_stricto_1	OTU128	0	0	0	0
Firmicutes	Clostridia	Clostridiales	Ruminococcaceae	Anaerotruncus	uncultured_bacterium_g__Anaerotruncus	OTU245	0	2	1	0
Firmicutes	Clostridia	Clostridiales	Ruminococcaceae	Ruminococcaceae_UCG-014	unclassified_g__Ruminococcaceae_UCG-014	OTU429	0	0	0	0
Firmicutes	Erysipelotrichia	Erysipelotrichales	Erysipelotrichaceae	Faecalibaculum	uncultured_bacterium_g__Faecalibaculum	OTU497	0	0	0	0
Firmicutes	Clostridia	Clostridiales	Ruminococcaceae	unclassified_f__Ruminococcaceae	unclassified_f__Ruminococcaceae	OTU525	0	0	0	0
Firmicutes	Clostridia	Clostridiales	Ruminococcaceae	Anaerotruncus	unclassified_g__Anaerotruncus	OTU640	0	0	0	0
Actinobacteria	Actinobacteria	Coriobacteriales	Coriobacteriaceae	Coriobacteriaceae_UCG-002	uncultured_bacterium_g__Coriobacteriaceae_UCG-002	OTU365	0	0	0	0
Firmicutes	Clostridia	Clostridiales	Christensenellaceae	Christensenellaceae_R-7_group	unclassified_g__Christensenellaceae_R-7_group	OTU670	2	1	0	0
Firmicutes	Clostridia	Clostridiales	Ruminococcaceae	Ruminococcaceae_UCG-010	uncultured_bacterium_g__Ruminococcaceae_UCG-010	OTU776	0	0	0	0
Firmicutes	Clostridia	Clostridiales	Christensenellaceae	norank_f__Christensenellaceae	uncultured_bacterium_g__norank_f__Christensenellaceae	OTU139	3	1	1	1
Firmicutes	Clostridia	Clostridiales	Christensenellaceae	Christensenellaceae_R-7_group	uncultured_bacterium_g__Christensenellaceae_R-7_group	OTU207	0	0	0	0
Actinobacteria	Actinobacteria	Coriobacteriales	Coriobacteriaceae	unclassified_f__Coriobacteriaceae	unclassified_f__Coriobacteriaceae	OTU477	0	0	1	0
Firmicutes	Clostridia	Clostridiales	Ruminococcaceae	unclassified_f__Ruminococcaceae	unclassified_f__Ruminococcaceae	OTU176	1	3	1	1
Bacteroidetes	Bacteroidia	Bacteroidales	Bacteroidales_S24-7_g	norank_f__Bacteroidales_S24-7_gro	uncultured_bacterium_g__norank_f__Bacteroidales_S24-7_group	OTU262	0	0	0	0
Firmicutes	Clostridia	Clostridiales	Ruminococcaceae	Anaerotruncus	unclassified_g__Anaerotruncus	OTU444	0	0	0	0
Firmicutes	Clostridia	Clostridiales	Ruminococcaceae	Ruminococcaceae_UCG-013	uncultured_Clostridiales_bacterium_g__Ruminococcaceae_UCG-013	OTU522	0	0	0	7
Firmicutes	Clostridia	Clostridiales	Ruminococcaceae	Ruminococcaceae_UCG-010	uncultured_organism_g__Ruminococcaceae_UCG-010	OTU782	0	3	0	0
Saccharibacteria	Unknown_Class_p__Sacc	Unknown_Order_c_	Unknown_Family_o__	Candidatus_Saccharimonas	uncultured_bacterium_g__Candidatus_Saccharimonas	OTU261	0	0	0	0
Firmicutes	Clostridia	Clostridiales	Lachnospiraceae	unclassified_f__Lachnospiraceae	unclassified_f__Lachnospiraceae	OTU120	0	0	1	0
Chloroflexi	KD4-96	norank_c__KD4-96	norank_c__KD4-96	norank_c__KD4-96	unclassified_g__norank_c__KD4-96	OTU254	0	0	0	0
Firmicutes	Clostridia	Clostridiales	Clostridiales_vadinBB6	norank_f__Clostridiales_vadinBB60_	unclassified_g__norank_f__Clostridiales_vadinBB60_group	OTU209	1	2	0	1
Tenericutes	Mollicutes	Mollicutes_RF9	norank_o__Mollicutes	norank_o__Mollicutes_RF9	unclassified_g__norank_o__Mollicutes_RF9	OTU572	0	0	0	0
Firmicutes	Clostridia	Clostridiales	Ruminococcaceae	unclassified_f__Ruminococcaceae	unclassified_f__Ruminococcaceae	OTU195	1	0	0	1
Proteobacteria	Alphaproteobacteria	Sphingomonadales	Sphingomonadaceae	Sphingomonas	uncultured_Sphingomonadaceae_bacterium_g__Sphingomonas	OTU375	0	0	0	0
Tenericutes	Mollicutes	Mollicutes_RF9	norank_o__Mollicutes	norank_o__Mollicutes_RF9	unclassified_g__norank_o__Mollicutes_RF9	OTU727	1	2	1	0
Firmicutes	Erysipelotrichia	Erysipelotrichales	Erysipelotrichaceae	Turicibacter	uncultured_bacterium_g__Turicibacter	OTU40	0	0	0	0
Firmicutes	Clostridia	Clostridiales	Lachnospiraceae	Coprococcus_1	intestinal_bacterium_CG19-1	OTU45	0	1	2	0
Firmicutes	Clostridia	Clostridiales	Peptococcaceae	norank_f__Peptococcaceae	unclassified_g__norank_f__Peptococcaceae	OTU116	10	0	0	0
Firmicutes	Clostridia	Clostridiales	Peptococcaceae	norank_f__Peptococcaceae	unclassified_g__norank_f__Peptococcaceae	OTU199	3	0	0	1
Bacteroidetes	Bacteroidia	Bacteroidales	Bacteroidaceae	Bacteroides	Bacteroides_acidifaciens	OTU320	0	0	0	0
Firmicutes	Clostridia	Clostridiales	Ruminococcaceae	unclassified_f__Ruminococcaceae	unclassified_f__Ruminococcaceae	OTU552	0	3	0	0
Firmicutes	Clostridia	Clostridiales	Lachnospiraceae	Butyrivibrio	uncultured_organism_g__Butyrivibrio	OTU762	1	0	0	0
Tenericutes	Mollicutes	Mollicutes_RF9	norank_o__Mollicutes	norank_o__Mollicutes_RF9	uncultured_bacterium_g__norank_o__Mollicutes_RF9	OTU797	0	0	0	0
Firmicutes	Clostridia	Clostridiales	Lachnospiraceae	unclassified_f__Lachnospiraceae	unclassified_f__Lachnospiraceae	OTU852	0	0	1	0
Proteobacteria	Alphaproteobacteria	Rhizobiales	Bradyrhizobiaceae	Bradyrhizobium	Bradyrhizobium_elkanii	OTU185	0	0	0	0
Chloroflexi	Chloroflexia	Chloroflexales	Roseiflexaceae	Roseiflexus	uncultured_bacterium_g__Roseiflexus	OTU415	0	0	0	0
Proteobacteria	Alphaproteobacteria	Rhizobiales	Xanthobacteraceae	norank_f__Xanthobacteraceae	uncultured_bacterium_g__norank_f__Xanthobacteraceae	OTU427	0	0	0	0
Firmicutes	unclassified_p__Firmicut	unclassified_p__Firi	unclassified_p__Firmi	unclassified_p__Firmicutes	unclassified_p__Firmicutes	OTU454	0	0	0	0
Firmicutes	Clostridia	Clostridiales	Ruminococcaceae	Anaerotruncus	unclassified_g__Anaerotruncus	OTU621	0	0	2	0
Firmicutes	Clostridia	Clostridiales	Ruminococcaceae	Anaerotruncus	uncultured_Clostridiales_bacterium_g__Anaerotruncus	OTU736	1	1	0	0
Firmicutes	Clostridia	Clostridiales	Ruminococcaceae	Hydrogenoanaerobacterium	Selenomonadales_bacterium_Marseille-P2399	OTU5	0	0	0	0
Bacteroidetes	Bacteroidia	Bacteroidales	Bacteroidales_S24-7_g	norank_f__Bacteroidales_S24-7_gro	uncultured_bacterium_g__norank_f__Bacteroidales_S24-7_group	OTU300	0	0	0	0
Bacteroidetes	Bacteroidia	Bacteroidales	Prevotellaceae	Prevotellaceae_NK3B31_group	uncultured_bacterium_g__Prevotellaceae_NK3B31_group	OTU331	0	0	0	0
Proteobacteria	Betaproteobacteria	Burkholderiales	Burkholderiaceae	Ralstonia	unidentified_marine_bacterioplankton_g__Ralstonia	OTU593	0	2	0	0
Tenericutes	Mollicutes	Mollicutes_RF9	norank_o__Mollicutes	norank_o__Mollicutes_RF9	uncultured_Erysipelotrichia_bacterium_g__norank	OTU594	0	0	0	0
Firmicutes	Clostridia	Clostridiales	Ruminococcaceae	Anaerotruncus	unclassified_g__Anaerotruncus	OTU713	0	1	1	1
Firmicutes	Clostridia	Clostridiales	Clostridiales_vadinBB6	norank_f__Clostridiales_vadinBB60_	uncultured_bacterium_g__norank_f__Clostridiales_vadinBB60_group	OTU724	0	0	0	0
Firmicutes	Clostridia	Clostridiales	Christensenellaceae	unclassified_f__Christensenellaceae	unclassified_f__Christensenellaceae	OTU793	1	1	0	0
Firmicutes	Clostridia	Clostridiales	Lachnospiraceae	Tyzzerella	unclassified_g__Tyzzerella	OTU796	0	1	0	0
Actinobacteria	Actinobacteria	Bifidobacteriales	Bifidobacteriaceae	Bifidobacterium	Bifidobacterium_pseudocatenulatum_DSM_20438__JCM_1200__LMG_10505	OTU55	0	1	0	0
Firmicutes	Clostridia	Clostridiales	Clostridiales_vadinBB6	norank_f__Clostridiales_vadinBB60_	uncultured_bacterium_g__norank_f__Clostridiales_vadinBB60_group	OTU336	0	0	0	0
Bacteroidetes	Bacteroidia	Bacteroidales	Bacteroidaceae	Bacteroides	Bacteroides_sartorii	OTU345	0	0	0	0
Firmicutes	Clostridia	Clostridiales	Ruminococcaceae	Ruminiclostridium_5	unclassified_g__Ruminiclostridium_5	OTU818	1	0	0	0
Firmicutes	Clostridia	Clostridiales	Christensenellaceae	norank_f__Christensenellaceae	uncultured_bacterium_g__norank_f__Christensenellaceae	OTU144	1	2	0	3
Firmicutes	Clostridia	Clostridiales	Ruminococcaceae	[Eubacterium]_coprostanoligenes_g	unclassified_g__[Eubacterium]_coprostanoligenes_group	OTU212	0	0	1	0
Firmicutes	Clostridia	Clostridiales	Ruminococcaceae	Ruminococcaceae_UCG-013	unclassified_g__Ruminococcaceae_UCG-013	OTU342	0	0	0	4
Chloroflexi	Chloroflexia	Chloroflexales	Roseiflexaceae	Roseiflexus	uncultured_bacterium_g__Roseiflexus	OTU433	0	0	0	0
Firmicutes	Clostridia	Clostridiales	Ruminococcaceae	unclassified_f__Ruminococcaceae	unclassified_f__Ruminococcaceae	OTU646	0	2	0	0
Firmicutes	Bacilli	Lactobacillales	Lactobacillaceae	Lactobacillus	Lactobacillus_saerimneri	OTU719	0	0	0	0
Firmicutes	Clostridia	Clostridiales	Ruminococcaceae	Ruminococcus_1	uncultured_organism_g__Ruminococcus_1	OTU227	0	0	0	0
Deferribacteres	Deferribacteres	Deferribacterales	Deferribacteraceae	Mucispirillum	uncultured_bacterium_g__Mucispirillum	OTU272	0	0	0	0
Chloroflexi	Chloroflexia	Chloroflexales	Roseiflexaceae	Roseiflexus	uncultured_bacterium_g__Roseiflexus	OTU277	0	0	0	0
Actinobacteria	Actinobacteria	Solirubrobacterales	Solirubrobacteraceae	Solirubrobacter	uncultured_bacterium_g__Solirubrobacter	OTU414	0	0	0	0
Firmicutes	Clostridia	Clostridiales	Ruminococcaceae	Anaerotruncus	unclassified_g__Anaerotruncus	OTU546	2	0	0	0
Tenericutes	Mollicutes	Mollicutes_RF9	norank_o__Mollicutes	norank_o__Mollicutes_RF9	uncultured_bacterium_g__norank_o__Mollicutes_RF9	OTU571	2	0	0	0
Firmicutes	Clostridia	Clostridiales	Ruminococcaceae	Ruminiclostridium_5	uncultured_bacterium_g__Ruminiclostridium_5	OTU806	0	2	0	0
Firmicutes	Clostridia	Clostridiales	Ruminococcaceae	Ruminococcaceae_UCG-013	uncultured_bacterium_g__Ruminococcaceae_UCG-013	OTU108	3	0	0	0
Firmicutes	Clostridia	Clostridiales	Peptococcaceae	norank_f__Peptococcaceae	uncultured_bacterium_g__norank_f__Peptococcaceae	OTU112	1	0	0	0
Acidobacteria	Acidobacteria	Blastocatellales	Blastocatellaceae__Su	RB41	unclassified_g__RB41	OTU374	0	0	0	0
Actinobacteria	Actinobacteria	Propionibacteriales	Nocardioidaceae	Marmoricola	uncultured_bacterium_g__Marmoricola	OTU395	0	0	0	0
Firmicutes	Clostridia	Clostridiales	Ruminococcaceae	unclassified_f__Ruminococcaceae	unclassified_f__Ruminococcaceae	OTU650	8	0	0	0
Firmicutes	Clostridia	Clostridiales	Ruminococcaceae	Ruminiclostridium_9	unclassified_g__Ruminiclostridium_9	OTU92	0	0	0	0
Actinobacteria	Actinobacteria	Streptomycetales	Streptomycetaceae	Streptomyces	unclassified_g__Streptomyces	OTU358	0	0	0	0
Proteobacteria	Deltaproteobacteria	Desulfovibrionales	Desulfovibrionaceae	Desulfovibrio	unclassified_g__Desulfovibrio	OTU431	0	0	0	0
Firmicutes	Clostridia	Clostridiales	Lachnospiraceae	unclassified_f__Lachnospiraceae	unclassified_f__Lachnospiraceae	OTU668	0	0	0	1
Firmicutes	Clostridia	Clostridiales	Syntrophomonadacea	Syntrophomonas	unclassified_g__Syntrophomonas	OTU790	2	0	0	0
Bacteroidetes	Bacteroidia	Bacteroidales	Bacteroidales_S24-7_g	norank_f__Bacteroidales_S24-7_gro	uncultured_bacterium_g__norank_f__Bacteroidales_S24-7_group	OTU369	0	0	0	0
Firmicutes	Clostridia	Clostridiales	Peptostreptococcacea	Peptoclostridium	Peptoclostridium_difficile_g__Peptoclostridium	OTU459	0	0	0	0
Firmicutes	Erysipelotrichia	Erysipelotrichales	Erysipelotrichaceae	[Clostridium]_innocuum_group	uncultured_bacterium_g__[Clostridium]_innocuum_group	OTU503	1	0	0	0
Firmicutes	Clostridia	Clostridiales	Ruminococcaceae	Ruminococcaceae_UCG-009	uncultured_bacterium_g__Ruminococcaceae_UCG-009	OTU686	1	0	0	1
Firmicutes	Clostridia	Clostridiales	Ruminococcaceae	Anaerotruncus	unclassified_g__Anaerotruncus	OTU753	0	1	1	0
Firmicutes	Clostridia	Clostridiales	Ruminococcaceae	Ruminiclostridium	uncultured_organism_g__Ruminiclostridium	OTU829	0	5	0	1
Bacteroidetes	Bacteroidia	Bacteroidales	Bacteroidales_S24-7_g	norank_f__Bacteroidales_S24-7_gro	uncultured_bacterium_g__norank_f__Bacteroidales_S24-7_group	OTU304	0	0	0	0
Bacteroidetes	Bacteroidia	Bacteroidales	Bacteroidales_S24-7_g	norank_f__Bacteroidales_S24-7_gro	uncultured_bacterium_g__norank_f__Bacteroidales_S24-7_group	OTU350	0	0	0	0
Bacteroidetes	Bacteroidia	Bacteroidales	Bacteroidales_S24-7_g	norank_f__Bacteroidales_S24-7_gro	uncultured_bacterium_g__norank_f__Bacteroidales_S24-7_group	OTU380	0	0	0	1

Firmicutes	Clostridia	Clostridiales	Ruminococcaceae	Candidatus_Soleaferrea	Ruminococcaceae_bacterium_GD6	OTU456	2	0	0	0
Firmicutes	Clostridia	Clostridiales	Ruminococcaceae	unclassified_f__Ruminococcaceae	unclassified_f__Ruminococcaceae	OTU545	0	1	0	0
Firmicutes	Clostridia	Clostridiales	Ruminococcaceae	Ruminococcaceae_UCG-009	uncultured_bacterium_g__Ruminococcaceae_UCG-009	OTU595	0	5	0	0
Firmicutes	Clostridia	Clostridiales	Ruminococcaceae	unclassified_f__Ruminococcaceae	unclassified_f__Ruminococcaceae	OTU844	0	0	0	0
Firmicutes	Clostridia	Clostridiales	Christensenellaceae	norank_f__Christensenellaceae	uncultured_bacterium_g__norank_f__Christensenellaceae	OTU126	0	1	1	0
Firmicutes	Clostridia	Clostridiales	Clostridiales_vadinBB6	norank_f__Clostridiales_vadinBB60_	uncultured_bacterium_g__norank_f__Clostridiales_vadinBB60_group	OTU161	0	1	0	0
Bacteroidetes	Bacteroidia	Bacteroidales	Bacteroidales_S24-7_g	norank_f__Bacteroidales_S24-7_gro	unclassified_g__norank_f__Bacteroidales_S24-7_group	OTU267	0	0	1	1
Actinobacteria	Actinobacteria	Streptosporangiales	Thermomonosporacea	Actinomadura	Actinomadura_hallensis	OTU291	0	0	0	0
Firmicutes	Clostridia	Clostridiales	Ruminococcaceae	Ruminiclostridium_5	unclassified_g__Ruminiclostridium_5	OTU341	0	0	0	0
Actinobacteria	Actinobacteria	Corynebacteriales	Mycobacteriaceae	Mycobacterium	unclassified_g__Mycobacterium	OTU381	0	0	0	0
Firmicutes	Clostridia	Clostridiales	Ruminococcaceae	Ruminococcus_1	unclassified_g__Ruminococcus_1	OTU523	0	0	0	0
Bacteroidetes	Bacteroidia	Bacteroidales	Rikenellaceae	Alistipes	Alistipes_sp._CHKCI003	OTU15	0	1	0	0
Firmicutes	Clostridia	Clostridiales	Ruminococcaceae	Ruminococcaceae_UCG-010	unclassified_g__Ruminococcaceae_UCG-010	OTU222	0	0	0	0
Firmicutes	Clostridia	Clostridiales	Clostridiales_vadinBB6	norank_f__Clostridiales_vadinBB60_	uncultured_prokaryote_g__norank_f__Clostridiales_vadinBB60_group	OTU247	0	0	0	0
Bacteroidetes	Bacteroidia	Bacteroidales	Bacteroidales_S24-7_g	norank_f__Bacteroidales_S24-7_gro	uncultured_bacterium_g__norank_f__Bacteroidales_S24-7_group	OTU253	0	0	0	0
Verrucomicrobia	Spartobacteria	Chthoniobacterales	DA101_soil_group	norank_f__DA101_soil_group	uncultured_Prosthecobacter_sp._g__norank_f__DA101_soil_group	OTU279	0	0	0	0
Cyanobacteria	Cyanobacteria	Gastranaerophilales	norank_o__Gastranae	norank_o__Gastranaerophilales	unclassified_g__norank_o__Gastranaerophilales	OTU328	2	0	0	0
Proteobacteria	Epsilonproteobacteria	Campylobacterales	Helicobacteraceae	Helicobacter	Helicobacter_ganmani	OTU446	0	0	0	0
Firmicutes	Clostridia	Clostridiales	Ruminococcaceae	Ruminococcaceae_UCG-007	unclassified_g__Ruminococcaceae_UCG-007	OTU789	0	1	0	0
Proteobacteria	Alphaproteobacteria	Rhizobiales	Xanthobacteraceae	Variibacter	uncultured_forest_soil_bacterium_g__Variibacter	OTU840	0	0	0	0
Firmicutes	Clostridia	Clostridiales	Ruminococcaceae	Ruminiclostridium_5	uncultured_organism_g__Ruminiclostridium_5	OTU24	0	0	0	0
Gemmatimonade	Gemmatimonadetes	Gemmatimonadales	Gemmatimonadaceae	Gemmatimonas	uncultured_soil_bacterium_g__Gemmatimonas	OTU354	0	0	0	0
Actinobacteria	Actinobacteria	Propionibacteriales	Nocardioidaceae	Kribbella	uncultured_bacterium_g__Kribbella	OTU391	0	0	0	0
Actinobacteria	Actinobacteria	Pseudonocardiales	Pseudonocardiaceae	Pseudonocardia	uncultured_bacterium_g__Pseudonocardia	OTU392	0	0	0	0
Firmicutes	Clostridia	Clostridiales	Lachnospiraceae	unclassified_f__Lachnospiraceae	unclassified_f__Lachnospiraceae	OTU462	0	0	0	0
Firmicutes	Clostridia	Clostridiales	Ruminococcaceae	Anaerotruncus	unclassified_g__Anaerotruncus	OTU486	0	0	1	0
Actinobacteria	Actinobacteria	Coriobacteriales	Coriobacteriaceae	Gordonibacter	unclassified_g__Gordonibacter	OTU555	0	0	0	0
Firmicutes	Clostridia	Clostridiales	Ruminococcaceae	Anaerotruncus	unclassified_g__Anaerotruncus	OTU687	0	0	0	0
unclassified_k__r	unclassified_k__norank	unclassified_k__no	unclassified_k__noran	unclassified_k__norank	unclassified_k__norank	OTU706	0	0	0	0
Proteobacteria	Alphaproteobacteria	Rhizobiales	Xanthobacteraceae	Variibacter	uncultured_forest_soil_bacterium_g__Variibacter	OTU252	0	0	0	0
Actinobacteria	Actinobacteria	Micromonosporales	Micromonosporaceae	Longispora	uncultured_bacterium_g__Longispora	OTU357	0	0	0	0
Actinobacteria	Actinobacteria	Frankiales	Acidothermaceae	Acidothermus	Thermomonosporaceae_bacterium_YE4-D4-16-CH2	OTU403	0	0	0	0
Tenericutes	Mollicutes	Mollicutes_RF9	norank_o__Mollicutes	norank_o__Mollicutes_RF9	unclassified_g__norank_o__Mollicutes_RF9	OTU547	1	0	0	1
Bacteroidetes	Bacteroidia	Bacteroidales	Bacteroidaceae	Bacteroides	unclassified_g__Bacteroides	OTU554	0	0	0	0
Firmicutes	Clostridia	Clostridiales	Ruminococcaceae	unclassified_f__Ruminococcaceae	unclassified_f__Ruminococcaceae	OTU708	2	0	1	0
Firmicutes	Clostridia	Clostridiales	Christensenellaceae	unclassified_f__Christensenellaceae	unclassified_f__Christensenellaceae	OTU205	0	0	0	0
Firmicutes	Clostridia	Clostridiales	Ruminococcaceae	Ruminococcaceae_UCG-010	uncultured_bacterium_g__Ruminococcaceae_UCG-010	OTU240	0	0	0	1
Bacteroidetes	Bacteroidia	Bacteroidales	Rikenellaceae	Rikenella	uncultured_bacterium_g__Rikenella	OTU244	0	0	0	0
Bacteroidetes	Bacteroidia	Bacteroidales	Bacteroidales_S24-7_g	norank_f__Bacteroidales_S24-7_gro	uncultured_bacterium_g__norank_f__Bacteroidales_S24-7_group	OTU259	0	0	0	0
Bacteroidetes	Bacteroidia	Bacteroidales	Bacteroidales_S24-7_g	norank_f__Bacteroidales_S24-7_gro	unclassified_g__norank_f__Bacteroidales_S24-7_group	OTU280	0	0	0	0
Actinobacteria	Actinobacteria	Micromonosporales	Micromonosporaceae	unclassified_f__Micromonosporacea	unclassified_f__Micromonosporaceae	OTU286	0	0	0	0
Actinobacteria	Actinobacteria	Gaiellales	Gaiellaceae	Gaiella	uncultured_bacterium_g__Gaiella	OTU309	0	0	0	0
Bacteroidetes	Bacteroidia	Bacteroidales	Bacteroidales_S24-7_g	norank_f__Bacteroidales_S24-7_gro	uncultured_Bacteroidales_bacterium_g__norank_f__Bacteroidales_S24-7_group	OTU348	0	0	0	0
Actinobacteria	Actinobacteria	Propionibacteriales	Nocardioidaceae	Nocardioides	uncultured_bacterium_g__Nocardioides	OTU367	0	0	0	0
Actinobacteria	Actinobacteria	norank_c__Actinob	norank_c__Actinobact	norank_c__Actinobacteria	uncultured_bacterium_g__norank_c__Actinobacteria	OTU396	0	0	0	0
Firmicutes	Clostridia	Clostridiales	Ruminococcaceae	unclassified_f__Ruminococcaceae	unclassified_f__Ruminococcaceae	OTU617	1	1	0	0
Proteobacteria	Alphaproteobacteria	Rhizobiales	Methylobacteriaceae	Meganema	Meganema_perideroedes	OTU30	0	0	0	0
Firmicutes	Clostridia	Clostridiales	Ruminococcaceae	unclassified_f__Ruminococcaceae	unclassified_f__Ruminococcaceae	OTU239	0	0	1	0
Proteobacteria	Betaproteobacteria	Burkholderiales	Alcaligenaceae	Parasutterella	Sutterellaceae_bacterium_YL45	OTU281	0	0	0	0
Bacteroidetes	Bacteroidia	Bacteroidales	Bacteroidales_S24-7_g	norank_f__Bacteroidales_S24-7_gro	unclassified_g__norank_f__Bacteroidales_S24-7_group	OTU303	0	0	0	0
Bacteroidetes	Bacteroidia	Bacteroidales	Bacteroidales_S24-7_g	norank_f__Bacteroidales_S24-7_gro	uncultured_Bacteroidales_bacterium_g__norank_f__Bacteroidales_S24-7_group	OTU307	0	0	0	0
Chloroflexi	Anaerolineae	Anaerolineales	Anaerolineaceae	norank_f__Anaerolineaceae	wastewater_metagenome_g__norank_f__Anaerolineaceae	OTU352	0	0	0	0
Bacteroidetes	Bacteroidia	Bacteroidales	Bacteroidales_S24-7_g	norank_f__Bacteroidales_S24-7_gro	uncultured_bacterium_g__norank_f__Bacteroidales_S24-7_group	OTU389	0	0	0	0
Chloroflexi	Chloroflexia	Chloroflexales	Roseiflexaceae	Roseiflexus	uncultured_bacterium_g__Roseiflexus	OTU404	0	0	0	0
Firmicutes	Clostridia	Clostridiales	Lachnospiraceae	Lachnoclostridium	unclassified_g__Lachnoclostridium	OTU416	0	0	0	0
Chloroflexi	Chloroflexia	Chloroflexales	Roseiflexaceae	Roseiflexus	uncultured_bacterium_g__Roseiflexus	OTU425	0	0	0	0
Actinobacteria	Actinobacteria	Corynebacteriales	Nocardiaceae	Rhodococcus	Rhodococcus_erythropolis	OTU662	0	1	0	0
Proteobacteria	Gammaproteobacteria	Pseudomonadales	Moraxellaceae	Acinetobacter	unclassified_g__Acinetobacter	OTU685	0	0	0	0
Firmicutes	Clostridia	Clostridiales	Clostridiaceae_1	Clostridium_sensu_stricto_1	unclassified_g__Clostridium_sensu_stricto_1	OTU817	0	2	0	0
Firmicutes	Clostridia	Clostridiales	Christensenellaceae	Christensenellaceae_R-7_group	uncultured_Clostridiaceae_bacterium_g__Christensenellaceae_R-7_group	OTU47	0	0	0	0
Firmicutes	Clostridia	Clostridiales	Ruminococcaceae	Anaerotruncus	unclassified_g__Anaerotruncus	OTU96	0	0	0	0
Firmicutes	Clostridia	Clostridiales	Clostridiales_vadinBB6	norank_f__Clostridiales_vadinBB60_	uncultured_prokaryote_g__norank_f__Clostridiales_vadinBB60_group	OTU147	0	6	0	0
Firmicutes	Clostridia	Clostridiales	Ruminococcaceae	Candidatus_Soleaferrea	Ruminococcaceae_bacterium_Marseille-P2935	OTU183	0	0	0	0
Tenericutes	Mollicutes	Mollicutes_RF9	norank_o__Mollicutes	norank_o__Mollicutes_RF9	uncultured_bacterium_g__norank_o__Mollicutes_RF9	OTU208	0	0	0	0
Firmicutes	unclassified_p__Firmicut	unclassified_p__Fir	unclassified_p__Firmi	unclassified_p__Firmicutes	unclassified_p__Firmicutes	OTU246	0	0	0	0
Bacteroidetes	Bacteroidia	Bacteroidales	Bacteroidales_S24-7_g	norank_f__Bacteroidales_S24-7_gro	uncultured_bacterium_g__norank_f__Bacteroidales_S24-7_group	OTU266	0	0	0	0
Bacteroidetes	Bacteroidia	Bacteroidales	Rikenellaceae	Alistipes	uncultured_bacterium_g__Alistipes	OTU282	0	0	0	0
Actinobacteria	Actinobacteria	Solirubrobacterales	Solirubrobacteraceae	Solirubrobacter	uncultured_bacterium_g__Solirubrobacter	OTU288	0	0	0	0
Bacteroidetes	Bacteroidia	Bacteroidales	Bacteroidales_S24-7_g	norank_f__Bacteroidales_S24-7_gro	uncultured_bacterium_g__norank_f__Bacteroidales_S24-7_group	OTU289	0	0	0	0
Bacteroidetes	Bacteroidia	Bacteroidales	Bacteroidaceae	Bacteroides	unclassified_g__Bacteroides	OTU290	0	0	0	0
Chloroflexi	Chloroflexia	Chloroflexales	Roseiflexaceae	Roseiflexus	uncultured_bacterium_g__Roseiflexus	OTU318	0	0	0	0
Acidobacteria	Acidobacteria	norank_c__Acidoba	norank_c__Acidobacte	norank_c__Acidobacteria	uncultured_Acidobacteriales_bacterium_g__norank_c__Acidobacteria	OTU366	0	0	0	0
Proteobacteria	Epsilonproteobacteria	Campylobacterales	Campylobacteraceae	Arcobacter	unclassified_g__Arcobacter	OTU376	0	0	0	0
Gemmatimonade	Gemmatimonadetes	Gemmatimonadales	Gemmatimonadaceae	Gemmatimonas	unclassified_g__Gemmatimonas	OTU377	0	0	0	0
Proteobacteria	Alphaproteobacteria	Rhizobiales	Methylocystaceae	unclassified_f__Methylocystaceae	unclassified_f__Methylocystaceae	OTU379	0	0	0	0
Proteobacteria	Alphaproteobacteria	Rhodobacterales	Rhodobacteraceae	Pannonibacter	unclassified_g__Pannonibacter	OTU421	0	0	0	0
Proteobacteria	Epsilonproteobacteria	Campylobacterales	Helicobacteraceae	Helicobacter	Helicobacter_hepaticus	OTU422	0	0	0	0
Tenericutes	Mollicutes	Mollicutes_RF9	norank_o__Mollicutes	norank_o__Mollicutes_RF9	unclassified_g__norank_o__Mollicutes_RF9	OTU491	0	0	0	0
Firmicutes	Clostridia	Clostridiales	Ruminococcaceae	unclassified_f__Ruminococcaceae	unclassified_f__Ruminococcaceae	OTU696	0	0	0	0
Firmicutes	Clostridia	Clostridiales	Christensenellaceae	norank_f__Christensenellaceae	uncultured_bacterium_g__norank_f__Christensenellaceae	OTU819	0	0	1	1
Firmicutes	Clostridia	Clostridiales	Ruminococcaceae	unclassified_f__Ruminococcaceae	unclassified_f__Ruminococcaceae	OTU186	0	0	3	0
Firmicutes	Clostridia	Clostridiales	Ruminococcaceae	Ruminiclostridium	uncultured_bacterium_g__Ruminiclostridium	OTU269	0	0	0	0
Bacteroidetes	Bacteroidia	Bacteroidales	Porphyromonadaceae	Parabacteroides	uncultured_organism_g__Parabacteroides	OTU285	0	0	0	0
Bacteroidetes	Bacteroidia	Bacteroidales	Rikenellaceae	Alistipes	uncultured_bacterium_g__Alistipes	OTU299	0	0	0	0
Actinobacteria	Actinobacteria	Acidimicrobiales	norank_o__Acidimicr	norank_o__Acidimicrobiales	uncultured_Acidimicrobiales_bacterium_g__norank	OTU305	0	0	0	0
Chloroflexi	S085	norank_c__S085	norank_c__S085	norank_c__S085	uncultured_Chloroflexi_bacterium_g__norank_c__S085	OTU362	0	0	0	0
Proteobacteria	Alphaproteobacteria	Rhodospirillales	Acetobacteraceae	norank_f__Acetobacteraceae	uncultured_bacterium_g__norank_f__Acetobacteraceae	OTU371	0	0	0	0
Chloroflexi	TK10	norank_c__TK10	norank_c__TK10	norank_c__TK10	unclassified_g__norank_c__TK10	OTU382	0	0	0	0
Actinobacteria	Actinobacteria	Micrococcales	Brevibacteriaceae	Brevibacterium	unclassified_g__Brevibacterium	OTU399	0	0	0	0
Bacteroidetes	Bacteroidia	Bacteroidales	Bacteroidales_S24-7_g	norank_f__Bacteroidales_S24-7_gro	uncultured_organism_g__norank_f__Bacteroidales_S24-7_group	OTU401	0	0	0	0
Firmicutes	Erysipelotrichia	Erysipelotrichales	Erysipelotrichaceae	Erysipelatoclostridium	uncultured_bacterium_g__Erysipelatoclostridium	OTU407	0	0	0	0
Proteobacteria	Betaproteobacteria	Nitrosomonadales	Nitrosomonadaceae	norank_f__Nitrosomonadaceae	unclassified_g__norank_f__Nitrosomonadaceae	OTU420	0	0	0	0
Firmicutes	Clostridia	Clostridiales	Ruminococcaceae	Ruminococcaceae_UCG-014	uncultured_Firmicutes_bacterium_g__Ruminococcaceae_UCG-014	OTU798	0	0	0	0
Firmicutes	Clostridia	Clostridiales	Ruminococcaceae	Ruminococcaceae_UCG-010	unclassified_g__Ruminococcaceae_UCG-010	OTU800	0	0	0	0
Tenericutes	Mollicutes	Mycoplasmatales	Mycoplasmataceae	norank_f__Mycoplasmataceae	unclassified_g__norank_f__Mycoplasmataceae	OTU825	0	0	0	0
Firmicutes	Clostridia	Clostridiales	Christensenellaceae	Christensenellaceae_R-7_group	unclassified_g__Christensenellaceae_R-7_group	OTU833	0	0	0	0
Bacteroidetes	Bacteroidia	Bacteroidales	Bacteroidaceae	Bacteroides	Bacteroides_caccae	OTU46	0	0	0	0
Bacteroidetes	Flavobacteriia	Flavobacteriales	Flavobacteriaceae	Cloacibacterium	uncultured_bacterium_g__Cloacibacterium	OTU49	0	0	0	0
Firmicutes	Clostridia	Clostridiales	Ruminococcaceae	Ruminococcaceae_UCG-010	uncultured_bacterium_g__Ruminococcaceae_UCG-010	OTU97	4	0	0	0
Proteobacteria	Gammaproteobacteria	Pseudomonadales	Moraxellaceae	Acinetobacter	Acinetobacter_guillouiae	OTU138	0	2	0	0
Bacteroidetes	Bacteroidia	Bacteroidales	Bacteroidales_S24-7_g	norank_f__Bacteroidales_S24-7_gro	uncultured_bacterium_g__norank_f__Bacteroidales_S24-7_group	OTU211	0	0	0	0
Actinobacteria	Actinobacteria	Acidimicrobiales	Acidimicrobiaceae	CL500-29_marine_group	uncultured_bacterium_g__CL500-29_marine_group	OTU260	0	0	0	0
Acidobacteria	Acidobacteria	Acidobacteriales	Acidobacteriaceae__S	norank_f__Acidobacteriaceae__Subg	uncultured_bacterium_g__norank_f__Acidobacteriaceae__Subgroup_1_	OTU263	0	0	0	0
Proteobacteria	Alphaproteobacteria	Rhodospirillales	Rhodospirillaceae	norank_f__Rhodospirillaceae	unclassified_g__norank_f__Rhodospirillaceae	OTU273	0	0	0	0
Actinobacteria	Actinobacteria	Propionibacteriales	Nocardioidaceae	unclassified_f__Nocardioidaceae	unclassified_f__Nocardioidaceae	OTU298	0	0	0	0
Bacteroidetes	Bacteroidia	Bacteroidales	Bacteroidaceae	Bacteroides	Bacteroides_stercoris_ATCC_43183	OTU317	0	0	0	0
Chloroflexi	Chloroflexia	Chloroflexales	Roseiflexaceae	Roseiflexus	uncultured_bacterium_g__Roseiflexus	OTU372	0	0	0	0
Chloroflexi	Anaerolineae	Anaerolineales	Anaerolineaceae	norank_f__Anaerolineaceae	Chloroflexi_bacterium_OLB13	OTU383	0	0	0	0
Actinobacteria	Actinobacteria	Propionibacteriales	Nocardioidaceae	Nocardioides	unclassified_g__Nocardioides	OTU386	0	0	0	0
Chloroflexi	Thermomicrobia	JG30-KF-CM45	norank_o__JG30-KF-C	norank_o__JG30-KF-CM45	unclassified_g__norank_o__JG30-KF-CM45	OTU408	0	0	0	0
Chloroflexi	Gitt-GS-136	norank_c__Gitt-GS-	norank_c__Gitt-GS-13	norank_c__Gitt-GS-136	uncultured_bacterium_g__norank_c__Gitt-GS-136	OTU413	0	0	0	0

Actinobacteria	Actinobacteria	Acidimicrobiales	Iamiaceae	Iamia	uncultured_bacterium_g__Iamia	OTU417	0	0	0	0
Actinobacteria	Actinobacteria	Frankiales	Geodermatophilaceae	Blastococcus	uncultured_Blastococcus_sp._g__Blastococcus	OTU419	0	0	0	0
Actinobacteria	Actinobacteria	Propionibacteriales	Nocardioidaceae	Nocardioides	uncultured_organism_g__Nocardioides	OTU430	0	0	0	0
Gemmatimonade	Gemmatimonadetes	Gemmatimonadales	Gemmatimonadaceae	norank_f__Gemmatimonadaceae	uncultured_Gemmatimonadetes_bacterium_g__norank_f__Gemmatimonadaceae	OTU442	0	0	0	0
Firmicutes	Clostridia	Clostridiales	Ruminococcaceae	Anaerotruncus	uncultured_bacterium_g__Anaerotruncus	OTU592	2	0	0	0
Firmicutes	Clostridia	Clostridiales	Lachnospiraceae	Coprococcus_1	unclassified_g__Coprococcus_1	OTU734	1	0	0	0
Firmicutes	Clostridia	Clostridiales	Ruminococcaceae	Candidatus_Soleaferrea	Candidatus_Soleaferrea_massiliensis	OTU37	0	0	0	0
Cyanobacteria	Cyanobacteria	norank_c__Cyanoba	norank_c__Cyanobact	norank_c__Cyanobacteria	unclassified_g__norank_c__Cyanobacteria	OTU149	0	1	0	0
Firmicutes	Clostridia	Clostridiales	Ruminococcaceae	Ruminococcaceae_UCG-010	unclassified_g__Ruminococcaceae_UCG-010	OTU157	0	0	0	0
Bacteroidetes	Bacteroidia	Bacteroidales	Bacteroidales_S24-7_g	norank_f__Bacteroidales_S24-7_gro	unclassified_g__norank_f__Bacteroidales_S24-7_group	OTU190	0	0	0	0
Firmicutes	Clostridia	Clostridiales	Ruminococcaceae	Ruminococcaceae_UCG-010	uncultured_bacterium_g__Ruminococcaceae_UCG-010	OTU225	0	0	0	0
Proteobacteria	Deltaproteobacteria	Desulfovibrionales	Desulfovibrionaceae	Desulfovibrio	uncultured_bacterium_g__Desulfovibrio	OTU250	0	0	0	0
Bacteroidetes	Bacteroidia	Bacteroidales	Bacteroidales_S24-7_g	norank_f__Bacteroidales_S24-7_gro	uncultured_bacterium_g__norank_f__Bacteroidales_S24-7_group	OTU265	0	0	0	0
Proteobacteria	Betaproteobacteria	Burkholderiales	Comamonadaceae	unclassified_f__Comamonadaceae	unclassified_f__Comamonadaceae	OTU268	0	0	0	0
Firmicutes	Bacilli	Bacillales	Bacillaceae	unclassified_f__Bacillaceae	unclassified_f__Bacillaceae	OTU274	0	0	0	0
Firmicutes	Bacilli	Bacillales	Bacillaceae	Bacillus	Bacillus_anthracis_g__Bacillus	OTU275	0	0	0	0
Proteobacteria	Alphaproteobacteria	Rickettsiales	Mitochondria	norank_f__Mitochondria	Brassica_nigra__black_mustard_	OTU283	0	0	0	0
Firmicutes	Clostridia	Clostridiales	Ruminococcaceae	Anaerotruncus	Anaerotruncus_sp._G3_2012_	OTU296	0	0	0	0
Verrucomicrobia	Spartobacteria	Chthoniobacterales	DA101_soil_group	norank_f__DA101_soil_group	uncultured_Spartobacteria_bacterium_g__norank	OTU302	0	0	0	0
Actinobacteria	Actinobacteria	Acidimicrobiales	norank_o__Acidimicro	norank_o__Acidimicrobiales	uncultured_bacterium_g__norank_o__Acidimicrobiales	OTU314	0	0	0	0
Chloroflexi	Anaerolineae	Anaerolineales	Anaerolineaceae	norank_f__Anaerolineaceae	unclassified_g__norank_f__Anaerolineaceae	OTU316	0	0	0	0
Actinobacteria	Actinobacteria	Pseudonocardiales	Pseudonocardiaceae	Pseudonocardia	unclassified_g__Pseudonocardia	OTU351	0	0	0	0
Acidobacteria	Acidobacteria	Blastocatellales	Blastocatellaceae__Su	RB41	uncultured_Acidobacteria_bacterium_g__RB41	OTU359	0	0	0	0
Actinobacteria	Actinobacteria	Gaiellales	norank_o__Gaiellales	norank_o__Gaiellales	uncultured_bacterium_g__norank_o__Gaiellales	OTU370	0	0	0	0
Chloroflexi	Chloroflexia	Chloroflexales	Roseiflexaceae	Roseiflexus	uncultured_bacterium_g__Roseiflexus	OTU378	0	0	0	0
Chloroflexi	Chloroflexia	Chloroflexales	Roseiflexaceae	Roseiflexus	uncultured_bacterium_g__Roseiflexus	OTU384	0	0	0	0
Acidobacteria	Acidobacteria	Blastocatellales	Blastocatellaceae__Su	RB41	unclassified_g__RB41	OTU387	0	0	0	0
Acidobacteria	Acidobacteria	Solibacterales	Solibacteraceae__Sub	Bryobacter	uncultured_Acidobacterium_sp._g__Bryobacter	OTU388	0	0	0	0
Nitrospirae	Nitrospira	norank_c__Nitrospi	norank_c__Nitrospira	Nitrospira	unclassified_g__Nitrospira	OTU390	0	0	0	0
Actinobacteria	Actinobacteria	Acidimicrobiales	Acidimicrobiales_Ince	Candidatus_Microthrix	uncultured_bacterium_g__Candidatus_Microthrix	OTU393	0	0	0	0
Chloroflexi	Anaerolineae	Anaerolineales	Anaerolineaceae	norank_f__Anaerolineaceae	unclassified_g__norank_f__Anaerolineaceae	OTU397	0	0	0	0
Firmicutes	Clostridia	Clostridiales	Lachnospiraceae	norank_f__Lachnospiraceae	Lachnospiraceae_bacterium_A2	OTU398	0	0	0	0
Actinobacteria	Actinobacteria	Solirubrobacterales	Gsoil-1167	norank_f__Gsoil-1167	uncultured_bacterium_g__norank_f__Gsoil-1167	OTU406	0	0	0	0
Proteobacteria	Alphaproteobacteria	Rhizobiales	Bradyrhizobiaceae	Bosea	unclassified_g__Bosea	OTU426	0	0	0	0
Chloroflexi	Ktedonobacteria	JG30-KF-AS9	norank_o__JG30-KF-A	norank_o__JG30-KF-AS9	uncultured_bacterium_g__norank_o__JG30-KF-AS9	OTU440	0	0	0	0
Gemmatimonade	Gemmatimonadetes	Gemmatimonadales	Gemmatimonadaceae	Gemmatimonas	unclassified_g__Gemmatimonas	OTU441	0	0	0	0
Firmicutes	Clostridia	Clostridiales	Clostridiales_vadinBB6	norank_f__Clostridiales_vadinBB60_	unclassified_g__norank_f__Clostridiales_vadinBB60_group	OTU479	0	0	0	0
Firmicutes	Clostridia	Clostridiales	Lachnospiraceae	unclassified_f__Lachnospiraceae	unclassified_f__Lachnospiraceae	OTU514	0	0	0	0
Firmicutes	Clostridia	Clostridiales	Ruminococcaceae	unclassified_f__Ruminococcaceae	unclassified_f__Ruminococcaceae	OTU569	0	0	0	0
Firmicutes	Clostridia	Clostridiales	Ruminococcaceae	unclassified_f__Ruminococcaceae	unclassified_f__Ruminococcaceae	OTU760	0	0	0	0
Firmicutes	Clostridia	Clostridiales	Ruminococcaceae	Ruminococcaceae_UCG-005	unclassified_g__Ruminococcaceae_UCG-005	OTU814	0	0	0	0
Firmicutes	Clostridia	Clostridiales	Ruminococcaceae	Anaerotruncus	unclassified_g__Anaerotruncus	OTU44	0	0	0	0
Firmicutes	Clostridia	Clostridiales	Clostridiaceae_1	Clostridium_sensu_stricto_1	human_gut_metagenome_g__Clostridium_sensu_stricto_1	OTU77	0	0	0	0
Firmicutes	Clostridia	Clostridiales	Christensenellaceae	Christensenellaceae_R-7_group	unclassified_g__Christensenellaceae_R-7_group	OTU119	0	0	0	0
Bacteroidetes	Bacteroidia	Bacteroidales	Porphyromonadaceae	Odoribacter	uncultured_bacterium_g__Odoribacter	OTU210	0	0	0	0
Bacteroidetes	Bacteroidia	Bacteroidales	Bacteroidales_S24-7_g	norank_f__Bacteroidales_S24-7_gro	uncultured_bacterium_g__norank_f__Bacteroidales_S24-7_group	OTU217	0	0	0	0
Acidobacteria	Acidobacteria	norank_c__Acidoba	norank_c__Acidobacte	norank_c__Acidobacteria	uncultured_Acidobacteriales_bacterium_g__norank_c__Acidobacteria	OTU256	0	0	0	0
Gemmatimonade	Gemmatimonadetes	Gemmatimonadales	Gemmatimonadaceae	unclassified_f__Gemmatimonadacea	unclassified_f__Gemmatimonadaceae	OTU257	0	0	0	0
Acidobacteria	Acidobacteria	norank_c__Acidoba	norank_c__Acidobacte	norank_c__Acidobacteria	unclassified_g__norank_c__Acidobacteria	OTU264	0	0	0	0
Proteobacteria	Betaproteobacteria	Neisseriales	Neisseriaceae	Neisseria	unclassified_g__Neisseria	OTU271	0	0	0	0
Planctomycetes	OM190	norank_c__OM190	norank_c__OM190	norank_c__OM190	uncultured_bacterium_g__norank_c__OM190	OTU278	0	0	0	0
Actinobacteria	Actinobacteria	Coriobacteriales	Coriobacteriaceae	Parvibacter	uncultured_bacterium_g__Parvibacter	OTU292	0	0	0	0
Acidobacteria	Acidobacteria	norank_c__Acidoba	norank_c__Acidobacte	norank_c__Acidobacteria	uncultured_prokaryote_g__norank_c__Acidobacteria	OTU293	0	0	0	0
Actinobacteria	Actinobacteria	Gaiellales	norank_o__Gaiellales	norank_o__Gaiellales	uncultured_Rubrobacteria_bacterium_g__norank	OTU295	0	0	0	0
Proteobacteria	Gammaproteobacteria	BD7-8_marine_grou	norank_o__BD7-8_ma	norank_o__BD7-8_marine_group	unclassified_g__norank_o__BD7-8_marine_group	OTU297	0	0	0	0
Chloroflexi	Chloroflexia	Chloroflexales	Roseiflexaceae	Roseiflexus	uncultured_bacterium_g__Roseiflexus	OTU301	0	0	0	0
Actinobacteria	Actinobacteria	Propionibacteriales	Nocardioidaceae	unclassified_f__Nocardioidaceae	unclassified_f__Nocardioidaceae	OTU306	0	0	0	0
Saccharibacteria	norank_p__Saccharibact	norank_p__Sacchar	norank_p__Saccharib	norank_p__Saccharibacteria	uncultured_bacterium_g__norank_p__Saccharibacteria	OTU311	0	0	0	0
Acidobacteria	Acidobacteria	norank_c__Acidoba	norank_c__Acidobacte	norank_c__Acidobacteria	uncultured_bacterium_66	OTU315	0	0	0	0
Firmicutes	Bacilli	Bacillales	Bacillaceae	unclassified_f__Bacillaceae	unclassified_f__Bacillaceae	OTU319	0	0	0	0
Firmicutes	Clostridia	Clostridiales	Clostridiales_vadinBB6	norank_f__Clostridiales_vadinBB60_	uncultured_bacterium_g__norank_f__Clostridiales_vadinBB60_group	OTU340	0	0	0	0
Acidobacteria	Acidobacteria	norank_c__Acidoba	norank_c__Acidobacte	norank_c__Acidobacteria	unclassified_g__norank_c__Acidobacteria	OTU349	0	0	0	0
Saccharibacteria	norank_p__Saccharibact	norank_p__Sacchar	norank_p__Saccharib	norank_p__Saccharibacteria	uncultured_bacterium_g__norank_p__Saccharibacteria	OTU353	0	0	0	0
Chloroflexi	Anaerolineae	Anaerolineales	Anaerolineaceae	norank_f__Anaerolineaceae	unclassified_g__norank_f__Anaerolineaceae	OTU356	0	0	0	0
Bacteroidetes	Bacteroidia	Bacteroidales	Porphyromonadaceae	Odoribacter	uncultured_bacterium_g__Odoribacter	OTU363	0	0	0	0
Bacteroidetes	Bacteroidia	Bacteroidales	Bacteroidales_S24-7_g	norank_f__Bacteroidales_S24-7_gro	uncultured_bacterium_g__norank_f__Bacteroidales_S24-7_group	OTU364	0	0	0	0
Bacteroidetes	Cytophagia	Cytophagales	Cytophagaceae	Persicitalea	uncultured_bacterium_g__Persicitalea	OTU373	0	0	0	0
Proteobacteria	Deltaproteobacteria	Myxococcales	BIrii41	norank_f__BIrii41	unclassified_g__norank_f__BIrii41	OTU385	0	0	0	0
Chloroflexi	unclassified_p__Chlorofl	unclassified_p__Ch	unclassified_p__Chlor	unclassified_p__Chloroflexi	unclassified_p__Chloroflexi	OTU394	0	0	0	0
Planctomycetes	Planctomycetacia	Planctomycetales	Planctomycetaceae	norank_f__Planctomycetaceae	unclassified_g__norank_f__Planctomycetaceae	OTU400	0	0	0	0
Nitrospirae	Nitrospira	norank_c__Nitrospi	norank_c__Nitrospira	Nitrospira	unclassified_g__Nitrospira	OTU402	0	0	0	0
Actinobacteria	Actinobacteria	Acidimicrobiales	OM1_clade	norank_f__OM1_clade	unclassified_g__norank_f__OM1_clade	OTU412	0	0	0	0
Firmicutes	Clostridia	Clostridiales	Lachnospiraceae	norank_f__Lachnospiraceae	uncultured_bacterium_g__norank_f__Lachnospiraceae	OTU418	0	0	0	0
Gemmatimonade	Gemmatimonadetes	Gemmatimonadales	Gemmatimonadaceae	Gemmatimonas	uncultured_bacterium_g__Gemmatimonas	OTU424	0	0	0	0
Chloroflexi	Thermomicrobia	JG30-KF-CM45	norank_o__JG30-KF-C	norank_o__JG30-KF-CM45	uncultured_Sphaerobacter_sp._g__norank	OTU428	0	0	0	0
Proteobacteria	Alphaproteobacteria	Rhodospirillales	Acetobacteraceae	norank_f__Acetobacteraceae	uncultured_bacterium_g__norank_f__Acetobacteraceae	OTU434	0	0	0	0
Actinobacteria	Actinobacteria	Coriobacteriales	Coriobacteriaceae	Coriobacteriaceae_UCG-002	uncultured_bacterium_g__Coriobacteriaceae_UCG-002	OTU436	0	0	0	0
Acidobacteria	Acidobacteria	unclassified_c__Aci	unclassified_c__Acido	unclassified_c__Acidobacteria	unclassified_c__Acidobacteria	OTU438	0	0	0	0
Chloroflexi	Ktedonobacteria	C0119	norank_o__C0119	norank_o__C0119	unclassified_g__norank_o__C0119	OTU439	0	0	0	0
unclassified_k__r	unclassified_k__norank	unclassified_k__no	unclassified_k__noran	unclassified_k__norank	unclassified_k__norank	OTU453	0	0	0	0
unclassified_k__r	unclassified_k__norank	unclassified_k__no	unclassified_k__noran	unclassified_k__norank	unclassified_k__norank	OTU620	0	0	0	0
unclassified_k__r	unclassified_k__norank	unclassified_k__no	unclassified_k__noran	unclassified_k__norank	unclassified_k__norank	OTU786	0	0	0	0

Phylum	Class	Order	Family	Genus	Species	OTU	Green1	Green2	Green3	Green4
Firmicutes	Clostridia	Clostridiales	Ruminococcaceae	Faecalibacterium	uncultured_bacterium_g__Faecalibacterium	OTU705	4837	14186	4495	8480
Bacteroidetes	Bacteroidia	Bacteroidales	Rikenellaceae	Alistipes	gut_metagenome_g__Alistipes	OTU287	9959	5099	6170	5279
Firmicutes	Clostridia	Clostridiales	Lachnospiraceae	[Ruminococcus]_torques_group	uncultured_Clostridiales_bacterium_g__[Ruminococcus]_torques_group	OTU361	1339	2583	2219	4850
Proteobacteria	Gammaproteobacteria	Enterobacteriales	Enterobacteriaceae	Escherichia-Shigella	Escherichia_coli_g__Escherichia-Shigella	OTU589	2260	3435	1837	1128
Firmicutes	Clostridia	Clostridiales	Ruminococcaceae	Faecalibacterium	bacterium_ic1379	OTU50	11404	44	31	980
Bacteroidetes	Bacteroidia	Bacteroidales	Rikenellaceae	Alistipes	unclassified_g__Alistipes	OTU501	589	1106	1403	1391
Firmicutes	Bacilli	Lactobacillales	Lactobacillaceae	Lactobacillus	unclassified_g__Lactobacillus	OTU445	275	719	6667	758
Firmicutes	Clostridia	Clostridiales	Ruminococcaceae	Subdoligranulum	uncultured_bacterium_g__Subdoligranulum	OTU816	178	585	644	23
Firmicutes	Clostridia	Clostridiales	Lachnospiraceae	unclassified_f__Lachnospiraceae	unclassified_f__Lachnospiraceae	OTU581	89	1529	861	393
Firmicutes	Clostridia	Clostridiales	Lachnospiraceae	Blautia	unclassified_g__Blautia	OTU78	74	972	530	501
Firmicutes	Clostridia	Clostridiales	Lachnospiraceae	Eisenbergiella	uncultured_bacterium_g__Eisenbergiella	OTU48	512	769	487	724
Firmicutes	Clostridia	Clostridiales	Ruminococcaceae	Anaerotruncus	unclassified_g__Anaerotruncus	OTU213	321	1315	697	835
Firmicutes	Clostridia	Clostridiales	Lachnospiraceae	unclassified_f__Lachnospiraceae	unclassified_f__Lachnospiraceae	OTU710	146	498	255	307
Firmicutes	Bacilli	Lactobacillales	Lactobacillaceae	Lactobacillus	Lactobacillus_salivarius	OTU775	259	626	1	9
Firmicutes	Clostridia	Clostridiales	Lachnospiraceae	Shuttleworthia	uncultured_bacterium_g__Shuttleworthia	OTU683	260	679	1769	925
Firmicutes	Clostridia	Clostridiales	Ruminococcaceae	Butyricicoccus	uncultured_bacterium_g__Butyricicoccus	OTU616	500	248	631	293
Firmicutes	Clostridia	Clostridiales	Lachnospiraceae	[Ruminococcus]_torques_group	unclassified_g__[Ruminococcus]_torques_group	OTU196	9	1407	8	11
Firmicutes	Clostridia	Clostridiales	Lachnospiraceae	Lachnoclostridium	unclassified_g__Lachnoclostridium	OTU527	204	1359	292	363
Firmicutes	Clostridia	Clostridiales	Ruminococcaceae	Ruminiclostridium_9	unclassified_g__Ruminiclostridium_9	OTU174	402	888	844	617
Firmicutes	Clostridia	Clostridiales	Ruminococcaceae	Subdoligranulum	unclassified_g__Subdoligranulum	OTU88	156	586	421	24
Firmicutes	Clostridia	Clostridiales	Ruminococcaceae	Ruminococcaceae_UCG-014	unclassified_g__Ruminococcaceae_UCG-014	OTU712	409	393	623	690
Proteobacteria	Betaproteobacteria	Burkholderiales	Alcaligenaceae	Parasutterella	uncultured_organism_g__Parasutterella	OTU692	596	668	798	338
Firmicutes	Clostridia	Clostridiales	Ruminococcaceae	Faecalibacterium	uncultured_bacterium_g__Faecalibacterium	OTU122	10	12	87	11
Firmicutes	Clostridia	Clostridiales	Clostridiales_vadinBB6	norank_f__Clostridiales_vadinBB60_	uncultured_bacterium_g__norank_f__Clostridiales_vadinBB60_group	OTU151	1597	277	35	1100
Firmicutes	Clostridia	Clostridiales	Ruminococcaceae	Faecalibacterium	uncultured_bacterium_g__Faecalibacterium	OTU70	1830	800	391	346
Firmicutes	Clostridia	Clostridiales	Lachnospiraceae	unclassified_f__Lachnospiraceae	unclassified_f__Lachnospiraceae	OTU711	85	837	172	212
Firmicutes	Bacilli	Lactobacillales	Lactobacillaceae	Lactobacillus	Lactobacillus_vaginalis	OTU489	9	16	1007	192
Firmicutes	Clostridia	Clostridiales	Lachnospiraceae	Sellimonas	unclassified_g__Sellimonas	OTU476	113	522	196	403
Firmicutes	Clostridia	Clostridiales	Lachnospiraceae	unclassified_f__Lachnospiraceae	unclassified_f__Lachnospiraceae	OTU179	134	969	361	528
Firmicutes	Clostridia	Clostridiales	Ruminococcaceae	norank_f__Ruminococcaceae	unclassified_g__norank_f__Ruminococcaceae	OTU191	265	1719	285	89
Firmicutes	Erysipelotrichia	Erysipelotrichales	Erysipelotrichaceae	Erysipelatoclostridium	bacterium_ic1391	OTU561	40	396	451	734
Firmicutes	Clostridia	Clostridiales	Ruminococcaceae	[Eubacterium]_coprostanoligenes_g	unclassified_g__[Eubacterium]_coprostanoligenes_group	OTU13	70	832	518	926
Firmicutes	Bacilli	Lactobacillales	Enterococcaceae	Enterococcus	Enterococcus_cecorum	OTU562	121	27	3	4
Firmicutes	Clostridia	Clostridiales	Lachnospiraceae	unclassified_f__Lachnospiraceae	unclassified_f__Lachnospiraceae	OTU484	135	1082	106	98
Firmicutes	Clostridia	Clostridiales	Ruminococcaceae	unclassified_f__Ruminococcaceae	unclassified_f__Ruminococcaceae	OTU478	244	558	226	292
Firmicutes	Clostridia	Clostridiales	Lachnospiraceae	Lachnoclostridium	Clostridium_sp._BR31	OTU836	85	311	313	548
Firmicutes	Clostridia	Clostridiales	Ruminococcaceae	norank_f__Ruminococcaceae	Clostridiales_bacterium_24-4c	OTU743	194	39	48	146
Firmicutes	Bacilli	Bacillales	Bacillaceae	unclassified_f__Bacillaceae	unclassified_f__Bacillaceae	OTU851	72	0	99	94
Firmicutes	Clostridia	Clostridiales	Lachnospiraceae	[Ruminococcus]_torques_group	uncultured_Clostridiales_bacterium_g__[Ruminococcus]_torques_group	OTU774	4	188	6	45
Firmicutes	Clostridia	Clostridiales	Lachnospiraceae	[Eubacterium]_hallii_group	uncultured_bacterium_g__[Eubacterium]_hallii_group	OTU202	103	152	199	202
Firmicutes	Clostridia	Clostridiales	Lachnospiraceae	Anaerostipes	Anaerostipes_butyraticus	OTU189	28	526	121	137
Firmicutes	Clostridia	Clostridiales	Ruminococcaceae	Ruminococcaceae_UCG-014	uncultured_bacterium_g__Ruminococcaceae_UCG-014	OTU578	188	26	178	963
Firmicutes	Clostridia	Clostridiales	Lachnospiraceae	Sellimonas	bacterium_P1C11	OTU218	192	286	156	203
Firmicutes	Clostridia	Clostridiales	Christensenellaceae	Christensenellaceae_R-7_group	unclassified_g__Christensenellaceae_R-7_group	OTU709	81	280	261	666
Firmicutes	Clostridia	Clostridiales	Ruminococcaceae	Butyricicoccus	unclassified_g__Butyricicoccus	OTU33	385	55	155	68
Bacteroidetes	Bacteroidia	Bacteroidales	Bacteroidaceae	Bacteroides	Bacteroides_acidifaciens	OTU226	0	0	0	0
Firmicutes	Clostridia	Clostridiales	Ruminococcaceae	Ruminococcaceae_UCG-014	unclassified_g__Ruminococcaceae_UCG-014	OTU65	469	255	128	252
Firmicutes	Clostridia	Clostridiales	Lachnospiraceae	Lachnoclostridium	unclassified_g__Lachnoclostridium	OTU469	218	195	137	151
Firmicutes	Clostridia	Clostridiales	Ruminococcaceae	Ruminococcaceae_UCG-005	uncultured_organism_g__Ruminococcaceae_UCG-005	OTU690	240	41	74	346
Firmicutes	Erysipelotrichia	Erysipelotrichales	Erysipelotrichaceae	Erysipelatoclostridium	[Clostridium]_spiroforme	OTU625	38	100	98	412
Tenericutes	Mollicutes	Mollicutes_RF9	norank_o__Mollicutes	norank_o__Mollicutes_RF9	uncultured_bacterium_g__norank_o__Mollicutes_RF9	OTU588	333	1614	0	0
Firmicutes	Clostridia	Clostridiales	Lachnospiraceae	Tyzzerella	uncultured_organism_g__Tyzzerella	OTU795	460	74	422	241
Firmicutes	Clostridia	Clostridiales	Ruminococcaceae	Ruminiclostridium	uncultured_bacterium_g__Ruminiclostridium	OTU368	277	58	148	405
Firmicutes	Clostridia	Clostridiales	Ruminococcaceae	Butyricicoccus	Butyricicoccus_pullicaecorum_1.2	OTU769	192	92	335	676
Firmicutes	Clostridia	Clostridiales	Ruminococcaceae	Ruminococcaceae_UCG-014	uncultured_bacterium_g__Ruminococcaceae_UCG-014	OTU346	68	68	249	208
Firmicutes	Clostridia	Clostridiales	Lachnospiraceae	norank_f__Lachnospiraceae	Clostridiales_bacterium_CHKCI001	OTU738	93	320	90	260
Proteobacteria	Gammaproteobacteria	Pseudomonadales	Pseudomonadaceae	Pseudomonas	Pseudomonas_brenneri	OTU580	65	214	80	401
Firmicutes	Clostridia	Clostridiales	Ruminococcaceae	norank_f__Ruminococcaceae	unclassified_g__norank_f__Ruminococcaceae	OTU460	368	53	191	43
Firmicutes	Clostridia	Clostridiales	Ruminococcaceae	Ruminococcaceae_UCG-014	unidentified_rumen_bacterium_JW32	OTU565	105	32	194	145
Firmicutes	Clostridia	Clostridiales	Ruminococcaceae	Ruminococcaceae_UCG-014	uncultured_rumen_bacterium_g__Ruminococcaceae_UCG-014	OTU813	5	0	190	152
Firmicutes	Clostridia	Clostridiales	Lachnospiraceae	Lachnospiraceae_NK4A136_group	unclassified_g__Lachnospiraceae_NK4A136_group	OTU193	161	89	443	73
Firmicutes	Clostridia	Clostridiales	Clostridiales_vadinBB6	norank_f__Clostridiales_vadinBB60_	uncultured_bacterium_g__norank_f__Clostridiales_vadinBB60_group	OTU573	476	307	108	290
Firmicutes	Clostridia	Clostridiales	Lachnospiraceae	Coprococcus_1	uncultured_organism_g__Coprococcus_1	OTU591	81	157	134	138
Firmicutes	Clostridia	Clostridiales	Lachnospiraceae	unclassified_f__Lachnospiraceae	unclassified_f__Lachnospiraceae	OTU515	32	124	62	143
Firmicutes	Clostridia	Clostridiales	Ruminococcaceae	Ruminococcaceae_UCG-014	uncultured_organism_g__Ruminococcaceae_UCG-014	OTU457	34	24	46	91
Firmicutes	Clostridia	Clostridiales	Lachnospiraceae	Blautia	Blautia_hydrogenotrophica	OTU718	27	221	133	36
Firmicutes	Clostridia	Clostridiales	Ruminococcaceae	norank_f__Ruminococcaceae	unclassified_g__norank_f__Ruminococcaceae	OTU735	57	60	109	109
Firmicutes	Clostridia	Clostridiales	Peptostreptococcacea	Romboutsia	uncultured_bacterium_g__Romboutsia	OTU79	17	10	0	0
Firmicutes	Clostridia	Clostridiales	Ruminococcaceae	Anaerotruncus	uncultured_bacterium_g__Anaerotruncus	OTU499	167	486	307	145
Firmicutes	Clostridia	Clostridiales	Ruminococcaceae	Ruminococcaceae_UCG-014	uncultured_bacterium_g__Ruminococcaceae_UCG-014	OTU7	66	124	270	301
Firmicutes	Clostridia	Clostridiales	Ruminococcaceae	Ruminococcaceae_UCG-014	unclassified_g__Ruminococcaceae_UCG-014	OTU25	64	152	109	178
Firmicutes	Clostridia	Clostridiales	Ruminococcaceae	[Eubacterium]_coprostanoligenes_g	gut_metagenome_g__[Eubacterium]_coprostanoligenes_group	OTU214	11	172	286	53
Firmicutes	Clostridia	Clostridiales	Ruminococcaceae	Ruminococcaceae_UCG-005	unclassified_g__Ruminococcaceae_UCG-005	OTU160	160	49	33	116
Firmicutes	Clostridia	Clostridiales	Ruminococcaceae	Flavonifractor	uncultured_bacterium_g__Flavonifractor	OTU716	51	181	48	155
Firmicutes	Clostridia	Clostridiales	Ruminococcaceae	Ruminococcaceae_UCG-014	uncultured_bacterium_g__Ruminococcaceae_UCG-014	OTU691	31	16	53	109
Firmicutes	Clostridia	Clostridiales	Ruminococcaceae	Ruminococcaceae_UCG-005	uncultured_prokaryote_g__Ruminococcaceae_UCG-005	OTU504	193	0	57	353
Firmicutes	Clostridia	Clostridiales	Ruminococcaceae	unclassified_f__Ruminococcaceae	unclassified_f__Ruminococcaceae	OTU496	198	41	233	51
Firmicutes	Clostridia	Clostridiales	Ruminococcaceae	Butyricicoccus	unclassified_g__Butyricicoccus	OTU480	0	503	318	52
Firmicutes	Clostridia	Clostridiales	Lachnospiraceae	unclassified_f__Lachnospiraceae	unclassified_f__Lachnospiraceae	OTU533	147	58	62	83
Firmicutes	Bacilli	Lactobacillales	Streptococcaceae	Streptococcus	Streptococcus_gallolyticus_subsp._macedonicus	OTU474	1	0	0	0
Firmicutes	Clostridia	Clostridiales	Ruminococcaceae	norank_f__Ruminococcaceae	Ruminococcaceae_bacterium_AM2	OTU242	59	121	164	40
Firmicutes	Clostridia	Clostridiales	Clostridiales_vadinBB6	norank_f__Clostridiales_vadinBB60_	uncultured_organism_g__norank_f__Clostridiales_vadinBB60_group	OTU150	0	0	0	0
Firmicutes	Clostridia	Clostridiales	Lachnospiraceae	Shuttleworthia	uncultured_bacterium_g__Shuttleworthia	OTU507	67	64	402	225
Firmicutes	Clostridia	Clostridiales	Lachnospiraceae	Blautia	uncultured_bacterium_g__Blautia	OTU450	13	21	15	503
Firmicutes	Clostridia	Clostridiales	Ruminococcaceae	Ruminococcaceae_UCG-014	uncultured_rumen_bacterium_g__Ruminococcaceae_UCG-014	OTU230	248	5	1	787
Firmicutes	Clostridia	Clostridiales	Ruminococcaceae	Ruminococcaceae_UCG-004	unclassified_g__Ruminococcaceae_UCG-004	OTU355	83	137	61	80
Firmicutes	Clostridia	Clostridiales	Ruminococcaceae	norank_f__Ruminococcaceae	human_gut_metagenome_g__norank_f__Ruminococcaceae	OTU623	55	240	122	212
Firmicutes	Clostridia	Clostridiales	Lachnospiraceae	[Ruminococcus]_gauvreauii_group	bacterium_ic1296	OTU564	20	95	42	34
Firmicutes	Clostridia	Clostridiales	Ruminococcaceae	Anaerofilum	uncultured_bacterium_g__Anaerofilum	OTU123	47	27	110	88
Firmicutes	Clostridia	Clostridiales	Lachnospiraceae	unclassified_f__Lachnospiraceae	unclassified_f__Lachnospiraceae	OTU834	25	120	136	287
Firmicutes	Clostridia	Clostridiales	Clostridiales_vadinBB6	norank_f__Clostridiales_vadinBB60_	uncultured_bacterium_g__norank_f__Clostridiales_vadinBB60_group	OTU512	217	24	23	56
Firmicutes	Erysipelotrichia	Erysipelotrichales	Erysipelotrichaceae	Erysipelatoclostridium	Candidatus_Stoquefichus_sp._SN16	OTU707	1	5	173	14
Firmicutes	Clostridia	Clostridiales	Lachnospiraceae	[Eubacterium]_hallii_group	uncultured_bacterium_g__[Eubacterium]_hallii_group	OTU848	16	157	68	18
Firmicutes	Clostridia	Clostridiales	Ruminococcaceae	Ruminiclostridium_5	uncultured_bacterium_g__Ruminiclostridium_5	OTU856	91	163	54	70
Firmicutes	Clostridia	Clostridiales	Christensenellaceae	Christensenellaceae_R-7_group	uncultured_bacterium_g__Christensenellaceae_R-7_group	OTU171	37	109	19	99
Firmicutes	Clostridia	Clostridiales	Lachnospiraceae	Blautia	unclassified_g__Blautia	OTU767	0	69	50	53
Firmicutes	Clostridia	Clostridiales	Lachnospiraceae	unclassified_f__Lachnospiraceae	unclassified_f__Lachnospiraceae	OTU828	47	34	15	37
Firmicutes	Clostridia	Clostridiales	Ruminococcaceae	Ruminococcaceae_UCG-005	uncultured_Clostridiales_bacterium_g__Ruminococcaceae_UCG-005	OTU815	162	8	183	24
Firmicutes	Clostridia	Clostridiales	Ruminococcaceae	unclassified_f__Ruminococcaceae	unclassified_f__Ruminococcaceae	OTU845	12	29	52	79
Firmicutes	Clostridia	Clostridiales	Lachnospiraceae	unclassified_f__Lachnospiraceae	unclassified_f__Lachnospiraceae	OTU770	2	16	27	76
Firmicutes	Clostridia	Clostridiales	Ruminococcaceae	Anaerotruncus	uncultured_bacterium_g__Anaerotruncus	OTU838	37	277	71	104
Firmicutes	Clostridia	Clostridiales	Lachnospiraceae	unclassified_f__Lachnospiraceae	unclassified_f__Lachnospiraceae	OTU82	87	434	47	68
Firmicutes	Clostridia	Clostridiales	Ruminococcaceae	Anaerotruncus	Anaerotruncus_colihominis_DSM_17241	OTU125	897	5	106	24
Firmicutes	Clostridia	Clostridiales	Clostridiales_vadinBB6	norank_f__Clostridiales_vadinBB60_	uncultured_Clostridia_bacterium_g__norank_f__Clostridiales_vadinBB60_group	OTU697	404	131	3	237
Proteobacteria	Gammaproteobacteria	Pseudomonadales	Pseudomonadaceae	Pseudomonas	unclassified_g__Pseudomonas	OTU58	17	89	35	201
Firmicutes	Clostridia	Clostridiales	Christensenellaceae	Christensenellaceae_R-7_group	unclassified_g__Christensenellaceae_R-7_group	OTU830	86	25	42	115
Firmicutes	Clostridia	Clostridiales	Ruminococcaceae	Subdoligranulum	uncultured_Subdoligranulum_sp.	OTU519	48	3	1	80
Firmicutes	Clostridia	Clostridiales	Lachnospiraceae	Marvinbryantia	unclassified_g__Marvinbryantia	OTU177	7	5	294	18
Firmicutes	Clostridia	Clostridiales	Clostridiales_vadinBB6	norank_f__Clostridiales_vadinBB60_	unclassified_g__norank_f__Clostridiales_vadinBB60_group	OTU107	137	0	135	375
Firmicutes	Clostridia	Clostridiales	Lachnospiraceae	unclassified_f__Lachnospiraceae	unclassified_f__Lachnospiraceae	OTU216	2	87	132	78

Firmicutes	Clostridia	Clostridiales	Ruminococcaceae	Ruminococcaceae_NK4A214_group	uncultured_organism_g__Ruminococcaceae_NK4A214_group	OTU703	95	96	49	160
Firmicutes	Clostridia	Clostridiales	Lachnospiraceae	unclassified_f__Lachnospiraceae	unclassified_f__Lachnospiraceae	OTU540	70	106	5	311
Firmicutes	Clostridia	Clostridiales	Defluviitaleaceae	Defluviitaleaceae_UCG-011	uncultured_bacterium_g__Defluviitaleaceae_UCG-011	OTU725	16	115	48	64
Firmicutes	Clostridia	Clostridiales	Ruminococcaceae	Ruminococcaceae_UCG-010	uncultured_bacterium_g__Ruminococcaceae_UCG-010	OTU799	77	90	34	203
Firmicutes	Clostridia	Clostridiales	Ruminococcaceae	Ruminococcaceae_UCG-014	unclassified_g__Ruminococcaceae_UCG-014	OTU163	23	2	123	163
Firmicutes	Clostridia	Clostridiales	Clostridiales_vadinBB6	norank_f__Clostridiales_vadinBB60_	unclassified_g__norank_f__Clostridiales_vadinBB60_group	OTU57	26	96	15	55
Tenericutes	Mollicutes	Mollicutes_RF9	norank_o__Mollicutes	norank_o__Mollicutes_RF9	unclassified_g__norank_o__Mollicutes_RF9	OTU294	24	243	0	192
Firmicutes	Clostridia	Clostridiales	Ruminococcaceae	Hydrogenoanaerobacterium	uncultured_bacterium_g__Hydrogenoanaerobacterium	OTU613	114	38	133	162
Firmicutes	Clostridia	Clostridiales	Ruminococcaceae	Ruminococcaceae_UCG-014	unclassified_g__Ruminococcaceae_UCG-014	OTU405	4	31	50	55
Firmicutes	Clostridia	Clostridiales	Ruminococcaceae	Ruminococcaceae_UCG-014	uncultured_bacterium_g__Ruminococcaceae_UCG-014	OTU570	90	10	93	28
Firmicutes	Clostridia	Clostridiales	Ruminococcaceae	norank_f__Ruminococcaceae	unclassified_g__norank_f__Ruminococcaceae	OTU313	16	210	27	115
Firmicutes	Bacilli	Lactobacillales	Lactobacillaceae	Lactobacillus	Lactobacillus_aviarius	OTU599	0	0	1	0
Firmicutes	Clostridia	Clostridiales	Lachnospiraceae	unclassified_f__Lachnospiraceae	unclassified_f__Lachnospiraceae	OTU717	11	50	284	39
Firmicutes	Clostridia	Clostridiales	Lachnospiraceae	Eisenbergiella	unclassified_g__Eisenbergiella	OTU443	8	334	82	53
Firmicutes	Clostridia	Clostridiales	Ruminococcaceae	norank_f__Ruminococcaceae	Ruminococcus_sp._16442	OTU113	23	46	49	28
Firmicutes	Clostridia	Clostridiales	Ruminococcaceae	Ruminococcus_1	unclassified_g__Ruminococcus_1	OTU140	32	134	40	53
Firmicutes	Clostridia	Clostridiales	Lachnospiraceae	Lachnospira	unclassified_g__Lachnospira	OTU170	51	55	299	83
Tenericutes	Mollicutes	Mollicutes_RF9	norank_o__Mollicutes	norank_o__Mollicutes_RF9	uncultured_bacterium_g__norank_o__Mollicutes_RF9	OTU823	15	54	6	70
Firmicutes	Clostridia	Clostridiales	Ruminococcaceae	unclassified_f__Ruminococcaceae	unclassified_f__Ruminococcaceae	OTU835	31	337	25	19
Firmicutes	Erysipelotrichia	Erysipelotrichales	Erysipelotrichaceae	norank_f__Erysipelotrichaceae	Clostridiales_bacterium_10-3b	OTU821	7	36	38	49
Firmicutes	Clostridia	Clostridiales	Peptococcaceae	norank_f__Peptococcaceae	uncultured_bacterium_g__norank_f__Peptococcaceae	OTU451	30	32	45	75
Firmicutes	Clostridia	Clostridiales	Lachnospiraceae	unclassified_f__Lachnospiraceae	unclassified_f__Lachnospiraceae	OTU135	0	0	2	121
Firmicutes	Clostridia	Clostridiales	Lachnospiraceae	unclassified_f__Lachnospiraceae	unclassified_f__Lachnospiraceae	OTU550	2	61	4	0
Firmicutes	Clostridia	Clostridiales	Ruminococcaceae	Anaerotruncus	uncultured_bacterium_g__Anaerotruncus	OTU556	9	29	60	41
Firmicutes	Erysipelotrichia	Erysipelotrichales	Erysipelotrichaceae	norank_f__Erysipelotrichaceae	Clostridiales_bacterium_60-7e	OTU583	8	95	43	35
Firmicutes	Clostridia	Clostridiales	Ruminococcaceae	Ruminococcaceae_UCG-014	unclassified_g__Ruminococcaceae_UCG-014	OTU200	13	34	68	8
Bacteroidetes	Bacteroidia	Bacteroidales	Rikenellaceae	Alistipes	gut_metagenome_g__Alistipes	OTU197	67	48	117	40
Tenericutes	Mollicutes	Mollicutes_RF9	norank_o__Mollicutes	norank_o__Mollicutes_RF9	gut_metagenome_g__norank_o__Mollicutes_RF9	OTU156	4	2	6	180
Firmicutes	Clostridia	Clostridiales	Ruminococcaceae	unclassified_f__Ruminococcaceae	unclassified_f__Ruminococcaceae	OTU66	10	50	26	0
Firmicutes	Erysipelotrichia	Erysipelotrichales	Erysipelotrichaceae	Faecalitalea	unclassified_g__Faecalitalea	OTU784	27	6	9	81
Firmicutes	Bacilli	Lactobacillales	Lactobacillaceae	Lactobacillus	Lactobacillus_johnsonii	OTU601	124	77	1	379
Bacteroidetes	Bacteroidia	Bacteroidales	Rikenellaceae	Alistipes	unclassified_g__Alistipes	OTU577	79	31	95	54
Firmicutes	Clostridia	Clostridiales	Ruminococcaceae	Subdoligranulum	unclassified_g__Subdoligranulum	OTU83	14	34	22	3
Proteobacteria	Deltaproteobacteria	Desulfovibrionales	Desulfovibrionaceae	Desulfovibrio	unclassified_g__Desulfovibrio	OTU636	35	19	44	39
Firmicutes	Clostridia	Clostridiales	Clostridiales_vadinBB6	norank_f__Clostridiales_vadinBB60_	unclassified_g__norank_f__Clostridiales_vadinBB60_group	OTU102	209	0	3	3
Firmicutes	Erysipelotrichia	Erysipelotrichales	Erysipelotrichaceae	Erysipelatoclostridium	unclassified_g__Erysipelatoclostridium	OTU8	16	57	33	36
Firmicutes	Clostridia	Clostridiales	Lachnospiraceae	[Ruminococcus]_torques_group	unclassified_g__[Ruminococcus]_torques_group	OTU531	5	76	7	9
Firmicutes	Clostridia	Clostridiales	Ruminococcaceae	Ruminococcaceae_UCG-014	unclassified_g__Ruminococcaceae_UCG-014	OTU614	0	0	1	71
Actinobacteria	Actinobacteria	Coriobacteriales	Coriobacteriaceae	Senegalimassilia	Coriobacteriaceae_bacterium_CHKCI002	OTU206	27	46	12	17
Firmicutes	Clostridia	Clostridiales	Ruminococcaceae	Ruminococcaceae_UCG-014	uncultured_bacterium_g__Ruminococcaceae_UCG-014	OTU842	28	75	83	245
Firmicutes	Clostridia	Clostridiales	Ruminococcaceae	Anaerotruncus	uncultured_organism_g__Anaerotruncus	OTU568	119	11	116	58
Firmicutes	Clostridia	Clostridiales	Ruminococcaceae	norank_f__Ruminococcaceae	unclassified_g__norank_f__Ruminococcaceae	OTU141	7	80	60	20
Firmicutes	Clostridia	Clostridiales	Clostridiales_vadinBB6	norank_f__Clostridiales_vadinBB60_	uncultured_bacterium_g__norank_f__Clostridiales_vadinBB60_group	OTU109	388	0	0	1
Cyanobacteria	Cyanobacteria	Gastranaerophilales	norank_o__Gastranae	norank_o__Gastranaerophilales	uncultured_rumen_bacterium_g__norank_o__Gastranaerophilales	OTU142	0	0	0	0
Firmicutes	Clostridia	Clostridiales	Lachnospiraceae	unclassified_f__Lachnospiraceae	unclassified_f__Lachnospiraceae	OTU699	12	30	18	82
Firmicutes	Clostridia	Clostridiales	Ruminococcaceae	Ruminococcaceae_UCG-010	gut_metagenome_g__Ruminococcaceae_UCG-010	OTU827	43	47	41	28
Firmicutes	Clostridia	Clostridiales	Lachnospiraceae	unclassified_f__Lachnospiraceae	unclassified_f__Lachnospiraceae	OTU612	11	40	47	76
Firmicutes	Clostridia	Clostridiales	Defluviitaleaceae	Defluviitaleaceae_UCG-011	uncultured_bacterium_g__Defluviitaleaceae_UCG-011	OTU567	24	35	24	86
Firmicutes	Clostridia	Clostridiales	Lachnospiraceae	Blautia	uncultured_bacterium_g__Blautia	OTU766	2	110	27	1
Firmicutes	Clostridia	Clostridiales	Christensenellaceae	Christensenellaceae_R-7_group	unclassified_g__Christensenellaceae_R-7_group	OTU148	7	27	14	44
Tenericutes	Mollicutes	Mollicutes_RF9	norank_o__Mollicutes	norank_o__Mollicutes_RF9	uncultured_bacterium_g__norank_o__Mollicutes_RF9	OTU559	28	21	5	44
Firmicutes	Clostridia	Clostridiales	Ruminococcaceae	Anaerotruncus	unclassified_g__Anaerotruncus	OTU657	14	112	10	4
Firmicutes	Clostridia	Clostridiales	Clostridiales_vadinBB6	norank_f__Clostridiales_vadinBB60_	uncultured_bacterium_g__norank_f__Clostridiales_vadinBB60_group	OTU22	475	0	25	13
Firmicutes	Clostridia	Clostridiales	Ruminococcaceae	Anaerotruncus	uncultured_Clostridiaceae_bacterium_g__Anaerotruncus	OTU618	16	119	83	25
Firmicutes	Clostridia	Clostridiales	Ruminococcaceae	Flavonifractor	uncultured_bacterium_g__Flavonifractor	OTU511	26	5	84	18
Firmicutes	Clostridia	Clostridiales	Clostridiales_vadinBB6	norank_f__Clostridiales_vadinBB60_	uncultured_bacterium_g__norank_f__Clostridiales_vadinBB60_group	OTU761	8	0	0	18
Firmicutes	Clostridia	Clostridiales	Clostridiales_vadinBB6	norank_f__Clostridiales_vadinBB60_	unclassified_g__norank_f__Clostridiales_vadinBB60_group	OTU231	38	26	9	19
Firmicutes	Clostridia	Clostridiales	Lachnospiraceae	unclassified_f__Lachnospiraceae	unclassified_f__Lachnospiraceae	OTU73	59	36	18	33
Firmicutes	Erysipelotrichia	Erysipelotrichales	Erysipelotrichaceae	Erysipelatoclostridium	Clostridiales_bacterium_CHKCI006	OTU700	4	45	17	38
Firmicutes	Clostridia	Clostridiales	Lachnospiraceae	unclassified_f__Lachnospiraceae	unclassified_f__Lachnospiraceae	OTU742	29	2	22	44
Firmicutes	Clostridia	Clostridiales	Ruminococcaceae	Anaerotruncus	uncultured_bacterium_g__Anaerotruncus	OTU146	8	24	7	9
Firmicutes	Clostridia	Clostridiales	Ruminococcaceae	Ruminococcaceae_UCG-010	uncultured_bacterium_g__Ruminococcaceae_UCG-010	OTU741	45	107	66	107
Firmicutes	Clostridia	Clostridiales	Ruminococcaceae	Subdoligranulum	unclassified_g__Subdoligranulum	OTU62	70	2	64	98
Firmicutes	Clostridia	Clostridiales	Lachnospiraceae	Blautia	uncultured_bacterium_g__Blautia	OTU466	6	18	17	43
Firmicutes	Clostridia	Clostridiales	Christensenellaceae	Christensenellaceae_R-7_group	uncultured_bacterium_g__Christensenellaceae_R-7_group	OTU746	5	43	32	101
Tenericutes	Mollicutes	Mollicutes_RF9	norank_o__Mollicutes	norank_o__Mollicutes_RF9	uncultured_bacterium_g__norank_o__Mollicutes_RF9	OTU192	20	0	3	2
Firmicutes	Clostridia	Clostridiales	Clostridiales_vadinBB6	norank_f__Clostridiales_vadinBB60_	uncultured_organism_g__norank_f__Clostridiales_vadinBB60_group	OTU103	5	0	0	12
Firmicutes	Clostridia	Clostridiales	Lachnospiraceae	unclassified_f__Lachnospiraceae	unclassified_f__Lachnospiraceae	OTU473	0	49	1	8
Firmicutes	Clostridia	Clostridiales	Ruminococcaceae	Ruminiclostridium_9	uncultured_bacterium_g__Ruminiclostridium_9	OTU110	95	1	8	15
Firmicutes	Bacilli	Lactobacillales	Lactobacillaceae	Lactobacillus	Lactobacillus_agilis	OTU607	0	0	0	0
Firmicutes	Clostridia	Clostridiales	Lachnospiraceae	Marvinbryantia	uncultured_organism_g__Marvinbryantia	OTU437	5	16	11	26
Firmicutes	Clostridia	Clostridiales	Ruminococcaceae	Ruminococcaceae_UCG-014	unclassified_g__Ruminococcaceae_UCG-014	OTU338	36	0	180	162
Firmicutes	Clostridia	Clostridiales	Clostridiales_vadinBB6	norank_f__Clostridiales_vadinBB60_	uncultured_rumen_bacterium_g__norank_f__Clostridiales_vadinBB60_group	OTU330	46	0	3	9
Tenericutes	Mollicutes	Mollicutes_RF9	norank_o__Mollicutes	norank_o__Mollicutes_RF9	uncultured_bacterium_g__norank_o__Mollicutes_RF9	OTU28	3	16	9	30
Firmicutes	Clostridia	Clostridiales	Lachnospiraceae	Coprococcus_1	unclassified_g__Coprococcus_1	OTU539	9	3	12	40
Firmicutes	Clostridia	Clostridiales	Lachnospiraceae	unclassified_f__Lachnospiraceae	unclassified_f__Lachnospiraceae	OTU642	4	18	53	8
Firmicutes	Clostridia	Clostridiales	Ruminococcaceae	Ruminococcaceae_UCG-014	unclassified_g__Ruminococcaceae_UCG-014	OTU590	39	11	43	47
Firmicutes	Clostridia	Clostridiales	Ruminococcaceae	norank_f__Ruminococcaceae	unclassified_g__norank_f__Ruminococcaceae	OTU701	8	8	77	1
Firmicutes	Clostridia	Clostridiales	Ruminococcaceae	Anaerotruncus	uncultured_bacterium_g__Anaerotruncus	OTU249	31	5	19	164
Firmicutes	Clostridia	Clostridiales	Ruminococcaceae	Anaerotruncus	uncultured_bacterium_g__Anaerotruncus	OTU248	69	43	19	18
Firmicutes	Clostridia	Clostridiales	Ruminococcaceae	Ruminiclostridium_5	unclassified_g__Ruminiclostridium_5	OTU826	4	3	1	75
Firmicutes	Clostridia	Clostridiales	Family_XIII	[Eubacterium]_nodatum_group	Eubacteriaceae_bacterium_Marseille-P2843	OTU585	24	51	67	50
Firmicutes	Clostridia	Clostridiales	Christensenellaceae	Christensenellaceae_R-7_group	uncultured_bacterium_g__Christensenellaceae_R-7_group	OTU167	15	29	4	23
Firmicutes	Clostridia	Clostridiales	Lachnospiraceae	norank_f__Lachnospiraceae	Clostridiales_bacterium_CHKCI001	OTU520	12	66	13	37
Firmicutes	Clostridia	Clostridiales	Ruminococcaceae	unclassified_f__Ruminococcaceae	unclassified_f__Ruminococcaceae	OTU238	26	99	27	43
Firmicutes	Clostridia	Clostridiales	Ruminococcaceae	unclassified_f__Ruminococcaceae	unclassified_f__Ruminococcaceae	OTU133	72	157	21	22
Tenericutes	Mollicutes	Mollicutes_RF9	norank_o__Mollicutes	norank_o__Mollicutes_RF9	uncultured_bacterium_g__norank_o__Mollicutes_RF9	OTU606	28	20	3	18
Firmicutes	Clostridia	Clostridiales	Ruminococcaceae	Ruminococcaceae_UCG-009	uncultured_bacterium_g__Ruminococcaceae_UCG-009	OTU779	4	23	28	17
Firmicutes	Clostridia	Clostridiales	Ruminococcaceae	Ruminococcaceae_NK4A214_group	uncultured_organism_g__Ruminococcaceae_NK4A214_group	OTU410	8	13	20	39
Verrucomicrobia	Verrucomicrobiae	Verrucomicrobiales	Verrucomicrobiaceae	Akkermansia	unclassified_g__Akkermansia	OTU344	0	0	0	0
Firmicutes	Clostridia	Clostridiales	Clostridiales_vadinBB6	norank_f__Clostridiales_vadinBB60_	uncultured_bacterium_g__norank_f__Clostridiales_vadinBB60_group	OTU1	2	0	0	1
Firmicutes	Clostridia	Clostridiales	Ruminococcaceae	Oscillospira	uncultured_bacterium_g__Oscillospira	OTU158	64	0	24	27
Firmicutes	Clostridia	Clostridiales	Lachnospiraceae	Lachnospiraceae_NK4A136_group	unclassified_g__Lachnospiraceae_NK4A136_group	OTU187	49	1	0	84
Firmicutes	Clostridia	Clostridiales	Lachnospiraceae	unclassified_f__Lachnospiraceae	unclassified_f__Lachnospiraceae	OTU463	9	6	90	18
Firmicutes	Clostridia	Clostridiales	Peptostreptococcacea	unclassified_f__Peptostreptococca	unclassified_f__Peptostreptococcaceae	OTU526	7	7	0	5
Firmicutes	Bacilli	Lactobacillales	Lactobacillaceae	Lactobacillus	unclassified_g__Lactobacillus	OTU448	1	0	140	6
Firmicutes	Clostridia	Clostridiales	Ruminococcaceae	Ruminococcaceae_NK4A214_group	uncultured_rumen_bacterium_g__Ruminococcaceae_NK4A214_group	OTU181	0	0	1	0
Firmicutes	Clostridia	Clostridiales	Ruminococcaceae	Ruminococcaceae_UCG-014	unclassified_g__Ruminococcaceae_UCG-014	OTU822	6	7	98	51
Firmicutes	Clostridia	Clostridiales	Ruminococcaceae	Ruminococcaceae_UCG-013	unclassified_g__Ruminococcaceae_UCG-013	OTU458	0	0	0	91
Firmicutes	Clostridia	Clostridiales	Clostridiales_vadinBB6	norank_f__Clostridiales_vadinBB60_	unclassified_g__norank_f__Clostridiales_vadinBB60_group	OTU198	7	25	20	64
Firmicutes	Clostridia	Clostridiales	Clostridiales_vadinBB6	norank_f__Clostridiales_vadinBB60_	unclassified_g__norank_f__Clostridiales_vadinBB60_group	OTU241	0	0	0	0
Tenericutes	Mollicutes	Mollicutes_RF9	norank_o__Mollicutes	norank_o__Mollicutes_RF9	unclassified_g__norank_o__Mollicutes_RF9	OTU803	0	0	15	25
Firmicutes	Clostridia	Clostridiales	Lachnospiraceae	unclassified_f__Lachnospiraceae	unclassified_f__Lachnospiraceae	OTU81	5	21	28	0
Firmicutes	Clostridia	Clostridiales	Ruminococcaceae	Ruminococcaceae_UCG-014	unclassified_g__Ruminococcaceae_UCG-014	OTU12	3	14	87	22
Firmicutes	Clostridia	Clostridiales	Christensenellaceae	Christensenellaceae_R-7_group	unclassified_g__Christensenellaceae_R-7_group	OTU165	152	6	0	2
Firmicutes	Clostridia	Clostridiales	unclassified_o__Clostr	unclassified_o__Clostridiales	unclassified_o__Clostridiales	OTU682	12	7	22	6
Firmicutes	Clostridia	Clostridiales	Ruminococcaceae	unclassified_f__Ruminococcaceae	unclassified_f__Ruminococcaceae	OTU679	42	8	0	66
Firmicutes	Clostridia	Clostridiales	Ruminococcaceae	unclassified_f__Ruminococcaceae	unclassified_f__Ruminococcaceae	OTU455	6	6	17	6
Firmicutes	Clostridia	Clostridiales	Family_XIII	unclassified_f__Family_XIII	unclassified_f__Family_XIII	OTU488	5	31	21	31
Firmicutes	Clostridia	Clostridiales	Ruminococcaceae	Ruminococcaceae_UCG-013	uncultured_bacterium_g__Ruminococcaceae_UCG-013	OTU498	7	28	1	11
Firmicutes	Clostridia	Clostridiales	Ruminococcaceae	Anaerotruncus	unclassified_g__Anaerotruncus	OTU566	269	1	15	21

Firmicutes	Clostridia	Clostridiales	Clostridiales_vadinBB6	norank_f__Clostridiales_vadinBB60_	uncultured_Firmicutes_bacterium_g__norank_f__Clostridiales_vadinBB60_group	OTU638	112	9	29	2
Firmicutes	Clostridia	Clostridiales	Clostridiales_vadinBB6	norank_f__Clostridiales_vadinBB60_	uncultured_bacterium_g__norank_f__Clostridiales_vadinBB60_group	OTU3	5	2	0	57
Firmicutes	Clostridia	Clostridiales	Clostridiales_vadinBB6	norank_f__Clostridiales_vadinBB60_	uncultured_bacterium_g__norank_f__Clostridiales_vadinBB60_group	OTU494	19	27	40	65
Firmicutes	Clostridia	Clostridiales	Ruminococcaceae	Ruminiclostridium_5	uncultured_bacterium_g__Ruminiclostridium_5	OTU849	31	11	19	19
Firmicutes	Clostridia	Clostridiales	Ruminococcaceae	Ruminococcaceae_UCG-014	uncultured_bacterium_g__Ruminococcaceae_UCG-014	OTU114	25	1	58	79
Firmicutes	Clostridia	Clostridiales	Ruminococcaceae	Ruminococcaceae_UCG-010	uncultured_bacterium_g__Ruminococcaceae_UCG-010	OTU326	0	0	237	10
Firmicutes	Clostridia	Clostridiales	Ruminococcaceae	Ruminococcaceae_UCG-014	uncultured_bacterium_g__Ruminococcaceae_UCG-014	OTU749	17	3	17	25
Firmicutes	Clostridia	Clostridiales	Ruminococcaceae	[Eubacterium]_coprostanoligenes_g	unclassified_g__[Eubacterium]_coprostanoligenes_group	OTU233	7	16	0	13
Firmicutes	Clostridia	Clostridiales	Ruminococcaceae	Ruminococcaceae_UCG-014	unidentified_rumen_bacterium_JW32	OTU36	6	17	5	38
Firmicutes	Clostridia	Clostridiales	Ruminococcaceae	Ruminococcaceae_UCG-008	uncultured_bacterium_g__Ruminococcaceae_UCG-008	OTU39	0	1	0	0
Firmicutes	Clostridia	Clostridiales	Family_XIII	Family_XIII_UCG-001	uncultured_bacterium_g__Family_XIII_UCG-001	OTU329	20	25	35	11
Firmicutes	Clostridia	Clostridiales	Ruminococcaceae	Ruminiclostridium_5	uncultured_bacterium_g__Ruminiclostridium_5	OTU75	4	35	6	14
Firmicutes	Clostridia	Clostridiales	Peptococcaceae	norank_f__Peptococcaceae	uncultured_organism_g__norank_f__Peptococcaceae	OTU343	0	0	0	0
Firmicutes	Clostridia	Clostridiales	Ruminococcaceae	Anaerofilum	uncultured_bacterium_g__Anaerofilum	OTU660	10	21	6	17
Firmicutes	Clostridia	Clostridiales	Lachnospiraceae	Marvinbryantia	uncultured_bacterium_g__Marvinbryantia	OTU536	1	6	16	20
Firmicutes	Clostridia	Clostridiales	Ruminococcaceae	Anaerotruncus	unclassified_g__Anaerotruncus	OTU10	48	23	45	43
Firmicutes	Clostridia	Clostridiales	Clostridiales_vadinBB6	norank_f__Clostridiales_vadinBB60_	uncultured_bacterium_g__norank_f__Clostridiales_vadinBB60_group	OTU678	7	0	0	12
Firmicutes	Clostridia	Clostridiales	Ruminococcaceae	Ruminiclostridium_5	unclassified_g__Ruminiclostridium_5	OTU84	4	7	10	3
Firmicutes	Clostridia	Clostridiales	unclassified_o__Clostr	unclassified_o__Clostridiales	unclassified_o__Clostridiales	OTU137	9	20	10	18
Tenericutes	Mollicutes	Mollicutes_RF9	norank_o__Mollicutes	norank_o__Mollicutes_RF9	uncultured_bacterium_g__norank_o__Mollicutes_RF9	OTU604	1	2	19	28
Firmicutes	Erysipelotrichia	Erysipelotrichales	Erysipelotrichaceae	norank_f__Erysipelotrichaceae	uncultured_Erysipelotrichaceae_bacterium_g__norank	OTU772	0	0	0	0
Tenericutes	Mollicutes	Anaeroplasmatales	Anaeroplasmataceae	Anaeroplasma	uncultured_bacterium_g__Anaeroplasma	OTU801	25	35	6	9
Firmicutes	Clostridia	Clostridiales	Ruminococcaceae	Ruminococcaceae_UCG-014	unclassified_g__Ruminococcaceae_UCG-014	OTU220	0	0	1	2
Firmicutes	Clostridia	Clostridiales	Ruminococcaceae	Ruminiclostridium_9	unclassified_g__Ruminiclostridium_9	OTU587	12	43	16	21
Firmicutes	Clostridia	Clostridiales	Ruminococcaceae	norank_f__Ruminococcaceae	human_gut_metagenome_g__norank_f__Ruminococcaceae	OTU85	10	13	10	31
Cyanobacteria	Cyanobacteria	Gastranaerophilales	norank_o__Gastranae	norank_o__Gastranaerophilales	uncultured_bacterium_g__norank_o__Gastranaerophilales	OTU502	71	2	0	4
Firmicutes	Clostridia	Clostridiales	Clostridiales_vadinBB6	norank_f__Clostridiales_vadinBB60_	uncultured_bacterium_g__norank_f__Clostridiales_vadinBB60_group	OTU17	30	3	8	23
Firmicutes	Clostridia	Clostridiales	Lachnospiraceae	unclassified_f__Lachnospiraceae	unclassified_f__Lachnospiraceae	OTU513	3	13	4	6
Firmicutes	Clostridia	Clostridiales	Ruminococcaceae	unclassified_f__Ruminococcaceae	unclassified_f__Ruminococcaceae	OTU166	8	7	17	13
Tenericutes	Mollicutes	NB1-n	norank_o__NB1-n	norank_o__NB1-n	uncultured_organism_g__norank_o__NB1-n	OTU603	0	12	12	61
Firmicutes	Clostridia	Clostridiales	Ruminococcaceae	Ruminococcaceae_UCG-013	uncultured_bacterium_g__Ruminococcaceae_UCG-013	OTU720	17	10	45	27
Firmicutes	Clostridia	Clostridiales	Ruminococcaceae	Ruminococcaceae_UCG-014	unclassified_g__Ruminococcaceae_UCG-014	OTU347	4	3	23	23
Firmicutes	Clostridia	Clostridiales	Ruminococcaceae	Ruminiclostridium	uncultured_bacterium_g__Ruminiclostridium	OTU576	15	0	1	2
Cyanobacteria	Cyanobacteria	Gastranaerophilales	norank_o__Gastranae	norank_o__Gastranaerophilales	unclassified_g__norank_o__Gastranaerophilales	OTU563	69	0	0	16
Firmicutes	Clostridia	Clostridiales	Lachnospiraceae	unclassified_f__Lachnospiraceae	unclassified_f__Lachnospiraceae	OTU731	4	47	18	34
Firmicutes	Clostridia	Clostridiales	Lachnospiraceae	Roseburia	unclassified_g__Roseburia	OTU449	0	0	15	26
Firmicutes	Clostridia	Clostridiales	Clostridiales_vadinBB6	norank_f__Clostridiales_vadinBB60_	uncultured_bacterium_g__norank_f__Clostridiales_vadinBB60_group	OTU669	0	0	0	66
Tenericutes	Mollicutes	Mollicutes_RF9	norank_o__Mollicutes	norank_o__Mollicutes_RF9	unclassified_g__norank_o__Mollicutes_RF9	OTU104	1	0	10	40
Firmicutes	Clostridia	Clostridiales	Lachnospiraceae	Lachnospiraceae_UCG-010	uncultured_bacterium_g__Lachnospiraceae_UCG-010	OTU663	11	19	5	23
Firmicutes	Clostridia	Clostridiales	Ruminococcaceae	unclassified_f__Ruminococcaceae	unclassified_f__Ruminococcaceae	OTU847	1	0	19	6
Firmicutes	Clostridia	Clostridiales	Ruminococcaceae	Ruminococcaceae_UCG-013	uncultured_bacterium_g__Ruminococcaceae_UCG-013	OTU721	9	29	11	10
Tenericutes	Mollicutes	Mollicutes_RF9	norank_o__Mollicutes	norank_o__Mollicutes_RF9	uncultured_bacterium_g__norank_o__Mollicutes_RF9	OTU627	0	14	32	18
Firmicutes	Clostridia	Clostridiales	Ruminococcaceae	[Eubacterium]_coprostanoligenes_g	unclassified_g__[Eubacterium]_coprostanoligenes_group	OTU121	1	2	1	0
Firmicutes	Clostridia	Clostridiales	Clostridiales_vadinBB6	norank_f__Clostridiales_vadinBB60_	unclassified_g__norank_f__Clostridiales_vadinBB60_group	OTU180	39	12	9	17
Firmicutes	Clostridia	Clostridiales	Christensenellaceae	Christensenellaceae_R-7_group	unclassified_g__Christensenellaceae_R-7_group	OTU54	0	0	0	2
Firmicutes	Clostridia	Clostridiales	Ruminococcaceae	norank_f__Ruminococcaceae	unclassified_g__norank_f__Ruminococcaceae	OTU534	2	2	6	12
Firmicutes	Clostridia	Clostridiales	Ruminococcaceae	unclassified_f__Ruminococcaceae	unclassified_f__Ruminococcaceae	OTU493	9	38	14	15
Firmicutes	Clostridia	Clostridiales	Lachnospiraceae	norank_f__Lachnospiraceae	unclassified_g__norank_f__Lachnospiraceae	OTU560	6	10	3	18
Firmicutes	Clostridia	Clostridiales	Lachnospiraceae	[Eubacterium]_ventriosum_group	uncultured_organism_g__[Eubacterium]_ventriosum_group	OTU464	6	4	77	33
Tenericutes	Mollicutes	Mollicutes_RF9	norank_o__Mollicutes	norank_o__Mollicutes_RF9	unclassified_g__norank_o__Mollicutes_RF9	OTU234	9	0	11	0
Firmicutes	Clostridia	Clostridiales	Lachnospiraceae	unclassified_f__Lachnospiraceae	unclassified_f__Lachnospiraceae	OTU64	2	5	3	14
Tenericutes	Mollicutes	Mollicutes_RF9	norank_o__Mollicutes	norank_o__Mollicutes_RF9	uncultured_bacterium_g__norank_o__Mollicutes_RF9	OTU602	0	0	0	0
Firmicutes	Clostridia	Clostridiales	Ruminococcaceae	Anaerotruncus	uncultured_bacterium_g__Anaerotruncus	OTU6	17	11	13	0
Firmicutes	Bacilli	Lactobacillales	Lactobacillaceae	Lactobacillus	Lactobacillus_murinus_g__Lactobacillus	OTU843	0	0	0	0
Firmicutes	Clostridia	Clostridiales	Christensenellaceae	Christensenellaceae_R-7_group	uncultured_prokaryote_g__Christensenellaceae_R-7_group	OTU67	8	2	7	15
Firmicutes	Clostridia	Clostridiales	Clostridiales_vadinBB6	norank_f__Clostridiales_vadinBB60_	uncultured_bacterium_g__norank_f__Clostridiales_vadinBB60_group	OTU748	29	1	12	4
Firmicutes	Clostridia	Clostridiales	Lachnospiraceae	Coprococcus_1	unclassified_g__Coprococcus_1	OTU739	2	7	4	6
Tenericutes	Mollicutes	Mollicutes_RF9	norank_o__Mollicutes	norank_o__Mollicutes_RF9	unclassified_g__norank_o__Mollicutes_RF9	OTU229	1	36	23	0
Firmicutes	Clostridia	Clostridiales	Clostridiales_vadinBB6	norank_f__Clostridiales_vadinBB60_	uncultured_bacterium_g__norank_f__Clostridiales_vadinBB60_group	OTU23	212	0	0	1
Firmicutes	Clostridia	Clostridiales	Clostridiales_vadinBB6	norank_f__Clostridiales_vadinBB60_	uncultured_bacterium_g__norank_f__Clostridiales_vadinBB60_group	OTU131	3	0	0	0
Firmicutes	Clostridia	Clostridiales	Ruminococcaceae	Ruminococcaceae_UCG-014	unclassified_g__Ruminococcaceae_UCG-014	OTU837	23	0	46	0
Firmicutes	Clostridia	Clostridiales	unclassified_o__Clostr	unclassified_o__Clostridiales	unclassified_o__Clostridiales	OTU91	15	8	2	4
Firmicutes	Clostridia	Clostridiales	Lachnospiraceae	Roseburia	unclassified_g__Roseburia	OTU43	2	53	1	0
Firmicutes	Clostridia	Clostridiales	Lachnospiraceae	unclassified_f__Lachnospiraceae	unclassified_f__Lachnospiraceae	OTU11	8	5	3	7
Proteobacteria	Betaproteobacteria	Burkholderiales	Oxalobacteraceae	Oxalobacter	unclassified_g__Oxalobacter	OTU557	2	0	0	0
Firmicutes	Clostridia	Clostridiales	Ruminococcaceae	Faecalibacterium	unclassified_g__Faecalibacterium	OTU219	4	27	4	3
Firmicutes	Clostridia	Clostridiales	Ruminococcaceae	Ruminiclostridium_5	uncultured_bacterium_g__Ruminiclostridium_5	OTU59	10	9	10	11
Firmicutes	Clostridia	Clostridiales	Lachnospiraceae	Lachnospiraceae_UCG-010	uncultured_bacterium_g__Lachnospiraceae_UCG-010	OTU755	6	1	17	8
Bacteroidetes	Bacteroidia	Bacteroidales	Rikenellaceae	Alistipes	uncultured_bacterium_g__Alistipes	OTU765	0	0	0	0
Firmicutes	Erysipelotrichia	Erysipelotrichales	Erysipelotrichaceae	[Clostridium]_innocuum_group	uncultured_bacterium_g__[Clostridium]_innocuum_group	OTU794	0	0	0	0
Firmicutes	Clostridia	Clostridiales	unclassified_o__Clostr	unclassified_o__Clostridiales	unclassified_o__Clostridiales	OTU673	17	0	0	49
Firmicutes	Clostridia	Clostridiales	Clostridiales_vadinBB6	norank_f__Clostridiales_vadinBB60_	gut_metagenome_g__norank_f__Clostridiales_vadinBB60_group	OTU548	27	15	2	22
Tenericutes	Mollicutes	Mollicutes_RF9	norank_o__Mollicutes	norank_o__Mollicutes_RF9	uncultured_bacterium_g__norank_o__Mollicutes_RF9	OTU544	0	0	8	14
Cyanobacteria	Cyanobacteria	Gastranaerophilales	norank_o__Gastranae	norank_o__Gastranaerophilales	unclassified_g__norank_o__Gastranaerophilales	OTU155	30	0	15	5
Firmicutes	Clostridia	Clostridiales	Clostridiales_vadinBB6	norank_f__Clostridiales_vadinBB60_	uncultured_bacterium_g__norank_f__Clostridiales_vadinBB60_group	OTU481	24	0	0	0
Firmicutes	Clostridia	Clostridiales	Lachnospiraceae	unclassified_f__Lachnospiraceae	unclassified_f__Lachnospiraceae	OTU467	4	5	7	37
Firmicutes	Clostridia	Clostridiales	Ruminococcaceae	norank_f__Ruminococcaceae	unclassified_g__norank_f__Ruminococcaceae	OTU622	2	49	172	9
Firmicutes	Clostridia	Clostridiales	Lachnospiraceae	unclassified_f__Lachnospiraceae	unclassified_f__Lachnospiraceae	OTU201	1	17	7	13
Firmicutes	Clostridia	Clostridiales	Lachnospiraceae	norank_f__Lachnospiraceae	uncultured_organism_g__norank_f__Lachnospiraceae	OTU654	34	0	24	3
Firmicutes	Clostridia	Clostridiales	Ruminococcaceae	Ruminococcaceae_UCG-014	unclassified_g__Ruminococcaceae_UCG-014	OTU575	15	0	0	0
Tenericutes	Mollicutes	Mollicutes_RF9	norank_o__Mollicutes	norank_o__Mollicutes_RF9	unclassified_g__norank_o__Mollicutes_RF9	OTU600	2	0	12	25
Firmicutes	Clostridia	Clostridiales	Ruminococcaceae	unclassified_f__Ruminococcaceae	unclassified_f__Ruminococcaceae	OTU728	4	17	7	9
Firmicutes	Clostridia	Clostridiales	Ruminococcaceae	Anaerotruncus	unclassified_g__Anaerotruncus	OTU159	4	0	29	17
Tenericutes	Mollicutes	Mollicutes_RF9	norank_o__Mollicutes	norank_o__Mollicutes_RF9	uncultured_bacterium_g__norank_o__Mollicutes_RF9	OTU608	9	0	9	18
Firmicutes	Clostridia	Clostridiales	Ruminococcaceae	Ruminococcaceae_UCG-010	uncultured_bacterium_g__Ruminococcaceae_UCG-010	OTU674	7	3	10	5
Firmicutes	Clostridia	Clostridiales	Lachnospiraceae	Lachnospiraceae_FCS020_group	uncultured_bacterium_g__Lachnospiraceae_FCS020_group	OTU802	17	3	9	16
Firmicutes	Clostridia	Clostridiales	Ruminococcaceae	Caproiciproducens	uncultured_bacterium_g__Caproiciproducens	OTU68	17	15	23	5
Firmicutes	Clostridia	Clostridiales	Peptococcaceae	norank_f__Peptococcaceae	unclassified_g__norank_f__Peptococcaceae	OTU586	0	9	6	12
Firmicutes	Clostridia	Clostridiales	Ruminococcaceae	Anaerotruncus	uncultured_bacterium_g__Anaerotruncus	OTU175	10	10	16	26
Firmicutes	Clostridia	Clostridiales	Lachnospiraceae	unclassified_f__Lachnospiraceae	unclassified_f__Lachnospiraceae	OTU854	0	21	3	0
Firmicutes	Clostridia	Clostridiales	Christensenellaceae	Christensenellaceae_R-7_group	uncultured_bacterium_g__Christensenellaceae_R-7_group	OTU164	0	1	3	3
Firmicutes	Clostridia	Clostridiales	Ruminococcaceae	Ruminococcus_2	uncultured_bacterium_g__Ruminococcus_2	OTU447	0	0	0	0
Actinobacteria	Actinobacteria	Coriobacteriales	Coriobacteriaceae	unclassified_f__Coriobacteriaceae	unclassified_f__Coriobacteriaceae	OTU490	0	16	0	1
Proteobacteria	Alphaproteobacteria	Rhizobiales	Brucellaceae	Brucella	Brucella_melitensis_g__Brucella	OTU812	0	0	0	0
Firmicutes	Clostridia	Clostridiales	Ruminococcaceae	Ruminiclostridium_1	uncultured_bacterium_g__Ruminiclostridium_1	OTU485	0	47	1	7
Tenericutes	Mollicutes	Mollicutes_RF9	norank_o__Mollicutes	norank_o__Mollicutes_RF9	unclassified_g__norank_o__Mollicutes_RF9	OTU611	1	1	3	43
Firmicutes	Clostridia	Clostridiales	Ruminococcaceae	Ruminococcaceae_UCG-014	unclassified_g__Ruminococcaceae_UCG-014	OTU100	5	0	0	0
Firmicutes	Clostridia	Clostridiales	Ruminococcaceae	norank_f__Ruminococcaceae	unclassified_g__norank_f__Ruminococcaceae	OTU98	0	0	0	0
Firmicutes	Bacilli	Lactobacillales	Enterococcaceae	Enterococcus	Enterococcus_faecalis_g__Enterococcus	OTU763	0	0	0	2
Firmicutes	Bacilli	Lactobacillales	Lactobacillaceae	Lactobacillus	Lactobacillus_coleohominis	OTU628	0	0	84	2
Firmicutes	Erysipelotrichia	Erysipelotrichales	Erysipelotrichaceae	unclassified_f__Erysipelotrichaceae	unclassified_f__Erysipelotrichaceae	OTU839	0	16	5	15
Firmicutes	Clostridia	Clostridiales	Christensenellaceae	Christensenellaceae_R-7_group	bacterium_YE57	OTU754	16	12	38	17
Firmicutes	Clostridia	Clostridiales	Ruminococcaceae	Ruminiclostridium_9	unclassified_g__Ruminiclostridium_9	OTU500	4	0	0	1
Firmicutes	Clostridia	Clostridiales	Ruminococcaceae	Faecalibacterium	unclassified_g__Faecalibacterium	OTU846	4	9	10	8
Tenericutes	Mollicutes	Mollicutes_RF9	norank_o__Mollicutes	norank_o__Mollicutes_RF9	unclassified_g__norank_o__Mollicutes_RF9	OTU610	0	0	0	0
Firmicutes	Clostridia	Clostridiales	Ruminococcaceae	norank_f__Ruminococcaceae	unclassified_g__norank_f__Ruminococcaceae	OTU132	2	17	7	0
Firmicutes	Clostridia	Clostridiales	Ruminococcaceae	Ruminococcus_1	uncultured_bacterium_g__Ruminococcus_1	OTU639	0	0	0	0
Firmicutes	Clostridia	Clostridiales	Peptococcaceae	unclassified_f__Peptococcaceae	unclassified_f__Peptococcaceae	OTU483	6	14	1	7
Tenericutes	Mollicutes	Mollicutes_RF9	norank_o__Mollicutes	norank_o__Mollicutes_RF9	uncultured_bacterium_g__norank_o__Mollicutes_RF9	OTU631	0	8	6	34
Firmicutes	Clostridia	Clostridiales	Ruminococcaceae	Caproiciproducens	unclassified_g__Caproiciproducens	OTU824	1	0	0	1
Firmicutes	Clostridia	Clostridiales	Clostridiales_vadinBB6	norank_f__Clostridiales_vadinBB60_	uncultured_bacterium_g__norank_f__Clostridiales_vadinBB60_group	OTU757	0	0	0	0

Tenericutes	Mollicutes	Mollicutes_RF9	norank_o__Mollicutes	norank_o__Mollicutes_RF9	uncultured_bacterium_g__norank_o__Mollicutes_RF9	OTU792	12	33	2	14
Firmicutes	Clostridia	Clostridiales	Lachnospiraceae	Coprococcus_1	uncultured_bacterium_g__Coprococcus_1	OTU629	4	5	47	15
Firmicutes	Clostridia	Clostridiales	Peptococcaceae	Peptococcus	uncultured_organism_g__Peptococcus	OTU665	14	6	4	45
Firmicutes	Clostridia	Clostridiales	Ruminococcaceae	Ruminococcaceae_UCG-014	unclassified_g__Ruminococcaceae_UCG-014	OTU532	0	0	0	1
Firmicutes	Clostridia	Clostridiales	Ruminococcaceae	norank_f__Ruminococcaceae	unclassified_g__norank_f__Ruminococcaceae	OTU671	3	3	4	20
Firmicutes	Clostridia	Clostridiales	Ruminococcaceae	Ruminococcus_1	uncultured_organism_g__Ruminococcus_1	OTU56	7	4	23	9
Firmicutes	Clostridia	Clostridiales	Clostridiales_vadinBB6	norank_f__Clostridiales_vadinBB60_	uncultured_bacterium_g__norank_f__Clostridiales_vadinBB60_group	OTU714	17	10	0	8
Firmicutes	Clostridia	Clostridiales	Lachnospiraceae	unclassified_f__Lachnospiraceae	unclassified_f__Lachnospiraceae	OTU726	0	9	8	8
Firmicutes	Clostridia	Clostridiales	Ruminococcaceae	norank_f__Ruminococcaceae	Ruminococcaceae_bacterium_GD7	OTU72	24	0	12	19
Firmicutes	Erysipelotrichia	Erysipelotrichales	Erysipelotrichaceae	Holdemania	unclassified_g__Holdemania	OTU702	5	8	2	7
Firmicutes	Clostridia	Clostridiales	Ruminococcaceae	[Eubacterium]_coprostanoligenes_g	unclassified_g__[Eubacterium]_coprostanoligenes_group	OTU747	0	0	21	1
Firmicutes	Clostridia	Clostridiales	Family_XIII	Family_XIII_AD3011_group	uncultured_bacterium_g__Family_XIII_AD3011_group	OTU579	0	9	9	7
Tenericutes	Mollicutes	Mollicutes_RF9	norank_o__Mollicutes	norank_o__Mollicutes_RF9	uncultured_bacterium_g__norank_o__Mollicutes_RF9	OTU756	3	0	0	0
Actinobacteria	Actinobacteria	Coriobacteriales	Coriobacteriaceae	unclassified_f__Coriobacteriaceae	unclassified_f__Coriobacteriaceae	OTU4	6	0	1	0
Firmicutes	Clostridia	Clostridiales	Ruminococcaceae	Candidatus_Soleaferrea	uncultured_organism_g__Candidatus_Soleaferrea	OTU492	11	2	14	2
Firmicutes	Clostridia	Clostridiales	Ruminococcaceae	[Eubacterium]_coprostanoligenes_g	unclassified_g__[Eubacterium]_coprostanoligenes_group	OTU656	2	4	20	4
Firmicutes	Erysipelotrichia	Erysipelotrichales	Erysipelotrichaceae	Erysipelatoclostridium	unclassified_g__Erysipelatoclostridium	OTU506	25	0	8	10
Firmicutes	Clostridia	Clostridiales	Ruminococcaceae	Anaerotruncus	uncultured_bacterium_g__Anaerotruncus	OTU42	9	9	8	8
Firmicutes	Clostridia	Clostridiales	Lachnospiraceae	unclassified_f__Lachnospiraceae	unclassified_f__Lachnospiraceae	OTU509	1	1	2	24
Firmicutes	Clostridia	Clostridiales	Lachnospiraceae	Tyzzerella	uncultured_bacterium_g__Tyzzerella	OTU777	20	7	15	9
Firmicutes	Clostridia	Clostridiales	Clostridiales_vadinBB6	norank_f__Clostridiales_vadinBB60_	gut_metagenome_g__norank_f__Clostridiales_vadinBB60_group	OTU19	0	2	0	0
Firmicutes	Clostridia	Clostridiales	Ruminococcaceae	Ruminococcus_2	unclassified_g__Ruminococcus_2	OTU549	61	0	67	7
Firmicutes	Clostridia	Clostridiales	Ruminococcaceae	Anaerofilum	uncultured_bacterium_g__Anaerofilum	OTU677	17	2	6	7
Firmicutes	Clostridia	Clostridiales	Ruminococcaceae	Ruminococcaceae_UCG-014	unclassified_g__Ruminococcaceae_UCG-014	OTU487	3	0	11	15
Firmicutes	Clostridia	Clostridiales	Ruminococcaceae	Ruminococcaceae_UCG-013	unclassified_g__Ruminococcaceae_UCG-013	OTU145	0	1	0	0
Bacteroidetes	Bacteroidia	Bacteroidales	Rikenellaceae	Alistipes	unclassified_g__Alistipes	OTU667	6	5	10	6
Firmicutes	Clostridia	Clostridiales	Lachnospiraceae	unclassified_f__Lachnospiraceae	unclassified_f__Lachnospiraceae	OTU771	0	1	2	4
Firmicutes	Clostridia	Clostridiales	Ruminococcaceae	Ruminococcaceae_UCG-009	uncultured_bacterium_g__Ruminococcaceae_UCG-009	OTU750	1	5	4	0
Tenericutes	Mollicutes	Mollicutes_RF9	norank_o__Mollicutes	norank_o__Mollicutes_RF9	uncultured_bacterium_g__norank_o__Mollicutes_RF9	OTU543	0	0	3	6
Firmicutes	Clostridia	Clostridiales	unclassified_o__Clostr	unclassified_o__Clostridiales	unclassified_o__Clostridiales	OTU535	1	0	6	7
Tenericutes	Mollicutes	Mollicutes_RF9	norank_o__Mollicutes	norank_o__Mollicutes_RF9	uncultured_bacterium_g__norank_o__Mollicutes_RF9	OTU153	1	0	0	9
Firmicutes	Clostridia	Clostridiales	Clostridiales_vadinBB6	norank_f__Clostridiales_vadinBB60_	uncultured_bacterium_g__norank_f__Clostridiales_vadinBB60_group	OTU409	16	12	5	7
Firmicutes	Clostridia	Clostridiales	Ruminococcaceae	Oscillibacter	unclassified_g__Oscillibacter	OTU609	2	28	16	17
Firmicutes	Clostridia	Clostridiales	Ruminococcaceae	Butyricicoccus	unclassified_g__Butyricicoccus	OTU411	10	26	4	2
Firmicutes	Clostridia	Clostridiales	unclassified_o__Clostr	unclassified_o__Clostridiales	unclassified_o__Clostridiales	OTU34	23	7	1	18
Firmicutes	Clostridia	Clostridiales	Clostridiales_vadinBB6	norank_f__Clostridiales_vadinBB60_	uncultured_rumen_bacterium_g__norank_f__Clostridiales_vadinBB60_group	OTU517	2	0	0	16
Firmicutes	Clostridia	Clostridiales	Ruminococcaceae	Ruminococcaceae_UCG-014	uncultured_bacterium_g__Ruminococcaceae_UCG-014	OTU850	7	2	8	1
Firmicutes	Clostridia	Clostridiales	Ruminococcaceae	Ruminiclostridium_5	uncultured_organism_g__Ruminiclostridium_5	OTU541	5	7	16	0
Firmicutes	Clostridia	Clostridiales	Lachnospiraceae	Tyzzerella_3	uncultured_bacterium_g__Tyzzerella_3	OTU635	6	19	26	0
Firmicutes	Clostridia	Clostridiales	Eubacteriaceae	Anaerofustis	Anaerofustis_sp._Marseille-P2441	OTU136	5	10	3	9
Firmicutes	Clostridia	Clostridiales	Ruminococcaceae	Ruminococcaceae_UCG-013	unclassified_g__Ruminococcaceae_UCG-013	OTU76	4	21	1	5
Firmicutes	Clostridia	Clostridiales	Ruminococcaceae	Anaerotruncus	unclassified_g__Anaerotruncus	OTU542	11	5	14	12
Firmicutes	Clostridia	Clostridiales	Ruminococcaceae	unclassified_f__Ruminococcaceae	unclassified_f__Ruminococcaceae	OTU831	4	5	2	1
Firmicutes	Clostridia	Clostridiales	Ruminococcaceae	Ruminococcaceae_NK4A214_group	unclassified_g__Ruminococcaceae_NK4A214_group	OTU168	5	9	5	11
Firmicutes	Clostridia	Clostridiales	Ruminococcaceae	Ruminococcaceae_UCG-009	unclassified_g__Ruminococcaceae_UCG-009	OTU71	0	0	2	27
Firmicutes	Clostridia	Clostridiales	Ruminococcaceae	Ruminococcaceae_UCG-013	uncultured_organism_g__Ruminococcaceae_UCG-013	OTU204	0	0	0	9
Actinobacteria	Actinobacteria	Coriobacteriales	Coriobacteriaceae	Enterorhabdus	uncultured_bacterium_g__Enterorhabdus	OTU221	1	12	0	1
Tenericutes	Mollicutes	Mollicutes_RF9	norank_o__Mollicutes	norank_o__Mollicutes_RF9	unclassified_g__norank_o__Mollicutes_RF9	OTU788	1	0	6	2
Firmicutes	Clostridia	Clostridiales	Ruminococcaceae	Ruminococcaceae_UCG-010	uncultured_organism_g__Ruminococcaceae_UCG-010	OTU20	3	2	3	4
Firmicutes	Clostridia	Clostridiales	Ruminococcaceae	Anaerotruncus	uncultured_bacterium_g__Anaerotruncus	OTU582	0	3	0	2
Firmicutes	Clostridia	Clostridiales	Family_XIII	Family_XIII_AD3011_group	uncultured_bacterium_g__Family_XIII_AD3011_group	OTU634	8	9	8	10
Firmicutes	Clostridia	Clostridiales	Ruminococcaceae	Anaerofilum	uncultured_bacterium_g__Anaerofilum	OTU832	0	0	0	0
Firmicutes	Clostridia	Clostridiales	Clostridiales_vadinBB6	norank_f__Clostridiales_vadinBB60_	uncultured_bacterium_g__norank_f__Clostridiales_vadinBB60_group	OTU38	0	0	0	0
Firmicutes	Clostridia	Clostridiales	unclassified_o__Clostr	unclassified_o__Clostridiales	unclassified_o__Clostridiales	OTU672	30	9	1	19
Firmicutes	Clostridia	Clostridiales	Ruminococcaceae	unclassified_f__Ruminococcaceae	unclassified_f__Ruminococcaceae	OTU661	7	11	2	5
Firmicutes	Clostridia	Clostridiales	Ruminococcaceae	unclassified_f__Ruminococcaceae	unclassified_f__Ruminococcaceae	OTU169	1	3	5	9
Tenericutes	Mollicutes	Mollicutes_RF9	norank_o__Mollicutes	norank_o__Mollicutes_RF9	uncultured_bacterium_g__norank_o__Mollicutes_RF9	OTU820	0	0	6	0
Firmicutes	Clostridia	Clostridiales	Eubacteriaceae	Eubacterium	Eubacterium_limosum	OTU337	5	1	1	0
Tenericutes	Mollicutes	Mollicutes_RF9	norank_o__Mollicutes	norank_o__Mollicutes_RF9	uncultured_bacterium_g__norank_o__Mollicutes_RF9	OTU584	0	11	2	0
Firmicutes	Clostridia	Clostridiales	Ruminococcaceae	Anaerofilum	uncultured_bacterium_g__Anaerofilum	OTU643	4	9	0	1
Firmicutes	Clostridia	Clostridiales	Clostridiales_vadinBB6	norank_f__Clostridiales_vadinBB60_	uncultured_bacterium_g__norank_f__Clostridiales_vadinBB60_group	OTU35	8	0	0	0
Firmicutes	Clostridia	Clostridiales	Lachnospiraceae	Coprococcus_1	uncultured_bacterium_g__Coprococcus_1	OTU811	7	1	2	6
Firmicutes	Clostridia	Clostridiales	Ruminococcaceae	Ruminococcaceae_UCG-014	unclassified_g__Ruminococcaceae_UCG-014	OTU87	1	1	4	18
Firmicutes	Clostridia	Clostridiales	Ruminococcaceae	[Eubacterium]_coprostanoligenes_g	unclassified_g__[Eubacterium]_coprostanoligenes_group	OTU751	0	0	14	1
Firmicutes	Clostridia	Clostridiales	Clostridiales_vadinBB6	norank_f__Clostridiales_vadinBB60_	uncultured_bacterium_g__norank_f__Clostridiales_vadinBB60_group	OTU694	108	0	0	0
Firmicutes	Clostridia	Clostridiales	Clostridiales_vadinBB6	norank_f__Clostridiales_vadinBB60_	uncultured_Thermoanaerobacterales_bacterium_g__norank_f__Clostridiales_vadinBB60_gr	OTU224	0	5	0	7
Firmicutes	Clostridia	Clostridiales	Lachnospiraceae	unclassified_f__Lachnospiraceae	unclassified_f__Lachnospiraceae	OTU655	3	5	11	4
Firmicutes	Clostridia	Clostridiales	Ruminococcaceae	Oscillospira	uncultured_bacterium_g__Oscillospira	OTU574	64	0	3	5
Firmicutes	Clostridia	Clostridiales	Clostridiales_vadinBB6	norank_f__Clostridiales_vadinBB60_	unclassified_g__norank_f__Clostridiales_vadinBB60_group	OTU2	0	0	0	0
Firmicutes	Clostridia	Clostridiales	Clostridiales_vadinBB6	norank_f__Clostridiales_vadinBB60_	unclassified_g__norank_f__Clostridiales_vadinBB60_group	OTU778	0	0	0	0
Firmicutes	Clostridia	Clostridiales	Ruminococcaceae	Ruminococcaceae_UCG-013	unclassified_g__Ruminococcaceae_UCG-013	OTU676	0	0	21	10
Tenericutes	Mollicutes	Mollicutes_RF9	norank_o__Mollicutes	norank_o__Mollicutes_RF9	unclassified_g__norank_o__Mollicutes_RF9	OTU626	5	3	10	6
Firmicutes	Clostridia	Clostridiales	Ruminococcaceae	Ruminococcaceae_UCG-010	unclassified_g__Ruminococcaceae_UCG-010	OTU61	6	7	1	14
Firmicutes	Clostridia	Clostridiales	Ruminococcaceae	norank_f__Ruminococcaceae	unclassified_g__norank_f__Ruminococcaceae	OTU516	4	4	12	3
Firmicutes	Clostridia	Clostridiales	Ruminococcaceae	unclassified_f__Ruminococcaceae	unclassified_f__Ruminococcaceae	OTU228	5	4	9	10
Firmicutes	Clostridia	Clostridiales	Ruminococcaceae	Ruminococcaceae_UCG-010	uncultured_bacterium_g__Ruminococcaceae_UCG-010	OTU203	0	0	0	0
Firmicutes	Clostridia	Clostridiales	Ruminococcaceae	Anaerotruncus	uncultured_bacterium_g__Anaerotruncus	OTU853	0	14	12	3
Firmicutes	Clostridia	Thermoanaerobacte	Thermoanaerobactera	Gelria	uncultured_bacterium_g__Gelria	OTU16	7	1	5	1
Firmicutes	Clostridia	Clostridiales	Clostridiales_vadinBB6	norank_f__Clostridiales_vadinBB60_	uncultured_bacterium_g__norank_f__Clostridiales_vadinBB60_group	OTU143	0	0	0	1
Firmicutes	Clostridia	Clostridiales	Family_XIII	unclassified_f__Family_XIII	unclassified_f__Family_XIII	OTU537	3	5	1	1
Firmicutes	Clostridia	Clostridiales	Family_XIII	[Eubacterium]_brachy_group	uncultured_bacterium_g__[Eubacterium]_brachy_group	OTU69	3	2	3	6
Firmicutes	Clostridia	Clostridiales	Ruminococcaceae	unclassified_f__Ruminococcaceae	unclassified_f__Ruminococcaceae	OTU14	5	6	2	18
Firmicutes	Clostridia	Clostridiales	Ruminococcaceae	Intestinimonas	uncultured_bacterium_g__Intestinimonas	OTU615	4	6	5	5
Firmicutes	Clostridia	Clostridiales	Lachnospiraceae	unclassified_f__Lachnospiraceae	unclassified_f__Lachnospiraceae	OTU118	1	2	3	4
Firmicutes	Clostridia	Clostridiales	Ruminococcaceae	Anaerotruncus	unclassified_g__Anaerotruncus	OTU505	0	1	10	0
Firmicutes	Bacilli	Lactobacillales	Lactobacillaceae	Lactobacillus	uncultured_bacterium_g__Lactobacillus	OTU637	0	0	22	0
Firmicutes	Clostridia	Clostridiales	Ruminococcaceae	Ruminococcaceae_V9D2013_group	uncultured_bacterium_g__Ruminococcaceae_V9D2013_group	OTU652	1	6	2	5
Firmicutes	Erysipelotrichia	Erysipelotrichales	Erysipelotrichaceae	norank_f__Erysipelotrichaceae	uncultured_Erysipelotrichales_bacterium_g__norank	OTU323	0	0	0	0
Firmicutes	Clostridia	Clostridiales	Ruminococcaceae	Papillibacter	uncultured_bacterium_g__Papillibacter	OTU715	3	5	5	4
Tenericutes	Mollicutes	Mollicutes_RF9	norank_o__Mollicutes	norank_o__Mollicutes_RF9	unclassified_g__norank_o__Mollicutes_RF9	OTU729	7	0	6	9
Cyanobacteria	Cyanobacteria	Gastranaerophilales	norank_o__Gastranae	norank_o__Gastranaerophilales	uncultured_bacterium_g__norank_o__Gastranaerophilales	OTU94	0	0	0	0
Firmicutes	Clostridia	Clostridiales	Ruminococcaceae	Ruminococcaceae_UCG-013	uncultured_bacterium_g__Ruminococcaceae_UCG-013	OTU780	2	1	29	7
Firmicutes	Clostridia	Clostridiales	Ruminococcaceae	Anaerotruncus	unclassified_g__Anaerotruncus	OTU723	4	4	5	5
Firmicutes	Clostridia	Clostridiales	Ruminococcaceae	Anaerotruncus	unclassified_g__Anaerotruncus	OTU101	4	10	3	2
Tenericutes	Mollicutes	Mollicutes_RF9	norank_o__Mollicutes	norank_o__Mollicutes_RF9	unclassified_g__norank_o__Mollicutes_RF9	OTU117	0	0	0	6
Firmicutes	Clostridia	Clostridiales	Ruminococcaceae	unclassified_f__Ruminococcaceae	unclassified_f__Ruminococcaceae	OTU93	0	0	4	3
Firmicutes	Clostridia	Clostridiales	Lachnospiraceae	unclassified_f__Lachnospiraceae	unclassified_f__Lachnospiraceae	OTU759	1	2	1	3
Firmicutes	Clostridia	Clostridiales	Ruminococcaceae	Ruminococcaceae_UCG-014	unclassified_g__Ruminococcaceae_UCG-014	OTU95	0	0	0	0
Firmicutes	Erysipelotrichia	Erysipelotrichales	Erysipelotrichaceae	Dielma	unclassified_g__Dielma	OTU173	0	0	0	0
Firmicutes	Clostridia	Clostridiales	Ruminococcaceae	Anaerotruncus	unclassified_g__Anaerotruncus	OTU124	15	5	2	3
Firmicutes	Clostridia	Clostridiales	Ruminococcaceae	norank_f__Ruminococcaceae	unclassified_g__norank_f__Ruminococcaceae	OTU134	2	3	3	2
Firmicutes	Clostridia	Clostridiales	Lachnospiraceae	unclassified_f__Lachnospiraceae	unclassified_f__Lachnospiraceae	OTU510	6	1	4	4
Actinobacteria	Actinobacteria	Coriobacteriales	Coriobacteriaceae	Gordonibacter	unclassified_g__Gordonibacter	OTU461	5	5	5	3
Proteobacteria	Betaproteobacteria	Burkholderiales	Burkholderiaceae	Burkholderia-Paraburkholderia	unclassified_g__Burkholderia-Paraburkholderia	OTU435	0	0	0	0
Firmicutes	Clostridia	Clostridiales	Family_XIII	Family_XIII_AD3011_group	uncultured_bacterium_g__Family_XIII_AD3011_group	OTU648	0	0	0	1
Firmicutes	Clostridia	Clostridiales	Ruminococcaceae	unclassified_f__Ruminococcaceae	unclassified_f__Ruminococcaceae	OTU722	6	0	0	0
Firmicutes	Clostridia	Clostridiales	Clostridiales_vadinBB6	norank_f__Clostridiales_vadinBB60_	uncultured_bacterium_g__norank_f__Clostridiales_vadinBB60_group	OTU740	0	3	0	0
Bacteroidetes	Bacteroidia	Bacteroidales	Bacteroidales_S24-7_g	norank_f__Bacteroidales_S24-7_gro	uncultured_bacterium_g__norank_f__Bacteroidales_S24-7_group	OTU322	0	0	0	0
Firmicutes	Clostridia	Clostridiales	Ruminococcaceae	unclassified_f__Ruminococcaceae	unclassified_f__Ruminococcaceae	OTU538	1	4	7	2
Firmicutes	Clostridia	Clostridiales	Lachnospiraceae	unclassified_f__Lachnospiraceae	unclassified_f__Lachnospiraceae	OTU255	5	0	2	2
Firmicutes	Clostridia	Clostridiales	Christensenellaceae	Christensenellaceae_R-7_group	unclassified_g__Christensenellaceae_R-7_group	OTU106	0	0	0	4

Firmicutes	Clostridia	Clostridiales	Ruminococcaceae	[Eubacterium]_coprostanoligenes_g	uncultured_bacterium_g__[Eubacterium]_coprostanoligenes_group	OTU129	0	0	5	2
Firmicutes	Erysipelotrichia	Erysipelotrichales	Erysipelotrichaceae	Erysipelatoclostridium	Clostridiales_bacterium_CHKCI006	OTU232	3	0	0	0
Firmicutes	Clostridia	Clostridiales	Ruminococcaceae	unclassified_f__Ruminococcaceae	unclassified_f__Ruminococcaceae	OTU518	4	1	3	0
Firmicutes	Clostridia	Clostridiales	Ruminococcaceae	Ruminococcaceae_UCG-013	uncultured_Clostridiaceae_bacterium_g__Ruminococcaceae_UCG-013	OTU675	4	4	1	9
Firmicutes	Clostridia	Clostridiales	Christensenellaceae	Christensenellaceae_R-7_group	human_gut_metagenome_g__Christensenellaceae_R-7_group	OTU223	0	0	0	0
Firmicutes	Clostridia	Clostridiales	Ruminococcaceae	Faecalibacterium	uncultured_bacterium_g__Faecalibacterium	OTU41	11	0	0	1
Firmicutes	Clostridia	Clostridiales	Ruminococcaceae	Anaerotruncus	unclassified_g__Anaerotruncus	OTU647	0	0	0	1
Firmicutes	Erysipelotrichia	Erysipelotrichales	Erysipelotrichaceae	Turicibacter	uncultured_bacterium_g__Turicibacter	OTU598	0	0	0	0
Firmicutes	Clostridia	Clostridiales	Ruminococcaceae	Subdoligranulum	unclassified_g__Subdoligranulum	OTU188	1	1	1	0
Firmicutes	Clostridia	Clostridiales	Ruminococcaceae	Anaerotruncus	uncultured_Clostridiaceae_bacterium_g__Anaerotruncus	OTU235	2	3	1	4
Firmicutes	Clostridia	Clostridiales	Family_XIII	unclassified_f__Family_XIII	unclassified_f__Family_XIII	OTU236	0	5	2	3
Firmicutes	Clostridia	Clostridiales	Ruminococcaceae	Ruminococcaceae_UCG-014	unidentified_rumen_bacterium_JW32	OTU21	1	0	0	0
Firmicutes	Clostridia	Clostridiales	Ruminococcaceae	Papillibacter	unclassified_g__Papillibacter	OTU521	0	0	0	8
Firmicutes	Clostridia	Clostridiales	Ruminococcaceae	unclassified_f__Ruminococcaceae	unclassified_f__Ruminococcaceae	OTU653	1	5	3	2
Firmicutes	Clostridia	Clostridiales	Peptococcaceae	norank_f__Peptococcaceae	uncultured_bacterium_g__norank_f__Peptococcaceae	OTU605	1	0	0	6
Firmicutes	Bacilli	Lactobacillales	Lactobacillaceae	Lactobacillus	Lactobacillus_ingluviei	OTU182	0	0	0	0
Firmicutes	Clostridia	Clostridiales	Ruminococcaceae	[Eubacterium]_coprostanoligenes_g	unclassified_g__[Eubacterium]_coprostanoligenes_group	OTU333	0	0	0	0
Firmicutes	Clostridia	Clostridiales	unclassified_o__Clostr	unclassified_o__Clostridiales	unclassified_o__Clostridiales	OTU472	1	2	0	2
Firmicutes	Clostridia	Clostridiales	Ruminococcaceae	[Eubacterium]_coprostanoligenes_g	unclassified_g__[Eubacterium]_coprostanoligenes_group	OTU730	4	4	15	0
Firmicutes	Clostridia	Clostridiales	Peptococcaceae	norank_f__Peptococcaceae	unclassified_g__norank_f__Peptococcaceae	OTU172	0	0	1	0
Firmicutes	Clostridia	Clostridiales	Ruminococcaceae	Ruminococcaceae_UCG-013	uncultured_bacterium_g__Ruminococcaceae_UCG-013	OTU658	0	0	0	112
Proteobacteria	Gammaproteobacteria	Enterobacteriales	Enterobacteriaceae	Proteus	Proteus_mirabilis	OTU785	0	0	2	0
Firmicutes	Clostridia	Clostridiales	Ruminococcaceae	norank_f__Ruminococcaceae	unclassified_g__norank_f__Ruminococcaceae	OTU744	2	1	1	0
Firmicutes	Clostridia	Clostridiales	Lachnospiraceae	Tyzzerella_3	unclassified_g__Tyzzerella_3	OTU841	9	0	0	0
Bacteroidetes	Bacteroidia	Bacteroidales	Bacteroidales_S24-7_g	norank_f__Bacteroidales_S24-7_gro	uncultured_bacterium_g__norank_f__Bacteroidales_S24-7_group	OTU360	0	0	0	0
Firmicutes	Clostridia	Clostridiales	Ruminococcaceae	unclassified_f__Ruminococcaceae	unclassified_f__Ruminococcaceae	OTU737	4	0	2	5
Firmicutes	Clostridia	Clostridiales	Ruminococcaceae	Anaerotruncus	unclassified_g__Anaerotruncus	OTU752	6	2	6	3
Firmicutes	Clostridia	Clostridiales	Lachnospiraceae	unclassified_f__Lachnospiraceae	unclassified_f__Lachnospiraceae	OTU63	3	9	2	0
Firmicutes	Clostridia	Clostridiales	Ruminococcaceae	Caproiciproducens	unclassified_g__Caproiciproducens	OTU452	4	1	17	0
Firmicutes	Clostridia	Clostridiales	Ruminococcaceae	Butyricicoccus	unclassified_g__Butyricicoccus	OTU475	0	0	0	1
Proteobacteria	Betaproteobacteria	Burkholderiales	Oxalobacteraceae	Herminiimonas	uncultured_bacterium_g__Herminiimonas	OTU804	2	4	0	6
Tenericutes	Mollicutes	Mollicutes_RF9	norank_o__Mollicutes	norank_o__Mollicutes_RF9	unclassified_g__norank_o__Mollicutes_RF9	OTU630	2	0	6	15
Firmicutes	Clostridia	Clostridiales	Ruminococcaceae	Ruminococcaceae_UCG-013	unclassified_g__Ruminococcaceae_UCG-013	OTU680	1	6	3	5
Tenericutes	Mollicutes	Mollicutes_RF9	norank_o__Mollicutes	norank_o__Mollicutes_RF9	gut_metagenome_g__norank_o__Mollicutes_RF9	OTU773	0	0	0	5
Firmicutes	Clostridia	Clostridiales	Peptococcaceae	norank_f__Peptococcaceae	uncultured_bacterium_g__norank_f__Peptococcaceae	OTU624	3	4	3	1
Firmicutes	Clostridia	Clostridiales	Ruminococcaceae	Ruminiclostridium	unclassified_g__Ruminiclostridium	OTU154	2	3	1	3
Bacteroidetes	Bacteroidia	Bacteroidales	Bacteroidales_S24-7_g	norank_f__Bacteroidales_S24-7_gro	unclassified_g__norank_f__Bacteroidales_S24-7_group	OTU184	0	0	0	0
Firmicutes	Clostridia	Clostridiales	Ruminococcaceae	Ruminococcaceae_UCG-014	unclassified_g__Ruminococcaceae_UCG-014	OTU524	0	0	0	1
Firmicutes	Clostridia	Clostridiales	Peptococcaceae	norank_f__Peptococcaceae	uncultured_bacterium_g__norank_f__Peptococcaceae	OTU115	1	0	4	4
Firmicutes	Clostridia	Clostridiales	Lachnospiraceae	unclassified_f__Lachnospiraceae	unclassified_f__Lachnospiraceae	OTU127	0	0	2	14
Firmicutes	Clostridia	Clostridiales	Lachnospiraceae	unclassified_f__Lachnospiraceae	unclassified_f__Lachnospiraceae	OTU664	0	4	5	8
Firmicutes	Clostridia	Clostridiales	Lachnospiraceae	unclassified_f__Lachnospiraceae	unclassified_f__Lachnospiraceae	OTU465	1	2	4	0
Firmicutes	Clostridia	Clostridiales	Ruminococcaceae	Ruminococcaceae_UCG-005	uncultured_Clostridiales_bacterium_g__Ruminococcaceae_UCG-005	OTU689	1	2	0	1
Tenericutes	Mollicutes	Mollicutes_RF9	norank_o__Mollicutes	norank_o__Mollicutes_RF9	uncultured_bacterium_g__norank_o__Mollicutes_RF9	OTU52	1	0	0	5
Firmicutes	Clostridia	Clostridiales	Ruminococcaceae	[Eubacterium]_coprostanoligenes_g	unclassified_g__[Eubacterium]_coprostanoligenes_group	OTU74	3	3	3	1
Firmicutes	Clostridia	Clostridiales	Lachnospiraceae	Coprococcus_1	unclassified_g__Coprococcus_1	OTU659	3	3	0	4
Firmicutes	Clostridia	Clostridiales	Lachnospiraceae	unclassified_f__Lachnospiraceae	unclassified_f__Lachnospiraceae	OTU688	0	0	4	0
Firmicutes	Clostridia	Clostridiales	Ruminococcaceae	unclassified_f__Ruminococcaceae	unclassified_f__Ruminococcaceae	OTU732	1	0	0	0
Firmicutes	Clostridia	Clostridiales	Christensenellaceae	Christensenellaceae_R-7_group	uncultured_organism_g__Christensenellaceae_R-7_group	OTU684	3	8	0	3
Bacteroidetes	Bacteroidia	Bacteroidales	Porphyromonadaceae	Odoribacter	uncultured_bacterium_g__Odoribacter	OTU327	0	0	0	0
Firmicutes	Clostridia	Clostridiales	Clostridiales_vadinBB6	norank_f__Clostridiales_vadinBB60_	uncultured_bacterium_g__norank_f__Clostridiales_vadinBB60_group	OTU325	13	1	1	0
Firmicutes	Clostridia	Clostridiales	Lachnospiraceae	unclassified_f__Lachnospiraceae	unclassified_f__Lachnospiraceae	OTU551	11	0	0	1
Firmicutes	Bacilli	Lactobacillales	Enterococcaceae	Enterococcus	unclassified_g__Enterococcus	OTU237	1	0	0	0
Bacteroidetes	Bacteroidia	Bacteroidales	Prevotellaceae	Alloprevotella	gut_metagenome_g__Alloprevotella	OTU312	0	0	0	0
Firmicutes	Clostridia	Clostridiales	Ruminococcaceae	unclassified_f__Ruminococcaceae	unclassified_f__Ruminococcaceae	OTU855	1	1	0	1
Actinobacteria	Actinobacteria	Bifidobacteriales	Bifidobacteriaceae	Bifidobacterium	Bifidobacterium_pseudolongum_PV8-2	OTU468	0	0	0	0
Firmicutes	Clostridia	Clostridiales	Christensenellaceae	norank_f__Christensenellaceae	unclassified_g__norank_f__Christensenellaceae	OTU529	0	5	1	0
Tenericutes	Mollicutes	Mollicutes_RF9	norank_o__Mollicutes	norank_o__Mollicutes_RF9	unclassified_g__norank_o__Mollicutes_RF9	OTU764	0	2	0	5
Firmicutes	Clostridia	Clostridiales	Ruminococcaceae	norank_f__Ruminococcaceae	unclassified_g__norank_f__Ruminococcaceae	OTU805	2	0	6	4
Firmicutes	Clostridia	Clostridiales	Ruminococcaceae	Faecalibacterium	unclassified_g__Faecalibacterium	OTU60	1	5	1	6
Firmicutes	Clostridia	Clostridiales	Ruminococcaceae	unclassified_f__Ruminococcaceae	unclassified_f__Ruminococcaceae	OTU530	3	4	1	2
Firmicutes	Clostridia	Clostridiales	Peptococcaceae	norank_f__Peptococcaceae	uncultured_bacterium_g__norank_f__Peptococcaceae	OTU619	0	0	9	0
Firmicutes	Clostridia	Clostridiales	Ruminococcaceae	unclassified_f__Ruminococcaceae	unclassified_f__Ruminococcaceae	OTU471	7	0	0	0
Firmicutes	Clostridia	Clostridiales	Ruminococcaceae	Candidatus_Soleaferrea	uncultured_bacterium_g__Candidatus_Soleaferrea	OTU284	0	3	3	6
Firmicutes	Clostridia	Clostridiales	Ruminococcaceae	Anaerotruncus	unclassified_g__Anaerotruncus	OTU508	12	0	1	0
Firmicutes	Clostridia	Clostridiales	Ruminococcaceae	Anaerotruncus	uncultured_bacterium_g__Anaerotruncus	OTU704	3	6	3	2
Tenericutes	Mollicutes	Mollicutes_RF9	norank_o__Mollicutes	norank_o__Mollicutes_RF9	uncultured_bacterium_g__norank_o__Mollicutes_RF9	OTU783	0	0	0	0
Firmicutes	Clostridia	Clostridiales	Eubacteriaceae	Anaerofustis	uncultured_bacterium_g__Anaerofustis	OTU111	0	0	0	0
Firmicutes	Clostridia	Clostridiales	Clostridiales_vadinBB6	norank_f__Clostridiales_vadinBB60_	unclassified_g__norank_f__Clostridiales_vadinBB60_group	OTU645	4	0	5	1
Firmicutes	Clostridia	Clostridiales	Peptococcaceae	norank_f__Peptococcaceae	uncultured_bacterium_g__norank_f__Peptococcaceae	OTU27	0	3	1	1
Firmicutes	Clostridia	Clostridiales	Ruminococcaceae	Anaerotruncus	unclassified_g__Anaerotruncus	OTU809	3	0	1	1
Bacteroidetes	Bacteroidia	Bacteroidales	Rikenellaceae	Rikenellaceae_RC9_gut_group	unclassified_g__Rikenellaceae_RC9_gut_group	OTU321	0	0	0	0
Firmicutes	Clostridia	Clostridiales	Ruminococcaceae	Ruminococcaceae_UCG-005	unclassified_g__Ruminococcaceae_UCG-005	OTU482	0	0	1	2
Tenericutes	Mollicutes	Mollicutes_RF9	norank_o__Mollicutes	norank_o__Mollicutes_RF9	uncultured_Erysipelotrichaceae_bacterium_g__norank_o__Mollicutes_RF9	OTU558	1	0	2	1
Firmicutes	Clostridia	Clostridiales	Clostridiaceae_1	Candidatus_Arthromitus	Candidatus_Arthromitus_sp._SFB-mouse-Japan	OTU162	0	0	1	0
Firmicutes	Clostridia	Clostridiales	Ruminococcaceae	Subdoligranulum	unclassified_g__Subdoligranulum	OTU810	0	0	0	0
Firmicutes	Clostridia	Clostridiales	Ruminococcaceae	norank_f__Ruminococcaceae	unclassified_g__norank_f__Ruminococcaceae	OTU666	6	1	1	1
Firmicutes	Clostridia	Clostridiales	Ruminococcaceae	unclassified_f__Ruminococcaceae	unclassified_f__Ruminococcaceae	OTU693	0	2	0	0
Firmicutes	Clostridia	Clostridiales	Ruminococcaceae	unclassified_f__Ruminococcaceae	unclassified_f__Ruminococcaceae	OTU858	3	2	2	1
Firmicutes	Clostridia	Clostridiales	Ruminococcaceae	Ruminiclostridium_5	unclassified_g__Ruminiclostridium_5	OTU470	1	3	0	1
Firmicutes	Negativicutes	Selenomonadales	Acidaminococcaceae	Phascolarctobacterium	uncultured_organism_g__Phascolarctobacterium	OTU787	0	0	0	0
Firmicutes	Clostridia	Clostridiales	Christensenellaceae	Christensenellaceae_R-7_group	unclassified_g__Christensenellaceae_R-7_group	OTU745	1	1	2	4
Firmicutes	Clostridia	Clostridiales	Peptococcaceae	norank_f__Peptococcaceae	unclassified_g__norank_f__Peptococcaceae	OTU495	1	0	0	0
Firmicutes	Clostridia	Clostridiales	Ruminococcaceae	Ruminococcaceae_UCG-010	uncultured_bacterium_g__Ruminococcaceae_UCG-010	OTU18	1	0	1	1
Firmicutes	Clostridia	Clostridiales	Ruminococcaceae	Ruminococcaceae_UCG-013	unclassified_g__Ruminococcaceae_UCG-013	OTU89	0	0	0	0
Firmicutes	Clostridia	Clostridiales	Ruminococcaceae	Ruminiclostridium_5	unclassified_g__Ruminiclostridium_5	OTU649	1	2	0	2
Firmicutes	Clostridia	Clostridiales	Lachnospiraceae	unclassified_f__Lachnospiraceae	unclassified_f__Lachnospiraceae	OTU324	1	0	1	1
Firmicutes	Clostridia	Clostridiales	Ruminococcaceae	Ruminococcaceae_UCG-013	unclassified_g__Ruminococcaceae_UCG-013	OTU31	0	0	5	1
Firmicutes	Clostridia	Clostridiales	Ruminococcaceae	Ruminococcus_1	uncultured_organism_g__Ruminococcus_1	OTU651	0	0	0	0
Firmicutes	Clostridia	Clostridiales	Peptococcaceae	unclassified_f__Peptococcaceae	unclassified_f__Peptococcaceae	OTU768	1	3	5	1
Firmicutes	Clostridia	Clostridiales	Christensenellaceae	unclassified_f__Christensenellaceae	unclassified_f__Christensenellaceae	OTU857	0	0	2	0
Tenericutes	Mollicutes	Mollicutes_RF9	norank_o__Mollicutes	norank_o__Mollicutes_RF9	unclassified_g__norank_o__Mollicutes_RF9	OTU758	0	0	1	2
Firmicutes	Clostridia	Clostridiales	Ruminococcaceae	Candidatus_Soleaferrea	uncultured_bacterium_g__Candidatus_Soleaferrea	OTU791	1	4	0	1
unclassified_k__r	unclassified_k__norank	unclassified_k__nor	unclassified_k__noran	unclassified_k__norank	unclassified_k__norank	OTU9	0	1	2	0
Firmicutes	Clostridia	Clostridiales	Ruminococcaceae	unclassified_f__Ruminococcaceae	unclassified_f__Ruminococcaceae	OTU733	0	0	0	0
Firmicutes	Clostridia	Clostridiales	Peptococcaceae	norank_f__Peptococcaceae	unclassified_g__norank_f__Peptococcaceae	OTU90	0	1	0	2
Firmicutes	Clostridia	Clostridiales	Lachnospiraceae	unclassified_f__Lachnospiraceae	unclassified_f__Lachnospiraceae	OTU644	0	0	0	0
Firmicutes	Clostridia	Clostridiales	Ruminococcaceae	[Eubacterium]_coprostanoligenes_g	unclassified_g__[Eubacterium]_coprostanoligenes_group	OTU29	1	3	3	1
Firmicutes	Clostridia	Clostridiales	Ruminococcaceae	Ruminococcaceae_UCG-013	uncultured_Clostridiaceae_bacterium_g__Ruminococcaceae_UCG-013	OTU152	0	0	0	0
Firmicutes	Clostridia	Clostridiales	Ruminococcaceae	Faecalibacterium	unclassified_g__Faecalibacterium	OTU243	0	2	0	2
Bacteroidetes	Bacteroidia	Bacteroidales	Bacteroidales_S24-7_g	norank_f__Bacteroidales_S24-7_gro	uncultured_Bacteroidales_bacterium_g__norank_f__Bacteroidales_S24-7_group	OTU339	0	0	0	0
Tenericutes	Mollicutes	Mollicutes_RF9	norank_o__Mollicutes	norank_o__Mollicutes_RF9	unclassified_g__norank_o__Mollicutes_RF9	OTU51	0	0	0	0
Firmicutes	Clostridia	Clostridiales	Christensenellaceae	Christensenellaceae_R-7_group	uncultured_organism_g__Christensenellaceae_R-7_group	OTU332	0	0	0	0
Firmicutes	Clostridia	Clostridiales	Ruminococcaceae	Anaerotruncus	unclassified_g__Anaerotruncus	OTU194	0	3	5	0
Firmicutes	Clostridia	Clostridiales	Ruminococcaceae	Ruminococcaceae_UCG-013	uncultured_bacterium_g__Ruminococcaceae_UCG-013	OTU334	0	0	0	0
Firmicutes	Clostridia	Clostridiales	Ruminococcaceae	Ruminococcaceae_NK4A214_group	unclassified_g__Ruminococcaceae_NK4A214_group	OTU32	0	0	1	0
Firmicutes	Clostridia	Clostridiales	Christensenellaceae	Christensenellaceae_R-7_group	unclassified_g__Christensenellaceae_R-7_group	OTU105	2	1	1	2
Firmicutes	Bacilli	Bacillales	Staphylococcaceae	Staphylococcus	Staphylococcus_warneri	OTU597	0	0	1	2
Firmicutes	Bacilli	Bacillales	Staphylococcaceae	Staphylococcus	Staphylococcus_lentus_g__Staphylococcus	OTU432	0	0	0	0
Firmicutes	Clostridia	Clostridiales	Ruminococcaceae	unclassified_f__Ruminococcaceae	unclassified_f__Ruminococcaceae	OTU698	0	0	0	0
Tenericutes	Mollicutes	Mollicutes_RF9	norank_o__Mollicutes	norank_o__Mollicutes_RF9	unclassified_g__norank_o__Mollicutes_RF9	OTU781	0	0	0	0

Firmicutes	Clostridia	Clostridiales	Ruminococcaceae	Anaerotruncus	uncultured_bacterium_g__Anaerotruncus	OTU53	0	1	1	1
Actinobacteria	Actinobacteria	Micrococcales	Intrasporangiaceae	Terrabacter	uncultured_bacterium_g__Terrabacter	OTU270	0	0	0	0
Firmicutes	Clostridia	Clostridiales	Ruminococcaceae	unclassified_f__Ruminococcaceae	unclassified_f__Ruminococcaceae	OTU681	0	3	1	1
Bacteroidetes	Bacteroidia	Bacteroidales	Bacteroidales_S24-7_g	norank_f__Bacteroidales_S24-7_gro	uncultured_bacterium_g__norank_f__Bacteroidales_S24-7_group	OTU310	0	0	0	0
Firmicutes	Clostridia	Clostridiales	Clostridiales_vadinBB6	norank_f__Clostridiales_vadinBB60_	uncultured_bacterium_g__norank_f__Clostridiales_vadinBB60_group	OTU528	0	0	0	0
Firmicutes	Clostridia	Clostridiales	Ruminococcaceae	Ruminiclostridium	uncultured_bacterium_g__Ruminiclostridium	OTU633	0	0	3	0
Firmicutes	Clostridia	Clostridiales	Christensenellaceae	Christensenellaceae_R-7_group	unclassified_g__Christensenellaceae_R-7_group	OTU695	5	1	1	0
Cyanobacteria	Cyanobacteria	norank_c__Cyanoba	norank_c__Cyanobact	norank_c__Cyanobacteria	unclassified_g__norank_c__Cyanobacteria	OTU258	0	0	0	0
Bacteroidetes	Bacteroidia	Bacteroidales	Prevotellaceae	Prevotellaceae_UCG-001	uncultured_Bacteroidales_bacterium_g__Prevotellaceae_UCG-001	OTU276	0	0	0	0
Firmicutes	Bacilli	Lactobacillales	Lactobacillaceae	Lactobacillus	unclassified_g__Lactobacillus	OTU632	0	1	13	1
Firmicutes	Clostridia	Clostridiales	Ruminococcaceae	Ruminiclostridium_9	Clostridiales_bacterium_NK3B98_g__Ruminiclostridium_9	OTU130	0	0	0	0
Firmicutes	Clostridia	Clostridiales	Ruminococcaceae	Ruminococcaceae_UCG-013	uncultured_bacterium_g__Ruminococcaceae_UCG-013	OTU807	0	0	0	0
Bacteroidetes	Bacteroidia	Bacteroidales	Rikenellaceae	Alistipes	gut_metagenome_g__Alistipes	OTU808	0	1	0	1
Firmicutes	Clostridia	unclassified_c__Clo	unclassified_c__Clostr	unclassified_c__Clostridia	unclassified_c__Clostridia	OTU99	0	0	3	0
Firmicutes	Clostridia	Clostridiales	Christensenellaceae	norank_f__Christensenellaceae	unclassified_g__norank_f__Christensenellaceae	OTU553	0	0	0	0
Firmicutes	Clostridia	Clostridiales	Lachnospiraceae	unclassified_f__Lachnospiraceae	unclassified_f__Lachnospiraceae	OTU80	0	1	1	0
Bacteroidetes	Bacteroidia	Bacteroidales	Prevotellaceae	Paraprevotella	unclassified_g__Paraprevotella	OTU308	0	0	0	0
Firmicutes	Clostridia	Clostridiales	Peptococcaceae	norank_f__Peptococcaceae	unclassified_g__norank_f__Peptococcaceae	OTU596	0	0	0	0
Firmicutes	Clostridia	Clostridiales	Lachnospiraceae	Lachnospiraceae_NK4A136_group	unclassified_g__Lachnospiraceae_NK4A136_group	OTU251	0	0	0	0
Firmicutes	Clostridia	Clostridiales	Family_XIII	[Eubacterium]_brachy_group	uncultured_bacterium_g__[Eubacterium]_brachy_group	OTU335	0	0	0	0
Firmicutes	Clostridia	Clostridiales	Lachnospiraceae	unclassified_f__Lachnospiraceae	unclassified_f__Lachnospiraceae	OTU86	0	1	5	0
Firmicutes	Clostridia	Clostridiales	Ruminococcaceae	Ruminococcaceae_UCG-005	uncultured_bacterium_adhufec311	OTU215	0	0	0	0
Firmicutes	Clostridia	Clostridiales	Ruminococcaceae	Ruminococcaceae_UCG-009	uncultured_bacterium_g__Ruminococcaceae_UCG-009	OTU641	0	0	0	0
Firmicutes	Clostridia	Clostridiales	Ruminococcaceae	Ruminiclostridium_1	unclassified_g__Ruminiclostridium_1	OTU178	1	0	2	0
Actinobacteria	Actinobacteria	Micrococcales	Micrococcaceae	Pseudarthrobacter	unclassified_g__Pseudarthrobacter	OTU423	0	0	0	0
Firmicutes	Clostridia	Clostridiales	Ruminococcaceae	Ruminococcaceae_UCG-010	unclassified_g__Ruminococcaceae_UCG-010	OTU26	1	0	0	5
Firmicutes	Clostridia	Clostridiales	Clostridiaceae_1	Clostridium_sensu_stricto_1	uncultured_bacterium_g__Clostridium_sensu_stricto_1	OTU128	0	0	0	0
Firmicutes	Clostridia	Clostridiales	Ruminococcaceae	Anaerotruncus	uncultured_bacterium_g__Anaerotruncus	OTU245	0	0	1	0
Firmicutes	Clostridia	Clostridiales	Ruminococcaceae	Ruminococcaceae_UCG-014	unclassified_g__Ruminococcaceae_UCG-014	OTU429	0	0	0	0
Firmicutes	Erysipelotrichia	Erysipelotrichales	Erysipelotrichaceae	Faecalibaculum	uncultured_bacterium_g__Faecalibaculum	OTU497	0	0	0	0
Firmicutes	Clostridia	Clostridiales	Ruminococcaceae	unclassified_f__Ruminococcaceae	unclassified_f__Ruminococcaceae	OTU525	0	0	0	0
Firmicutes	Clostridia	Clostridiales	Ruminococcaceae	Anaerotruncus	unclassified_g__Anaerotruncus	OTU640	0	0	0	1
Actinobacteria	Actinobacteria	Coriobacteriales	Coriobacteriaceae	Coriobacteriaceae_UCG-002	uncultured_bacterium_g__Coriobacteriaceae_UCG-002	OTU365	0	0	0	0
Firmicutes	Clostridia	Clostridiales	Christensenellaceae	Christensenellaceae_R-7_group	unclassified_g__Christensenellaceae_R-7_group	OTU670	0	0	1	3
Firmicutes	Clostridia	Clostridiales	Ruminococcaceae	Ruminococcaceae_UCG-010	uncultured_bacterium_g__Ruminococcaceae_UCG-010	OTU776	0	0	0	0
Firmicutes	Clostridia	Clostridiales	Christensenellaceae	norank_f__Christensenellaceae	uncultured_bacterium_g__norank_f__Christensenellaceae	OTU139	0	0	0	0
Firmicutes	Clostridia	Clostridiales	Christensenellaceae	Christensenellaceae_R-7_group	uncultured_bacterium_g__Christensenellaceae_R-7_group	OTU207	0	0	0	0
Actinobacteria	Actinobacteria	Coriobacteriales	Coriobacteriaceae	unclassified_f__Coriobacteriaceae	unclassified_f__Coriobacteriaceae	OTU477	0	2	0	1
Firmicutes	Clostridia	Clostridiales	Ruminococcaceae	unclassified_f__Ruminococcaceae	unclassified_f__Ruminococcaceae	OTU176	0	0	0	0
Bacteroidetes	Bacteroidia	Bacteroidales	Bacteroidales_S24-7_g	norank_f__Bacteroidales_S24-7_gro	uncultured_bacterium_g__norank_f__Bacteroidales_S24-7_group	OTU262	0	0	0	0
Firmicutes	Clostridia	Clostridiales	Ruminococcaceae	Anaerotruncus	unclassified_g__Anaerotruncus	OTU444	0	0	0	0
Firmicutes	Clostridia	Clostridiales	Ruminococcaceae	Ruminococcaceae_UCG-013	uncultured_Clostridiales_bacterium_g__Ruminococcaceae_UCG-013	OTU522	0	0	0	0
Firmicutes	Clostridia	Clostridiales	Ruminococcaceae	Ruminococcaceae_UCG-010	uncultured_organism_g__Ruminococcaceae_UCG-010	OTU782	0	0	0	10
Saccharibacteria	Unknown_Class_p__Sacc	Unknown_Order_c_	Unknown_Family_o__	Candidatus_Saccharimonas	uncultured_bacterium_g__Candidatus_Saccharimonas	OTU261	0	0	0	0
Firmicutes	Clostridia	Clostridiales	Lachnospiraceae	unclassified_f__Lachnospiraceae	unclassified_f__Lachnospiraceae	OTU120	1	0	0	0
Chloroflexi	KD4-96	norank_c__KD4-96	norank_c__KD4-96	norank_c__KD4-96	unclassified_g__norank_c__KD4-96	OTU254	0	0	0	0
Firmicutes	Clostridia	Clostridiales	Clostridiales_vadinBB6	norank_f__Clostridiales_vadinBB60_	unclassified_g__norank_f__Clostridiales_vadinBB60_group	OTU209	0	0	0	3
Tenericutes	Mollicutes	Mollicutes_RF9	norank_o__Mollicutes	norank_o__Mollicutes_RF9	unclassified_g__norank_o__Mollicutes_RF9	OTU572	6	0	0	1
Firmicutes	Clostridia	Clostridiales	Ruminococcaceae	unclassified_f__Ruminococcaceae	unclassified_f__Ruminococcaceae	OTU195	0	0	0	0
Proteobacteria	Alphaproteobacteria	Sphingomonadales	Sphingomonadaceae	Sphingomonas	uncultured_Sphingomonadaceae_bacterium_g__Sphingomonas	OTU375	0	0	0	0
Tenericutes	Mollicutes	Mollicutes_RF9	norank_o__Mollicutes	norank_o__Mollicutes_RF9	unclassified_g__norank_o__Mollicutes_RF9	OTU727	0	0	1	0
Firmicutes	Erysipelotrichia	Erysipelotrichales	Erysipelotrichaceae	Turicibacter	uncultured_bacterium_g__Turicibacter	OTU40	0	0	0	0
Firmicutes	Clostridia	Clostridiales	Lachnospiraceae	Coprococcus_1	intestinal_bacterium_CG19-1	OTU45	0	1	0	0
Firmicutes	Clostridia	Clostridiales	Peptococcaceae	norank_f__Peptococcaceae	unclassified_g__norank_f__Peptococcaceae	OTU116	0	0	0	0
Firmicutes	Clostridia	Clostridiales	Peptococcaceae	norank_f__Peptococcaceae	unclassified_g__norank_f__Peptococcaceae	OTU199	0	1	0	0
Bacteroidetes	Bacteroidia	Bacteroidales	Bacteroidaceae	Bacteroides	Bacteroides_acidifaciens	OTU320	0	0	0	0
Firmicutes	Clostridia	Clostridiales	Ruminococcaceae	unclassified_f__Ruminococcaceae	unclassified_f__Ruminococcaceae	OTU552	0	0	0	0
Firmicutes	Clostridia	Clostridiales	Lachnospiraceae	Butyrivibrio	uncultured_organism_g__Butyrivibrio	OTU762	0	0	0	1
Tenericutes	Mollicutes	Mollicutes_RF9	norank_o__Mollicutes	norank_o__Mollicutes_RF9	uncultured_bacterium_g__norank_o__Mollicutes_RF9	OTU797	1	0	0	0
Firmicutes	Clostridia	Clostridiales	Lachnospiraceae	unclassified_f__Lachnospiraceae	unclassified_f__Lachnospiraceae	OTU852	1	0	0	0
Proteobacteria	Alphaproteobacteria	Rhizobiales	Bradyrhizobiaceae	Bradyrhizobium	Bradyrhizobium_elkanii	OTU185	0	0	0	0
Chloroflexi	Chloroflexia	Chloroflexales	Roseiflexaceae	Roseiflexus	uncultured_bacterium_g__Roseiflexus	OTU415	0	0	0	0
Proteobacteria	Alphaproteobacteria	Rhizobiales	Xanthobacteraceae	norank_f__Xanthobacteraceae	uncultured_bacterium_g__norank_f__Xanthobacteraceae	OTU427	0	0	0	0
Firmicutes	unclassified_p__Firmicut	unclassified_p__Fir	unclassified_p__Firmi	unclassified_p__Firmicutes	unclassified_p__Firmicutes	OTU454	0	0	0	0
Firmicutes	Clostridia	Clostridiales	Ruminococcaceae	Anaerotruncus	unclassified_g__Anaerotruncus	OTU621	1	0	1	0
Firmicutes	Clostridia	Clostridiales	Ruminococcaceae	Anaerotruncus	uncultured_Clostridiales_bacterium_g__Anaerotruncus	OTU736	0	1	0	1
Firmicutes	Clostridia	Clostridiales	Ruminococcaceae	Hydrogenoanaerobacterium	Selenomonadales_bacterium_Marseille-P2399	OTU5	0	0	0	0
Bacteroidetes	Bacteroidia	Bacteroidales	Bacteroidales_S24-7_g	norank_f__Bacteroidales_S24-7_gro	uncultured_bacterium_g__norank_f__Bacteroidales_S24-7_group	OTU300	0	0	0	0
Bacteroidetes	Bacteroidia	Bacteroidales	Prevotellaceae	Prevotellaceae_NK3B31_group	uncultured_bacterium_g__Prevotellaceae_NK3B31_group	OTU331	0	0	0	0
Proteobacteria	Betaproteobacteria	Burkholderiales	Burkholderiaceae	Ralstonia	unidentified_marine_bacterioplankton_g__Ralstonia	OTU593	0	1	0	2
Tenericutes	Mollicutes	Mollicutes_RF9	norank_o__Mollicutes	norank_o__Mollicutes_RF9	uncultured_Erysipelotrichia_bacterium_g__norank	OTU594	0	0	0	0
Firmicutes	Clostridia	Clostridiales	Ruminococcaceae	Anaerotruncus	unclassified_g__Anaerotruncus	OTU713	0	0	0	1
Firmicutes	Clostridia	Clostridiales	Clostridiales_vadinBB6	norank_f__Clostridiales_vadinBB60_	uncultured_bacterium_g__norank_f__Clostridiales_vadinBB60_group	OTU724	0	0	0	0
Firmicutes	Clostridia	Clostridiales	Christensenellaceae	unclassified_f__Christensenellaceae	unclassified_f__Christensenellaceae	OTU793	0	0	0	0
Firmicutes	Clostridia	Clostridiales	Lachnospiraceae	Tyzzerella	unclassified_g__Tyzzerella	OTU796	0	2	1	1
Actinobacteria	Actinobacteria	Bifidobacteriales	Bifidobacteriaceae	Bifidobacterium	Bifidobacterium_pseudocatenulatum_DSM_20438__JCM_1200__LMG_10505	OTU55	0	0	0	0
Firmicutes	Clostridia	Clostridiales	Clostridiales_vadinBB6	norank_f__Clostridiales_vadinBB60_	uncultured_bacterium_g__norank_f__Clostridiales_vadinBB60_group	OTU336	0	0	0	0
Bacteroidetes	Bacteroidia	Bacteroidales	Bacteroidaceae	Bacteroides	Bacteroides_sartorii	OTU345	0	0	0	0
Firmicutes	Clostridia	Clostridiales	Ruminococcaceae	Ruminiclostridium_5	unclassified_g__Ruminiclostridium_5	OTU818	0	0	1	0
Firmicutes	Clostridia	Clostridiales	Christensenellaceae	norank_f__Christensenellaceae	uncultured_bacterium_g__norank_f__Christensenellaceae	OTU144	0	0	0	0
Firmicutes	Clostridia	Clostridiales	Ruminococcaceae	[Eubacterium]_coprostanoligenes_g	unclassified_g__[Eubacterium]_coprostanoligenes_group	OTU212	0	0	0	0
Firmicutes	Clostridia	Clostridiales	Ruminococcaceae	Ruminococcaceae_UCG-013	unclassified_g__Ruminococcaceae_UCG-013	OTU342	0	0	0	0
Chloroflexi	Chloroflexia	Chloroflexales	Roseiflexaceae	Roseiflexus	uncultured_bacterium_g__Roseiflexus	OTU433	0	0	0	0
Firmicutes	Clostridia	Clostridiales	Ruminococcaceae	unclassified_f__Ruminococcaceae	unclassified_f__Ruminococcaceae	OTU646	0	0	0	1
Firmicutes	Bacilli	Lactobacillales	Lactobacillaceae	Lactobacillus	Lactobacillus_saerimneri	OTU719	0	0	0	0
Firmicutes	Clostridia	Clostridiales	Ruminococcaceae	Ruminococcus_1	uncultured_organism_g__Ruminococcus_1	OTU227	0	0	0	0
Deferribacteres	Deferribacteres	Deferribacterales	Deferribacteraceae	Mucispirillum	uncultured_bacterium_g__Mucispirillum	OTU272	0	0	0	0
Chloroflexi	Chloroflexia	Chloroflexales	Roseiflexaceae	Roseiflexus	uncultured_bacterium_g__Roseiflexus	OTU277	0	0	0	0
Actinobacteria	Actinobacteria	Solirubrobacterales	Solirubrobacteraceae	Solirubrobacter	uncultured_bacterium_g__Solirubrobacter	OTU414	0	0	0	0
Firmicutes	Clostridia	Clostridiales	Ruminococcaceae	Anaerotruncus	unclassified_g__Anaerotruncus	OTU546	0	0	0	0
Tenericutes	Mollicutes	Mollicutes_RF9	norank_o__Mollicutes	norank_o__Mollicutes_RF9	uncultured_bacterium_g__norank_o__Mollicutes_RF9	OTU571	2	0	0	1
Firmicutes	Clostridia	Clostridiales	Ruminococcaceae	Ruminiclostridium_5	uncultured_bacterium_g__Ruminiclostridium_5	OTU806	6	0	0	0
Firmicutes	Clostridia	Clostridiales	Ruminococcaceae	Ruminococcaceae_UCG-013	uncultured_bacterium_g__Ruminococcaceae_UCG-013	OTU108	0	0	0	0
Firmicutes	Clostridia	Clostridiales	Peptococcaceae	norank_f__Peptococcaceae	uncultured_bacterium_g__norank_f__Peptococcaceae	OTU112	0	0	0	0
Acidobacteria	Acidobacteria	Blastocatellales	Blastocatellaceae__Su	RB41	unclassified_g__RB41	OTU374	0	0	0	0
Actinobacteria	Actinobacteria	Propionibacteriales	Nocardioidaceae	Marmoricola	uncultured_bacterium_g__Marmoricola	OTU395	0	0	0	0
Firmicutes	Clostridia	Clostridiales	Ruminococcaceae	unclassified_f__Ruminococcaceae	unclassified_f__Ruminococcaceae	OTU650	0	0	0	0
Firmicutes	Clostridia	Clostridiales	Ruminococcaceae	Ruminiclostridium_9	unclassified_g__Ruminiclostridium_9	OTU92	0	0	0	0
Actinobacteria	Actinobacteria	Streptomycetales	Streptomycetaceae	Streptomyces	unclassified_g__Streptomyces	OTU358	0	0	0	0
Proteobacteria	Deltaproteobacteria	Desulfovibrionales	Desulfovibrionaceae	Desulfovibrio	unclassified_g__Desulfovibrio	OTU431	0	0	0	0
Firmicutes	Clostridia	Clostridiales	Lachnospiraceae	unclassified_f__Lachnospiraceae	unclassified_f__Lachnospiraceae	OTU668	0	1	0	1
Firmicutes	Clostridia	Clostridiales	Syntrophomonadacea	Syntrophomonas	unclassified_g__Syntrophomonas	OTU790	0	0	0	0
Bacteroidetes	Bacteroidia	Bacteroidales	Bacteroidales_S24-7_g	norank_f__Bacteroidales_S24-7_gro	uncultured_bacterium_g__norank_f__Bacteroidales_S24-7_group	OTU369	0	0	0	0
Firmicutes	Clostridia	Clostridiales	Peptostreptococcacea	Peptoclostridium	Peptoclostridium_difficile_g__Peptoclostridium	OTU459	0	0	0	0
Firmicutes	Erysipelotrichia	Erysipelotrichales	Erysipelotrichaceae	[Clostridium]_innocuum_group	uncultured_bacterium_g__[Clostridium]_innocuum_group	OTU503	0	0	0	0
Firmicutes	Clostridia	Clostridiales	Ruminococcaceae	Ruminococcaceae_UCG-009	uncultured_bacterium_g__Ruminococcaceae_UCG-009	OTU686	0	3	0	0
Firmicutes	Clostridia	Clostridiales	Ruminococcaceae	Anaerotruncus	unclassified_g__Anaerotruncus	OTU753	0	0	0	2
Firmicutes	Clostridia	Clostridiales	Ruminococcaceae	Ruminiclostridium	uncultured_organism_g__Ruminiclostridium	OTU829	0	0	0	0
Bacteroidetes	Bacteroidia	Bacteroidales	Bacteroidales_S24-7_g	norank_f__Bacteroidales_S24-7_gro	uncultured_bacterium_g__norank_f__Bacteroidales_S24-7_group	OTU304	0	0	0	0
Bacteroidetes	Bacteroidia	Bacteroidales	Bacteroidales_S24-7_g	norank_f__Bacteroidales_S24-7_gro	uncultured_bacterium_g__norank_f__Bacteroidales_S24-7_group	OTU350	0	0	0	0
Bacteroidetes	Bacteroidia	Bacteroidales	Bacteroidales_S24-7_g	norank_f__Bacteroidales_S24-7_gro	uncultured_bacterium_g__norank_f__Bacteroidales_S24-7_group	OTU380	0	0	0	0

Firmicutes	Clostridia	Clostridiales	Ruminococcaceae	Candidatus_Soleaferrea	Ruminococcaceae_bacterium_GD6	OTU456	0	0	0	0
Firmicutes	Clostridia	Clostridiales	Ruminococcaceae	unclassified_f__Ruminococcaceae	unclassified_f__Ruminococcaceae	OTU545	0	0	0	0
Firmicutes	Clostridia	Clostridiales	Ruminococcaceae	Ruminococcaceae_UCG-009	uncultured_bacterium_g__Ruminococcaceae_UCG-009	OTU595	0	0	0	0
Firmicutes	Clostridia	Clostridiales	Ruminococcaceae	unclassified_f__Ruminococcaceae	unclassified_f__Ruminococcaceae	OTU844	0	0	1	0
Firmicutes	Clostridia	Clostridiales	Christensenellaceae	norank_f__Christensenellaceae	uncultured_bacterium_g__norank_f__Christensenellaceae	OTU126	0	0	0	0
Firmicutes	Clostridia	Clostridiales	Clostridiales_vadinBB6	norank_f__Clostridiales_vadinBB60_	uncultured_bacterium_g__norank_f__Clostridiales_vadinBB60_group	OTU161	0	0	0	0
Bacteroidetes	Bacteroidia	Bacteroidales	Bacteroidales_S24-7_g	norank_f__Bacteroidales_S24-7_gro	unclassified_g__norank_f__Bacteroidales_S24-7_group	OTU267	0	0	0	0
Actinobacteria	Actinobacteria	Streptosporangiales	Thermomonosporacea	Actinomadura	Actinomadura_hallensis	OTU291	0	0	0	0
Firmicutes	Clostridia	Clostridiales	Ruminococcaceae	Ruminiclostridium_5	unclassified_g__Ruminiclostridium_5	OTU341	0	0	0	0
Actinobacteria	Actinobacteria	Corynebacteriales	Mycobacteriaceae	Mycobacterium	unclassified_g__Mycobacterium	OTU381	0	0	0	0
Firmicutes	Clostridia	Clostridiales	Ruminococcaceae	Ruminococcus_1	unclassified_g__Ruminococcus_1	OTU523	0	0	0	0
Bacteroidetes	Bacteroidia	Bacteroidales	Rikenellaceae	Alistipes	Alistipes_sp._CHKCI003	OTU15	0	0	0	0
Firmicutes	Clostridia	Clostridiales	Ruminococcaceae	Ruminococcaceae_UCG-010	unclassified_g__Ruminococcaceae_UCG-010	OTU222	0	0	0	0
Firmicutes	Clostridia	Clostridiales	Clostridiales_vadinBB6	norank_f__Clostridiales_vadinBB60_	uncultured_prokaryote_g__norank_f__Clostridiales_vadinBB60_group	OTU247	0	0	0	0
Bacteroidetes	Bacteroidia	Bacteroidales	Bacteroidales_S24-7_g	norank_f__Bacteroidales_S24-7_gro	uncultured_bacterium_g__norank_f__Bacteroidales_S24-7_group	OTU253	0	0	0	0
Verrucomicrobia	Spartobacteria	Chthoniobacterales	DA101_soil_group	norank_f__DA101_soil_group	uncultured_Prosthecobacter_sp._g__norank_f__DA101_soil_group	OTU279	0	0	0	0
Cyanobacteria	Cyanobacteria	Gastranaerophiales	norank_o__Gastranae	norank_o__Gastranaerophilales	unclassified_g__norank_o__Gastranaerophilales	OTU328	0	0	0	0
Proteobacteria	Epsilonproteobacteria	Campylobacterales	Helicobacteraceae	Helicobacter	Helicobacter_ganmani	OTU446	0	0	0	0
Firmicutes	Clostridia	Clostridiales	Ruminococcaceae	Ruminococcaceae_UCG-007	unclassified_g__Ruminococcaceae_UCG-007	OTU789	0	0	0	0
Proteobacteria	Alphaproteobacteria	Rhizobiales	Xanthobacteraceae	Variibacter	uncultured_forest_soil_bacterium_g__Variibacter	OTU840	0	0	0	0
Firmicutes	Clostridia	Clostridiales	Ruminococcaceae	Ruminiclostridium_5	uncultured_organism_g__Ruminiclostridium_5	OTU24	0	0	0	0
Gemmatimonade	Gemmatimonadetes	Gemmatimonadales	Gemmatimonadaceae	Gemmatimonas	uncultured_soil_bacterium_g__Gemmatimonas	OTU354	0	0	0	0
Actinobacteria	Actinobacteria	Propionibacteriales	Nocardioidaceae	Kribbella	uncultured_bacterium_g__Kribbella	OTU391	0	0	0	0
Actinobacteria	Actinobacteria	Pseudonocardiales	Pseudonocardiaceae	Pseudonocardia	uncultured_bacterium_g__Pseudonocardia	OTU392	0	0	0	0
Firmicutes	Clostridia	Clostridiales	Lachnospiraceae	unclassified_f__Lachnospiraceae	unclassified_f__Lachnospiraceae	OTU462	0	0	0	0
Firmicutes	Clostridia	Clostridiales	Ruminococcaceae	Anaerotruncus	unclassified_g__Anaerotruncus	OTU486	2	0	0	0
Actinobacteria	Actinobacteria	Coriobacteriales	Coriobacteriaceae	Gordonibacter	unclassified_g__Gordonibacter	OTU555	0	0	0	0
Firmicutes	Clostridia	Clostridiales	Ruminococcaceae	Anaerotruncus	unclassified_g__Anaerotruncus	OTU687	0	0	0	0
unclassified_k__r	unclassified_k__norank	unclassified_k__no	unclassified_k__noran	unclassified_k__norank	unclassified_k__norank	OTU706	0	2	0	0
Proteobacteria	Alphaproteobacteria	Rhizobiales	Xanthobacteraceae	Variibacter	uncultured_forest_soil_bacterium_g__Variibacter	OTU252	0	0	0	0
Actinobacteria	Actinobacteria	Micromonosporales	Micromonosporaceae	Longispora	uncultured_bacterium_g__Longispora	OTU357	0	0	0	0
Actinobacteria	Actinobacteria	Frankiales	Acidothermaceae	Acidothermus	Thermomonosporaceae_bacterium_YE4-D4-16-CH2	OTU403	0	0	0	0
Tenericutes	Mollicutes	Mollicutes_RF9	norank_o__Mollicutes	norank_o__Mollicutes_RF9	unclassified_g__norank_o__Mollicutes_RF9	OTU547	0	0	0	0
Bacteroidetes	Bacteroidia	Bacteroidales	Bacteroidaceae	Bacteroides	unclassified_g__Bacteroides	OTU554	0	0	0	0
Firmicutes	Clostridia	Clostridiales	Ruminococcaceae	unclassified_f__Ruminococcaceae	unclassified_f__Ruminococcaceae	OTU708	0	0	0	0
Firmicutes	Clostridia	Clostridiales	Christensenellaceae	unclassified_f__Christensenellaceae	unclassified_f__Christensenellaceae	OTU205	0	0	0	0
Firmicutes	Clostridia	Clostridiales	Ruminococcaceae	Ruminococcaceae_UCG-010	uncultured_bacterium_g__Ruminococcaceae_UCG-010	OTU240	0	0	0	0
Bacteroidetes	Bacteroidia	Bacteroidales	Rikenellaceae	Rikenella	uncultured_bacterium_g__Rikenella	OTU244	0	0	0	0
Bacteroidetes	Bacteroidia	Bacteroidales	Bacteroidales_S24-7_g	norank_f__Bacteroidales_S24-7_gro	uncultured_bacterium_g__norank_f__Bacteroidales_S24-7_group	OTU259	0	0	0	0
Bacteroidetes	Bacteroidia	Bacteroidales	Bacteroidales_S24-7_g	norank_f__Bacteroidales_S24-7_gro	unclassified_g__norank_f__Bacteroidales_S24-7_group	OTU280	0	0	0	0
Actinobacteria	Actinobacteria	Micromonosporales	Micromonosporaceae	unclassified_f__Micromonosporacea	unclassified_f__Micromonosporaceae	OTU286	0	0	0	0
Actinobacteria	Actinobacteria	Gaiellales	Gaiellaceae	Gaiella	uncultured_bacterium_g__Gaiella	OTU309	0	0	0	0
Bacteroidetes	Bacteroidia	Bacteroidales	Bacteroidales_S24-7_g	norank_f__Bacteroidales_S24-7_gro	uncultured_Bacteroidales_bacterium_g__norank_f__Bacteroidales_S24-7_group	OTU348	0	0	0	0
Actinobacteria	Actinobacteria	Propionibacteriales	Nocardioidaceae	Nocardioides	uncultured_bacterium_g__Nocardioides	OTU367	0	0	0	0
Actinobacteria	Actinobacteria	norank_c__Actinob	norank_c__Actinobact	norank_c__Actinobacteria	uncultured_bacterium_g__norank_c__Actinobacteria	OTU396	0	0	0	0
Firmicutes	Clostridia	Clostridiales	Ruminococcaceae	unclassified_f__Ruminococcaceae	unclassified_f__Ruminococcaceae	OTU617	0	0	0	0
Proteobacteria	Alphaproteobacteria	Rhizobiales	Methylobacteriaceae	Meganema	Meganema_perideroedes	OTU30	0	0	0	0
Firmicutes	Clostridia	Clostridiales	Ruminococcaceae	unclassified_f__Ruminococcaceae	unclassified_f__Ruminococcaceae	OTU239	0	0	0	0
Proteobacteria	Betaproteobacteria	Burkholderiales	Alcaligenaceae	Parasutterella	Sutterellaceae_bacterium_YL45	OTU281	0	0	0	0
Bacteroidetes	Bacteroidia	Bacteroidales	Bacteroidales_S24-7_g	norank_f__Bacteroidales_S24-7_gro	unclassified_g__norank_f__Bacteroidales_S24-7_group	OTU303	0	0	0	0
Bacteroidetes	Bacteroidia	Bacteroidales	Bacteroidales_S24-7_g	norank_f__Bacteroidales_S24-7_gro	uncultured_Bacteroidales_bacterium_g__norank_f__Bacteroidales_S24-7_group	OTU307	0	0	0	0
Chloroflexi	Anaerolineae	Anaerolineales	Anaerolineaceae	norank_f__Anaerolineaceae	wastewater_metagenome_g__norank_f__Anaerolineaceae	OTU352	0	0	0	0
Bacteroidetes	Bacteroidia	Bacteroidales	Bacteroidales_S24-7_g	norank_f__Bacteroidales_S24-7_gro	uncultured_bacterium_g__norank_f__Bacteroidales_S24-7_group	OTU389	0	0	0	0
Chloroflexi	Chloroflexia	Chloroflexales	Roseiflexaceae	Roseiflexus	uncultured_bacterium_g__Roseiflexus	OTU404	0	0	0	0
Firmicutes	Clostridia	Clostridiales	Lachnospiraceae	Lachnoclostridium	unclassified_g__Lachnoclostridium	OTU416	0	0	0	0
Chloroflexi	Chloroflexia	Chloroflexales	Roseiflexaceae	Roseiflexus	uncultured_bacterium_g__Roseiflexus	OTU425	0	0	0	0
Actinobacteria	Actinobacteria	Corynebacteriales	Nocardiaceae	Rhodococcus	Rhodococcus_erythropolis	OTU662	0	0	0	1
Proteobacteria	Gammaproteobacteria	Pseudomonadales	Moraxellaceae	Acinetobacter	unclassified_g__Acinetobacter	OTU685	0	0	0	0
Firmicutes	Clostridia	Clostridiales	Clostridiaceae_1	Clostridium_sensu_stricto_1	unclassified_g__Clostridium_sensu_stricto_1	OTU817	0	0	0	0
Firmicutes	Clostridia	Clostridiales	Christensenellaceae	Christensenellaceae_R-7_group	uncultured_Clostridiaceae_bacterium_g__Christensenellaceae_R-7_group	OTU47	0	0	0	0
Firmicutes	Clostridia	Clostridiales	Ruminococcaceae	Anaerotruncus	unclassified_g__Anaerotruncus	OTU96	0	0	0	0
Firmicutes	Clostridia	Clostridiales	Clostridiales_vadinBB6	norank_f__Clostridiales_vadinBB60_	uncultured_prokaryote_g__norank_f__Clostridiales_vadinBB60_group	OTU147	0	0	0	0
Firmicutes	Clostridia	Clostridiales	Ruminococcaceae	Candidatus_Soleaferrea	Ruminococcaceae_bacterium_Marseille-P2935	OTU183	0	1	0	0
Tenericutes	Mollicutes	Mollicutes_RF9	norank_o__Mollicutes	norank_o__Mollicutes_RF9	uncultured_bacterium_g__norank_o__Mollicutes_RF9	OTU208	0	0	0	0
Firmicutes	unclassified_p__Firmicut	unclassified_p__Fir	unclassified_p__Firmi	unclassified_p__Firmicutes	unclassified_p__Firmicutes	OTU246	0	0	0	0
Bacteroidetes	Bacteroidia	Bacteroidales	Bacteroidales_S24-7_g	norank_f__Bacteroidales_S24-7_gro	uncultured_bacterium_g__norank_f__Bacteroidales_S24-7_group	OTU266	0	0	0	0
Bacteroidetes	Bacteroidia	Bacteroidales	Rikenellaceae	Alistipes	uncultured_bacterium_g__Alistipes	OTU282	0	0	0	0
Actinobacteria	Actinobacteria	Solirubrobacterales	Solirubrobacteraceae	Solirubrobacter	uncultured_bacterium_g__Solirubrobacter	OTU288	0	0	0	0
Bacteroidetes	Bacteroidia	Bacteroidales	Bacteroidales_S24-7_g	norank_f__Bacteroidales_S24-7_gro	uncultured_bacterium_g__norank_f__Bacteroidales_S24-7_group	OTU289	0	0	0	0
Bacteroidetes	Bacteroidia	Bacteroidales	Bacteroidaceae	Bacteroides	unclassified_g__Bacteroides	OTU290	0	0	0	0
Chloroflexi	Chloroflexia	Chloroflexales	Roseiflexaceae	Roseiflexus	uncultured_bacterium_g__Roseiflexus	OTU318	0	0	0	0
Acidobacteria	Acidobacteria	norank_c__Acidoba	norank_c__Acidobacte	norank_c__Acidobacteria	uncultured_Acidobacteriales_bacterium_g__norank_c__Acidobacteria	OTU366	0	0	0	0
Proteobacteria	Epsilonproteobacteria	Campylobacterales	Campylobacteraceae	Arcobacter	unclassified_g__Arcobacter	OTU376	0	0	0	0
Gemmatimonade	Gemmatimonadetes	Gemmatimonadales	Gemmatimonadaceae	Gemmatimonas	unclassified_g__Gemmatimonas	OTU377	0	0	0	0
Proteobacteria	Alphaproteobacteria	Rhizobiales	Methylocystaceae	unclassified_f__Methylocystaceae	unclassified_f__Methylocystaceae	OTU379	0	0	0	0
Proteobacteria	Alphaproteobacteria	Rhodobacterales	Rhodobacteraceae	Pannonibacter	unclassified_g__Pannonibacter	OTU421	0	0	0	0
Proteobacteria	Epsilonproteobacteria	Campylobacterales	Helicobacteraceae	Helicobacter	Helicobacter_hepaticus	OTU422	0	0	0	0
Tenericutes	Mollicutes	Mollicutes_RF9	norank_o__Mollicutes	norank_o__Mollicutes_RF9	unclassified_g__norank_o__Mollicutes_RF9	OTU491	0	0	0	0
Firmicutes	Clostridia	Clostridiales	Ruminococcaceae	unclassified_f__Ruminococcaceae	unclassified_f__Ruminococcaceae	OTU696	0	0	0	0
Firmicutes	Clostridia	Clostridiales	Christensenellaceae	norank_f__Christensenellaceae	uncultured_bacterium_g__norank_f__Christensenellaceae	OTU819	0	0	0	0
Firmicutes	Clostridia	Clostridiales	Ruminococcaceae	unclassified_f__Ruminococcaceae	unclassified_f__Ruminococcaceae	OTU186	0	0	0	0
Firmicutes	Clostridia	Clostridiales	Ruminococcaceae	Ruminiclostridium	uncultured_bacterium_g__Ruminiclostridium	OTU269	0	0	0	0
Bacteroidetes	Bacteroidia	Bacteroidales	Porphyromonadaceae	Parabacteroides	uncultured_organism_g__Parabacteroides	OTU285	0	0	0	0
Bacteroidetes	Bacteroidia	Bacteroidales	Rikenellaceae	Alistipes	uncultured_bacterium_g__Alistipes	OTU299	0	0	0	0
Actinobacteria	Actinobacteria	Acidimicrobiales	norank_o__Acidimicro	norank_o__Acidimicrobiales	uncultured_Acidimicrobiales_bacterium_g__norank	OTU305	0	0	0	0
Chloroflexi	S085	norank_c__S085	norank_c__S085	norank_c__S085	uncultured_Chloroflexi_bacterium_g__norank_c__S085	OTU362	0	0	0	0
Proteobacteria	Alphaproteobacteria	Rhodospirillales	Acetobacteraceae	norank_f__Acetobacteraceae	uncultured_bacterium_g__norank_f__Acetobacteraceae	OTU371	0	0	0	0
Chloroflexi	TK10	norank_c__TK10	norank_c__TK10	norank_c__TK10	unclassified_g__norank_c__TK10	OTU382	0	0	0	0
Actinobacteria	Actinobacteria	Micrococcales	Brevibacteriaceae	Brevibacterium	unclassified_g__Brevibacterium	OTU399	0	0	0	0
Bacteroidetes	Bacteroidia	Bacteroidales	Bacteroidales_S24-7_g	norank_f__Bacteroidales_S24-7_gro	uncultured_organism_g__norank_f__Bacteroidales_S24-7_group	OTU401	0	0	0	0
Firmicutes	Erysipelotrichia	Erysipelotrichales	Erysipelotrichaceae	Erysipelatoclostridium	uncultured_bacterium_g__Erysipelatoclostridium	OTU407	0	0	0	0
Proteobacteria	Betaproteobacteria	Nitrosomonadales	Nitrosomonadaceae	norank_f__Nitrosomonadaceae	unclassified_g__norank_f__Nitrosomonadaceae	OTU420	0	0	0	0
Firmicutes	Clostridia	Clostridiales	Ruminococcaceae	Ruminococcaceae_UCG-014	uncultured_Firmicutes_bacterium_g__Ruminococcaceae_UCG-014	OTU798	0	0	0	0
Firmicutes	Clostridia	Clostridiales	Ruminococcaceae	Ruminococcaceae_UCG-010	unclassified_g__Ruminococcaceae_UCG-010	OTU800	0	0	0	0
Tenericutes	Mollicutes	Mycoplasmatales	Mycoplasmataceae	norank_f__Mycoplasmataceae	unclassified_g__norank_f__Mycoplasmataceae	OTU825	0	0	0	0
Firmicutes	Clostridia	Clostridiales	Christensenellaceae	Christensenellaceae_R-7_group	unclassified_g__Christensenellaceae_R-7_group	OTU833	0	0	0	0
Bacteroidetes	Bacteroidia	Bacteroidales	Bacteroidaceae	Bacteroides	Bacteroides_caccae	OTU46	0	0	0	0
Bacteroidetes	Flavobacteriia	Flavobacteriales	Flavobacteriaceae	Cloacibacterium	uncultured_bacterium_g__Cloacibacterium	OTU49	0	0	0	0
Firmicutes	Clostridia	Clostridiales	Ruminococcaceae	Ruminococcaceae_UCG-010	uncultured_bacterium_g__Ruminococcaceae_UCG-010	OTU97	0	0	0	0
Proteobacteria	Gammaproteobacteria	Pseudomonadales	Moraxellaceae	Acinetobacter	Acinetobacter_guillouiae	OTU138	0	0	0	0
Bacteroidetes	Bacteroidia	Bacteroidales	Bacteroidales_S24-7_g	norank_f__Bacteroidales_S24-7_gro	uncultured_bacterium_g__norank_f__Bacteroidales_S24-7_group	OTU211	0	0	0	0
Actinobacteria	Actinobacteria	Acidimicrobiales	Acidimicrobiaceae	CL500-29_marine_group	uncultured_bacterium_g__CL500-29_marine_group	OTU260	0	0	0	0
Acidobacteria	Acidobacteria	Acidobacteriales	Acidobacteriaceae__S	norank_f__Acidobacteriaceae__Subg	uncultured_bacterium_g__norank_f__Acidobacteriaceae__Subgroup_1_	OTU263	0	0	0	0
Proteobacteria	Alphaproteobacteria	Rhodospirillales	Rhodospirillaceae	norank_f__Rhodospirillaceae	unclassified_g__norank_f__Rhodospirillaceae	OTU273	0	0	0	0
Actinobacteria	Actinobacteria	Propionibacteriales	Nocardioidaceae	unclassified_f__Nocardioidaceae	unclassified_f__Nocardioidaceae	OTU298	0	0	0	0
Bacteroidetes	Bacteroidia	Bacteroidales	Bacteroidaceae	Bacteroides	Bacteroides_stercoris_ATCC_43183	OTU317	0	0	0	0
Chloroflexi	Chloroflexia	Chloroflexales	Roseiflexaceae	Roseiflexus	uncultured_bacterium_g__Roseiflexus	OTU372	0	0	0	0
Chloroflexi	Anaerolineae	Anaerolineales	Anaerolineaceae	norank_f__Anaerolineaceae	Chloroflexi_bacterium_OLB13	OTU383	0	0	0	0
Actinobacteria	Actinobacteria	Propionibacteriales	Nocardioidaceae	Nocardioides	unclassified_g__Nocardioides	OTU386	0	0	0	0
Chloroflexi	Thermomicrobia	JG30-KF-CM45	norank_o__JG30-KF-C	norank_o__JG30-KF-CM45	unclassified_g__norank_o__JG30-KF-CM45	OTU408	0	0	0	0
Chloroflexi	Gitt-GS-136	norank_c__Gitt-GS-	norank_c__Gitt-GS-13	norank_c__Gitt-GS-136	uncultured_bacterium_g__norank_c__Gitt-GS-136	OTU413	0	0	0	0

Actinobacteria	Actinobacteria	Acidimicrobiales	Iamiaceae	Iamia	uncultured_bacterium_g__Iamia	OTU417	0	0	0	0
Actinobacteria	Actinobacteria	Frankiales	Geodermatophilaceae	Blastococcus	uncultured_Blastococcus_sp._g__Blastococcus	OTU419	0	0	0	0
Actinobacteria	Actinobacteria	Propionibacteriales	Nocardioidaceae	Nocardioides	uncultured_organism_g__Nocardioides	OTU430	0	0	0	0
Gemmatimonade	Gemmatimonadetes	Gemmatimonadales	Gemmatimonadaceae	norank_f__Gemmatimonadaceae	uncultured_Gemmatimonadetes_bacterium_g__norank_f__Gemmatimonadaceae	OTU442	0	0	0	0
Firmicutes	Clostridia	Clostridiales	Ruminococcaceae	Anaerotruncus	uncultured_bacterium_g__Anaerotruncus	OTU592	0	0	0	0
Firmicutes	Clostridia	Clostridiales	Lachnospiraceae	Coprococcus_1	unclassified_g__Coprococcus_1	OTU734	0	1	0	0
Firmicutes	Clostridia	Clostridiales	Ruminococcaceae	Candidatus_Soleaferrea	Candidatus_Soleaferrea_massiliensis	OTU37	0	0	0	0
Cyanobacteria	Cyanobacteria	norank_c__Cyanoba	norank_c__Cyanobact	norank_c__Cyanobacteria	unclassified_g__norank_c__Cyanobacteria	OTU149	0	0	0	0
Firmicutes	Clostridia	Clostridiales	Ruminococcaceae	Ruminococcaceae_UCG-010	unclassified_g__Ruminococcaceae_UCG-010	OTU157	0	0	0	0
Bacteroidetes	Bacteroidia	Bacteroidales	Bacteroidales_S24-7_g	norank_f__Bacteroidales_S24-7_gro	unclassified_g__norank_f__Bacteroidales_S24-7_group	OTU190	0	0	0	0
Firmicutes	Clostridia	Clostridiales	Ruminococcaceae	Ruminococcaceae_UCG-010	uncultured_bacterium_g__Ruminococcaceae_UCG-010	OTU225	0	0	0	0
Proteobacteria	Deltaproteobacteria	Desulfovibrionales	Desulfovibrionaceae	Desulfovibrio	uncultured_bacterium_g__Desulfovibrio	OTU250	0	0	0	0
Bacteroidetes	Bacteroidia	Bacteroidales	Bacteroidales_S24-7_g	norank_f__Bacteroidales_S24-7_gro	uncultured_bacterium_g__norank_f__Bacteroidales_S24-7_group	OTU265	0	0	0	0
Proteobacteria	Betaproteobacteria	Burkholderiales	Comamonadaceae	unclassified_f__Comamonadaceae	unclassified_f__Comamonadaceae	OTU268	0	0	0	0
Firmicutes	Bacilli	Bacillales	Bacillaceae	unclassified_f__Bacillaceae	unclassified_f__Bacillaceae	OTU274	0	0	0	0
Firmicutes	Bacilli	Bacillales	Bacillaceae	Bacillus	Bacillus_anthracis_g__Bacillus	OTU275	0	0	0	0
Proteobacteria	Alphaproteobacteria	Rickettsiales	Mitochondria	norank_f__Mitochondria	Brassica_nigra__black_mustard_	OTU283	0	0	0	0
Firmicutes	Clostridia	Clostridiales	Ruminococcaceae	Anaerotruncus	Anaerotruncus_sp._G3_2012_	OTU296	0	0	0	0
Verrucomicrobia	Spartobacteria	Chthoniobacterales	DA101_soil_group	norank_f__DA101_soil_group	uncultured_Spartobacteria_bacterium_g__norank	OTU302	0	0	0	0
Actinobacteria	Actinobacteria	Acidimicrobiales	norank_o__Acidimicro	norank_o__Acidimicrobiales	uncultured_bacterium_g__norank_o__Acidimicrobiales	OTU314	0	0	0	0
Chloroflexi	Anaerolineae	Anaerolineales	Anaerolineaceae	norank_f__Anaerolineaceae	unclassified_g__norank_f__Anaerolineaceae	OTU316	0	0	0	0
Actinobacteria	Actinobacteria	Pseudonocardiales	Pseudonocardiaceae	Pseudonocardia	unclassified_g__Pseudonocardia	OTU351	0	0	0	0
Acidobacteria	Acidobacteria	Blastocatellales	Blastocatellaceae__Su	RB41	uncultured_Acidobacteria_bacterium_g__RB41	OTU359	0	0	0	0
Actinobacteria	Actinobacteria	Gaiellales	norank_o__Gaiellales	norank_o__Gaiellales	uncultured_bacterium_g__norank_o__Gaiellales	OTU370	0	0	0	0
Chloroflexi	Chloroflexia	Chloroflexales	Roseiflexaceae	Roseiflexus	uncultured_bacterium_g__Roseiflexus	OTU378	0	0	0	0
Chloroflexi	Chloroflexia	Chloroflexales	Roseiflexaceae	Roseiflexus	uncultured_bacterium_g__Roseiflexus	OTU384	0	0	0	0
Acidobacteria	Acidobacteria	Blastocatellales	Blastocatellaceae__Su	RB41	unclassified_g__RB41	OTU387	0	0	0	0
Acidobacteria	Acidobacteria	Solibacterales	Solibacteraceae__Sub	Bryobacter	uncultured_Acidobacterium_sp._g__Bryobacter	OTU388	0	0	0	0
Nitrospirae	Nitrospira	norank_c__Nitrospi	norank_c__Nitrospira	Nitrospira	unclassified_g__Nitrospira	OTU390	0	0	0	0
Actinobacteria	Actinobacteria	Acidimicrobiales	Acidimicrobiales_Ince	Candidatus_Microthrix	uncultured_bacterium_g__Candidatus_Microthrix	OTU393	0	0	0	0
Chloroflexi	Anaerolineae	Anaerolineales	Anaerolineaceae	norank_f__Anaerolineaceae	unclassified_g__norank_f__Anaerolineaceae	OTU397	0	0	0	0
Firmicutes	Clostridia	Clostridiales	Lachnospiraceae	norank_f__Lachnospiraceae	Lachnospiraceae_bacterium_A2	OTU398	0	0	0	0
Actinobacteria	Actinobacteria	Solirubrobacterales	Gsoil-1167	norank_f__Gsoil-1167	uncultured_bacterium_g__norank_f__Gsoil-1167	OTU406	0	0	0	0
Proteobacteria	Alphaproteobacteria	Rhizobiales	Bradyrhizobiaceae	Bosea	unclassified_g__Bosea	OTU426	0	0	0	0
Chloroflexi	Ktedonobacteria	JG30-KF-AS9	norank_o__JG30-KF-A	norank_o__JG30-KF-AS9	uncultured_bacterium_g__norank_o__JG30-KF-AS9	OTU440	0	0	0	0
Gemmatimonade	Gemmatimonadetes	Gemmatimonadales	Gemmatimonadaceae	Gemmatimonas	unclassified_g__Gemmatimonas	OTU441	0	0	0	0
Firmicutes	Clostridia	Clostridiales	Clostridiales_vadinBB6	norank_f__Clostridiales_vadinBB60_	unclassified_g__norank_f__Clostridiales_vadinBB60_group	OTU479	0	0	0	0
Firmicutes	Clostridia	Clostridiales	Lachnospiraceae	unclassified_f__Lachnospiraceae	unclassified_f__Lachnospiraceae	OTU514	0	0	0	0
Firmicutes	Clostridia	Clostridiales	Ruminococcaceae	unclassified_f__Ruminococcaceae	unclassified_f__Ruminococcaceae	OTU569	2	0	0	0
Firmicutes	Clostridia	Clostridiales	Ruminococcaceae	unclassified_f__Ruminococcaceae	unclassified_f__Ruminococcaceae	OTU760	0	0	0	0
Firmicutes	Clostridia	Clostridiales	Ruminococcaceae	Ruminococcaceae_UCG-005	unclassified_g__Ruminococcaceae_UCG-005	OTU814	0	0	0	0
Firmicutes	Clostridia	Clostridiales	Ruminococcaceae	Anaerotruncus	unclassified_g__Anaerotruncus	OTU44	0	0	0	0
Firmicutes	Clostridia	Clostridiales	Clostridiaceae_1	Clostridium_sensu_stricto_1	human_gut_metagenome_g__Clostridium_sensu_stricto_1	OTU77	0	0	0	0
Firmicutes	Clostridia	Clostridiales	Christensenellaceae	Christensenellaceae_R-7_group	unclassified_g__Christensenellaceae_R-7_group	OTU119	0	0	0	0
Bacteroidetes	Bacteroidia	Bacteroidales	Porphyromonadaceae	Odoribacter	uncultured_bacterium_g__Odoribacter	OTU210	0	0	0	0
Bacteroidetes	Bacteroidia	Bacteroidales	Bacteroidales_S24-7_g	norank_f__Bacteroidales_S24-7_gro	uncultured_bacterium_g__norank_f__Bacteroidales_S24-7_group	OTU217	0	0	0	0
Acidobacteria	Acidobacteria	norank_c__Acidoba	norank_c__Acidobacte	norank_c__Acidobacteria	uncultured_Acidobacteriales_bacterium_g__norank_c__Acidobacteria	OTU256	0	0	0	0
Gemmatimonade	Gemmatimonadetes	Gemmatimonadales	Gemmatimonadaceae	unclassified_f__Gemmatimonadacea	unclassified_f__Gemmatimonadaceae	OTU257	0	0	0	0
Acidobacteria	Acidobacteria	norank_c__Acidoba	norank_c__Acidobacte	norank_c__Acidobacteria	unclassified_g__norank_c__Acidobacteria	OTU264	0	0	0	0
Proteobacteria	Betaproteobacteria	Neisseriales	Neisseriaceae	Neisseria	unclassified_g__Neisseria	OTU271	0	0	0	0
Planctomycetes	OM190	norank_c__OM190	norank_c__OM190	norank_c__OM190	uncultured_bacterium_g__norank_c__OM190	OTU278	0	0	0	0
Actinobacteria	Actinobacteria	Coriobacteriales	Coriobacteriaceae	Parvibacter	uncultured_bacterium_g__Parvibacter	OTU292	0	0	0	0
Acidobacteria	Acidobacteria	norank_c__Acidoba	norank_c__Acidobacte	norank_c__Acidobacteria	uncultured_prokaryote_g__norank_c__Acidobacteria	OTU293	0	0	0	0
Actinobacteria	Actinobacteria	Gaiellales	norank_o__Gaiellales	norank_o__Gaiellales	uncultured_Rubrobacteria_bacterium_g__norank	OTU295	0	0	0	0
Proteobacteria	Gammaproteobacteria	BD7-8_marine_grou	norank_o__BD7-8_ma	norank_o__BD7-8_marine_group	unclassified_g__norank_o__BD7-8_marine_group	OTU297	0	0	0	0
Chloroflexi	Chloroflexia	Chloroflexales	Roseiflexaceae	Roseiflexus	uncultured_bacterium_g__Roseiflexus	OTU301	0	0	0	0
Actinobacteria	Actinobacteria	Propionibacteriales	Nocardioidaceae	unclassified_f__Nocardioidaceae	unclassified_f__Nocardioidaceae	OTU306	0	0	0	0
Saccharibacteria	norank_p__Saccharibact	norank_p__Sacchar	norank_p__Sacchariba	norank_p__Saccharibacteria	uncultured_bacterium_g__norank_p__Saccharibacteria	OTU311	0	0	0	0
Acidobacteria	Acidobacteria	norank_c__Acidoba	norank_c__Acidobacte	norank_c__Acidobacteria	uncultured_bacterium_66	OTU315	0	0	0	0
Firmicutes	Bacilli	Bacillales	Bacillaceae	unclassified_f__Bacillaceae	unclassified_f__Bacillaceae	OTU319	0	0	0	0
Firmicutes	Clostridia	Clostridiales	Clostridiales_vadinBB6	norank_f__Clostridiales_vadinBB60_	uncultured_bacterium_g__norank_f__Clostridiales_vadinBB60_group	OTU340	0	0	0	0
Acidobacteria	Acidobacteria	norank_c__Acidoba	norank_c__Acidobacte	norank_c__Acidobacteria	unclassified_g__norank_c__Acidobacteria	OTU349	0	0	0	0
Saccharibacteria	norank_p__Saccharibact	norank_p__Sacchar	norank_p__Saccharib	norank_p__Saccharibacteria	uncultured_bacterium_g__norank_p__Saccharibacteria	OTU353	0	0	0	0
Chloroflexi	Anaerolineae	Anaerolineales	Anaerolineaceae	norank_f__Anaerolineaceae	unclassified_g__norank_f__Anaerolineaceae	OTU356	0	0	0	0
Bacteroidetes	Bacteroidia	Bacteroidales	Porphyromonadaceae	Odoribacter	uncultured_bacterium_g__Odoribacter	OTU363	0	0	0	0
Bacteroidetes	Bacteroidia	Bacteroidales	Bacteroidales_S24-7_g	norank_f__Bacteroidales_S24-7_gro	uncultured_bacterium_g__norank_f__Bacteroidales_S24-7_group	OTU364	0	0	0	0
Bacteroidetes	Cytophagia	Cytophagales	Cytophagaceae	Persicitalea	uncultured_bacterium_g__Persicitalea	OTU373	0	0	0	0
Proteobacteria	Deltaproteobacteria	Myxococcales	BIrii41	norank_f__BIrii41	unclassified_g__norank_f__BIrii41	OTU385	0	0	0	0
Chloroflexi	unclassified_p__Chlorofl	unclassified_p__Chl	unclassified_p__Chlor	unclassified_p__Chloroflexi	unclassified_p__Chloroflexi	OTU394	0	0	0	0
Planctomycetes	Planctomycetacia	Planctomycetales	Planctomycetaceae	norank_f__Planctomycetaceae	unclassified_g__norank_f__Planctomycetaceae	OTU400	0	0	0	0
Nitrospirae	Nitrospira	norank_c__Nitrospi	norank_c__Nitrospira	Nitrospira	unclassified_g__Nitrospira	OTU402	0	0	0	0
Actinobacteria	Actinobacteria	Acidimicrobiales	OM1_clade	norank_f__OM1_clade	unclassified_g__norank_f__OM1_clade	OTU412	0	0	0	0
Firmicutes	Clostridia	Clostridiales	Lachnospiraceae	norank_f__Lachnospiraceae	uncultured_bacterium_g__norank_f__Lachnospiraceae	OTU418	0	0	0	0
Gemmatimonade	Gemmatimonadetes	Gemmatimonadales	Gemmatimonadaceae	Gemmatimonas	uncultured_bacterium_g__Gemmatimonas	OTU424	0	0	0	0
Chloroflexi	Thermomicrobia	JG30-KF-CM45	norank_o__JG30-KF-C	norank_o__JG30-KF-CM45	uncultured_Sphaerobacter_sp._g__norank	OTU428	0	0	0	0
Proteobacteria	Alphaproteobacteria	Rhodospirillales	Acetobacteraceae	norank_f__Acetobacteraceae	uncultured_bacterium_g__norank_f__Acetobacteraceae	OTU434	0	0	0	0
Actinobacteria	Actinobacteria	Coriobacteriales	Coriobacteriaceae	Coriobacteriaceae_UCG-002	uncultured_bacterium_g__Coriobacteriaceae_UCG-002	OTU436	0	0	0	0
Acidobacteria	Acidobacteria	unclassified_c__Aci	unclassified_c__Acido	unclassified_c__Acidobacteria	unclassified_c__Acidobacteria	OTU438	0	0	0	0
Chloroflexi	Ktedonobacteria	C0119	norank_o__C0119	norank_o__C0119	unclassified_g__norank_o__C0119	OTU439	0	0	0	0
unclassified_k__r	unclassified_k__norank	unclassified_k__no	unclassified_k__noran	unclassified_k__norank	unclassified_k__norank	OTU453	0	0	0	0
unclassified_k__r	unclassified_k__norank	unclassified_k__no	unclassified_k__noran	unclassified_k__norank	unclassified_k__norank	OTU620	0	0	2	0
unclassified_k__r	unclassified_k__norank	unclassified_k__no	unclassified_k__noran	unclassified_k__norank	unclassified_k__norank	OTU786	0	0	0	0

Phylum	Class	Order	Family	Genus	Species	OTU	Yellow1	Yellow2	Yellow3	Yellow4
Firmicutes	Clostridia	Clostridiales	Ruminococcaceae	Faecalibacterium	uncultured_bacterium_g__Faecalibacterium	OTU705	20737	7297	12004	12228
Bacteroidetes	Bacteroidia	Bacteroidales	Rikenellaceae	Alistipes	gut_metagenome_g__Alistipes	OTU287	1175	10999	7332	8122
Firmicutes	Clostridia	Clostridiales	Lachnospiraceae	[Ruminococcus]_torques_group	uncultured_Clostridiales_bacterium_g__[Ruminococcus]_torques_group	OTU361	3438	1690	3830	3540
Proteobacteria	Gammaproteobacteria	Enterobacteriales	Enterobacteriaceae	Escherichia-Shigella	Escherichia_coli_g__Escherichia-Shigella	OTU589	1108	2597	91	26
Firmicutes	Clostridia	Clostridiales	Ruminococcaceae	Faecalibacterium	bacterium_ic1379	OTU50	1	2260	525	4
Bacteroidetes	Bacteroidia	Bacteroidales	Rikenellaceae	Alistipes	unclassified_g__Alistipes	OTU501	588	2872	973	472
Firmicutes	Bacilli	Lactobacillales	Lactobacillaceae	Lactobacillus	unclassified_g__Lactobacillus	OTU445	2	1096	151	45
Firmicutes	Clostridia	Clostridiales	Ruminococcaceae	Subdoligranulum	uncultured_bacterium_g__Subdoligranulum	OTU816	702	56	43	182
Firmicutes	Clostridia	Clostridiales	Lachnospiraceae	unclassified_f__Lachnospiraceae	unclassified_f__Lachnospiraceae	OTU581	874	204	120	105
Firmicutes	Clostridia	Clostridiales	Lachnospiraceae	Blautia	unclassified_g__Blautia	OTU78	1574	269	98	106
Firmicutes	Clostridia	Clostridiales	Lachnospiraceae	Eisenbergiella	uncultured_bacterium_g__Eisenbergiella	OTU48	680	871	1160	587
Firmicutes	Clostridia	Clostridiales	Ruminococcaceae	Anaerotruncus	unclassified_g__Anaerotruncus	OTU213	811	516	1206	647
Firmicutes	Clostridia	Clostridiales	Lachnospiraceae	unclassified_f__Lachnospiraceae	unclassified_f__Lachnospiraceae	OTU710	723	132	237	954
Firmicutes	Bacilli	Lactobacillales	Lactobacillaceae	Lactobacillus	Lactobacillus_salivarius	OTU775	7	166	752	483
Firmicutes	Clostridia	Clostridiales	Lachnospiraceae	Shuttleworthia	uncultured_bacterium_g__Shuttleworthia	OTU683	363	331	1265	398
Firmicutes	Clostridia	Clostridiales	Ruminococcaceae	Butyricicoccus	uncultured_bacterium_g__Butyricicoccus	OTU616	58	157	891	510
Firmicutes	Clostridia	Clostridiales	Lachnospiraceae	[Ruminococcus]_torques_group	unclassified_g__[Ruminococcus]_torques_group	OTU196	43	20	8	24
Firmicutes	Clostridia	Clostridiales	Lachnospiraceae	Lachnoclostridium	unclassified_g__Lachnoclostridium	OTU527	344	192	655	670
Firmicutes	Clostridia	Clostridiales	Ruminococcaceae	Ruminiclostridium_9	unclassified_g__Ruminiclostridium_9	OTU174	280	1008	338	608
Firmicutes	Clostridia	Clostridiales	Ruminococcaceae	Subdoligranulum	unclassified_g__Subdoligranulum	OTU88	265	64	18	69
Firmicutes	Clostridia	Clostridiales	Ruminococcaceae	Ruminococcaceae_UCG-014	unclassified_g__Ruminococcaceae_UCG-014	OTU712	584	311	525	833
Proteobacteria	Betaproteobacteria	Burkholderiales	Alcaligenaceae	Parasutterella	uncultured_organism_g__Parasutterella	OTU692	1234	501	271	1360
Firmicutes	Clostridia	Clostridiales	Ruminococcaceae	Faecalibacterium	uncultured_bacterium_g__Faecalibacterium	OTU122	78	87	0	12
Firmicutes	Clostridia	Clostridiales	Clostridiales_vadinBB6	norank_f__Clostridiales_vadinBB60_	uncultured_bacterium_g__norank_f__Clostridiales_vadinBB60_group	OTU151	0	113	431	125
Firmicutes	Clostridia	Clostridiales	Ruminococcaceae	Faecalibacterium	uncultured_bacterium_g__Faecalibacterium	OTU70	2820	2061	58	0
Firmicutes	Clostridia	Clostridiales	Lachnospiraceae	unclassified_f__Lachnospiraceae	unclassified_f__Lachnospiraceae	OTU711	669	122	175	394
Firmicutes	Bacilli	Lactobacillales	Lactobacillaceae	Lactobacillus	Lactobacillus_vaginalis	OTU489	0	91	71	47
Firmicutes	Clostridia	Clostridiales	Lachnospiraceae	Sellimonas	unclassified_g__Sellimonas	OTU476	189	143	163	234
Firmicutes	Clostridia	Clostridiales	Lachnospiraceae	unclassified_f__Lachnospiraceae	unclassified_f__Lachnospiraceae	OTU179	226	499	622	658
Firmicutes	Clostridia	Clostridiales	Ruminococcaceae	norank_f__Ruminococcaceae	unclassified_g__norank_f__Ruminococcaceae	OTU191	1029	53	35	103
Firmicutes	Erysipelotrichia	Erysipelotrichales	Erysipelotrichaceae	Erysipelatoclostridium	bacterium_ic1391	OTU561	17	48	536	363
Firmicutes	Clostridia	Clostridiales	Ruminococcaceae	[Eubacterium]_coprostanoligenes_g	unclassified_g__[Eubacterium]_coprostanoligenes_group	OTU13	56	208	387	451
Firmicutes	Bacilli	Lactobacillales	Enterococcaceae	Enterococcus	Enterococcus_cecorum	OTU562	20	25	47	10
Firmicutes	Clostridia	Clostridiales	Lachnospiraceae	unclassified_f__Lachnospiraceae	unclassified_f__Lachnospiraceae	OTU484	427	91	389	390
Firmicutes	Clostridia	Clostridiales	Ruminococcaceae	unclassified_f__Ruminococcaceae	unclassified_f__Ruminococcaceae	OTU478	261	367	310	585
Firmicutes	Clostridia	Clostridiales	Lachnospiraceae	Lachnoclostridium	Clostridium_sp._BR31	OTU836	357	338	814	439
Firmicutes	Clostridia	Clostridiales	Ruminococcaceae	norank_f__Ruminococcaceae	Clostridiales_bacterium_24-4c	OTU743	6	311	665	124
Firmicutes	Bacilli	Bacillales	Bacillaceae	unclassified_f__Bacillaceae	unclassified_f__Bacillaceae	OTU851	299	206	191	275
Firmicutes	Clostridia	Clostridiales	Lachnospiraceae	[Ruminococcus]_torques_group	uncultured_Clostridiales_bacterium_g__[Ruminococcus]_torques_group	OTU774	172	23	83	393
Firmicutes	Clostridia	Clostridiales	Lachnospiraceae	[Eubacterium]_hallii_group	uncultured_bacterium_g__[Eubacterium]_hallii_group	OTU202	410	207	109	198
Firmicutes	Clostridia	Clostridiales	Lachnospiraceae	Anaerostipes	Anaerostipes_butyraticus	OTU189	344	110	74	90
Firmicutes	Clostridia	Clostridiales	Ruminococcaceae	Ruminococcaceae_UCG-014	uncultured_bacterium_g__Ruminococcaceae_UCG-014	OTU578	5	232	131	86
Firmicutes	Clostridia	Clostridiales	Lachnospiraceae	Sellimonas	bacterium_P1C11	OTU218	68	73	285	249
Firmicutes	Clostridia	Clostridiales	Christensenellaceae	Christensenellaceae_R-7_group	unclassified_g__Christensenellaceae_R-7_group	OTU709	0	137	861	595
Firmicutes	Clostridia	Clostridiales	Ruminococcaceae	Butyricicoccus	unclassified_g__Butyricicoccus	OTU33	14	20	113	145
Bacteroidetes	Bacteroidia	Bacteroidales	Bacteroidaceae	Bacteroides	Bacteroides_acidifaciens	OTU226	0	2	0	0
Firmicutes	Clostridia	Clostridiales	Ruminococcaceae	Ruminococcaceae_UCG-014	unclassified_g__Ruminococcaceae_UCG-014	OTU65	89	174	343	728
Firmicutes	Clostridia	Clostridiales	Lachnospiraceae	Lachnoclostridium	unclassified_g__Lachnoclostridium	OTU469	200	131	172	186
Firmicutes	Clostridia	Clostridiales	Ruminococcaceae	Ruminococcaceae_UCG-005	uncultured_organism_g__Ruminococcaceae_UCG-005	OTU690	0	754	79	218
Firmicutes	Erysipelotrichia	Erysipelotrichales	Erysipelotrichaceae	Erysipelatoclostridium	[Clostridium]_spiroforme	OTU625	24	350	664	361
Tenericutes	Mollicutes	Mollicutes_RF9	norank_o__Mollicutes	norank_o__Mollicutes_RF9	uncultured_bacterium_g__norank_o__Mollicutes_RF9	OTU588	0	9	3	34
Firmicutes	Clostridia	Clostridiales	Lachnospiraceae	Tyzzerella	uncultured_organism_g__Tyzzerella	OTU795	60	151	185	260
Firmicutes	Clostridia	Clostridiales	Ruminococcaceae	Ruminiclostridium	uncultured_bacterium_g__Ruminiclostridium	OTU368	15	264	197	349
Firmicutes	Clostridia	Clostridiales	Ruminococcaceae	Butyricicoccus	Butyricicoccus_pullicaecorum_1.2	OTU769	19	521	62	162
Firmicutes	Clostridia	Clostridiales	Ruminococcaceae	Ruminococcaceae_UCG-014	uncultured_bacterium_g__Ruminococcaceae_UCG-014	OTU346	26	46	182	351
Firmicutes	Clostridia	Clostridiales	Lachnospiraceae	norank_f__Lachnospiraceae	Clostridiales_bacterium_CHKCI001	OTU738	1	96	369	570
Proteobacteria	Gammaproteobacteria	Pseudomonadales	Pseudomonadaceae	Pseudomonas	Pseudomonas_brenneri	OTU580	19	203	809	296
Firmicutes	Clostridia	Clostridiales	Ruminococcaceae	norank_f__Ruminococcaceae	unclassified_g__norank_f__Ruminococcaceae	OTU460	137	611	48	173
Firmicutes	Clostridia	Clostridiales	Ruminococcaceae	Ruminococcaceae_UCG-014	unidentified_rumen_bacterium_JW32	OTU565	0	74	472	59
Firmicutes	Clostridia	Clostridiales	Ruminococcaceae	Ruminococcaceae_UCG-014	uncultured_rumen_bacterium_g__Ruminococcaceae_UCG-014	OTU813	164	9	10	4
Firmicutes	Clostridia	Clostridiales	Lachnospiraceae	Lachnospiraceae_NK4A136_group	unclassified_g__Lachnospiraceae_NK4A136_group	OTU193	15	24	227	12
Firmicutes	Clostridia	Clostridiales	Clostridiales_vadinBB6	norank_f__Clostridiales_vadinBB60_	uncultured_bacterium_g__norank_f__Clostridiales_vadinBB60_group	OTU573	141	156	329	571
Firmicutes	Clostridia	Clostridiales	Lachnospiraceae	Coprococcus_1	uncultured_organism_g__Coprococcus_1	OTU591	101	272	170	397
Firmicutes	Clostridia	Clostridiales	Lachnospiraceae	unclassified_f__Lachnospiraceae	unclassified_f__Lachnospiraceae	OTU515	80	38	30	22
Firmicutes	Clostridia	Clostridiales	Ruminococcaceae	Ruminococcaceae_UCG-014	uncultured_organism_g__Ruminococcaceae_UCG-014	OTU457	53	39	10	657
Firmicutes	Clostridia	Clostridiales	Lachnospiraceae	Blautia	Blautia_hydrogenotrophica	OTU718	211	28	110	91
Firmicutes	Clostridia	Clostridiales	Ruminococcaceae	norank_f__Ruminococcaceae	unclassified_g__norank_f__Ruminococcaceae	OTU735	138	1	101	50
Firmicutes	Clostridia	Clostridiales	Peptostreptococcacea	Romboutsia	uncultured_bacterium_g__Romboutsia	OTU79	1	64	4	3
Firmicutes	Clostridia	Clostridiales	Ruminococcaceae	Anaerotruncus	uncultured_bacterium_g__Anaerotruncus	OTU499	263	184	127	149
Firmicutes	Clostridia	Clostridiales	Ruminococcaceae	Ruminococcaceae_UCG-014	uncultured_bacterium_g__Ruminococcaceae_UCG-014	OTU7	2	51	14	55
Firmicutes	Clostridia	Clostridiales	Ruminococcaceae	Ruminococcaceae_UCG-014	unclassified_g__Ruminococcaceae_UCG-014	OTU25	112	180	307	345
Firmicutes	Clostridia	Clostridiales	Ruminococcaceae	[Eubacterium]_coprostanoligenes_g	gut_metagenome_g__[Eubacterium]_coprostanoligenes_group	OTU214	402	252	314	29
Firmicutes	Clostridia	Clostridiales	Ruminococcaceae	Ruminococcaceae_UCG-005	unclassified_g__Ruminococcaceae_UCG-005	OTU160	60	432	51	244
Firmicutes	Clostridia	Clostridiales	Ruminococcaceae	Flavonifractor	uncultured_bacterium_g__Flavonifractor	OTU716	167	77	215	236
Firmicutes	Clostridia	Clostridiales	Ruminococcaceae	Ruminococcaceae_UCG-014	uncultured_bacterium_g__Ruminococcaceae_UCG-014	OTU691	152	91	233	56
Firmicutes	Clostridia	Clostridiales	Ruminococcaceae	Ruminococcaceae_UCG-005	uncultured_prokaryote_g__Ruminococcaceae_UCG-005	OTU504	0	106	8	2
Firmicutes	Clostridia	Clostridiales	Ruminococcaceae	unclassified_f__Ruminococcaceae	unclassified_f__Ruminococcaceae	OTU496	22	220	83	158
Firmicutes	Clostridia	Clostridiales	Ruminococcaceae	Butyricicoccus	unclassified_g__Butyricicoccus	OTU480	185	19	16	251
Firmicutes	Clostridia	Clostridiales	Lachnospiraceae	unclassified_f__Lachnospiraceae	unclassified_f__Lachnospiraceae	OTU533	32	462	251	190
Firmicutes	Bacilli	Lactobacillales	Streptococcaceae	Streptococcus	Streptococcus_gallolyticus_subsp._macedonicus	OTU474	2	0	1	4
Firmicutes	Clostridia	Clostridiales	Ruminococcaceae	norank_f__Ruminococcaceae	Ruminococcaceae_bacterium_AM2	OTU242	0	204	31	0
Firmicutes	Clostridia	Clostridiales	Clostridiales_vadinBB6	norank_f__Clostridiales_vadinBB60_	uncultured_organism_g__norank_f__Clostridiales_vadinBB60_group	OTU150	0	0	0	0
Firmicutes	Clostridia	Clostridiales	Lachnospiraceae	Shuttleworthia	uncultured_bacterium_g__Shuttleworthia	OTU507	3	91	124	54
Firmicutes	Clostridia	Clostridiales	Lachnospiraceae	Blautia	uncultured_bacterium_g__Blautia	OTU450	1	272	119	50
Firmicutes	Clostridia	Clostridiales	Ruminococcaceae	Ruminococcaceae_UCG-014	uncultured_rumen_bacterium_g__Ruminococcaceae_UCG-014	OTU230	0	2034	61	4
Firmicutes	Clostridia	Clostridiales	Ruminococcaceae	Ruminococcaceae_UCG-004	unclassified_g__Ruminococcaceae_UCG-004	OTU355	71	115	45	175
Firmicutes	Clostridia	Clostridiales	Ruminococcaceae	norank_f__Ruminococcaceae	human_gut_metagenome_g__norank_f__Ruminococcaceae	OTU623	86	108	84	225
Firmicutes	Clostridia	Clostridiales	Lachnospiraceae	[Ruminococcus]_gauvreauii_group	bacterium_ic1296	OTU564	100	33	37	59
Firmicutes	Clostridia	Clostridiales	Ruminococcaceae	Anaerofilum	uncultured_bacterium_g__Anaerofilum	OTU123	13	181	117	327
Firmicutes	Clostridia	Clostridiales	Lachnospiraceae	unclassified_f__Lachnospiraceae	unclassified_f__Lachnospiraceae	OTU834	63	54	143	39
Firmicutes	Clostridia	Clostridiales	Clostridiales_vadinBB6	norank_f__Clostridiales_vadinBB60_	uncultured_bacterium_g__norank_f__Clostridiales_vadinBB60_group	OTU512	0	12	20	74
Firmicutes	Erysipelotrichia	Erysipelotrichales	Erysipelotrichaceae	Erysipelatoclostridium	Candidatus_Stoquefichus_sp._SN16	OTU707	1	6	45	0
Firmicutes	Clostridia	Clostridiales	Lachnospiraceae	[Eubacterium]_hallii_group	uncultured_bacterium_g__[Eubacterium]_hallii_group	OTU848	38	22	149	29
Firmicutes	Clostridia	Clostridiales	Ruminococcaceae	Ruminiclostridium_5	uncultured_bacterium_g__Ruminiclostridium_5	OTU856	150	32	28	114
Firmicutes	Clostridia	Clostridiales	Christensenellaceae	Christensenellaceae_R-7_group	uncultured_bacterium_g__Christensenellaceae_R-7_group	OTU171	0	60	69	128
Firmicutes	Clostridia	Clostridiales	Lachnospiraceae	Blautia	unclassified_g__Blautia	OTU767	0	0	22	74
Firmicutes	Clostridia	Clostridiales	Lachnospiraceae	unclassified_f__Lachnospiraceae	unclassified_f__Lachnospiraceae	OTU828	43	4	72	34
Firmicutes	Clostridia	Clostridiales	Ruminococcaceae	Ruminococcaceae_UCG-005	uncultured_Clostridiales_bacterium_g__Ruminococcaceae_UCG-005	OTU815	31	489	9	52
Firmicutes	Clostridia	Clostridiales	Ruminococcaceae	unclassified_f__Ruminococcaceae	unclassified_f__Ruminococcaceae	OTU845	109	27	25	19
Firmicutes	Clostridia	Clostridiales	Lachnospiraceae	unclassified_f__Lachnospiraceae	unclassified_f__Lachnospiraceae	OTU770	62	62	199	449
Firmicutes	Clostridia	Clostridiales	Ruminococcaceae	Anaerotruncus	uncultured_bacterium_g__Anaerotruncus	OTU838	137	29	59	124
Firmicutes	Clostridia	Clostridiales	Lachnospiraceae	unclassified_f__Lachnospiraceae	unclassified_f__Lachnospiraceae	OTU82	59	62	105	256
Firmicutes	Clostridia	Clostridiales	Ruminococcaceae	Anaerotruncus	Anaerotruncus_colihominis_DSM_17241	OTU125	16	16	7	40
Firmicutes	Clostridia	Clostridiales	Clostridiales_vadinBB6	norank_f__Clostridiales_vadinBB60_	uncultured_Clostridia_bacterium_g__norank_f__Clostridiales_vadinBB60_group	OTU697	0	22	37	4
Proteobacteria	Gammaproteobacteria	Pseudomonadales	Pseudomonadaceae	Pseudomonas	unclassified_g__Pseudomonas	OTU58	4	81	335	130
Firmicutes	Clostridia	Clostridiales	Christensenellaceae	Christensenellaceae_R-7_group	unclassified_g__Christensenellaceae_R-7_group	OTU830	1	60	77	37
Firmicutes	Clostridia	Clostridiales	Ruminococcaceae	Subdoligranulum	uncultured_Subdoligranulum_sp.	OTU519	357	1	19	1
Firmicutes	Clostridia	Clostridiales	Lachnospiraceae	Marvinbryantia	unclassified_g__Marvinbryantia	OTU177	7	1	10	6
Firmicutes	Clostridia	Clostridiales	Clostridiales_vadinBB6	norank_f__Clostridiales_vadinBB60_	unclassified_g__norank_f__Clostridiales_vadinBB60_group	OTU107	42	312	42	133
Firmicutes	Clostridia	Clostridiales	Lachnospiraceae	unclassified_f__Lachnospiraceae	unclassified_f__Lachnospiraceae	OTU216	165	64	6	69

Firmicutes	Clostridia	Clostridiales	Ruminococcaceae	Ruminococcaceae_NK4A214_group	uncultured_organism_g__Ruminococcaceae_NK4A214_group	OTU703	12	176	45	21
Firmicutes	Clostridia	Clostridiales	Lachnospiraceae	unclassified_f__Lachnospiraceae	unclassified_f__Lachnospiraceae	OTU540	21	37	14	74
Firmicutes	Clostridia	Clostridiales	Defluviitaleaceae	Defluviitaleaceae_UCG-011	uncultured_bacterium_g__Defluviitaleaceae_UCG-011	OTU725	267	31	15	283
Firmicutes	Clostridia	Clostridiales	Ruminococcaceae	Ruminococcaceae_UCG-010	uncultured_bacterium_g__Ruminococcaceae_UCG-010	OTU799	0	64	50	60
Firmicutes	Clostridia	Clostridiales	Ruminococcaceae	Ruminococcaceae_UCG-014	unclassified_g__Ruminococcaceae_UCG-014	OTU163	0	3	459	0
Firmicutes	Clostridia	Clostridiales	Clostridiales_vadinBB6	norank_f__Clostridiales_vadinBB60_	unclassified_g__norank_f__Clostridiales_vadinBB60_group	OTU57	50	264	142	136
Tenericutes	Mollicutes	Mollicutes_RF9	norank_o__Mollicutes	norank_o__Mollicutes_RF9	unclassified_g__norank_o__Mollicutes_RF9	OTU294	0	23	1023	62
Firmicutes	Clostridia	Clostridiales	Ruminococcaceae	Hydrogenoanaerobacterium	uncultured_bacterium_g__Hydrogenoanaerobacterium	OTU613	0	79	76	116
Firmicutes	Clostridia	Clostridiales	Ruminococcaceae	Ruminococcaceae_UCG-014	unclassified_g__Ruminococcaceae_UCG-014	OTU405	82	2	26	187
Firmicutes	Clostridia	Clostridiales	Ruminococcaceae	Ruminococcaceae_UCG-014	uncultured_bacterium_g__Ruminococcaceae_UCG-014	OTU570	3	4	102	193
Firmicutes	Clostridia	Clostridiales	Ruminococcaceae	norank_f__Ruminococcaceae	unclassified_g__norank_f__Ruminococcaceae	OTU313	91	85	88	112
Firmicutes	Bacilli	Lactobacillales	Lactobacillaceae	Lactobacillus	Lactobacillus_aviarius	OTU599	0	2	0	0
Firmicutes	Clostridia	Clostridiales	Lachnospiraceae	unclassified_f__Lachnospiraceae	unclassified_f__Lachnospiraceae	OTU717	115	72	55	210
Firmicutes	Clostridia	Clostridiales	Lachnospiraceae	Eisenbergiella	unclassified_g__Eisenbergiella	OTU443	0	193	84	49
Firmicutes	Clostridia	Clostridiales	Ruminococcaceae	norank_f__Ruminococcaceae	Ruminococcus_sp._16442	OTU113	47	11	13	132
Firmicutes	Clostridia	Clostridiales	Ruminococcaceae	Ruminococcus_1	unclassified_g__Ruminococcus_1	OTU140	0	1	81	148
Firmicutes	Clostridia	Clostridiales	Lachnospiraceae	Lachnospira	unclassified_g__Lachnospira	OTU170	90	50	29	21
Tenericutes	Mollicutes	Mollicutes_RF9	norank_o__Mollicutes	norank_o__Mollicutes_RF9	uncultured_bacterium_g__norank_o__Mollicutes_RF9	OTU823	53	78	37	74
Firmicutes	Clostridia	Clostridiales	Ruminococcaceae	unclassified_f__Ruminococcaceae	unclassified_f__Ruminococcaceae	OTU835	138	18	55	45
Firmicutes	Erysipelotrichia	Erysipelotrichales	Erysipelotrichaceae	norank_f__Erysipelotrichaceae	Clostridiales_bacterium_10-3b	OTU821	69	38	53	109
Firmicutes	Clostridia	Clostridiales	Peptococcaceae	norank_f__Peptococcaceae	uncultured_bacterium_g__norank_f__Peptococcaceae	OTU451	7	58	38	84
Firmicutes	Clostridia	Clostridiales	Lachnospiraceae	unclassified_f__Lachnospiraceae	unclassified_f__Lachnospiraceae	OTU135	0	0	71	0
Firmicutes	Clostridia	Clostridiales	Lachnospiraceae	unclassified_f__Lachnospiraceae	unclassified_f__Lachnospiraceae	OTU550	0	89	7	1
Firmicutes	Clostridia	Clostridiales	Ruminococcaceae	Anaerotruncus	uncultured_bacterium_g__Anaerotruncus	OTU556	2	3	19	231
Firmicutes	Erysipelotrichia	Erysipelotrichales	Erysipelotrichaceae	norank_f__Erysipelotrichaceae	Clostridiales_bacterium_60-7e	OTU583	36	4	5	21
Firmicutes	Clostridia	Clostridiales	Ruminococcaceae	Ruminococcaceae_UCG-014	unclassified_g__Ruminococcaceae_UCG-014	OTU200	24	34	49	28
Bacteroidetes	Bacteroidia	Bacteroidales	Rikenellaceae	Alistipes	gut_metagenome_g__Alistipes	OTU197	33	92	28	28
Tenericutes	Mollicutes	Mollicutes_RF9	norank_o__Mollicutes	norank_o__Mollicutes_RF9	gut_metagenome_g__norank_o__Mollicutes_RF9	OTU156	0	6	25	313
Firmicutes	Clostridia	Clostridiales	Ruminococcaceae	unclassified_f__Ruminococcaceae	unclassified_f__Ruminococcaceae	OTU66	107	0	2	14
Firmicutes	Erysipelotrichia	Erysipelotrichales	Erysipelotrichaceae	Faecalitalea	unclassified_g__Faecalitalea	OTU784	1	66	5	106
Firmicutes	Bacilli	Lactobacillales	Lactobacillaceae	Lactobacillus	Lactobacillus_johnsonii	OTU601	0	18	174	119
Bacteroidetes	Bacteroidia	Bacteroidales	Rikenellaceae	Alistipes	unclassified_g__Alistipes	OTU577	39	100	25	27
Firmicutes	Clostridia	Clostridiales	Ruminococcaceae	Subdoligranulum	unclassified_g__Subdoligranulum	OTU83	116	3	0	7
Proteobacteria	Deltaproteobacteria	Desulfovibrionales	Desulfovibrionaceae	Desulfovibrio	unclassified_g__Desulfovibrio	OTU636	11	71	18	107
Firmicutes	Clostridia	Clostridiales	Clostridiales_vadinBB6	norank_f__Clostridiales_vadinBB60_	unclassified_g__norank_f__Clostridiales_vadinBB60_group	OTU102	0	157	0	9
Firmicutes	Erysipelotrichia	Erysipelotrichales	Erysipelotrichaceae	Erysipelatoclostridium	unclassified_g__Erysipelatoclostridium	OTU8	126	85	65	28
Firmicutes	Clostridia	Clostridiales	Lachnospiraceae	[Ruminococcus]_torques_group	unclassified_g__[Ruminococcus]_torques_group	OTU531	27	6	8	21
Firmicutes	Clostridia	Clostridiales	Ruminococcaceae	Ruminococcaceae_UCG-014	unclassified_g__Ruminococcaceae_UCG-014	OTU614	0	1	0	0
Actinobacteria	Actinobacteria	Coriobacteriales	Coriobacteriaceae	Senegalimassilia	Coriobacteriaceae_bacterium_CHKCI002	OTU206	9	35	47	29
Firmicutes	Clostridia	Clostridiales	Ruminococcaceae	Ruminococcaceae_UCG-014	uncultured_bacterium_g__Ruminococcaceae_UCG-014	OTU842	0	95	27	25
Firmicutes	Clostridia	Clostridiales	Ruminococcaceae	Anaerotruncus	uncultured_organism_g__Anaerotruncus	OTU568	0	1	36	0
Firmicutes	Clostridia	Clostridiales	Ruminococcaceae	norank_f__Ruminococcaceae	unclassified_g__norank_f__Ruminococcaceae	OTU141	1	2	84	59
Firmicutes	Clostridia	Clostridiales	Clostridiales_vadinBB6	norank_f__Clostridiales_vadinBB60_	uncultured_bacterium_g__norank_f__Clostridiales_vadinBB60_group	OTU109	0	0	0	0
Cyanobacteria	Cyanobacteria	Gastranaerophilales	norank_o__Gastranae	norank_o__Gastranaerophilales	uncultured_rumen_bacterium_g__norank_o__Gastranaerophilales	OTU142	0	0	0	2
Firmicutes	Clostridia	Clostridiales	Lachnospiraceae	unclassified_f__Lachnospiraceae	unclassified_f__Lachnospiraceae	OTU699	13	22	142	15
Firmicutes	Clostridia	Clostridiales	Ruminococcaceae	Ruminococcaceae_UCG-010	gut_metagenome_g__Ruminococcaceae_UCG-010	OTU827	18	26	15	0
Firmicutes	Clostridia	Clostridiales	Lachnospiraceae	unclassified_f__Lachnospiraceae	unclassified_f__Lachnospiraceae	OTU612	93	23	58	60
Firmicutes	Clostridia	Clostridiales	Defluviitaleaceae	Defluviitaleaceae_UCG-011	uncultured_bacterium_g__Defluviitaleaceae_UCG-011	OTU567	41	38	56	33
Firmicutes	Clostridia	Clostridiales	Lachnospiraceae	Blautia	uncultured_bacterium_g__Blautia	OTU766	37	1	3	31
Firmicutes	Clostridia	Clostridiales	Christensenellaceae	Christensenellaceae_R-7_group	unclassified_g__Christensenellaceae_R-7_group	OTU148	0	20	24	31
Tenericutes	Mollicutes	Mollicutes_RF9	norank_o__Mollicutes	norank_o__Mollicutes_RF9	uncultured_bacterium_g__norank_o__Mollicutes_RF9	OTU559	0	75	66	146
Firmicutes	Clostridia	Clostridiales	Ruminococcaceae	Anaerotruncus	unclassified_g__Anaerotruncus	OTU657	72	10	15	11
Firmicutes	Clostridia	Clostridiales	Clostridiales_vadinBB6	norank_f__Clostridiales_vadinBB60_	uncultured_bacterium_g__norank_f__Clostridiales_vadinBB60_group	OTU22	0	114	9	106
Firmicutes	Clostridia	Clostridiales	Ruminococcaceae	Anaerotruncus	uncultured_Clostridiaceae_bacterium_g__Anaerotruncus	OTU618	50	0	7	20
Firmicutes	Clostridia	Clostridiales	Ruminococcaceae	Flavonifractor	uncultured_bacterium_g__Flavonifractor	OTU511	7	39	3	9
Firmicutes	Clostridia	Clostridiales	Clostridiales_vadinBB6	norank_f__Clostridiales_vadinBB60_	uncultured_bacterium_g__norank_f__Clostridiales_vadinBB60_group	OTU761	0	964	1	0
Firmicutes	Clostridia	Clostridiales	Clostridiales_vadinBB6	norank_f__Clostridiales_vadinBB60_	unclassified_g__norank_f__Clostridiales_vadinBB60_group	OTU231	0	391	45	199
Firmicutes	Clostridia	Clostridiales	Lachnospiraceae	unclassified_f__Lachnospiraceae	unclassified_f__Lachnospiraceae	OTU73	12	5	18	87
Firmicutes	Erysipelotrichia	Erysipelotrichales	Erysipelotrichaceae	Erysipelatoclostridium	Clostridiales_bacterium_CHKCI006	OTU700	40	25	39	38
Firmicutes	Clostridia	Clostridiales	Lachnospiraceae	unclassified_f__Lachnospiraceae	unclassified_f__Lachnospiraceae	OTU742	0	5	13	8
Firmicutes	Clostridia	Clostridiales	Ruminococcaceae	Anaerotruncus	uncultured_bacterium_g__Anaerotruncus	OTU146	88	3	49	37
Firmicutes	Clostridia	Clostridiales	Ruminococcaceae	Ruminococcaceae_UCG-010	uncultured_bacterium_g__Ruminococcaceae_UCG-010	OTU741	0	24	40	87
Firmicutes	Clostridia	Clostridiales	Ruminococcaceae	Subdoligranulum	unclassified_g__Subdoligranulum	OTU62	0	0	22	0
Firmicutes	Clostridia	Clostridiales	Lachnospiraceae	Blautia	uncultured_bacterium_g__Blautia	OTU466	21	11	19	32
Firmicutes	Clostridia	Clostridiales	Christensenellaceae	Christensenellaceae_R-7_group	uncultured_bacterium_g__Christensenellaceae_R-7_group	OTU746	4	44	23	34
Tenericutes	Mollicutes	Mollicutes_RF9	norank_o__Mollicutes	norank_o__Mollicutes_RF9	uncultured_bacterium_g__norank_o__Mollicutes_RF9	OTU192	0	58	0	17
Firmicutes	Clostridia	Clostridiales	Clostridiales_vadinBB6	norank_f__Clostridiales_vadinBB60_	uncultured_organism_g__norank_f__Clostridiales_vadinBB60_group	OTU103	0	57	0	0
Firmicutes	Clostridia	Clostridiales	Lachnospiraceae	unclassified_f__Lachnospiraceae	unclassified_f__Lachnospiraceae	OTU473	219	1	0	5
Firmicutes	Clostridia	Clostridiales	Ruminococcaceae	Ruminiclostridium_9	uncultured_bacterium_g__Ruminiclostridium_9	OTU110	1	21	5	0
Firmicutes	Bacilli	Lactobacillales	Lactobacillaceae	Lactobacillus	Lactobacillus_agilis	OTU607	0	0	1	0
Firmicutes	Clostridia	Clostridiales	Lachnospiraceae	Marvinbryantia	uncultured_organism_g__Marvinbryantia	OTU437	0	2	28	10
Firmicutes	Clostridia	Clostridiales	Ruminococcaceae	Ruminococcaceae_UCG-014	unclassified_g__Ruminococcaceae_UCG-014	OTU338	0	167	0	13
Firmicutes	Clostridia	Clostridiales	Clostridiales_vadinBB6	norank_f__Clostridiales_vadinBB60_	uncultured_rumen_bacterium_g__norank_f__Clostridiales_vadinBB60_group	OTU330	0	0	11	2
Tenericutes	Mollicutes	Mollicutes_RF9	norank_o__Mollicutes	norank_o__Mollicutes_RF9	uncultured_bacterium_g__norank_o__Mollicutes_RF9	OTU28	0	8	16	60
Firmicutes	Clostridia	Clostridiales	Lachnospiraceae	Coprococcus_1	unclassified_g__Coprococcus_1	OTU539	1	1	70	1
Firmicutes	Clostridia	Clostridiales	Lachnospiraceae	unclassified_f__Lachnospiraceae	unclassified_f__Lachnospiraceae	OTU642	12	5	19	103
Firmicutes	Clostridia	Clostridiales	Ruminococcaceae	Ruminococcaceae_UCG-014	unclassified_g__Ruminococcaceae_UCG-014	OTU590	3	26	83	32
Firmicutes	Clostridia	Clostridiales	Ruminococcaceae	norank_f__Ruminococcaceae	unclassified_g__norank_f__Ruminococcaceae	OTU701	12	6	4	117
Firmicutes	Clostridia	Clostridiales	Ruminococcaceae	Anaerotruncus	uncultured_bacterium_g__Anaerotruncus	OTU249	1	2	149	0
Firmicutes	Clostridia	Clostridiales	Ruminococcaceae	Anaerotruncus	uncultured_bacterium_g__Anaerotruncus	OTU248	4	140	52	31
Firmicutes	Clostridia	Clostridiales	Ruminococcaceae	Ruminiclostridium_5	unclassified_g__Ruminiclostridium_5	OTU826	1	0	12	25
Firmicutes	Clostridia	Clostridiales	Family_XIII	[Eubacterium]_nodatum_group	Eubacteriaceae_bacterium_Marseille-P2843	OTU585	19	26	45	41
Firmicutes	Clostridia	Clostridiales	Christensenellaceae	Christensenellaceae_R-7_group	uncultured_bacterium_g__Christensenellaceae_R-7_group	OTU167	23	17	8	14
Firmicutes	Clostridia	Clostridiales	Lachnospiraceae	norank_f__Lachnospiraceae	Clostridiales_bacterium_CHKCI001	OTU520	0	7	33	92
Firmicutes	Clostridia	Clostridiales	Ruminococcaceae	unclassified_f__Ruminococcaceae	unclassified_f__Ruminococcaceae	OTU238	28	40	104	95
Firmicutes	Clostridia	Clostridiales	Ruminococcaceae	unclassified_f__Ruminococcaceae	unclassified_f__Ruminococcaceae	OTU133	42	35	20	83
Tenericutes	Mollicutes	Mollicutes_RF9	norank_o__Mollicutes	norank_o__Mollicutes_RF9	uncultured_bacterium_g__norank_o__Mollicutes_RF9	OTU606	16	26	11	38
Firmicutes	Clostridia	Clostridiales	Ruminococcaceae	Ruminococcaceae_UCG-009	uncultured_bacterium_g__Ruminococcaceae_UCG-009	OTU779	16	8	4	33
Firmicutes	Clostridia	Clostridiales	Ruminococcaceae	Ruminococcaceae_NK4A214_group	uncultured_organism_g__Ruminococcaceae_NK4A214_group	OTU410	0	26	41	65
Verrucomicrobia	Verrucomicrobiae	Verrucomicrobiales	Verrucomicrobiaceae	Akkermansia	unclassified_g__Akkermansia	OTU344	0	0	13	10
Firmicutes	Clostridia	Clostridiales	Clostridiales_vadinBB6	norank_f__Clostridiales_vadinBB60_	uncultured_bacterium_g__norank_f__Clostridiales_vadinBB60_group	OTU1	0	0	0	0
Firmicutes	Clostridia	Clostridiales	Ruminococcaceae	Oscillospira	uncultured_bacterium_g__Oscillospira	OTU158	0	8	6	50
Firmicutes	Clostridia	Clostridiales	Lachnospiraceae	Lachnospiraceae_NK4A136_group	unclassified_g__Lachnospiraceae_NK4A136_group	OTU187	0	0	0	7
Firmicutes	Clostridia	Clostridiales	Lachnospiraceae	unclassified_f__Lachnospiraceae	unclassified_f__Lachnospiraceae	OTU463	0	5	6	51
Firmicutes	Clostridia	Clostridiales	Peptostreptococcacea	unclassified_f__Peptostreptococcac	unclassified_f__Peptostreptococcaceae	OTU526	13	1	0	8
Firmicutes	Bacilli	Lactobacillales	Lactobacillaceae	Lactobacillus	unclassified_g__Lactobacillus	OTU448	0	3	0	0
Firmicutes	Clostridia	Clostridiales	Ruminococcaceae	Ruminococcaceae_NK4A214_group	uncultured_rumen_bacterium_g__Ruminococcaceae_NK4A214_group	OTU181	0	1	0	0
Firmicutes	Clostridia	Clostridiales	Ruminococcaceae	Ruminococcaceae_UCG-014	unclassified_g__Ruminococcaceae_UCG-014	OTU822	1	97	109	87
Firmicutes	Clostridia	Clostridiales	Ruminococcaceae	Ruminococcaceae_UCG-013	unclassified_g__Ruminococcaceae_UCG-013	OTU458	0	0	1	0
Firmicutes	Clostridia	Clostridiales	Clostridiales_vadinBB6	norank_f__Clostridiales_vadinBB60_	unclassified_g__norank_f__Clostridiales_vadinBB60_group	OTU198	1	0	5	25
Firmicutes	Clostridia	Clostridiales	Clostridiales_vadinBB6	norank_f__Clostridiales_vadinBB60_	unclassified_g__norank_f__Clostridiales_vadinBB60_group	OTU241	0	0	2	0
Tenericutes	Mollicutes	Mollicutes_RF9	norank_o__Mollicutes	norank_o__Mollicutes_RF9	unclassified_g__norank_o__Mollicutes_RF9	OTU803	0	3	0	0
Firmicutes	Clostridia	Clostridiales	Lachnospiraceae	unclassified_f__Lachnospiraceae	unclassified_f__Lachnospiraceae	OTU81	63	4	10	6
Firmicutes	Clostridia	Clostridiales	Ruminococcaceae	Ruminococcaceae_UCG-014	unclassified_g__Ruminococcaceae_UCG-014	OTU12	0	2	2	31
Firmicutes	Clostridia	Clostridiales	Christensenellaceae	Christensenellaceae_R-7_group	unclassified_g__Christensenellaceae_R-7_group	OTU165	0	0	0	0
Firmicutes	Clostridia	Clostridiales	unclassified_o__Clostr	unclassified_o__Clostridiales	unclassified_o__Clostridiales	OTU682	0	15	133	3
Firmicutes	Clostridia	Clostridiales	Ruminococcaceae	unclassified_f__Ruminococcaceae	unclassified_f__Ruminococcaceae	OTU679	0	56	64	0
Firmicutes	Clostridia	Clostridiales	Ruminococcaceae	unclassified_f__Ruminococcaceae	unclassified_f__Ruminococcaceae	OTU455	97	2	1	3
Firmicutes	Clostridia	Clostridiales	Family_XIII	unclassified_f__Family_XIII	unclassified_f__Family_XIII	OTU488	22	39	60	28
Firmicutes	Clostridia	Clostridiales	Ruminococcaceae	Ruminococcaceae_UCG-013	uncultured_bacterium_g__Ruminococcaceae_UCG-013	OTU498	76	7	7	4
Firmicutes	Clostridia	Clostridiales	Ruminococcaceae	Anaerotruncus	unclassified_g__Anaerotruncus	OTU566	0	88	0	0

Firmicutes	Clostridia	Clostridiales	Clostridiales_vadinBB6	norank_f__Clostridiales_vadinBB60_	uncultured_Firmicutes_bacterium_g__norank_f__Clostridiales_vadinBB60_group	OTU638	0	55	1	14
Firmicutes	Clostridia	Clostridiales	Clostridiales_vadinBB6	norank_f__Clostridiales_vadinBB60_	uncultured_bacterium_g__norank_f__Clostridiales_vadinBB60_group	OTU3	0	3	0	10
Firmicutes	Clostridia	Clostridiales	Clostridiales_vadinBB6	norank_f__Clostridiales_vadinBB60_	uncultured_bacterium_g__norank_f__Clostridiales_vadinBB60_group	OTU494	0	31	46	11
Firmicutes	Clostridia	Clostridiales	Ruminococcaceae	Ruminiclostridium_5	uncultured_bacterium_g__Ruminiclostridium_5	OTU849	63	7	4	16
Firmicutes	Clostridia	Clostridiales	Ruminococcaceae	Ruminococcaceae_UCG-014	uncultured_bacterium_g__Ruminococcaceae_UCG-014	OTU114	0	29	55	12
Firmicutes	Clostridia	Clostridiales	Ruminococcaceae	Ruminococcaceae_UCG-010	uncultured_bacterium_g__Ruminococcaceae_UCG-010	OTU326	0	5	71	0
Firmicutes	Clostridia	Clostridiales	Ruminococcaceae	Ruminococcaceae_UCG-014	uncultured_bacterium_g__Ruminococcaceae_UCG-014	OTU749	28	40	12	21
Firmicutes	Clostridia	Clostridiales	Ruminococcaceae	[Eubacterium]_coprostanoligenes_g	unclassified_g__[Eubacterium]_coprostanoligenes_group	OTU233	0	38	0	0
Firmicutes	Clostridia	Clostridiales	Ruminococcaceae	Ruminococcaceae_UCG-014	unidentified_rumen_bacterium_JW32	OTU36	0	12	8	12
Firmicutes	Clostridia	Clostridiales	Ruminococcaceae	Ruminococcaceae_UCG-008	uncultured_bacterium_g__Ruminococcaceae_UCG-008	OTU39	11	0	0	1
Firmicutes	Clostridia	Clostridiales	Family_XIII	Family_XIII_UCG-001	uncultured_bacterium_g__Family_XIII_UCG-001	OTU329	38	9	19	14
Firmicutes	Clostridia	Clostridiales	Ruminococcaceae	Ruminiclostridium_5	uncultured_bacterium_g__Ruminiclostridium_5	OTU75	25	23	22	30
Firmicutes	Clostridia	Clostridiales	Peptococcaceae	norank_f__Peptococcaceae	uncultured_organism_g__norank_f__Peptococcaceae	OTU343	0	0	0	1
Firmicutes	Clostridia	Clostridiales	Ruminococcaceae	Anaerofilum	uncultured_bacterium_g__Anaerofilum	OTU660	5	9	15	14
Firmicutes	Clostridia	Clostridiales	Lachnospiraceae	Marvinbryantia	uncultured_bacterium_g__Marvinbryantia	OTU536	2	3	15	5
Firmicutes	Clostridia	Clostridiales	Ruminococcaceae	Anaerotruncus	unclassified_g__Anaerotruncus	OTU10	8	26	13	28
Firmicutes	Clostridia	Clostridiales	Clostridiales_vadinBB6	norank_f__Clostridiales_vadinBB60_	uncultured_bacterium_g__norank_f__Clostridiales_vadinBB60_group	OTU678	0	0	0	0
Firmicutes	Clostridia	Clostridiales	Ruminococcaceae	Ruminiclostridium_5	unclassified_g__Ruminiclostridium_5	OTU84	4	12	13	40
Firmicutes	Clostridia	Clostridiales	unclassified_o__Clostr	unclassified_o__Clostridiales	unclassified_o__Clostridiales	OTU137	48	5	12	26
Tenericutes	Mollicutes	Mollicutes_RF9	norank_o__Mollicutes	norank_o__Mollicutes_RF9	uncultured_bacterium_g__norank_o__Mollicutes_RF9	OTU604	0	20	68	13
Firmicutes	Erysipelotrichia	Erysipelotrichales	Erysipelotrichaceae	norank_f__Erysipelotrichaceae	uncultured_Erysipelotrichaceae_bacterium_g__norank	OTU772	0	0	0	0
Tenericutes	Mollicutes	Anaeroplasmatales	Anaeroplasmataceae	Anaeroplasma	uncultured_bacterium_g__Anaeroplasma	OTU801	0	64	12	65
Firmicutes	Clostridia	Clostridiales	Ruminococcaceae	Ruminococcaceae_UCG-014	unclassified_g__Ruminococcaceae_UCG-014	OTU220	0	0	0	75
Firmicutes	Clostridia	Clostridiales	Ruminococcaceae	Ruminiclostridium_9	unclassified_g__Ruminiclostridium_9	OTU587	26	28	1	28
Firmicutes	Clostridia	Clostridiales	Ruminococcaceae	norank_f__Ruminococcaceae	human_gut_metagenome_g__norank_f__Ruminococcaceae	OTU85	1	15	13	28
Cyanobacteria	Cyanobacteria	Gastranaerophilales	norank_o__Gastranae	norank_o__Gastranaerophilales	uncultured_bacterium_g__norank_o__Gastranaerophilales	OTU502	0	26	1	6
Firmicutes	Clostridia	Clostridiales	Clostridiales_vadinBB6	norank_f__Clostridiales_vadinBB60_	uncultured_bacterium_g__norank_f__Clostridiales_vadinBB60_group	OTU17	0	140	7	72
Firmicutes	Clostridia	Clostridiales	Lachnospiraceae	unclassified_f__Lachnospiraceae	unclassified_f__Lachnospiraceae	OTU513	8	12	8	8
Firmicutes	Clostridia	Clostridiales	Ruminococcaceae	unclassified_f__Ruminococcaceae	unclassified_f__Ruminococcaceae	OTU166	1	9	9	5
Tenericutes	Mollicutes	NB1-n	norank_o__NB1-n	norank_o__NB1-n	uncultured_organism_g__norank_o__NB1-n	OTU603	0	254	51	57
Firmicutes	Clostridia	Clostridiales	Ruminococcaceae	Ruminococcaceae_UCG-013	uncultured_bacterium_g__Ruminococcaceae_UCG-013	OTU720	16	11	91	107
Firmicutes	Clostridia	Clostridiales	Ruminococcaceae	Ruminococcaceae_UCG-014	unclassified_g__Ruminococcaceae_UCG-014	OTU347	1	8	8	26
Firmicutes	Clostridia	Clostridiales	Ruminococcaceae	Ruminiclostridium	uncultured_bacterium_g__Ruminiclostridium	OTU576	0	0	0	0
Cyanobacteria	Cyanobacteria	Gastranaerophilales	norank_o__Gastranae	norank_o__Gastranaerophilales	unclassified_g__norank_o__Gastranaerophilales	OTU563	0	15	0	0
Firmicutes	Clostridia	Clostridiales	Lachnospiraceae	unclassified_f__Lachnospiraceae	unclassified_f__Lachnospiraceae	OTU731	13	20	14	31
Firmicutes	Clostridia	Clostridiales	Lachnospiraceae	Roseburia	unclassified_g__Roseburia	OTU449	0	1	118	21
Firmicutes	Clostridia	Clostridiales	Clostridiales_vadinBB6	norank_f__Clostridiales_vadinBB60_	uncultured_bacterium_g__norank_f__Clostridiales_vadinBB60_group	OTU669	0	1	0	0
Tenericutes	Mollicutes	Mollicutes_RF9	norank_o__Mollicutes	norank_o__Mollicutes_RF9	unclassified_g__norank_o__Mollicutes_RF9	OTU104	0	0	0	0
Firmicutes	Clostridia	Clostridiales	Lachnospiraceae	Lachnospiraceae_UCG-010	uncultured_bacterium_g__Lachnospiraceae_UCG-010	OTU663	17	24	30	28
Firmicutes	Clostridia	Clostridiales	Ruminococcaceae	unclassified_f__Ruminococcaceae	unclassified_f__Ruminococcaceae	OTU847	0	2	0	1
Firmicutes	Clostridia	Clostridiales	Ruminococcaceae	Ruminococcaceae_UCG-013	uncultured_bacterium_g__Ruminococcaceae_UCG-013	OTU721	2	12	28	16
Tenericutes	Mollicutes	Mollicutes_RF9	norank_o__Mollicutes	norank_o__Mollicutes_RF9	uncultured_bacterium_g__norank_o__Mollicutes_RF9	OTU627	0	17	13	16
Firmicutes	Clostridia	Clostridiales	Ruminococcaceae	[Eubacterium]_coprostanoligenes_g	unclassified_g__[Eubacterium]_coprostanoligenes_group	OTU121	0	0	1	0
Firmicutes	Clostridia	Clostridiales	Clostridiales_vadinBB6	norank_f__Clostridiales_vadinBB60_	unclassified_g__norank_f__Clostridiales_vadinBB60_group	OTU180	2	47	24	30
Firmicutes	Clostridia	Clostridiales	Christensenellaceae	Christensenellaceae_R-7_group	unclassified_g__Christensenellaceae_R-7_group	OTU54	0	0	0	0
Firmicutes	Clostridia	Clostridiales	Ruminococcaceae	norank_f__Ruminococcaceae	unclassified_g__norank_f__Ruminococcaceae	OTU534	0	16	10	6
Firmicutes	Clostridia	Clostridiales	Ruminococcaceae	unclassified_f__Ruminococcaceae	unclassified_f__Ruminococcaceae	OTU493	22	6	43	26
Firmicutes	Clostridia	Clostridiales	Lachnospiraceae	norank_f__Lachnospiraceae	unclassified_g__norank_f__Lachnospiraceae	OTU560	30	7	9	1
Firmicutes	Clostridia	Clostridiales	Lachnospiraceae	[Eubacterium]_ventriosum_group	uncultured_organism_g__[Eubacterium]_ventriosum_group	OTU464	0	20	35	44
Tenericutes	Mollicutes	Mollicutes_RF9	norank_o__Mollicutes	norank_o__Mollicutes_RF9	unclassified_g__norank_o__Mollicutes_RF9	OTU234	0	25	9	6
Firmicutes	Clostridia	Clostridiales	Lachnospiraceae	unclassified_f__Lachnospiraceae	unclassified_f__Lachnospiraceae	OTU64	3	0	11	2
Tenericutes	Mollicutes	Mollicutes_RF9	norank_o__Mollicutes	norank_o__Mollicutes_RF9	uncultured_bacterium_g__norank_o__Mollicutes_RF9	OTU602	0	0	0	0
Firmicutes	Clostridia	Clostridiales	Ruminococcaceae	Anaerotruncus	uncultured_bacterium_g__Anaerotruncus	OTU6	0	28	3	91
Firmicutes	Bacilli	Lactobacillales	Lactobacillaceae	Lactobacillus	Lactobacillus_murinus_g__Lactobacillus	OTU843	0	0	333	379
Firmicutes	Clostridia	Clostridiales	Christensenellaceae	Christensenellaceae_R-7_group	uncultured_prokaryote_g__Christensenellaceae_R-7_group	OTU67	0	10	1	20
Firmicutes	Clostridia	Clostridiales	Clostridiales_vadinBB6	norank_f__Clostridiales_vadinBB60_	uncultured_bacterium_g__norank_f__Clostridiales_vadinBB60_group	OTU748	0	30	0	63
Firmicutes	Clostridia	Clostridiales	Lachnospiraceae	Coprococcus_1	unclassified_g__Coprococcus_1	OTU739	16	0	11	17
Tenericutes	Mollicutes	Mollicutes_RF9	norank_o__Mollicutes	norank_o__Mollicutes_RF9	unclassified_g__norank_o__Mollicutes_RF9	OTU229	0	34	7	7
Firmicutes	Clostridia	Clostridiales	Clostridiales_vadinBB6	norank_f__Clostridiales_vadinBB60_	uncultured_bacterium_g__norank_f__Clostridiales_vadinBB60_group	OTU23	0	0	0	0
Firmicutes	Clostridia	Clostridiales	Clostridiales_vadinBB6	norank_f__Clostridiales_vadinBB60_	uncultured_bacterium_g__norank_f__Clostridiales_vadinBB60_group	OTU131	0	0	0	0
Firmicutes	Clostridia	Clostridiales	Ruminococcaceae	Ruminococcaceae_UCG-014	unclassified_g__Ruminococcaceae_UCG-014	OTU837	0	0	1	0
Firmicutes	Clostridia	Clostridiales	unclassified_o__Clostr	unclassified_o__Clostridiales	unclassified_o__Clostridiales	OTU91	0	10	21	4
Firmicutes	Clostridia	Clostridiales	Lachnospiraceae	Roseburia	unclassified_g__Roseburia	OTU43	48	0	1	16
Firmicutes	Clostridia	Clostridiales	Lachnospiraceae	unclassified_f__Lachnospiraceae	unclassified_f__Lachnospiraceae	OTU11	35	3	4	34
Proteobacteria	Betaproteobacteria	Burkholderiales	Oxalobacteraceae	Oxalobacter	unclassified_g__Oxalobacter	OTU557	0	0	0	1
Firmicutes	Clostridia	Clostridiales	Ruminococcaceae	Faecalibacterium	unclassified_g__Faecalibacterium	OTU219	84	0	1	4
Firmicutes	Clostridia	Clostridiales	Ruminococcaceae	Ruminiclostridium_5	uncultured_bacterium_g__Ruminiclostridium_5	OTU59	32	8	7	48
Firmicutes	Clostridia	Clostridiales	Lachnospiraceae	Lachnospiraceae_UCG-010	uncultured_bacterium_g__Lachnospiraceae_UCG-010	OTU755	0	9	8	7
Bacteroidetes	Bacteroidia	Bacteroidales	Rikenellaceae	Alistipes	uncultured_bacterium_g__Alistipes	OTU765	0	0	1	0
Firmicutes	Erysipelotrichia	Erysipelotrichales	Erysipelotrichaceae	[Clostridium]_innocuum_group	uncultured_bacterium_g__[Clostridium]_innocuum_group	OTU794	0	5	1	0
Firmicutes	Clostridia	Clostridiales	unclassified_o__Clostr	unclassified_o__Clostridiales	unclassified_o__Clostridiales	OTU673	0	46	0	0
Firmicutes	Clostridia	Clostridiales	Clostridiales_vadinBB6	norank_f__Clostridiales_vadinBB60_	gut_metagenome_g__norank_f__Clostridiales_vadinBB60_group	OTU548	2	4	5	26
Tenericutes	Mollicutes	Mollicutes_RF9	norank_o__Mollicutes	norank_o__Mollicutes_RF9	uncultured_bacterium_g__norank_o__Mollicutes_RF9	OTU544	0	7	0	5
Cyanobacteria	Cyanobacteria	Gastranaerophilales	norank_o__Gastranae	norank_o__Gastranaerophilales	unclassified_g__norank_o__Gastranaerophilales	OTU155	0	82	24	75
Firmicutes	Clostridia	Clostridiales	Clostridiales_vadinBB6	norank_f__Clostridiales_vadinBB60_	uncultured_bacterium_g__norank_f__Clostridiales_vadinBB60_group	OTU481	0	0	0	0
Firmicutes	Clostridia	Clostridiales	Lachnospiraceae	unclassified_f__Lachnospiraceae	unclassified_f__Lachnospiraceae	OTU467	0	10	27	15
Firmicutes	Clostridia	Clostridiales	Ruminococcaceae	norank_f__Ruminococcaceae	unclassified_g__norank_f__Ruminococcaceae	OTU622	1	50	2	3
Firmicutes	Clostridia	Clostridiales	Lachnospiraceae	unclassified_f__Lachnospiraceae	unclassified_f__Lachnospiraceae	OTU201	20	2	21	3
Firmicutes	Clostridia	Clostridiales	Lachnospiraceae	norank_f__Lachnospiraceae	uncultured_organism_g__norank_f__Lachnospiraceae	OTU654	0	2	0	10
Firmicutes	Clostridia	Clostridiales	Ruminococcaceae	Ruminococcaceae_UCG-014	unclassified_g__Ruminococcaceae_UCG-014	OTU575	0	1	0	331
Tenericutes	Mollicutes	Mollicutes_RF9	norank_o__Mollicutes	norank_o__Mollicutes_RF9	unclassified_g__norank_o__Mollicutes_RF9	OTU600	0	15	65	16
Firmicutes	Clostridia	Clostridiales	Ruminococcaceae	unclassified_f__Ruminococcaceae	unclassified_f__Ruminococcaceae	OTU728	36	7	10	13
Firmicutes	Clostridia	Clostridiales	Ruminococcaceae	Anaerotruncus	unclassified_g__Anaerotruncus	OTU159	0	0	0	0
Tenericutes	Mollicutes	Mollicutes_RF9	norank_o__Mollicutes	norank_o__Mollicutes_RF9	uncultured_bacterium_g__norank_o__Mollicutes_RF9	OTU608	0	0	16	9
Firmicutes	Clostridia	Clostridiales	Ruminococcaceae	Ruminococcaceae_UCG-010	uncultured_bacterium_g__Ruminococcaceae_UCG-010	OTU674	5	2	3	1
Firmicutes	Clostridia	Clostridiales	Lachnospiraceae	Lachnospiraceae_FCS020_group	uncultured_bacterium_g__Lachnospiraceae_FCS020_group	OTU802	22	13	3	10
Firmicutes	Clostridia	Clostridiales	Ruminococcaceae	Caproiciproducens	uncultured_bacterium_g__Caproiciproducens	OTU68	0	1	24	12
Firmicutes	Clostridia	Clostridiales	Peptococcaceae	norank_f__Peptococcaceae	unclassified_g__norank_f__Peptococcaceae	OTU586	0	19	3	12
Firmicutes	Clostridia	Clostridiales	Ruminococcaceae	Anaerotruncus	uncultured_bacterium_g__Anaerotruncus	OTU175	11	17	8	19
Firmicutes	Clostridia	Clostridiales	Lachnospiraceae	unclassified_f__Lachnospiraceae	unclassified_f__Lachnospiraceae	OTU854	11	11	5	25
Firmicutes	Clostridia	Clostridiales	Christensenellaceae	Christensenellaceae_R-7_group	uncultured_bacterium_g__Christensenellaceae_R-7_group	OTU164	0	2	5	0
Firmicutes	Clostridia	Clostridiales	Ruminococcaceae	Ruminococcus_2	uncultured_bacterium_g__Ruminococcus_2	OTU447	0	1	0	0
Actinobacteria	Actinobacteria	Coriobacteriales	Coriobacteriaceae	unclassified_f__Coriobacteriaceae	unclassified_f__Coriobacteriaceae	OTU490	4	19	2	7
Proteobacteria	Alphaproteobacteria	Rhizobiales	Brucellaceae	Brucella	Brucella_melitensis_g__Brucella	OTU812	0	0	105	396
Firmicutes	Clostridia	Clostridiales	Ruminococcaceae	Ruminiclostridium_1	uncultured_bacterium_g__Ruminiclostridium_1	OTU485	3	14	1	59
Tenericutes	Mollicutes	Mollicutes_RF9	norank_o__Mollicutes	norank_o__Mollicutes_RF9	unclassified_g__norank_o__Mollicutes_RF9	OTU611	0	15	41	13
Firmicutes	Clostridia	Clostridiales	Ruminococcaceae	Ruminococcaceae_UCG-014	unclassified_g__Ruminococcaceae_UCG-014	OTU100	0	0	0	0
Firmicutes	Clostridia	Clostridiales	Ruminococcaceae	norank_f__Ruminococcaceae	unclassified_g__norank_f__Ruminococcaceae	OTU98	0	0	0	0
Firmicutes	Bacilli	Lactobacillales	Enterococcaceae	Enterococcus	Enterococcus_faecalis_g__Enterococcus	OTU763	0	3	64	70
Firmicutes	Bacilli	Lactobacillales	Lactobacillaceae	Lactobacillus	Lactobacillus_coleohominis	OTU628	0	3	1	0
Firmicutes	Erysipelotrichia	Erysipelotrichales	Erysipelotrichaceae	unclassified_f__Erysipelotrichaceae	unclassified_f__Erysipelotrichaceae	OTU839	0	16	19	31
Firmicutes	Clostridia	Clostridiales	Christensenellaceae	Christensenellaceae_R-7_group	bacterium_YE57	OTU754	4	4	16	29
Firmicutes	Clostridia	Clostridiales	Ruminococcaceae	Ruminiclostridium_9	unclassified_g__Ruminiclostridium_9	OTU500	0	1	0	1
Firmicutes	Clostridia	Clostridiales	Ruminococcaceae	Faecalibacterium	unclassified_g__Faecalibacterium	OTU846	3	6	11	9
Tenericutes	Mollicutes	Mollicutes_RF9	norank_o__Mollicutes	norank_o__Mollicutes_RF9	unclassified_g__norank_o__Mollicutes_RF9	OTU610	0	0	0	0
Firmicutes	Clostridia	Clostridiales	Ruminococcaceae	norank_f__Ruminococcaceae	unclassified_g__norank_f__Ruminococcaceae	OTU132	19	0	0	0
Firmicutes	Clostridia	Clostridiales	Ruminococcaceae	Ruminococcus_1	uncultured_bacterium_g__Ruminococcus_1	OTU639	0	1	59	0
Firmicutes	Clostridia	Clostridiales	Peptococcaceae	unclassified_f__Peptococcaceae	unclassified_f__Peptococcaceae	OTU483	0	4	19	29
Tenericutes	Mollicutes	Mollicutes_RF9	norank_o__Mollicutes	norank_o__Mollicutes_RF9	uncultured_bacterium_g__norank_o__Mollicutes_RF9	OTU631	1	1	7	5
Firmicutes	Clostridia	Clostridiales	Ruminococcaceae	Caproiciproducens	unclassified_g__Caproiciproducens	OTU824	0	0	0	0
Firmicutes	Clostridia	Clostridiales	Clostridiales_vadinBB6	norank_f__Clostridiales_vadinBB60_	uncultured_bacterium_g__norank_f__Clostridiales_vadinBB60_group	OTU757	0	1	0	0

Tenericutes	Mollicutes	Mollicutes_RF9	norank_o__Mollicutes	norank_o__Mollicutes_RF9	uncultured_bacterium_g__norank_o__Mollicutes_RF9	OTU792	0	1	26	15
Firmicutes	Clostridia	Clostridiales	Lachnospiraceae	Coprococcus_1	uncultured_bacterium_g__Coprococcus_1	OTU629	0	0	3	0
Firmicutes	Clostridia	Clostridiales	Peptococcaceae	Peptococcus	uncultured_organism_g__Peptococcus	OTU665	0	10	10	0
Firmicutes	Clostridia	Clostridiales	Ruminococcaceae	Ruminococcaceae_UCG-014	unclassified_g__Ruminococcaceae_UCG-014	OTU532	0	0	0	0
Firmicutes	Clostridia	Clostridiales	Ruminococcaceae	norank_f__Ruminococcaceae	unclassified_g__norank_f__Ruminococcaceae	OTU671	0	13	11	3
Firmicutes	Clostridia	Clostridiales	Ruminococcaceae	Ruminococcus_1	uncultured_organism_g__Ruminococcus_1	OTU56	10	3	5	3
Firmicutes	Clostridia	Clostridiales	Clostridiales_vadinBB6	norank_f__Clostridiales_vadinBB60_	uncultured_bacterium_g__norank_f__Clostridiales_vadinBB60_group	OTU714	18	47	28	7
Firmicutes	Clostridia	Clostridiales	Lachnospiraceae	unclassified_f__Lachnospiraceae	unclassified_f__Lachnospiraceae	OTU726	9	1	18	19
Firmicutes	Clostridia	Clostridiales	Ruminococcaceae	norank_f__Ruminococcaceae	Ruminococcaceae_bacterium_GD7	OTU72	0	0	4	0
Firmicutes	Erysipelotrichia	Erysipelotrichales	Erysipelotrichaceae	Holdemania	unclassified_g__Holdemania	OTU702	7	5	2	25
Firmicutes	Clostridia	Clostridiales	Ruminococcaceae	[Eubacterium]_coprostanoligenes_gr	unclassified_g__[Eubacterium]_coprostanoligenes_group	OTU747	0	1	0	0
Firmicutes	Clostridia	Clostridiales	Family_XIII	Family_XIII_AD3011_group	uncultured_bacterium_g__Family_XIII_AD3011_group	OTU579	8	7	2	3
Tenericutes	Mollicutes	Mollicutes_RF9	norank_o__Mollicutes	norank_o__Mollicutes_RF9	uncultured_bacterium_g__norank_o__Mollicutes_RF9	OTU756	0	0	0	5
Actinobacteria	Actinobacteria	Coriobacteriales	Coriobacteriaceae	unclassified_f__Coriobacteriaceae	unclassified_f__Coriobacteriaceae	OTU4	0	0	0	0
Firmicutes	Clostridia	Clostridiales	Ruminococcaceae	Candidatus_Soleaferrea	uncultured_organism_g__Candidatus_Soleaferrea	OTU492	16	2	0	14
Firmicutes	Clostridia	Clostridiales	Ruminococcaceae	[Eubacterium]_coprostanoligenes_gr	unclassified_g__[Eubacterium]_coprostanoligenes_group	OTU656	9	4	1	16
Firmicutes	Erysipelotrichia	Erysipelotrichales	Erysipelotrichaceae	Erysipelatoclostridium	unclassified_g__Erysipelatoclostridium	OTU506	0	0	0	0
Firmicutes	Clostridia	Clostridiales	Ruminococcaceae	Anaerotruncus	uncultured_bacterium_g__Anaerotruncus	OTU42	3	7	29	12
Firmicutes	Clostridia	Clostridiales	Lachnospiraceae	unclassified_f__Lachnospiraceae	unclassified_f__Lachnospiraceae	OTU509	2	2	3	1
Firmicutes	Clostridia	Clostridiales	Lachnospiraceae	Tyzzerella	uncultured_bacterium_g__Tyzzerella	OTU777	0	18	11	4
Firmicutes	Clostridia	Clostridiales	Clostridiales_vadinBB6	norank_f__Clostridiales_vadinBB60_	gut_metagenome_g__norank_f__Clostridiales_vadinBB60_group	OTU19	31	227	7	0
Firmicutes	Clostridia	Clostridiales	Ruminococcaceae	Ruminococcus_2	unclassified_g__Ruminococcus_2	OTU549	0	0	0	0
Firmicutes	Clostridia	Clostridiales	Ruminococcaceae	Anaerofilum	uncultured_bacterium_g__Anaerofilum	OTU677	13	12	2	12
Firmicutes	Clostridia	Clostridiales	Ruminococcaceae	Ruminococcaceae_UCG-014	unclassified_g__Ruminococcaceae_UCG-014	OTU487	0	5	16	11
Firmicutes	Clostridia	Clostridiales	Ruminococcaceae	Ruminococcaceae_UCG-013	unclassified_g__Ruminococcaceae_UCG-013	OTU145	2	4	2	1
Bacteroidetes	Bacteroidia	Bacteroidales	Rikenellaceae	Alistipes	unclassified_g__Alistipes	OTU667	7	20	3	0
Firmicutes	Clostridia	Clostridiales	Lachnospiraceae	unclassified_f__Lachnospiraceae	unclassified_f__Lachnospiraceae	OTU771	1	0	1	2
Firmicutes	Clostridia	Clostridiales	Ruminococcaceae	Ruminococcaceae_UCG-009	uncultured_bacterium_g__Ruminococcaceae_UCG-009	OTU750	0	17	18	12
Tenericutes	Mollicutes	Mollicutes_RF9	norank_o__Mollicutes	norank_o__Mollicutes_RF9	uncultured_bacterium_g__norank_o__Mollicutes_RF9	OTU543	0	0	20	1
Firmicutes	Clostridia	Clostridiales	unclassified_o__Clostr	unclassified_o__Clostridiales	unclassified_o__Clostridiales	OTU535	17	0	6	2
Tenericutes	Mollicutes	Mollicutes_RF9	norank_o__Mollicutes	norank_o__Mollicutes_RF9	uncultured_bacterium_g__norank_o__Mollicutes_RF9	OTU153	0	0	7	0
Firmicutes	Clostridia	Clostridiales	Clostridiales_vadinBB6	norank_f__Clostridiales_vadinBB60_	uncultured_bacterium_g__norank_f__Clostridiales_vadinBB60_group	OTU409	0	7	4	2
Firmicutes	Clostridia	Clostridiales	Ruminococcaceae	Oscillibacter	unclassified_g__Oscillibacter	OTU609	0	0	0	0
Firmicutes	Clostridia	Clostridiales	Ruminococcaceae	Butyricicoccus	unclassified_g__Butyricicoccus	OTU411	132	5	0	3
Firmicutes	Clostridia	Clostridiales	unclassified_o__Clostr	unclassified_o__Clostridiales	unclassified_o__Clostridiales	OTU34	2	2	9	1
Firmicutes	Clostridia	Clostridiales	Clostridiales_vadinBB6	norank_f__Clostridiales_vadinBB60_	uncultured_rumen_bacterium_g__norank_f__Clostridiales_vadinBB60_group	OTU517	0	0	0	0
Firmicutes	Clostridia	Clostridiales	Ruminococcaceae	Ruminococcaceae_UCG-014	uncultured_bacterium_g__Ruminococcaceae_UCG-014	OTU850	0	0	3	28
Firmicutes	Clostridia	Clostridiales	Ruminococcaceae	Ruminiclostridium_5	uncultured_organism_g__Ruminiclostridium_5	OTU541	9	9	1	3
Firmicutes	Clostridia	Clostridiales	Lachnospiraceae	Tyzzerella_3	uncultured_bacterium_g__Tyzzerella_3	OTU635	9	1	13	1
Firmicutes	Clostridia	Clostridiales	Eubacteriaceae	Anaerofustis	Anaerofustis_sp._Marseille-P2441	OTU136	0	5	2	3
Firmicutes	Clostridia	Clostridiales	Ruminococcaceae	Ruminococcaceae_UCG-013	unclassified_g__Ruminococcaceae_UCG-013	OTU76	1	0	0	13
Firmicutes	Clostridia	Clostridiales	Ruminococcaceae	Anaerotruncus	unclassified_g__Anaerotruncus	OTU542	0	5	20	14
Firmicutes	Clostridia	Clostridiales	Ruminococcaceae	unclassified_f__Ruminococcaceae	unclassified_f__Ruminococcaceae	OTU831	11	1	0	11
Firmicutes	Clostridia	Clostridiales	Ruminococcaceae	Ruminococcaceae_NK4A214_group	unclassified_g__Ruminococcaceae_NK4A214_group	OTU168	4	12	10	17
Firmicutes	Clostridia	Clostridiales	Ruminococcaceae	Ruminococcaceae_UCG-009	unclassified_g__Ruminococcaceae_UCG-009	OTU71	0	0	2	0
Firmicutes	Clostridia	Clostridiales	Ruminococcaceae	Ruminococcaceae_UCG-013	uncultured_organism_g__Ruminococcaceae_UCG-013	OTU204	0	0	2	0
Actinobacteria	Actinobacteria	Coriobacteriales	Coriobacteriaceae	Enterorhabdus	uncultured_bacterium_g__Enterorhabdus	OTU221	2	6	1	4
Tenericutes	Mollicutes	Mollicutes_RF9	norank_o__Mollicutes	norank_o__Mollicutes_RF9	unclassified_g__norank_o__Mollicutes_RF9	OTU788	0	2	1	20
Firmicutes	Clostridia	Clostridiales	Ruminococcaceae	Ruminococcaceae_UCG-010	uncultured_organism_g__Ruminococcaceae_UCG-010	OTU20	0	3	4	4
Firmicutes	Clostridia	Clostridiales	Ruminococcaceae	Anaerotruncus	uncultured_bacterium_g__Anaerotruncus	OTU582	20	0	8	3
Firmicutes	Clostridia	Clostridiales	Family_XIII	Family_XIII_AD3011_group	uncultured_bacterium_g__Family_XIII_AD3011_group	OTU634	4	6	12	7
Firmicutes	Clostridia	Clostridiales	Ruminococcaceae	Anaerofilum	uncultured_bacterium_g__Anaerofilum	OTU832	1	0	0	0
Firmicutes	Clostridia	Clostridiales	Clostridiales_vadinBB6	norank_f__Clostridiales_vadinBB60_	uncultured_bacterium_g__norank_f__Clostridiales_vadinBB60_group	OTU38	0	0	0	0
Firmicutes	Clostridia	Clostridiales	unclassified_o__Clostr	unclassified_o__Clostridiales	unclassified_o__Clostridiales	OTU672	0	2	36	21
Firmicutes	Clostridia	Clostridiales	Ruminococcaceae	unclassified_f__Ruminococcaceae	unclassified_f__Ruminococcaceae	OTU661	5	5	19	6
Firmicutes	Clostridia	Clostridiales	Ruminococcaceae	unclassified_f__Ruminococcaceae	unclassified_f__Ruminococcaceae	OTU169	0	5	6	0
Tenericutes	Mollicutes	Mollicutes_RF9	norank_o__Mollicutes	norank_o__Mollicutes_RF9	uncultured_bacterium_g__norank_o__Mollicutes_RF9	OTU820	0	0	0	0
Firmicutes	Clostridia	Clostridiales	Eubacteriaceae	Eubacterium	Eubacterium_limosum	OTU337	4	12	3	0
Tenericutes	Mollicutes	Mollicutes_RF9	norank_o__Mollicutes	norank_o__Mollicutes_RF9	uncultured_bacterium_g__norank_o__Mollicutes_RF9	OTU584	5	0	2	5
Firmicutes	Clostridia	Clostridiales	Ruminococcaceae	Anaerofilum	uncultured_bacterium_g__Anaerofilum	OTU643	0	9	19	5
Firmicutes	Clostridia	Clostridiales	Clostridiales_vadinBB6	norank_f__Clostridiales_vadinBB60_	uncultured_bacterium_g__norank_f__Clostridiales_vadinBB60_group	OTU35	0	5	4	18
Firmicutes	Clostridia	Clostridiales	Lachnospiraceae	Coprococcus_1	uncultured_bacterium_g__Coprococcus_1	OTU811	8	1	1	1
Firmicutes	Clostridia	Clostridiales	Ruminococcaceae	Ruminococcaceae_UCG-014	unclassified_g__Ruminococcaceae_UCG-014	OTU87	0	20	0	6
Firmicutes	Clostridia	Clostridiales	Ruminococcaceae	[Eubacterium]_coprostanoligenes_gr	unclassified_g__[Eubacterium]_coprostanoligenes_group	OTU751	0	0	14	58
Firmicutes	Clostridia	Clostridiales	Clostridiales_vadinBB6	norank_f__Clostridiales_vadinBB60_	uncultured_bacterium_g__norank_f__Clostridiales_vadinBB60_group	OTU694	0	0	0	0
Firmicutes	Clostridia	Clostridiales	Clostridiales_vadinBB6	norank_f__Clostridiales_vadinBB60_	uncultured_Thermoanaerobacterales_bacterium_g__norank_f__Clostridiales_vadinBB60_gro	OTU224	0	4	2	1
Firmicutes	Clostridia	Clostridiales	Lachnospiraceae	unclassified_f__Lachnospiraceae	unclassified_f__Lachnospiraceae	OTU655	4	5	6	4
Firmicutes	Clostridia	Clostridiales	Ruminococcaceae	Oscillospira	uncultured_bacterium_g__Oscillospira	OTU574	0	3	0	0
Firmicutes	Clostridia	Clostridiales	Clostridiales_vadinBB6	norank_f__Clostridiales_vadinBB60_	unclassified_g__norank_f__Clostridiales_vadinBB60_group	OTU2	0	0	0	0
Firmicutes	Clostridia	Clostridiales	Clostridiales_vadinBB6	norank_f__Clostridiales_vadinBB60_	unclassified_g__norank_f__Clostridiales_vadinBB60_group	OTU778	0	0	0	0
Firmicutes	Clostridia	Clostridiales	Ruminococcaceae	Ruminococcaceae_UCG-013	unclassified_g__Ruminococcaceae_UCG-013	OTU676	0	0	0	0
Tenericutes	Mollicutes	Mollicutes_RF9	norank_o__Mollicutes	norank_o__Mollicutes_RF9	unclassified_g__norank_o__Mollicutes_RF9	OTU626	0	4	17	9
Firmicutes	Clostridia	Clostridiales	Ruminococcaceae	Ruminococcaceae_UCG-010	unclassified_g__Ruminococcaceae_UCG-010	OTU61	0	6	22	3
Firmicutes	Clostridia	Clostridiales	Ruminococcaceae	norank_f__Ruminococcaceae	unclassified_g__norank_f__Ruminococcaceae	OTU516	0	1	0	10
Firmicutes	Clostridia	Clostridiales	Ruminococcaceae	unclassified_f__Ruminococcaceae	unclassified_f__Ruminococcaceae	OTU228	0	14	15	15
Firmicutes	Clostridia	Clostridiales	Ruminococcaceae	Ruminococcaceae_UCG-010	uncultured_bacterium_g__Ruminococcaceae_UCG-010	OTU203	0	0	0	0
Firmicutes	Clostridia	Clostridiales	Ruminococcaceae	Anaerotruncus	uncultured_bacterium_g__Anaerotruncus	OTU853	0	10	0	53
Firmicutes	Clostridia	Thermoanaerobacte	Thermoanaerobactera	Gelria	uncultured_bacterium_g__Gelria	OTU16	0	13	2	0
Firmicutes	Clostridia	Clostridiales	Clostridiales_vadinBB6	norank_f__Clostridiales_vadinBB60_	uncultured_bacterium_g__norank_f__Clostridiales_vadinBB60_group	OTU143	0	0	0	0
Firmicutes	Clostridia	Clostridiales	Family_XIII	unclassified_f__Family_XIII	unclassified_f__Family_XIII	OTU537	2	5	1	0
Firmicutes	Clostridia	Clostridiales	Family_XIII	[Eubacterium]_brachy_group	uncultured_bacterium_g__[Eubacterium]_brachy_group	OTU69	9	5	4	2
Firmicutes	Clostridia	Clostridiales	Ruminococcaceae	unclassified_f__Ruminococcaceae	unclassified_f__Ruminococcaceae	OTU14	0	1	1	1
Firmicutes	Clostridia	Clostridiales	Ruminococcaceae	Intestinimonas	uncultured_bacterium_g__Intestinimonas	OTU615	8	11	6	5
Firmicutes	Clostridia	Clostridiales	Lachnospiraceae	unclassified_f__Lachnospiraceae	unclassified_f__Lachnospiraceae	OTU118	8	3	0	8
Firmicutes	Clostridia	Clostridiales	Ruminococcaceae	Anaerotruncus	unclassified_g__Anaerotruncus	OTU505	6	1	0	5
Firmicutes	Bacilli	Lactobacillales	Lactobacillaceae	Lactobacillus	uncultured_bacterium_g__Lactobacillus	OTU637	0	1	0	0
Firmicutes	Clostridia	Clostridiales	Ruminococcaceae	Ruminococcaceae_V9D2013_group	uncultured_bacterium_g__Ruminococcaceae_V9D2013_group	OTU652	0	6	5	6
Firmicutes	Erysipelotrichia	Erysipelotrichales	Erysipelotrichaceae	norank_f__Erysipelotrichaceae	uncultured_Erysipelotrichales_bacterium_g__norank	OTU323	0	0	90	109
Firmicutes	Clostridia	Clostridiales	Ruminococcaceae	Papillibacter	uncultured_bacterium_g__Papillibacter	OTU715	2	4	8	6
Tenericutes	Mollicutes	Mollicutes_RF9	norank_o__Mollicutes	norank_o__Mollicutes_RF9	unclassified_g__norank_o__Mollicutes_RF9	OTU729	0	0	0	0
Cyanobacteria	Cyanobacteria	Gastranaerophilales	norank_o__Gastranae	norank_o__Gastranaerophilales	uncultured_bacterium_g__norank_o__Gastranaerophilales	OTU94	0	0	0	0
Firmicutes	Clostridia	Clostridiales	Ruminococcaceae	Ruminococcaceae_UCG-013	uncultured_bacterium_g__Ruminococcaceae_UCG-013	OTU780	0	8	3	4
Firmicutes	Clostridia	Clostridiales	Ruminococcaceae	Anaerotruncus	unclassified_g__Anaerotruncus	OTU723	3	2	8	12
Firmicutes	Clostridia	Clostridiales	Ruminococcaceae	Anaerotruncus	unclassified_g__Anaerotruncus	OTU101	2	4	9	8
Tenericutes	Mollicutes	Mollicutes_RF9	norank_o__Mollicutes	norank_o__Mollicutes_RF9	unclassified_g__norank_o__Mollicutes_RF9	OTU117	0	0	6	5
Firmicutes	Clostridia	Clostridiales	Ruminococcaceae	unclassified_f__Ruminococcaceae	unclassified_f__Ruminococcaceae	OTU93	0	0	2	0
Firmicutes	Clostridia	Clostridiales	Lachnospiraceae	unclassified_f__Lachnospiraceae	unclassified_f__Lachnospiraceae	OTU759	4	0	0	1
Firmicutes	Clostridia	Clostridiales	Ruminococcaceae	Ruminococcaceae_UCG-014	unclassified_g__Ruminococcaceae_UCG-014	OTU95	0	0	0	0
Firmicutes	Erysipelotrichia	Erysipelotrichales	Erysipelotrichaceae	Dielma	unclassified_g__Dielma	OTU173	0	0	0	0
Firmicutes	Clostridia	Clostridiales	Ruminococcaceae	Anaerotruncus	unclassified_g__Anaerotruncus	OTU124	5	10	6	10
Firmicutes	Clostridia	Clostridiales	Ruminococcaceae	norank_f__Ruminococcaceae	unclassified_g__norank_f__Ruminococcaceae	OTU134	4	0	0	0
Firmicutes	Clostridia	Clostridiales	Lachnospiraceae	unclassified_f__Lachnospiraceae	unclassified_f__Lachnospiraceae	OTU510	1	1	0	3
Actinobacteria	Actinobacteria	Coriobacteriales	Coriobacteriaceae	Gordonibacter	unclassified_g__Gordonibacter	OTU461	2	6	1	5
Proteobacteria	Betaproteobacteria	Burkholderiales	Burkholderiaceae	Burkholderia-Paraburkholderia	unclassified_g__Burkholderia-Paraburkholderia	OTU435	0	0	67	98
Firmicutes	Clostridia	Clostridiales	Family_XIII	Family_XIII_AD3011_group	uncultured_bacterium_g__Family_XIII_AD3011_group	OTU648	0	0	0	0
Firmicutes	Clostridia	Clostridiales	Ruminococcaceae	unclassified_f__Ruminococcaceae	unclassified_f__Ruminococcaceae	OTU722	0	1	0	17
Firmicutes	Clostridia	Clostridiales	Clostridiales_vadinBB6	norank_f__Clostridiales_vadinBB60_	uncultured_bacterium_g__norank_f__Clostridiales_vadinBB60_group	OTU740	0	1	0	3
Bacteroidetes	Bacteroidia	Bacteroidales	Bacteroidales_S24-7_g	norank_f__Bacteroidales_S24-7_gro	uncultured_bacterium_g__norank_f__Bacteroidales_S24-7_group	OTU322	0	0	88	76
Firmicutes	Clostridia	Clostridiales	Ruminococcaceae	unclassified_f__Ruminococcaceae	unclassified_f__Ruminococcaceae	OTU538	1	5	1	0
Firmicutes	Clostridia	Clostridiales	Lachnospiraceae	unclassified_f__Lachnospiraceae	unclassified_f__Lachnospiraceae	OTU255	0	1	21	12
Firmicutes	Clostridia	Clostridiales	Christensenellaceae	Christensenellaceae_R-7_group	unclassified_g__Christensenellaceae_R-7_group	OTU106	0	0	0	0

Firmicutes	Clostridia	Clostridiales	Ruminococcaceae	[Eubacterium]_coprostanoligenes_g	uncultured_bacterium_g__[Eubacterium]_coprostanoligenes_group	OTU129	0	15	0	8
Firmicutes	Erysipelotrichia	Erysipelotrichales	Erysipelotrichaceae	Erysipelatoclostridium	Clostridiales_bacterium_CHKCI006	OTU232	0	1	0	0
Firmicutes	Clostridia	Clostridiales	Ruminococcaceae	unclassified_f__Ruminococcaceae	unclassified_f__Ruminococcaceae	OTU518	2	2	3	3
Firmicutes	Clostridia	Clostridiales	Ruminococcaceae	Ruminococcaceae_UCG-013	uncultured_Clostridiaceae_bacterium_g__Ruminococcaceae_UCG-013	OTU675	0	1	15	0
Firmicutes	Clostridia	Clostridiales	Christensenellaceae	Christensenellaceae_R-7_group	human_gut_metagenome_g__Christensenellaceae_R-7_group	OTU223	0	0	0	0
Firmicutes	Clostridia	Clostridiales	Ruminococcaceae	Faecalibacterium	uncultured_bacterium_g__Faecalibacterium	OTU41	9	5	3	3
Firmicutes	Clostridia	Clostridiales	Ruminococcaceae	Anaerotruncus	unclassified_g__Anaerotruncus	OTU647	0	0	0	0
Firmicutes	Erysipelotrichia	Erysipelotrichales	Erysipelotrichaceae	Turicibacter	uncultured_bacterium_g__Turicibacter	OTU598	0	0	1	1
Firmicutes	Clostridia	Clostridiales	Ruminococcaceae	Subdoligranulum	unclassified_g__Subdoligranulum	OTU188	10	0	0	2
Firmicutes	Clostridia	Clostridiales	Ruminococcaceae	Anaerotruncus	uncultured_Clostridiaceae_bacterium_g__Anaerotruncus	OTU235	1	9	0	2
Firmicutes	Clostridia	Clostridiales	Family_XIII	unclassified_f__Family_XIII	unclassified_f__Family_XIII	OTU236	0	1	2	10
Firmicutes	Clostridia	Clostridiales	Ruminococcaceae	Ruminococcaceae_UCG-014	unidentified_rumen_bacterium_JW32	OTU21	0	0	37	0
Firmicutes	Clostridia	Clostridiales	Ruminococcaceae	Papillibacter	unclassified_g__Papillibacter	OTU521	0	1	0	0
Firmicutes	Clostridia	Clostridiales	Ruminococcaceae	unclassified_f__Ruminococcaceae	unclassified_f__Ruminococcaceae	OTU653	2	4	2	9
Firmicutes	Clostridia	Clostridiales	Peptococcaceae	norank_f__Peptococcaceae	uncultured_bacterium_g__norank_f__Peptococcaceae	OTU605	0	0	8	0
Firmicutes	Bacilli	Lactobacillales	Lactobacillaceae	Lactobacillus	Lactobacillus_ingluviei	OTU182	0	0	0	0
Firmicutes	Clostridia	Clostridiales	Ruminococcaceae	[Eubacterium]_coprostanoligenes_g	unclassified_g__[Eubacterium]_coprostanoligenes_group	OTU333	0	0	0	0
Firmicutes	Clostridia	Clostridiales	unclassified_o__Clostr	unclassified_o__Clostridiales	unclassified_o__Clostridiales	OTU472	10	0	1	0
Firmicutes	Clostridia	Clostridiales	Ruminococcaceae	[Eubacterium]_coprostanoligenes_g	unclassified_g__[Eubacterium]_coprostanoligenes_group	OTU730	1	0	0	2
Firmicutes	Clostridia	Clostridiales	Peptococcaceae	norank_f__Peptococcaceae	unclassified_g__norank_f__Peptococcaceae	OTU172	0	2	0	1
Firmicutes	Clostridia	Clostridiales	Ruminococcaceae	Ruminococcaceae_UCG-013	uncultured_bacterium_g__Ruminococcaceae_UCG-013	OTU658	0	1	0	0
Proteobacteria	Gammaproteobacteria	Enterobacteriales	Enterobacteriaceae	Proteus	Proteus_mirabilis	OTU785	0	0	5	8
Firmicutes	Clostridia	Clostridiales	Ruminococcaceae	norank_f__Ruminococcaceae	unclassified_g__norank_f__Ruminococcaceae	OTU744	1	2	1	4
Firmicutes	Clostridia	Clostridiales	Lachnospiraceae	Tyzzerella_3	unclassified_g__Tyzzerella_3	OTU841	12	0	0	0
Bacteroidetes	Bacteroidia	Bacteroidales	Bacteroidales_S24-7_g	norank_f__Bacteroidales_S24-7_gro	uncultured_bacterium_g__norank_f__Bacteroidales_S24-7_group	OTU360	0	0	46	69
Firmicutes	Clostridia	Clostridiales	Ruminococcaceae	unclassified_f__Ruminococcaceae	unclassified_f__Ruminococcaceae	OTU737	1	4	2	6
Firmicutes	Clostridia	Clostridiales	Ruminococcaceae	Anaerotruncus	unclassified_g__Anaerotruncus	OTU752	3	3	2	5
Firmicutes	Clostridia	Clostridiales	Lachnospiraceae	unclassified_f__Lachnospiraceae	unclassified_f__Lachnospiraceae	OTU63	3	0	1	2
Firmicutes	Clostridia	Clostridiales	Ruminococcaceae	Caproiciproducens	unclassified_g__Caproiciproducens	OTU452	7	0	1	0
Firmicutes	Clostridia	Clostridiales	Ruminococcaceae	Butyricicoccus	unclassified_g__Butyricicoccus	OTU475	0	0	0	0
Proteobacteria	Betaproteobacteria	Burkholderiales	Oxalobacteraceae	Herminiimonas	uncultured_bacterium_g__Herminiimonas	OTU804	0	3	6	2
Tenericutes	Mollicutes	Mollicutes_RF9	norank_o__Mollicutes	norank_o__Mollicutes_RF9	unclassified_g__norank_o__Mollicutes_RF9	OTU630	0	0	7	1
Firmicutes	Clostridia	Clostridiales	Ruminococcaceae	Ruminococcaceae_UCG-013	unclassified_g__Ruminococcaceae_UCG-013	OTU680	0	9	3	2
Tenericutes	Mollicutes	Mollicutes_RF9	norank_o__Mollicutes	norank_o__Mollicutes_RF9	gut_metagenome_g__norank_o__Mollicutes_RF9	OTU773	0	0	0	5
Firmicutes	Clostridia	Clostridiales	Peptococcaceae	norank_f__Peptococcaceae	uncultured_bacterium_g__norank_f__Peptococcaceae	OTU624	0	4	4	5
Firmicutes	Clostridia	Clostridiales	Ruminococcaceae	Ruminiclostridium	unclassified_g__Ruminiclostridium	OTU154	1	3	2	5
Bacteroidetes	Bacteroidia	Bacteroidales	Bacteroidales_S24-7_g	norank_f__Bacteroidales_S24-7_gro	unclassified_g__norank_f__Bacteroidales_S24-7_group	OTU184	0	0	40	48
Firmicutes	Clostridia	Clostridiales	Ruminococcaceae	Ruminococcaceae_UCG-014	unclassified_g__Ruminococcaceae_UCG-014	OTU524	6	1	2	0
Firmicutes	Clostridia	Clostridiales	Peptococcaceae	norank_f__Peptococcaceae	uncultured_bacterium_g__norank_f__Peptococcaceae	OTU115	0	0	1	3
Firmicutes	Clostridia	Clostridiales	Lachnospiraceae	unclassified_f__Lachnospiraceae	unclassified_f__Lachnospiraceae	OTU127	0	0	0	0
Firmicutes	Clostridia	Clostridiales	Lachnospiraceae	unclassified_f__Lachnospiraceae	unclassified_f__Lachnospiraceae	OTU664	1	3	6	4
Firmicutes	Clostridia	Clostridiales	Lachnospiraceae	unclassified_f__Lachnospiraceae	unclassified_f__Lachnospiraceae	OTU465	1	3	2	1
Firmicutes	Clostridia	Clostridiales	Ruminococcaceae	Ruminococcaceae_UCG-005	uncultured_Clostridiales_bacterium_g__Ruminococcaceae_UCG-005	OTU689	0	2	2	2
Tenericutes	Mollicutes	Mollicutes_RF9	norank_o__Mollicutes	norank_o__Mollicutes_RF9	uncultured_bacterium_g__norank_o__Mollicutes_RF9	OTU52	0	2	9	6
Firmicutes	Clostridia	Clostridiales	Ruminococcaceae	[Eubacterium]_coprostanoligenes_g	unclassified_g__[Eubacterium]_coprostanoligenes_group	OTU74	0	3	2	2
Firmicutes	Clostridia	Clostridiales	Lachnospiraceae	Coprococcus_1	unclassified_g__Coprococcus_1	OTU659	0	0	5	0
Firmicutes	Clostridia	Clostridiales	Lachnospiraceae	unclassified_f__Lachnospiraceae	unclassified_f__Lachnospiraceae	OTU688	0	1	1	0
Firmicutes	Clostridia	Clostridiales	Ruminococcaceae	unclassified_f__Ruminococcaceae	unclassified_f__Ruminococcaceae	OTU732	2	5	0	1
Firmicutes	Clostridia	Clostridiales	Christensenellaceae	Christensenellaceae_R-7_group	uncultured_organism_g__Christensenellaceae_R-7_group	OTU684	1	6	2	2
Bacteroidetes	Bacteroidia	Bacteroidales	Porphyromonadaceae	Odoribacter	uncultured_bacterium_g__Odoribacter	OTU327	0	0	0	0
Firmicutes	Clostridia	Clostridiales	Clostridiales_vadinBB	norank_f__Clostridiales_vadinBB60_	uncultured_bacterium_g__norank_f__Clostridiales_vadinBB60_group	OTU325	0	5	0	0
Firmicutes	Clostridia	Clostridiales	Lachnospiraceae	unclassified_f__Lachnospiraceae	unclassified_f__Lachnospiraceae	OTU551	0	10	1	0
Firmicutes	Bacilli	Lactobacillales	Enterococcaceae	Enterococcus	unclassified_g__Enterococcus	OTU237	0	0	1	0
Bacteroidetes	Bacteroidia	Bacteroidales	Prevotellaceae	Alloprevotella	gut_metagenome_g__Alloprevotella	OTU312	0	0	46	35
Firmicutes	Clostridia	Clostridiales	Ruminococcaceae	unclassified_f__Ruminococcaceae	unclassified_f__Ruminococcaceae	OTU855	7	1	1	0
Actinobacteria	Actinobacteria	Bifidobacteriales	Bifidobacteriaceae	Bifidobacterium	Bifidobacterium_pseudolongum_PV8-2	OTU468	0	0	38	37
Firmicutes	Clostridia	Clostridiales	Christensenellaceae	norank_f__Christensenellaceae	unclassified_g__norank_f__Christensenellaceae	OTU529	2	1	3	1
Tenericutes	Mollicutes	Mollicutes_RF9	norank_o__Mollicutes	norank_o__Mollicutes_RF9	unclassified_g__norank_o__Mollicutes_RF9	OTU764	0	0	0	0
Firmicutes	Clostridia	Clostridiales	Ruminococcaceae	norank_f__Ruminococcaceae	unclassified_g__norank_f__Ruminococcaceae	OTU805	0	4	2	0
Firmicutes	Clostridia	Clostridiales	Ruminococcaceae	Faecalibacterium	uncultured_bacterium_g__Faecalibacterium	OTU60	0	0	2	0
Firmicutes	Clostridia	Clostridiales	Ruminococcaceae	unclassified_f__Ruminococcaceae	unclassified_f__Ruminococcaceae	OTU530	0	6	1	1
Firmicutes	Clostridia	Clostridiales	Peptococcaceae	norank_f__Peptococcaceae	uncultured_bacterium_g__norank_f__Peptococcaceae	OTU619	0	4	2	3
Firmicutes	Clostridia	Clostridiales	Ruminococcaceae	unclassified_f__Ruminococcaceae	unclassified_f__Ruminococcaceae	OTU471	0	0	0	0
Firmicutes	Clostridia	Clostridiales	Ruminococcaceae	Candidatus_Soleaferrea	uncultured_bacterium_g__Candidatus_Soleaferrea	OTU284	1	0	4	0
Firmicutes	Clostridia	Clostridiales	Ruminococcaceae	Anaerotruncus	unclassified_g__Anaerotruncus	OTU508	2	0	0	1
Firmicutes	Clostridia	Clostridiales	Ruminococcaceae	Anaerotruncus	uncultured_bacterium_g__Anaerotruncus	OTU704	3	1	0	3
Tenericutes	Mollicutes	Mollicutes_RF9	norank_o__Mollicutes	norank_o__Mollicutes_RF9	uncultured_bacterium_g__norank_o__Mollicutes_RF9	OTU783	0	0	0	0
Firmicutes	Clostridia	Clostridiales	Eubacteriaceae	Anaerofustis	uncultured_bacterium_g__Anaerofustis	OTU111	0	0	0	0
Firmicutes	Clostridia	Clostridiales	Clostridiales_vadinBB	norank_f__Clostridiales_vadinBB60_	unclassified_g__norank_f__Clostridiales_vadinBB60_group	OTU645	0	0	0	2
Firmicutes	Clostridia	Clostridiales	Peptococcaceae	norank_f__Peptococcaceae	uncultured_bacterium_g__norank_f__Peptococcaceae	OTU27	0	1	1	1
Firmicutes	Clostridia	Clostridiales	Ruminococcaceae	Anaerotruncus	unclassified_g__Anaerotruncus	OTU809	0	0	0	0
Bacteroidetes	Bacteroidia	Bacteroidales	Rikenellaceae	Rikenellaceae_RC9_gut_group	unclassified_g__Rikenellaceae_RC9_gut_group	OTU321	0	0	38	28
Firmicutes	Clostridia	Clostridiales	Ruminococcaceae	Ruminococcaceae_UCG-005	unclassified_g__Ruminococcaceae_UCG-005	OTU482	0	0	1	1
Tenericutes	Mollicutes	Mollicutes_RF9	norank_o__Mollicutes	norank_o__Mollicutes_RF9	uncultured_Erysipelotrichaceae_bacterium_g__norank_o__Mollicutes_RF9	OTU558	0	0	2	2
Firmicutes	Clostridia	Clostridiales	Clostridiaceae_1	Candidatus_Arthromitus	Candidatus_Arthromitus_sp._SFB-mouse-Japan	OTU162	6	0	0	0
Firmicutes	Clostridia	Clostridiales	Ruminococcaceae	Subdoligranulum	unclassified_g__Subdoligranulum	OTU810	0	1	4	1
Firmicutes	Clostridia	Clostridiales	Ruminococcaceae	norank_f__Ruminococcaceae	unclassified_g__norank_f__Ruminococcaceae	OTU666	2	2	0	0
Firmicutes	Clostridia	Clostridiales	Ruminococcaceae	unclassified_f__Ruminococcaceae	unclassified_f__Ruminococcaceae	OTU693	4	0	0	3
Firmicutes	Clostridia	Clostridiales	Ruminococcaceae	unclassified_f__Ruminococcaceae	unclassified_f__Ruminococcaceae	OTU858	0	1	3	3
Firmicutes	Clostridia	Clostridiales	Ruminococcaceae	Ruminiclostridium_5	unclassified_g__Ruminiclostridium_5	OTU470	2	0	0	1
Firmicutes	Negativicutes	Selenomonadales	Acidaminococcaceae	Phascolarctobacterium	uncultured_organism_g__Phascolarctobacterium	OTU787	0	1	2	0
Firmicutes	Clostridia	Clostridiales	Christensenellaceae	Christensenellaceae_R-7_group	unclassified_g__Christensenellaceae_R-7_group	OTU745	0	0	1	2
Firmicutes	Clostridia	Clostridiales	Peptococcaceae	norank_f__Peptococcaceae	unclassified_g__norank_f__Peptococcaceae	OTU495	0	0	0	0
Firmicutes	Clostridia	Clostridiales	Ruminococcaceae	Ruminococcaceae_UCG-010	uncultured_bacterium_g__Ruminococcaceae_UCG-010	OTU18	0	1	0	0
Firmicutes	Clostridia	Clostridiales	Ruminococcaceae	Ruminococcaceae_UCG-013	unclassified_g__Ruminococcaceae_UCG-013	OTU89	0	0	0	0
Firmicutes	Clostridia	Clostridiales	Ruminococcaceae	Ruminiclostridium_5	unclassified_g__Ruminiclostridium_5	OTU649	0	0	6	1
Firmicutes	Clostridia	Clostridiales	Lachnospiraceae	unclassified_f__Lachnospiraceae	unclassified_f__Lachnospiraceae	OTU324	4	0	1	0
Firmicutes	Clostridia	Clostridiales	Ruminococcaceae	Ruminococcaceae_UCG-013	unclassified_g__Ruminococcaceae_UCG-013	OTU31	0	0	1	0
Firmicutes	Clostridia	Clostridiales	Ruminococcaceae	Ruminococcus_1	uncultured_organism_g__Ruminococcus_1	OTU651	0	0	0	1
Firmicutes	Clostridia	Clostridiales	Peptococcaceae	unclassified_f__Peptococcaceae	unclassified_f__Peptococcaceae	OTU768	0	1	2	6
Firmicutes	Clostridia	Clostridiales	Christensenellaceae	unclassified_f__Christensenellaceae	unclassified_f__Christensenellaceae	OTU857	0	0	0	0
Tenericutes	Mollicutes	Mollicutes_RF9	norank_o__Mollicutes	norank_o__Mollicutes_RF9	unclassified_g__norank_o__Mollicutes_RF9	OTU758	0	0	0	0
Firmicutes	Clostridia	Clostridiales	Ruminococcaceae	Candidatus_Soleaferrea	uncultured_bacterium_g__Candidatus_Soleaferrea	OTU791	0	0	2	6
unclassified_k__r	unclassified_k__norank	unclassified_k__nor	unclassified_k__norar	unclassified_k__norank	unclassified_k__norank	OTU9	1	0	0	0
Firmicutes	Clostridia	Clostridiales	Ruminococcaceae	unclassified_f__Ruminococcaceae	unclassified_f__Ruminococcaceae	OTU733	0	1	0	2
Firmicutes	Clostridia	Clostridiales	Peptococcaceae	norank_f__Peptococcaceae	unclassified_g__norank_f__Peptococcaceae	OTU90	0	0	0	0
Firmicutes	Clostridia	Clostridiales	Lachnospiraceae	unclassified_f__Lachnospiraceae	unclassified_f__Lachnospiraceae	OTU644	0	0	0	0
Firmicutes	Clostridia	Clostridiales	Ruminococcaceae	[Eubacterium]_coprostanoligenes_g	unclassified_g__[Eubacterium]_coprostanoligenes_group	OTU29	1	1	0	3
Firmicutes	Clostridia	Clostridiales	Ruminococcaceae	Ruminococcaceae_UCG-013	uncultured_Clostridiaceae_bacterium_g__Ruminococcaceae_UCG-013	OTU152	0	0	0	0
Firmicutes	Clostridia	Clostridiales	Ruminococcaceae	Faecalibacterium	unclassified_g__Faecalibacterium	OTU243	0	3	0	0
Bacteroidetes	Bacteroidia	Bacteroidales	Bacteroidales_S24-7_g	norank_f__Bacteroidales_S24-7_gro	uncultured_Bacteroidales_bacterium_g__norank_f__Bacteroidales_S24-7_group	OTU339	0	0	24	22
Tenericutes	Mollicutes	Mollicutes_RF9	norank_o__Mollicutes	norank_o__Mollicutes_RF9	unclassified_g__norank_o__Mollicutes_RF9	OTU51	0	0	3	0
Firmicutes	Clostridia	Clostridiales	Christensenellaceae	Christensenellaceae_R-7_group	uncultured_organism_g__Christensenellaceae_R-7_group	OTU332	0	0	0	0
Firmicutes	Clostridia	Clostridiales	Ruminococcaceae	Anaerotruncus	unclassified_g__Anaerotruncus	OTU194	1	0	0	0
Firmicutes	Clostridia	Clostridiales	Ruminococcaceae	Ruminococcaceae_UCG-013	uncultured_bacterium_g__Ruminococcaceae_UCG-013	OTU334	0	0	0	0
Firmicutes	Clostridia	Clostridiales	Ruminococcaceae	Ruminococcaceae_NK4A214_group	unclassified_g__Ruminococcaceae_NK4A214_group	OTU32	0	0	1	0
Firmicutes	Clostridia	Clostridiales	Christensenellaceae	Christensenellaceae_R-7_group	unclassified_g__Christensenellaceae_R-7_group	OTU105	0	1	3	0
Firmicutes	Bacilli	Bacillales	Staphylococcaceae	Staphylococcus	Staphylococcus_warneri	OTU597	0	0	16	12
Firmicutes	Bacilli	Bacillales	Staphylococcaceae	Staphylococcus	Staphylococcus_lentus_g__Staphylococcus	OTU432	0	0	19	26
Firmicutes	Clostridia	Clostridiales	Ruminococcaceae	unclassified_f__Ruminococcaceae	unclassified_f__Ruminococcaceae	OTU698	0	0	0	0
Tenericutes	Mollicutes	Mollicutes_RF9	norank_o__Mollicutes	norank_o__Mollicutes_RF9	unclassified_g__norank_o__Mollicutes_RF9	OTU781	0	0	0	4

Firmicutes	Clostridia	Clostridiales	Ruminococcaceae	Anaerotruncus	uncultured_bacterium_g__Anaerotruncus	OTU53	0	0	1	1
Actinobacteria	Actinobacteria	Micrococcales	Intrasporangiaceae	Terrabacter	uncultured_bacterium_g__Terrabacter	OTU270	0	0	13	31
Firmicutes	Clostridia	Clostridiales	Ruminococcaceae	unclassified_f__Ruminococcaceae	unclassified_f__Ruminococcaceae	OTU681	0	0	1	0
Bacteroidetes	Bacteroidia	Bacteroidales	Bacteroidales_S24-7_g	norank_f__Bacteroidales_S24-7_gro	uncultured_bacterium_g__norank_f__Bacteroidales_S24-7_group	OTU310	0	0	17	26
Firmicutes	Clostridia	Clostridiales	Clostridiales_vadinBB6	norank_f__Clostridiales_vadinBB60_	unclassified_g__norank_f__Clostridiales_vadinBB60_group	OTU528	0	0	0	0
Firmicutes	Clostridia	Clostridiales	Ruminococcaceae	Ruminiclostridium	uncultured_bacterium_g__Ruminiclostridium	OTU633	0	0	0	0
Firmicutes	Clostridia	Clostridiales	Christensenellaceae	Christensenellaceae_R-7_group	unclassified_g__Christensenellaceae_R-7_group	OTU695	0	2	2	2
Cyanobacteria	Cyanobacteria	norank_c__Cyanoba	norank_c__Cyanobact	norank_c__Cyanobacteria	unclassified_g__norank_c__Cyanobacteria	OTU258	0	0	29	2
Bacteroidetes	Bacteroidia	Bacteroidales	Prevotellaceae	Prevotellaceae_UCG-001	uncultured_Bacteroidales_bacterium_g__Prevotellaceae_UCG-001	OTU276	0	0	26	14
Firmicutes	Bacilli	Lactobacillales	Lactobacillaceae	Lactobacillus	unclassified_g__Lactobacillus	OTU632	0	0	0	0
Firmicutes	Clostridia	Clostridiales	Ruminococcaceae	Ruminiclostridium_9	Clostridiales_bacterium_NK3B98_g__Ruminiclostridium_9	OTU130	0	0	0	0
Firmicutes	Clostridia	Clostridiales	Ruminococcaceae	Ruminococcaceae_UCG-013	uncultured_bacterium_g__Ruminococcaceae_UCG-013	OTU807	0	1	2	0
Bacteroidetes	Bacteroidia	Bacteroidales	Rikenellaceae	Alistipes	gut_metagenome_g__Alistipes	OTU808	0	3	0	0
Firmicutes	Clostridia	unclassified_c__Clo	unclassified_c__Clostr	unclassified_c__Clostridia	unclassified_c__Clostridia	OTU99	0	0	0	0
Firmicutes	Clostridia	Clostridiales	Christensenellaceae	norank_f__Christensenellaceae	unclassified_g__norank_f__Christensenellaceae	OTU553	0	0	0	0
Firmicutes	Clostridia	Clostridiales	Lachnospiraceae	unclassified_f__Lachnospiraceae	unclassified_f__Lachnospiraceae	OTU80	2	0	0	0
Bacteroidetes	Bacteroidia	Bacteroidales	Prevotellaceae	Paraprevotella	unclassified_g__Paraprevotella	OTU308	0	0	15	22
Firmicutes	Clostridia	Clostridiales	Peptococcaceae	norank_f__Peptococcaceae	unclassified_g__norank_f__Peptococcaceae	OTU596	0	0	0	0
Firmicutes	Clostridia	Clostridiales	Lachnospiraceae	Lachnospiraceae_NK4A136_group	unclassified_g__Lachnospiraceae_NK4A136_group	OTU251	0	0	18	16
Firmicutes	Clostridia	Clostridiales	Family_XIII	[Eubacterium]_brachy_group	uncultured_bacterium_g__[Eubacterium]_brachy_group	OTU335	0	0	0	0
Firmicutes	Clostridia	Clostridiales	Lachnospiraceae	unclassified_f__Lachnospiraceae	unclassified_f__Lachnospiraceae	OTU86	0	0	1	1
Firmicutes	Clostridia	Clostridiales	Ruminococcaceae	Ruminococcaceae_UCG-005	uncultured_bacterium_adhufec311	OTU215	2	2	1	0
Firmicutes	Clostridia	Clostridiales	Ruminococcaceae	Ruminococcaceae_UCG-009	uncultured_bacterium_g__Ruminococcaceae_UCG-009	OTU641	0	0	0	4
Firmicutes	Clostridia	Clostridiales	Ruminococcaceae	Ruminiclostridium_1	unclassified_g__Ruminiclostridium_1	OTU178	0	0	1	0
Actinobacteria	Actinobacteria	Micrococcales	Micrococcaceae	Pseudarthrobacter	unclassified_g__Pseudarthrobacter	OTU423	0	1	13	18
Firmicutes	Clostridia	Clostridiales	Ruminococcaceae	Ruminococcaceae_UCG-010	unclassified_g__Ruminococcaceae_UCG-010	OTU26	0	3	0	0
Firmicutes	Clostridia	Clostridiales	Clostridiaceae_1	Clostridium_sensu_stricto_1	uncultured_bacterium_g__Clostridium_sensu_stricto_1	OTU128	0	0	1	1
Firmicutes	Clostridia	Clostridiales	Ruminococcaceae	Anaerotruncus	uncultured_bacterium_g__Anaerotruncus	OTU245	1	0	1	0
Firmicutes	Clostridia	Clostridiales	Ruminococcaceae	Ruminococcaceae_UCG-014	unclassified_g__Ruminococcaceae_UCG-014	OTU429	0	0	13	12
Firmicutes	Erysipelotrichia	Erysipelotrichales	Erysipelotrichaceae	Faecalibaculum	uncultured_bacterium_g__Faecalibaculum	OTU497	0	0	16	15
Firmicutes	Clostridia	Clostridiales	Ruminococcaceae	unclassified_f__Ruminococcaceae	unclassified_f__Ruminococcaceae	OTU525	0	0	0	0
Firmicutes	Clostridia	Clostridiales	Ruminococcaceae	Anaerotruncus	unclassified_g__Anaerotruncus	OTU640	0	0	0	0
Actinobacteria	Actinobacteria	Coriobacteriales	Coriobacteriaceae	Coriobacteriaceae_UCG-002	uncultured_bacterium_g__Coriobacteriaceae_UCG-002	OTU365	0	0	12	18
Firmicutes	Clostridia	Clostridiales	Christensenellaceae	Christensenellaceae_R-7_group	unclassified_g__Christensenellaceae_R-7_group	OTU670	0	1	0	0
Firmicutes	Clostridia	Clostridiales	Ruminococcaceae	Ruminococcaceae_UCG-010	uncultured_bacterium_g__Ruminococcaceae_UCG-010	OTU776	0	0	0	0
Firmicutes	Clostridia	Clostridiales	Christensenellaceae	norank_f__Christensenellaceae	uncultured_bacterium_g__norank_f__Christensenellaceae	OTU139	0	2	0	1
Firmicutes	Clostridia	Clostridiales	Christensenellaceae	Christensenellaceae_R-7_group	uncultured_bacterium_g__Christensenellaceae_R-7_group	OTU207	0	0	0	0
Actinobacteria	Actinobacteria	Coriobacteriales	Coriobacteriaceae	unclassified_f__Coriobacteriaceae	unclassified_f__Coriobacteriaceae	OTU477	0	0	0	0
Firmicutes	Clostridia	Clostridiales	Ruminococcaceae	unclassified_f__Ruminococcaceae	unclassified_f__Ruminococcaceae	OTU176	0	2	0	0
Bacteroidetes	Bacteroidia	Bacteroidales	Bacteroidales_S24-7_g	norank_f__Bacteroidales_S24-7_gro	uncultured_bacterium_g__norank_f__Bacteroidales_S24-7_group	OTU262	0	0	12	13
Firmicutes	Clostridia	Clostridiales	Ruminococcaceae	Anaerotruncus	unclassified_g__Anaerotruncus	OTU444	3	0	0	0
Firmicutes	Clostridia	Clostridiales	Ruminococcaceae	Ruminococcaceae_UCG-013	uncultured_Clostridiales_bacterium_g__Ruminococcaceae_UCG-013	OTU522	0	0	0	0
Firmicutes	Clostridia	Clostridiales	Ruminococcaceae	Ruminococcaceae_UCG-010	uncultured_organism_g__Ruminococcaceae_UCG-010	OTU782	0	0	0	0
Saccharibacteria	Unknown_Class_p__Sacc	Unknown_Order_c_	Unknown_Family_o__	Candidatus_Saccharimonas	uncultured_bacterium_g__Candidatus_Saccharimonas	OTU261	0	0	17	10
Firmicutes	Clostridia	Clostridiales	Lachnospiraceae	unclassified_f__Lachnospiraceae	unclassified_f__Lachnospiraceae	OTU120	0	0	1	0
Chloroflexi	KD4-96	norank_c__KD4-96	norank_c__KD4-96	norank_c__KD4-96	unclassified_g__norank_c__KD4-96	OTU254	0	0	6	17
Firmicutes	Clostridia	Clostridiales	Clostridiales_vadinBB6	norank_f__Clostridiales_vadinBB60_	unclassified_g__norank_f__Clostridiales_vadinBB60_group	OTU209	0	0	0	0
Tenericutes	Mollicutes	Mollicutes_RF9	norank_o__Mollicutes	norank_o__Mollicutes_RF9	unclassified_g__norank_o__Mollicutes_RF9	OTU572	0	0	0	0
Firmicutes	Clostridia	Clostridiales	Ruminococcaceae	unclassified_f__Ruminococcaceae	unclassified_f__Ruminococcaceae	OTU195	0	0	0	0
Proteobacteria	Alphaproteobacteria	Sphingomonadales	Sphingomonadaceae	Sphingomonas	uncultured_Sphingomonadaceae_bacterium_g__Sphingomonas	OTU375	0	0	10	14
Tenericutes	Mollicutes	Mollicutes_RF9	norank_o__Mollicutes	norank_o__Mollicutes_RF9	unclassified_g__norank_o__Mollicutes_RF9	OTU727	0	1	0	1
Firmicutes	Erysipelotrichia	Erysipelotrichales	Erysipelotrichaceae	Turicibacter	uncultured_bacterium_g__Turicibacter	OTU40	0	0	6	5
Firmicutes	Clostridia	Clostridiales	Lachnospiraceae	Coprococcus_1	intestinal_bacterium_CG19-1	OTU45	0	0	0	0
Firmicutes	Clostridia	Clostridiales	Peptococcaceae	norank_f__Peptococcaceae	unclassified_g__norank_f__Peptococcaceae	OTU116	0	0	0	0
Firmicutes	Clostridia	Clostridiales	Peptococcaceae	norank_f__Peptococcaceae	unclassified_g__norank_f__Peptococcaceae	OTU199	0	0	0	2
Bacteroidetes	Bacteroidia	Bacteroidales	Bacteroidaceae	Bacteroides	Bacteroides_acidifaciens	OTU320	0	0	12	10
Firmicutes	Clostridia	Clostridiales	Ruminococcaceae	unclassified_f__Ruminococcaceae	unclassified_f__Ruminococcaceae	OTU552	1	0	0	1
Firmicutes	Clostridia	Clostridiales	Lachnospiraceae	Butyrivibrio	uncultured_organism_g__Butyrivibrio	OTU762	0	0	0	0
Tenericutes	Mollicutes	Mollicutes_RF9	norank_o__Mollicutes	norank_o__Mollicutes_RF9	uncultured_bacterium_g__norank_o__Mollicutes_RF9	OTU797	0	0	0	0
Firmicutes	Clostridia	Clostridiales	Lachnospiraceae	unclassified_f__Lachnospiraceae	unclassified_f__Lachnospiraceae	OTU852	0	2	0	5
Proteobacteria	Alphaproteobacteria	Rhizobiales	Bradyrhizobiaceae	Bradyrhizobium	Bradyrhizobium_elkanii	OTU185	0	0	7	11
Chloroflexi	Chloroflexia	Chloroflexales	Roseiflexaceae	Roseiflexus	uncultured_bacterium_g__Roseiflexus	OTU415	0	0	6	16
Proteobacteria	Alphaproteobacteria	Rhizobiales	Xanthobacteraceae	norank_f__Xanthobacteraceae	uncultured_bacterium_g__norank_f__Xanthobacteraceae	OTU427	0	0	10	12
Firmicutes	unclassified_p__Firmicut	unclassified_p__Fir	unclassified_p__Firmi	unclassified_p__Firmicutes	unclassified_p__Firmicutes	OTU454	0	0	0	0
Firmicutes	Clostridia	Clostridiales	Ruminococcaceae	Anaerotruncus	unclassified_g__Anaerotruncus	OTU621	0	0	2	1
Firmicutes	Clostridia	Clostridiales	Ruminococcaceae	Anaerotruncus	uncultured_Clostridiales_bacterium_g__Anaerotruncus	OTU736	0	0	2	0
Firmicutes	Clostridia	Clostridiales	Ruminococcaceae	Hydrogenoanaerobacterium	Selenomonadales_bacterium_Marseille-P2399	OTU5	0	0	0	0
Bacteroidetes	Bacteroidia	Bacteroidales	Bacteroidales_S24-7_g	norank_f__Bacteroidales_S24-7_gro	uncultured_bacterium_g__norank_f__Bacteroidales_S24-7_group	OTU300	0	0	9	11
Bacteroidetes	Bacteroidia	Bacteroidales	Prevotellaceae	Prevotellaceae_NK3B31_group	uncultured_bacterium_g__Prevotellaceae_NK3B31_group	OTU331	0	0	10	8
Proteobacteria	Betaproteobacteria	Burkholderiales	Burkholderiaceae	Ralstonia	unidentified_marine_bacterioplankton_g__Ralstonia	OTU593	0	1	2	0
Tenericutes	Mollicutes	Mollicutes_RF9	norank_o__Mollicutes	norank_o__Mollicutes_RF9	uncultured_Erysipelotrichia_bacterium_g__norank	OTU594	0	0	0	0
Firmicutes	Clostridia	Clostridiales	Ruminococcaceae	Anaerotruncus	unclassified_g__Anaerotruncus	OTU713	0	0	2	0
Firmicutes	Clostridia	Clostridiales	Clostridiales_vadinBB6	norank_f__Clostridiales_vadinBB60_	uncultured_bacterium_g__norank_f__Clostridiales_vadinBB60_group	OTU724	0	0	0	0
Firmicutes	Clostridia	Clostridiales	Christensenellaceae	unclassified_f__Christensenellaceae	unclassified_f__Christensenellaceae	OTU793	0	0	0	0
Firmicutes	Clostridia	Clostridiales	Lachnospiraceae	Tyzzerella	unclassified_g__Tyzzerella	OTU796	0	1	0	0
Actinobacteria	Actinobacteria	Bifidobacteriales	Bifidobacteriaceae	Bifidobacterium	Bifidobacterium_pseudocatenulatum_DSM_20438__JCM_1200__LMG_10505	OTU55	0	0	0	0
Firmicutes	Clostridia	Clostridiales	Clostridiales_vadinBB6	norank_f__Clostridiales_vadinBB60_	uncultured_bacterium_g__norank_f__Clostridiales_vadinBB60_group	OTU336	0	0	0	0
Bacteroidetes	Bacteroidia	Bacteroidales	Bacteroidaceae	Bacteroides	Bacteroides_sartorii	OTU345	0	0	7	9
Firmicutes	Clostridia	Clostridiales	Ruminococcaceae	Ruminiclostridium_5	unclassified_g__Ruminiclostridium_5	OTU818	0	1	0	0
Firmicutes	Clostridia	Clostridiales	Christensenellaceae	norank_f__Christensenellaceae	uncultured_bacterium_g__norank_f__Christensenellaceae	OTU144	0	0	0	0
Firmicutes	Clostridia	Clostridiales	Ruminococcaceae	[Eubacterium]_coprostanoligenes_g	unclassified_g__[Eubacterium]_coprostanoligenes_group	OTU212	0	0	1	2
Firmicutes	Clostridia	Clostridiales	Ruminococcaceae	Ruminococcaceae_UCG-013	unclassified_g__Ruminococcaceae_UCG-013	OTU342	0	0	0	0
Chloroflexi	Chloroflexia	Chloroflexales	Roseiflexaceae	Roseiflexus	uncultured_bacterium_g__Roseiflexus	OTU433	0	0	3	14
Firmicutes	Clostridia	Clostridiales	Ruminococcaceae	unclassified_f__Ruminococcaceae	unclassified_f__Ruminococcaceae	OTU646	0	0	0	0
Firmicutes	Bacilli	Lactobacillales	Lactobacillaceae	Lactobacillus	Lactobacillus_saerimneri	OTU719	0	0	0	0
Firmicutes	Clostridia	Clostridiales	Ruminococcaceae	Ruminococcus_1	uncultured_organism_g__Ruminococcus_1	OTU227	0	0	0	0
Deferribacteres	Deferribacteres	Deferribacterales	Deferribacteraceae	Mucispirillum	uncultured_bacterium_g__Mucispirillum	OTU272	0	0	7	10
Chloroflexi	Chloroflexia	Chloroflexales	Roseiflexaceae	Roseiflexus	uncultured_bacterium_g__Roseiflexus	OTU277	0	0	5	12
Actinobacteria	Actinobacteria	Solirubrobacterales	Solirubrobacteraceae	Solirubrobacter	uncultured_bacterium_g__Solirubrobacter	OTU414	0	0	5	11
Firmicutes	Clostridia	Clostridiales	Ruminococcaceae	Anaerotruncus	unclassified_g__Anaerotruncus	OTU546	0	0	3	0
Tenericutes	Mollicutes	Mollicutes_RF9	norank_o__Mollicutes	norank_o__Mollicutes_RF9	uncultured_bacterium_g__norank_o__Mollicutes_RF9	OTU571	0	0	0	0
Firmicutes	Clostridia	Clostridiales	Ruminococcaceae	Ruminiclostridium_5	uncultured_bacterium_g__Ruminiclostridium_5	OTU806	0	0	0	0
Firmicutes	Clostridia	Clostridiales	Ruminococcaceae	Ruminococcaceae_UCG-013	unclassified_g__Ruminococcaceae_UCG-013	OTU108	0	2	0	0
Firmicutes	Clostridia	Clostridiales	Peptococcaceae	norank_f__Peptococcaceae	uncultured_bacterium_g__norank_f__Peptococcaceae	OTU112	0	0	0	2
Acidobacteria	Acidobacteria	Blastocatellales	Blastocatellaceae__Su	RB41	unclassified_g__RB41	OTU374	0	0	4	12
Actinobacteria	Actinobacteria	Propionibacteriales	Nocardioidaceae	Marmoricola	uncultured_bacterium_g__Marmoricola	OTU395	0	0	4	12
Firmicutes	Clostridia	Clostridiales	Ruminococcaceae	unclassified_f__Ruminococcaceae	unclassified_f__Ruminococcaceae	OTU650	0	0	0	0
Firmicutes	Clostridia	Clostridiales	Ruminococcaceae	Ruminiclostridium_9	unclassified_g__Ruminiclostridium_9	OTU92	0	0	0	0
Actinobacteria	Actinobacteria	Streptomycetales	Streptomycetaceae	Streptomyces	unclassified_g__Streptomyces	OTU358	0	0	8	7
Proteobacteria	Deltaproteobacteria	Desulfovibrionales	Desulfovibrionaceae	Desulfovibrio	unclassified_g__Desulfovibrio	OTU431	0	0	6	8
Firmicutes	Clostridia	Clostridiales	Lachnospiraceae	unclassified_f__Lachnospiraceae	unclassified_f__Lachnospiraceae	OTU668	0	1	0	0
Firmicutes	Clostridia	Clostridiales	Syntrophomonadacea	Syntrophomonas	unclassified_g__Syntrophomonas	OTU790	0	0	0	0
Bacteroidetes	Bacteroidia	Bacteroidales	Bacteroidales_S24-7_g	norank_f__Bacteroidales_S24-7_gro	uncultured_bacterium_g__norank_f__Bacteroidales_S24-7_group	OTU369	0	0	3	10
Firmicutes	Clostridia	Clostridiales	Peptostreptococcacea	Peptoclostridium	Peptoclostridium_difficile_g__Peptoclostridium	OTU459	0	0	4	2
Firmicutes	Erysipelotrichia	Erysipelotrichales	Erysipelotrichaceae	[Clostridium]_innocuum_group	uncultured_bacterium_g__[Clostridium]_innocuum_group	OTU503	2	0	0	0
Firmicutes	Clostridia	Clostridiales	Ruminococcaceae	Ruminococcaceae_UCG-009	uncultured_bacterium_g__Ruminococcaceae_UCG-009	OTU686	0	0	1	1
Firmicutes	Clostridia	Clostridiales	Ruminococcaceae	Anaerotruncus	unclassified_g__Anaerotruncus	OTU753	0	0	0	0
Firmicutes	Clostridia	Clostridiales	Ruminococcaceae	Ruminiclostridium	uncultured_organism_g__Ruminiclostridium	OTU829	0	0	0	0
Bacteroidetes	Bacteroidia	Bacteroidales	Bacteroidales_S24-7_g	norank_f__Bacteroidales_S24-7_gro	uncultured_bacterium_g__norank_f__Bacteroidales_S24-7_group	OTU304	0	0	6	5
Bacteroidetes	Bacteroidia	Bacteroidales	Bacteroidales_S24-7_g	norank_f__Bacteroidales_S24-7_gro	uncultured_bacterium_g__norank_f__Bacteroidales_S24-7_group	OTU350	0	0	2	11
Bacteroidetes	Bacteroidia	Bacteroidales	Bacteroidales_S24-7_g	norank_f__Bacteroidales_S24-7_gro	uncultured_bacterium_g__norank_f__Bacteroidales_S24-7_group	OTU380	0	0	7	4

Firmicutes	Clostridia	Clostridiales	Ruminococcaceae	Candidatus_Soleaferrea	Ruminococcaceae_bacterium_GD6	OTU456	0	0	0	0
Firmicutes	Clostridia	Clostridiales	Ruminococcaceae	unclassified_f__Ruminococcaceae	unclassified_f__Ruminococcaceae	OTU545	0	0	0	0
Firmicutes	Clostridia	Clostridiales	Ruminococcaceae	Ruminococcaceae_UCG-009	uncultured_bacterium_g__Ruminococcaceae_UCG-009	OTU595	0	0	0	0
Firmicutes	Clostridia	Clostridiales	Ruminococcaceae	unclassified_f__Ruminococcaceae	unclassified_f__Ruminococcaceae	OTU844	1	0	0	0
Firmicutes	Clostridia	Clostridiales	Christensenellaceae	norank_f__Christensenellaceae	uncultured_bacterium_g__norank_f__Christensenellaceae	OTU126	0	0	2	0
Firmicutes	Clostridia	Clostridiales	Clostridiales_vadinBB6	norank_f__Clostridiales_vadinBB60_	uncultured_bacterium_g__norank_f__Clostridiales_vadinBB60_group	OTU161	0	0	0	0
Bacteroidetes	Bacteroidia	Bacteroidales	Bacteroidales_S24-7_g	norank_f__Bacteroidales_S24-7_gro	unclassified_g__norank_f__Bacteroidales_S24-7_group	OTU267	0	0	6	1
Actinobacteria	Actinobacteria	Streptosporangiales	Thermomonosporacea	Actinomadura	Actinomadura_hallensis	OTU291	0	0	11	1
Firmicutes	Clostridia	Clostridiales	Ruminococcaceae	Ruminiclostridium_5	unclassified_g__Ruminiclostridium_5	OTU341	0	0	0	0
Actinobacteria	Actinobacteria	Corynebacteriales	Mycobacteriaceae	Mycobacterium	unclassified_g__Mycobacterium	OTU381	0	0	3	9
Firmicutes	Clostridia	Clostridiales	Ruminococcaceae	Ruminococcus_1	unclassified_g__Ruminococcus_1	OTU523	0	0	0	0
Bacteroidetes	Bacteroidia	Bacteroidales	Rikenellaceae	Alistipes	Alistipes_sp._CHKCI003	OTU15	0	1	0	0
Firmicutes	Clostridia	Clostridiales	Ruminococcaceae	Ruminococcaceae_UCG-010	unclassified_g__Ruminococcaceae_UCG-010	OTU222	0	0	0	0
Firmicutes	Clostridia	Clostridiales	Clostridiales_vadinBB6	norank_f__Clostridiales_vadinBB60_	uncultured_prokaryote_g__norank_f__Clostridiales_vadinBB60_group	OTU247	0	0	0	0
Bacteroidetes	Bacteroidia	Bacteroidales	Bacteroidales_S24-7_g	norank_f__Bacteroidales_S24-7_gro	uncultured_bacterium_g__norank_f__Bacteroidales_S24-7_group	OTU253	0	0	6	3
Verrucomicrobia	Spartobacteria	Chthoniobacterales	DA101_soil_group	norank_f__DA101_soil_group	uncultured_Prosthecobacter_sp._g__norank_f__DA101_soil_group	OTU279	0	0	4	6
Cyanobacteria	Cyanobacteria	Gastranaerophilales	norank_o__Gastranae	norank_o__Gastranaerophilales	unclassified_g__norank_o__Gastranaerophilales	OTU328	0	0	0	0
Proteobacteria	Epsilonproteobacteria	Campylobacterales	Helicobacteraceae	Helicobacter	Helicobacter_ganmani	OTU446	0	1	3	6
Firmicutes	Clostridia	Clostridiales	Ruminococcaceae	Ruminococcaceae_UCG-007	unclassified_g__Ruminococcaceae_UCG-007	OTU789	0	0	0	0
Proteobacteria	Alphaproteobacteria	Rhizobiales	Xanthobacteraceae	Variibacter	uncultured_forest_soil_bacterium_g__Variibacter	OTU840	0	0	3	6
Firmicutes	Clostridia	Clostridiales	Ruminococcaceae	Ruminiclostridium_5	uncultured_organism_g__Ruminiclostridium_5	OTU24	0	0	0	0
Gemmatimonade	Gemmatimonadetes	Gemmatimonadales	Gemmatimonadaceae	Gemmatimonas	uncultured_soil_bacterium_g__Gemmatimonas	OTU354	0	0	0	10
Actinobacteria	Actinobacteria	Propionibacteriales	Nocardioidaceae	Kribbella	uncultured_bacterium_g__Kribbella	OTU391	0	0	2	8
Actinobacteria	Actinobacteria	Pseudonocardiales	Pseudonocardiaceae	Pseudonocardia	uncultured_bacterium_g__Pseudonocardia	OTU392	0	0	2	8
Firmicutes	Clostridia	Clostridiales	Lachnospiraceae	unclassified_f__Lachnospiraceae	unclassified_f__Lachnospiraceae	OTU462	0	0	0	0
Firmicutes	Clostridia	Clostridiales	Ruminococcaceae	Anaerotruncus	unclassified_g__Anaerotruncus	OTU486	0	1	0	1
Actinobacteria	Actinobacteria	Coriobacteriales	Coriobacteriaceae	Gordonibacter	unclassified_g__Gordonibacter	OTU555	0	0	0	0
Firmicutes	Clostridia	Clostridiales	Ruminococcaceae	Anaerotruncus	unclassified_g__Anaerotruncus	OTU687	0	0	0	0
unclassified_k__n	unclassified_k__norank	unclassified_k__no	unclassified_k__noran	unclassified_k__norank	unclassified_k__norank	OTU706	0	0	0	0
Proteobacteria	Alphaproteobacteria	Rhizobiales	Xanthobacteraceae	Variibacter	uncultured_forest_soil_bacterium_g__Variibacter	OTU252	0	0	4	5
Actinobacteria	Actinobacteria	Micromonosporales	Micromonosporaceae	Longispora	uncultured_bacterium_g__Longispora	OTU357	0	0	6	3
Actinobacteria	Actinobacteria	Frankiales	Acidothermaceae	Acidothermus	Thermomonosporaceae_bacterium_YE4-D4-16-CH2	OTU403	0	0	2	7
Tenericutes	Mollicutes	Mollicutes_RF9	norank_o__Mollicutes	norank_o__Mollicutes_RF9	unclassified_g__norank_o__Mollicutes_RF9	OTU547	0	0	0	0
Bacteroidetes	Bacteroidia	Bacteroidales	Bacteroidaceae	Bacteroides	unclassified_g__Bacteroides	OTU554	0	0	0	2
Firmicutes	Clostridia	Clostridiales	Ruminococcaceae	unclassified_f__Ruminococcaceae	unclassified_f__Ruminococcaceae	OTU708	0	1	0	0
Firmicutes	Clostridia	Clostridiales	Christensenellaceae	unclassified_f__Christensenellaceae	unclassified_f__Christensenellaceae	OTU205	0	0	0	0
Firmicutes	Clostridia	Clostridiales	Ruminococcaceae	Ruminococcaceae_UCG-010	uncultured_bacterium_g__Ruminococcaceae_UCG-010	OTU240	0	0	0	0
Bacteroidetes	Bacteroidia	Bacteroidales	Rikenellaceae	Rikenella	uncultured_bacterium_g__Rikenella	OTU244	0	0	6	0
Bacteroidetes	Bacteroidia	Bacteroidales	Bacteroidales_S24-7_g	norank_f__Bacteroidales_S24-7_gro	uncultured_bacterium_g__norank_f__Bacteroidales_S24-7_group	OTU259	0	0	4	3
Bacteroidetes	Bacteroidia	Bacteroidales	Bacteroidales_S24-7_g	norank_f__Bacteroidales_S24-7_gro	unclassified_g__norank_f__Bacteroidales_S24-7_group	OTU280	0	0	3	4
Actinobacteria	Actinobacteria	Micromonosporales	Micromonosporaceae	unclassified_f__Micromonosporacea	unclassified_f__Micromonosporaceae	OTU286	0	0	1	6
Actinobacteria	Actinobacteria	Gaiellales	Gaiellaceae	Gaiella	uncultured_bacterium_g__Gaiella	OTU309	0	0	5	3
Bacteroidetes	Bacteroidia	Bacteroidales	Bacteroidales_S24-7_g	norank_f__Bacteroidales_S24-7_gro	uncultured_Bacteroidales_bacterium_g__norank_f__Bacteroidales_S24-7_group	OTU348	0	0	2	3
Actinobacteria	Actinobacteria	Propionibacteriales	Nocardioidaceae	Nocardioides	uncultured_bacterium_g__Nocardioides	OTU367	0	0	2	6
Actinobacteria	Actinobacteria	norank_c__Actinob	norank_c__Actinobact	norank_c__Actinobacteria	uncultured_bacterium_g__norank_c__Actinobacteria	OTU396	0	0	3	5
Firmicutes	Clostridia	Clostridiales	Ruminococcaceae	unclassified_f__Ruminococcaceae	unclassified_f__Ruminococcaceae	OTU617	0	0	0	0
Proteobacteria	Alphaproteobacteria	Rhizobiales	Methylobacteriaceae	Meganema	Meganema_perideroedes	OTU30	0	0	0	0
Firmicutes	Clostridia	Clostridiales	Ruminococcaceae	unclassified_f__Ruminococcaceae	unclassified_f__Ruminococcaceae	OTU239	0	0	0	0
Proteobacteria	Betaproteobacteria	Burkholderiales	Alcaligenaceae	Parasutterella	Sutterellaceae_bacterium_YL45	OTU281	0	0	6	1
Bacteroidetes	Bacteroidia	Bacteroidales	Bacteroidales_S24-7_g	norank_f__Bacteroidales_S24-7_gro	unclassified_g__norank_f__Bacteroidales_S24-7_group	OTU303	0	0	1	4
Bacteroidetes	Bacteroidia	Bacteroidales	Bacteroidales_S24-7_g	norank_f__Bacteroidales_S24-7_gro	uncultured_Bacteroidales_bacterium_g__norank_f__Bacteroidales_S24-7_group	OTU307	0	0	6	1
Chloroflexi	Anaerolineae	Anaerolineales	Anaerolineaceae	norank_f__Anaerolineaceae	wastewater_metagenome_g__norank_f__Anaerolineaceae	OTU352	0	0	4	3
Bacteroidetes	Bacteroidia	Bacteroidales	Bacteroidales_S24-7_g	norank_f__Bacteroidales_S24-7_gro	uncultured_bacterium_g__norank_f__Bacteroidales_S24-7_group	OTU389	0	0	3	4
Chloroflexi	Chloroflexia	Chloroflexales	Roseiflexaceae	Roseiflexus	uncultured_bacterium_g__Roseiflexus	OTU404	0	0	2	5
Firmicutes	Clostridia	Clostridiales	Lachnospiraceae	Lachnoclostridium	unclassified_g__Lachnoclostridium	OTU416	0	0	2	3
Chloroflexi	Chloroflexia	Chloroflexales	Roseiflexaceae	Roseiflexus	uncultured_bacterium_g__Roseiflexus	OTU425	0	0	2	5
Actinobacteria	Actinobacteria	Corynebacteriales	Nocardiaceae	Rhodococcus	Rhodococcus_erythropolis	OTU662	0	0	0	5
Proteobacteria	Gammaproteobacteria	Pseudomonadales	Moraxellaceae	Acinetobacter	unclassified_g__Acinetobacter	OTU685	0	0	0	0
Firmicutes	Clostridia	Clostridiales	Clostridiaceae_1	Clostridium_sensu_stricto_1	unclassified_g__Clostridium_sensu_stricto_1	OTU817	0	0	1	0
Firmicutes	Clostridia	Clostridiales	Christensenellaceae	Christensenellaceae_R-7_group	uncultured_Clostridiaceae_bacterium_g__Christensenellaceae_R-7_group	OTU47	0	0	0	0
Firmicutes	Clostridia	Clostridiales	Ruminococcaceae	Anaerotruncus	unclassified_g__Anaerotruncus	OTU96	0	0	0	1
Firmicutes	Clostridia	Clostridiales	Clostridiales_vadinBB6	norank_f__Clostridiales_vadinBB60_	uncultured_prokaryote_g__norank_f__Clostridiales_vadinBB60_group	OTU147	0	0	0	0
Firmicutes	Clostridia	Clostridiales	Ruminococcaceae	Candidatus_Soleaferrea	Ruminococcaceae_bacterium_Marseille-P2935	OTU183	0	0	0	0
Tenericutes	Mollicutes	Mollicutes_RF9	norank_o__Mollicutes	norank_o__Mollicutes_RF9	uncultured_bacterium_g__norank_o__Mollicutes_RF9	OTU208	0	0	0	0
Firmicutes	unclassified_p__Firmicut	unclassified_p__Fir	unclassified_p__Firmi	unclassified_p__Firmicutes	unclassified_p__Firmicutes	OTU246	0	0	0	0
Bacteroidetes	Bacteroidia	Bacteroidales	Bacteroidales_S24-7_g	norank_f__Bacteroidales_S24-7_gro	uncultured_bacterium_g__norank_f__Bacteroidales_S24-7_group	OTU266	0	0	4	2
Bacteroidetes	Bacteroidia	Bacteroidales	Rikenellaceae	Alistipes	uncultured_bacterium_g__Alistipes	OTU282	0	0	5	1
Actinobacteria	Actinobacteria	Solirubrobacterales	Solirubrobacteraceae	Solirubrobacter	uncultured_bacterium_g__Solirubrobacter	OTU288	0	0	3	3
Bacteroidetes	Bacteroidia	Bacteroidales	Bacteroidales_S24-7_g	norank_f__Bacteroidales_S24-7_gro	uncultured_bacterium_g__norank_f__Bacteroidales_S24-7_group	OTU289	0	0	4	2
Bacteroidetes	Bacteroidia	Bacteroidales	Bacteroidaceae	Bacteroides	unclassified_g__Bacteroides	OTU290	0	0	4	2
Chloroflexi	Chloroflexia	Chloroflexales	Roseiflexaceae	Roseiflexus	uncultured_bacterium_g__Roseiflexus	OTU318	0	0	2	4
Acidobacteria	Acidobacteria	norank_c__Acidoba	norank_c__Acidobacte	norank_c__Acidobacteria	uncultured_Acidobacteriales_bacterium_g__norank_c__Acidobacteria	OTU366	0	0	2	4
Proteobacteria	Epsilonproteobacteria	Campylobacterales	Campylobacteraceae	Arcobacter	unclassified_g__Arcobacter	OTU376	0	0	2	3
Gemmatimonade	Gemmatimonadetes	Gemmatimonadales	Gemmatimonadaceae	Gemmatimonas	unclassified_g__Gemmatimonas	OTU377	0	0	1	5
Proteobacteria	Alphaproteobacteria	Rhizobiales	Methylocystaceae	unclassified_f__Methylocystaceae	unclassified_f__Methylocystaceae	OTU379	0	0	0	4
Proteobacteria	Alphaproteobacteria	Rhodobacterales	Rhodobacteraceae	Pannonibacter	unclassified_g__Pannonibacter	OTU421	0	0	1	4
Proteobacteria	Epsilonproteobacteria	Campylobacterales	Helicobacteraceae	Helicobacter	Helicobacter_hepaticus	OTU422	0	0	2	4
Tenericutes	Mollicutes	Mollicutes_RF9	norank_o__Mollicutes	norank_o__Mollicutes_RF9	unclassified_g__norank_o__Mollicutes_RF9	OTU491	0	0	0	0
Firmicutes	Clostridia	Clostridiales	Ruminococcaceae	unclassified_f__Ruminococcaceae	unclassified_f__Ruminococcaceae	OTU696	0	0	0	0
Firmicutes	Clostridia	Clostridiales	Christensenellaceae	norank_f__Christensenellaceae	uncultured_bacterium_g__norank_f__Christensenellaceae	OTU819	0	0	0	0
Firmicutes	Clostridia	Clostridiales	Ruminococcaceae	unclassified_f__Ruminococcaceae	unclassified_f__Ruminococcaceae	OTU186	0	0	0	0
Firmicutes	Clostridia	Clostridiales	Ruminococcaceae	Ruminiclostridium	uncultured_bacterium_g__Ruminiclostridium	OTU269	0	0	4	0
Bacteroidetes	Bacteroidia	Bacteroidales	Porphyromonadaceae	Parabacteroides	uncultured_organism_g__Parabacteroides	OTU285	0	0	2	3
Bacteroidetes	Bacteroidia	Bacteroidales	Rikenellaceae	Alistipes	uncultured_bacterium_g__Alistipes	OTU299	0	0	3	2
Actinobacteria	Actinobacteria	Acidimicrobiales	norank_o__Acidimicro	norank_o__Acidimicrobiales	uncultured_Acidimicrobiales_bacterium_g__norank	OTU305	0	0	3	2
Chloroflexi	S085	norank_c__S085	norank_c__S085	norank_c__S085	uncultured_Chloroflexi_bacterium_g__norank_c__S085	OTU362	0	0	2	3
Proteobacteria	Alphaproteobacteria	Rhodospirillales	Acetobacteraceae	norank_f__Acetobacteraceae	uncultured_bacterium_g__norank_f__Acetobacteraceae	OTU371	0	0	2	3
Chloroflexi	TK10	norank_c__TK10	norank_c__TK10	norank_c__TK10	unclassified_g__norank_c__TK10	OTU382	0	0	0	5
Actinobacteria	Actinobacteria	Micrococcales	Brevibacteriaceae	Brevibacterium	unclassified_g__Brevibacterium	OTU399	0	0	0	5
Bacteroidetes	Bacteroidia	Bacteroidales	Bacteroidales_S24-7_g	norank_f__Bacteroidales_S24-7_gro	uncultured_organism_g__norank_f__Bacteroidales_S24-7_group	OTU401	0	0	0	4
Firmicutes	Erysipelotrichia	Erysipelotrichales	Erysipelotrichaceae	Erysipelatoclostridium	uncultured_bacterium_g__Erysipelatoclostridium	OTU407	0	0	2	3
Proteobacteria	Betaproteobacteria	Nitrosomonadales	Nitrosomonadaceae	norank_f__Nitrosomonadaceae	unclassified_g__norank_f__Nitrosomonadaceae	OTU420	0	0	1	3
Firmicutes	Clostridia	Clostridiales	Ruminococcaceae	Ruminococcaceae_UCG-014	uncultured_Firmicutes_bacterium_g__Ruminococcaceae_UCG-014	OTU798	0	0	0	0
Firmicutes	Clostridia	Clostridiales	Ruminococcaceae	Ruminococcaceae_UCG-010	unclassified_g__Ruminococcaceae_UCG-010	OTU800	0	0	0	0
Tenericutes	Mollicutes	Mycoplasmatales	Mycoplasmataceae	norank_f__Mycoplasmataceae	unclassified_g__norank_f__Mycoplasmataceae	OTU825	0	0	1	3
Firmicutes	Clostridia	Clostridiales	Christensenellaceae	Christensenellaceae_R-7_group	unclassified_g__Christensenellaceae_R-7_group	OTU833	0	0	0	0
Bacteroidetes	Bacteroidia	Bacteroidales	Bacteroidaceae	Bacteroides	Bacteroides_caccae	OTU46	0	0	0	0
Bacteroidetes	Flavobacteriia	Flavobacteriales	Flavobacteriaceae	Cloacibacterium	uncultured_bacterium_g__Cloacibacterium	OTU49	0	0	0	0
Firmicutes	Clostridia	Clostridiales	Ruminococcaceae	Ruminococcaceae_UCG-010	uncultured_bacterium_g__Ruminococcaceae_UCG-010	OTU97	0	0	0	0
Proteobacteria	Gammaproteobacteria	Pseudomonadales	Moraxellaceae	Acinetobacter	Acinetobacter_guillouiae	OTU138	0	0	0	0
Bacteroidetes	Bacteroidia	Bacteroidales	Bacteroidales_S24-7_g	norank_f__Bacteroidales_S24-7_gro	uncultured_bacterium_g__norank_f__Bacteroidales_S24-7_group	OTU211	0	0	1	2
Actinobacteria	Actinobacteria	Acidimicrobiales	Acidimicrobiaceae	CL500-29_marine_group	uncultured_bacterium_g__CL500-29_marine_group	OTU260	0	0	3	0
Acidobacteria	Acidobacteria	Acidobacteriales	Acidobacteriaceae__S	norank_f__Acidobacteriaceae__Subg	uncultured_bacterium_g__norank_f__Acidobacteriaceae__Subgroup_1_	OTU263	0	0	4	0
Proteobacteria	Alphaproteobacteria	Rhodospirillales	Rhodospirillaceae	norank_f__Rhodospirillaceae	unclassified_g__norank_f__Rhodospirillaceae	OTU273	0	0	4	0
Actinobacteria	Actinobacteria	Propionibacteriales	Nocardioidaceae	unclassified_f__Nocardioidaceae	unclassified_f__Nocardioidaceae	OTU298	0	0	1	3
Bacteroidetes	Bacteroidia	Bacteroidales	Bacteroidaceae	Bacteroides	Bacteroides_stercoris_ATCC_43183	OTU317	0	0	4	0
Chloroflexi	Chloroflexia	Chloroflexales	Roseiflexaceae	Roseiflexus	uncultured_bacterium_g__Roseiflexus	OTU372	0	0	0	4
Chloroflexi	Anaerolineae	Anaerolineales	Anaerolineaceae	norank_f__Anaerolineaceae	Chloroflexi_bacterium_OLB13	OTU383	0	0	0	2
Actinobacteria	Actinobacteria	Propionibacteriales	Nocardioidaceae	Nocardioides	unclassified_g__Nocardioides	OTU386	0	0	2	2
Chloroflexi	Thermomicrobia	JG30-KF-CM45	norank_o__JG30-KF-C	norank_o__JG30-KF-CM45	unclassified_g__norank_o__JG30-KF-CM45	OTU408	0	0	0	4
Chloroflexi	Gitt-GS-136	norank_c__Gitt-GS-	norank_c__Gitt-GS-13	norank_c__Gitt-GS-136	uncultured_bacterium_g__norank_c__Gitt-GS-136	OTU413	0	0	2	2

Actinobacteria	Actinobacteria	Acidimicrobiales	Iamiaceae	Iamia	uncultured_bacterium_g__Iamia	OTU417	0	0	1	3
Actinobacteria	Actinobacteria	Frankiales	Geodermatophilaceae	Blastococcus	uncultured_Blastococcus_sp._g__Blastococcus	OTU419	0	0	0	4
Actinobacteria	Actinobacteria	Propionibacteriales	Nocardioidaceae	Nocardioides	uncultured_organism_g__Nocardioides	OTU430	0	0	1	3
Gemmatimonade	Gemmatimonadetes	Gemmatimonadales	Gemmatimonadaceae	norank_f__Gemmatimonadaceae	uncultured_Gemmatimonadetes_bacterium_g__norank_f__Gemmatimonadaceae	OTU442	0	0	2	2
Firmicutes	Clostridia	Clostridiales	Ruminococcaceae	Anaerotruncus	uncultured_bacterium_g__Anaerotruncus	OTU592	0	0	0	0
Firmicutes	Clostridia	Clostridiales	Lachnospiraceae	Coprococcus_1	unclassified_g__Coprococcus_1	OTU734	0	0	1	0
Firmicutes	Clostridia	Clostridiales	Ruminococcaceae	Candidatus_Soleaferrea	Candidatus_Soleaferrea_massiliensis	OTU37	0	0	0	0
Cyanobacteria	Cyanobacteria	norank_c__Cyanoba	norank_c__Cyanobact	norank_c__Cyanobacteria	unclassified_g__norank_c__Cyanobacteria	OTU149	0	0	0	0
Firmicutes	Clostridia	Clostridiales	Ruminococcaceae	Ruminococcaceae_UCG-010	unclassified_g__Ruminococcaceae_UCG-010	OTU157	0	0	0	0
Bacteroidetes	Bacteroidia	Bacteroidales	Bacteroidales_S24-7_g	norank_f__Bacteroidales_S24-7_gro	unclassified_g__norank_f__Bacteroidales_S24-7_group	OTU190	0	0	1	0
Firmicutes	Clostridia	Clostridiales	Ruminococcaceae	Ruminococcaceae_UCG-010	uncultured_bacterium_g__Ruminococcaceae_UCG-010	OTU225	0	0	0	0
Proteobacteria	Deltaproteobacteria	Desulfovibrionales	Desulfovibrionaceae	Desulfovibrio	uncultured_bacterium_g__Desulfovibrio	OTU250	0	0	0	0
Bacteroidetes	Bacteroidia	Bacteroidales	Bacteroidales_S24-7_g	norank_f__Bacteroidales_S24-7_gro	uncultured_bacterium_g__norank_f__Bacteroidales_S24-7_group	OTU265	0	0	2	1
Proteobacteria	Betaproteobacteria	Burkholderiales	Comamonadaceae	unclassified_f__Comamonadaceae	unclassified_f__Comamonadaceae	OTU268	0	0	3	0
Firmicutes	Bacilli	Bacillales	Bacillaceae	unclassified_f__Bacillaceae	unclassified_f__Bacillaceae	OTU274	0	0	1	2
Firmicutes	Bacilli	Bacillales	Bacillaceae	Bacillus	Bacillus_anthracis_g__Bacillus	OTU275	0	0	2	0
Proteobacteria	Alphaproteobacteria	Rickettsiales	Mitochondria	norank_f__Mitochondria	Brassica_nigra__black_mustard_	OTU283	0	0	3	0
Firmicutes	Clostridia	Clostridiales	Ruminococcaceae	Anaerotruncus	Anaerotruncus_sp._G3_2012_	OTU296	0	0	2	1
Verrucomicrobia	Spartobacteria	Chthoniobacterales	DA101_soil_group	norank_f__DA101_soil_group	uncultured_Spartobacteria_bacterium_g__norank	OTU302	0	0	1	2
Actinobacteria	Actinobacteria	Acidimicrobiales	norank_o__Acidimicro	norank_o__Acidimicrobiales	uncultured_bacterium_g__norank_o__Acidimicrobiales	OTU314	0	0	3	0
Chloroflexi	Anaerolineae	Anaerolineales	Anaerolineaceae	norank_f__Anaerolineaceae	unclassified_g__norank_f__Anaerolineaceae	OTU316	0	0	1	2
Actinobacteria	Actinobacteria	Pseudonocardiales	Pseudonocardiaceae	Pseudonocardia	unclassified_g__Pseudonocardia	OTU351	0	0	1	2
Acidobacteria	Acidobacteria	Blastocatellales	Blastocatellaceae__Su	RB41	uncultured_Acidobacteria_bacterium_g__RB41	OTU359	0	0	0	3
Actinobacteria	Actinobacteria	Gaiellales	norank_o__Gaiellales	norank_o__Gaiellales	uncultured_bacterium_g__norank_o__Gaiellales	OTU370	0	0	0	3
Chloroflexi	Chloroflexia	Chloroflexales	Roseiflexaceae	Roseiflexus	uncultured_bacterium_g__Roseiflexus	OTU378	0	0	0	3
Chloroflexi	Chloroflexia	Chloroflexales	Roseiflexaceae	Roseiflexus	uncultured_bacterium_g__Roseiflexus	OTU384	0	0	1	2
Acidobacteria	Acidobacteria	Blastocatellales	Blastocatellaceae__Su	RB41	unclassified_g__RB41	OTU387	0	0	1	2
Acidobacteria	Acidobacteria	Solibacterales	Solibacteraceae__Sub	Bryobacter	uncultured_Acidobacterium_sp._g__Bryobacter	OTU388	0	0	2	1
Nitrospirae	Nitrospira	norank_c__Nitrospi	norank_c__Nitrospira	Nitrospira	unclassified_g__Nitrospira	OTU390	0	0	0	3
Actinobacteria	Actinobacteria	Acidimicrobiales	Acidimicrobiales_Ince	Candidatus_Microthrix	uncultured_bacterium_g__Candidatus_Microthrix	OTU393	0	0	1	2
Chloroflexi	Anaerolineae	Anaerolineales	Anaerolineaceae	norank_f__Anaerolineaceae	unclassified_g__norank_f__Anaerolineaceae	OTU397	0	0	0	3
Firmicutes	Clostridia	Clostridiales	Lachnospiraceae	norank_f__Lachnospiraceae	Lachnospiraceae_bacterium_A2	OTU398	0	0	1	2
Actinobacteria	Actinobacteria	Solirubrobacterales	Gsoil-1167	norank_f__Gsoil-1167	uncultured_bacterium_g__norank_f__Gsoil-1167	OTU406	0	0	0	3
Proteobacteria	Alphaproteobacteria	Rhizobiales	Bradyrhizobiaceae	Bosea	unclassified_g__Bosea	OTU426	0	0	1	2
Chloroflexi	Ktedonobacteria	JG30-KF-AS9	norank_o__JG30-KF-A	norank_o__JG30-KF-AS9	uncultured_bacterium_g__norank_o__JG30-KF-AS9	OTU440	0	0	1	2
Gemmatimonade	Gemmatimonadetes	Gemmatimonadales	Gemmatimonadaceae	Gemmatimonas	unclassified_g__Gemmatimonas	OTU441	0	0	1	2
Firmicutes	Clostridia	Clostridiales	Clostridiales_vadinBB6	norank_f__Clostridiales_vadinBB60_	unclassified_g__norank_f__Clostridiales_vadinBB60_group	OTU479	0	0	0	0
Firmicutes	Clostridia	Clostridiales	Lachnospiraceae	unclassified_f__Lachnospiraceae	unclassified_f__Lachnospiraceae	OTU514	0	0	0	0
Firmicutes	Clostridia	Clostridiales	Ruminococcaceae	unclassified_f__Ruminococcaceae	unclassified_f__Ruminococcaceae	OTU569	0	0	0	0
Firmicutes	Clostridia	Clostridiales	Ruminococcaceae	unclassified_f__Ruminococcaceae	unclassified_f__Ruminococcaceae	OTU760	0	0	0	0
Firmicutes	Clostridia	Clostridiales	Ruminococcaceae	Ruminococcaceae_UCG-005	unclassified_g__Ruminococcaceae_UCG-005	OTU814	0	1	0	0
Firmicutes	Clostridia	Clostridiales	Ruminococcaceae	Anaerotruncus	unclassified_g__Anaerotruncus	OTU44	0	0	0	0
Firmicutes	Clostridia	Clostridiales	Clostridiaceae_1	Clostridium_sensu_stricto_1	human_gut_metagenome_g__Clostridium_sensu_stricto_1	OTU77	0	0	0	0
Firmicutes	Clostridia	Clostridiales	Christensenellaceae	Christensenellaceae_R-7_group	unclassified_g__Christensenellaceae_R-7_group	OTU119	0	0	0	0
Bacteroidetes	Bacteroidia	Bacteroidales	Porphyromonadaceae	Odoribacter	uncultured_bacterium_g__Odoribacter	OTU210	0	0	1	0
Bacteroidetes	Bacteroidia	Bacteroidales	Bacteroidales_S24-7_g	norank_f__Bacteroidales_S24-7_gro	uncultured_bacterium_g__norank_f__Bacteroidales_S24-7_group	OTU217	1	0	1	0
Acidobacteria	Acidobacteria	norank_c__Acidoba	norank_c__Acidobacte	norank_c__Acidobacteria	uncultured_Acidobacteriales_bacterium_g__norank_c__Acidobacteria	OTU256	0	0	2	0
Gemmatimonade	Gemmatimonadetes	Gemmatimonadales	Gemmatimonadaceae	unclassified_f__Gemmatimonadacea	unclassified_f__Gemmatimonadaceae	OTU257	0	0	2	0
Acidobacteria	Acidobacteria	norank_c__Acidoba	norank_c__Acidobacte	norank_c__Acidobacteria	unclassified_g__norank_c__Acidobacteria	OTU264	0	0	2	0
Proteobacteria	Betaproteobacteria	Neisseriales	Neisseriaceae	Neisseria	unclassified_g__Neisseria	OTU271	0	0	2	0
Planctomycetes	OM190	norank_c__OM190	norank_c__OM190	norank_c__OM190	uncultured_bacterium_g__norank_c__OM190	OTU278	0	0	2	0
Actinobacteria	Actinobacteria	Coriobacteriales	Coriobacteriaceae	Parvibacter	uncultured_bacterium_g__Parvibacter	OTU292	0	0	2	0
Acidobacteria	Acidobacteria	norank_c__Acidoba	norank_c__Acidobacte	norank_c__Acidobacteria	uncultured_prokaryote_g__norank_c__Acidobacteria	OTU293	0	0	2	0
Actinobacteria	Actinobacteria	Gaiellales	norank_o__Gaiellales	norank_o__Gaiellales	uncultured_Rubrobacteria_bacterium_g__norank	OTU295	0	0	1	1
Proteobacteria	Gammaproteobacteria	BD7-8_marine_grou	norank_o__BD7-8_ma	norank_o__BD7-8_marine_group	unclassified_g__norank_o__BD7-8_marine_group	OTU297	0	0	1	1
Chloroflexi	Chloroflexia	Chloroflexales	Roseiflexaceae	Roseiflexus	uncultured_bacterium_g__Roseiflexus	OTU301	0	0	1	1
Actinobacteria	Actinobacteria	Propionibacteriales	Nocardioidaceae	unclassified_f__Nocardioidaceae	unclassified_f__Nocardioidaceae	OTU306	0	0	2	0
Saccharibacteria	norank_p__Saccharibact	norank_p__Sacchar	norank_p__Saccharib	norank_p__Saccharibacteria	uncultured_bacterium_g__norank_p__Saccharibacteria	OTU311	0	0	2	0
Acidobacteria	Acidobacteria	norank_c__Acidoba	norank_c__Acidobacte	norank_c__Acidobacteria	uncultured_bacterium_66	OTU315	0	0	2	0
Firmicutes	Bacilli	Bacillales	Bacillaceae	unclassified_f__Bacillaceae	unclassified_f__Bacillaceae	OTU319	0	0	1	1
Firmicutes	Clostridia	Clostridiales	Clostridiales_vadinBB6	norank_f__Clostridiales_vadinBB60_	uncultured_bacterium_g__norank_f__Clostridiales_vadinBB60_group	OTU340	0	0	0	0
Acidobacteria	Acidobacteria	norank_c__Acidoba	norank_c__Acidobacte	norank_c__Acidobacteria	unclassified_g__norank_c__Acidobacteria	OTU349	0	0	0	2
Saccharibacteria	norank_p__Saccharibact	norank_p__Sacchar	norank_p__Saccharib	norank_p__Saccharibacteria	uncultured_bacterium_g__norank_p__Saccharibacteria	OTU353	0	0	1	1
Chloroflexi	Anaerolineae	Anaerolineales	Anaerolineaceae	norank_f__Anaerolineaceae	unclassified_g__norank_f__Anaerolineaceae	OTU356	0	0	0	2
Bacteroidetes	Bacteroidia	Bacteroidales	Porphyromonadaceae	Odoribacter	uncultured_bacterium_g__Odoribacter	OTU363	0	0	0	2
Bacteroidetes	Bacteroidia	Bacteroidales	Bacteroidales_S24-7_g	norank_f__Bacteroidales_S24-7_gro	uncultured_bacterium_g__norank_f__Bacteroidales_S24-7_group	OTU364	0	0	0	2
Bacteroidetes	Cytophagia	Cytophagales	Cytophagaceae	Persicitalea	uncultured_bacterium_g__Persicitalea	OTU373	0	0	0	2
Proteobacteria	Deltaproteobacteria	Myxococcales	BIrii41	norank_f__BIrii41	unclassified_g__norank_f__BIrii41	OTU385	0	0	0	2
Chloroflexi	unclassified_p__Chlorofl	unclassified_p__Ch	unclassified_p__Chlor	unclassified_p__Chloroflexi	unclassified_p__Chloroflexi	OTU394	0	0	0	2
Planctomycetes	Planctomycetacia	Planctomycetales	Planctomycetaceae	norank_f__Planctomycetaceae	unclassified_g__norank_f__Planctomycetaceae	OTU400	0	0	0	2
Nitrospirae	Nitrospira	norank_c__Nitrospi	norank_c__Nitrospira	Nitrospira	unclassified_g__Nitrospira	OTU402	0	0	0	2
Actinobacteria	Actinobacteria	Acidimicrobiales	OM1_clade	norank_f__OM1_clade	unclassified_g__norank_f__OM1_clade	OTU412	0	0	0	2
Firmicutes	Clostridia	Clostridiales	Lachnospiraceae	norank_f__Lachnospiraceae	uncultured_bacterium_g__norank_f__Lachnospiraceae	OTU418	0	0	0	2
Gemmatimonade	Gemmatimonadetes	Gemmatimonadales	Gemmatimonadaceae	Gemmatimonas	uncultured_bacterium_g__Gemmatimonas	OTU424	0	0	0	2
Chloroflexi	Thermomicrobia	JG30-KF-CM45	norank_o__JG30-KF-C	norank_o__JG30-KF-CM45	uncultured_Sphaerobacter_sp._g__norank	OTU428	0	0	1	1
Proteobacteria	Alphaproteobacteria	Rhodospirillales	Acetobacteraceae	norank_f__Acetobacteraceae	uncultured_bacterium_g__norank_f__Acetobacteraceae	OTU434	0	0	0	2
Actinobacteria	Actinobacteria	Coriobacteriales	Coriobacteriaceae	Coriobacteriaceae_UCG-002	uncultured_bacterium_g__Coriobacteriaceae_UCG-002	OTU436	0	0	1	1
Acidobacteria	Acidobacteria	unclassified_c__Aci	unclassified_c__Acido	unclassified_c__Acidobacteria	unclassified_c__Acidobacteria	OTU438	0	0	0	2
Chloroflexi	Ktedonobacteria	C0119	norank_o__C0119	norank_o__C0119	unclassified_g__norank_o__C0119	OTU439	0	0	0	2
unclassified_k__r	unclassified_k__norank	unclassified_k__noi	unclassified_k__noran	unclassified_k__norank	unclassified_k__norank	OTU453	0	0	0	0
unclassified_k__r	unclassified_k__norank	unclassified_k__noi	unclassified_k__noran	unclassified_k__norank	unclassified_k__norank	OTU620	0	0	0	0
unclassified_k__r	unclassified_k__norank	unclassified_k__noi	unclassified_k__noran	unclassified_k__norank	unclassified_k__norank	OTU786	0	0	0	0

Phylum	Class	Order	Family	Genus	Species	OTU	Blue1	Blue2	Blue3	Blue4
Firmicutes	Clostridia	Clostridiales	Ruminococcaceae	Faecalibacterium	uncultured_bacterium_g__Faecalibacterium	OTU705	6189	666	5776	1943
Bacteroidetes	Bacteroidia	Bacteroidales	Rikenellaceae	Alistipes	gut_metagenome_g__Alistipes	OTU287	1673	7867	3320	4271
Firmicutes	Clostridia	Clostridiales	Lachnospiraceae	[Ruminococcus]_torques_group	uncultured_Clostridiales_bacterium_g__[Ruminococcus]_torques_group	OTU361	3722	3243	3223	3490
Proteobacteria	Gammaproteobacteria	Enterobacteriales	Enterobacteriaceae	Escherichia-Shigella	Escherichia_coli_g__Escherichia-Shigella	OTU589	332	263	5	95
Firmicutes	Clostridia	Clostridiales	Ruminococcaceae	Faecalibacterium	bacterium_ic1379	OTU50	0	3140	584	2364
Bacteroidetes	Bacteroidia	Bacteroidales	Rikenellaceae	Alistipes	unclassified_g__Alistipes	OTU501	669	981	2619	532
Firmicutes	Bacilli	Lactobacillales	Lactobacillaceae	Lactobacillus	unclassified_g__Lactobacillus	OTU445	188	1324	1018	4128
Firmicutes	Clostridia	Clostridiales	Ruminococcaceae	Subdoligranulum	uncultured_bacterium_g__Subdoligranulum	OTU816	11554	476	47	54
Firmicutes	Clostridia	Clostridiales	Lachnospiraceae	unclassified_f__Lachnospiraceae	unclassified_f__Lachnospiraceae	OTU581	780	593	327	186
Firmicutes	Clostridia	Clostridiales	Lachnospiraceae	Blautia	unclassified_g__Blautia	OTU78	1703	1178	676	248
Firmicutes	Clostridia	Clostridiales	Lachnospiraceae	Eisenbergiella	uncultured_bacterium_g__Eisenbergiella	OTU48	318	615	986	1344
Firmicutes	Clostridia	Clostridiales	Ruminococcaceae	Anaerotruncus	unclassified_g__Anaerotruncus	OTU213	674	436	185	664
Firmicutes	Clostridia	Clostridiales	Lachnospiraceae	unclassified_f__Lachnospiraceae	unclassified_f__Lachnospiraceae	OTU710	1550	66	1076	147
Firmicutes	Bacilli	Lactobacillales	Lactobacillaceae	Lactobacillus	Lactobacillus_salivarius	OTU775	1099	48	3	194
Firmicutes	Clostridia	Clostridiales	Lachnospiraceae	Shuttleworthia	uncultured_bacterium_g__Shuttleworthia	OTU683	277	630	243	553
Firmicutes	Clostridia	Clostridiales	Ruminococcaceae	Butyricicoccus	uncultured_bacterium_g__Butyricicoccus	OTU616	616	611	761	1994
Firmicutes	Clostridia	Clostridiales	Lachnospiraceae	[Ruminococcus]_torques_group	unclassified_g__[Ruminococcus]_torques_group	OTU196	922	13	13	19
Firmicutes	Clostridia	Clostridiales	Lachnospiraceae	Lachnoclostridium	unclassified_g__Lachnoclostridium	OTU527	498	144	211	395
Firmicutes	Clostridia	Clostridiales	Ruminococcaceae	Ruminiclostridium_9	unclassified_g__Ruminiclostridium_9	OTU174	284	306	234	439
Firmicutes	Clostridia	Clostridiales	Ruminococcaceae	Subdoligranulum	unclassified_g__Subdoligranulum	OTU88	187	739	7	405
Firmicutes	Clostridia	Clostridiales	Ruminococcaceae	Ruminococcaceae_UCG-014	unclassified_g__Ruminococcaceae_UCG-014	OTU712	70	760	126	197
Proteobacteria	Betaproteobacteria	Burkholderiales	Alcaligenaceae	Parasutterella	uncultured_organism_g__Parasutterella	OTU692	376	488	958	718
Firmicutes	Clostridia	Clostridiales	Ruminococcaceae	Faecalibacterium	uncultured_bacterium_g__Faecalibacterium	OTU122	5	133	2478	6
Firmicutes	Clostridia	Clostridiales	Clostridiales_vadinBB6	norank_f__Clostridiales_vadinBB60_	uncultured_bacterium_g__norank_f__Clostridiales_vadinBB60_group	OTU151	0	319	20	71
Firmicutes	Clostridia	Clostridiales	Ruminococcaceae	Faecalibacterium	uncultured_bacterium_g__Faecalibacterium	OTU70	18	311	238	348
Firmicutes	Clostridia	Clostridiales	Lachnospiraceae	unclassified_f__Lachnospiraceae	unclassified_f__Lachnospiraceae	OTU711	454	365	48	1706
Firmicutes	Bacilli	Lactobacillales	Lactobacillaceae	Lactobacillus	Lactobacillus_vaginalis	OTU489	8	4845	911	432
Firmicutes	Clostridia	Clostridiales	Lachnospiraceae	Sellimonas	unclassified_g__Sellimonas	OTU476	996	889	577	805
Firmicutes	Clostridia	Clostridiales	Lachnospiraceae	unclassified_f__Lachnospiraceae	unclassified_f__Lachnospiraceae	OTU179	208	262	71	408
Firmicutes	Clostridia	Clostridiales	Ruminococcaceae	norank_f__Ruminococcaceae	unclassified_g__norank_f__Ruminococcaceae	OTU191	173	80	1	2
Firmicutes	Erysipelotrichia	Erysipelotrichales	Erysipelotrichaceae	Erysipelatoclostridium	bacterium_ic1391	OTU561	15	2128	100	191
Firmicutes	Clostridia	Clostridiales	Ruminococcaceae	[Eubacterium]_coprostanoligenes_g	unclassified_g__[Eubacterium]_coprostanoligenes_group	OTU13	386	156	110	158
Firmicutes	Bacilli	Lactobacillales	Enterococcaceae	Enterococcus	Enterococcus_cecorum	OTU562	94	5	2	20
Firmicutes	Clostridia	Clostridiales	Lachnospiraceae	unclassified_f__Lachnospiraceae	unclassified_f__Lachnospiraceae	OTU484	930	126	77	328
Firmicutes	Clostridia	Clostridiales	Ruminococcaceae	unclassified_f__Ruminococcaceae	unclassified_f__Ruminococcaceae	OTU478	288	101	137	212
Firmicutes	Clostridia	Clostridiales	Lachnospiraceae	Lachnoclostridium	Clostridium_sp._BR31	OTU836	337	154	138	216
Firmicutes	Clostridia	Clostridiales	Ruminococcaceae	norank_f__Ruminococcaceae	Clostridiales_bacterium_24-4c	OTU743	0	63	674	463
Firmicutes	Bacilli	Bacillales	Bacillaceae	unclassified_f__Bacillaceae	unclassified_f__Bacillaceae	OTU851	43	1504	921	1178
Firmicutes	Clostridia	Clostridiales	Lachnospiraceae	[Ruminococcus]_torques_group	uncultured_Clostridiales_bacterium_g__[Ruminococcus]_torques_group	OTU774	16	19	9	4
Firmicutes	Clostridia	Clostridiales	Lachnospiraceae	[Eubacterium]_hallii_group	uncultured_bacterium_g__[Eubacterium]_hallii_group	OTU202	229	71	2	65
Firmicutes	Clostridia	Clostridiales	Lachnospiraceae	Anaerostipes	Anaerostipes_butyraticus	OTU189	1750	251	24	175
Firmicutes	Clostridia	Clostridiales	Ruminococcaceae	Ruminococcaceae_UCG-014	uncultured_bacterium_g__Ruminococcaceae_UCG-014	OTU578	6	253	72	120
Firmicutes	Clostridia	Clostridiales	Lachnospiraceae	Sellimonas	bacterium_P1C11	OTU218	225	709	131	178
Firmicutes	Clostridia	Clostridiales	Christensenellaceae	Christensenellaceae_R-7_group	unclassified_g__Christensenellaceae_R-7_group	OTU709	0	1039	0	5
Firmicutes	Clostridia	Clostridiales	Ruminococcaceae	Butyricicoccus	unclassified_g__Butyricicoccus	OTU33	452	448	612	1477
Bacteroidetes	Bacteroidia	Bacteroidales	Bacteroidaceae	Bacteroides	Bacteroides_acidifaciens	OTU226	0	0	0	0
Firmicutes	Clostridia	Clostridiales	Ruminococcaceae	Ruminococcaceae_UCG-014	unclassified_g__Ruminococcaceae_UCG-014	OTU65	80	120	67	67
Firmicutes	Clostridia	Clostridiales	Lachnospiraceae	Lachnoclostridium	unclassified_g__Lachnoclostridium	OTU469	250	193	290	334
Firmicutes	Clostridia	Clostridiales	Ruminococcaceae	Ruminococcaceae_UCG-005	uncultured_organism_g__Ruminococcaceae_UCG-005	OTU690	0	151	828	1107
Firmicutes	Erysipelotrichia	Erysipelotrichales	Erysipelotrichaceae	Erysipelatoclostridium	[Clostridium]_spiroforme	OTU625	7	1127	388	87
Tenericutes	Mollicutes	Mollicutes_RF9	norank_o__Mollicutes	norank_o__Mollicutes_RF9	uncultured_bacterium_g__norank_o__Mollicutes_RF9	OTU588	0	11	0	124
Firmicutes	Clostridia	Clostridiales	Lachnospiraceae	Tyzzerella	uncultured_organism_g__Tyzzerella	OTU795	76	482	392	406
Firmicutes	Clostridia	Clostridiales	Ruminococcaceae	Ruminiclostridium	uncultured_bacterium_g__Ruminiclostridium	OTU368	11	251	96	52
Firmicutes	Clostridia	Clostridiales	Ruminococcaceae	Butyricicoccus	Butyricicoccus_pullicaecorum_1.2	OTU769	17	487	1095	580
Firmicutes	Clostridia	Clostridiales	Ruminococcaceae	Ruminococcaceae_UCG-014	uncultured_bacterium_g__Ruminococcaceae_UCG-014	OTU346	40	187	349	102
Firmicutes	Clostridia	Clostridiales	Lachnospiraceae	norank_f__Lachnospiraceae	Clostridiales_bacterium_CHKCI001	OTU738	1	281	136	112
Proteobacteria	Gammaproteobacteria	Pseudomonadales	Pseudomonadaceae	Pseudomonas	Pseudomonas_brenneri	OTU580	58	280	65	176
Firmicutes	Clostridia	Clostridiales	Ruminococcaceae	norank_f__Ruminococcaceae	unclassified_g__norank_f__Ruminococcaceae	OTU460	6	53	1379	604
Firmicutes	Clostridia	Clostridiales	Ruminococcaceae	Ruminococcaceae_UCG-014	unidentified_rumen_bacterium_JW32	OTU565	1	232	265	18
Firmicutes	Clostridia	Clostridiales	Ruminococcaceae	Ruminococcaceae_UCG-014	uncultured_rumen_bacterium_g__Ruminococcaceae_UCG-014	OTU813	0	290	139	25
Firmicutes	Clostridia	Clostridiales	Lachnospiraceae	Lachnospiraceae_NK4A136_group	unclassified_g__Lachnospiraceae_NK4A136_group	OTU193	5	12	6	411
Firmicutes	Clostridia	Clostridiales	Clostridiales_vadinBB6	norank_f__Clostridiales_vadinBB60_	uncultured_bacterium_g__norank_f__Clostridiales_vadinBB60_group	OTU573	76	166	143	110
Firmicutes	Clostridia	Clostridiales	Lachnospiraceae	Coprococcus_1	uncultured_organism_g__Coprococcus_1	OTU591	184	338	164	106
Firmicutes	Clostridia	Clostridiales	Lachnospiraceae	unclassified_f__Lachnospiraceae	unclassified_f__Lachnospiraceae	OTU515	111	63	260	44
Firmicutes	Clostridia	Clostridiales	Ruminococcaceae	Ruminococcaceae_UCG-014	uncultured_organism_g__Ruminococcaceae_UCG-014	OTU457	344	89	11	16
Firmicutes	Clostridia	Clostridiales	Lachnospiraceae	Blautia	Blautia_hydrogenotrophica	OTU718	626	66	40	69
Firmicutes	Clostridia	Clostridiales	Ruminococcaceae	norank_f__Ruminococcaceae	unclassified_g__norank_f__Ruminococcaceae	OTU735	178	379	75	26
Firmicutes	Clostridia	Clostridiales	Peptostreptococcacea	Romboutsia	uncultured_bacterium_g__Romboutsia	OTU79	7	1	7	29
Firmicutes	Clostridia	Clostridiales	Ruminococcaceae	Anaerotruncus	uncultured_bacterium_g__Anaerotruncus	OTU499	80	60	27	117
Firmicutes	Clostridia	Clostridiales	Ruminococcaceae	Ruminococcaceae_UCG-014	uncultured_bacterium_g__Ruminococcaceae_UCG-014	OTU7	2	137	12	0
Firmicutes	Clostridia	Clostridiales	Ruminococcaceae	Ruminococcaceae_UCG-014	unclassified_g__Ruminococcaceae_UCG-014	OTU25	219	87	36	32
Firmicutes	Clostridia	Clostridiales	Ruminococcaceae	[Eubacterium]_coprostanoligenes_g	gut_metagenome_g__[Eubacterium]_coprostanoligenes_group	OTU214	328	468	0	16
Firmicutes	Clostridia	Clostridiales	Ruminococcaceae	Ruminococcaceae_UCG-005	unclassified_g__Ruminococcaceae_UCG-005	OTU160	140	198	43	155
Firmicutes	Clostridia	Clostridiales	Ruminococcaceae	Flavonifractor	uncultured_bacterium_g__Flavonifractor	OTU716	175	98	69	101
Firmicutes	Clostridia	Clostridiales	Ruminococcaceae	Ruminococcaceae_UCG-014	uncultured_bacterium_g__Ruminococcaceae_UCG-014	OTU691	50	134	78	60
Firmicutes	Clostridia	Clostridiales	Ruminococcaceae	Ruminococcaceae_UCG-005	uncultured_prokaryote_g__Ruminococcaceae_UCG-005	OTU504	3	158	1257	481
Firmicutes	Clostridia	Clostridiales	Ruminococcaceae	unclassified_f__Ruminococcaceae	unclassified_f__Ruminococcaceae	OTU496	17	136	524	1110
Firmicutes	Clostridia	Clostridiales	Ruminococcaceae	Butyricicoccus	unclassified_g__Butyricicoccus	OTU480	79	0	6	417
Firmicutes	Clostridia	Clostridiales	Lachnospiraceae	unclassified_f__Lachnospiraceae	unclassified_f__Lachnospiraceae	OTU533	3	185	71	176
Firmicutes	Bacilli	Lactobacillales	Streptococcaceae	Streptococcus	Streptococcus_gallolyticus_subsp._macedonicus	OTU474	432	0	1	1
Firmicutes	Clostridia	Clostridiales	Ruminococcaceae	norank_f__Ruminococcaceae	Ruminococcaceae_bacterium_AM2	OTU242	6	127	14	82
Firmicutes	Clostridia	Clostridiales	Clostridiales_vadinBB6	norank_f__Clostridiales_vadinBB60_	uncultured_organism_g__norank_f__Clostridiales_vadinBB60_group	OTU150	0	0	0	0
Firmicutes	Clostridia	Clostridiales	Lachnospiraceae	Shuttleworthia	uncultured_bacterium_g__Shuttleworthia	OTU507	26	202	121	110
Firmicutes	Clostridia	Clostridiales	Lachnospiraceae	Blautia	uncultured_bacterium_g__Blautia	OTU450	5	526	332	111
Firmicutes	Clostridia	Clostridiales	Ruminococcaceae	Ruminococcaceae_UCG-014	uncultured_rumen_bacterium_g__Ruminococcaceae_UCG-014	OTU230	0	1	509	0
Firmicutes	Clostridia	Clostridiales	Ruminococcaceae	Ruminococcaceae_UCG-004	unclassified_g__Ruminococcaceae_UCG-004	OTU355	113	84	97	67
Firmicutes	Clostridia	Clostridiales	Ruminococcaceae	norank_f__Ruminococcaceae	human_gut_metagenome_g__norank_f__Ruminococcaceae	OTU623	117	98	139	144
Firmicutes	Clostridia	Clostridiales	Lachnospiraceae	[Ruminococcus]_gauvreauii_group	bacterium_ic1296	OTU564	142	206	36	85
Firmicutes	Clostridia	Clostridiales	Ruminococcaceae	Anaerofilum	uncultured_bacterium_g__Anaerofilum	OTU123	2	25	19	78
Firmicutes	Clostridia	Clostridiales	Lachnospiraceae	unclassified_f__Lachnospiraceae	unclassified_f__Lachnospiraceae	OTU834	23	49	25	359
Firmicutes	Clostridia	Clostridiales	Clostridiales_vadinBB6	norank_f__Clostridiales_vadinBB60_	uncultured_bacterium_g__norank_f__Clostridiales_vadinBB60_group	OTU512	0	47	1741	42
Firmicutes	Erysipelotrichia	Erysipelotrichales	Erysipelotrichaceae	Erysipelatoclostridium	Candidatus_Stoquefichus_sp._SN16	OTU707	81	0	0	34
Firmicutes	Clostridia	Clostridiales	Lachnospiraceae	[Eubacterium]_hallii_group	uncultured_bacterium_g__[Eubacterium]_hallii_group	OTU848	9	202	12	6
Firmicutes	Clostridia	Clostridiales	Ruminococcaceae	Ruminiclostridium_5	uncultured_bacterium_g__Ruminiclostridium_5	OTU856	149	158	48	34
Firmicutes	Clostridia	Clostridiales	Christensenellaceae	Christensenellaceae_R-7_group	uncultured_bacterium_g__Christensenellaceae_R-7_group	OTU171	35	114	96	12
Firmicutes	Clostridia	Clostridiales	Lachnospiraceae	Blautia	unclassified_g__Blautia	OTU767	2	10	5	472
Firmicutes	Clostridia	Clostridiales	Lachnospiraceae	unclassified_f__Lachnospiraceae	unclassified_f__Lachnospiraceae	OTU828	67	5	13	106
Firmicutes	Clostridia	Clostridiales	Ruminococcaceae	Ruminococcaceae_UCG-005	uncultured_Clostridiales_bacterium_g__Ruminococcaceae_UCG-005	OTU815	9	141	690	291
Firmicutes	Clostridia	Clostridiales	Ruminococcaceae	unclassified_f__Ruminococcaceae	unclassified_f__Ruminococcaceae	OTU845	55	161	14	235
Firmicutes	Clostridia	Clostridiales	Lachnospiraceae	unclassified_f__Lachnospiraceae	unclassified_f__Lachnospiraceae	OTU770	179	121	16	22
Firmicutes	Clostridia	Clostridiales	Ruminococcaceae	Anaerotruncus	uncultured_bacterium_g__Anaerotruncus	OTU838	189	28	59	16
Firmicutes	Clostridia	Clostridiales	Lachnospiraceae	unclassified_f__Lachnospiraceae	unclassified_f__Lachnospiraceae	OTU82	168	65	32	76
Firmicutes	Clostridia	Clostridiales	Ruminococcaceae	Anaerotruncus	Anaerotruncus_colihominis_DSM_17241	OTU125	2	17	575	111
Firmicutes	Clostridia	Clostridiales	Clostridiales_vadinBB6	norank_f__Clostridiales_vadinBB60_	uncultured_Clostridia_bacterium_g__norank_f__Clostridiales_vadinBB60_group	OTU697	0	25	128	16
Proteobacteria	Gammaproteobacteria	Pseudomonadales	Pseudomonadaceae	Pseudomonas	unclassified_g__Pseudomonas	OTU58	23	114	29	74
Firmicutes	Clostridia	Clostridiales	Christensenellaceae	Christensenellaceae_R-7_group	unclassified_g__Christensenellaceae_R-7_group	OTU830	2	35	2	37
Firmicutes	Clostridia	Clostridiales	Ruminococcaceae	Subdoligranulum	uncultured_Subdoligranulum_sp.	OTU519	6	544	9	5
Firmicutes	Clostridia	Clostridiales	Lachnospiraceae	Marvinbryantia	unclassified_g__Marvinbryantia	OTU177	4	607	423	130
Firmicutes	Clostridia	Clostridiales	Clostridiales_vadinBB6	norank_f__Clostridiales_vadinBB60_	unclassified_g__norank_f__Clostridiales_vadinBB60_group	OTU107	9	79	152	90
Firmicutes	Clostridia	Clostridiales	Lachnospiraceae	unclassified_f__Lachnospiraceae	unclassified_f__Lachnospiraceae	OTU216	44	82	112	227

Firmicutes	Clostridia	Clostridiales	Ruminococcaceae	Ruminococcaceae_NK4A214_group	uncultured_organism_g__Ruminococcaceae_NK4A214_group	OTU703	35	76	13	102
Firmicutes	Clostridia	Clostridiales	Lachnospiraceae	unclassified_f__Lachnospiraceae	unclassified_f__Lachnospiraceae	OTU540	79	43	127	298
Firmicutes	Clostridia	Clostridiales	Defluviitaleaceae	Defluviitaleaceae_UCG-011	uncultured_bacterium_g__Defluviitaleaceae_UCG-011	OTU725	84	14	56	91
Firmicutes	Clostridia	Clostridiales	Ruminococcaceae	Ruminococcaceae_UCG-010	uncultured_bacterium_g__Ruminococcaceae_UCG-010	OTU799	17	76	98	137
Firmicutes	Clostridia	Clostridiales	Ruminococcaceae	Ruminococcaceae_UCG-014	unclassified_g__Ruminococcaceae_UCG-014	OTU163	0	78	70	5
Firmicutes	Clostridia	Clostridiales	Clostridiales_vadinBB6	norank_f__Clostridiales_vadinBB60_	unclassified_g__norank_f__Clostridiales_vadinBB60_group	OTU57	29	47	49	24
Tenericutes	Mollicutes	Mollicutes_RF9	norank_o__Mollicutes	norank_o__Mollicutes_RF9	unclassified_g__norank_o__Mollicutes_RF9	OTU294	1	0	10	49
Firmicutes	Clostridia	Clostridiales	Ruminococcaceae	Hydrogenoanaerobacterium	uncultured_bacterium_g__Hydrogenoanaerobacterium	OTU613	26	20	170	205
Firmicutes	Clostridia	Clostridiales	Ruminococcaceae	Ruminococcaceae_UCG-014	unclassified_g__Ruminococcaceae_UCG-014	OTU405	31	9	6	12
Firmicutes	Clostridia	Clostridiales	Ruminococcaceae	Ruminococcaceae_UCG-014	uncultured_bacterium_g__Ruminococcaceae_UCG-014	OTU570	2	115	27	5
Firmicutes	Clostridia	Clostridiales	Ruminococcaceae	norank_f__Ruminococcaceae	unclassified_g__norank_f__Ruminococcaceae	OTU313	151	48	17	143
Firmicutes	Bacilli	Lactobacillales	Lactobacillaceae	Lactobacillus	Lactobacillus_aviarius	OTU599	1	0	0	0
Firmicutes	Clostridia	Clostridiales	Lachnospiraceae	unclassified_f__Lachnospiraceae	unclassified_f__Lachnospiraceae	OTU717	36	27	30	27
Firmicutes	Clostridia	Clostridiales	Lachnospiraceae	Eisenbergiella	unclassified_g__Eisenbergiella	OTU443	66	36	12	309
Firmicutes	Clostridia	Clostridiales	Ruminococcaceae	norank_f__Ruminococcaceae	Ruminococcus_sp._16442	OTU113	36	35	0	34
Firmicutes	Clostridia	Clostridiales	Ruminococcaceae	Ruminococcus_1	unclassified_g__Ruminococcus_1	OTU140	0	37	4	60
Firmicutes	Clostridia	Clostridiales	Lachnospiraceae	Lachnospira	unclassified_g__Lachnospira	OTU170	1	55	175	91
Tenericutes	Mollicutes	Mollicutes_RF9	norank_o__Mollicutes	norank_o__Mollicutes_RF9	uncultured_bacterium_g__norank_o__Mollicutes_RF9	OTU823	82	8	211	48
Firmicutes	Clostridia	Clostridiales	Ruminococcaceae	unclassified_f__Ruminococcaceae	unclassified_f__Ruminococcaceae	OTU835	225	14	0	4
Firmicutes	Erysipelotrichia	Erysipelotrichales	Erysipelotrichaceae	norank_f__Erysipelotrichaceae	Clostridiales_bacterium_10-3b	OTU821	101	77	209	15
Firmicutes	Clostridia	Clostridiales	Peptococcaceae	norank_f__Peptococcaceae	uncultured_bacterium_g__norank_f__Peptococcaceae	OTU451	80	233	57	114
Firmicutes	Clostridia	Clostridiales	Lachnospiraceae	unclassified_f__Lachnospiraceae	unclassified_f__Lachnospiraceae	OTU135	0	0	0	0
Firmicutes	Clostridia	Clostridiales	Lachnospiraceae	unclassified_f__Lachnospiraceae	unclassified_f__Lachnospiraceae	OTU550	56	102	155	351
Firmicutes	Clostridia	Clostridiales	Ruminococcaceae	Anaerotruncus	uncultured_bacterium_g__Anaerotruncus	OTU556	3	14	35	11
Firmicutes	Erysipelotrichia	Erysipelotrichales	Erysipelotrichaceae	norank_f__Erysipelotrichaceae	Clostridiales_bacterium_60-7e	OTU583	343	25	20	42
Firmicutes	Clostridia	Clostridiales	Ruminococcaceae	Ruminococcaceae_UCG-014	unclassified_g__Ruminococcaceae_UCG-014	OTU200	25	18	71	18
Bacteroidetes	Bacteroidia	Bacteroidales	Rikenellaceae	Alistipes	gut_metagenome_g__Alistipes	OTU197	51	13	215	25
Tenericutes	Mollicutes	Mollicutes_RF9	norank_o__Mollicutes	norank_o__Mollicutes_RF9	gut_metagenome_g__norank_o__Mollicutes_RF9	OTU156	0	40	13	5
Firmicutes	Clostridia	Clostridiales	Ruminococcaceae	unclassified_f__Ruminococcaceae	unclassified_f__Ruminococcaceae	OTU66	451	1	7	5
Firmicutes	Erysipelotrichia	Erysipelotrichales	Erysipelotrichaceae	Faecalitalea	unclassified_g__Faecalitalea	OTU784	3	87	320	67
Firmicutes	Bacilli	Lactobacillales	Lactobacillaceae	Lactobacillus	Lactobacillus_johnsonii	OTU601	42	377	4	61
Bacteroidetes	Bacteroidia	Bacteroidales	Rikenellaceae	Alistipes	unclassified_g__Alistipes	OTU577	41	10	194	19
Firmicutes	Clostridia	Clostridiales	Ruminococcaceae	Subdoligranulum	unclassified_g__Subdoligranulum	OTU83	471	3	11	6
Proteobacteria	Deltaproteobacteria	Desulfovibrionales	Desulfovibrionaceae	Desulfovibrio	unclassified_g__Desulfovibrio	OTU636	23	46	190	56
Firmicutes	Clostridia	Clostridiales	Clostridiales_vadinBB6	norank_f__Clostridiales_vadinBB60_	unclassified_g__norank_f__Clostridiales_vadinBB60_group	OTU102	0	0	68	0
Firmicutes	Erysipelotrichia	Erysipelotrichales	Erysipelotrichaceae	Erysipelatoclostridium	unclassified_g__Erysipelatoclostridium	OTU8	51	66	51	75
Firmicutes	Clostridia	Clostridiales	Lachnospiraceae	[Ruminococcus]_torques_group	unclassified_g__[Ruminococcus]_torques_group	OTU531	113	4	25	6
Firmicutes	Clostridia	Clostridiales	Ruminococcaceae	Ruminococcaceae_UCG-014	unclassified_g__Ruminococcaceae_UCG-014	OTU614	0	0	0	0
Actinobacteria	Actinobacteria	Coriobacteriales	Coriobacteriaceae	Senegalimassilia	Coriobacteriaceae_bacterium_CHKCI002	OTU206	36	76	9	78
Firmicutes	Clostridia	Clostridiales	Ruminococcaceae	Ruminococcaceae_UCG-014	uncultured_bacterium_g__Ruminococcaceae_UCG-014	OTU842	1	94	28	1
Firmicutes	Clostridia	Clostridiales	Ruminococcaceae	Anaerotruncus	uncultured_organism_g__Anaerotruncus	OTU568	1	21	95	142
Firmicutes	Clostridia	Clostridiales	Ruminococcaceae	norank_f__Ruminococcaceae	unclassified_g__norank_f__Ruminococcaceae	OTU141	3	48	4	21
Firmicutes	Clostridia	Clostridiales	Clostridiales_vadinBB6	norank_f__Clostridiales_vadinBB60_	uncultured_bacterium_g__norank_f__Clostridiales_vadinBB60_group	OTU109	0	45	9	1
Cyanobacteria	Cyanobacteria	Gastranaerophilales	norank_o__Gastranae	norank_o__Gastranaerophilales	uncultured_rumen_bacterium_g__norank_o__Gastranaerophilales	OTU142	0	93	0	0
Firmicutes	Clostridia	Clostridiales	Lachnospiraceae	unclassified_f__Lachnospiraceae	unclassified_f__Lachnospiraceae	OTU699	3	15	53	104
Firmicutes	Clostridia	Clostridiales	Ruminococcaceae	Ruminococcaceae_UCG-010	gut_metagenome_g__Ruminococcaceae_UCG-010	OTU827	17	13	5	10
Firmicutes	Clostridia	Clostridiales	Lachnospiraceae	unclassified_f__Lachnospiraceae	unclassified_f__Lachnospiraceae	OTU612	84	85	130	35
Firmicutes	Clostridia	Clostridiales	Defluviitaleaceae	Defluviitaleaceae_UCG-011	uncultured_bacterium_g__Defluviitaleaceae_UCG-011	OTU567	4	3	12	69
Firmicutes	Clostridia	Clostridiales	Lachnospiraceae	Blautia	uncultured_bacterium_g__Blautia	OTU766	157	17	17	424
Firmicutes	Clostridia	Clostridiales	Christensenellaceae	Christensenellaceae_R-7_group	unclassified_g__Christensenellaceae_R-7_group	OTU148	5	55	24	11
Tenericutes	Mollicutes	Mollicutes_RF9	norank_o__Mollicutes	norank_o__Mollicutes_RF9	uncultured_bacterium_g__norank_o__Mollicutes_RF9	OTU559	0	19	71	11
Firmicutes	Clostridia	Clostridiales	Ruminococcaceae	Anaerotruncus	unclassified_g__Anaerotruncus	OTU657	199	53	77	0
Firmicutes	Clostridia	Clostridiales	Clostridiales_vadinBB6	norank_f__Clostridiales_vadinBB60_	uncultured_bacterium_g__norank_f__Clostridiales_vadinBB60_group	OTU22	0	167	108	252
Firmicutes	Clostridia	Clostridiales	Ruminococcaceae	Anaerotruncus	uncultured_Clostridiaceae_bacterium_g__Anaerotruncus	OTU618	37	18	34	4
Firmicutes	Clostridia	Clostridiales	Ruminococcaceae	Flavonifractor	uncultured_bacterium_g__Flavonifractor	OTU511	0	10	491	198
Firmicutes	Clostridia	Clostridiales	Clostridiales_vadinBB6	norank_f__Clostridiales_vadinBB60_	uncultured_bacterium_g__norank_f__Clostridiales_vadinBB60_group	OTU761	0	0	23	0
Firmicutes	Clostridia	Clostridiales	Clostridiales_vadinBB6	norank_f__Clostridiales_vadinBB60_	unclassified_g__norank_f__Clostridiales_vadinBB60_group	OTU231	0	12	132	5
Firmicutes	Clostridia	Clostridiales	Lachnospiraceae	unclassified_f__Lachnospiraceae	unclassified_f__Lachnospiraceae	OTU73	40	68	167	34
Firmicutes	Erysipelotrichia	Erysipelotrichales	Erysipelotrichaceae	Erysipelatoclostridium	Clostridiales_bacterium_CHKCI006	OTU700	29	61	34	17
Firmicutes	Clostridia	Clostridiales	Lachnospiraceae	unclassified_f__Lachnospiraceae	unclassified_f__Lachnospiraceae	OTU742	0	104	753	29
Firmicutes	Clostridia	Clostridiales	Ruminococcaceae	Anaerotruncus	uncultured_bacterium_g__Anaerotruncus	OTU146	62	3	0	11
Firmicutes	Clostridia	Clostridiales	Ruminococcaceae	Ruminococcaceae_UCG-010	uncultured_bacterium_g__Ruminococcaceae_UCG-010	OTU741	0	11	20	104
Firmicutes	Clostridia	Clostridiales	Ruminococcaceae	Subdoligranulum	unclassified_g__Subdoligranulum	OTU62	0	95	42	0
Firmicutes	Clostridia	Clostridiales	Lachnospiraceae	Blautia	uncultured_bacterium_g__Blautia	OTU466	67	26	57	62
Firmicutes	Clostridia	Clostridiales	Christensenellaceae	Christensenellaceae_R-7_group	uncultured_bacterium_g__Christensenellaceae_R-7_group	OTU746	13	67	38	10
Tenericutes	Mollicutes	Mollicutes_RF9	norank_o__Mollicutes	norank_o__Mollicutes_RF9	uncultured_bacterium_g__norank_o__Mollicutes_RF9	OTU192	0	8	21	16
Firmicutes	Clostridia	Clostridiales	Clostridiales_vadinBB6	norank_f__Clostridiales_vadinBB60_	uncultured_organism_g__norank_f__Clostridiales_vadinBB60_group	OTU103	0	2	0	11
Firmicutes	Clostridia	Clostridiales	Lachnospiraceae	unclassified_f__Lachnospiraceae	unclassified_f__Lachnospiraceae	OTU473	392	2	14	12
Firmicutes	Clostridia	Clostridiales	Ruminococcaceae	Ruminiclostridium_9	uncultured_bacterium_g__Ruminiclostridium_9	OTU110	1	132	52	240
Firmicutes	Bacilli	Lactobacillales	Lactobacillaceae	Lactobacillus	Lactobacillus_agilis	OTU607	0	0	0	0
Firmicutes	Clostridia	Clostridiales	Lachnospiraceae	Marvinbryantia	uncultured_organism_g__Marvinbryantia	OTU437	1	378	86	60
Firmicutes	Clostridia	Clostridiales	Ruminococcaceae	Ruminococcaceae_UCG-014	unclassified_g__Ruminococcaceae_UCG-014	OTU338	0	196	228	52
Firmicutes	Clostridia	Clostridiales	Clostridiales_vadinBB6	norank_f__Clostridiales_vadinBB60_	uncultured_rumen_bacterium_g__norank_f__Clostridiales_vadinBB60_group	OTU330	0	42	25	35
Tenericutes	Mollicutes	Mollicutes_RF9	norank_o__Mollicutes	norank_o__Mollicutes_RF9	uncultured_bacterium_g__norank_o__Mollicutes_RF9	OTU28	0	40	7	1
Firmicutes	Clostridia	Clostridiales	Lachnospiraceae	Coprococcus_1	unclassified_g__Coprococcus_1	OTU539	3	162	28	28
Firmicutes	Clostridia	Clostridiales	Lachnospiraceae	unclassified_f__Lachnospiraceae	unclassified_f__Lachnospiraceae	OTU642	11	37	71	115
Firmicutes	Clostridia	Clostridiales	Ruminococcaceae	Ruminococcaceae_UCG-014	unclassified_g__Ruminococcaceae_UCG-014	OTU590	0	15	11	9
Firmicutes	Clostridia	Clostridiales	Ruminococcaceae	norank_f__Ruminococcaceae	unclassified_g__norank_f__Ruminococcaceae	OTU701	60	5	9	436
Firmicutes	Clostridia	Clostridiales	Ruminococcaceae	Anaerotruncus	uncultured_bacterium_g__Anaerotruncus	OTU249	0	85	186	24
Firmicutes	Clostridia	Clostridiales	Ruminococcaceae	Anaerotruncus	uncultured_bacterium_g__Anaerotruncus	OTU248	12	25	10	57
Firmicutes	Clostridia	Clostridiales	Ruminococcaceae	Ruminiclostridium_5	unclassified_g__Ruminiclostridium_5	OTU826	0	41	30	32
Firmicutes	Clostridia	Clostridiales	Family_XIII	[Eubacterium]_nodatum_group	Eubacteriaceae_bacterium_Marseille-P2843	OTU585	27	36	1	46
Firmicutes	Clostridia	Clostridiales	Christensenellaceae	Christensenellaceae_R-7_group	uncultured_bacterium_g__Christensenellaceae_R-7_group	OTU167	51	23	14	19
Firmicutes	Clostridia	Clostridiales	Lachnospiraceae	norank_f__Lachnospiraceae	Clostridiales_bacterium_CHKCI001	OTU520	3	18	34	26
Firmicutes	Clostridia	Clostridiales	Ruminococcaceae	unclassified_f__Ruminococcaceae	unclassified_f__Ruminococcaceae	OTU238	8	5	12	16
Firmicutes	Clostridia	Clostridiales	Ruminococcaceae	unclassified_f__Ruminococcaceae	unclassified_f__Ruminococcaceae	OTU133	80	25	4	10
Tenericutes	Mollicutes	Mollicutes_RF9	norank_o__Mollicutes	norank_o__Mollicutes_RF9	uncultured_bacterium_g__norank_o__Mollicutes_RF9	OTU606	1	43	38	30
Firmicutes	Clostridia	Clostridiales	Ruminococcaceae	Ruminococcaceae_UCG-009	uncultured_bacterium_g__Ruminococcaceae_UCG-009	OTU779	24	13	90	221
Firmicutes	Clostridia	Clostridiales	Ruminococcaceae	Ruminococcaceae_NK4A214_group	uncultured_organism_g__Ruminococcaceae_NK4A214_group	OTU410	0	4	27	4
Verrucomicrobia	Verrucomicrobiae	Verrucomicrobiales	Verrucomicrobiaceae	Akkermansia	unclassified_g__Akkermansia	OTU344	0	56	49	0
Firmicutes	Clostridia	Clostridiales	Clostridiales_vadinBB6	norank_f__Clostridiales_vadinBB60_	uncultured_bacterium_g__norank_f__Clostridiales_vadinBB60_group	OTU1	0	1	23	1
Firmicutes	Clostridia	Clostridiales	Ruminococcaceae	Oscillospira	uncultured_bacterium_g__Oscillospira	OTU158	5	45	78	52
Firmicutes	Clostridia	Clostridiales	Lachnospiraceae	Lachnospiraceae_NK4A136_group	unclassified_g__Lachnospiraceae_NK4A136_group	OTU187	0	0	43	3
Firmicutes	Clostridia	Clostridiales	Lachnospiraceae	unclassified_f__Lachnospiraceae	unclassified_f__Lachnospiraceae	OTU463	0	70	24	93
Firmicutes	Clostridia	Clostridiales	Peptostreptococcacea	unclassified_f__Peptostreptococcac	unclassified_f__Peptostreptococcaceae	OTU526	26	5	0	5
Firmicutes	Bacilli	Lactobacillales	Lactobacillaceae	Lactobacillus	unclassified_g__Lactobacillus	OTU448	1	29	62	47
Firmicutes	Clostridia	Clostridiales	Ruminococcaceae	Ruminococcaceae_NK4A214_group	uncultured_rumen_bacterium_g__Ruminococcaceae_NK4A214_group	OTU181	0	0	137	0
Firmicutes	Clostridia	Clostridiales	Ruminococcaceae	Ruminococcaceae_UCG-014	unclassified_g__Ruminococcaceae_UCG-014	OTU822	0	133	7	2
Firmicutes	Clostridia	Clostridiales	Ruminococcaceae	Ruminococcaceae_UCG-013	unclassified_g__Ruminococcaceae_UCG-013	OTU458	1	277	7	0
Firmicutes	Clostridia	Clostridiales	Clostridiales_vadinBB6	norank_f__Clostridiales_vadinBB60_	unclassified_g__norank_f__Clostridiales_vadinBB60_group	OTU198	1	4	74	11
Firmicutes	Clostridia	Clostridiales	Clostridiales_vadinBB6	norank_f__Clostridiales_vadinBB60_	unclassified_g__norank_f__Clostridiales_vadinBB60_group	OTU241	0	0	0	0
Tenericutes	Mollicutes	Mollicutes_RF9	norank_o__Mollicutes	norank_o__Mollicutes_RF9	unclassified_g__norank_o__Mollicutes_RF9	OTU803	0	160	11	5
Firmicutes	Clostridia	Clostridiales	Lachnospiraceae	unclassified_f__Lachnospiraceae	unclassified_f__Lachnospiraceae	OTU81	98	5	3	3
Firmicutes	Clostridia	Clostridiales	Ruminococcaceae	Ruminococcaceae_UCG-014	unclassified_g__Ruminococcaceae_UCG-014	OTU12	0	46	9	1
Firmicutes	Clostridia	Clostridiales	Christensenellaceae	Christensenellaceae_R-7_group	unclassified_g__Christensenellaceae_R-7_group	OTU165	0	0	0	0
Firmicutes	Clostridia	Clostridiales	unclassified_o__Clostr	unclassified_o__Clostridiales	unclassified_o__Clostridiales	OTU682	2	0	0	0
Firmicutes	Clostridia	Clostridiales	Ruminococcaceae	unclassified_f__Ruminococcaceae	unclassified_f__Ruminococcaceae	OTU679	0	26	6	20
Firmicutes	Clostridia	Clostridiales	Ruminococcaceae	unclassified_f__Ruminococcaceae	unclassified_f__Ruminococcaceae	OTU455	110	8	0	0
Firmicutes	Clostridia	Clostridiales	Family_XIII	unclassified_f__Family_XIII	unclassified_f__Family_XIII	OTU488	37	37	5	7
Firmicutes	Clostridia	Clostridiales	Ruminococcaceae	Ruminococcaceae_UCG-013	uncultured_bacterium_g__Ruminococcaceae_UCG-013	OTU498	47	7	67	37
Firmicutes	Clostridia	Clostridiales	Ruminococcaceae	Anaerotruncus	unclassified_g__Anaerotruncus	OTU566	0	18	19	237

Firmicutes	Clostridia	Clostridiales	Clostridiales_vadinBB6	norank_f__Clostridiales_vadinBB60_	uncultured_Firmicutes_bacterium_g__norank_f__Clostridiales_vadinBB60_group	OTU638	0	21	18	37
Firmicutes	Clostridia	Clostridiales	Clostridiales_vadinBB6	norank_f__Clostridiales_vadinBB60_	uncultured_bacterium_g__norank_f__Clostridiales_vadinBB60_group	OTU3	0	0	13	0
Firmicutes	Clostridia	Clostridiales	Clostridiales_vadinBB6	norank_f__Clostridiales_vadinBB60_	uncultured_bacterium_g__norank_f__Clostridiales_vadinBB60_group	OTU494	4	66	33	23
Firmicutes	Clostridia	Clostridiales	Ruminococcaceae	Ruminiclostridium_5	uncultured_bacterium_g__Ruminiclostridium_5	OTU849	27	11	7	12
Firmicutes	Clostridia	Clostridiales	Ruminococcaceae	Ruminococcaceae_UCG-014	uncultured_bacterium_g__Ruminococcaceae_UCG-014	OTU114	0	166	2	0
Firmicutes	Clostridia	Clostridiales	Ruminococcaceae	Ruminococcaceae_UCG-010	uncultured_bacterium_g__Ruminococcaceae_UCG-010	OTU326	0	92	30	132
Firmicutes	Clostridia	Clostridiales	Ruminococcaceae	Ruminococcaceae_UCG-014	uncultured_bacterium_g__Ruminococcaceae_UCG-014	OTU749	1	7	3	12
Firmicutes	Clostridia	Clostridiales	Ruminococcaceae	[Eubacterium]_coprostanoligenes_g	unclassified_g__[Eubacterium]_coprostanoligenes_group	OTU233	0	0	3	0
Firmicutes	Clostridia	Clostridiales	Ruminococcaceae	Ruminococcaceae_UCG-014	unidentified_rumen_bacterium_JW32	OTU36	0	20	0	10
Firmicutes	Clostridia	Clostridiales	Ruminococcaceae	Ruminococcaceae_UCG-008	uncultured_bacterium_g__Ruminococcaceae_UCG-008	OTU39	0	0	0	0
Firmicutes	Clostridia	Clostridiales	Family_XIII	Family_XIII_UCG-001	uncultured_bacterium_g__Family_XIII_UCG-001	OTU329	27	16	23	29
Firmicutes	Clostridia	Clostridiales	Ruminococcaceae	Ruminiclostridium_5	uncultured_bacterium_g__Ruminiclostridium_5	OTU75	22	11	11	26
Firmicutes	Clostridia	Clostridiales	Peptococcaceae	norank_f__Peptococcaceae	uncultured_organism_g__norank_f__Peptococcaceae	OTU343	0	0	1	0
Firmicutes	Clostridia	Clostridiales	Ruminococcaceae	Anaerofilum	uncultured_bacterium_g__Anaerofilum	OTU660	4	20	12	3
Firmicutes	Clostridia	Clostridiales	Lachnospiraceae	Marvinbryantia	uncultured_bacterium_g__Marvinbryantia	OTU536	56	43	156	7
Firmicutes	Clostridia	Clostridiales	Ruminococcaceae	Anaerotruncus	unclassified_g__Anaerotruncus	OTU10	20	20	12	24
Firmicutes	Clostridia	Clostridiales	Clostridiales_vadinBB6	norank_f__Clostridiales_vadinBB60_	uncultured_bacterium_g__norank_f__Clostridiales_vadinBB60_group	OTU678	0	49	348	34
Firmicutes	Clostridia	Clostridiales	Ruminococcaceae	Ruminiclostridium_5	unclassified_g__Ruminiclostridium_5	OTU84	26	62	8	9
Firmicutes	Clostridia	Clostridiales	unclassified_o__Clostr	unclassified_o__Clostridiales	unclassified_o__Clostridiales	OTU137	25	1	37	4
Tenericutes	Mollicutes	Mollicutes_RF9	norank_o__Mollicutes	norank_o__Mollicutes_RF9	uncultured_bacterium_g__norank_o__Mollicutes_RF9	OTU604	0	11	1	0
Firmicutes	Erysipelotrichia	Erysipelotrichales	Erysipelotrichaceae	norank_f__Erysipelotrichaceae	uncultured_Erysipelotrichaceae_bacterium_g__norank	OTU772	0	0	1	0
Tenericutes	Mollicutes	Anaeroplasmatales	Anaeroplasmataceae	Anaeroplasma	uncultured_bacterium_g__Anaeroplasma	OTU801	0	7	24	96
Firmicutes	Clostridia	Clostridiales	Ruminococcaceae	Ruminococcaceae_UCG-014	unclassified_g__Ruminococcaceae_UCG-014	OTU220	0	0	0	0
Firmicutes	Clostridia	Clostridiales	Ruminococcaceae	Ruminiclostridium_9	unclassified_g__Ruminiclostridium_9	OTU587	17	3	5	9
Firmicutes	Clostridia	Clostridiales	Ruminococcaceae	norank_f__Ruminococcaceae	human_gut_metagenome_g__norank_f__Ruminococcaceae	OTU85	8	3	8	5
Cyanobacteria	Cyanobacteria	Gastranaerophilales	norank_o__Gastranae	norank_o__Gastranaerophilales	uncultured_bacterium_g__norank_o__Gastranaerophilales	OTU502	0	19	55	76
Firmicutes	Clostridia	Clostridiales	Clostridiales_vadinBB6	norank_f__Clostridiales_vadinBB60_	uncultured_bacterium_g__norank_f__Clostridiales_vadinBB60_group	OTU17	0	15	58	51
Firmicutes	Clostridia	Clostridiales	Lachnospiraceae	unclassified_f__Lachnospiraceae	unclassified_f__Lachnospiraceae	OTU513	33	10	6	8
Firmicutes	Clostridia	Clostridiales	Ruminococcaceae	unclassified_f__Ruminococcaceae	unclassified_f__Ruminococcaceae	OTU166	0	7	31	37
Tenericutes	Mollicutes	NB1-n	norank_o__NB1-n	norank_o__NB1-n	uncultured_organism_g__norank_o__NB1-n	OTU603	0	17	26	2
Firmicutes	Clostridia	Clostridiales	Ruminococcaceae	Ruminococcaceae_UCG-013	uncultured_bacterium_g__Ruminococcaceae_UCG-013	OTU720	2	37	2	0
Firmicutes	Clostridia	Clostridiales	Ruminococcaceae	Ruminococcaceae_UCG-014	unclassified_g__Ruminococcaceae_UCG-014	OTU347	2	4	79	2
Firmicutes	Clostridia	Clostridiales	Ruminococcaceae	Ruminiclostridium	uncultured_bacterium_g__Ruminiclostridium	OTU576	0	0	33	121
Cyanobacteria	Cyanobacteria	Gastranaerophilales	norank_o__Gastranae	norank_o__Gastranaerophilales	unclassified_g__norank_o__Gastranaerophilales	OTU563	0	14	27	109
Firmicutes	Clostridia	Clostridiales	Lachnospiraceae	unclassified_f__Lachnospiraceae	unclassified_f__Lachnospiraceae	OTU731	6	4	1	24
Firmicutes	Clostridia	Clostridiales	Lachnospiraceae	Roseburia	unclassified_g__Roseburia	OTU449	0	5	2	1
Firmicutes	Clostridia	Clostridiales	Clostridiales_vadinBB6	norank_f__Clostridiales_vadinBB60_	uncultured_bacterium_g__norank_f__Clostridiales_vadinBB60_group	OTU669	0	48	0	102
Tenericutes	Mollicutes	Mollicutes_RF9	norank_o__Mollicutes	norank_o__Mollicutes_RF9	unclassified_g__norank_o__Mollicutes_RF9	OTU104	0	0	3	0
Firmicutes	Clostridia	Clostridiales	Lachnospiraceae	Lachnospiraceae_UCG-010	uncultured_bacterium_g__Lachnospiraceae_UCG-010	OTU663	15	10	13	21
Firmicutes	Clostridia	Clostridiales	Ruminococcaceae	unclassified_f__Ruminococcaceae	unclassified_f__Ruminococcaceae	OTU847	1	3	50	7
Firmicutes	Clostridia	Clostridiales	Ruminococcaceae	Ruminococcaceae_UCG-013	uncultured_bacterium_g__Ruminococcaceae_UCG-013	OTU721	1	25	0	6
Tenericutes	Mollicutes	Mollicutes_RF9	norank_o__Mollicutes	norank_o__Mollicutes_RF9	uncultured_bacterium_g__norank_o__Mollicutes_RF9	OTU627	0	30	37	70
Firmicutes	Clostridia	Clostridiales	Ruminococcaceae	[Eubacterium]_coprostanoligenes_g	unclassified_g__[Eubacterium]_coprostanoligenes_group	OTU121	0	1	1	0
Firmicutes	Clostridia	Clostridiales	Clostridiales_vadinBB6	norank_f__Clostridiales_vadinBB60_	unclassified_g__norank_f__Clostridiales_vadinBB60_group	OTU180	9	39	7	48
Firmicutes	Clostridia	Clostridiales	Christensenellaceae	Christensenellaceae_R-7_group	unclassified_g__Christensenellaceae_R-7_group	OTU54	0	6	0	9
Firmicutes	Clostridia	Clostridiales	Ruminococcaceae	norank_f__Ruminococcaceae	unclassified_g__norank_f__Ruminococcaceae	OTU534	22	5	0	0
Firmicutes	Clostridia	Clostridiales	Ruminococcaceae	unclassified_f__Ruminococcaceae	unclassified_f__Ruminococcaceae	OTU493	10	11	17	10
Firmicutes	Clostridia	Clostridiales	Lachnospiraceae	norank_f__Lachnospiraceae	unclassified_g__norank_f__Lachnospiraceae	OTU560	42	2	14	30
Firmicutes	Clostridia	Clostridiales	Lachnospiraceae	[Eubacterium]_ventriosum_group	uncultured_organism_g__[Eubacterium]_ventriosum_group	OTU464	1	32	7	8
Tenericutes	Mollicutes	Mollicutes_RF9	norank_o__Mollicutes	norank_o__Mollicutes_RF9	unclassified_g__norank_o__Mollicutes_RF9	OTU234	0	1	46	0
Firmicutes	Clostridia	Clostridiales	Lachnospiraceae	unclassified_f__Lachnospiraceae	unclassified_f__Lachnospiraceae	OTU64	11	9	31	4
Tenericutes	Mollicutes	Mollicutes_RF9	norank_o__Mollicutes	norank_o__Mollicutes_RF9	uncultured_bacterium_g__norank_o__Mollicutes_RF9	OTU602	0	0	4	1
Firmicutes	Clostridia	Clostridiales	Ruminococcaceae	Anaerotruncus	uncultured_bacterium_g__Anaerotruncus	OTU6	0	17	166	110
Firmicutes	Bacilli	Lactobacillales	Lactobacillaceae	Lactobacillus	Lactobacillus_murinus_g__Lactobacillus	OTU843	0	0	1	0
Firmicutes	Clostridia	Clostridiales	Christensenellaceae	Christensenellaceae_R-7_group	uncultured_prokaryote_g__Christensenellaceae_R-7_group	OTU67	0	31	34	4
Firmicutes	Clostridia	Clostridiales	Clostridiales_vadinBB6	norank_f__Clostridiales_vadinBB60_	uncultured_bacterium_g__norank_f__Clostridiales_vadinBB60_group	OTU748	0	30	0	13
Firmicutes	Clostridia	Clostridiales	Lachnospiraceae	Coprococcus_1	unclassified_g__Coprococcus_1	OTU739	56	12	15	8
Tenericutes	Mollicutes	Mollicutes_RF9	norank_o__Mollicutes	norank_o__Mollicutes_RF9	unclassified_g__norank_o__Mollicutes_RF9	OTU229	0	0	1	9
Firmicutes	Clostridia	Clostridiales	Clostridiales_vadinBB6	norank_f__Clostridiales_vadinBB60_	uncultured_bacterium_g__norank_f__Clostridiales_vadinBB60_group	OTU23	0	0	13	1
Firmicutes	Clostridia	Clostridiales	Clostridiales_vadinBB6	norank_f__Clostridiales_vadinBB60_	uncultured_bacterium_g__norank_f__Clostridiales_vadinBB60_group	OTU131	0	0	0	0
Firmicutes	Clostridia	Clostridiales	Ruminococcaceae	Ruminococcaceae_UCG-014	unclassified_g__Ruminococcaceae_UCG-014	OTU837	0	21	4	0
Firmicutes	Clostridia	Clostridiales	unclassified_o__Clostr	unclassified_o__Clostridiales	unclassified_o__Clostridiales	OTU91	0	10	6	9
Firmicutes	Clostridia	Clostridiales	Lachnospiraceae	Roseburia	unclassified_g__Roseburia	OTU43	56	1	0	1
Firmicutes	Clostridia	Clostridiales	Lachnospiraceae	unclassified_f__Lachnospiraceae	unclassified_f__Lachnospiraceae	OTU11	8	1	14	2
Proteobacteria	Betaproteobacteria	Burkholderiales	Oxalobacteraceae	Oxalobacter	unclassified_g__Oxalobacter	OTU557	0	0	0	0
Firmicutes	Clostridia	Clostridiales	Ruminococcaceae	Faecalibacterium	unclassified_g__Faecalibacterium	OTU219	7	0	0	0
Firmicutes	Clostridia	Clostridiales	Ruminococcaceae	Ruminiclostridium_5	uncultured_bacterium_g__Ruminiclostridium_5	OTU59	12	4	4	3
Firmicutes	Clostridia	Clostridiales	Lachnospiraceae	Lachnospiraceae_UCG-010	uncultured_bacterium_g__Lachnospiraceae_UCG-010	OTU755	1	1	15	70
Bacteroidetes	Bacteroidia	Bacteroidales	Rikenellaceae	Alistipes	uncultured_bacterium_g__Alistipes	OTU765	0	0	0	0
Firmicutes	Erysipelotrichia	Erysipelotrichales	Erysipelotrichaceae	[Clostridium]_innocuum_group	uncultured_bacterium_g__[Clostridium]_innocuum_group	OTU794	0	0	0	1
Firmicutes	Clostridia	Clostridiales	unclassified_o__Clostr	unclassified_o__Clostridiales	unclassified_o__Clostridiales	OTU673	0	71	5	4
Firmicutes	Clostridia	Clostridiales	Clostridiales_vadinBB6	norank_f__Clostridiales_vadinBB60_	gut_metagenome_g__norank_f__Clostridiales_vadinBB60_group	OTU548	1	4	14	70
Tenericutes	Mollicutes	Mollicutes_RF9	norank_o__Mollicutes	norank_o__Mollicutes_RF9	uncultured_bacterium_g__norank_o__Mollicutes_RF9	OTU544	0	50	8	65
Cyanobacteria	Cyanobacteria	Gastranaerophilales	norank_o__Gastranae	norank_o__Gastranaerophilales	unclassified_g__norank_o__Gastranaerophilales	OTU155	0	4	7	16
Firmicutes	Clostridia	Clostridiales	Clostridiales_vadinBB6	norank_f__Clostridiales_vadinBB60_	uncultured_bacterium_g__norank_f__Clostridiales_vadinBB60_group	OTU481	0	0	10	10
Firmicutes	Clostridia	Clostridiales	Lachnospiraceae	unclassified_f__Lachnospiraceae	unclassified_f__Lachnospiraceae	OTU467	0	7	5	5
Firmicutes	Clostridia	Clostridiales	Ruminococcaceae	norank_f__Ruminococcaceae	unclassified_g__norank_f__Ruminococcaceae	OTU622	0	0	7	117
Firmicutes	Clostridia	Clostridiales	Lachnospiraceae	unclassified_f__Lachnospiraceae	unclassified_f__Lachnospiraceae	OTU201	26	0	3	5
Firmicutes	Clostridia	Clostridiales	Lachnospiraceae	norank_f__Lachnospiraceae	uncultured_organism_g__norank_f__Lachnospiraceae	OTU654	1	25	24	1
Firmicutes	Clostridia	Clostridiales	Ruminococcaceae	Ruminococcaceae_UCG-014	unclassified_g__Ruminococcaceae_UCG-014	OTU575	0	71	13	7
Tenericutes	Mollicutes	Mollicutes_RF9	norank_o__Mollicutes	norank_o__Mollicutes_RF9	unclassified_g__norank_o__Mollicutes_RF9	OTU600	0	4	3	16
Firmicutes	Clostridia	Clostridiales	Ruminococcaceae	unclassified_f__Ruminococcaceae	unclassified_f__Ruminococcaceae	OTU728	14	0	5	4
Firmicutes	Clostridia	Clostridiales	Ruminococcaceae	Anaerotruncus	unclassified_g__Anaerotruncus	OTU159	1	0	69	11
Tenericutes	Mollicutes	Mollicutes_RF9	norank_o__Mollicutes	norank_o__Mollicutes_RF9	uncultured_bacterium_g__norank_o__Mollicutes_RF9	OTU608	0	5	2	1
Firmicutes	Clostridia	Clostridiales	Ruminococcaceae	Ruminococcaceae_UCG-010	uncultured_bacterium_g__Ruminococcaceae_UCG-010	OTU674	6	7	0	0
Firmicutes	Clostridia	Clostridiales	Lachnospiraceae	Lachnospiraceae_FCS020_group	uncultured_bacterium_g__Lachnospiraceae_FCS020_group	OTU802	11	3	9	31
Firmicutes	Clostridia	Clostridiales	Ruminococcaceae	Caproiciproducens	uncultured_bacterium_g__Caproiciproducens	OTU68	5	11	10	4
Firmicutes	Clostridia	Clostridiales	Peptococcaceae	norank_f__Peptococcaceae	unclassified_g__norank_f__Peptococcaceae	OTU586	0	3	3	4
Firmicutes	Clostridia	Clostridiales	Ruminococcaceae	Anaerotruncus	uncultured_bacterium_g__Anaerotruncus	OTU175	24	5	5	4
Firmicutes	Clostridia	Clostridiales	Lachnospiraceae	unclassified_f__Lachnospiraceae	unclassified_f__Lachnospiraceae	OTU854	253	0	28	5
Firmicutes	Clostridia	Clostridiales	Christensenellaceae	Christensenellaceae_R-7_group	uncultured_bacterium_g__Christensenellaceae_R-7_group	OTU164	1	7	4	1
Firmicutes	Clostridia	Clostridiales	Ruminococcaceae	Ruminococcus_2	uncultured_bacterium_g__Ruminococcus_2	OTU447	0	1	0	0
Actinobacteria	Actinobacteria	Coriobacteriales	Coriobacteriaceae	unclassified_f__Coriobacteriaceae	unclassified_f__Coriobacteriaceae	OTU490	11	15	0	39
Proteobacteria	Alphaproteobacteria	Rhizobiales	Brucellaceae	Brucella	Brucella_melitensis_g__Brucella	OTU812	0	0	0	0
Firmicutes	Clostridia	Clostridiales	Ruminococcaceae	Ruminiclostridium_1	uncultured_bacterium_g__Ruminiclostridium_1	OTU485	6	4	1	0
Tenericutes	Mollicutes	Mollicutes_RF9	norank_o__Mollicutes	norank_o__Mollicutes_RF9	unclassified_g__norank_o__Mollicutes_RF9	OTU611	0	13	9	0
Firmicutes	Clostridia	Clostridiales	Ruminococcaceae	Ruminococcaceae_UCG-014	unclassified_g__Ruminococcaceae_UCG-014	OTU100	0	0	3	0
Firmicutes	Clostridia	Clostridiales	Ruminococcaceae	norank_f__Ruminococcaceae	unclassified_g__norank_f__Ruminococcaceae	OTU98	0	0	0	0
Firmicutes	Bacilli	Lactobacillales	Enterococcaceae	Enterococcus	Enterococcus_faecalis_g__Enterococcus	OTU763	4	0	1	7
Firmicutes	Bacilli	Lactobacillales	Lactobacillaceae	Lactobacillus	Lactobacillus_coleohominis	OTU628	0	4	53	19
Firmicutes	Erysipelotrichia	Erysipelotrichales	Erysipelotrichaceae	unclassified_f__Erysipelotrichaceae	unclassified_f__Erysipelotrichaceae	OTU839	0	1	0	5
Firmicutes	Clostridia	Clostridiales	Christensenellaceae	Christensenellaceae_R-7_group	bacterium_YE57	OTU754	1	0	14	0
Firmicutes	Clostridia	Clostridiales	Ruminococcaceae	Ruminiclostridium_9	unclassified_g__Ruminiclostridium_9	OTU500	0	3	263	49
Firmicutes	Clostridia	Clostridiales	Ruminococcaceae	Faecalibacterium	unclassified_g__Faecalibacterium	OTU846	9	0	4	9
Tenericutes	Mollicutes	Mollicutes_RF9	norank_o__Mollicutes	norank_o__Mollicutes_RF9	unclassified_g__norank_o__Mollicutes_RF9	OTU610	0	1	2	18
Firmicutes	Clostridia	Clostridiales	Ruminococcaceae	norank_f__Ruminococcaceae	unclassified_g__norank_f__Ruminococcaceae	OTU132	26	0	0	0
Firmicutes	Clostridia	Clostridiales	Ruminococcaceae	Ruminococcus_1	uncultured_bacterium_g__Ruminococcus_1	OTU639	0	0	0	0
Firmicutes	Clostridia	Clostridiales	Peptococcaceae	unclassified_f__Peptococcaceae	unclassified_f__Peptococcaceae	OTU483	7	2	5	4
Tenericutes	Mollicutes	Mollicutes_RF9	norank_o__Mollicutes	norank_o__Mollicutes_RF9	uncultured_bacterium_g__norank_o__Mollicutes_RF9	OTU631	0	0	5	10
Firmicutes	Clostridia	Clostridiales	Ruminococcaceae	Caproiciproducens	unclassified_g__Caproiciproducens	OTU824	0	62	1	0
Firmicutes	Clostridia	Clostridiales	Clostridiales_vadinBB6	norank_f__Clostridiales_vadinBB60_	uncultured_bacterium_g__norank_f__Clostridiales_vadinBB60_group	OTU757	0	0	23	0

Tenericutes	Mollicutes	Mollicutes_RF9	norank_o__Mollicutes	norank_o__Mollicutes_RF9	uncultured_bacterium_g__norank_o__Mollicutes_RF9	OTU792	0	31	10	0
Firmicutes	Clostridia	Clostridiales	Lachnospiraceae	Coprococcus_1	uncultured_bacterium_g__Coprococcus_1	OTU629	1	1	0	2
Firmicutes	Clostridia	Clostridiales	Peptococcaceae	Peptococcus	uncultured_organism_g__Peptococcus	OTU665	0	3	1	3
Firmicutes	Clostridia	Clostridiales	Ruminococcaceae	Ruminococcaceae_UCG-014	unclassified_g__Ruminococcaceae_UCG-014	OTU532	0	0	3	0
Firmicutes	Clostridia	Clostridiales	Ruminococcaceae	norank_f__Ruminococcaceae	unclassified_g__norank_f__Ruminococcaceae	OTU671	1	3	6	10
Firmicutes	Clostridia	Clostridiales	Ruminococcaceae	Ruminococcus_1	uncultured_organism_g__Ruminococcus_1	OTU56	3	4	2	11
Firmicutes	Clostridia	Clostridiales	Clostridiales_vadinBB6	norank_f__Clostridiales_vadinBB60_	uncultured_bacterium_g__norank_f__Clostridiales_vadinBB60_group	OTU714	1	7	5	11
Firmicutes	Clostridia	Clostridiales	Lachnospiraceae	unclassified_f__Lachnospiraceae	unclassified_f__Lachnospiraceae	OTU726	0	3	7	11
Firmicutes	Clostridia	Clostridiales	Ruminococcaceae	norank_f__Ruminococcaceae	Ruminococcaceae_bacterium_GD7	OTU72	0	3	4	0
Firmicutes	Erysipelotrichia	Erysipelotrichales	Erysipelotrichaceae	Holdemania	unclassified_g__Holdemania	OTU702	1	15	4	4
Firmicutes	Clostridia	Clostridiales	Ruminococcaceae	[Eubacterium]_coprostanoligenes_g	unclassified_g__[Eubacterium]_coprostanoligenes_group	OTU747	0	0	0	0
Firmicutes	Clostridia	Clostridiales	Family_XIII	Family_XIII_AD3011_group	uncultured_bacterium_g__Family_XIII_AD3011_group	OTU579	19	4	5	5
Tenericutes	Mollicutes	Mollicutes_RF9	norank_o__Mollicutes	norank_o__Mollicutes_RF9	uncultured_bacterium_g__norank_o__Mollicutes_RF9	OTU756	0	40	2	2
Actinobacteria	Actinobacteria	Coriobacteriales	Coriobacteriaceae	unclassified_f__Coriobacteriaceae	unclassified_f__Coriobacteriaceae	OTU4	5	29	0	1
Firmicutes	Clostridia	Clostridiales	Ruminococcaceae	Candidatus_Soleaferrea	uncultured_organism_g__Candidatus_Soleaferrea	OTU492	2	26	9	8
Firmicutes	Clostridia	Clostridiales	Ruminococcaceae	[Eubacterium]_coprostanoligenes_g	unclassified_g__[Eubacterium]_coprostanoligenes_group	OTU656	3	2	4	10
Firmicutes	Erysipelotrichia	Erysipelotrichales	Erysipelotrichaceae	Erysipelatoclostridium	unclassified_g__Erysipelatoclostridium	OTU506	0	0	113	0
Firmicutes	Clostridia	Clostridiales	Ruminococcaceae	Anaerotruncus	uncultured_bacterium_g__Anaerotruncus	OTU42	2	4	4	18
Firmicutes	Clostridia	Clostridiales	Lachnospiraceae	unclassified_f__Lachnospiraceae	unclassified_f__Lachnospiraceae	OTU509	2	17	62	3
Firmicutes	Clostridia	Clostridiales	Lachnospiraceae	Tyzzerella	uncultured_bacterium_g__Tyzzerella	OTU777	0	4	5	11
Firmicutes	Clostridia	Clostridiales	Clostridiales_vadinBB6	norank_f__Clostridiales_vadinBB60_	gut_metagenome_g__norank_f__Clostridiales_vadinBB60_group	OTU19	0	1	0	0
Firmicutes	Clostridia	Clostridiales	Ruminococcaceae	Ruminococcus_2	unclassified_g__Ruminococcus_2	OTU549	0	0	5	65
Firmicutes	Clostridia	Clostridiales	Ruminococcaceae	Anaerofilum	uncultured_bacterium_g__Anaerofilum	OTU677	0	3	3	0
Firmicutes	Clostridia	Clostridiales	Ruminococcaceae	Ruminococcaceae_UCG-014	unclassified_g__Ruminococcaceae_UCG-014	OTU487	1	40	4	2
Firmicutes	Clostridia	Clostridiales	Ruminococcaceae	Ruminococcaceae_UCG-013	unclassified_g__Ruminococcaceae_UCG-013	OTU145	0	3	0	1
Bacteroidetes	Bacteroidia	Bacteroidales	Rikenellaceae	Alistipes	unclassified_g__Alistipes	OTU667	4	1	31	5
Firmicutes	Clostridia	Clostridiales	Lachnospiraceae	unclassified_f__Lachnospiraceae	unclassified_f__Lachnospiraceae	OTU771	11	0	3	3
Firmicutes	Clostridia	Clostridiales	Ruminococcaceae	Ruminococcaceae_UCG-009	uncultured_bacterium_g__Ruminococcaceae_UCG-009	OTU750	1	1	8	0
Tenericutes	Mollicutes	Mollicutes_RF9	norank_o__Mollicutes	norank_o__Mollicutes_RF9	uncultured_bacterium_g__norank_o__Mollicutes_RF9	OTU543	0	5	3	13
Firmicutes	Clostridia	Clostridiales	unclassified_o__Clostr	unclassified_o__Clostridiales	unclassified_o__Clostridiales	OTU535	6	2	5	0
Tenericutes	Mollicutes	Mollicutes_RF9	norank_o__Mollicutes	norank_o__Mollicutes_RF9	uncultured_bacterium_g__norank_o__Mollicutes_RF9	OTU153	0	5	14	0
Firmicutes	Clostridia	Clostridiales	Clostridiales_vadinBB6	norank_f__Clostridiales_vadinBB60_	uncultured_bacterium_g__norank_f__Clostridiales_vadinBB60_group	OTU409	0	9	6	9
Firmicutes	Clostridia	Clostridiales	Ruminococcaceae	Oscillibacter	unclassified_g__Oscillibacter	OTU609	0	0	2	5
Firmicutes	Clostridia	Clostridiales	Ruminococcaceae	Butyricicoccus	unclassified_g__Butyricicoccus	OTU411	35	1	0	9
Firmicutes	Clostridia	Clostridiales	unclassified_o__Clostridiales	unclassified_o__Clostridiales	unclassified_o__Clostridiales	OTU34	4	6	16	8
Firmicutes	Clostridia	Clostridiales	Clostridiales_vadinBB6	norank_f__Clostridiales_vadinBB60_	uncultured_rumen_bacterium_g__norank_f__Clostridiales_vadinBB60_group	OTU517	0	12	10	0
Firmicutes	Clostridia	Clostridiales	Ruminococcaceae	Ruminococcaceae_UCG-014	uncultured_bacterium_g__Ruminococcaceae_UCG-014	OTU850	0	13	8	0
Firmicutes	Clostridia	Clostridiales	Ruminococcaceae	Ruminiclostridium_5	uncultured_organism_g__Ruminiclostridium_5	OTU541	16	3	14	0
Firmicutes	Clostridia	Clostridiales	Lachnospiraceae	Tyzzerella_3	uncultured_bacterium_g__Tyzzerella_3	OTU635	3	0	0	6
Firmicutes	Clostridia	Clostridiales	Eubacteriaceae	Anaerofustis	Anaerofustis_sp._Marseille-P2441	OTU136	3	0	3	9
Firmicutes	Clostridia	Clostridiales	Ruminococcaceae	Ruminococcaceae_UCG-013	unclassified_g__Ruminococcaceae_UCG-013	OTU76	0	15	0	2
Firmicutes	Clostridia	Clostridiales	Ruminococcaceae	Anaerotruncus	unclassified_g__Anaerotruncus	OTU542	8	4	1	3
Firmicutes	Clostridia	Clostridiales	Ruminococcaceae	unclassified_f__Ruminococcaceae	unclassified_f__Ruminococcaceae	OTU831	27	10	0	3
Firmicutes	Clostridia	Clostridiales	Ruminococcaceae	Ruminococcaceae_NK4A214_group	unclassified_g__Ruminococcaceae_NK4A214_group	OTU168	0	3	3	11
Firmicutes	Clostridia	Clostridiales	Ruminococcaceae	Ruminococcaceae_UCG-009	unclassified_g__Ruminococcaceae_UCG-009	OTU71	0	0	2	1
Firmicutes	Clostridia	Clostridiales	Ruminococcaceae	Ruminococcaceae_UCG-013	uncultured_organism_g__Ruminococcaceae_UCG-013	OTU204	0	5	0	21
Actinobacteria	Actinobacteria	Coriobacteriales	Coriobacteriaceae	Enterorhabdus	uncultured_bacterium_g__Enterorhabdus	OTU221	6	26	1	4
Tenericutes	Mollicutes	Mollicutes_RF9	norank_o__Mollicutes	norank_o__Mollicutes_RF9	unclassified_g__norank_o__Mollicutes_RF9	OTU788	0	0	3	0
Firmicutes	Clostridia	Clostridiales	Ruminococcaceae	Ruminococcaceae_UCG-010	uncultured_organism_g__Ruminococcaceae_UCG-010	OTU20	2	5	1	2
Firmicutes	Clostridia	Clostridiales	Ruminococcaceae	Anaerotruncus	uncultured_bacterium_g__Anaerotruncus	OTU582	29	0	0	0
Firmicutes	Clostridia	Clostridiales	Family_XIII	Family_XIII_AD3011_group	uncultured_bacterium_g__Family_XIII_AD3011_group	OTU634	11	9	1	5
Firmicutes	Clostridia	Clostridiales	Ruminococcaceae	Anaerofilum	uncultured_bacterium_g__Anaerofilum	OTU832	0	0	0	1
Firmicutes	Clostridia	Clostridiales	Clostridiales_vadinBB6	norank_f__Clostridiales_vadinBB60_	uncultured_bacterium_g__norank_f__Clostridiales_vadinBB60_group	OTU38	0	0	0	0
Firmicutes	Clostridia	Clostridiales	unclassified_o__Clostr	unclassified_o__Clostridiales	unclassified_o__Clostridiales	OTU672	0	0	2	0
Firmicutes	Clostridia	Clostridiales	Ruminococcaceae	unclassified_f__Ruminococcaceae	unclassified_f__Ruminococcaceae	OTU661	7	6	3	1
Firmicutes	Clostridia	Clostridiales	Ruminococcaceae	unclassified_f__Ruminococcaceae	unclassified_f__Ruminococcaceae	OTU169	0	2	0	0
Tenericutes	Mollicutes	Mollicutes_RF9	norank_o__Mollicutes	norank_o__Mollicutes_RF9	uncultured_bacterium_g__norank_o__Mollicutes_RF9	OTU820	0	2	0	0
Firmicutes	Clostridia	Clostridiales	Eubacteriaceae	Eubacterium	Eubacterium_limosum	OTU337	4	102	0	0
Tenericutes	Mollicutes	Mollicutes_RF9	norank_o__Mollicutes	norank_o__Mollicutes_RF9	uncultured_bacterium_g__norank_o__Mollicutes_RF9	OTU584	6	1	1	5
Firmicutes	Clostridia	Clostridiales	Ruminococcaceae	Anaerofilum	uncultured_bacterium_g__Anaerofilum	OTU643	4	4	0	0
Firmicutes	Clostridia	Clostridiales	Clostridiales_vadinBB6	norank_f__Clostridiales_vadinBB60_	uncultured_bacterium_g__norank_f__Clostridiales_vadinBB60_group	OTU35	0	3	2	7
Firmicutes	Clostridia	Clostridiales	Lachnospiraceae	Coprococcus_1	uncultured_bacterium_g__Coprococcus_1	OTU811	15	1	0	8
Firmicutes	Clostridia	Clostridiales	Ruminococcaceae	Ruminococcaceae_UCG-014	unclassified_g__Ruminococcaceae_UCG-014	OTU87	0	20	6	0
Firmicutes	Clostridia	Clostridiales	Ruminococcaceae	[Eubacterium]_coprostanoligenes_g	unclassified_g__[Eubacterium]_coprostanoligenes_group	OTU751	0	14	0	14
Firmicutes	Clostridia	Clostridiales	Clostridiales_vadinBB6	norank_f__Clostridiales_vadinBB60_	uncultured_bacterium_g__norank_f__Clostridiales_vadinBB60_group	OTU694	0	0	2	0
Firmicutes	Clostridia	Clostridiales	Clostridiales_vadinBB6	norank_f__Clostridiales_vadinBB60_	uncultured_Thermoanaerobacterales_bacterium_g__norank_f__Clostridiales_vadinBB60_gr	OTU224	0	4	0	0
Firmicutes	Clostridia	Clostridiales	Lachnospiraceae	unclassified_f__Lachnospiraceae	unclassified_f__Lachnospiraceae	OTU655	4	0	4	5
Firmicutes	Clostridia	Clostridiales	Ruminococcaceae	Oscillospira	uncultured_bacterium_g__Oscillospira	OTU574	0	6	3	0
Firmicutes	Clostridia	Clostridiales	Clostridiales_vadinBB6	norank_f__Clostridiales_vadinBB60_	unclassified_g__norank_f__Clostridiales_vadinBB60_group	OTU2	0	0	0	0
Firmicutes	Clostridia	Clostridiales	Clostridiales_vadinBB6	norank_f__Clostridiales_vadinBB60_	unclassified_g__norank_f__Clostridiales_vadinBB60_group	OTU778	0	0	0	0
Firmicutes	Clostridia	Clostridiales	Ruminococcaceae	Ruminococcaceae_UCG-013	unclassified_g__Ruminococcaceae_UCG-013	OTU676	0	3	0	2
Tenericutes	Mollicutes	Mollicutes_RF9	norank_o__Mollicutes	norank_o__Mollicutes_RF9	unclassified_g__norank_o__Mollicutes_RF9	OTU626	0	8	6	5
Firmicutes	Clostridia	Clostridiales	Ruminococcaceae	Ruminococcaceae_UCG-010	unclassified_g__Ruminococcaceae_UCG-010	OTU61	2	5	4	0
Firmicutes	Clostridia	Clostridiales	Ruminococcaceae	norank_f__Ruminococcaceae	unclassified_g__norank_f__Ruminococcaceae	OTU516	0	4	0	3
Firmicutes	Clostridia	Clostridiales	Ruminococcaceae	unclassified_f__Ruminococcaceae	unclassified_f__Ruminococcaceae	OTU228	3	0	1	1
Firmicutes	Clostridia	Clostridiales	Ruminococcaceae	Ruminococcaceae_UCG-010	uncultured_bacterium_g__Ruminococcaceae_UCG-010	OTU203	0	0	0	0
Firmicutes	Clostridia	Clostridiales	Ruminococcaceae	Anaerotruncus	uncultured_bacterium_g__Anaerotruncus	OTU853	0	15	31	1
Firmicutes	Clostridia	Thermoanaerobacte	Thermoanaerobactera	Gelria	uncultured_bacterium_g__Gelria	OTU16	0	3	3	12
Firmicutes	Clostridia	Clostridiales	Clostridiales_vadinBB6	norank_f__Clostridiales_vadinBB60_	uncultured_bacterium_g__norank_f__Clostridiales_vadinBB60_group	OTU143	0	6	2	3
Firmicutes	Clostridia	Clostridiales	Family_XIII	unclassified_f__Family_XIII	unclassified_f__Family_XIII	OTU537	2	17	17	0
Firmicutes	Clostridia	Clostridiales	Family_XIII	[Eubacterium]_brachy_group	uncultured_bacterium_g__[Eubacterium]_brachy_group	OTU69	11	2	3	1
Firmicutes	Clostridia	Clostridiales	Ruminococcaceae	unclassified_f__Ruminococcaceae	unclassified_f__Ruminococcaceae	OTU14	0	0	0	5
Firmicutes	Clostridia	Clostridiales	Ruminococcaceae	Intestinimonas	uncultured_bacterium_g__Intestinimonas	OTU615	5	0	7	4
Firmicutes	Clostridia	Clostridiales	Lachnospiraceae	unclassified_f__Lachnospiraceae	unclassified_f__Lachnospiraceae	OTU118	14	1	1	5
Firmicutes	Clostridia	Clostridiales	Ruminococcaceae	Anaerotruncus	unclassified_g__Anaerotruncus	OTU505	0	2	42	14
Firmicutes	Bacilli	Lactobacillales	Lactobacillaceae	Lactobacillus	uncultured_bacterium_g__Lactobacillus	OTU637	0	3	8	5
Firmicutes	Clostridia	Clostridiales	Ruminococcaceae	Ruminococcaceae_V9D2013_group	uncultured_bacterium_g__Ruminococcaceae_V9D2013_group	OTU652	2	10	4	4
Firmicutes	Erysipelotrichia	Erysipelotrichales	Erysipelotrichaceae	norank_f__Erysipelotrichaceae	uncultured_Erysipelotrichales_bacterium_g__norank	OTU323	0	0	0	0
Firmicutes	Clostridia	Clostridiales	Ruminococcaceae	Papillibacter	uncultured_bacterium_g__Papillibacter	OTU715	1	9	3	0
Tenericutes	Mollicutes	Mollicutes_RF9	norank_o__Mollicutes	norank_o__Mollicutes_RF9	unclassified_g__norank_o__Mollicutes_RF9	OTU729	0	5	0	0
Cyanobacteria	Cyanobacteria	Gastranaerophilales	norank_o__Gastranae	norank_o__Gastranaerophilales	uncultured_bacterium_g__norank_o__Gastranaerophilales	OTU94	0	29	0	0
Firmicutes	Clostridia	Clostridiales	Ruminococcaceae	Ruminococcaceae_UCG-013	uncultured_bacterium_g__Ruminococcaceae_UCG-013	OTU780	0	22	0	0
Firmicutes	Clostridia	Clostridiales	Ruminococcaceae	Anaerotruncus	unclassified_g__Anaerotruncus	OTU723	3	0	4	2
Firmicutes	Clostridia	Clostridiales	Ruminococcaceae	Anaerotruncus	unclassified_g__Anaerotruncus	OTU101	8	2	1	0
Tenericutes	Mollicutes	Mollicutes_RF9	norank_o__Mollicutes	norank_o__Mollicutes_RF9	unclassified_g__norank_o__Mollicutes_RF9	OTU117	0	8	9	1
Firmicutes	Clostridia	Clostridiales	Ruminococcaceae	unclassified_f__Ruminococcaceae	unclassified_f__Ruminococcaceae	OTU93	0	0	1	1
Firmicutes	Clostridia	Clostridiales	Lachnospiraceae	unclassified_f__Lachnospiraceae	unclassified_f__Lachnospiraceae	OTU759	11	1	9	1
Firmicutes	Clostridia	Clostridiales	Ruminococcaceae	Ruminococcaceae_UCG-014	unclassified_g__Ruminococcaceae_UCG-014	OTU95	0	0	0	0
Firmicutes	Erysipelotrichia	Erysipelotrichales	Erysipelotrichaceae	Dielma	unclassified_g__Dielma	OTU173	0	0	0	0
Firmicutes	Clostridia	Clostridiales	Ruminococcaceae	Anaerotruncus	unclassified_g__Anaerotruncus	OTU124	2	5	1	4
Firmicutes	Clostridia	Clostridiales	Ruminococcaceae	norank_f__Ruminococcaceae	unclassified_g__norank_f__Ruminococcaceae	OTU134	7	0	1	2
Firmicutes	Clostridia	Clostridiales	Lachnospiraceae	unclassified_f__Lachnospiraceae	unclassified_f__Lachnospiraceae	OTU510	2	4	5	9
Actinobacteria	Actinobacteria	Coriobacteriales	Coriobacteriaceae	Gordonibacter	unclassified_g__Gordonibacter	OTU461	0	1	0	2
Proteobacteria	Betaproteobacteria	Burkholderiales	Burkholderiaceae	Burkholderia-Paraburkholderia	unclassified_g__Burkholderia-Paraburkholderia	OTU435	0	1	0	0
Firmicutes	Clostridia	Clostridiales	Family_XIII	Family_XIII_AD3011_group	uncultured_bacterium_g__Family_XIII_AD3011_group	OTU648	0	0	0	0
Firmicutes	Clostridia	Clostridiales	Ruminococcaceae	unclassified_f__Ruminococcaceae	unclassified_f__Ruminococcaceae	OTU722	2	4	0	2
Firmicutes	Clostridia	Clostridiales	Clostridiales_vadinBB6	norank_f__Clostridiales_vadinBB60_	uncultured_bacterium_g__norank_f__Clostridiales_vadinBB60_group	OTU740	0	0	2	0
Bacteroidetes	Bacteroidia	Bacteroidales	Bacteroidales_S24-7_g	norank_f__Bacteroidales_S24-7_gro	uncultured_bacterium_g__norank_f__Bacteroidales_S24-7_group	OTU322	0	0	0	0
Firmicutes	Clostridia	Clostridiales	Ruminococcaceae	unclassified_f__Ruminococcaceae	unclassified_f__Ruminococcaceae	OTU538	0	0	12	6
Firmicutes	Clostridia	Clostridiales	Lachnospiraceae	unclassified_f__Lachnospiraceae	unclassified_f__Lachnospiraceae	OTU255	0	2	12	2
Firmicutes	Clostridia	Clostridiales	Christensenellaceae	Christensenellaceae_R-7_group	unclassified_g__Christensenellaceae_R-7_group	OTU106	0	0	0	0

Firmicutes	Clostridia	Clostridiales	Ruminococcaceae	[Eubacterium]_coprostanoligenes_g	uncultured_bacterium_g__[Eubacterium]_coprostanoligenes_group	OTU129	0	0	0	2
Firmicutes	Erysipelotrichia	Erysipelotrichales	Erysipelotrichaceae	Erysipelatoclostridium	Clostridiales_bacterium_CHKCI006	OTU232	0	0	39	7
Firmicutes	Clostridia	Clostridiales	Ruminococcaceae	unclassified_f__Ruminococcaceae	unclassified_f__Ruminococcaceae	OTU518	3	3	6	0
Firmicutes	Clostridia	Clostridiales	Ruminococcaceae	Ruminococcaceae_UCG-013	uncultured_Clostridiaceae_bacterium_g__Ruminococcaceae_UCG-013	OTU675	0	60	0	2
Firmicutes	Clostridia	Clostridiales	Christensenellaceae	Christensenellaceae_R-7_group	human_gut_metagenome_g__Christensenellaceae_R-7_group	OTU223	0	0	0	0
Firmicutes	Clostridia	Clostridiales	Ruminococcaceae	Faecalibacterium	uncultured_bacterium_g__Faecalibacterium	OTU41	0	6	9	3
Firmicutes	Clostridia	Clostridiales	Ruminococcaceae	Anaerotruncus	unclassified_g__Anaerotruncus	OTU647	0	0	1	0
Firmicutes	Erysipelotrichia	Erysipelotrichales	Erysipelotrichaceae	Turicibacter	uncultured_bacterium_g__Turicibacter	OTU598	0	0	0	0
Firmicutes	Clostridia	Clostridiales	Ruminococcaceae	Subdoligranulum	unclassified_g__Subdoligranulum	OTU188	9	0	0	2
Firmicutes	Clostridia	Clostridiales	Ruminococcaceae	Anaerotruncus	uncultured_Clostridiaceae_bacterium_g__Anaerotruncus	OTU235	0	2	2	7
Firmicutes	Clostridia	Clostridiales	Family_XIII	unclassified_f__Family_XIII	unclassified_f__Family_XIII	OTU236	0	0	3	4
Firmicutes	Clostridia	Clostridiales	Ruminococcaceae	Ruminococcaceae_UCG-014	unidentified_rumen_bacterium_JW32	OTU21	0	0	0	12
Firmicutes	Clostridia	Clostridiales	Ruminococcaceae	Papillibacter	unclassified_g__Papillibacter	OTU521	0	0	0	0
Firmicutes	Clostridia	Clostridiales	Ruminococcaceae	unclassified_f__Ruminococcaceae	unclassified_f__Ruminococcaceae	OTU653	0	8	0	3
Firmicutes	Clostridia	Clostridiales	Peptococcaceae	norank_f__Peptococcaceae	uncultured_bacterium_g__norank_f__Peptococcaceae	OTU605	0	1	2	0
Firmicutes	Bacilli	Lactobacillales	Lactobacillaceae	Lactobacillus	Lactobacillus_ingluviei	OTU182	0	0	0	0
Firmicutes	Clostridia	Clostridiales	Ruminococcaceae	[Eubacterium]_coprostanoligenes_g	unclassified_g__[Eubacterium]_coprostanoligenes_group	OTU333	0	0	14	8
Firmicutes	Clostridia	Clostridiales	unclassified_o__Clostr	unclassified_o__Clostridiales	unclassified_o__Clostridiales	OTU472	8	0	2	3
Firmicutes	Clostridia	Clostridiales	Ruminococcaceae	[Eubacterium]_coprostanoligenes_g	unclassified_g__[Eubacterium]_coprostanoligenes_group	OTU730	1	0	0	0
Firmicutes	Clostridia	Clostridiales	Peptococcaceae	norank_f__Peptococcaceae	unclassified_g__norank_f__Peptococcaceae	OTU172	0	1	0	0
Firmicutes	Clostridia	Clostridiales	Ruminococcaceae	Ruminococcaceae_UCG-013	uncultured_bacterium_g__Ruminococcaceae_UCG-013	OTU658	0	0	0	0
Proteobacteria	Gammaproteobacteria	Enterobacteriales	Enterobacteriaceae	Proteus	Proteus_mirabilis	OTU785	6	4	0	0
Firmicutes	Clostridia	Clostridiales	Ruminococcaceae	norank_f__Ruminococcaceae	unclassified_g__norank_f__Ruminococcaceae	OTU744	1	0	0	0
Firmicutes	Clostridia	Clostridiales	Lachnospiraceae	Tyzzerella_3	unclassified_g__Tyzzerella_3	OTU841	0	0	5	1
Bacteroidetes	Bacteroidia	Bacteroidales	Bacteroidales_S24-7_g	norank_f__Bacteroidales_S24-7_grou	uncultured_bacterium_g__norank_f__Bacteroidales_S24-7_group	OTU360	0	0	0	0
Firmicutes	Clostridia	Clostridiales	Ruminococcaceae	unclassified_f__Ruminococcaceae	unclassified_f__Ruminococcaceae	OTU737	0	2	0	7
Firmicutes	Clostridia	Clostridiales	Ruminococcaceae	Anaerotruncus	unclassified_g__Anaerotruncus	OTU752	4	1	2	1
Firmicutes	Clostridia	Clostridiales	Lachnospiraceae	unclassified_f__Lachnospiraceae	unclassified_f__Lachnospiraceae	OTU63	6	0	2	2
Firmicutes	Clostridia	Clostridiales	Ruminococcaceae	Caproiciproducens	unclassified_g__Caproiciproducens	OTU452	3	1	1	5
Firmicutes	Clostridia	Clostridiales	Ruminococcaceae	Butyricicoccus	unclassified_g__Butyricicoccus	OTU475	52	1	1	0
Proteobacteria	Betaproteobacteria	Burkholderiales	Oxalobacteraceae	Herminiimonas	uncultured_bacterium_g__Herminiimonas	OTU804	1	5	0	2
Tenericutes	Mollicutes	Mollicutes_RF9	norank_o__Mollicutes	norank_o__Mollicutes_RF9	unclassified_g__norank_o__Mollicutes_RF9	OTU630	0	0	0	2
Firmicutes	Clostridia	Clostridiales	Ruminococcaceae	Ruminococcaceae_UCG-013	unclassified_g__Ruminococcaceae_UCG-013	OTU680	0	0	1	4
Tenericutes	Mollicutes	Mollicutes_RF9	norank_o__Mollicutes	norank_o__Mollicutes_RF9	gut_metagenome_g__norank_o__Mollicutes_RF9	OTU773	0	1	0	0
Firmicutes	Clostridia	Clostridiales	Peptococcaceae	norank_f__Peptococcaceae	uncultured_bacterium_g__norank_f__Peptococcaceae	OTU624	0	0	1	2
Firmicutes	Clostridia	Clostridiales	Ruminococcaceae	Ruminiclostridium	unclassified_g__Ruminiclostridium	OTU154	0	0	0	5
Bacteroidetes	Bacteroidia	Bacteroidales	Bacteroidales_S24-7_g	norank_f__Bacteroidales_S24-7_grou	unclassified_g__norank_f__Bacteroidales_S24-7_group	OTU184	0	0	1	0
Firmicutes	Clostridia	Clostridiales	Ruminococcaceae	Ruminococcaceae_UCG-014	unclassified_g__Ruminococcaceae_UCG-014	OTU524	3	1	0	0
Firmicutes	Clostridia	Clostridiales	Peptococcaceae	norank_f__Peptococcaceae	uncultured_bacterium_g__norank_f__Peptococcaceae	OTU115	0	0	0	0
Firmicutes	Clostridia	Clostridiales	Lachnospiraceae	unclassified_f__Lachnospiraceae	unclassified_f__Lachnospiraceae	OTU127	0	3	0	0
Firmicutes	Clostridia	Clostridiales	Lachnospiraceae	unclassified_f__Lachnospiraceae	unclassified_f__Lachnospiraceae	OTU664	2	2	2	1
Firmicutes	Clostridia	Clostridiales	Lachnospiraceae	unclassified_f__Lachnospiraceae	unclassified_f__Lachnospiraceae	OTU465	2	0	1	8
Firmicutes	Clostridia	Clostridiales	Ruminococcaceae	Ruminococcaceae_UCG-005	uncultured_Clostridiales_bacterium_g__Ruminococcaceae_UCG-005	OTU689	0	1	2	4
Tenericutes	Mollicutes	Mollicutes_RF9	norank_o__Mollicutes	norank_o__Mollicutes_RF9	uncultured_bacterium_g__norank_o__Mollicutes_RF9	OTU52	0	0	0	0
Firmicutes	Clostridia	Clostridiales	Ruminococcaceae	[Eubacterium]_coprostanoligenes_g	unclassified_g__[Eubacterium]_coprostanoligenes_group	OTU74	1	1	1	2
Firmicutes	Clostridia	Clostridiales	Lachnospiraceae	Coprococcus_1	unclassified_g__Coprococcus_1	OTU659	3	1	1	0
Firmicutes	Clostridia	Clostridiales	Lachnospiraceae	unclassified_f__Lachnospiraceae	unclassified_f__Lachnospiraceae	OTU688	0	0	4	1
Firmicutes	Clostridia	Clostridiales	Ruminococcaceae	unclassified_f__Ruminococcaceae	unclassified_f__Ruminococcaceae	OTU732	0	3	28	12
Firmicutes	Clostridia	Clostridiales	Christensenellaceae	Christensenellaceae_R-7_group	uncultured_organism_g__Christensenellaceae_R-7_group	OTU684	1	0	3	0
Bacteroidetes	Bacteroidia	Bacteroidales	Porphyromonadaceae	Odoribacter	uncultured_bacterium_g__Odoribacter	OTU327	0	0	0	0
Firmicutes	Clostridia	Clostridiales	Clostridiales_vadinBB6	norank_f__Clostridiales_vadinBB60_	uncultured_bacterium_g__norank_f__Clostridiales_vadinBB60_group	OTU325	0	0	17	0
Firmicutes	Clostridia	Clostridiales	Lachnospiraceae	unclassified_f__Lachnospiraceae	unclassified_f__Lachnospiraceae	OTU551	0	11	4	16
Firmicutes	Bacilli	Lactobacillales	Enterococcaceae	Enterococcus	unclassified_g__Enterococcus	OTU237	0	0	0	0
Bacteroidetes	Bacteroidia	Bacteroidales	Prevotellaceae	Alloprevotella	gut_metagenome_g__Alloprevotella	OTU312	0	0	0	0
Firmicutes	Clostridia	Clostridiales	Ruminococcaceae	unclassified_f__Ruminococcaceae	unclassified_f__Ruminococcaceae	OTU855	2	0	0	1
Actinobacteria	Actinobacteria	Bifidobacteriales	Bifidobacteriaceae	Bifidobacterium	Bifidobacterium_pseudolongum_PV8-2	OTU468	0	0	0	0
Firmicutes	Clostridia	Clostridiales	Christensenellaceae	norank_f__Christensenellaceae	unclassified_g__norank_f__Christensenellaceae	OTU529	3	0	0	3
Tenericutes	Mollicutes	Mollicutes_RF9	norank_o__Mollicutes	norank_o__Mollicutes_RF9	unclassified_g__norank_o__Mollicutes_RF9	OTU764	0	1	1	0
Firmicutes	Clostridia	Clostridiales	Ruminococcaceae	norank_f__Ruminococcaceae	unclassified_g__norank_f__Ruminococcaceae	OTU805	0	1	3	1
Firmicutes	Clostridia	Clostridiales	Ruminococcaceae	Faecalibacterium	uncultured_bacterium_g__Faecalibacterium	OTU60	1	0	0	1
Firmicutes	Clostridia	Clostridiales	Ruminococcaceae	unclassified_f__Ruminococcaceae	unclassified_f__Ruminococcaceae	OTU530	0	0	1	0
Firmicutes	Clostridia	Clostridiales	Peptococcaceae	norank_f__Peptococcaceae	uncultured_bacterium_g__norank_f__Peptococcaceae	OTU619	0	0	1	0
Firmicutes	Clostridia	Clostridiales	Ruminococcaceae	unclassified_f__Ruminococcaceae	unclassified_f__Ruminococcaceae	OTU471	2	0	0	2
Firmicutes	Clostridia	Clostridiales	Ruminococcaceae	Candidatus_Soleaferrea	uncultured_bacterium_g__Candidatus_Soleaferrea	OTU284	2	1	2	0
Firmicutes	Clostridia	Clostridiales	Ruminococcaceae	Anaerotruncus	unclassified_g__Anaerotruncus	OTU508	0	0	18	1
Firmicutes	Clostridia	Clostridiales	Ruminococcaceae	Anaerotruncus	uncultured_bacterium_g__Anaerotruncus	OTU704	5	3	2	1
Tenericutes	Mollicutes	Mollicutes_RF9	norank_o__Mollicutes	norank_o__Mollicutes_RF9	uncultured_bacterium_g__norank_o__Mollicutes_RF9	OTU783	0	0	4	3
Firmicutes	Clostridia	Clostridiales	Eubacteriaceae	Anaerofustis	uncultured_bacterium_g__Anaerofustis	OTU111	0	0	0	1
Firmicutes	Clostridia	Clostridiales	Clostridiales_vadinBB6	norank_f__Clostridiales_vadinBB60_	unclassified_g__norank_f__Clostridiales_vadinBB60_group	OTU645	0	3	1	1
Firmicutes	Clostridia	Clostridiales	Peptococcaceae	norank_f__Peptococcaceae	uncultured_bacterium_g__norank_f__Peptococcaceae	OTU27	0	0	0	0
Firmicutes	Clostridia	Clostridiales	Ruminococcaceae	Anaerotruncus	unclassified_g__Anaerotruncus	OTU809	0	0	0	3
Bacteroidetes	Bacteroidia	Bacteroidales	Rikenellaceae	Rikenellaceae_RC9_gut_group	unclassified_g__Rikenellaceae_RC9_gut_group	OTU321	0	0	0	0
Firmicutes	Clostridia	Clostridiales	Ruminococcaceae	Ruminococcaceae_UCG-005	unclassified_g__Ruminococcaceae_UCG-005	OTU482	4	2	5	1
Tenericutes	Mollicutes	Mollicutes_RF9	norank_o__Mollicutes	norank_o__Mollicutes_RF9	uncultured_Erysipelotrichaceae_bacterium_g__norank_o__Mollicutes_RF9	OTU558	0	0	0	0
Firmicutes	Clostridia	Clostridiales	Clostridiaceae_1	Candidatus_Arthromitus	Candidatus_Arthromitus_sp._SFB-mouse-Japan	OTU162	0	0	0	0
Firmicutes	Clostridia	Clostridiales	Ruminococcaceae	Subdoligranulum	unclassified_g__Subdoligranulum	OTU810	8	1	1	0
Firmicutes	Clostridia	Clostridiales	Ruminococcaceae	norank_f__Ruminococcaceae	unclassified_g__norank_f__Ruminococcaceae	OTU666	3	12	1	10
Firmicutes	Clostridia	Clostridiales	Ruminococcaceae	unclassified_f__Ruminococcaceae	unclassified_f__Ruminococcaceae	OTU693	0	0	0	0
Firmicutes	Clostridia	Clostridiales	Ruminococcaceae	unclassified_f__Ruminococcaceae	unclassified_f__Ruminococcaceae	OTU858	0	2	3	1
Firmicutes	Clostridia	Clostridiales	Ruminococcaceae	Ruminiclostridium_5	unclassified_g__Ruminiclostridium_5	OTU470	2	0	0	0
Firmicutes	Negativicutes	Selenomonadales	Acidaminococcaceae	Phascolarctobacterium	uncultured_organism_g__Phascolarctobacterium	OTU787	0	5	0	0
Firmicutes	Clostridia	Clostridiales	Christensenellaceae	Christensenellaceae_R-7_group	unclassified_g__Christensenellaceae_R-7_group	OTU745	0	6	0	0
Firmicutes	Clostridia	Clostridiales	Peptococcaceae	norank_f__Peptococcaceae	unclassified_g__norank_f__Peptococcaceae	OTU495	0	5	0	0
Firmicutes	Clostridia	Clostridiales	Ruminococcaceae	Ruminococcaceae_UCG-010	uncultured_bacterium_g__Ruminococcaceae_UCG-010	OTU18	0	1	0	0
Firmicutes	Clostridia	Clostridiales	Ruminococcaceae	Ruminococcaceae_UCG-013	unclassified_g__Ruminococcaceae_UCG-013	OTU89	0	0	0	0
Firmicutes	Clostridia	Clostridiales	Ruminococcaceae	Ruminiclostridium_5	unclassified_g__Ruminiclostridium_5	OTU649	1	4	1	0
Firmicutes	Clostridia	Clostridiales	Lachnospiraceae	unclassified_f__Lachnospiraceae	unclassified_f__Lachnospiraceae	OTU324	2	1	5	1
Firmicutes	Clostridia	Clostridiales	Ruminococcaceae	Ruminococcaceae_UCG-013	unclassified_g__Ruminococcaceae_UCG-013	OTU31	0	0	1	1
Firmicutes	Clostridia	Clostridiales	Ruminococcaceae	Ruminococcus_1	uncultured_organism_g__Ruminococcus_1	OTU651	0	0	0	0
Firmicutes	Clostridia	Clostridiales	Peptococcaceae	unclassified_f__Peptococcaceae	unclassified_f__Peptococcaceae	OTU768	0	1	2	1
Firmicutes	Clostridia	Clostridiales	Christensenellaceae	unclassified_f__Christensenellaceae	unclassified_f__Christensenellaceae	OTU857	0	0	1	0
Tenericutes	Mollicutes	Mollicutes_RF9	norank_o__Mollicutes	norank_o__Mollicutes_RF9	unclassified_g__norank_o__Mollicutes_RF9	OTU758	0	3	2	0
Firmicutes	Clostridia	Clostridiales	Ruminococcaceae	Candidatus_Soleaferrea	uncultured_bacterium_g__Candidatus_Soleaferrea	OTU791	1	0	1	0
unclassified_k__r	unclassified_k__norank	unclassified_k__nor	unclassified_k__noran	unclassified_k__norank	unclassified_k__norank	OTU9	2	1	2	1
Firmicutes	Clostridia	Clostridiales	Ruminococcaceae	unclassified_f__Ruminococcaceae	unclassified_f__Ruminococcaceae	OTU733	0	1	0	3
Firmicutes	Clostridia	Clostridiales	Peptococcaceae	norank_f__Peptococcaceae	unclassified_g__norank_f__Peptococcaceae	OTU90	0	2	3	0
Firmicutes	Clostridia	Clostridiales	Lachnospiraceae	unclassified_f__Lachnospiraceae	unclassified_f__Lachnospiraceae	OTU644	0	14	5	6
Firmicutes	Clostridia	Clostridiales	Ruminococcaceae	[Eubacterium]_coprostanoligenes_g	unclassified_g__[Eubacterium]_coprostanoligenes_group	OTU29	1	1	0	0
Firmicutes	Clostridia	Clostridiales	Ruminococcaceae	Ruminococcaceae_UCG-013	uncultured_Clostridiaceae_bacterium_g__Ruminococcaceae_UCG-013	OTU152	0	0	0	0
Firmicutes	Clostridia	Clostridiales	Ruminococcaceae	Faecalibacterium	unclassified_g__Faecalibacterium	OTU243	0	0	1	0
Bacteroidetes	Bacteroidia	Bacteroidales	Bacteroidales_S24-7_g	norank_f__Bacteroidales_S24-7_grou	uncultured_Bacteroidales_bacterium_g__norank_f__Bacteroidales_S24-7_group	OTU339	0	0	0	0
Tenericutes	Mollicutes	Mollicutes_RF9	norank_o__Mollicutes	norank_o__Mollicutes_RF9	unclassified_g__norank_o__Mollicutes_RF9	OTU51	0	0	1	0
Firmicutes	Clostridia	Clostridiales	Christensenellaceae	Christensenellaceae_R-7_group	uncultured_organism_g__Christensenellaceae_R-7_group	OTU332	0	0	0	0
Firmicutes	Clostridia	Clostridiales	Ruminococcaceae	Anaerotruncus	unclassified_g__Anaerotruncus	OTU194	0	0	1	1
Firmicutes	Clostridia	Clostridiales	Ruminococcaceae	Ruminococcaceae_UCG-013	uncultured_bacterium_g__Ruminococcaceae_UCG-013	OTU334	0	0	0	1
Firmicutes	Clostridia	Clostridiales	Ruminococcaceae	Ruminococcaceae_NK4A214_group	unclassified_g__Ruminococcaceae_NK4A214_group	OTU32	0	1	1	1
Firmicutes	Clostridia	Clostridiales	Christensenellaceae	Christensenellaceae_R-7_group	unclassified_g__Christensenellaceae_R-7_group	OTU105	0	2	0	1
Firmicutes	Bacilli	Bacillales	Staphylococcaceae	Staphylococcus	Staphylococcus_warneri	OTU597	0	0	0	0
Firmicutes	Bacilli	Bacillales	Staphylococcaceae	Staphylococcus	Staphylococcus_lentus_g__Staphylococcus	OTU432	0	0	0	0
Firmicutes	Clostridia	Clostridiales	Ruminococcaceae	unclassified_f__Ruminococcaceae	unclassified_f__Ruminococcaceae	OTU698	0	0	0	0
Tenericutes	Mollicutes	Mollicutes_RF9	norank_o__Mollicutes	norank_o__Mollicutes_RF9	unclassified_g__norank_o__Mollicutes_RF9	OTU781	0	0	4	0

Firmicutes	Clostridia	Clostridiales	Ruminococcaceae	Anaerotruncus	uncultured_bacterium_g__Anaerotruncus	OTU53	0	1	4	0
Actinobacteria	Actinobacteria	Micrococcales	Intrasporangiaceae	Terrabacter	uncultured_bacterium_g__Terrabacter	OTU270	0	0	0	0
Firmicutes	Clostridia	Clostridiales	Ruminococcaceae	unclassified_f__Ruminococcaceae	unclassified_f__Ruminococcaceae	OTU681	0	0	2	2
Bacteroidetes	Bacteroidia	Bacteroidales	Bacteroidales_S24-7_g	norank_f__Bacteroidales_S24-7_gro	uncultured_bacterium_g__norank_f__Bacteroidales_S24-7_group	OTU310	0	0	0	0
Firmicutes	Clostridia	Clostridiales	Clostridiales_vadinBB6	norank_f__Clostridiales_vadinBB60_	uncultured_bacterium_g__norank_f__Clostridiales_vadinBB60_group	OTU528	0	0	0	0
Firmicutes	Clostridia	Clostridiales	Ruminococcaceae	Ruminiclostridium	uncultured_bacterium_g__Ruminiclostridium	OTU633	0	0	0	17
Firmicutes	Clostridia	Clostridiales	Christensenellaceae	Christensenellaceae_R-7_group	unclassified_g__Christensenellaceae_R-7_group	OTU695	0	0	1	0
Cyanobacteria	Cyanobacteria	norank_c__Cyanoba	norank_c__Cyanobact	norank_c__Cyanobacteria	unclassified_g__norank_c__Cyanobacteria	OTU258	0	0	0	0
Bacteroidetes	Bacteroidia	Bacteroidales	Prevotellaceae	Prevotellaceae_UCG-001	uncultured_Bacteroidales_bacterium_g__Prevotellaceae_UCG-001	OTU276	0	0	0	0
Firmicutes	Bacilli	Lactobacillales	Lactobacillaceae	Lactobacillus	unclassified_g__Lactobacillus	OTU632	0	0	0	1
Firmicutes	Clostridia	Clostridiales	Ruminococcaceae	Ruminiclostridium_9	Clostridiales_bacterium_NK3B98_g__Ruminiclostridium_9	OTU130	0	0	0	0
Firmicutes	Clostridia	Clostridiales	Ruminococcaceae	Ruminococcaceae_UCG-013	uncultured_bacterium_g__Ruminococcaceae_UCG-013	OTU807	0	0	0	0
Bacteroidetes	Bacteroidia	Bacteroidales	Rikenellaceae	Alistipes	gut_metagenome_g__Alistipes	OTU808	0	0	0	0
Firmicutes	Clostridia	unclassified_c__Clo	unclassified_c__Clostr	unclassified_c__Clostridia	unclassified_c__Clostridia	OTU99	0	1	0	0
Firmicutes	Clostridia	Clostridiales	Christensenellaceae	norank_f__Christensenellaceae	unclassified_g__norank_f__Christensenellaceae	OTU553	0	0	0	2
Firmicutes	Clostridia	Clostridiales	Lachnospiraceae	unclassified_f__Lachnospiraceae	unclassified_f__Lachnospiraceae	OTU80	0	0	0	0
Bacteroidetes	Bacteroidia	Bacteroidales	Prevotellaceae	Paraprevotella	unclassified_g__Paraprevotella	OTU308	0	0	0	0
Firmicutes	Clostridia	Clostridiales	Peptococcaceae	norank_f__Peptococcaceae	unclassified_g__norank_f__Peptococcaceae	OTU596	0	0	0	0
Firmicutes	Clostridia	Clostridiales	Lachnospiraceae	Lachnospiraceae_NK4A136_group	unclassified_g__Lachnospiraceae_NK4A136_group	OTU251	0	0	0	0
Firmicutes	Clostridia	Clostridiales	Family_XIII	[Eubacterium]_brachy_group	uncultured_bacterium_g__[Eubacterium]_brachy_group	OTU335	0	0	0	0
Firmicutes	Clostridia	Clostridiales	Lachnospiraceae	unclassified_f__Lachnospiraceae	unclassified_f__Lachnospiraceae	OTU86	0	0	0	0
Firmicutes	Clostridia	Clostridiales	Ruminococcaceae	Ruminococcaceae_UCG-005	uncultured_bacterium_adhufec311	OTU215	2	0	2	0
Firmicutes	Clostridia	Clostridiales	Ruminococcaceae	Ruminococcaceae_UCG-009	uncultured_bacterium_g__Ruminococcaceae_UCG-009	OTU641	0	0	1	0
Firmicutes	Clostridia	Clostridiales	Ruminococcaceae	Ruminiclostridium_1	unclassified_g__Ruminiclostridium_1	OTU178	0	2	0	0
Actinobacteria	Actinobacteria	Micrococcales	Micrococcaceae	Pseudarthrobacter	unclassified_g__Pseudarthrobacter	OTU423	0	0	0	0
Firmicutes	Clostridia	Clostridiales	Ruminococcaceae	Ruminococcaceae_UCG-010	unclassified_g__Ruminococcaceae_UCG-010	OTU26	0	1	0	0
Firmicutes	Clostridia	Clostridiales	Clostridiaceae_1	Clostridium_sensu_stricto_1	uncultured_bacterium_g__Clostridium_sensu_stricto_1	OTU128	0	0	0	0
Firmicutes	Clostridia	Clostridiales	Ruminococcaceae	Anaerotruncus	uncultured_bacterium_g__Anaerotruncus	OTU245	0	0	0	0
Firmicutes	Clostridia	Clostridiales	Ruminococcaceae	Ruminococcaceae_UCG-014	unclassified_g__Ruminococcaceae_UCG-014	OTU429	0	0	0	0
Firmicutes	Erysipelotrichia	Erysipelotrichales	Erysipelotrichaceae	Faecalibaculum	uncultured_bacterium_g__Faecalibaculum	OTU497	0	1	0	0
Firmicutes	Clostridia	Clostridiales	Ruminococcaceae	unclassified_f__Ruminococcaceae	unclassified_f__Ruminococcaceae	OTU525	0	0	0	0
Firmicutes	Clostridia	Clostridiales	Ruminococcaceae	Anaerotruncus	unclassified_g__Anaerotruncus	OTU640	0	0	2	0
Actinobacteria	Actinobacteria	Coriobacteriales	Coriobacteriaceae	Coriobacteriaceae_UCG-002	uncultured_bacterium_g__Coriobacteriaceae_UCG-002	OTU365	0	0	0	0
Firmicutes	Clostridia	Clostridiales	Christensenellaceae	Christensenellaceae_R-7_group	unclassified_g__Christensenellaceae_R-7_group	OTU670	0	2	0	0
Firmicutes	Clostridia	Clostridiales	Ruminococcaceae	Ruminococcaceae_UCG-010	uncultured_bacterium_g__Ruminococcaceae_UCG-010	OTU776	0	0	0	0
Firmicutes	Clostridia	Clostridiales	Christensenellaceae	norank_f__Christensenellaceae	uncultured_bacterium_g__norank_f__Christensenellaceae	OTU139	0	0	2	0
Firmicutes	Clostridia	Clostridiales	Christensenellaceae	Christensenellaceae_R-7_group	uncultured_bacterium_g__Christensenellaceae_R-7_group	OTU207	0	0	0	0
Actinobacteria	Actinobacteria	Coriobacteriales	Coriobacteriaceae	unclassified_f__Coriobacteriaceae	unclassified_f__Coriobacteriaceae	OTU477	6	2	0	0
Firmicutes	Clostridia	Clostridiales	Ruminococcaceae	unclassified_f__Ruminococcaceae	unclassified_f__Ruminococcaceae	OTU176	0	0	0	0
Bacteroidetes	Bacteroidia	Bacteroidales	Bacteroidales_S24-7_g	norank_f__Bacteroidales_S24-7_gro	uncultured_bacterium_g__norank_f__Bacteroidales_S24-7_group	OTU262	0	0	0	0
Firmicutes	Clostridia	Clostridiales	Ruminococcaceae	Anaerotruncus	unclassified_g__Anaerotruncus	OTU444	0	0	0	0
Firmicutes	Clostridia	Clostridiales	Ruminococcaceae	Ruminococcaceae_UCG-013	uncultured_Clostridiales_bacterium_g__Ruminococcaceae_UCG-013	OTU522	0	0	0	0
Firmicutes	Clostridia	Clostridiales	Ruminococcaceae	Ruminococcaceae_UCG-010	uncultured_organism_g__Ruminococcaceae_UCG-010	OTU782	0	0	0	0
Saccharibacteria	Unknown_Class_p__Sacc	Unknown_Order_c_	Unknown_Family_o__	Candidatus_Saccharimonas	uncultured_bacterium_g__Candidatus_Saccharimonas	OTU261	0	0	0	0
Firmicutes	Clostridia	Clostridiales	Lachnospiraceae	unclassified_f__Lachnospiraceae	unclassified_f__Lachnospiraceae	OTU120	1	1	0	1
Chloroflexi	KD4-96	norank_c__KD4-96	norank_c__KD4-96	norank_c__KD4-96	unclassified_g__norank_c__KD4-96	OTU254	1	0	0	0
Firmicutes	Clostridia	Clostridiales	Clostridiales_vadinBB6	norank_f__Clostridiales_vadinBB60_	unclassified_g__norank_f__Clostridiales_vadinBB60_group	OTU209	0	2	0	0
Tenericutes	Mollicutes	Mollicutes_RF9	norank_o__Mollicutes	norank_o__Mollicutes_RF9	unclassified_g__norank_o__Mollicutes_RF9	OTU572	0	0	0	0
Firmicutes	Clostridia	Clostridiales	Ruminococcaceae	unclassified_f__Ruminococcaceae	unclassified_f__Ruminococcaceae	OTU195	0	0	0	0
Proteobacteria	Alphaproteobacteria	Sphingomonadales	Sphingomonadaceae	Sphingomonas	uncultured_Sphingomonadaceae_bacterium_g__Sphingomonas	OTU375	0	0	0	0
Tenericutes	Mollicutes	Mollicutes_RF9	norank_o__Mollicutes	norank_o__Mollicutes_RF9	unclassified_g__norank_o__Mollicutes_RF9	OTU727	0	0	1	0
Firmicutes	Erysipelotrichia	Erysipelotrichales	Erysipelotrichaceae	Turicibacter	uncultured_bacterium_g__Turicibacter	OTU40	0	1	0	0
Firmicutes	Clostridia	Clostridiales	Lachnospiraceae	Coprococcus_1	intestinal_bacterium_CG19-1	OTU45	2	1	0	1
Firmicutes	Clostridia	Clostridiales	Peptococcaceae	norank_f__Peptococcaceae	unclassified_g__norank_f__Peptococcaceae	OTU116	0	0	0	0
Firmicutes	Clostridia	Clostridiales	Peptococcaceae	norank_f__Peptococcaceae	unclassified_g__norank_f__Peptococcaceae	OTU199	0	0	0	0
Bacteroidetes	Bacteroidia	Bacteroidales	Bacteroidaceae	Bacteroides	Bacteroides_acidifaciens	OTU320	0	0	0	0
Firmicutes	Clostridia	Clostridiales	Ruminococcaceae	unclassified_f__Ruminococcaceae	unclassified_f__Ruminococcaceae	OTU552	0	0	0	3
Firmicutes	Clostridia	Clostridiales	Lachnospiraceae	Butyrivibrio	uncultured_organism_g__Butyrivibrio	OTU762	0	0	0	0
Tenericutes	Mollicutes	Mollicutes_RF9	norank_o__Mollicutes	norank_o__Mollicutes_RF9	uncultured_bacterium_g__norank_o__Mollicutes_RF9	OTU797	0	0	0	0
Firmicutes	Clostridia	Clostridiales	Lachnospiraceae	unclassified_f__Lachnospiraceae	unclassified_f__Lachnospiraceae	OTU852	3	0	0	0
Proteobacteria	Alphaproteobacteria	Rhizobiales	Bradyrhizobiaceae	Bradyrhizobium	Bradyrhizobium_elkanii	OTU185	0	1	0	0
Chloroflexi	Chloroflexia	Chloroflexales	Roseiflexaceae	Roseiflexus	uncultured_bacterium_g__Roseiflexus	OTU415	0	0	0	0
Proteobacteria	Alphaproteobacteria	Rhizobiales	Xanthobacteraceae	norank_f__Xanthobacteraceae	uncultured_bacterium_g__norank_f__Xanthobacteraceae	OTU427	0	0	0	0
Firmicutes	unclassified_p__Firmicut	unclassified_p__Firi	unclassified_p__Firmi	unclassified_p__Firmicutes	unclassified_p__Firmicutes	OTU454	0	0	1	0
Firmicutes	Clostridia	Clostridiales	Ruminococcaceae	Anaerotruncus	unclassified_g__Anaerotruncus	OTU621	0	1	0	0
Firmicutes	Clostridia	Clostridiales	Ruminococcaceae	Anaerotruncus	uncultured_Clostridiales_bacterium_g__Anaerotruncus	OTU736	2	0	0	0
Firmicutes	Clostridia	Clostridiales	Ruminococcaceae	Hydrogenoanaerobacterium	Selenomonadales_bacterium_Marseille-P2399	OTU5	0	0	0	0
Bacteroidetes	Bacteroidia	Bacteroidales	Bacteroidales_S24-7_g	norank_f__Bacteroidales_S24-7_gro	uncultured_bacterium_g__norank_f__Bacteroidales_S24-7_group	OTU300	0	0	0	0
Bacteroidetes	Bacteroidia	Bacteroidales	Prevotellaceae	Prevotellaceae_NK3B31_group	uncultured_bacterium_g__Prevotellaceae_NK3B31_group	OTU331	0	0	0	0
Proteobacteria	Betaproteobacteria	Burkholderiales	Burkholderiaceae	Ralstonia	unidentified_marine_bacterioplankton_g__Ralstonia	OTU593	0	0	0	0
Tenericutes	Mollicutes	Mollicutes_RF9	norank_o__Mollicutes	norank_o__Mollicutes_RF9	uncultured_Erysipelotrichia_bacterium_g__norank	OTU594	0	1	1	0
Firmicutes	Clostridia	Clostridiales	Ruminococcaceae	Anaerotruncus	unclassified_g__Anaerotruncus	OTU713	0	0	0	1
Firmicutes	Clostridia	Clostridiales	Clostridiales_vadinBB6	norank_f__Clostridiales_vadinBB60_	uncultured_bacterium_g__norank_f__Clostridiales_vadinBB60_group	OTU724	0	0	0	0
Firmicutes	Clostridia	Clostridiales	Christensenellaceae	unclassified_f__Christensenellaceae	unclassified_f__Christensenellaceae	OTU793	0	1	0	1
Firmicutes	Clostridia	Clostridiales	Lachnospiraceae	Tyzzerella	unclassified_g__Tyzzerella	OTU796	0	0	3	1
Actinobacteria	Actinobacteria	Bifidobacteriales	Bifidobacteriaceae	Bifidobacterium	Bifidobacterium_pseudocatenulatum_DSM_20438__JCM_1200__LMG_10505	OTU55	0	2	0	0
Firmicutes	Clostridia	Clostridiales	Clostridiales_vadinBB6	norank_f__Clostridiales_vadinBB60_	uncultured_bacterium_g__norank_f__Clostridiales_vadinBB60_group	OTU336	0	0	0	0
Bacteroidetes	Bacteroidia	Bacteroidales	Bacteroidaceae	Bacteroides	Bacteroides_sartorii	OTU345	0	0	0	0
Firmicutes	Clostridia	Clostridiales	Ruminococcaceae	Ruminiclostridium_5	unclassified_g__Ruminiclostridium_5	OTU818	0	0	2	1
Firmicutes	Clostridia	Clostridiales	Christensenellaceae	norank_f__Christensenellaceae	uncultured_bacterium_g__norank_f__Christensenellaceae	OTU144	0	0	0	0
Firmicutes	Clostridia	Clostridiales	Ruminococcaceae	[Eubacterium]_coprostanoligenes_g	unclassified_g__[Eubacterium]_coprostanoligenes_group	OTU212	0	0	0	0
Firmicutes	Clostridia	Clostridiales	Ruminococcaceae	Ruminococcaceae_UCG-013	unclassified_g__Ruminococcaceae_UCG-013	OTU342	0	0	0	0
Chloroflexi	Chloroflexia	Chloroflexales	Roseiflexaceae	Roseiflexus	uncultured_bacterium_g__Roseiflexus	OTU433	0	0	0	0
Firmicutes	Clostridia	Clostridiales	Ruminococcaceae	unclassified_f__Ruminococcaceae	unclassified_f__Ruminococcaceae	OTU646	0	0	0	0
Firmicutes	Bacilli	Lactobacillales	Lactobacillaceae	Lactobacillus	Lactobacillus_saerimneri	OTU719	0	0	0	0
Firmicutes	Clostridia	Clostridiales	Ruminococcaceae	Ruminococcus_1	uncultured_organism_g__Ruminococcus_1	OTU227	0	0	0	0
Deferribacteres	Deferribacteres	Deferribacterales	Deferribacteraceae	Mucispirillum	uncultured_bacterium_g__Mucispirillum	OTU272	0	0	0	0
Chloroflexi	Chloroflexia	Chloroflexales	Roseiflexaceae	Roseiflexus	uncultured_bacterium_g__Roseiflexus	OTU277	0	0	0	0
Actinobacteria	Actinobacteria	Solirubrobacterales	Solirubrobacteraceae	Solirubrobacter	uncultured_bacterium_g__Solirubrobacter	OTU414	0	0	0	0
Firmicutes	Clostridia	Clostridiales	Ruminococcaceae	Anaerotruncus	unclassified_g__Anaerotruncus	OTU546	0	0	1	2
Tenericutes	Mollicutes	Mollicutes_RF9	norank_o__Mollicutes	norank_o__Mollicutes_RF9	uncultured_bacterium_g__norank_o__Mollicutes_RF9	OTU571	0	0	0	0
Firmicutes	Clostridia	Clostridiales	Ruminococcaceae	Ruminiclostridium_5	uncultured_bacterium_g__Ruminiclostridium_5	OTU806	0	0	0	0
Firmicutes	Clostridia	Clostridiales	Ruminococcaceae	Ruminococcaceae_UCG-013	uncultured_bacterium_g__Ruminococcaceae_UCG-013	OTU108	0	0	0	0
Firmicutes	Clostridia	Clostridiales	Peptococcaceae	norank_f__Peptococcaceae	uncultured_bacterium_g__norank_f__Peptococcaceae	OTU112	0	0	0	0
Acidobacteria	Acidobacteria	Blastocatellales	Blastocatellaceae__Su	RB41	unclassified_g__RB41	OTU374	0	0	0	0
Actinobacteria	Actinobacteria	Propionibacteriales	Nocardioidaceae	Marmoricola	uncultured_bacterium_g__Marmoricola	OTU395	0	0	0	0
Firmicutes	Clostridia	Clostridiales	Ruminococcaceae	unclassified_f__Ruminococcaceae	unclassified_f__Ruminococcaceae	OTU650	0	0	0	0
Firmicutes	Clostridia	Clostridiales	Ruminococcaceae	Ruminiclostridium_9	unclassified_g__Ruminiclostridium_9	OTU92	0	0	0	0
Actinobacteria	Actinobacteria	Streptomycetales	Streptomycetaceae	Streptomyces	unclassified_g__Streptomyces	OTU358	0	0	0	0
Proteobacteria	Deltaproteobacteria	Desulfovibrionales	Desulfovibrionaceae	Desulfovibrio	unclassified_g__Desulfovibrio	OTU431	0	0	0	0
Firmicutes	Clostridia	Clostridiales	Lachnospiraceae	unclassified_f__Lachnospiraceae	unclassified_f__Lachnospiraceae	OTU668	0	0	0	0
Firmicutes	Clostridia	Clostridiales	Syntrophomonadacea	Syntrophomonas	unclassified_g__Syntrophomonas	OTU790	0	0	0	0
Bacteroidetes	Bacteroidia	Bacteroidales	Bacteroidales_S24-7_g	norank_f__Bacteroidales_S24-7_gro	uncultured_bacterium_g__norank_f__Bacteroidales_S24-7_group	OTU369	0	0	0	0
Firmicutes	Clostridia	Clostridiales	Peptostreptococcacea	Peptoclostridium	Peptoclostridium_difficile_g__Peptoclostridium	OTU459	0	0	0	0
Firmicutes	Erysipelotrichia	Erysipelotrichales	Erysipelotrichaceae	[Clostridium]_innocuum_group	uncultured_bacterium_g__[Clostridium]_innocuum_group	OTU503	0	0	6	1
Firmicutes	Clostridia	Clostridiales	Ruminococcaceae	Ruminococcaceae_UCG-009	uncultured_bacterium_g__Ruminococcaceae_UCG-009	OTU686	0	0	0	0
Firmicutes	Clostridia	Clostridiales	Ruminococcaceae	Anaerotruncus	unclassified_g__Anaerotruncus	OTU753	0	0	0	0
Firmicutes	Clostridia	Clostridiales	Ruminococcaceae	Ruminiclostridium	uncultured_organism_g__Ruminiclostridium	OTU829	0	0	0	0
Bacteroidetes	Bacteroidia	Bacteroidales	Bacteroidales_S24-7_g	norank_f__Bacteroidales_S24-7_gro	uncultured_bacterium_g__norank_f__Bacteroidales_S24-7_group	OTU304	0	0	0	0
Bacteroidetes	Bacteroidia	Bacteroidales	Bacteroidales_S24-7_g	norank_f__Bacteroidales_S24-7_gro	uncultured_bacterium_g__norank_f__Bacteroidales_S24-7_group	OTU350	0	0	0	0
Bacteroidetes	Bacteroidia	Bacteroidales	Bacteroidales_S24-7_g	norank_f__Bacteroidales_S24-7_gro	uncultured_bacterium_g__norank_f__Bacteroidales_S24-7_group	OTU380	0	0	0	0

Firmicutes	Clostridia	Clostridiales	Ruminococcaceae	Candidatus_Soleaferrea	Ruminococcaceae_bacterium_GD6	OTU456	0	0	0	1
Firmicutes	Clostridia	Clostridiales	Ruminococcaceae	unclassified_f__Ruminococcaceae	unclassified_f__Ruminococcaceae	OTU545	0	0	2	5
Firmicutes	Clostridia	Clostridiales	Ruminococcaceae	Ruminococcaceae_UCG-009	uncultured_bacterium_g__Ruminococcaceae_UCG-009	OTU595	0	0	0	0
Firmicutes	Clostridia	Clostridiales	Ruminococcaceae	unclassified_f__Ruminococcaceae	unclassified_f__Ruminococcaceae	OTU844	0	0	0	0
Firmicutes	Clostridia	Clostridiales	Christensenellaceae	norank_f__Christensenellaceae	uncultured_bacterium_g__norank_f__Christensenellaceae	OTU126	0	0	0	0
Firmicutes	Clostridia	Clostridiales	Clostridiales_vadinBB6	norank_f__Clostridiales_vadinBB60_	uncultured_bacterium_g__norank_f__Clostridiales_vadinBB60_group	OTU161	0	0	0	0
Bacteroidetes	Bacteroidia	Bacteroidales	Bacteroidales_S24-7_g	norank_f__Bacteroidales_S24-7_gro	unclassified_g__norank_f__Bacteroidales_S24-7_group	OTU267	0	0	0	0
Actinobacteria	Actinobacteria	Streptosporangiales	Thermomonosporacea	Actinomadura	Actinomadura_hallensis	OTU291	0	0	0	0
Firmicutes	Clostridia	Clostridiales	Ruminococcaceae	Ruminiclostridium_5	unclassified_g__Ruminiclostridium_5	OTU341	0	0	0	0
Actinobacteria	Actinobacteria	Corynebacteriales	Mycobacteriaceae	Mycobacterium	unclassified_g__Mycobacterium	OTU381	0	0	0	0
Firmicutes	Clostridia	Clostridiales	Ruminococcaceae	Ruminococcus_1	unclassified_g__Ruminococcus_1	OTU523	0	0	0	0
Bacteroidetes	Bacteroidia	Bacteroidales	Rikenellaceae	Alistipes	Alistipes_sp._CHKCI003	OTU15	0	0	0	0
Firmicutes	Clostridia	Clostridiales	Ruminococcaceae	Ruminococcaceae_UCG-010	unclassified_g__Ruminococcaceae_UCG-010	OTU222	0	0	0	0
Firmicutes	Clostridia	Clostridiales	Clostridiales_vadinBB6	norank_f__Clostridiales_vadinBB60_	uncultured_prokaryote_g__norank_f__Clostridiales_vadinBB60_group	OTU247	0	0	0	1
Bacteroidetes	Bacteroidia	Bacteroidales	Bacteroidales_S24-7_g	norank_f__Bacteroidales_S24-7_gro	uncultured_bacterium_g__norank_f__Bacteroidales_S24-7_group	OTU253	0	0	0	0
Verrucomicrobia	Spartobacteria	Chthoniobacterales	DA101_soil_group	norank_f__DA101_soil_group	uncultured_Prosthecobacter_sp._g__norank_f__DA101_soil_group	OTU279	0	0	0	0
Cyanobacteria	Cyanobacteria	Gastranaerophilales	norank_o__Gastranae	norank_o__Gastranaerophilales	unclassified_g__norank_o__Gastranaerophilales	OTU328	0	0	0	0
Proteobacteria	Epsilonproteobacteria	Campylobacterales	Helicobacteraceae	Helicobacter	Helicobacter_ganmani	OTU446	0	0	0	0
Firmicutes	Clostridia	Clostridiales	Ruminococcaceae	Ruminococcaceae_UCG-007	unclassified_g__Ruminococcaceae_UCG-007	OTU789	0	1	0	0
Proteobacteria	Alphaproteobacteria	Rhizobiales	Xanthobacteraceae	Variibacter	uncultured_forest_soil_bacterium_g__Variibacter	OTU840	0	0	0	0
Firmicutes	Clostridia	Clostridiales	Ruminococcaceae	Ruminiclostridium_5	uncultured_organism_g__Ruminiclostridium_5	OTU24	0	0	0	0
Gemmatimonade	Gemmatimonadetes	Gemmatimonadales	Gemmatimonadaceae	Gemmatimonas	uncultured_soil_bacterium_g__Gemmatimonas	OTU354	0	0	0	0
Actinobacteria	Actinobacteria	Propionibacteriales	Nocardioidaceae	Kribbella	uncultured_bacterium_g__Kribbella	OTU391	0	0	0	0
Actinobacteria	Actinobacteria	Pseudonocardiales	Pseudonocardiaceae	Pseudonocardia	uncultured_bacterium_g__Pseudonocardia	OTU392	0	0	0	0
Firmicutes	Clostridia	Clostridiales	Lachnospiraceae	unclassified_f__Lachnospiraceae	unclassified_f__Lachnospiraceae	OTU462	0	0	0	0
Firmicutes	Clostridia	Clostridiales	Ruminococcaceae	Anaerotruncus	unclassified_g__Anaerotruncus	OTU486	0	1	0	0
Actinobacteria	Actinobacteria	Coriobacteriales	Coriobacteriaceae	Gordonibacter	unclassified_g__Gordonibacter	OTU555	0	0	0	0
Firmicutes	Clostridia	Clostridiales	Ruminococcaceae	Anaerotruncus	unclassified_g__Anaerotruncus	OTU687	0	0	0	1
unclassified_k__r	unclassified_k__norank	unclassified_k__no	unclassified_k__noran	unclassified_k__norank	unclassified_k__norank	OTU706	0	0	0	0
Proteobacteria	Alphaproteobacteria	Rhizobiales	Xanthobacteraceae	Variibacter	uncultured_forest_soil_bacterium_g__Variibacter	OTU252	0	0	0	0
Actinobacteria	Actinobacteria	Micromonosporales	Micromonosporaceae	Longispora	uncultured_bacterium_g__Longispora	OTU357	0	0	0	0
Actinobacteria	Actinobacteria	Frankiales	Acidothermaceae	Acidothermus	Thermomonosporaceae_bacterium_YE4-D4-16-CH2	OTU403	0	0	0	0
Tenericutes	Mollicutes	Mollicutes_RF9	norank_o__Mollicutes	norank_o__Mollicutes_RF9	unclassified_g__norank_o__Mollicutes_RF9	OTU547	0	0	0	4
Bacteroidetes	Bacteroidia	Bacteroidales	Bacteroidaceae	Bacteroides	unclassified_g__Bacteroides	OTU554	1	0	0	0
Firmicutes	Clostridia	Clostridiales	Ruminococcaceae	unclassified_f__Ruminococcaceae	unclassified_f__Ruminococcaceae	OTU708	0	0	0	0
Firmicutes	Clostridia	Clostridiales	Christensenellaceae	unclassified_f__Christensenellaceae	unclassified_f__Christensenellaceae	OTU205	0	0	0	0
Firmicutes	Clostridia	Clostridiales	Ruminococcaceae	Ruminococcaceae_UCG-010	uncultured_bacterium_g__Ruminococcaceae_UCG-010	OTU240	0	0	0	0
Bacteroidetes	Bacteroidia	Bacteroidales	Rikenellaceae	Rikenella	uncultured_bacterium_g__Rikenella	OTU244	0	0	0	0
Bacteroidetes	Bacteroidia	Bacteroidales	Bacteroidales_S24-7_g	norank_f__Bacteroidales_S24-7_gro	uncultured_bacterium_g__norank_f__Bacteroidales_S24-7_group	OTU259	0	0	0	0
Bacteroidetes	Bacteroidia	Bacteroidales	Bacteroidales_S24-7_g	norank_f__Bacteroidales_S24-7_gro	unclassified_g__norank_f__Bacteroidales_S24-7_group	OTU280	0	0	0	0
Actinobacteria	Actinobacteria	Micromonosporales	Micromonosporaceae	unclassified_f__Micromonosporacea	unclassified_f__Micromonosporaceae	OTU286	0	0	0	0
Actinobacteria	Actinobacteria	Gaiellales	Gaiellaceae	Gaiella	uncultured_bacterium_g__Gaiella	OTU309	0	0	0	0
Bacteroidetes	Bacteroidia	Bacteroidales	Bacteroidales_S24-7_g	norank_f__Bacteroidales_S24-7_gro	uncultured_Bacteroidales_bacterium_g__norank_f__Bacteroidales_S24-7_group	OTU348	0	0	0	0
Actinobacteria	Actinobacteria	Propionibacteriales	Nocardioidaceae	Nocardioides	uncultured_bacterium_g__Nocardioides	OTU367	0	0	0	0
Actinobacteria	Actinobacteria	norank_c__Actinob	norank_c__Actinobact	norank_c__Actinobacteria	uncultured_bacterium_g__norank_c__Actinobacteria	OTU396	0	0	0	0
Firmicutes	Clostridia	Clostridiales	Ruminococcaceae	unclassified_f__Ruminococcaceae	unclassified_f__Ruminococcaceae	OTU617	0	0	0	0
Proteobacteria	Alphaproteobacteria	Rhizobiales	Methylobacteriaceae	Meganema	Meganema_perideroedes	OTU30	0	0	0	0
Firmicutes	Clostridia	Clostridiales	Ruminococcaceae	unclassified_f__Ruminococcaceae	unclassified_f__Ruminococcaceae	OTU239	0	0	0	0
Proteobacteria	Betaproteobacteria	Burkholderiales	Alcaligenaceae	Parasutterella	Sutterellaceae_bacterium_YL45	OTU281	0	0	0	0
Bacteroidetes	Bacteroidia	Bacteroidales	Bacteroidales_S24-7_g	norank_f__Bacteroidales_S24-7_gro	unclassified_g__norank_f__Bacteroidales_S24-7_group	OTU303	0	0	0	0
Bacteroidetes	Bacteroidia	Bacteroidales	Bacteroidales_S24-7_g	norank_f__Bacteroidales_S24-7_gro	uncultured_Bacteroidales_bacterium_g__norank_f__Bacteroidales_S24-7_group	OTU307	0	0	0	0
Chloroflexi	Anaerolineae	Anaerolineales	Anaerolineaceae	norank_f__Anaerolineaceae	wastewater_metagenome_g__norank_f__Anaerolineaceae	OTU352	0	0	0	0
Bacteroidetes	Bacteroidia	Bacteroidales	Bacteroidales_S24-7_g	norank_f__Bacteroidales_S24-7_gro	uncultured_bacterium_g__norank_f__Bacteroidales_S24-7_group	OTU389	0	0	0	0
Chloroflexi	Chloroflexia	Chloroflexales	Roseiflexaceae	Roseiflexus	uncultured_bacterium_g__Roseiflexus	OTU404	0	0	0	0
Firmicutes	Clostridia	Clostridiales	Lachnospiraceae	Lachnoclostridium	unclassified_g__Lachnoclostridium	OTU416	0	0	0	1
Chloroflexi	Chloroflexia	Chloroflexales	Roseiflexaceae	Roseiflexus	uncultured_bacterium_g__Roseiflexus	OTU425	0	0	0	0
Actinobacteria	Actinobacteria	Corynebacteriales	Nocardiaceae	Rhodococcus	Rhodococcus_erythropolis	OTU662	0	0	0	0
Proteobacteria	Gammaproteobacteria	Pseudomonadales	Moraxellaceae	Acinetobacter	unclassified_g__Acinetobacter	OTU685	0	0	0	0
Firmicutes	Clostridia	Clostridiales	Clostridiaceae_1	Clostridium_sensu_stricto_1	unclassified_g__Clostridium_sensu_stricto_1	OTU817	0	0	0	0
Firmicutes	Clostridia	Clostridiales	Christensenellaceae	Christensenellaceae_R-7_group	uncultured_Clostridiaceae_bacterium_g__Christensenellaceae_R-7_group	OTU47	0	0	0	0
Firmicutes	Clostridia	Clostridiales	Ruminococcaceae	Anaerotruncus	unclassified_g__Anaerotruncus	OTU96	0	0	0	0
Firmicutes	Clostridia	Clostridiales	Clostridiales_vadinBB6	norank_f__Clostridiales_vadinBB60_	uncultured_prokaryote_g__norank_f__Clostridiales_vadinBB60_group	OTU147	0	0	0	0
Firmicutes	Clostridia	Clostridiales	Ruminococcaceae	Candidatus_Soleaferrea	Ruminococcaceae_bacterium_Marseille-P2935	OTU183	0	0	0	0
Tenericutes	Mollicutes	Mollicutes_RF9	norank_o__Mollicutes	norank_o__Mollicutes_RF9	uncultured_bacterium_g__norank_o__Mollicutes_RF9	OTU208	0	0	0	0
Firmicutes	unclassified_p__Firmicut	unclassified_p__Firi	unclassified_p__Firmi	unclassified_p__Firmicutes	unclassified_p__Firmicutes	OTU246	0	0	0	0
Bacteroidetes	Bacteroidia	Bacteroidales	Bacteroidales_S24-7_g	norank_f__Bacteroidales_S24-7_gro	uncultured_bacterium_g__norank_f__Bacteroidales_S24-7_group	OTU266	0	0	0	0
Bacteroidetes	Bacteroidia	Bacteroidales	Rikenellaceae	Alistipes	uncultured_bacterium_g__Alistipes	OTU282	0	0	0	0
Actinobacteria	Actinobacteria	Solirubrobacterales	Solirubrobacteraceae	Solirubrobacter	uncultured_bacterium_g__Solirubrobacter	OTU288	0	0	0	0
Bacteroidetes	Bacteroidia	Bacteroidales	Bacteroidales_S24-7_g	norank_f__Bacteroidales_S24-7_gro	uncultured_bacterium_g__norank_f__Bacteroidales_S24-7_group	OTU289	0	0	0	0
Bacteroidetes	Bacteroidia	Bacteroidales	Bacteroidaceae	Bacteroides	unclassified_g__Bacteroides	OTU290	0	0	0	0
Chloroflexi	Chloroflexia	Chloroflexales	Roseiflexaceae	Roseiflexus	uncultured_bacterium_g__Roseiflexus	OTU318	0	0	0	0
Acidobacteria	Acidobacteria	norank_c__Acidoba	norank_c__Acidobacte	norank_c__Acidobacteria	uncultured_Acidobacteriales_bacterium_g__norank_c__Acidobacteria	OTU366	0	0	0	0
Proteobacteria	Epsilonproteobacteria	Campylobacterales	Campylobacteraceae	Arcobacter	unclassified_g__Arcobacter	OTU376	0	0	0	0
Gemmatimonade	Gemmatimonadetes	Gemmatimonadales	Gemmatimonadaceae	Gemmatimonas	unclassified_g__Gemmatimonas	OTU377	0	0	0	0
Proteobacteria	Alphaproteobacteria	Rhizobiales	Methylocystaceae	unclassified_f__Methylocystaceae	unclassified_f__Methylocystaceae	OTU379	0	0	0	0
Proteobacteria	Alphaproteobacteria	Rhodobacterales	Rhodobacteraceae	Pannonibacter	unclassified_g__Pannonibacter	OTU421	0	0	0	0
Proteobacteria	Epsilonproteobacteria	Campylobacterales	Helicobacteraceae	Helicobacter	Helicobacter_hepaticus	OTU422	0	0	0	0
Tenericutes	Mollicutes	Mollicutes_RF9	norank_o__Mollicutes	norank_o__Mollicutes_RF9	unclassified_g__norank_o__Mollicutes_RF9	OTU491	0	6	0	0
Firmicutes	Clostridia	Clostridiales	Ruminococcaceae	unclassified_f__Ruminococcaceae	unclassified_f__Ruminococcaceae	OTU696	0	0	0	0
Firmicutes	Clostridia	Clostridiales	Christensenellaceae	norank_f__Christensenellaceae	uncultured_bacterium_g__norank_f__Christensenellaceae	OTU819	0	0	0	0
Firmicutes	Clostridia	Clostridiales	Ruminococcaceae	unclassified_f__Ruminococcaceae	unclassified_f__Ruminococcaceae	OTU186	0	0	0	0
Firmicutes	Clostridia	Clostridiales	Ruminococcaceae	Ruminiclostridium	uncultured_bacterium_g__Ruminiclostridium	OTU269	0	0	0	0
Bacteroidetes	Bacteroidia	Bacteroidales	Porphyromonadaceae	Parabacteroides	uncultured_organism_g__Parabacteroides	OTU285	0	0	0	0
Bacteroidetes	Bacteroidia	Bacteroidales	Rikenellaceae	Alistipes	uncultured_bacterium_g__Alistipes	OTU299	0	0	0	0
Actinobacteria	Actinobacteria	Acidimicrobiales	norank_o__Acidimicro	norank_o__Acidimicrobiales	uncultured_Acidimicrobiales_bacterium_g__norank	OTU305	0	0	0	0
Chloroflexi	S085	norank_c__S085	norank_c__S085	norank_c__S085	uncultured_Chloroflexi_bacterium_g__norank_c__S085	OTU362	0	0	0	0
Proteobacteria	Alphaproteobacteria	Rhodospirillales	Acetobacteraceae	norank_f__Acetobacteraceae	uncultured_bacterium_g__norank_f__Acetobacteraceae	OTU371	0	0	0	0
Chloroflexi	TK10	norank_c__TK10	norank_c__TK10	norank_c__TK10	unclassified_g__norank_c__TK10	OTU382	0	0	0	0
Actinobacteria	Actinobacteria	Micrococcales	Brevibacteriaceae	Brevibacterium	unclassified_g__Brevibacterium	OTU399	0	0	0	0
Bacteroidetes	Bacteroidia	Bacteroidales	Bacteroidales_S24-7_g	norank_f__Bacteroidales_S24-7_gro	uncultured_organism_g__norank_f__Bacteroidales_S24-7_group	OTU401	0	0	0	0
Firmicutes	Erysipelotrichia	Erysipelotrichales	Erysipelotrichaceae	Erysipelatoclostridium	uncultured_bacterium_g__Erysipelatoclostridium	OTU407	0	0	0	0
Proteobacteria	Betaproteobacteria	Nitrosomonadales	Nitrosomonadaceae	norank_f__Nitrosomonadaceae	unclassified_g__norank_f__Nitrosomonadaceae	OTU420	0	0	0	0
Firmicutes	Clostridia	Clostridiales	Ruminococcaceae	Ruminococcaceae_UCG-014	uncultured_Firmicutes_bacterium_g__Ruminococcaceae_UCG-014	OTU798	0	0	0	0
Firmicutes	Clostridia	Clostridiales	Ruminococcaceae	Ruminococcaceae_UCG-010	unclassified_g__Ruminococcaceae_UCG-010	OTU800	0	0	0	0
Tenericutes	Mollicutes	Mycoplasmatales	Mycoplasmataceae	norank_f__Mycoplasmataceae	unclassified_g__norank_f__Mycoplasmataceae	OTU825	0	0	0	0
Firmicutes	Clostridia	Clostridiales	Christensenellaceae	Christensenellaceae_R-7_group	unclassified_g__Christensenellaceae_R-7_group	OTU833	0	0	0	0
Bacteroidetes	Bacteroidia	Bacteroidales	Bacteroidaceae	Bacteroides	Bacteroides_caccae	OTU46	0	1	0	0
Bacteroidetes	Flavobacteriia	Flavobacteriales	Flavobacteriaceae	Cloacibacterium	uncultured_bacterium_g__Cloacibacterium	OTU49	0	0	0	0
Firmicutes	Clostridia	Clostridiales	Ruminococcaceae	Ruminococcaceae_UCG-010	uncultured_bacterium_g__Ruminococcaceae_UCG-010	OTU97	0	0	0	0
Proteobacteria	Gammaproteobacteria	Pseudomonadales	Moraxellaceae	Acinetobacter	Acinetobacter_guillouiae	OTU138	0	0	0	0
Bacteroidetes	Bacteroidia	Bacteroidales	Bacteroidales_S24-7_g	norank_f__Bacteroidales_S24-7_gro	uncultured_bacterium_g__norank_f__Bacteroidales_S24-7_group	OTU211	0	0	0	0
Actinobacteria	Actinobacteria	Acidimicrobiales	Acidimicrobiaceae	CL500-29_marine_group	uncultured_bacterium_g__CL500-29_marine_group	OTU260	0	0	0	0
Acidobacteria	Acidobacteria	Acidobacteriales	Acidobacteriaceae__S	norank_f__Acidobacteriaceae__Subg	uncultured_bacterium_g__norank_f__Acidobacteriaceae__Subgroup_1_	OTU263	0	0	0	0
Proteobacteria	Alphaproteobacteria	Rhodospirillales	Rhodospirillaceae	norank_f__Rhodospirillaceae	unclassified_g__norank_f__Rhodospirillaceae	OTU273	0	0	0	0
Actinobacteria	Actinobacteria	Propionibacteriales	Nocardioidaceae	unclassified_f__Nocardioidaceae	unclassified_f__Nocardioidaceae	OTU298	0	0	0	0
Bacteroidetes	Bacteroidia	Bacteroidales	Bacteroidaceae	Bacteroides	Bacteroides_stercoris_ATCC_43183	OTU317	0	0	0	0
Chloroflexi	Chloroflexia	Chloroflexales	Roseiflexaceae	Roseiflexus	uncultured_bacterium_g__Roseiflexus	OTU372	0	0	0	0
Chloroflexi	Anaerolineae	Anaerolineales	Anaerolineaceae	norank_f__Anaerolineaceae	Chloroflexi_bacterium_OLB13	OTU383	0	0	0	0
Actinobacteria	Actinobacteria	Propionibacteriales	Nocardioidaceae	Nocardioides	unclassified_g__Nocardioides	OTU386	0	0	0	0
Chloroflexi	Thermomicrobia	JG30-KF-CM45	norank_o__JG30-KF-C	norank_o__JG30-KF-CM45	unclassified_g__norank_o__JG30-KF-CM45	OTU408	0	0	0	0
Chloroflexi	Gitt-GS-136	norank_c__Gitt-GS-	norank_c__Gitt-GS-13	norank_c__Gitt-GS-136	uncultured_bacterium_g__norank_c__Gitt-GS-136	OTU413	0	0	0	0

Actinobacteria	Actinobacteria	Acidimicrobiales	Iamiaceae	Iamia	uncultured_bacterium_g__Iamia	OTU417	0	0	0	0
Actinobacteria	Actinobacteria	Frankiales	Geodermatophilaceae	Blastococcus	uncultured_Blastococcus_sp._g__Blastococcus	OTU419	0	0	0	0
Actinobacteria	Actinobacteria	Propionibacteriales	Nocardioidaceae	Nocardioides	uncultured_organism_g__Nocardioides	OTU430	0	0	0	0
Gemmatimonade	Gemmatimonadetes	Gemmatimonadale	Gemmatimonadaceae	norank_f__Gemmatimonadaceae	uncultured_Gemmatimonadetes_bacterium_g__norank_f__Gemmatimonadaceae	OTU442	0	0	0	0
Firmicutes	Clostridia	Clostridiales	Ruminococcaceae	Anaerotruncus	uncultured_bacterium_g__Anaerotruncus	OTU592	0	0	0	0
Firmicutes	Clostridia	Clostridiales	Lachnospiraceae	Coprococcus_1	unclassified_g__Coprococcus_1	OTU734	0	0	0	0
Firmicutes	Clostridia	Clostridiales	Ruminococcaceae	Candidatus_Soleaferrea	Candidatus_Soleaferrea_massiliensis	OTU37	0	0	0	0
Cyanobacteria	Cyanobacteria	norank_c__Cyanoba	norank_c__Cyanobact	norank_c__Cyanobacteria	unclassified_g__norank_c__Cyanobacteria	OTU149	0	1	0	0
Firmicutes	Clostridia	Clostridiales	Ruminococcaceae	Ruminococcaceae_UCG-010	unclassified_g__Ruminococcaceae_UCG-010	OTU157	0	0	0	0
Bacteroidetes	Bacteroidia	Bacteroidales	Bacteroidales_S24-7_g	norank_f__Bacteroidales_S24-7_gro	unclassified_g__norank_f__Bacteroidales_S24-7_group	OTU190	0	0	0	0
Firmicutes	Clostridia	Clostridiales	Ruminococcaceae	Ruminococcaceae_UCG-010	uncultured_bacterium_g__Ruminococcaceae_UCG-010	OTU225	0	0	0	0
Proteobacteria	Deltaproteobacteria	Desulfovibrionales	Desulfovibrionaceae	Desulfovibrio	uncultured_bacterium_g__Desulfovibrio	OTU250	0	0	0	0
Bacteroidetes	Bacteroidia	Bacteroidales	Bacteroidales_S24-7_g	norank_f__Bacteroidales_S24-7_gro	uncultured_bacterium_g__norank_f__Bacteroidales_S24-7_group	OTU265	0	0	0	0
Proteobacteria	Betaproteobacteria	Burkholderiales	Comamonadaceae	unclassified_f__Comamonadaceae	unclassified_f__Comamonadaceae	OTU268	0	0	0	0
Firmicutes	Bacilli	Bacillales	Bacillaceae	unclassified_f__Bacillaceae	unclassified_f__Bacillaceae	OTU274	0	0	0	0
Firmicutes	Bacilli	Bacillales	Bacillaceae	Bacillus	Bacillus_anthracis_g__Bacillus	OTU275	0	0	0	0
Proteobacteria	Alphaproteobacteria	Rickettsiales	Mitochondria	norank_f__Mitochondria	Brassica_nigra__black_mustard_	OTU283	0	0	0	0
Firmicutes	Clostridia	Clostridiales	Ruminococcaceae	Anaerotruncus	Anaerotruncus_sp._G3_2012_	OTU296	0	0	0	0
Verrucomicrobia	Spartobacteria	Chthoniobacterales	DA101_soil_group	norank_f__DA101_soil_group	uncultured_Spartobacteria_bacterium_g__norank	OTU302	0	0	0	0
Actinobacteria	Actinobacteria	Acidimicrobiales	norank_o__Acidimicro	norank_o__Acidimicrobiales	uncultured_bacterium_g__norank_o__Acidimicrobiales	OTU314	0	0	0	0
Chloroflexi	Anaerolineae	Anaerolineales	Anaerolineaceae	norank_f__Anaerolineaceae	unclassified_g__norank_f__Anaerolineaceae	OTU316	0	0	0	0
Actinobacteria	Actinobacteria	Pseudonocardiales	Pseudonocardiaceae	Pseudonocardia	unclassified_g__Pseudonocardia	OTU351	0	0	0	0
Acidobacteria	Acidobacteria	Blastocatellales	Blastocatellaceae__Su	RB41	uncultured_Acidobacteria_bacterium_g__RB41	OTU359	0	0	0	0
Actinobacteria	Actinobacteria	Gaiellales	norank_o__Gaiellales	norank_o__Gaiellales	uncultured_bacterium_g__norank_o__Gaiellales	OTU370	0	0	0	0
Chloroflexi	Chloroflexia	Chloroflexales	Roseiflexaceae	Roseiflexus	uncultured_bacterium_g__Roseiflexus	OTU378	0	0	0	0
Chloroflexi	Chloroflexia	Chloroflexales	Roseiflexaceae	Roseiflexus	uncultured_bacterium_g__Roseiflexus	OTU384	0	0	0	0
Acidobacteria	Acidobacteria	Blastocatellales	Blastocatellaceae__Su	RB41	unclassified_g__RB41	OTU387	0	0	0	0
Acidobacteria	Acidobacteria	Solibacterales	Solibacteraceae__Subg	Bryobacter	uncultured_Acidobacterium_sp._g__Bryobacter	OTU388	0	0	0	0
Nitrospirae	Nitrospira	norank_c__Nitrospi	norank_c__Nitrospira	Nitrospira	unclassified_g__Nitrospira	OTU390	0	0	0	0
Actinobacteria	Actinobacteria	Acidimicrobiales	Acidimicrobiales_Incer	Candidatus_Microthrix	uncultured_bacterium_g__Candidatus_Microthrix	OTU393	0	0	0	0
Chloroflexi	Anaerolineae	Anaerolineales	Anaerolineaceae	norank_f__Anaerolineaceae	unclassified_g__norank_f__Anaerolineaceae	OTU397	0	0	0	0
Firmicutes	Clostridia	Clostridiales	Lachnospiraceae	norank_f__Lachnospiraceae	Lachnospiraceae_bacterium_A2	OTU398	0	0	0	0
Actinobacteria	Actinobacteria	Solirubrobacterales	Gsoil-1167	norank_f__Gsoil-1167	uncultured_bacterium_g__norank_f__Gsoil-1167	OTU406	0	0	0	0
Proteobacteria	Alphaproteobacteria	Rhizobiales	Bradyrhizobiaceae	Bosea	unclassified_g__Bosea	OTU426	0	0	0	0
Chloroflexi	Ktedonobacteria	JG30-KF-AS9	norank_o__JG30-KF-A	norank_o__JG30-KF-AS9	uncultured_bacterium_g__norank_o__JG30-KF-AS9	OTU440	0	0	0	0
Gemmatimonade	Gemmatimonadetes	Gemmatimonadale	Gemmatimonadaceae	Gemmatimonas	unclassified_g__Gemmatimonas	OTU441	0	0	0	0
Firmicutes	Clostridia	Clostridiales	Clostridiales_vadinBB6	norank_f__Clostridiales_vadinBB60_	unclassified_g__norank_f__Clostridiales_vadinBB60_group	OTU479	0	0	0	0
Firmicutes	Clostridia	Clostridiales	Lachnospiraceae	unclassified_f__Lachnospiraceae	unclassified_f__Lachnospiraceae	OTU514	0	0	0	0
Firmicutes	Clostridia	Clostridiales	Ruminococcaceae	unclassified_f__Ruminococcaceae	unclassified_f__Ruminococcaceae	OTU569	0	0	0	0
Firmicutes	Clostridia	Clostridiales	Ruminococcaceae	unclassified_f__Ruminococcaceae	unclassified_f__Ruminococcaceae	OTU760	0	0	0	0
Firmicutes	Clostridia	Clostridiales	Ruminococcaceae	Ruminococcaceae_UCG-005	unclassified_g__Ruminococcaceae_UCG-005	OTU814	0	0	0	0
Firmicutes	Clostridia	Clostridiales	Ruminococcaceae	Anaerotruncus	unclassified_g__Anaerotruncus	OTU44	0	0	0	0
Firmicutes	Clostridia	Clostridiales	Clostridiaceae_1	Clostridium_sensu_stricto_1	human_gut_metagenome_g__Clostridium_sensu_stricto_1	OTU77	0	0	0	0
Firmicutes	Clostridia	Clostridiales	Christensenellaceae	Christensenellaceae_R-7_group	unclassified_g__Christensenellaceae_R-7_group	OTU119	0	0	0	0
Bacteroidetes	Bacteroidia	Bacteroidales	Porphyromonadaceae	Odoribacter	uncultured_bacterium_g__Odoribacter	OTU210	0	0	0	0
Bacteroidetes	Bacteroidia	Bacteroidales	Bacteroidales_S24-7_g	norank_f__Bacteroidales_S24-7_gro	uncultured_bacterium_g__norank_f__Bacteroidales_S24-7_group	OTU217	0	0	0	0
Acidobacteria	Acidobacteria	norank_c__Acidoba	norank_c__Acidobacte	norank_c__Acidobacteria	uncultured_Acidobacteriales_bacterium_g__norank_c__Acidobacteria	OTU256	0	0	0	0
Gemmatimonade	Gemmatimonadetes	Gemmatimonadale	Gemmatimonadaceae	unclassified_f__Gemmatimonadacea	unclassified_f__Gemmatimonadaceae	OTU257	0	0	0	0
Acidobacteria	Acidobacteria	norank_c__Acidoba	norank_c__Acidobacte	norank_c__Acidobacteria	unclassified_g__norank_c__Acidobacteria	OTU264	0	0	0	0
Proteobacteria	Betaproteobacteria	Neisseriales	Neisseriaceae	Neisseria	unclassified_g__Neisseria	OTU271	0	0	0	0
Planctomycetes	OM190	norank_c__OM190	norank_c__OM190	norank_c__OM190	uncultured_bacterium_g__norank_c__OM190	OTU278	0	0	0	0
Actinobacteria	Actinobacteria	Coriobacteriales	Coriobacteriaceae	Parvibacter	uncultured_bacterium_g__Parvibacter	OTU292	0	0	0	0
Acidobacteria	Acidobacteria	norank_c__Acidoba	norank_c__Acidobacte	norank_c__Acidobacteria	uncultured_prokaryote_g__norank_c__Acidobacteria	OTU293	0	0	0	0
Actinobacteria	Actinobacteria	Gaiellales	norank_o__Gaiellales	norank_o__Gaiellales	uncultured_Rubrobacteria_bacterium_g__norank	OTU295	0	0	0	0
Proteobacteria	Gammaproteobacteria	BD7-8_marine_grou	norank_o__BD7-8_ma	norank_o__BD7-8_marine_group	unclassified_g__norank_o__BD7-8_marine_group	OTU297	0	0	0	0
Chloroflexi	Chloroflexia	Chloroflexales	Roseiflexaceae	Roseiflexus	uncultured_bacterium_g__Roseiflexus	OTU301	0	0	0	0
Actinobacteria	Actinobacteria	Propionibacteriales	Nocardioidaceae	unclassified_f__Nocardioidaceae	unclassified_f__Nocardioidaceae	OTU306	0	0	0	0
Saccharibacteria	norank_p__Saccharibact	norank_p__Sacchar	norank_p__Saccharibac	norank_p__Saccharibacteria	uncultured_bacterium_g__norank_p__Saccharibacteria	OTU311	0	0	0	0
Acidobacteria	Acidobacteria	norank_c__Acidoba	norank_c__Acidobacte	norank_c__Acidobacteria	uncultured_bacterium_66	OTU315	0	0	0	0
Firmicutes	Bacilli	Bacillales	Bacillaceae	unclassified_f__Bacillaceae	unclassified_f__Bacillaceae	OTU319	0	0	0	0
Firmicutes	Clostridia	Clostridiales	Clostridiales_vadinBB6	norank_f__Clostridiales_vadinBB60_	uncultured_bacterium_g__norank_f__Clostridiales_vadinBB60_group	OTU340	0	0	0	0
Acidobacteria	Acidobacteria	norank_c__Acidoba	norank_c__Acidobacte	norank_c__Acidobacteria	unclassified_g__norank_c__Acidobacteria	OTU349	0	0	0	0
Saccharibacteria	norank_p__Saccharibact	norank_p__Sacchar	norank_p__Saccharibac	norank_p__Saccharibacteria	uncultured_bacterium_g__norank_p__Saccharibacteria	OTU353	0	0	0	0
Chloroflexi	Anaerolineae	Anaerolineales	Anaerolineaceae	norank_f__Anaerolineaceae	unclassified_g__norank_f__Anaerolineaceae	OTU356	0	0	0	0
Bacteroidetes	Bacteroidia	Bacteroidales	Porphyromonadaceae	Odoribacter	uncultured_bacterium_g__Odoribacter	OTU363	0	0	0	0
Bacteroidetes	Bacteroidia	Bacteroidales	Bacteroidales_S24-7_g	norank_f__Bacteroidales_S24-7_gro	uncultured_bacterium_g__norank_f__Bacteroidales_S24-7_group	OTU364	0	0	0	0
Bacteroidetes	Cytophagia	Cytophagales	Cytophagaceae	Persicitalea	uncultured_bacterium_g__Persicitalea	OTU373	0	0	0	0
Proteobacteria	Deltaproteobacteria	Myxococcales	BIrii41	norank_f__BIrii41	unclassified_g__norank_f__BIrii41	OTU385	0	0	0	0
Chloroflexi	unclassified_p__Chlorofl	unclassified_p__Ch	unclassified_p__Chlor	unclassified_p__Chloroflexi	unclassified_p__Chloroflexi	OTU394	0	0	0	0
Planctomycetes	Planctomycetacia	Planctomycetales	Planctomycetaceae	norank_f__Planctomycetaceae	unclassified_g__norank_f__Planctomycetaceae	OTU400	0	0	0	0
Nitrospirae	Nitrospira	norank_c__Nitrospi	norank_c__Nitrospira	Nitrospira	unclassified_g__Nitrospira	OTU402	0	0	0	0
Actinobacteria	Actinobacteria	Acidimicrobiales	OM1_clade	norank_f__OM1_clade	unclassified_g__norank_f__OM1_clade	OTU412	0	0	0	0
Firmicutes	Clostridia	Clostridiales	Lachnospiraceae	norank_f__Lachnospiraceae	uncultured_bacterium_g__norank_f__Lachnospiraceae	OTU418	0	0	0	0
Gemmatimonade	Gemmatimonadetes	Gemmatimonadale	Gemmatimonadaceae	Gemmatimonas	uncultured_bacterium_g__Gemmatimonas	OTU424	0	0	0	0
Chloroflexi	Thermomicrobia	JG30-KF-CM45	norank_o__JG30-KF-C	norank_o__JG30-KF-CM45	uncultured_Sphaerobacter_sp._g__norank	OTU428	0	0	0	0
Proteobacteria	Alphaproteobacteria	Rhodospirillales	Acetobacteraceae	norank_f__Acetobacteraceae	uncultured_bacterium_g__norank_f__Acetobacteraceae	OTU434	0	0	0	0
Actinobacteria	Actinobacteria	Coriobacteriales	Coriobacteriaceae	Coriobacteriaceae_UCG-002	uncultured_bacterium_g__Coriobacteriaceae_UCG-002	OTU436	0	0	0	0
Acidobacteria	Acidobacteria	unclassified_c__Aci	unclassified_c__Acido	unclassified_c__Acidobacteria	unclassified_c__Acidobacteria	OTU438	0	0	0	0
Chloroflexi	Ktedonobacteria	C0119	norank_o__C0119	norank_o__C0119	unclassified_g__norank_o__C0119	OTU439	0	0	0	0
unclassified_k__r	unclassified_k__norank	unclassified_k__no	unclassified_k__noran	unclassified_k__norank	unclassified_k__norank	OTU453	0	0	0	0
unclassified_k__r	unclassified_k__norank	unclassified_k__no	unclassified_k__noran	unclassified_k__norank	unclassified_k__norank	OTU620	0	0	0	0
unclassified_k__r	unclassified_k__norank	unclassified_k__no	unclassified_k__noran	unclassified_k__norank	unclassified_k__norank	OTU786	0	0	0	0

Figure S1. Correlation analyses between abundances of cecal bacteria and FI, BWG, eviscerated weight, T-SOD, breast meat weight, leg meat weight, abdominal fat weight, eyeball weight, MDA, GSH-Px, Mel, dressing weight, FCR on the level of genus. The red color represents a positive correlation while the green color represents a negative correlation. FI, feed intake; BWG, body weight gain; T-SOD, total superoxide dismutase; MDA, malonaldehyde; GSH-Px, glutathione peroxidase; FCR, (feed intake)/(body weight gain). * Means the correlation was significant ($p<0.05$).

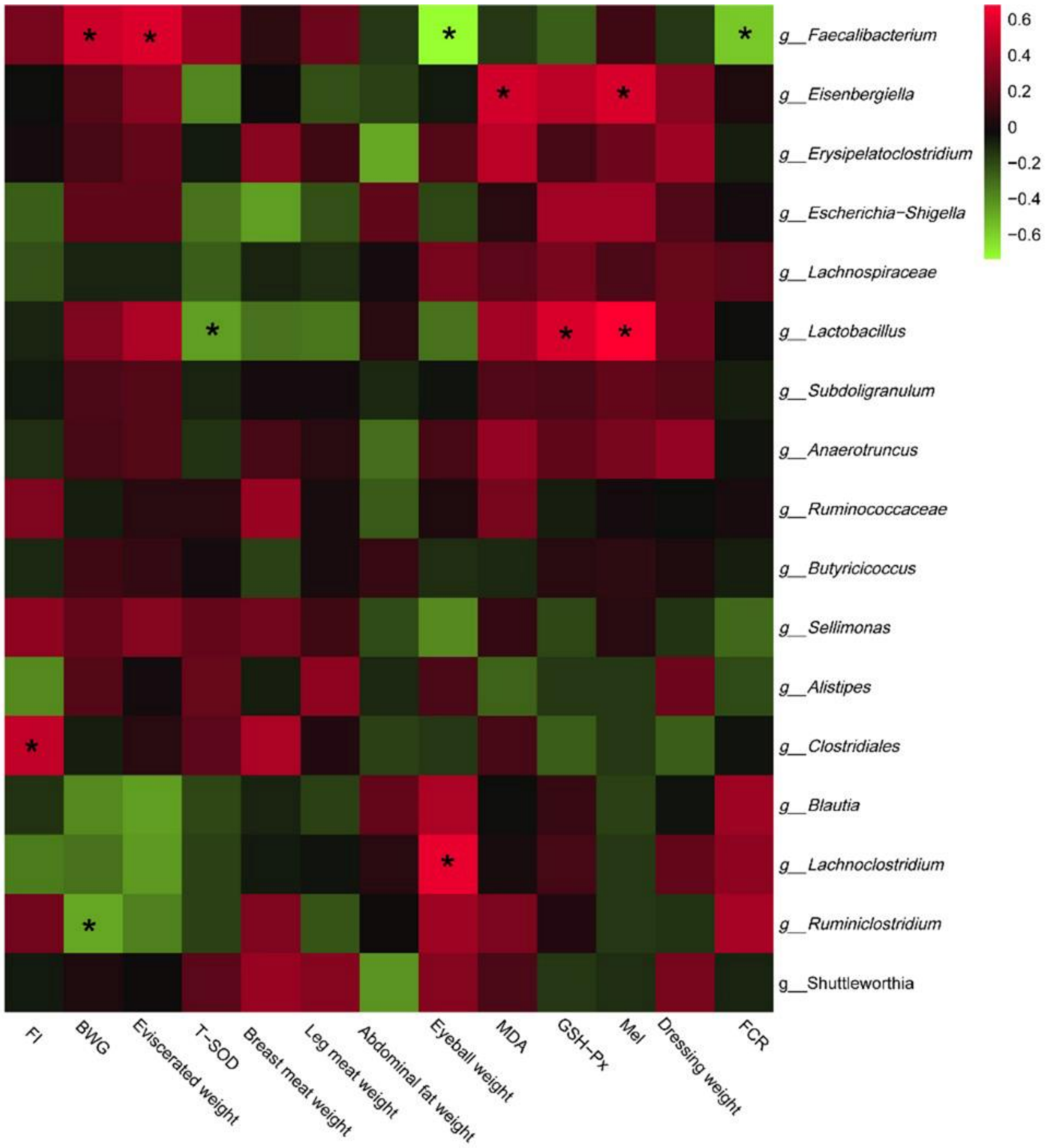
g__Faecalibacterium
g__Eisenbergiella
g__Erysipelatoclostridium
g__Escherichia−Shigella
g__Lachnospiraceae
g__Lactobacillus
g__Subdoligranulum
g__Anaerotruncus
g__Ruminococcaceae
g__Butyricicoccus
g__Sellimonas
g__Alistipes
g__Clostridiales
g__Blautia
g__Lachnoclostridium
g__Ruminiclostridium
g__Shuttleworthia
FI
BWG
Eviscerated weight
T-SOD
Breast meat weight
Leg meat weight
Abdominal fat weight
Eyeball weight
MDA
GSH−Px
Mel
Dressing weight
FCR
0.6
0.4
0.2
0
−0.2
−0.4
−0.6
